# Solvation and surface effects on polymorph stabilities at the nanoscale†
†Electronic supplementary information (ESI) available: Detailed procedure for the solid-state DCC model reaction, HPLC data, PXRD patterns, data and its corresponding Rietveld results for each of the solvent studies and together with discussion of methodology used for the Rietveld refinement and computational modelling. See DOI: 10.1039/c6sc03457h



**DOI:** 10.1039/c6sc03457h

**Published:** 2016-09-02

**Authors:** A. M. Belenguer, G. I. Lampronti, A. J. Cruz-Cabeza, C. A. Hunter, J. K. M. Sanders

**Affiliations:** a Department of Chemistry , University of Cambridge , Lensfield Road , Cambridge CB2 1EW , UK . Email: jkms@cam.ac.uk ; Email: amb84@cam.ac.uk; b Department of Earth Sciences , University of Cambridge , Downing St , Cambridge , CB2 3EQ , UK; c School of Chemical Engineering and Analytical Science , The University of Manchester , Oxford Road , Manchester , M13 9PL , UK

## Abstract

We explore the effects of particle size and solvent environment on the thermodynamic stability of two pairs of polymorphs subjected to ball-mill neat grinding (NG) and liquid assisted grinding (LAG). Two systems were studied: (i) forms I and II of a 1 : 1 theophylline : benzamide cocrystal and (ii) forms A and B of an aromatic disulfide compound. For both systems, the most stable-bulk polymorph converted to the metastable-bulk polymorph upon NG. LAG experiments yielded different outcomes depending on the amount of solvent used. This was further investigated by performing carefully controlled LAG experiments with increasing μL amounts of solvents of different nature. With these experiments, we were able to monitor form A to B and form I to II conversions as a function of solvent concentration and derive polymorph equilibrium curves. The concentration required for a switch in polymorphic outcome was found to be dependent on solvent nature. We propose that these experiments demonstrate a switch in thermodynamic stability of the polymorphs in the milling jar. Form B, the stable-bulk polymorph, has less stable surfaces than form A, thus becoming metastable at the nanoscale when surface effects become important. *Ex situ* diffraction and electron microscopy data confirm crystal sizes in the order of tens of nanometers after the ball mill grinding experiments reach equilibrium. DFT-d computations of the polymorph particles stabilities support these findings and were used to calculate cross-over sizes of forms A and B as a function of solvent. Attachment energies and surface stabilities of the various crystalline faces exposed were found to be very sensitive to the solvent environment. Our findings suggest that surface effects are significant in polymorphism at the nanoscale and that the outcomes of equilibrium ball-mill NG and LAG experiments are in general controlled by thermodynamics.

## Abstract 2

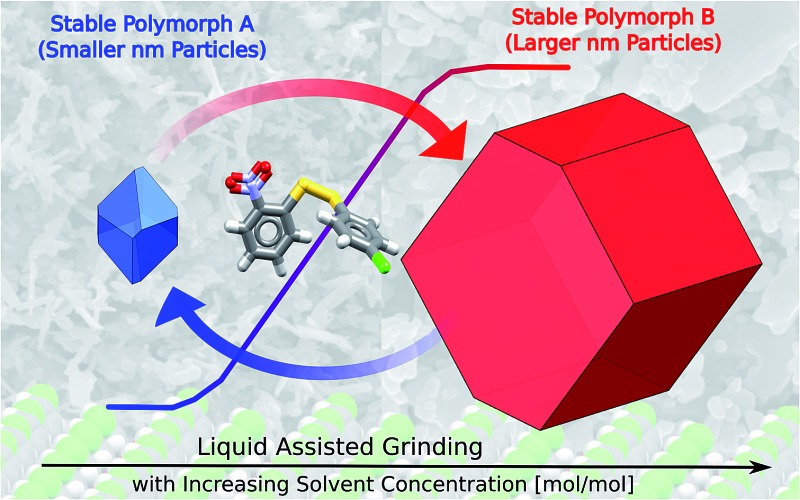
We explore the effects of particle size and solvent environment on the thermodynamic stability of two pairs of polymorphs subjected to ball-mill neat grinding (NG) and liquid assisted grinding (LAG).

## 1. Introduction

Polymorphism is the ability of a compound to crystallise in more than one crystal structure; it is a property inherent to the solid state.^2^ Structural differences between polymorphs may be significant or very subtle. For example, whilst forms I and II ritonavir differ in conformers,^3^ intermolecular interactions and crystal symmetry, forms I and II aspirin are so similar that they are very hard to distinguish and they can even grow together.^4–6^ As well as differences in the bulk structure, polymorphs might significantly differ in the structure and stability of the surfaces they expose as a consequence of many factors including the inherent crystallography, the solvent of crystallisation or the presence of impurities.^7^

Mechanochemistry using manual or ball mill grinding equipment is emerging as an attractive and sustainable synthetic tool.^8^ Beyond the many environmental advantages, mechanochemistry can enable access to new polymorphs that are otherwise inaccessible by classic solution methods. Liquid assisted grinding (LAG), a technique which consists of grinding solid materials in the presence of a few drops of solvent, has dramatically broadened the applications of mechanochemistry;^9^ LAG often accelerates reaction kinetics and sometimes yields different outcomes to neat grinding (NG) techniques.^10–12^

Ball mill grinding processes have been applied to the synthesis of organic,^13,14^ inorganic^15,16^ and supramolecular materials.^17,18^ Although there is growing interest in the pharmaceutical industry in mechanochemistry and its applications for the discovery of new polymorphs,^8^ ball mill grinding methods are not yet routinely applied in industry partly because of our lack of understanding of the underlying mechanisms and thermodynamics of grinding processes.^9^ Only a few systematic kinetic studies on mechanochemical processes and the role of solvent in LAG reactions have been reported to date.^11,12,19–24^ Some authors state that LAG provides greater molecular mobility than NG.^9^ It has also been suggested that the solubility of the reactants in the LAG solvent is critical^25^ since this plays a fundamental role in the wetting layer covering the particle surfaces.^11^ In an effort to shed some light in these mechanisms, solid form conversion under ball mill NG and LAG have recently been studied *in situ* by synchrotron powder X-ray diffraction (PXRD)^26,27^ and by Raman spectroscopy.^20,28^

**Fig. 1:**
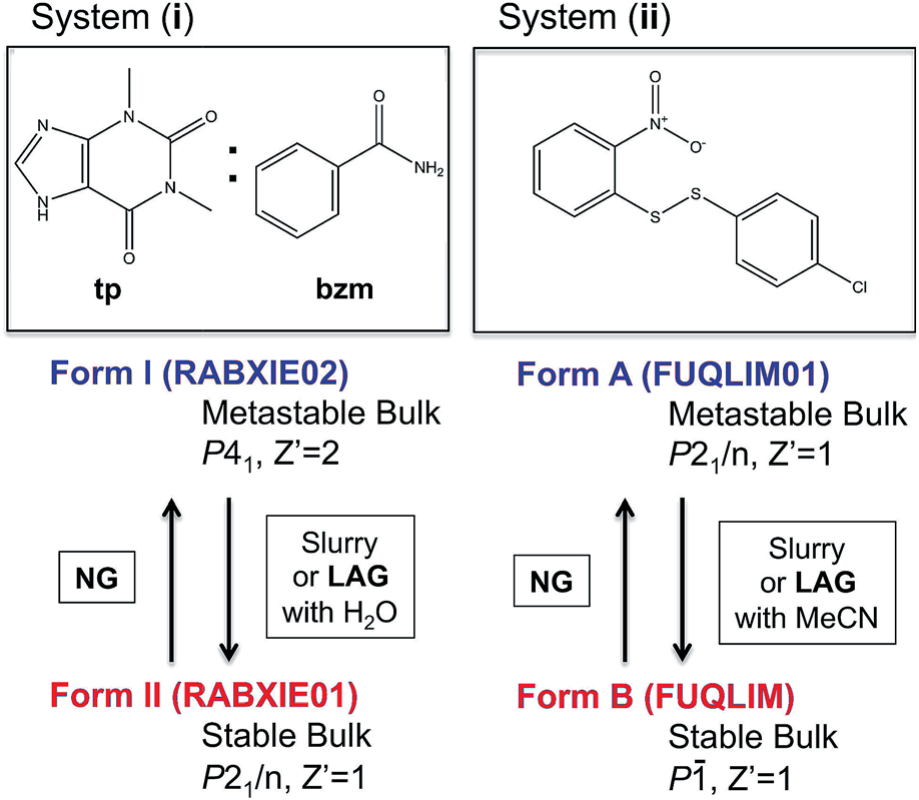
Molecular diagrams of systems (i) and (ii) and summary information on their polymorphs and methods for their interconversion. Cambridge structural database refcodes are given in parenthesis.^1^

Despite some of these recent mechanistic studies concerning NG and LAG, the thermodynamic aspects and the nature of equilibria under milling conditions have largely remained unexplored. Recently, we have systematically investigated the influence of milling conditions on polymorph outcomes and stabilities,^23^ work which we have further developed in the present contribution with a particular focus on the role of the surface. We present combined state-of-the art experimental and computational studies of the reversible interconversions of a pair of polymorphs for the cocrystal system (i) recently reported by Fischer *et al.* ^29,30^ and the single component system (ii) (Fig. 1) that we have been investigating for some time.^23^

## 2. Thermodynamics at the nanoscale

Unlike crystallisation in solution or a slurry experiment, ball mill grinding processes lead to extremely small crystals whose size is hard to predict or even estimate. One report of *in situ* time resolved PXRD monitoring of a ball mill grinding reaction describes the crystal size in a ball mill grinding reaction as approaching an average crystal size in the order of tens of nm, giving a very different surface to volume ratio (*S*/*V*) to that of microcrystals.^26^ Small crystallites have higher enthalpies and free energies than large crystals because of a positive surface energy.^31^ Moreover the polymorph with the thermodynamically most stable bulk structure at some given pressure and temperature is not necessarily the one with the most stable surface structure in those same conditions. Therefore, polymorphs that are metastable as micrometer-sized or larger crystals can be thermodynamically stable at the nanoscale.^31,32^ Surface to volume ratios are, of course, proportionally related to the number of molecules that a crystal has on the surface. For illustration purposes, we estimate the percentage of molecules at the surface of a spherical particle being 100% for 1 nm sizes, ∼40% for 10 nm, ∼4% for 100 nm, ∼0.4% for 1 μm and just ∼0.0004% for 1 mm sizes.‡
‡Theoretical estimation considering the molecules are also spheres of 7.5 Å radius packing with a packing efficiency typical of organic crystals (70%). Thus, while the classic thermodynamic literature on polymorphism conventionally assumes surface effects to be negligible (*i.e.* infinite bulk structures, as in the case of slurry experiments where crystals are in the mm length-scales), this is not the case in continuously mechanically ground systems where crystal size is decreased to the nm scales.^15^ These thermodynamic aspects are general, and must apply to any milling system, independent of the mechanisms and type of bond (ionic, covalent, metallic or supramolecular) involved in the chemical reaction. For clarity, we will refer to “stable-bulk” polymorph as the polymorphic form which has the most stable lattice (lower lattice free energy) at a given temperature and pressure. Likewise, the term “metastable-bulk” polymorph will be used to refer to a polymorph form with less stable lattice (higher lattice free energy) at a given temperature and pressure. In describing the polymorphic systems in this article, we will refer to the *R* index as the ratio of the stable-bulk polymorph to the total amount of the two interconverting polymorphs.

## 3. Polymorphs of system (i)

In two recent publications, Fischer and co-workers presented two polymorphs of the 1 : 1 cocrystal of theophylline (**tp**) with benzamide (**bzm**), form I and form II,^29,30^ the latter being the thermodynamically stable-bulk polymorph at ambient conditions as confirmed by slurry experiments (Fig. 1). The authors investigated the early kinetics (up to 25 minutes) of formation and the stabilities of these two polymorphs under milling conditions. Some seeds of the form II cocrystal were added to the original physical mixture of **tp** and **bzm** and it was concluded that, while the form I cocrystal could be obtained from the original components by NG or LAG with apolar solvents, form I was only a kinetic product; form II was always obtained, eventually, under any of their milling condition.

Prompted by these results, and as a test of the general conclusions reported below, we have studied the same co-crystal system for short and prolonged milling times, up to 24 hours. We demonstrate that particles of form I (metastable-bulk) are in fact thermodynamically stable under NG conditions and under LAG conditions using cyclohexane, while particles of form II (stable-bulk) are the stable ones under LAG with water (see Fig. 1). Form II can also be obtained with acetonitrile, ethanol and acetone (see ESI, Sections 4.6 and 14†), as reported by Fischer *et al.* ^29^ Furthermore, by using total loads of 200 mg or 1 g in the experiments, we show how the amount of material affects the milling reaction kinetics. In order to understand how the relative stability of particles of forms I and II change as a function of water concentration under LAG conditions, we monitored the molar ratio of these forms once the grinding experiment reaches equilibrium. These milling experiments were performed in the presence of water at concentrations ranging from units of μL to 250 μL per 1 g of total powder.

## 4. Polymorphs of system (ii)

In earlier work,^10,23^ we investigated the solid-state reaction between bis-2-nitrophenyldisulfide and bis-4-chlorophenyldisulfide in the presence of a small amount of base catalyst (dbu)§
§The base catalyst used is 1,8-diazabicyclo[5.4.0]undec-7-ene (dbu). to produce the compound 4-chlorophenyl-2-nitrophenyl-disulfide (compound (ii)) upon ball mill NG and LAG. Our experiments gave rise to the discovery of two polymorphs for system (ii), forms A and B (Fig. 1). These two forms can be obtained in two ways: (a) starting from the original reactants *via* the breaking and making of S–S bonds under various grinding conditions and in the presence of dbu; (b) once compound (ii) is quantitatively obtained and recrystallised, NG or LAG of compound (ii) can reversibly lead to forms A and B by varying the grinding conditions in the presence or in the absence of dbu.¶
¶The presence of 2% M of dbu base catalyst cannot change the relative thermodynamic stabilities of form A and B – see also Section 6.2. Just like with system (i), the resulting form of compound (ii) after the grinding experiment only depends on the grinding conditions and not on the starting phase composition. Methods (a) and (b) lead to the same crystalline form of compound (ii) if the same experimental conditions for grinding are employed (see ESI Section 6 and 7 for more details†). As a matter of convenience, however, we have mostly employed method (a) to produce forms A and B of system (ii) because equilibrium upon grinding is reached more quickly. We provide extensive proof in the ESI† on how both methods always lead to identical results.

We have demonstrated previously that, despite form B being the stable bulk (Fig. 1), form A is the thermodynamic product under NG conditions whilst form B is the thermodynamic product under LAG conditions with 50 μL of acetonitrile. Our conclusions are an inevitable consequence of the fact that the polymorphs transform into each other when the experimental conditions are changed and that no further change in the equilibrium composition is observed after up to 24 h of continuous grinding.^23^

In order to understand how the relative stability of particles of forms A and B change as a function of solvent concentration under LAG conditions, we have monitored the molar ratio of these forms once the grinding experiment reaches equilibrium.‖
‖A grinding experiment reaching equilibrium means that grinding the system for longer times will not change the composition and nature of the products. If further grinding times affect the outcome (in this case being the amounts of forms A and B), this means the system had not reached equilibrium under those particular milling conditions. All the milling experiments were performed in the presence of solvent at concentrations ranging from a few to tens of μL per 200 mg of total powder using method (a) as described above. For low solvent concentrations form A is more stable under ball-mill grinding conditions than form B; for high solvent concentrations form B is more stable than form A. This work constitutes the first systematic investigation on the effect of solvent nature and concentration on the solid form outcomes of ball-mill LAG experiments at equilibrium.

## 5. Methods

### 5.1. Experimental methods

For system (i), the procedure described by Fischer *et al.* ^29^ was used to prepare 1 : 1 **tp** : **bzm** co-crystal in forms I and II. For system (ii), dbu-free form B was quantitatively prepared by crystallizing compound (ii) after its synthesis by means of method (a). dbu-free form A was then quantitatively prepared by milling dbu-free form B under NG conditions for up to 2.5 hours, *i.e.* using method (b).

Slurry experiments were performed to determine the thermodynamic stability of both pairs of polymorphs under ambient conditions. For this, supersaturated solvent suspensions including 1 : 1 mixtures of polymorphs were stirred until the kinetic polymorph had fully converted to the stable form. These slurry experiments were performed in water for system (i) (see ESI Section 14.10†) and in acetonitrile for system (ii) (see ESI Section 11†). For both systems, equilibrium was reached within a week.

Ball-mill grinding polymorph stability experiments were performed using snap close milling jars together with stainless steel ball bearings of either 7 mm (200 mg loads) or 10 mm diameter (1 g loads). For system (i), a 1 : 1 mixture of forms I and II was ground under NG as well as LAG conditions. For system (ii), pure forms A or B were ground under NG as well as LAG conditions. For the LAG grinding, either 50 μL (200 mg loads) or 250 μL (1 g loads) of solvent were added to the milling jars. Jars were carefully sealed and milling was performed at 30 Hz on a MM400 Retsch automated grinder for up to 24 hours. The ball mill grinder was programmed to achieve the total grinding times in consecutive 60 minute runs separated by 10 minute intervals. The grinder shielding was removed and replaced with an external safety shield in order to prevent the ventilation system of the motor from heating the milling jar over prolonged milling times. Immediately after completion of each grinding experiment, jars were opened and samples were analysed using PXRD and specimens were taken for scanning electron microscopy (SEM).

For system (ii), form A/B equilibrium experiments as a function of the solvent concentration were performed starting from the original reactants as described above as method (a). A total of 200 mg of equimolar quantities of the disulfide starting materials were added to a 14.5 mL stainless steel screw-closure milling jar together with two 7 mm diameter stainless steel ball bearings. After the addition of 2 μL (2% M) of dbu catalyst into the jar, the solvent was added in a known quantity ranging between 0 and 200 μL measured by direct or inverse pipetting depending on the solvent. We used sixteen different solvents, namely acetonitrile (MeCN), acetone, dimethylformamide (DMF), tetrahydrofuran (THF), ethyl-acetate (EtOAc), chloroform (CHCl_3_), methanol (MeOH), ethanol (EtOH), isopropanol (IPA), dichloromethane (DCM), dimethylsulfoxide (DMSO), water, benzene, toluene, cyclohexane and perfluorodecalin. The experimental procedure used ensured that all the solvent is in close contact with the powder (using pre-soaking if necessary) before grinding is started. The jars were closed and sealed with Teflon washer covers, and milling was performed until equilibrium was achieved. The sealing avoids evaporation of the grinding solvent and was found to be essential for accurate reproducibility of results. Immediately after completion of grinding, jars were opened and samples analysed using high performance liquid chromatography (HPLC) and PXRD. The results are presented making use of equilibrium curves in which the *R* index is plotted *versus* solvent concentration (in moles of solvent per mole of compound (ii)). Each equilibrium curve required tens of individual grinding experiments at varying solvent concentration. The *R* index was derived from quantitative PXRD analysis.

For each solvent, equilibration times were assessed by means of kinetic experiments using the methodology described previously.^23^ Chemical equilibrium was achieved when compound (ii) was obtained quantitatively and the chemical composition of the solid (analysed by HPLC) remained constant upon further milling. A few experimental conditions were tested for phase equilibration with both HPLC and XRD (see ESI Section 9†). The agreement between HPLC and XRD was found to be excellent (see ESI†) as in our previous work.^23^ The exact number of independent milling experiments required for the definition of the curves was determined by the form A to B transition profile (which is different in different solvents). Different experimental precautions had to be taken for different solvents (see ESI Section 4.5†). The solid-state composition of the samples, reported here as % M, was determined by Rietveld refinements from powder XRD data (estimated accuracy: ±5% M absolute; estimated sensitivity as limit of detection (LOD): 3% M). The solubility of forms A and B of compound (ii), as well as those of the initial reactants, were measured for fifteen of the sixteen solvents used (see ESI Sections 4.7, 10 and 12†).

For system (i), form I/II equilibrium experiments as a function of the water concentration were performed in a similar fashion as form A/B equilibrium experiments, starting from form I added to a 14.5 mL stainless steel screw-closure milling jar together with two 10 mm diameter stainless steel ball bearings. The resulting equilibrium curve is plotted as *R* index *versus* water concentration (in moles of water per mole of co-crystal).

### 5.2. Computational methods

Density Functional Theory with van der Waals corrections (DFT-d) was used for crystal geometry relaxations of forms A and B of system (ii). Structural relaxations were performed allowing all possible structural parameters to freely optimise. The PBE functional^33^ was used with PAW pseudopotentials^34,35^ and the Grimme's van der Waals corrections (d2)^36^ as implemented in the VASP code.^37–40^ The Brillouin zone was sampled using the Monkhorst–Pack approximation^41^ and a variety of *k*-point grids with increasing number of *k*-points until the form energies converged (see ESI Section 2.2†). Structural relaxations were halted when the calculated force on every atom was less than 0.003 eV Å^–1^. Lattice energies (*E*_latt_) for both forms were calculated by subtracting the electronic energy of a single molecule in the gas-phase from the electronic energy of the simulation cell in the crystal divided by the number of molecules (*N*).

Slices of the most important morphological faces for each form were generated from the DFT-d optimized structures. Four main surfaces were considered for each form plus variations of those due to choices of origin (see ESI Section 2.5†). A vacuum slab of at least 15 Å was build above the slices to construct a 3D periodic supercell. Then, a single point energy calculation of the cut crystal slice was performed using the same DFT-d model in the presence of a dielectric continuum with the VASPsol module.^42,43^ Slice energies per molecule (*E*_slice_, see ESI†) were calculated for dielectric constants typical of vacuum (*ε* = 1), acetone (*ε* = 20), acetonitrile (*ε* = 40), a 1 : 1 acetonitrile : water mixture (*ε* = 60) and water (*ε* = 80). Once the slice energies were computed in different solvents, attachment energies (*E*_att_) were then calculated as the difference between the lattice energy and the slice energy (eqn (1)). Finally, surface energies were estimated as the sum of the slice energy plus half of the attachment energy (eqn (2)).

1 *E*_att_ [*hkl*] = *E*_latt_ – *E*_slice_ [*hkl*]

2 *E*_face_ [*hkl*] = *E*_latt_ + 0.5*E*_att_ [*hkl*]

Predicted growth morphologies for forms A and B were then derived making use of the computed attachment energies in the different solvents. These morphologies were then used for the estimation of particle shape, particle aspect ratios and particle surface/volume ratios. Making use of these, a python algorithm was written to calculate the stability of forms A and B particles as a function of size. We refer to size as the smallest dimension of a length × width × thickness crystal. Crystals of different sizes were simply built by multiplying the particle size under consideration by the crystal aspect ratio which was derived from the growth morphology in a given solvent. The stability of each particle for a given size and solvent environment was then computed using eqn (3) in which *N*_bulk_, *N*_face_[*hkl*] and *N*_particle_ are the number of molecules in the particle placed in the bulk, in an *hkl* face and the total number of molecules and *E*_latt_ and *E*_face_[*hkl*] are the lattice energy and the surface energy of the *hkl* face. Using this approach, we were able to derive form A and B particles stability plots as a function or size for the five solvents considered.3
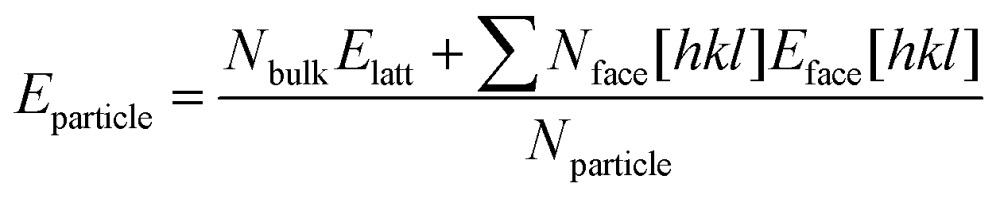



## 6. Results

### 6.1. System (i)

**Fig. 2:**
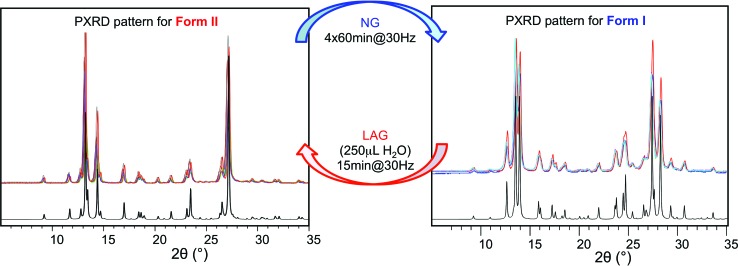
Superimposed powder diffractograms demonstrating polymorph conversions in system (i) under NG and LAG conditions with 250 μL of water. The “turnover” polymorph conversion experiment has been repeated 5 times. Calculated patterns for form II (left) and form I (right) are shown in black.

Polymorph equilibrium experiments were performed starting from an equimolar mixture of forms I and II. LAG experiments confirmed the observations of Fischer *et al.*:^29^ form II is always obtained in the presence of water, acetone, ethanol or acetonitrile after sufficient grinding time (see ESI Section 4.6 and 14†). By contrast, under NG conditions or under LAG conditions with cyclohexane form I is always obtained. We present results that demonstrate form I is quantitatively transformed into form II under LAG conditions with 250 μL of water per 1 g of powder, while form II is quantitatively transformed into form I under NG conditions. Fig. 2 shows a summary for these “turnover” polymorph conversion experiments (*i.e.* experiments where the same sample is converted a number of times from polymorph I to II and back to I).

**Fig. 3:**
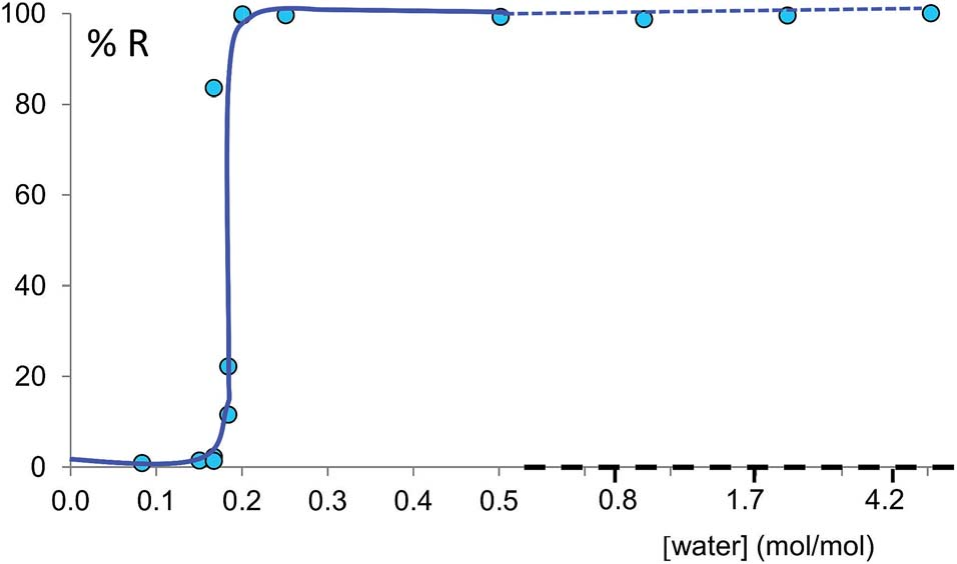
Experimental milling equilibrium curve plotted as water concentration *versus R* index [form II]/([form I] + [form II]), in %. No fitting was performed and the curve drawn is only a guide to the eye.

**Fig. 4:**
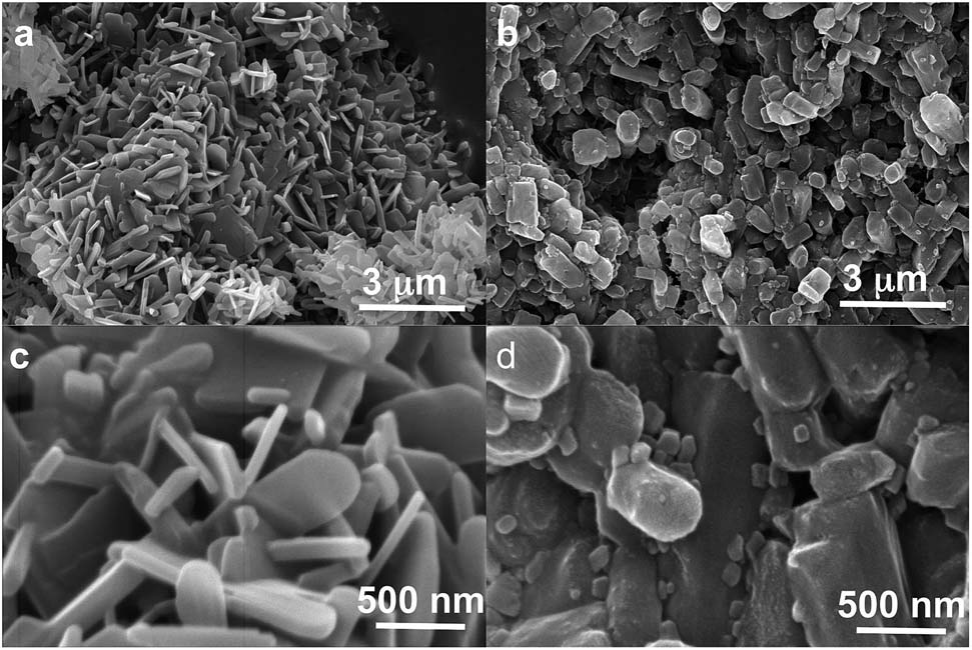
SEM images of powders of form I as obtained under NG conditions (left, a and c) and powders of form II as obtained under LAG conditions (right, b and c) at two magnifications (30 000× upper and 150 000× lower).

The discrepancy with results reported by Fischer *et al.* can be explained by the low water concentration required for the stabilities of the two polymorphs to be reversed. The water concentration *versus R* equilibrium curve reported in Fig. 3 shows that this threshold is at a water concentration of 0.2 mol mol^–1^ corresponding to 10 μL g^–1^. Form I and II crystals had to be kept in a dry atmosphere overnight in order to achieve consistent results. In fact, there is a narrow solvent concentration window between 10 and 12 μL g^–1^, where both polymorphs are present at equilibrium. As the same effect is seen for a few solvents in system (ii) (see Section 6.2), this seems likely to be a common or universal aspect of ball mill LAG conditions. Our kinetic studies and prolonged experiments (up to 24 hours) were also essential to establish the equilibria. Our SEM analyses (Fig. 4a and c) suggest that under milling conditions the crystallites of form I approach an equilibrium average size as small as tens of nm in at least one crystal direction. The images show that whilst form I crystallites are platelets with thickness smaller than 100 nm, form II crystallites have an equant shape with a wide size distribution from tens to hundreds of nanometers. Similar observations have been reported for some MOF materials that were studied under milling conditions *in situ* at synchrotron facilities.^26^

### 6.2. System (ii)

**Fig. 5:**
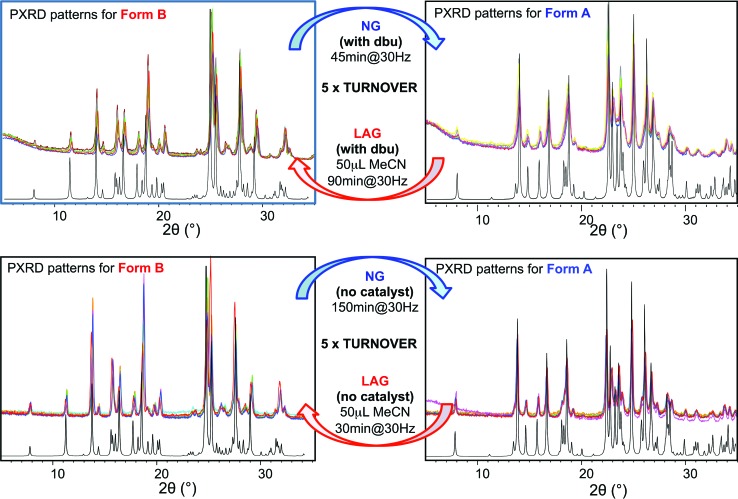
Superimposed powder diffractograms demonstrating polymorph conversions in system (ii) under NG and LAG conditions with 50 μL of acetonitrile, with method (b) (top) with dbu and (bottom) without dbu. The “turnover” polymorph conversion experiment has been repeated 5 times with dbu, 5 times without dbu. Calculated patterns for form B (left) and form A (right) are shown in black.

For system (ii), we have previously reported that form A is the stable polymorph under ball mill NG conditions, whilst form B is the stable polymorph under ball mill LAG conditions with 50 μL of acetonitrile.^23^ We first present results that demonstrate form A is quantitatively transformed into form B under LAG conditions with 50 μL of acetonitrile, with or without 2% M of dbu base catalyst (method (b), see Section 5.1); by contrast, form B is quantitatively transformed into form A under NG conditions (method (b)), with or without 2% M of dbu base catalyst. The presence of 2% M of dbu base catalyst allows for the disulfide bond to break and reform, changing the kinetics and the nature of the intermediate products but it cannot change the relative thermodynamic stabilities of form A and B. Fig. 5 shows a summary for these “turnover” polymorph conversion experiments (*i.e.* experiments where the same sample is converted a number of times from polymorph B to A and back to B) performed in the absence and presence of dbu. The turnover demonstrates conclusively that the nature of the polymorph of compound (ii) under milling conditions is determined by the presence or absence of 50 μL of acetonitrile in both cases. Kinetic studies for the thermodynamically driven polymorph interconversion between form A and form B and *vice versa*, with and without dbu, are documented in the ESI (Section 6.3 and 7.3 respectively†).

Moreover, we present further results that demonstrate that form A is transformed into form B under LAG conditions and that this transformation is dependent on the amount of solvent used. The proportion of polymorphs A and B present at equilibrium was assessed by PXRD and analysed effectively as a titration curve by plotting solvent concentration against *R*.

**Fig. 6:**
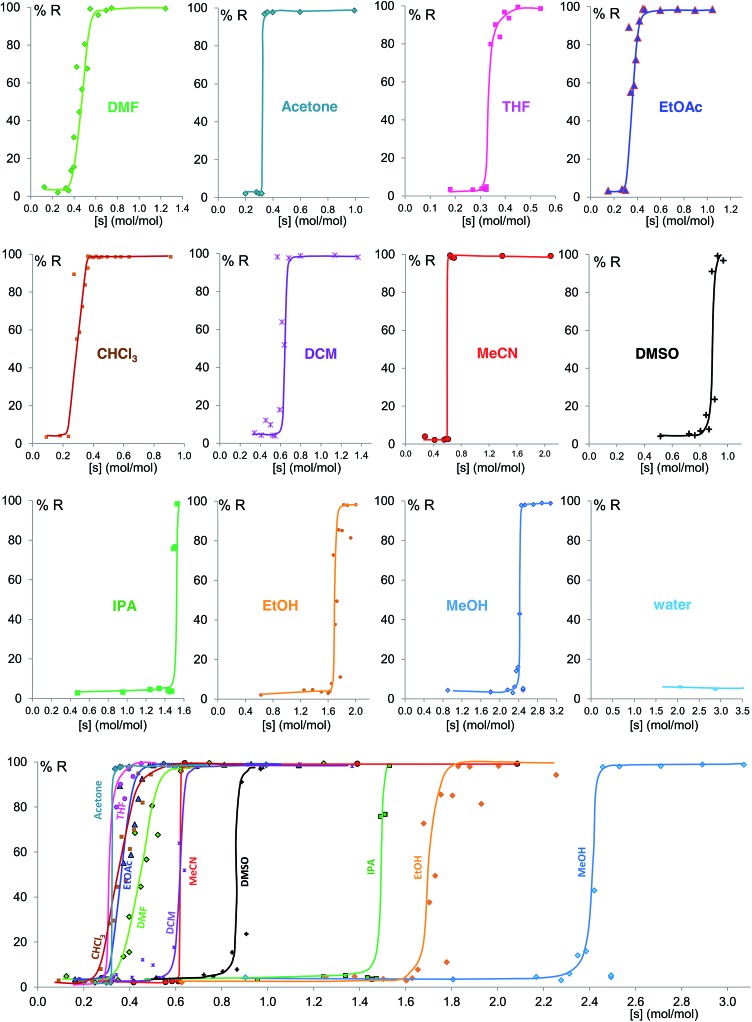
Experimental milling equilibrium curves plotted as solvent concentration *versus R* index [form B]/([form A] + [form B]), in %. No fitting was performed and the curves drawn are only a guide to the eye.

Fig. 6 shows the equilibrium results for twelve of the sixteen solvents as %*R versus* solvent molar concentration (mol of solvent/mol of solid) (see ESI Section 8†). No fitting was performed and the curves drawn are only a guide to the eye. While most of these solvents give form B at equilibrium above a certain solvent concentration, the curves vary in onset values and slopes. Most solvents stabilize form B at solvent concentrations of 0.3 to 0.7 mol mol^–1^, while the alcohols examined require solvent levels of 1.5 to 2.5 mol mol^–1^. For the most effective solvents (acetonitrile and acetone) the transition from form A to form B takes place over an extremely small concentration range (0.02 mol mol^–1^), while for others there is a significant solvent concentration range (0.06 to 0.26 mol mol^–1^) where the two polymorphs co-exist. For some of the solvents here studied which wet the crystals inefficiently, such as DMSO and the alcohols, the equilibrium curve remains noisy, although its position and shape are clear; we are currently exploring different experimental procedures, such as better solvent and powder pre-mixing methodologies, to reduce this problem.

**Fig. 7:**
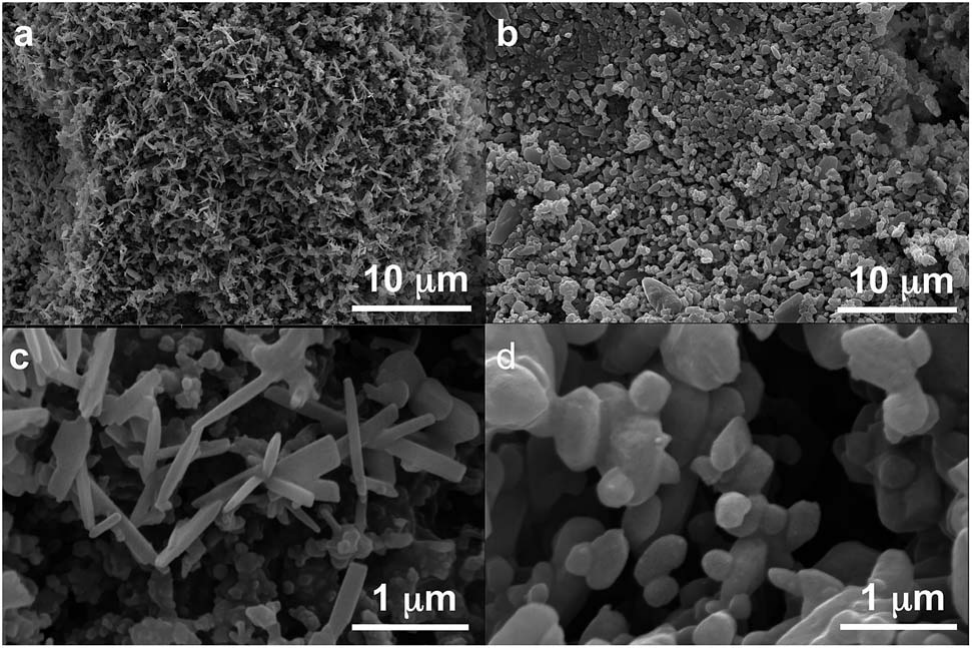
SEM images of powders of form A as obtained under NG conditions (left, a and c) and powders of form B as obtained under LAG conditions in acetonitrile (right, b and d) at two magnifications (10 000 upper and 100 000 lower).

We believe that these equilibrium curves are the result of the interaction of solvent molecules with the surfaces of form A and form B particles: below a solvent concentration threshold, which is specific for the solvent used, the solvated form A crystallites are more stable than the solvated form B crystallites; above this threshold the equilibrium is reversed. We estimate, based on *ex situ* diffraction data (using the Scherrer equation, see ESI Section 13 for details†) and SEM observations (Fig. 7), that in these experiments crystallite sizes are in the order of tens of nm, often around 70 nm, in at least one crystallographic direction.

**Fig. 8:**
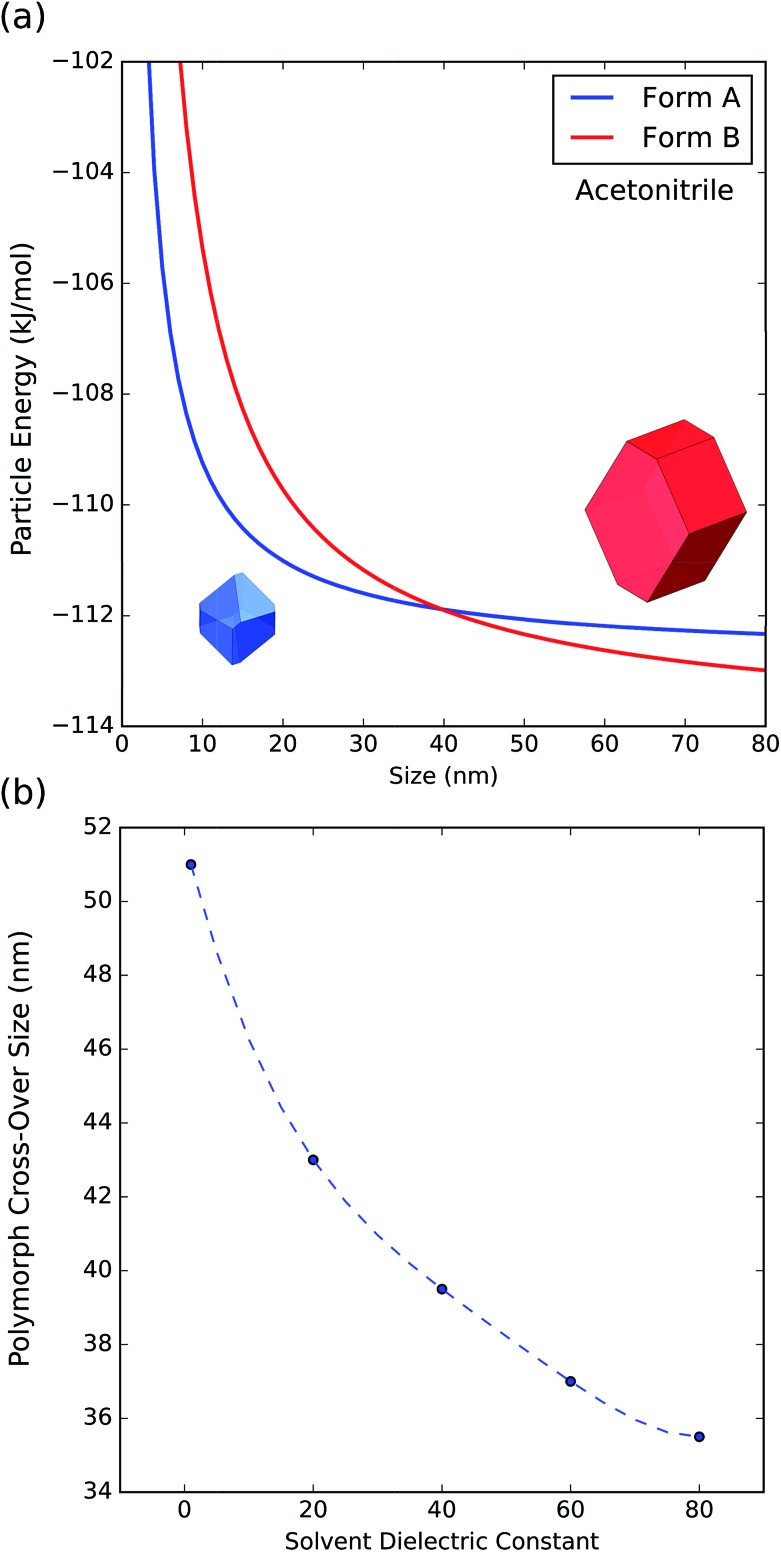
(a) Energy of particles of forms A and B as a function of size in acetonitrile. (b) Change of polymorph cross-over sizes as a function of solvent dielectric.

To further understand the effect of size and solvation on polymorph stability switches, we calculated the stability of particles of forms A and B as a function of size by means of DFT-d calculations. As it can be seen in Fig. 8a, in acetonitrile, form A is the most stable for particles below 41 nm in size and form B is the most stable for particles above 41 nm. Computation of form stability as a function of size for five different solvent environments resulted in the calculation of five cross-over sizes. As the solvent dielectric constant is increased, the surface stabilization effect is relatively more important in form B than in form A. This results in a reduction of cross-over sizes as a function of solvent dielectric (Fig. 8b).

## 7. Discussion

NG and LAG have been used in the past for accessing new polymorphic forms in an unsystematic way and so the mechanistic basis for these observations has been unclear until now. Here, we have demonstrated with improved experimental set-ups for LAG and state-of-the art computational techniques, that particle size and solvent environment have a profound effect on the stability of polymorphic crystallites.

Grinding experiments under prolonged milling times afford thermodynamic products. These may consist of different polymorphs depending on the achieved particle size. Further, the presence of solvent has a dual effect: it stabilises certain surfaces in the crystallites and it provides a medium for dissolution, nucleation and further growth.

The effect of particle size on polymorph stabilities has long been understood in the crystal growth community in the context of nucleation clusters. Nucleation, being a balance between thermodynamics and kinetics, can result in the observation of metastable forms because their energy barrier for nucleation is lower than that of the thermodynamic stable forms. Simulations of polymorphic clusters up to a few hundreds of molecules have been performed by Hammond, Pencheva and Roberts for l-glutamic acid^44^ and benzophenone.^45^ The authors reported a change of polymorph stabilities as a function of cluster size.

Beyond nucleation clusters and into the nm scales, Navrotsky and co-workers have shown similar results in the last couple of decades for inorganic materials.^31,46^ Crystallisation in nanoconfinement has also shed light into this: Ward *et al.* have reported polymorph stability switches for systems such as pimelic acid, glutaric acid, suberic acid and coumarin amongst others^47,48^ when crystallised in a nanoconfined environment.^49,50^ They also report how these confined nanocrystals display “melting point depression, polymorph stability crossovers and transitions from enantiotropic phase behavior in the bulk to monotropic behavior in nanoconfinement”.^47^ We have arrived at the same conclusions from grinding experiments supported by Scherrer size estimations, SEM images and DFT-d calculations of particles as a function of size.

Beyond the stability change as a function of size, Navrotsky and other authors also pointed out that solvation of surfaces might also have an effect on the stability of nanocrystallites.^31,46,51,52^ We have shown, for system (ii), that the type and amount of solvent clearly determines the polymorphic outcome of the LAG experiment. Different solvents required of different solvent/solute concentrations for reaching the polymorphic crossovers as shown by our polymorph equilibrium curves. This conclusion is also supported by the theoretical calculations which indicate changes in the cross-over sizes as a function of solvent dielectric.

The interpretation of the shape of the polymorph equilibrium curves may be a matter of further discussion and experiment. The shape of these curves may be interpreted in terms of cooperativity of solvent binding at the surfaces. If we assume crystallites with a diameter of tens of nm and one to two adsorption sites per square nm, we estimate thousands of adsorption sites per crystallite. According to the classification by Hunter and Anderson,^53^ in a system with such a large number of adsorption sites *n*, if the adsorption reactions have strong positive cooperativity, then the many potential states in which the adsorption sites are only partially occupied given by the large *n* are never populated, and a two-state all-or-nothing behavior is observed, *i.e.* the equilibrium curve presents an almost vertical slope. This is the case for acetone and acetonitrile, providing the experimental evidence (a) that there must be an interaction between the nanocrystal surfaces and the solvent molecules at equilibrium and (b) that this interaction contributes significantly to the surface energy and thus the polymorph relative stabilities.

**Fig. 9:**
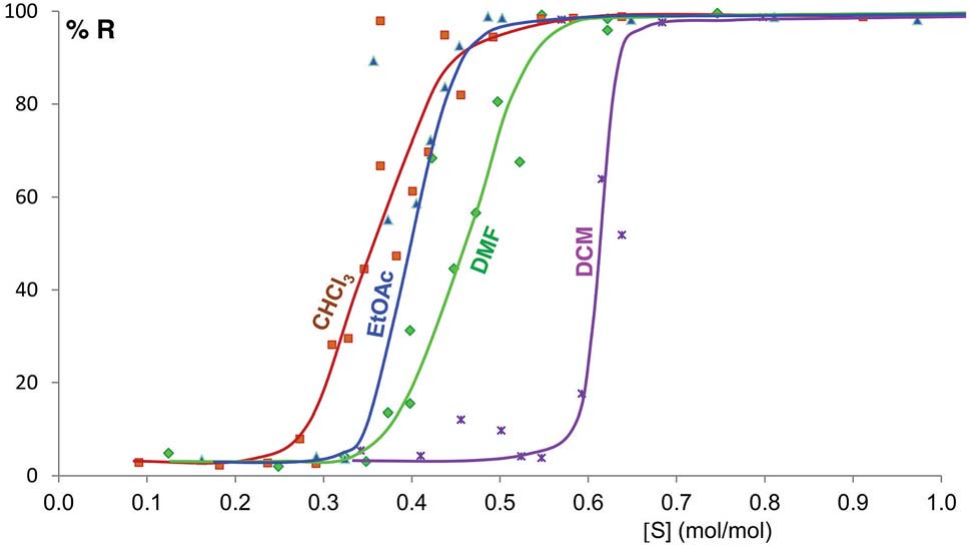
Experimental milling equilibrium curves showing non infinite slope plotted as solvent concentration *versus R* index [form B]/([form A] + [form B]), in %.

Some of these equilibrium curves show a shallower slope, *e.g.* ethyl acetate, DMF, DCM and CHCl_3_ (see Fig. 9). This indicates that another phase in which the adsorption sites are only partially occupied is formed; in other words, the equilibrium transition between form A and form B when increasing the solvent concentration involves a third phase. As reported in the ESI,† form B is relatively soluble in all four solvents that show a relatively shallow slope. We suggest that this third state in which the adsorption sites are only partially occupied must involve the solvent and it must be a non crystalline phase since we do not detect any other crystalline phase by diffraction. This could be an amorphous form with adsorption sites only partially occupied or an amorphous solvate, as possibly suggested by diffraction analyses (see ESI Section 5.4†), or a liquid phase, although the latter seems unlikely considering the solubility values reported in the ESI Section 10† and the amount of solvent used in the LAG experiments.

With very polar (water, see ESI Section 8.15†) and very apolar (cyclohexane, benzene, toluene and perfluorodecalin, see ESI Section 8.16–8.19†) solvents ball mill LAG experiments do not yield form B in the concentration ranges that that we studied, although it is possible that these solvents could yield form B at higher concentration. This hypothesis could not be explored as the kinetics of the DCC reaction becomes too slow at high solvent concentrations. We have shown how both ball mill NG and ball mill LAG reactions exhibited a nucleation phase (around 5 minutes) and a sharp transition before reaching a constant plateau in our previous paper.^23^ Evidently, the nucleation phase must be extended the more solvent is added. This is unexpected as reactions under LAG conditions are generally reported to be faster than under NG conditions.

In effect, we have experimentally explored three polymorphic systems rather than two: system (i), a co-crystal polymorphic system with supramolecular interactions only; system (ii) using method (b) in the absence of dbu catalyst, a single component system with supramolecular interactions only; system (ii) using methods (a) and method (b) in the presence of dbu, a dynamic covalent system where both supramolecular and covalent bonds are involved in the polymorph conversion. Our interpretations, supported by extensive computational evidence in the case of system (ii), can explain all these three cases independently of the type of chemical interaction involved. Thus the conclusions we draw here must be general.

## 8. Conclusions

The thermodynamics of polymorphs depend on the crystal size because of the effect of the surface energies. The surface energy is affected by the nature and concentration of the solvent. These effects only become significant for nanosized crystals, which is why they become visible in mechanochemistry. In this sense, mechanochemistry is a powerful tool for exploring polymorph stabilities at the nanoscale. We have demonstrated that polymorph relative stabilities can change depending on the presence (or absence), nature and concentration of solvent under milling conditions, using two different molecular mechanochemical systems – one involving only supramolecular interactions, one that also involves covalent chemistry.

We have presented here for the first time results that show the influence of solvent on the polymorph stabilities at equilibrium under ball mill LAG conditions. We were able to define the equilibrium curves here presented by using sealed milling jars and exploring the solvent concentration over a wide range and with a resolution of 1 μL when necessary – an approach that has not previously been used in any mechanochemistry system. These rigorous experimental and analytical procedures and precautions have been found to be crucial in order to obtain these results. We believe similar equilibrium curves as a function of solvent concentration can be in principle obtained for any polymorphic milling system.

The polymorph relative stabilities reverse over a concentration range that varies from solvent to solvent. Some of the equilibrium curves here presented are very sharp, showing an “all-or-nothing” behaviour. This is characteristic of particles with a large number of adsorption sites and positive cooperativity of the binding process. This provides experimental evidence that there must be an interaction between the nanocrystal surfaces and the solvent molecules at equilibrium and that this interaction contributes significantly to the surface energy and thus the polymorph relative stabilities. Shallower curves indicate a lower level of cooperativity and the presence of a third phase.

We have computationally shown the importance that the surface structure has in determining the energy of the system. Our interpretation is that the relative stability of its surface energy could be the reason why the metastable-bulk polymorph becomes the thermodynamic form under specific milling conditions. The fact that surface energy is significant for polymorph stabilities at the nanoscale has been known and studied experimentally in the last couple of decades by Navrotsky and co-workers.^31,46^ We have shown here that the concept is general.

In order to establish which is the stable polymorph under specific milling conditions, a series of milling experiments has to be performed where a mixture of the two polymorphs is milled for different times, and the result has to be either monitored *in situ* or immediately analysed by diffraction. The approach is analogous to slurry experiments, *i.e.* experiments where a supersaturated solvent suspension including all the polymorphs that co-exist at ambient conditions is stirred until all polymorphs but the thermodynamic one disappear. However, sequential and delayed reactions/transformations may occur, and prolonged experiments (up to 24 hours) were essential to the definition of equilibria. Furthermore the utmost experimental rigour is essential, since a tiny amount of solvent can determine the final outcome. Finally, careful phase quantification by powder diffraction data analysis is necessary due to the presence of equilibrium phase mixtures.

We believe that these findings will be common to many systems under ball mill grinding conditions. Small molecule polymorphs generally differ in lattice energy by less than 4 kJ mol^–1^ (our two polymorphs differ by 1.3 kJ mol^–1^ according to our calculations),^10^ and mechanochemistry has been described as one of those crystallization tools able to obtain those polymorphs that are metastable under room conditions.^54^ This is because the stability order can be reversed by milling in the presence of solvent: polymorphs that are metastable at room conditions can be stable under milling conditions due to the nanocrystal surface–solvent interaction contributing to the polymorph free energy. Therefore this work suggests that polymorphism, far from being a rare event or phenomenon, will actually prove to be very common, in LAG ball-mill mechanochemistry.

This work lays a more secure foundation and understanding for the design and investigation of future mechanochemical systems. Systems that show two or more polymorphs with similar bulk energies and morphologically important surfaces of different nature at ambient conditions are good candidates for this kind of studies since their relative stabilities are more likely to be reversed by surface effects at high S/V ratio. Finally stabilities should be explored under LAG conditions with a range of different solvents and concentrations.
