# Supplementary material for: Solvation and surface effects on polymorph stabilities at the nanoscale
Source: Chem Sci. 2016 Sep 2;7(11):6617–27. doi: 10.1039/c6sc03457h (PMC5450528; doi:10.1039/c6sc03457h)
Supplement: Supplementary file 1 [file SC-007-C6SC03457H-s001.pdf]

Electronic Supplementary Material (ESI) for Chemical Science.
This journal is © The Royal Society of Chemistry 2016

Supplementary Material

Solvation and Surface Effects on Polymorph Stabilities at the Nanoscale

Ana M. Belenguer[1*‡], Giulio I. Lampronti[1,2‡], Aurora J. Cruz-Cabeza,[3] Christopher A. Hunter,[1] and Jeremy K. M. Sanders[1]*

[1]Department of Chemistry, University of Cambridge, Lensfield Road, Cambridge CB2 1EW, UK.

[2]Department of Earth Sciences, University of Cambridge, Downing Street, Cambridge, CB2 3EQ, UK.

[3]School of Chemical Engineering and Analytical Science, The University of Manchester, Oxford Road, Manchester, UK, M13 9PL, UK.Index

1 Nomenclature and abbreviations used 9
2 Computer Simulations for Form A and Form B (system ii) 11
2.1 Retrieval of crystal structures 11
2.2 Calculation of lattice energies (Parameters and convergence tests) 11
2.3 Calculation of lattice energies (Reported energies) 12
2.4 Identifying important faces of the crystal morphologies 12
2.5 Generation of Slices 13
2.6 Testing Vacuum width for energy convergence 14
2.7 Calculation of Slice, Attachment and Face Energies in Vaccum and Water 14
2.8 Results of Slice, Attachment and Face Energies in various Solvents 15
2.9 Calculation of Crystallite Energies as a Function of size 17
3 Experimental Section: Materials and Equipment 19
3.1 Materials 19
3.2 Equipment 19
3.2.1 Ball mill grinder 19
3.2.2 Automated solenoid to press the Retsch MM400 Shaker Mill start button 20
3.3 Grinding jars 20
3.3.1 Snap closure grinding jars 20
3.3.2 Screw closure grinding jars with Teflon washer 21
3.3.3 Large scale snap closure grinding jars 21
3.4 Balance 21
3.5 Automatic pipettes 21
3.6 Hamilton syringes for dispensing DCM 22
3.7 HPLC equipment & columns 22
3.8 PXRD equipment 22
4 Experimental Section: methodology 23
4.1 General concepts 23
4.1.1 Dynamic covalent Chemistry 23

4.1.2 Solid state dynamic covalent chemistry 23
4.2 Preparation of polymorphs of the disulfide heterodimer from System ii: Form A and Form B 24
4.2.1 Preparation of Form A and Form B by solid-state DCC (200 mg scale) 24
4.2.2 Preparation of large scale amount of Form B by solid-state DCC (1.6g scale) 24
4.2.3 Preparation of crystals of Form B 24
4.2.4 Preparation of crystals/powder of Form A 24
4.3 Experimental procedures: kinetic studies and turnover experiments of system ii by polymorph interconversion between Form A and Form B in solid state DCC by ball mill grinding (contains dbu) 25
4.3.1 Procedure: Kinetic studies of polymorph transformation of Form B to Form A in solid state DCC under ball mill Neat grinding conditions (contains dbu) 25
4.3.2 Procedure: Kinetic studies of polymorph transformation of Form A to Form B in solid state DCC under ball mill LAG conditions with 50μL MeCN (contains dbu) 25
4.3.3 Procedure: Turnover experiments of polymorph interconversion in System ii between Form A & Form B in solid state DCC by ball mill grinding (contains dbu) 26
4.4 Experimental procedures: kinetic studies and turnover experiments of system ii by direct polymorph interconversion between Form B and Form A (absence of catalyst) 27
4.4.1 Procedure: Kinetic studies: direct polymorph transformation of Form B to Form A under ball mill neat grinding conditions (absence of catalyst) 27
4.4.2 Procedure: Kinetic studies: direct polymorph transformation of Form A to Form B under ball mill LAG conditions with 50μL MeCN (absence of catalyst) 27
4.4.3 Procedure: Turnover experiments: direct polymorph interconversion between Form A and Form B by ball mill grinding (absence of catalyst) 27
4.5 Procedures for ball mill LAG of System (ii) under solid state DCC conditions using different LAG solvents (presence of dbu) 28
4.5.1 PROCEDURE 1: Ball mill grinding 1:1 $(2NO_2PhS)_2$: $(4ClPhS)_2$+2%M dbu to equilibrium with MeCN, Acetone, THF, DMF, EtOAc, and $CHCl_3$ as LAG solvents. 29
4.5.2 PROCEDURE 2: Ball mill grinding 1:1 $(2NO_2PhS)_2$: $(4ClPhS)_2$+2%M dbu to equilibrium with DCM as LAG solvents. 30
4.5.3 PROCEDURE 3: Ball mill grinding 1:1 $(2NO_2PhS)_2$: $(4ClPhS)_2$+2%M dbu to equilibrium with poorly wetting LAG solvents (MeOH). 31
4.5.4 PROCEDURE 4: Ball mill grinding 1:1 $(2NO_2PhS)_2$: $(4ClPhS)_2$+2%M dbu to equilibrium with all poorly wetting LAG solvents (excluding water). 32
4.5.5 PROCEDURE 5: Ball mill grinding 1:1 $(2NO_2PhS)_2$: $(4ClPhS)_2$+2%M dbu to equilibrium with water as LAG solvent 33
4.6 Procedures for preparation of Theophylline: Benzamide cocrystals 34
4.6.1 Preparation of Form I of Theophylline: Benzamide cocrystal 34
4.6.2 Preparation of Form II of Theophylline: Benzamide cocrystal 34
4.6.3 Investigation of the thermodynamic product of the Theophylline: Benzamide cocrystals under Neat grinding or LAG with apolar solvents conditions 35
4.6.4 Procedure: Kinetic studies of polymorph interconversion from Form I to Form II by ball mill LAG with polar solvent at 1g payload 37

4.6.5 Procedure: Kinetic studies of polymorph interconversion from Form II to Form I by ball mill neat grinding at 1g payload 37
4.6.6 Procedure: Turnover experiments of polymorph interconversion between Form I and Form II of the 1:1 tp:bzm cocrystals at 1g payload 37
4.7 Procedure: Solubility studies in 15 solvents of $(2NO_2PhS)_2$, $(4ClPhS)_2$ and the 2 polymorphs of heterodimer in System ii: Form A and Form B 38
4.7.1 Design of the protocol for solubility studies 38
4.7.2 Methodology for solubility determination by HPLC 39
4.7.3 Procedure for the solubility determination of Form A and Form B 40
5 Analysis of the solid state samples 43
5.1 Analysis by HPLC 43
5.1.1 Preparation of HPLC samples from solid state DCC studies 43
5.2 Analysis by PXRD 44
5.2.1 Sample preparation for PXRD analysis 44
5.3 Rietveld quantitative analysis of solid state samples 45
5.4 Quantification of amorphous phase using Rietveld refinement 47
5.5 Crystal size determination from powder diffraction data 49
6 Polymorph interconversion Form B and Form A in solid state dynamic covalent chemistry by ball mill grinding using base catalyst 50
6.1 Kinetic studies for polymorph transformation from Form B to Form A in solid state DCC under ball mill neat grinding conditions (contains dbu). 50
6.2 Kinetic studies for polymorph transformation from Form A to Form B in solid state DCC under ball mill LAG conditions (contains dbu). 53
6.3 Turnover experiments for polymorph interconversion between Form A and Form B in solid state DCC by ball mill grinding. 57
6.3.1 Example 1 (5 turnover experiments performed) 57
6.3.2 Example 2 (3 turnover experiments performed) 59
7 Direct Polymorph interconversion between Form B and Form A in the absence of base catalyst 61
7.1 Kinetic studies for direct polymorph transformation from Form B to Form A under ball mill neat grinding conditions (absence of catalyst). 61
7.2 Kinetic studies for the direct polymorph transformation from Form A to Form B under ball mill LAG conditions with 50μL MeCN (absence of catalyst). 64
7.3 Turnover experiments for the direct polymorph interconversion between Form A and Form B in the absence of base catalysts 67
8 Formation of disulfide polymorphs (Form A and Form B) in System i-i by ball mill LAG using 16 LAG solvents 69
8.1 Tabulation of the locations for each solvent system 69
8.2 Solid-state DCC reaction by ball mill LAG using MeCN as LAG solvent (Procedure 1) 70
8.2.1 Accuracy and precision of dispensing MeCN (Procedure 1) 70
8.2.1 Experimental details and HPLC data (Procedure 1) 71

8.2.2 PXRD scans, tabulation of Rietveld refinement of PXRD data and corresponding experimental milling equilibrium curves (Procedure 1) 72
8.3 LAG experiments using Acetone as grinding liquid on solid-state DCC reaction. 75
8.3.1 Accuracy and precision of dispensing acetone (Procedure 1) 75
8.3.2 Experimental details and HPLC data (Procedure 1) 76
8.3.3 PXRD scans, tabulation of Rietveld refinement of PXRD data and corresponding experimental milling equilibrium curves (Procedure 1) 77
8.4 LAG experiments using tetrahydrofuran as grinding liquid on solid-state DCC reaction. 80
8.4.1 Accuracy and precision of dispensing THF (Procedure 1) 80
8.4.2 Experimental details and HPLC data (Procedure 1) 81
8.4.3 PXRD scans, tabulation of Rietveld refinement of PXRD data and corresponding experimental milling equilibrium curves (Procedure 1) 82
8.5 LAG experiments using dimethylformamide as grinding liquid on solid-state DCC reaction. 85
8.5.1 Accuracy and precision of dispensing DMF (Procedure 1) 85
8.5.2 Experimental details and HPLC data (Procedure 1) 86
8.5.3 PXRD scans, tabulation of Rietveld refinement of PXRD data and corresponding experimental milling equilibrium curves (Procedure 1) 87
8.6 LAG experiments using ethyl acetate as grinding liquid on solid-state DCC reaction. 90
8.6.1 Accuracy and precision of dispensing ethyl acetate (Procedure 1) 90
8.6.2 Experimental details and HPLC data (Procedure 1) 91
8.6.3 PXRD scans, tabulation of Rietveld refinement of PXRD data and corresponding experimental milling equilibrium curves (Procedure 1) 92
8.7 LAG experiments using Chloroform as grinding liquid on solid-state DCC reaction. 95
8.7.1 Accuracy and precision of dispensing chloroform 95
8.7.2 Experimental details and HPLC data (Procedure 1) 96
8.7.3 PXRD scans, tabulation of Rietveld refinement of PXRD data and corresponding experimental milling equilibrium curves (Procedure 1) 97
8.8 LAG experiments using dichloromethane as grinding liquid on solid-state DCC reaction. (Procedure 2) 100
8.8.1 Accuracy and precision of dispensing DCM 100
8.8.2 Experimental details and HPLC data (Procedure 2) 101
8.8.3 PXRD scans, tabulation of Rietveld refinement of PXRD data and corresponding experimental milling equilibrium curves (Procedure 2) 102
8.9 LAG experiments using dimethyl sulfoxide as grinding liquid on solid-state DCC reaction (Procedure 4) 105
8.9.1 Accuracy and precision of dispensing DMSO (Procedure 4) 105
8.9.2 Experimental details and HPLC data (Procedure 4) 106
8.9.3 PXRD scans, tabulation of Rietveld refinement of PXRD data and corresponding experimental milling equilibrium curves (Procedure 4) 107
8.10 LAG experiments using methanol as grinding liquid on solid-state DCC reaction (Procedure 1) 110

8.10.1 Experimental details and HPLC data (Procedure 1) 110
8.10.2 PXRD scans, tabulation of Rietveld refinement of PXRD data and corresponding experimental milling equilibrium curves (Procedure 1) 111
8.11 LAG experiments using methanol as grinding liquid on solid-state DCC reaction (Procedure 3) 114
8.11.1 Accuracy and precision of dispensing volume of MeOH (normal pipetting) 114
8.11.2 Experimental details and HPLC data (Procedure 3) 115
8.11.3 PXRD scans, tabulation of Rietveld refinement of PXRD data and corresponding experimental milling equilibrium curves (Procedure 3) 116
8.12 LAG experiments using ethanol as grinding liquid on solid-state DCC reaction (Procedure 4) 119
8.12.1 Accuracy and precision of dispensing volume of EtOH (Procedure 4) 119
8.12.2 Experimental details and HPLC data (Procedure 4) 120
8.12.3 PXRD scans, tabulation of Rietveld refinement of PXRD data and corresponding experimental milling equilibrium curves (Procedure 4) 121
8.13 LAG experiments using isopropanol as grinding liquid on solid-state DCC reaction (Procedure 1) 124
8.13.1 Experimental details and HPLC data (Procedure 1) 124
8.13.2 PXRD scans, tabulation of Rietveld refinement of PXRD data and corresponding experimental milling equilibrium curves (Procedure 1) 125
8.14 LAG experiments using isopropanol as grinding liquid on solid-state DCC reaction (Procedure 4) 128
8.14.1 Accuracy and precision of dispensing volume of IPA (Procedure 4) 128
8.14.2 Experimental details and HPLC data (Procedure 4) 129
8.14.3 PXRD scans, tabulation of Rietveld refinement of PXRD data and corresponding experimental milling equilibrium curves (Procedure 4) 130
8.15 LAG experiments using water as grinding liquid on solid-state DCC reaction (Procedure 5) 133
8.15.1 Experimental details and HPLC data (Procedure 5) 133
8.15.2 PXRD scans, tabulation of Rietveld refinement of PXRD data and corresponding experimental milling equilibrium curves (Procedure 5) 134
8.16 LAG experiments using cyclohexane as grinding liquid on solid-state DCC reaction (Procedure 4) 137
8.16.1 Experimental details and HPLC data (Procedure 4) 137
8.16.2 PXRD scans, tabulation of Rietveld refinement of PXRD data and corresponding experimental milling equilibrium curves (Procedure 4) 138
8.17 LAG experiments using benzene as grinding liquid on solid-state DCC reaction (Procedure 4) 141
8.17.1 Experimental details and HPLC data (Procedure 4) 141
8.17.2 PXRD scans, tabulation of Rietveld refinement of PXRD data and corresponding experimental milling equilibrium curves (Procedure 4) 142
8.18 LAG experiments using toluene as grinding liquid on solid-state DCC reaction (Procedure 4) 145

8.18.1 Experimental details and HPLC data (Procedure 4) 145
8.18.2 PXRD scans, tabulation of Rietveld refinement of PXRD data and corresponding experimental milling equilibrium curves (Procedure 4) 146
8.19 LAG experiments using perfluorodecalin as grinding liquid on solid-state DCC reaction (Procedure 4) 149
8.19.1 Experimental details and HPLC data (Procedure 4) 149
8.19.2 PXRD scans, tabulation of Rietveld refinement of PXRD data and corresponding experimental milling equilibrium curves (Procedure 4) 150
8.20 Summary of results of polymorph compositions (%R) at thermodynamic equilibrium versus type and volume of solvent used in ball mill LAG....152
9 Preliminary investigation of phase equilibria and chemical equilibria on ball mill LAG of system ii....156
9.1 Chemical and phase equilibria of ball mill LAG with 25 μL Acetone of system ii at different grinding times....157
9.2 Chemical and phase equilibria of ball mill LAG with 30μL EtOAc of system ii at different grinding times....159
9.3 Chemical and phase equilibria of ball mill LAG with 25μL Chloroform of system ii at different grinding times....161
9.4 Chemical and phase equilibria of ball mill LAG with 60μL DCM of system ii at different grinding times....163
10 Determination of the solubility of Form A and Form B in System ii in 15 solvents 165
10.1 HPLC Method used for the quantitative determination of saturated solutions of Form A &Form B 165
10.1.1 Linearity of HPLC method for Form B; applicable to Form A....165
10.2 Solubility of Form B in water, MeOH, EtOH, IPA and cyclohexane 167
10.3 Solubility of Form B in MeCN, DMSO, Acetone, EtOAc and Toluene....168
10.4 Solubility of Form B in Benzene, DCM, DMF, $CHCl_3$ and THF 169
10.5 Summary of solubility data: Form B in 15 solvents 170
10.6 Solubility of Form A in MeCN, MeOH, EtOH, IPA and cyclohexane....171
10.7 Solubility of Form A in Water, DMSO, Acetone, EtOAc and Toluene 172
10.8 Solubility of Form A in Benzene, DCM, DMF, $CHCl_3$ and THF 173
10.9 Summary of solubility data: Form A in 15 solvents 174
10.10 Summary solubility data for Form A & Form B....175
10.11 Summary solubility data for Form A & Form B in comparison with 1-1 & 2-2 176
10.12 Summary solubility data for individual graphs for Form A & Form B in comparison with 1-1 & 2-2 expressed as mg/mL 177
10.13 Summary solubility data for individual graphs for Form A & Form B in comparison with 1-1 & 2-2 expressed as mmol/mol solvent 178
11 Investigating which of the 2 polymorphs of system ii (Form A and Form B) is the thermodynamic product under ambient conditions (slurry experiment)....179
12 Determination of polymorph stability of Form A and Form B under slurry conditions performed in 15 solvents....180
12.1 Determination of polymorph stability of Form B under slurry conditions....180

12.1.1 Slurry experiment: Form B in MeCN 180
12.1.2 Slurry experiment of Form B in Acetone 180
12.1.3 Slurry experiment of Form B in Tetrahydrofuran 181
12.1.4 Slurry experiment of Form B in Ethyl Acetate 181
12.1.5 Slurry experiment of Form B in Chloroform 182
12.1.6 Slurry experiment of Form B in Dichloromethane 182
12.1.7 Slurry experiment of Form B in Dimethylformamide 183
12.1.8 Slurry experiment of Form B in Dimethylsulphoxide 183
12.1.9 Slurry experiment of Form B in Cyclohexane 184
12.1.10 Slurry experiment of Form B in Benzene 184
12.1.11 Slurry experiment of Form B in Toluene 185
12.1.12 Slurry experiment of Form B in Methanol 185
12.1.13 Slurry experiment of Form B in Ethanol 186
12.1.14 Slurry experiment of Form B in Isopropanol 186
12.1.15 Slurry experiment of Form B in Water 187
12.1.16 Discussion and conclusions on slurry experiments of Form B in 15 solvents 187
12.2 Determination of polymorph stability of Form A under slurry conditions 188
12.2.1 Slurry experiment Form A in MeCN 188
12.2.2 Slurry experiment of Form A in Acetone 188
12.2.3 Slurry experiment of Form A in Tetrahydrofuran 189
12.2.4 Slurry experiment of Form A in Ethyl Acetate 189
12.2.5 Slurry experiment of Form A in Chloroform 190
12.2.6 Slurry experiment of Form A in Dichloromethane 190
12.2.7 Slurry experiment of Form A in Dimethylformamide 191
12.2.8 Slurry experiment of Form A in Dimethylsulphoxide 191
12.2.9 Slurry experiment of Form A in Cyclohexane 192
12.2.10 Slurry experiment of Form A in Benzene 192
12.2.11 Slurry experiment of Form A in Toluene 193
12.2.12 Slurry experiment of Form A in Methanol 193
12.2.13 Slurry experiment of Form A in Ethanol 194
12.2.14 Slurry experiment of Form A in Isopropanol 194
12.2.15 Slurry experiment of Form A in Water 195
12.2.16 Discussion and conclusions on slurry experiments of Form A in 15 solvents 195
12.3 Overall conclusions of slurry experiments 195
12.3.1 Summary of solubility determination and slurry experiments of 1-1, 2-2, Form A and Form B in 15 solvents expressed as mg/mL 196
12.3.2 Summary of solubility determination and slurry experiments of 1-1, 2-2, Form A and Form B in 15 solvents expressed as mmol/mol solvent 197

13 Determination of the particle size of Form A and From B from PXRD scans by Scherrer calculations 198
13.1 Determination of particle size of Form A and From B from turnover experiments of polymorph interconversion of solid state DCC (contains dbu) 198
13.2 Determination of particle size of Form A and From B from turnover experiments of direct polymorph interconversion (absence of dbu) 199
13.3 Determination of particle size of Form A and From B from LAG experiments with different LAG solvents in solid state DCC (presence of dbu) 200
13.3.1 Particle size determination for Form A & Form B with Acetone as LAG solvent 200
13.3.2 Particle size determination for Form A & Form B with THF as LAG solvent 201
13.3.3 Particle size determination for Form A & Form B with DMF as LAG solvent 202
13.3.4 Particle size determination for Form A & Form B with EtOAc as LAG solvent 203
13.3.5 Particle size determination for Form A & Form B with $CHCl_3$ as LAG solvent 204
13.3.6 Particle size determination for Form A & Form B with DCM as LAG solvent 205
13.3.7 Particle size determination for Form A & Form B with DMSO as LAG solvent 206
13.3.8 Particle size determination for Form A & Form B with MeOH as LAG solvent 207
13.3.9 Particle size determination for Form A & Form B with EtOH as LAG solvent 208
13.3.10 Particle size determination for Form A & Form B with IPA as LAG solvent 209
13.3.11 Particle size determination for Form A & Form B with Water as LAG solvent 210
13.3.12 Particle size determination for Form A & Form B with cyclohexane as LAG solvent 211
13.3.13 Particle size determination for Form A & Form B with Benzene as LAG solvent 212
13.3.14 Particle size determination for Form A & Form B with Toluene as LAG solvent 213
13.4 Conclusion of particle size determination by Scherrer equation of PXRD scans 214
14 Thermodynamic experiments with 1:1 Theophilline: Benzamide cocrystals 215
14.1 Introduction 215
14.2 Cocrystals of 1:1 Theophilline: Benzamide: preparation of Form I 216
14.3 Cocrystals of 1:1 Theophilline: Benzamide. Preparation of Form II 218
14.4 Determining which of the cocrystals of 1:1 tp:bzm, Form I or Form II, is the thermodynamic product under ball mill neat grinding conditions 220
14.5 Determining which of the cocrystals of 1:1 tp:bzm, Form I or Form II, is the thermodynamic product under ball mill LAG conditions with polar solvents 222
14.6 Polymorph transformation of Form I to Form II under ball mill LAG conditions 224
14.7 Polymorph transformation of Form II to Form I under ball mill neat grinding conditions 226
14.8 Turnover experiments: polymorph interconversion between Form I and Form II cocrystals of 1:1 tp:bzm by ball mill grinding 228
14.9 Thermodynamic study: ball mill LAG conditions taken to equilibrium to 1g of Form I with increasing volume of water as LAG solvent. 231
14.10 Determination if Form I or Form II of 1:1 tp:bzm cocrystals is the thermodynamic product under slurry conditions 234
14.10.1 Conclusion of this experiment: 234
15 Scanning Electron Microscopy (SEM) 235

16 References 236

1 Nomenclature and abbreviations used

LAG	Refers to ball mill liquid assisted grinding. The term "LAG" is equivalent and assumes we are discussing ball mill LAG
NG	Refers to ball mill neat grinding. The term "NG" is equivalent and assumes we are discussing ball mill neat grinding
System ii	Refers to the reaction and reaction outcome ($2NO_2PhSSPh4Cl$) from: $(2NO_2PhS)_2$ + $(4ClPhS)_2$<==> $2NO_2PhSSPh4Cl$
1-1	Refers to the homodimer used in system ii: $(2NO_2PhS)_2$ or bis(2-nitrophenyl) disulfide
2-2	Refers to the homodimer used in system ii: $(4ClPhS)_2$ or bis(4-chlorophenyl) disulfide
1-2 (also referred as System ii)	Refers to chemical $2NO_2PhSSPh4Cl$ regardless of the polymorphic form<br>1-2 = **Form A** + **Form B**
**Form A**	Refers to the polymorph of System ii ($2NO_2PhSSPh4Cl$) obtained typically from NG: CSD refcode FUQLIM01. Also referred to as (1-2)A.
**Form B**	Refers to the polymorph of System ii ($2NO_2PhSSPh4Cl$) obtained typically from LAG (50μL MeCN): CSD refcode FUQLIM. Also referred to as (1-2)B.
System i	Refers to the cocrystal system 1:1 Theophylline:Benzamide
tp	Theophylline anhydrous
bzm	Benzamide
Form I	Refers to the polymorph of System i (1:1 tp:bzm) obtained typically from NG:<br>CSD refcode RABXIE02.
Form II	Refers to the polymorph of System i (1:1 tp:bzm) obtained typically from NG:<br>CSD refcode RABXIE01.
MeCN	Acetonitrile
THF	Tetrahydrofuran
EtOAc	Ethyl Acetate
$CHCl_3$	Chloroform
DCM	Dichloromethane
DMF	Dimethylformamide
DMSO	Dimethylsulfoxide
cHexane	Cyclohexane
MeOH	Methanol
EtOH	Ethanol
IPA	Isopropanol or 2-Propanol
H_2O	water
F8-decaline	Octadecafluorodecahydronaphthalene

dbu	1,8-Diazabicyclo[5.4.0]undec-7-ene (base catalyst)
TFA	Trifluoroacetic acid
DCC	Dynamic covalent chemistry (see section 4.1.1)
Solid state DCC	Solid state dynamic covalent chemistry (see section 4.1.2)
DCL	dynamic combinatorial library: library resulting from DCC
HPLC	High performance liquid chromatography
PXRD	Powder X-ray diffractometry
GC	Gas Chromatography
ID	Internal diameter
Hz	Hertz (frequency used to swing the grinding jars by the ball mill grinder)
ss	Stainless steel
h	hours
m	Minutes

2 Computer Simulations for Form A and Form B (system ii)

2.1 Retrieval of crystal structures

Coordinates and unit cell parameters for polymorphs A and B of system (ii) were retrieved from the Cambridge Structural Database[1] version 5.37 using Conquest version 1.18. **Form B** corresponds to the CSD refcode FUQLIM (space group P-1)[2] and **Form A** corresponds to the CSD refcode FUQLIM01 (space group P21/n).[3] The space group settings of FUQLIM01 was changed from $P2_1/n$ to $P2_1/c$. Both structures were exported as cif files. A view of the unit cells of both polymorphs is given in Figure S1. We note that **Form A** has four molecules in the unit cell (Z=4) and **Form B** has two molecules in the unit cell (Z=2).

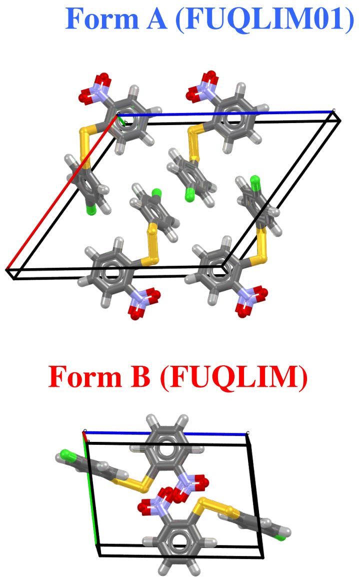

Figure S 1.Unit cell representations of forms A (upper) and B (lower).

2.2 Calculation of lattice energies (Parameters and convergence tests)

Density Functional Theory with van der Waals corrections (DFT-d) was used for crystal geometry relaxations of forms A and B. Structural relaxations were performed allowing all possible structural parameters to relax (unit cell parameters as well as atomic positions). The PBE functional[4] was used with PAW pseudopotentials[5,6] and the Grimme's van der Waals corrections (d2)[7] as implemented in the VASP code. [8–11] The VASP version 5.3.3 was first used in the initial geometry relaxations and in the convergence tests of the simulation cells. The Brillouin zone was sampled using the Monkhorst-Pack approximation[12] and a variety of k-point grids (see below). Structural relaxations were halted when the calculated force on every atom was less than 0.003 eV/Å.

Lattice energies were calculated by subtracting the electronic energy of a single molecule in the gas-phase from the electronic energy of the simulation cell in the crystal divided by the number of molecules (N). The electronic energy of a single molecule in the gas-phase was computed with the same models by placing a single molecule of (ii) on the center of a $(30Å)^3$ supercell. For this calculation, supercell parameters were kept fixed and all atomic coordinates of the molecule were allowed to relax. Lattice energies were then calculated as in equation (1).

$$E_{latt}\,[\text{Form A}] = E_e[\text{Form A}]/N - E_e[\text{single molecule gas-phase}] \qquad \textbf{(1)}$$

Relative energies of forms A and B (ΔE_{latt}) were then calculated by substracting the respective lattice energies as given in equation (2).

$$\Delta E_{latt} = E_{latt}[\text{Form A}] - E_{latt}[\text{form B}] \qquad \textbf{(2)}$$

Convergence of the energies were tested by varying the energy cut-off of the plane wave as well as the number of points in the k-point sampling. This was tested for four different energy cut-off values (220, 320, 420 and 520 eV) and three different sets of k-points for forms A/B (1x2x1/2x2x1, 2x3x2/3x3x2 and 3x4x3). Convergence was achieved for cut-off energy values of 520 eV and k-point sampling of 2x3x2 for **Form A** and 3x3x2 for **Form B**. These parameters were used for the rest of the calculations in this article.

2.3 Calculation of lattice energies (Reported energies)

After the convergence tests, the structures of forms A and B (as well as the gas-phase single molecule) were reoptimised a second time using a VASP version 5.4.1 installed with the VASPsol module.[13,14] The parameters described in the section above were used with the optimal cut-off energies and k-points. A comparison of the experimental and optimised unit cell parameters, as well as energetics and the $rmsd_{20}$ parameter are given in table S1. The $rmsd_{20}$ parameter is calculated using COMPACK as implemented in mercury. For this, a 20-molecule cluster is overlaid for the reference (experimental) and the comparison (simulated) structures and the root mean square deviation of the atomic positions is calculated ($rmsd_{20}$). This parameter is standardly used for comparing experimental with simulated crystal structures. As we can see in table S1, there is an excellent agreement between the experimental and the simulated structures. We see a slight contraction of the cell upon the simulations; this is because our calculations are done at 0 K whilst the structure determination is usually done between 100-300 K. Our DFT-d model predicts form B to be the most able form by only 1.3 kJ/mol.

Table S 1. Experimental and optimised unit-cell parameters for forms A and B.

	**Form A (FUQLIM01)**		**Form B (FUQLIM), P-1**	
Space Group	P21/c		P-1	
Z	4		2	
Z'	1		1	
Structure	Experimental	Simulated	Experimental	Simulated
Cell Volum ($Å^3$)	1244.7	1189.7	610.7	591.9
a (Å)	13.835	13.624	7.043	6.847
b (Å)	7.103	7.055	7.832	7.812
c (Å)	15.709	15.324	11.371	11.346
Alpha	90.0	90.0	82.6	81.7
Beta	126.3	126.1	80.7	80.8
Gamma	30.0	90.0	83.2	85.5
T (k)	180	0	150	0
R-factor (%)	3.48	-	4.4	-
ΔE_{latt} (kJ/mol)	-	1.3	-	0.0
Elatt (kJ/mol)	-	-112.8	-	-114.1
$rmsd_{20}$(Å)	0.258		0.237	

2.4 Identifying important faces of the crystal morphologies

The first step for the simulation of surface effects in our polymorphs is the identification of crystal surfaces that would dominate in the crystal morphologies. Planes in crystals are identified by their miller indexes (hkl) and planes that are related by the symmetry of the lattice can be grouped into a set of planes denoted as {hkℓ}. For example, the plane (1 0 0) in **Form B** is related by inversion to the plane (-1 0 0). Since these two planes are symmetrically equivalent, the surfaces generated by cutting the lattice along that plane have the same structure (related by inversion), the same energy and they contribute equally to the particle morphology. There are infinite planes that could be generated from a crystal lattice and infinite surfaces that could be cut along those planes. Those with lower miller indexes, however, are usually the most important surfaces present in crystal morphologies.

Since the calculation of surface energies with a DFT-d methods is very computationally demanding, we proceeded to identify key surfaces for polymorphs A and B using two simple and computationally inexpensive models first. First, we used the Bravais, Friedel, Donnay and Harker (BFDH) crystal morphology model, which relates d-spacings to crystal morphology, as implemented in Mercury through the CSD Materials module.[15] Second, we calculated attachment energies and growth morphologies (GM)[16] for both forms A and B. For this, the Dreiding forcefield[17] was employed making use of the Gasteiger charges[18] with tight convergence criteria as implemented in Material studio(MS).[19] The percentage of the surface morphology for each particular family of faces {hkℓ} is given in table S2 for forms A and B and the two morphology prediction models.

Table S 2. Summary of set of planes and their importance in the BFDH and AE morphologies (expressed as the % of the total morphology) for forms A and B.

**Form A** ($P2_1/c$)					**Form B** (P-1)				
**h k l**	**d_{hkl}**	**# Planes**	**BFDH**[¶]	**GM-MS**[§]	**h k l**	**d_{hkl}**	**# Planes**	**BFDH**[¶]	**GM-MS**[§]
{1 0 0}	11.005	2	40 %	35 %	{0 0 1}	11.150	2	39 %	29 %
{0 1 1}	6.129	4	14 %	25 %	{1 0 0}	6.313	2	20 %	28 %
{1 0-2}	7.657	2	24 %	22 %	{0 1 0}	7.727	2	25 %	28 %
{0 0 2}	6.189	2	0 %	8 %	{1 0 1}	6.313	2	7 %	9 %
{1 1-1}	6.175	4	17 %	7 %	{1 1 1}	5.307	2	2 %	3 %
{1 1 0}	5.939	4	3 %	3 %	{0 1-1}	6.916	2	0 %	3 %

¶ Calculated using the CSD Materials module as implemented in Mercury.
§Calculated with Materials Studio using the Dreiding forcefield with Gasteiger charges. Over 2000 faces were generated for the AE calculations.

2.5 Generation of Slices

Slices of the important surfaces identified with the BFDH as well as the attachment energy models were generated from the DFT-d optimised crystal structures using knowledge of crystallography. An example of such generation is given in Figure S2. From the optimised unit cell (S2a), a supercell is generated by expanding the necessary unit cell direction. For example, for the (002) face in **Form A**, this corresponds to the c axis. The new c axis, c' is equal to the old c axis plus 15Å to ensure that the vacuum in the new super cell is large enough. If necessary, molecules are removed to ensure that the computation concerns to a unique layer of molecules (S2b).

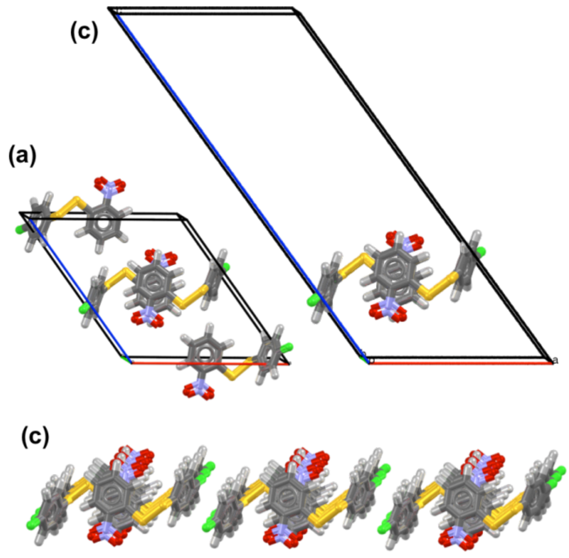

Figure S 2 Example of slice generation for the (002) surface of **Form A**.

Expansion of the supercell in the a and b direction gives rise to the entire slice (S2c). A visual representation of all the slices generated for this study is given in Figure S3. We note that, in certain cases, the choice of origin for the slice generation is important and could result in two different slices. For those cases, both configurations were generated and optimised. For example, for form B, slice (001) can be generated in in two configurations depending on the choice of origin (A and B). We considered both.

Five slices were generated for **Form A** and seven slices were generated for **Form B**. The packing efficiency, surface roughness and chemistry of the slices can be very different as it can be seen from the visual images in S3.

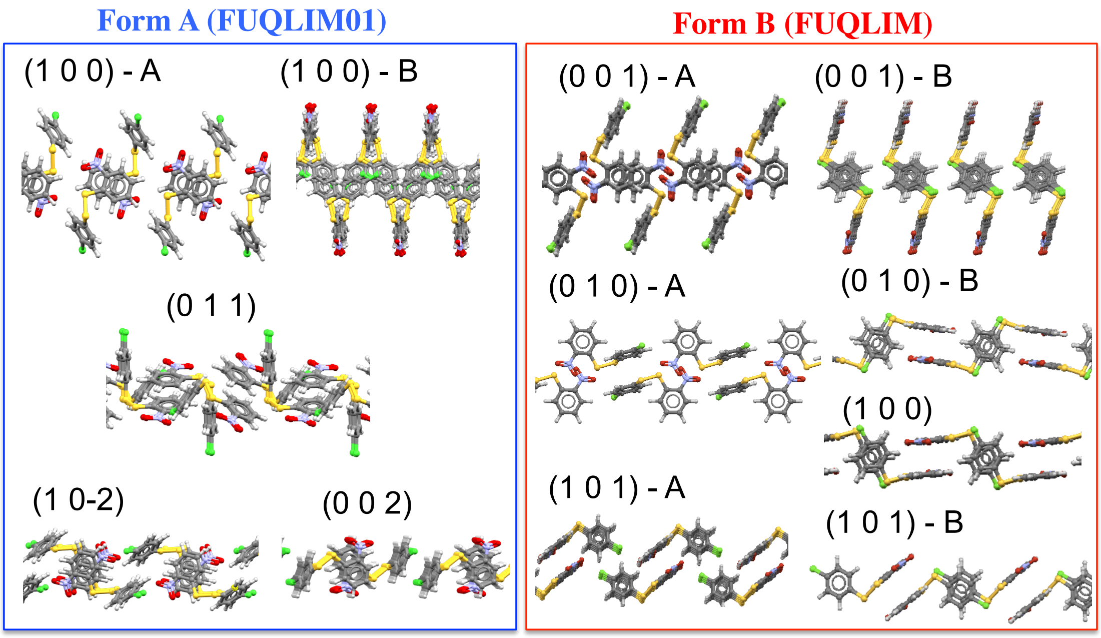

Figure S 3 Side view of the twelve slices constructed for forms A and B.

2.6 Testing Vacuum width for energy convergence

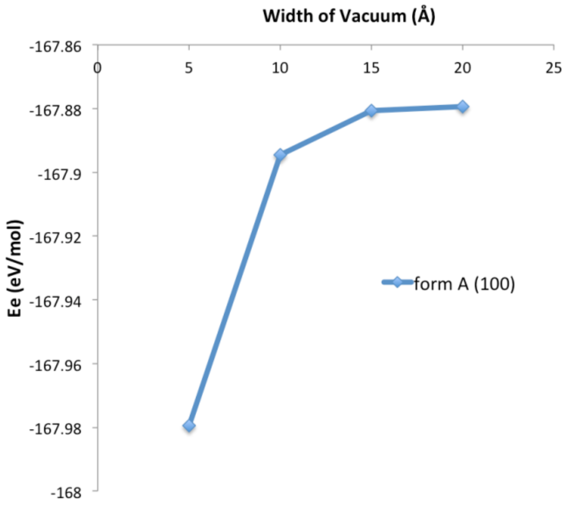

Figure S 4 Electronic energy of system (ii) in a **Form A** (100) slice as a function of the width of the vacuum in the simulation cell.

When running calculations of slice energies, it is very important that the width of the vacuum in the simulation cell is large enough so that slices in consecutive simulation cells do not feel each other. We performed several simulations in order to arrive to the optimal vacuum width for our system (Figure S3). We found that the energy of the molecules on surface (100) of **Form A** converged when the vacuum had a width of 15Å. As a consequence, for the simulations of all the slices presented in this work have been done with a vacuum width of at least 15Å.

2.7 Calculation of Slice, Attachment and Face Energies in Vaccum and Water

Electronic energies of the generated slices were calculated using VASP 5.4.1 and VASPsol. The parameters used for the calculations are all identical to those specified for the lattice energy calculations except when specified. K-points were adjusted to the new simulation cells. They were kept the same for those cell lengths that remained unchaged and only K-point was used in the direction perpendicular to the slice since, in that direction, the supercell length would take large values between 30-50 Å.

First a single point energy calculation was performed for the generated slices using VASP 5.4.1. Wavefunctions from this calculation were then saved and taken as initial guesses for the calculations with VASPsol.[13,14] With VASPsol,[13,14] five different environments were simulated: i) ε = 1 (vacuum), ii) ε = 20 (acetone), iii) ε = 40 (acetonitrile), iv) ε = 60 and ii) ε = 80 (water). VASPsol is a module which allows the calculation of solvation effects through an implicit model of solvation. For these calculations, the dielectric constants of the implicit solvents were specified.

Electronic supercell energies (in both the vacuum and the water environment) were then converted to slice, attachment and surface energies with equations 3, 4 and 5 and the energies were converted to kJ/mol.

$$E_{slice}[formX/faceY] = (E_e[formX/faceY])/N - E_e[single\ molecule\ gas\text{-}phase] \quad (3)$$

$$E_{att}[formX/faceY] = E_{latt}[formX] - E_{slice}[formX/faceY] \quad (4)$$

$$E_{face}[formX/faceY] = E_{latt}[formX] + (E_{att}[formX/faceY])/2 \quad (5)$$

The slice energy (E_{slice}) is the energy of a molecule within the simulation slice and is given in kJ/mol. The attachment energy (E_{att}) is defined as the difference between the lattice and the slice energy and is also given in kJ/mol. Attachment energies are widely used for the prediction of crystal growth morphologies.

Finally, we refer to face energy (E_{face}) to the potential energy felt by a molecule in that particular face of the crystal in kJ/mol. This is approximated as the sum between the lattice energy and half of the attachment energy. In order to validate this approximation, we computed the energy of molecules on six different surfaces of **Form B** and plotted the E_{face} as computed from first principles and E_{face} as estimated from lattice and attachment energies. There is an excellent correlation between these two values. Because of the expense of the computations (much larger supercells are needed to compute E_{face} form first principles), however, we used the estimated values.

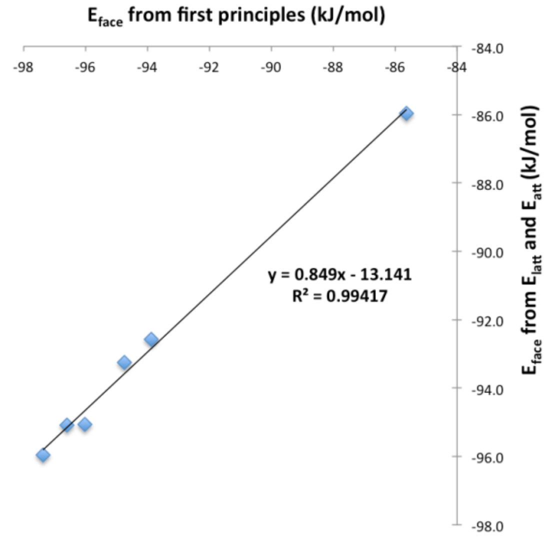

Figure S 5 Correlation between Eface values as computed from first principles (x) and as estimated from lattice and attachment energies.

2.8 Results of Slice, Attachment and Face Energies in various Solvents

Slice, attachment and face energies were computed for forms A and B in five different solvent environments (Table S3). Attachment energies are greatly affected by the environment as seen in table S3. Faces can be significantly stabilised in the presence of a solvent and so is illustrated in the computed values.

Using the attachment energy values in different solvents, growth morphologies were derived for both polymorphs A and B. The extreme cases (for vacuum and water) are visualised in Figure S6.

With the growth morphologies, then, the importance of each face to the overall crystal was calculated and is summarised in table S4. For the model used, morphological changes were small in going from vacuum to water, however, the slice, attachment and face energies can change substantially (table S3).

Table S 3 Summary of slice, attachment and face energies for the most important morphological surfaces in forms A and B. Energies are given in kJ/mol.

	**Form A** ($P2_1/c$) – FUQLIM**01** (E_{latt} = -112.8)				**Form B** (P-1) – FUQLIM (E_{latt} = -114.1)			
	{h k l}	E_{slice}	E_{att}	E_{face}	{h k l}	E_{slice}	E_{att}	E_{face}
Vacuum ε= 1	{1 0 0}*	-70.4	-42.3	-91.6	{0 0 1}*	-61.8	-52.2	-88.0
	{0 1 1}	-50.7	-62.1	-81.7	{1 0 0}	-69.1	-45.0	-91.6
	{1 0-2}	-66.8	-46.0	-89.8	{0 1 0}*	-54.0	-60.0	-84.0
	{0 0 2}	-52.3	-60.5	-82.5	{1 0 1}*	-58.6	-55.5	-86.3
Acetone ε= 20	{1 0 0}*	-79.7	-33.1	-96.2	{0 0 1}*	-71.1	-42.9	-92.6
	{0 1 1}	-68.6	-44.1	-90.7	{1 0 0}	-75.5	-38.6	-94.8
	{1 0-2}	-77.7	-35.1	-95.2	{0 1 0}*	-70.3	-43.8	-92.2
	{0 0 2}	-70.9	-41.9	-91.8	{1 0 1}*	-70.6	-43.5	-92.3
Acetonitrile ε= 40	{1 0 0}*	-82.1	-30.7	-97.4	{0 0 1}*	-73.6	-40.5	-93.8
	{0 1 1}	-69.1	-43.6	-90.9	{1 0 0}	-77.4	-36.7	-95.7
	{1 0-2}	-80.2	-32.6	-96.5	{0 1 0}*	-74.1	-39.9	-94.1
	{0 0 2}	-74.5	-38.3	-93.6	{1 0 1}*	-73.3	-40.7	-93.7
ε= 60	{1 0 0}*	-83.6	-29.2	-98.2	{0 0 1}*	-75.1	-39.0	-94.6
	{0 1 1}	-70.5	-42.3	-91.6	{1 0 0}	-78.6	-35.4	-96.3
	{1 0-2}	-81.6	-31.1	-97.2	{0 1 0}*	-76.4	-37.7	-95.2
	{0 0 2}	-76.6	-36.2	-94.7	{1 0 1}*	-75.0	-39.1	-94.5
Water ε= 80	{1 0 0}*	-84.6	-28.2	-98.7	{0 0 1}*	-76.1	-38.0	-95.1
	{0 1 1}	-71.7	-41.0	-92.3	{1 0 0}	-79.5	-34.6	-96.8
	{1 0-2}	-82.7	-30.1	-97.7	{0 1 0}*	-77.8	-36.2	-96.0
	{0 0 2}	-78.1	-34.7	-95.4	{1 0 1}*	-76.1	-38.0	-95.1

*Two different slice configurations were computed for two different origins. The energy of the most stable one is given in each case.

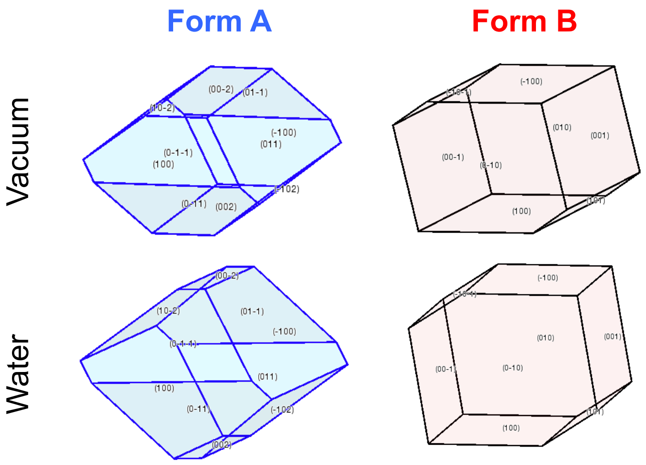

Figure S 6. Growth morphologies for forms A and B in vacuum and water.

Table S 4 Morphological importance (in%) of the different faces according to the more basic BFDH and GM-MS models and the DFT-d derived growth morphologies (GM) in vacuum and water from DFT-d models.

		Form A ($P2_1/c$)				Form B (P-1)			
**Model**	ε	{1 0 0}	{0 1 1}	{1 0-2}	{0 0 2}	{0 0 1}	{1 0 0}	{0 1 0}	{1 0 1}
BFDH	-	40%	14%	24%	0%	39%	20%	25%	7%
**GM-MS**	**-**	35%	25%	22%	8%	29%	28%	28%	9%
Vacuum	1	37%	28%	35%	4%	33%	34%	33%	12%
Acetone	20	39%	25%	33%	3%	28%	27%	31%	14%
Acetonitrile	40	35%	27%	34%	3%	28%	27%	29%	15%
ε= 60	60	38%	25%	35%	3%	28%	27%	29%	15%
Water	80	33%	30%	31%	3%	28%	27%	29%	15%

2.9 Calculation of Crystallite Energies as a Function of size

A python code was written to compute the particle energies as a function of size for forms A and B in the different simulation environment. We assumed that the growth morphologies could be a good estimate of the shape of the ground particle. This will be investigated further in future studies.

To compute the particle energies, first aspect ratios of the forms were derived (table S5) for all the solvent environments considered. For this, we measured the overall shape of the crystal in three dimensions and presented them relative the smallest one. Aspect ratios of the growth morphologies are reported in table S5. The solvent environment did not seem to affect the overall aspect ratio of the crystal. We note that for A has a direction considerably longer than the other two (1.7: 1.1: 1.0), so it should be more needle or prism like. **Form B**, by contrast, is more close to a cubic morphology (1.0:1.0:1.0). Volume and surface factors were then derive. This is, the factors needed to convert the volume/surface as calculated with the aspect ratio to the real volume/surface (V_{real} = VolumeFactor * $V_{aspect\text{-}ratio}$).

Table S 5 Aspect ratios of the derived growth morphologies and scaling factors for crystal volumes and surfaces.

Form	ε	Aspect Ratio Long	Med	Short	Volume Factor	Surface Factor
Form A ($P2_1/c$)	1	1.8	1.1	1.0	0.951	0.678
	20	1.6	1.1	1.0	0.948	0.823
	40	1.8	1.1	1.0	0.971	0.815
	60	1.7	1.0	1.0	0.903	0.818
	80	1.6	1.1	1.0	0.974	0.910
Form B (P-1)	1	1.3	1.1	1.0	0.867	0.796
	20	1.1	1.1	1.0	0.874	0.872
	40	1.1	1.1	1.0	0.888	0.864
	60	1.0	1.1	1.0	0.834	0.830
	80	1.1	1.1	1.0	0.888	0.864

A simulation was run in the python code in which the particle energy was computed as a function of size where size refers to the shortest-axis of the particle. For each given size, the aspect ratio was used to derive the number of molecules which are on the bulk of the particle and on the difference surfaces of the particle. The energy was then computed using equation (6) in which N_{bulk}, N_{hkl} and $N_{particle}$ are the number of molecules in the particle in the bulk, in the hkl face and the total number of molecules and E_{latt} and E_{hkl} are the lattice energy and the surface energy of the hkl face. This process was repeated for each solvent environment. Plots of particle energies as a function of size are given in figure S7.

$$E_{particle} = \frac{N_{bulk}E_{Latt} + \sum N_{hkl}E_{hkl}}{N_{particle}} \quad \textbf{(6)}$$

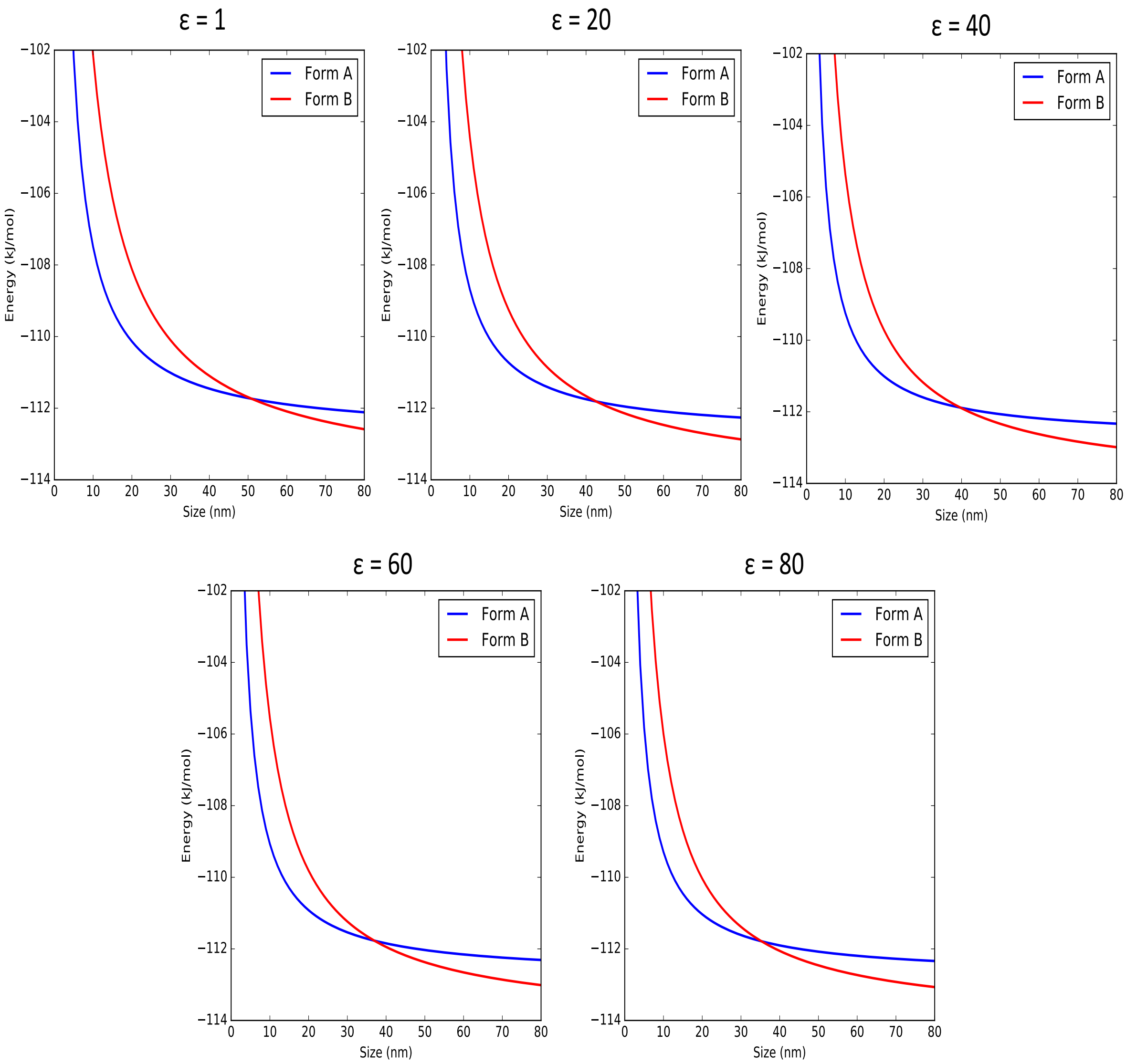

Figure S 7 Particle energy per mol as a function of size in nm for forms A and B particles in five different solvation environments.

3 Experimental Section: Materials and Equipment

3.1 Materials

All HPLC solvents used with for the analysis of the samples from system ii (disulfide) were of high purity HPLC grade: Methanol was acquired from Fisher as HPLC gradient grade. Water was freshly collected from MilliQ Synthesis reverse osmosis unit or acquired from Fisher as HPLC grade. Formic acid was obtained from Fluka, puriss for Mass Spectrometry grade. Acetonitrile for HPLC and HPLC sample preparation was purchased from Fisher as HPLC grade and TFA was purchased from Sigma Aldrich as Reagent-Plus 99%.

All solvent used for ball mill grinding experiments were obtained as follows: acetonitrile (MeCN), acetone, dichloromethane (DCM, unstabilised), methanol (MeOH), isopropanol (IPA), tetrahydrofurane (THF, unstabilised) and toluene were HPLC grade from Fisher Scientific; ethanol was puriss, absolute, >99.8% by GC from Sigma Aldrich; water was HPLC grade from Rathburn; dimethylformamide (DMF) and dimethylsulfoxide (DMSO) were 99+% pure from Alfa Aesar, chloroform ($CHCl_3$, stabilised with amylose) was analytical reagent grade from Fisher; cyclohexane was laboratory reagent grade, ≥99.8% from Fisher; benzene was puriss pa reagent from Sigma Aldrich; ethyl acetate (EtOAc) was freshly distilled in house from LR grade and Octadecafluorodecahydronaphthalene [306-94-5] >95% by GC was obtained from TCI.

All disulfide starting materials for system ii and reagents used in the solids state DCL experiments were purchased from commercial suppliers: 1,8-Diazabicyclo[5.4.0]undec-7-ene (dbu) [6674-22-2] (>97.5 % by GC) was obtained from Acros Organics, bis(4-chlorophenyl) disulfide [1142-19-4] (98+%) referred here as **2-2** was purchased from TCI and bis(2-nitrophenyl) disulfide [1155-00-6] (98%) referred here as **1-1** was purchased from Sigma Aldrich.

For the cocrystals experiments (System i), theophylline anhydrous was obtained from Sigma as >99%, while benzamide was obtained from Aldrich as 99%pure.

3.2 Equipment

3.2.1 Ball mill grinder

The ball mill grinding experiments were all performed using in a Retsch MM400 Shaker Mill with the safety cover removed as the grinder motor vents to the front of the equipment warming the grinding jars on prolonged grinding. An external safety shield was used for safety.

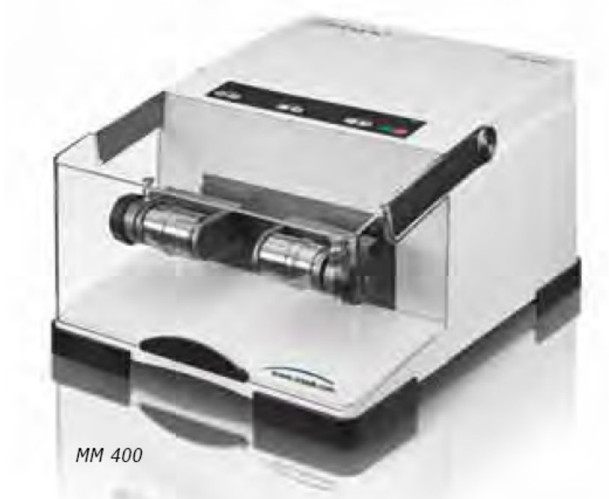

Figure S 8: Retsch MM400 Shaker Mill (ball mill grinder)

3.2.2 Automated solenoid to press the Retsch MM400 Shaker Mill start button

Retsch MM400 Shaker Mill stops automatically once the run time has elapsed. It can only be automatically run for a maximum of 99 minutes. As these studies required in many cases up to multi hours grinding, an automated solenoid was manufactured in house and controlled with in-house software to press repeatedly the START button as often as the experiment required it. To prevent the Retsch MM400 motor from overheating, 5-10 minutes rest was allowed for each hour grinding.

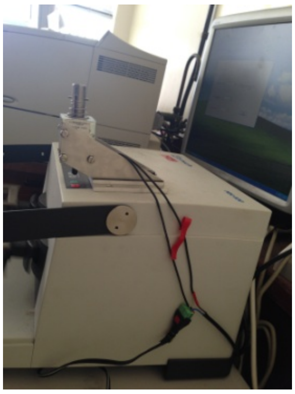

Figure S 9: Solenoid used as push-finger to automatically press the START button on the Retsch MM400

3.3 Grinding jars

3.3.1 Snap closure grinding jars

The snap closure grinding jars were manufactured in-house from hardened stainless steel with a 14.5 mL internal volume (19 mm ID x 54mm internal length). These snap-closure grinding jars were used in previous studies.[3,20] and we have used them here for System i cocrystals studies (See Section 4.6 and Section 14) and for system ii (See Section 4.2.1 and 4.2.4, Section 4.3 and Section 4.4.1 and 4.4.2).

a)

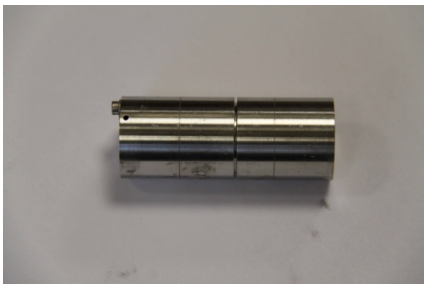

b)

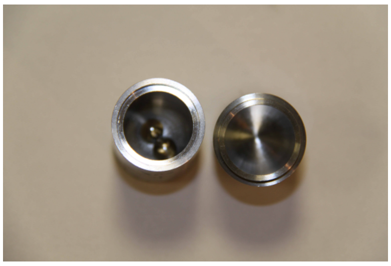

c)

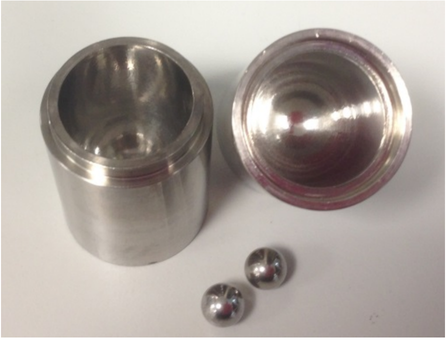

d)

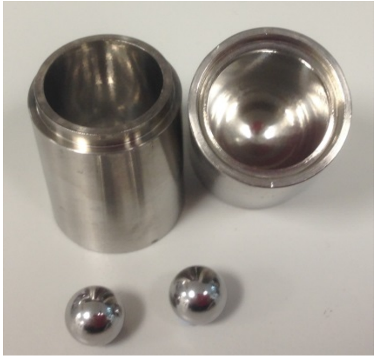

Figure S 10 14.5mL Stainless steel snap grinding jars; a) closed: b & c) open with two 7 mm ID hardened stainless steel ball bearings (each 1.37g). b) inside and c) outside the open grinding jar and d) with two 10 mm ID hardened stainless steel ball bearings (each 4.00g) outside the open grinding jar.

3.3.2 Screw closure grinding jars with Teflon washer

The grinding jars were manufactured in-house from hardened stainless steel with the same internal dimensions and volume (14.5 mL internal volume: 19 mm ID x 54mm internal length) as the snap closure grinding jars discussed in 3.3.1, the closure being a screw closure lined with a Teflon washer to ensure good sealing when the grinding jar is closed. This design should prevent the escape of the vapor phase of the solvent and also prevent powder and added solvent from being trapped in the junction of the closure. These grinding jars were used for system ii for turnover experiments of polymorph interconversion in the absence of dbu (Section 4.4.3 & Section 7.3) and for the solvent experiments (Section 4.5, Section 8, Section 9 and Section 13).

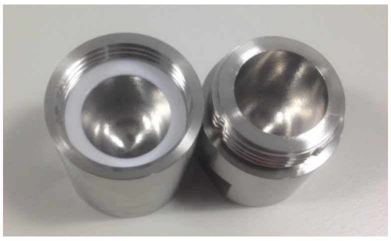

Figure S 11: 14.5mL Stainless steel screw closure grinding jars with Teflon washer

3.3.3 Large scale snap closure grinding jars

Large scale snap-closed grinding jars were manufactured in-house from hardened stainless steel with the same overall outside length as the normal grinding jars so as to fit the Retsch MM400 Shaker Mill. The internal diameter of the hemispheres was set to 38 mm ID, and the grinding jar had 54mm internal length giving an internal volume 46 mL. These grinding jars were used to prepare 1.6 g scales of **Form B** from system ii (Section 4.2.2).

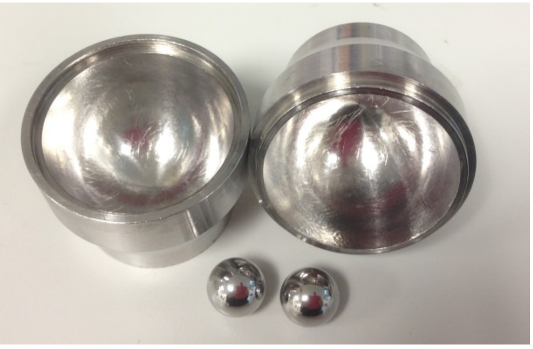

Figure S 12 46 mL large scale stainless steel snap closure grinding jars with two ½ inch ID hardened stainless steel ball bearings (each 8.22g).

3.4 Balance

A Mettler Toledo XPE 205 Delta Range with an accuracy of 0.01mg was used for weighing the starting materials using weighing paper.

3.5 Automatic pipettes

A Sartorius eLINE Picus Electronic Pipette, 1-channel, 5 - 120 μL, in combination with Sartorius SafetySpaceTM, Low Retention, 2-120 μL, Sterile Filter Tip was used to achieve maximum accuracy allowing an accurate increment of 0.1 μL to be pipetted. The systematic error disclosed by the manufacturers in the pipette specification is: for 120μL is ±0.48 μL, for 60 μL is ±0.36 μL and for 12 μL is ±0.24 μL.

The Picus pipette was primed repeatedly for each solvent until the liquid was securely held by the pipette tip before dispensing the selected volume of the solvent.

For direct pipetting as with DMSO, MeOH, EtOH, IPA, water, cyclohexane, benzene, toluene and perfluorodecalin, the full volume of the solvent aspirated by the automatic pipette, was automatically dispensed to the grinding jar; no residual solvent remained in the pipette tip (See Procedure 3 on Section 4.5.3, Procedure 4 on Section 4.5.4 and Procedure 5 on Section 4.5.5).

For reverse pipetting as with MeCN, Acetone, THF, DMF, EtOAc and $CHCl_3$, excess of solvent is aspirated by the Picus pipette and it requires for the tip to rest on a surface (internal wall or the grinding jar or top of ball bearing with no dbu) to accurate dispense the required volume of solvent (See Procedure 1 on Section 4.5.1). This reverse pipetting procedure left a residual volume on the tip which required disposing off to waste by double clicking on the pipette mechanism.

With adequate training, all solvent investigated could be accurately dispensed with exception of dichoromethane (DCM), which due to its very low boiling point (39.6^{o}C) could not be equilibrated in the pipette chamber. For DCM, a gas tight syringe needed to be used (See Procedure 2 on Section 4.5.2).

To evaluate the pipetting skills with the Picus pipette for the different experiments, a calibration by weight was performed using a 5 figure balance of the following solvents: MeCN (Section 8.2.1), Acetone (Section 8.3.1), THF (Section 8.4.1), DMF (Section 8.5.1), EtOAc (Section 8.6.1), $CHCl_3$, (Section 8.7.1), DMSO (Section 8.9.1), MeOH (Section 8.11.1), EtOH (Section 8.12.1) and IPA (Section 8.14.1).

For volumes of solvent above 120 μL, a 20-200 μ Proline Plus Mechanical Pipette with TipOne 200 μL Graduated Filter tip from Starlab was used.

For dispensing 2uL dbu, a 0.5-10 μL Proline Plus Mechanical Pipette with TipOne XL Graduated Filter tip, max volume 20 μL from Starlab was used.

3.6 Hamilton syringes for dispensing DCM

A 50 μL Hamilton gas tight syringe (cat. No 80975- 1705 model) with 51mm length, 22s gauge, 90° bevel point, fixed (cemented), needle recommended for precision dosing was used to dispense DMC in the range 25 to 50 μL.

A 25 μL Hamilton gas tight syringe (cat. No 80275- 1702 model) with 51mm length, 22s gauge, 90° bevel point, fixed (cemented), needle was used to dispensed DMC in the range 10 to 25 μL.

To evaluate the skills with the Hamilton syringe for dispensing DCM, a calibration by weight was performed using a 5 figure balance as documented in Section 8.8.1.

3.7 HPLC equipment & columns

HPLC analysis of the chemical composition of the powder were performed using a modular Agilent 1200 Series HPLC system composed of a HPLC high pressure binary pump, autosampler with injector programming capabilities, Peltier type column oven with 6 µL heat exchanger and a Diode Array Detector with a semi-micro flow cell (1.6uL, 6mm pathlength) to reduce peak dispersion when using short columns as in this case. The flow-path was connected using 0.12 mm ID stainless steel tubing to minimize peak dispersion. For the analysis of system ii, an HPLC method described in Section 5.1 was used.

3.8 PXRD equipment

X-ray powder diffractograms in the 2θ range 4-45° (Cu Kα radiation, step size 0.03°, time/step 100 s, 0.04 rad soller, VxA 40x40) were collected either (1) on an X-Pert PRO MPD powder X-ray diffractometer or (2) on a Panalytical X'Pert Pro diffractometer both equipped with an X'Celerator detector. Both PXRD equipment were available at the Department of Chemistry, University of Cambridge.

4 Experimental Section: methodology

4.1 General concepts

4.1.1 Dynamic covalent Chemistry

Dynamic combinatorial chemistry (DCC) is a method to the generation of new molecules formed by reversible reaction of simple building blocks under thermodynamic control; in a dynamic combinatorial library (DCL) all constituents are in equilibrium under the particular conditions of the experiment.[21] Typically, DCC is performed in solution using reversible chemistries, such as disulfide metathesis[22] and imine exchange.[23-24] DCC has the great advantage of proof reading and self-repair resulting in the most stable and reproducible composition at equilibrium. Furthermore DCC is very sensitive to changes in the experimental conditions and adapts the composition of the product at the equilibrium accordingly. This makes dynamic covalent libraries (DCLs) powerful tools in identifying thermodynamic minima in a number of different contexts.[25]

4.1.2 Solid state dynamic covalent chemistry

Solid state dynamic covalent chemistry (solid state DCC) is an innovative, clean and highly atom-efficient approach to the synthesis of molecules such as system ii. For the reasons listed in Section 4.1.1, DCC can yield valuable tools to study equilibria under ball mill grinding conditions. This one pot reaction is achieved by the improved stability of the crystal structures of the product as compared to that of its starting materials in a reversible reaction governed by thermodynamic control under the given experimental conditions.[3, 20] In contrast; one pot synthesis in traditional solution DCC requires the stabilising effect of a host-guest complex. In the absence of a suitable guest, a statistical mixture of starting material and products can be expected, assuming all components have similar thermodynamic stability. As reported in our "proof of concept" paper[3] based on disulfide exchange chemistry, different polymorphs of the product of system ii can be directly synthesised from starting materials by solvent-free DCC, depending if the grinding is performed in the presence of substochiometric amounts of solvent known as (Liquid Assisted Grinding or **LAG)** or by ball mill neat grinding (**NT**).[3.20]

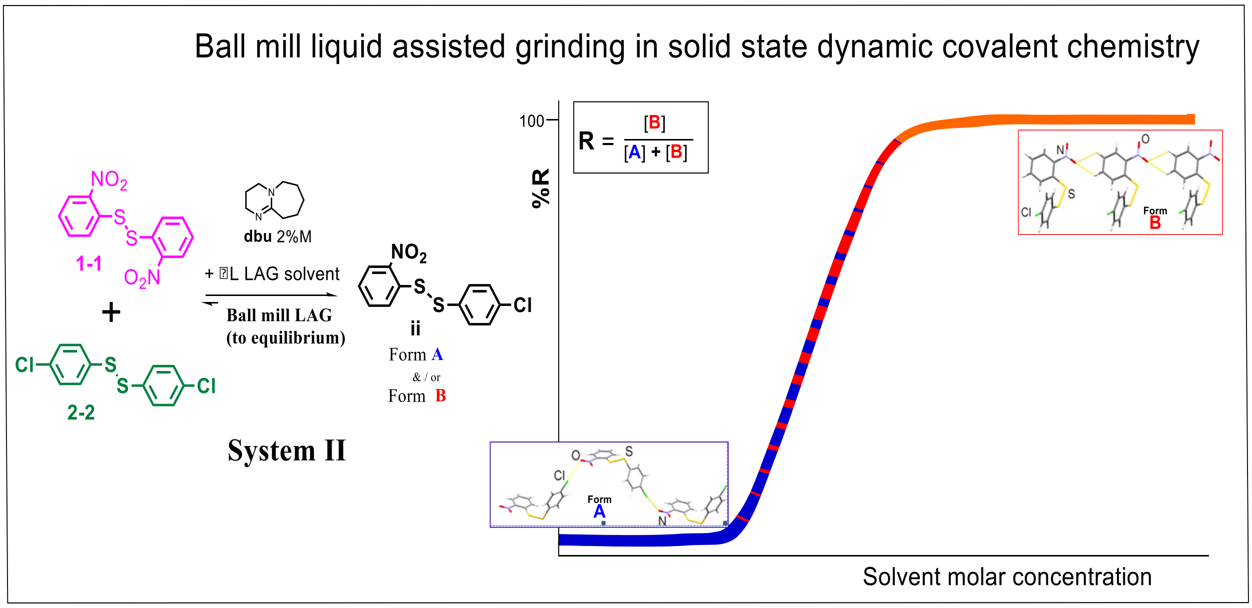

Scheme S 1 Effect of the surface of the nanocrystalline particles by a few drops of solvent determines which polymorph or mixture of the two polymorphs of the product of the reaction presented here, is formed when grinding long enough using a ball mill grinder to achieve equilibrium conditions.

4.2 Preparation of polymorphs of the disulfide heterodimer from System ii: Form A and Form B

Form A and **Form B**, the polymorphs from system ii can only be prepared quantitatively by disulfide exchange from the disulfide homodimers **1-1** and **2-2** using solid state DCC by ball mill grinding.[3, 20] The disulfide exchange reaction is a base catalyzed reaction. We use dbu as the base catalyst. The catalyst dbu can be removed from **Form A** or **Form B** powder by recrystallization and careful washing of the formed crystals.

4.2.1 Preparation of Form A and Form B by solid-state DCC (200 mg scale)

Form A and **Form B** of the $2NO_2PhSSPh4Cl$ disulfide heterodimer was prepared at a 200 mg scale by grinding 0.34 mmol of $(2NO_2PhS)_2$ crystals (104.82mg) and 0.34 mmol of $(4ClPhS)_2$ crystals (97.66mg) in a 14.5mL stainless steel grinding jar. We have used either a snap-closure (See Section 3.3.1) or a screw closure (See Section 3.3.2) grinding jar. Two 7 mm ID stainless steel ball bearings were added and 2μL dbu were pipetted on top of a ball bearing. Nothing else was added to prepare **Form A,** while 50 μL of acetonitrile was typically added to the powder to prepare **Form B**. The grinding jars were closed and secured with tape, and the solid was milled to equilibrium in a Retsch MM400 Shaker Mill typically for 45 minutes at 30 Hz. A conversion of the homodimers to **Form A** and **Form B** was achieved, as demonstrated by HPLC analysis with over 95%M conversion to the heterodimer and the PXRD scans consistent with **Form A** and **Form B**, respectively as shown in Section 5.2.1.

4.2.2 Preparation of large scale amount of Form B by solid-state DCC (1.6g scale)

1.6 g scale batches of **Form B** were prepared by grinding 1.36 mmol of $(2NO_2PhS)_2$ crystals (836.66mg) and 1.36 mmol of $(4ClPhS)_2$ crystals (781.27mg) in a large scale snap closured grinding jar (See Section 3.3.3). Two ½" stainless steel ball bearings were added and 5μL dbu were pipetted on top of a ball bearing. The grinding jar was snap-closed and secured with tape, and the solid was milled in a Retsch MM400 Shaker Mill for 20 minutes at 30 Hz. A conversion of the homodimers to **Form B** was achieved, as demonstrated by PXRD (see Section 5.2.1) with over 95% conversion to the heterodimer by HPLC analysis.

4.2.3 Preparation of crystals of Form B

Crystals of **Form B (**CSD refcode FUQLIM) are easily obtained by warming a saturated solution of the powder of **Form B** from various solvents to 50-60°C such as MeOH and IPA. As **Form B** had been obtained by solid state DCC as described in Section 4.2.1 and Section 4.2.2 and contains dbu, an equivalent amount of TFA was added to the solution to neutralize the dbu. This was done to avoid **Form B** from scrambling and forming a statistical mixture of the DCC library (**1-1**, **2-2** and **1-2**). Triclinic crystals of **Form B** (CCDC code FUQLIM) were obtained from crystallization from IPA, MeOH and MeCN. Unfortunately all crystals of **Form B** were contaminated with around 1%M of $(2NO_2PhS)_2$ but showed no presence of $(4ClPhS)_2$. The collected crystals of **Form B** were rinsed with pure solvent to remove any trace of TFA and dbu.

These batches of crystals once dried were used for the solubility studies (See Section 4.7 and Section 10), slurry experiments (See Section 11 and 12) and for the direct polymorph interconversion turnover experiments (See Section 4.4.3 and Section 7.3). Other solvents such as DCM and $CHCl_3$ were investigated giving large crystals of **Form B**. These small batches were not used for experimental studies.

4.2.4 Preparation of crystals/powder of Form A

Despite Tomislav Friščić having previously prepared the monoclinic crystals of **Form A** which he used to obtain the crystal structure (CCDC FUQLIM01, code 796996),[3] we have not been able to obtain crystals of **Form A** despite trying different solvent systems and recrystallization methodologies. We always obtain crystals of **Form B**, the thermodynamically stable polymorph of **1-2** under crystallization or slurry

conditions. We have demonstrated in our slurry studies in 15 solvents (see Section 12.2), that **Form A** is the metastable polymorph at the macroscale and experiences polymorphic interconversion to the thermodynamic polymorph **Form B** immediately or with time under slurry conditions. We prepared **Form A** by direct polymorphic interconversion from crystals of **Form B** which does not contain dbu. This polymorphic interconversion was achieved by ball mill neat grinding at 30 Hz over 150 minutes, 200 mg of the crystals of **Form B** in a 14.5 mL grinding jar using two 7 mm diameter stainless steel ball bearings. The resulting powder of **Form A** (quantitative) was so fine and static that **Form A** could not be transferred from the grinding jar unless a little bit of solvent (IPA, MeOH or MeCN) was added to the fine powder and lightly ground with Retsch MM400 Shaker Mill for a few seconds to impart the resulting powder a sticky texture which allowed the powder to be easily handled. 50μL IPA was therefore added to the grinding jar containing the very fine powder of **Form A** and the fine powder was milled for an extra 20 seconds @ 20 Hz. No polymorph interconversion to **Form B** was experienced with this short grinding period in IPA, MeOH or MeCN as confirmed by Powder XRD analysis. The powder of **Form A** could only be transferred while it was moist with a solvent, as **Form A** becomes very static and impossible to transfer once it becomes dry.

4.3 Experimental procedures: kinetic studies and turnover experiments of system ii by polymorph interconversion between Form A and Form B in solid state DCC by ball mill grinding (contains dbu)

4.3.1 Procedure: Kinetic studies of polymorph transformation of Form B to Form A in solid state DCC under ball mill Neat grinding conditions (contains dbu)

Form B material used for the kinetic experiments was obtained by ball mill LAG for 45 minutes at 30 Hz in a Retsch MM400 Shaker Mill equimolar amounts of **1-1** and **2-2** (total amount 200 mg) with 2%M dbu and 50μL of MeCN using two 7mm stainless steel ball bearings. Around 200 mg of **Form B** was accurately weighed with a 5 decimal figure balance and transferred accurately to one half of a 14.5 mL internal volume, homemade snap closure stainless steel grinding jar. The composition at equilibrium of **Form B** is: 97%M **Form B**, 1.5%M **1-1** and 1.5%M **2-2**. To the half of the grinding jar containing the weighed **Form B**, two 7 mm-diameter hardened stainless steel grinding balls were added. The two halves of the grinding jar were immediately snap-closed. The mixture was then milled at ambient temperature for the allocated time at 30 Hz in a Retsch MM400 Shaker Mill. The grinding jar was opened within 10 minutes of completion of the grinding time and the reaction product was removed from the grinding jar with a metal spatula. The PXRD samples were prepared and immediately run. The product mixture was then transferred to an amber screw top glass vials and stored in the fridge. The product mixture was analysed by HPLC as soon as possible and always within the same day of preparation.

4.3.2 Procedure: Kinetic studies of polymorph transformation of Form A to Form B in solid state DCC under ball mill LAG conditions with 50μL MeCN (contains dbu)

Form A material used for the kinetic experiments was obtained by ball mill neat grinding for 45 minutes at 30 Hz in a Retsch MM400 Shaker Mill equimolar amounts of **1-1** and **2-2** (total amount 200 mg) with 2%M dbu using two 7mm stainless steel ball bearings. Around 200 mg of **Form A** was accurately weighed with a 5 decimal figure balance and transferred accurately to one half of a 14.5 mL internal volume, homemade hardened snap closure stainless steel grinding jar. The composition at equilibrium of **Form A** is: 97%M **Form A** , 1.5%M **1-1** and 1.5%M **2-2**. To the half of the grinding jar containing the weighed **Form A**, two 7 mm-diameter hardened stainless steel grinding balls were added. 50 μL acetonitrile was directly added to the solid mixture using a 10-100μL automatic pipette. The two halves of the grinding jar were immediately snap-closed. The mixture was then milled at ambient temperature for the allocated time at 30 Hz in a Retsch MM400 Shaker Mill. The grinding jar was opened within 10 minutes of completion of the grinding time and the reaction product was removed from the grinding jar with a metal spatula. The PXRD samples were prepared and immediately run. The product mixture was

then transferred to an amber screw top glass vials and stored in the fridge. The product mixture was analysed by HPLC as soon as possible and always within the same day of preparation.

4.3.3 Procedure: Turnover experiments of polymorph interconversion in System ii between Form A & Form B in solid state DCC by ball mill grinding (contains dbu)

This experiment was started by ball mill LAG for 45 minutes at 30 Hz in a Retsch MM400 Shaker Mill equimolar amounts of **1-1** and **2-2** (total amount 200 mg) with 2%M dbu and 50µL MeCN using two 7mm stainless steel ball bearings. The grinding jar was opened within 10 minutes of completion of the grinding time and the reaction product was removed from the grinding jar with a metal spatula. The PXRD samples were prepared and immediately run and shown to be quantitative **Form B**. The powder from the PXRD sample slide was quantitatively returned to the grinding jar. The grinding jar was left open in the fumehood for a few hours to ensure the MeCN was fully evaporated. The grinding jar containing now dried **Form B** was allowed to ball mill neat grind for 45 minutes@30 Hz. As before, the snap grinding jar was opened and powder was transferred to a PXRD slide consistent with quantitative **Form A**. The powder was again quantitatively transferred to the snap closure grinding jar, 50µL MeCN added, snap closed and allowed to ball mill LAG for 90 min@30 Hz. This cycle was repeated 4 extra times for the 1st try (Section 6.3.1) and 2 extra times for the 2nd try (Section 6.3.2).

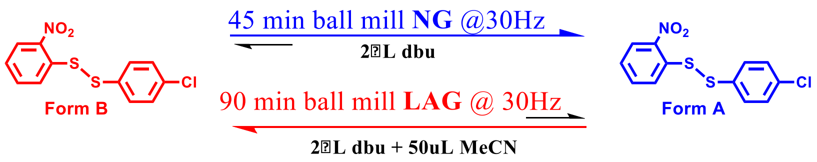

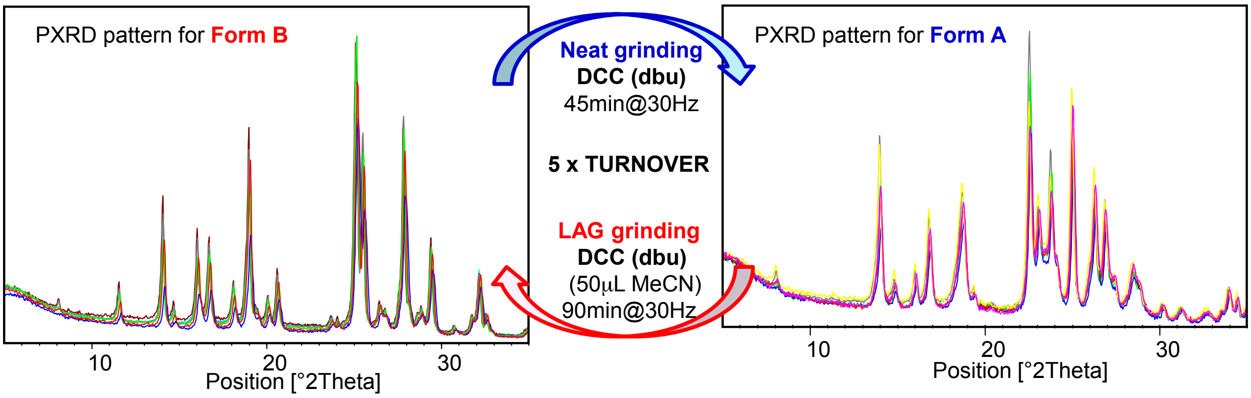

Figure S 13 Superimposed powder diffractograms demonstrating polymorph conversions in system (ii) using base catalyst (dbu) under NG and LAG conditions with 50 µL of MeCN. The "turnover" polymorph conversion experiment has been repeated 5 times.

4.4 Experimental procedures: kinetic studies and turnover experiments of system ii by direct polymorph interconversion between Form B and Form A (absence of catalyst)

4.4.1 Procedure: Kinetic studies: direct polymorph transformation of Form B to Form A under ball mill neat grinding conditions (absence of catalyst)

Around 200 mg of crystals of **Form B** obtained as explained in Section 4.2.3 were accurately weighed with a 5 decimal figure balance and transferred accurately to one half of a 14.5 mL internal volume, homemade snap closure stainless steel grinding jar. The chemical composition of **Form B** crystals is: 98.5%M **Form B**, 1.5%M **1-1** and 0%M **2-2**. To the half of the grinding jar containing the weighed crystals of **Form B**, two 7 mm-diameter hardened stainless steel grinding balls were added. For ball mill neat grinding we added nothing else. The two halves of the grinding jar were immediately snap-closed. The mixture was then milled at ambient temperature for the allocated time at 30 Hz in a Retsch MM400 Shaker Mill. The grinding jar was opened within 10 minutes of completion of the grinding time and the reaction product was removed from the grinding jar with a metal spatula. As explained in Section 4.2.4 **Form A** prepared in this way was very fine and static and much too difficult to transfer unless a little bit of solvent (IPA, MeOH or MeCN) was added to the fine powder and lightly ground with Retsch MM400 Shaker Mill for a few seconds to impart the resulting powder a sticky texture which allowed the powder to be easily handled. The PXRD samples were prepared and immediately run. The product mixture was then transferred to an amber screw top glass vials and stored in the fridge. The product mixture was analysed by HPLC as soon as possible and always within the same day of preparation. The kinetic experiments are reported in Section 7.1.

4.4.2 Procedure: Kinetic studies: direct polymorph transformation of Form A to Form B under ball mill LAG conditions with 50μL MeCN (absence of catalyst)

Form A material used for the kinetic experiments was obtained by ball mill neat grinding for 45 minutes at 30 Hz in a Retsch MM400 Shaker Mill equimolar amounts of **1-1** and **2-2** (total amount 200 mg) with 2%M dbu using two 7mm stainless steel ball bearings. To neutralize the 2μL bdu catalyst incorporated in 200 mg of **Form A**, 2μL of HCl was added and ball mill ground for one additional minute. Around 200 mg of **Form A** (absent of dbu) were accurately weighed with a 5 decimal figure balance and transferred accurately to one half of a 14.5 mL internal volume, homemade snap closure stainless steel grinding jar. The composition at equilibrium of **Form A** is: 97%M **Form A** , 1.5%M **1-1** and 1.5%M **2-2**. To the half of the grinding jar containing the weighed **Form A**, two 7 mm-diameter hardened stainless steel grinding balls were added. For the LAG, 50 μL acetonitrile was directly added to the solid mixture using a 10-100μL automatic pipette. The two halves of the grinding jar were immediately snap-closed. The mixture was then milled at ambient temperature for the allocated time at 30 Hz in a Retsch MM400 Shaker Mill. The grinding jar was opened within 10 minutes of completion of the grinding time and the reaction product was removed from the grinding jar with a metal spatula. The PXRD samples were prepared and immediately run. The product mixture was then transferred to an amber screw top glass vials and stored in the fridge. The product mixture was analysed by HPLC as soon as possible and always within the same day of preparation. The kinetic experiments are reported in Section 7.2.

4.4.3 Procedure: Turnover experiments: direct polymorph interconversion between Form A and Form B by ball mill grinding (absence of catalyst)

Before starting these experiments, around 1g of **Form B** crystals which do not contain any dbu base catalyst, obtained as in Section 4.2.3, were manually slightly ground with mortar and pestle, the powder transferred to an open glass beaker and the powder allowed to equilibrate overnight inside a high humidity desiccator. **Form B** crystals contains 1.5%M of **1-1** but no **2-2**.

200 mg of the humid powder of the original **Form B** crystals were accurately weighed with a 5 decimal figure balance and transferred accurately to one half of a 14.5 mL internal volume, homemade snap

closure stainless steel grinding jar. Two 7mm ID hardened stainless steel ball bearing were added and the grinding jar was snap-closed and secured with insulating tape. For neat grinding nothing else was added and the powder was ball mill neat ground for 3x50 minutes at 30 Hz in a Retsch MM400 Shaker Mill. On completion of the grinding time, the grinding jar was opened. The resulting powder was very fine and extremely static. A procedure of adding 50μL IPA and grinding the powder for just 20 seconds at 20 Hz was found to result in a sticky powder which was easy to transfer without affecting the solid state composition. This sticky powder could be easily transferred to the PXRD sample slide as far as this was done quickly before the powder became dry and static. The PXRD pattern of the powder was consistent with quantitative **Form A**. After quantitatively returning the powder from the PXRD slide to the grinding jar, the jar was left open for a few hours in a fumehood to remove any residual IPA. 50μL MeCN was added to the fine powder of **Form A**, the two halves of the grinding jar were snap-closed and secured with insulating tape. The powder was then ball mill LAG for 30 minutes at 30 Hz. Immediately on opening the grinding jar, the powder was found sometimes to be much too fine and static for easy transfer to the PXRD slide. In those cases, 50μL IPA was added to the powder, the two halves of the grinding jar were snap-closed and ball mill ground for just 20 seconds at 20 Hz. The wet powder could be easily transferred to the PXRD slide which gave a scan consistent with quantitative **Form B**. This completes the 1st cycle of the turnover experiment. For the next turnover cycle, the powder in the PXRD slide was quantitatively transferred to the grinding jar, and the grinding jar left open in a fumehood for a few hours to ensure full removal of any residual solvent. Four additional turnover experiments were performed.

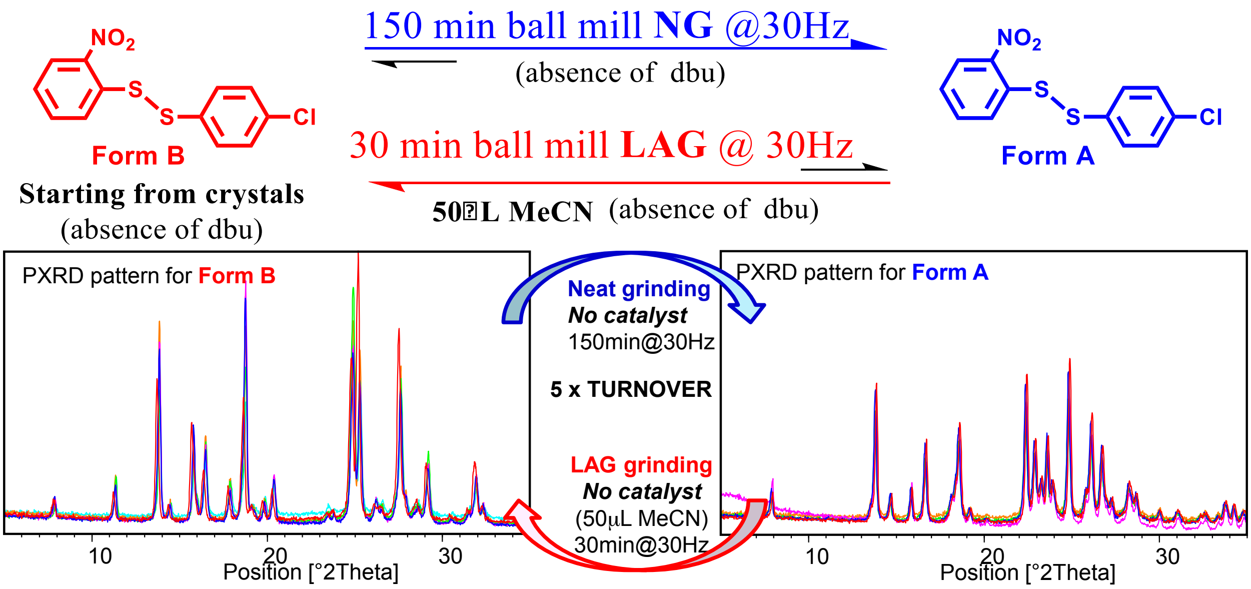

Figure S 14 Superimposed powder diffractograms demonstrating direct polymorph conversions in system (ii) in the absence base catalyst under NG and LAG conditions with 50 μL of MeCN. The "turnover" polymorph conversion experiment has been repeated 5 times.

4.5 Procedures for ball mill LAG of System (ii) under solid state DCC conditions using different LAG solvents (presence of dbu)

For experimental convenience we have performed these experiments using solid state DCC starting from the homodimers **1-1** and **2-2** in the presence of the base catalyst **dbu** leading to disulfide exchange (See Section 4.1.2). The, same outcome could have been obtained starting from the polymorphs **Form A** or **Form B** of the disulfide heterodimer as far as we allowed equilibration to be achieved (See Section 4.3 and Section 6). The same outcome can also be achieved starting the polymorphs **Form A** and **Form B** in the absence of base catalyst dbu (See Section 4.4 and Section 7), as far as we allowed equilibration to be achieved, however kinetics are much slower.

4.5.1 PROCEDURE 1: Ball mill grinding 1:1 $(2NO_2PhS)_2$: $(4ClPhS)_2$+2%M dbu to equilibrium with MeCN, Acetone, THF, DMF, EtOAc, and $CHCl_3$ as LAG solvents.

This experimental procedure is general for ball mill grinding with the following LAG liquids: acetonitrile (MeCN), acetone, tetrahydrofuran (THF), dimethylformamide (DMF), Ethyl Acetate (EtOAc) and chloroform ($CHCl_3$). These solvents have proven to soak readily into the powder (**1-1**+**2-2**) inside the grinding jar. $(2NO_2PhS)_2$ designated as **1-1** is used as the crystalline solid obtained from Sigma Aldrich, while the large crystalline platelets of $(4ClPhS)_2$ designated as **2-2** which is manufactured by TCI, are manually pre-ground with a mortar and pestle to obtain a medium fine powder to facilitate weighing to 0.01mg accuracy. 0.34 mmol of each disulfide homodimer, **1-1** (104.82mg) and **2-2** (97.66mg) as discussed in Section 4.2.1, were weighed with a 5 decimal figure balance and transferred accurately to one half of a screw closure grinding jar. The total weight of the homodimers added up to around 200 mg. The solid chemicals were thoroughly mixed in the grinding jar with a microspatula until homogeneity of the powder was achieved. To the half of the grinding jar containing the weighed powders, two 7 mm-internal diameter hardened stainless steel ball bearings were added. 2μL of dbu was carefully deposited on top of one ball bearing using a 1-10μL automatic pipette. The given volume in μL of the respective grinding liquids (MeCN, Acetone, THF, DMF, EtOAc and $CHCl_3$) was directly added by reverse pipetting (see Section 3.5) using a 10-120μL automatic pipette over the powder taking care not to touch it. The tip of the pipette was rested at the end of the delivery on the internal wall of the grinding jar or on top of the other ball bearing, therefore ensuring accurate transfer of the volume of solvent to the grinding jar. The residual solvent on the pipette tip was disposed of. The two halves of the grinding jar were immediately hand tight screwed together to avoid evaporation and leakage during ball mill grinding of the grinding liquid. The junction was secured with insulating tape. The mixture was then milled at 30 Hz in a Retsch MM400 Shaker Mill at ambient temperature (19-22^oC) for an extended period of time to ensure thermodynamic control was achieved. The required repeat grinding (i.e. 5h = 5 x 1h) was achieved by using an in-house automatic finger (see Section 3.2.2) allowing a rest of 5-10 minutes in between grinding period to prevent the motor from overheating. On completion of the set grinding period, the grinding jar was immediately opened. In some cases, the grinding jar had to be secured on a vice and opened with a large spanner to loosen the screw fitting. The solid was immediately transferred with a metal spatula to a mortar and gently ground with a pestle to prepare the PXRD slide. The PXRD slide was immediately scanned, always within 5 minutes from the completion of the set grinding time. If the PXRD equipment was not available when required, the unopened grinding jars were further ground for a few minutes at 30Hz to ensure that the powder was kept under thermodynamic control when opened. This further grinding should not affect the solid state composition of the sample at equilibrium. The solid was analysed by HPLC for chemical composition immediately or stored in the fridge until it could be analysed within the same day.

Experimental procedure 1 depicted in Figure S 15 resulted in an excellent correlation between the volume of LAG solvent added and the formation of %R, R being the formation of **Form B** normalized to the total amount of **1-2** formed in the solid state DCC reaction. See Section 8.2 for MeCN, Section 8.3 for Acetone, Section 8.4 for THF, Section 8.5 for DMF, Section 8.6 for EtOAc and Section 8.7 for $CHCl_3$. Procedure 1 resulted in very poor correlation between the volume of LAG solvent added and the formation of %R for MeOH (Section 8.10) and IPA (Section 8.13).

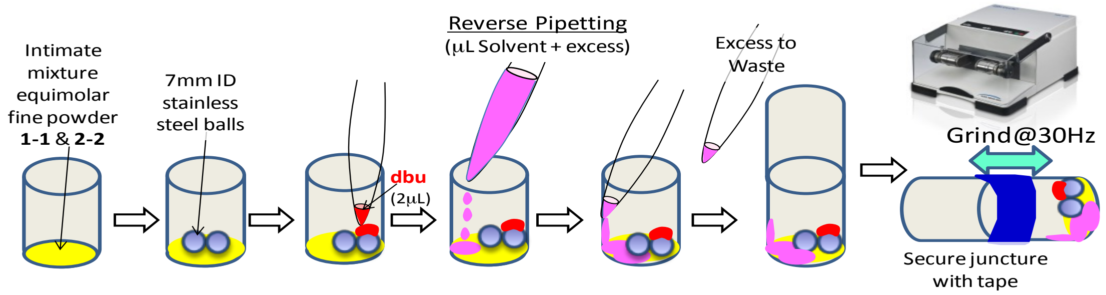

Figure S 15: Experimental Procedure 1 for preparation of (ii) using MeCN, acetone, THF, DMF, EtOAc & $CHCl_3$ as LAG solvents

4.5.2 PROCEDURE 2: Ball mill grinding 1:1 $(2NO_2PhS)_2$: $(4ClPhS)_2$+2%M dbu to equilibrium with DCM as LAG solvents.

Although DCM soaks easily in the powder (**1-1**+**2-2**) inside the grinding jar, it has a very low boiling point (39-40°C). The low boiling point does not make it possible to dispense dichloromethane (DCM) with an automatic pipette; therefore a suitable glass syringe is used (see Section 3.6). This experimental procedure 2 depicted in Figure S16 has been designed to prevent unnecessary evaporation of DCM while preparing the solid state DCC reaction.

0.34 mmol of each homodimeric disulfides **1-1** (104.82mg) and **2-2** (97.66mg) were weighed with a 5 decimal figure balance and transferred accurately to one half of a 14.5 mL internal volume, homemade stainless steel screw-closure grinding jar. The total weight of the homodimers was around 200 mg. The solid chemicals were mixed with a microspatula until homogeneity was achieved. To the half of the grinding jar containing the weighed solids, two 7 mm-internal diameter hardened stainless steel balls bearings were added. A tall heap was made of the powder between the stainless steel balls. 2μL of dbu was carefully added on top of one ball bearing using a 1-10μL automatic pipette ensuring the ball with the dbu was not disturbed. A shaped piece of grease-proof weighing paper was pierced and located at the top of the needle of the glass syringe. DCM was dispensed at a steady rate with a 25 μL or a 50 μL (See section 3.6), inserting the tip of the needle deep inside the heap of powder. The syringe barrel was pressed to the end to displace all the DCM. To avoid the residual solvent contained in the needed being transferred to the powder by capillary action, the syringe was immediately lifted from the powder and the pierced weighing paper moved down the needle to transfer any attached powder back into the grinding jar. The two half of the grinding jar were screw closed and the junction was secured with an insulating tape. The mixture was then milled at 30 Hz in a Retsch MM400 Shaker Mill at ambient temperature (19-22°C) for an extended period of time to ensure thermodynamic control was achieved. The required repeat grinding (i.e. 4h = 4 x 1h) was achieved by using an in-house automatic finger (see Section 3.2.2) allowing a rest of 5-10 minutes in between grinding period to prevent the motor from overheating. On completion of the experiment the solid was analysed by HPLC for chemical composition and by PXRD for solid state composition as discussed in experimental procedure 1. See Section 8.8 for experimental data using DCM as the LAG solvent.

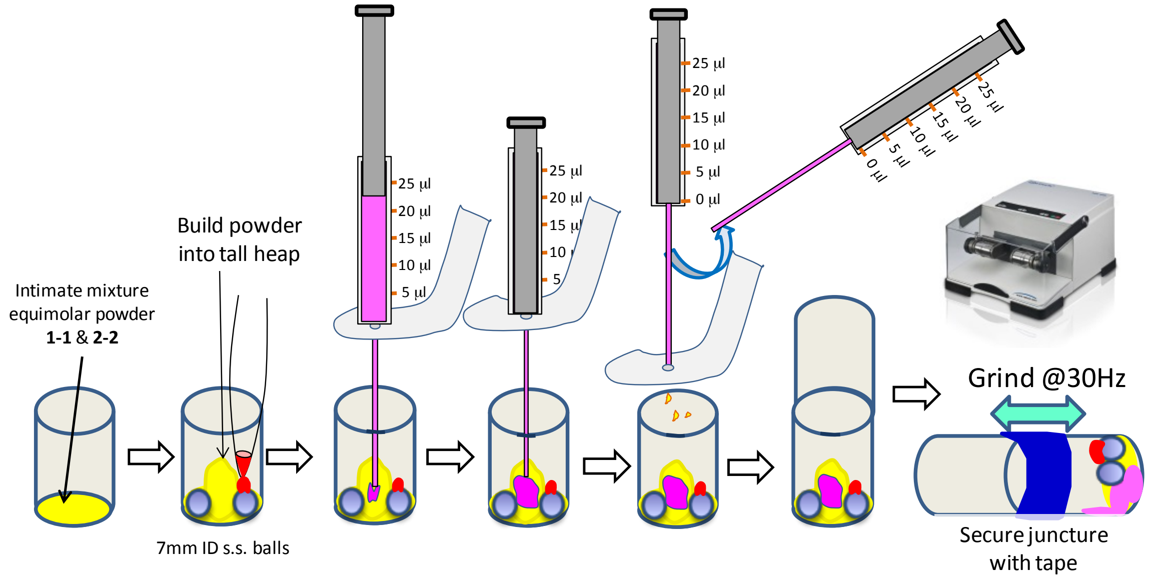

Figure S 16: Experimental Procedure 2 for preparation of (ii) using DCM as a LAG solvent

4.5.3 PROCEDURE 3: Ball mill grinding 1:1 $(2NO_2PhS)_2$: $(4ClPhS)_2$+2%M dbu to equilibrium with poorly wetting LAG solvents (MeOH).

This experimental procedure was used for grinding the LAG liquids which were found to soak poorly into the powder (**1-1**+**2-2**) under experimental procedure 1. Experimental procedure 3, depicted in Figure S17, resulted in a good correlation for MeOH (See Section 8.11) between the solvent added and the formation of %R, R being the formation of **Form B** normalized to the total amount of **1-2** formed in the solid state DCC reaction. However, experimental procedure 3 resulted in very poor correlation for IPA (not reported here), this solvent probably soaks much slower into the powder than MeOH does.

0.34 mmol of each homodimeric disulfides **1-1** (104.82mg) and **2-2** (97.66mg) were weighed with a 5 decimal figure balance and transferred accurately to one half of a 14.5 mL internal volume, homemade, hardened stainless steel screw-closure grinding jar. The total weight of the homodimers was around 200 mg. The solid chemicals were mixed with a microspatula until homogeneity was achieved. Around 60mg of the powder was then transferred to a weighing paper and reserved for later use in the procedure. Using normal pipetting (See section 3.5), the desired volume of MeOH or IPA was added to the powder carefully avoiding contact of the pipette tip with the powder. The pipette tip was rested at the end of the delivery on the internal wall of the grinding jar to quantitatively transfer the solvent to the grinding jar. The wetted area of the powder was immediately covered with the reserved powder, swirling and tapping the grinding jar to ensure that as much of the dry powder was in intimate contact with the solvent as possible. The bottom part of the half grinding jar was fixed to the bench with Bluetak. This was done to avoid later on in the procedure the ball bearing with dbu on the top, from rolling and touching the powder during the resting time. All subsequent steps were performed as quickly as possible to avoid the solvent from evaporating. Two 7 mm diameter hardened stainless steel ball bearing were added to the grinding jar. 2μL of dbu was carefully added on top of one ball bearing using a 1-10μL automatic pipette. On completion of the resting period, the screw closure was then firmly hand tightened and secured with insulating tape. The mixture was then milled at 30 Hz in a Retsch MM400 Shaker Mill at ambient temperature (19-22°C) for an extended period of time to ensure thermodynamic control was achieved. The required repeat grinding (i.e. 5h = 5 x 1h) was achieved by using an in-house automatic finger (see Section 3.2.2) allowing a rest of 5-10 minutes in between grinding period to prevent the motor from overheating. On completion of the experiment, the solid was analysed by HPLC for chemical composition and by PXRD for solid state composition as discussed in experimental procedure 1.

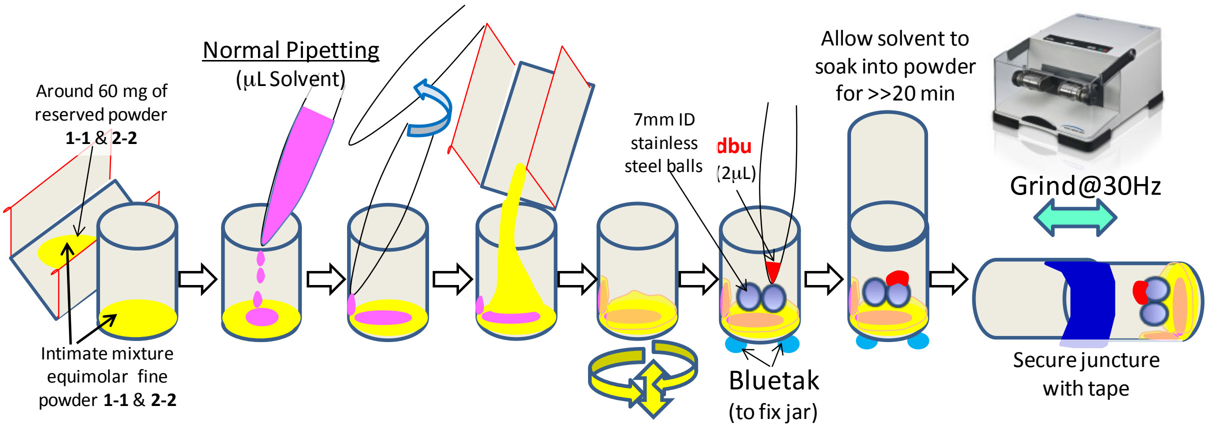

Figure S 17: Experimental Procedure 3 for preparation of (ii) using IPA and MeOH as LAG solvents (proved unsuitable for IPA)

4.5.4 PROCEDURE 4: Ball mill grinding 1:1 $(2NO_2PhS)_2$: $(4ClPhS)_2$+2%M dbu to equilibrium with all poorly wetting LAG solvents (excluding water).

This experimental procedure depicted in Figure S18 is general for grinding experiments with all the LAG liquids such as MeOH, EtOH (Section 8.12), IPA (Section 8.14) and DMSO (Section 8.9) shown to soak very slowly onto the surface of the powder composed by **1-1**+**2-2**. Experimental procedure 4 has been also used for all those liquids such as cyclohexane (Section 8.16), benzene (Section 8.17), toluene (Section 8.18) and perfluorodecaline (Section 8.19)that did not result in any formation of **Form B**; this procedure being adopted in order to allow the solvent added to soak into the surface of the powder. Experimental procedure 5, a modification of procedure 4, will be used specifically for water which does not form **Form B** either. Experimental procedure 4 is a modification of procedure 3, discussed previously in which a) the liquid must be now dripped carefully onto the center of the powder enabling the powder to soak the added solvent and b) avoiding contact of the tip of the pipette with the internal walls of the grinding jar. We hypothesise that the kinetics of absorption of these liquids onto the surface of the powder is so slow, that any drop of liquid not directly in contact with the powder will not have the ability to be absorbed into the powder during ball mill grinding.

0.34 mmol of each homodimeric disulfides **1-1** (104.82mg) and **2-2** (97.66mg) were weighed with a 5 decimal figure balance and transferred accurately to one half of a 14.5 mL internal volume, homemade, hardened stainless steel screw-closure grinding jar. The total weight of the homodimers was around 200 mg. The solid chemicals were mixed with a microspatula until homogeneity was achieved. Around 60mg of the powder was then transferred to a weighing paper and reserved for later use in the procedure. Using normal pipetting (See section 3.5), the desired volume of the solvents was dripped onto the center of the powder taking care not to touch the internal wall of the grinding jar. The pipette tip could be quickly rested at the end of the delivery on the powder to quantitatively transfer the solvent, the powder not adhering to the pipette tip. The wetted area of the powder was immediately covered with the reserved powder, swirling and tapping the grinding jar to ensure that as much of the dry powder was in intimate contact with the solvent as possible. The bottom part of the half grinding jar was fixed to the bench with Bluetak. This was done to avoid later on in the procedure the ball bearing with dbu on the top, from rolling and touching the powder during the resting time. All subsequent steps were performed as quickly as possible to avoid the solvent from evaporating. Two 7 mm diameter hardened stainless steel ball bearing were added to the grinding jar. 2μL of dbu was carefully added on top of one ball bearing using a 1-10μL automatic pipette. The two halves of the grinding jar were screw closed and the grinding jar was left undisturbed to rest for a set period of time to encourage the solvent to soak into the surface of the powder. On completion of the resting period, the screw closure was then firmly hand tightened and secured with insulating tape. The sealed grinding jar was milled at ambient temperature (19-22°C) for an extended period of time at 30 Hz in a Retsch MM400 Shaker Mill to ensure equilibrium was achieved. The resulting powder was analysed by HPLC for chemical composition and by PXRD for solid state composition as discussed in experimental procedure 1.

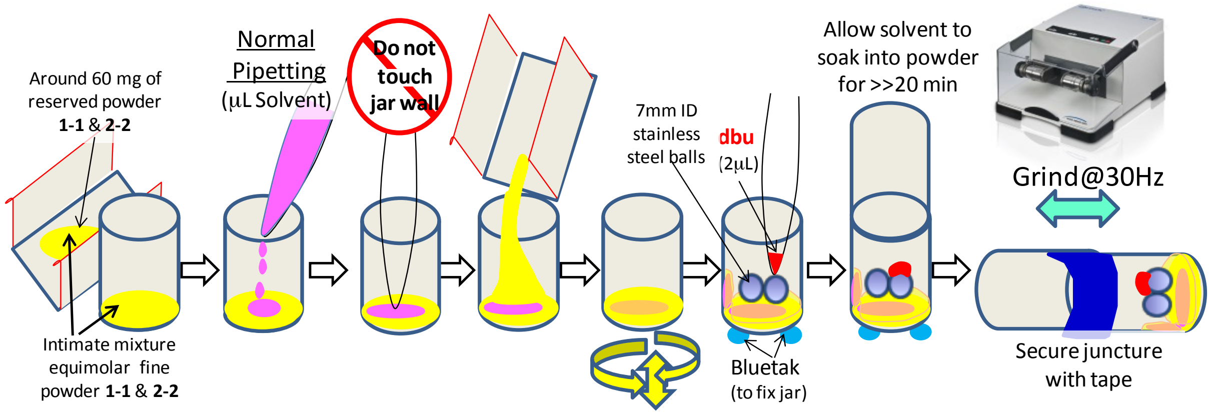

Figure S 18: Experimental Procedure 4 for preparation of (ii) using MeOH, EtOH, IPA, DMSO, cyclohexane, benzene, toluene and perfluoro-decalin as LAG solvents

4.5.5 PROCEDURE 5: Ball mill grinding 1:1 $(2NO_2PhS)_2$: $(4ClPhS)_2$+2%M dbu to equilibrium with water as LAG solvent

This experimental procedure depicted in Figure S19 is specific for grinding experiments with water, as water forms a globule on top of the powder to avoid contact with the powder. Experimental procedure 5 is a modification of procedure 4.

0.34 mmol of each homodimeric disulfides **1-1** [$(2NO_2PhS;)_2$; 104.82mg] and **2-2** [$(4ClPhS)_2$; 97.66mg] were weighed with a 5 decimal figure balance and transferred accurately to one half of a 14.5 mL internal volume, homemade, hardened stainless steel screw-closure grinding jar. The total weight of the homodimers was around 200 mg. The solid chemicals were mixed with a microspatula until homogeneity was achieved. Around 60mg of the powder was then transferred to a weighing paper and reserved for later use in the procedure.

Using normal pipetting (see Section 3.5), the desired volume of water was dripped onto the center of the powder taking care not to touch the internal surface of the grinding jar. The droplets of water immediately coalesced into a large globule of water. The tip of the pipette could be quickly rested at the end of the delivery on the powder to quantitatively transfer the solvent, the powder not adhering to the tip. One ball bearing was added to break the globule of water. On adding the ball bearing, this ball bearing was engulfed by the water globule as shown in Figure S19. By tapping and swirling the grinding jar for over one minute, the water globule was transferred from the ball bearing to the powder now as very small droplets. The area of the powder containing the small droplets of water was immediately covered with the reserved powder, swirling and tapping the grinding jar to ensure that as much of the dry powder was in intimate contact with the solvent as possible. The bottom part of the half grinding jar was fixed to the bench with Bluetak. This was done to avoid later on in the procedure the ball bearing with dbu on the top, from rolling and touching the powder during the resting time. The other 7 mm diameter hardened stainless steel ball bearing was added to the grinding jar and 2μL of dbu was carefully added on top of this ball bearing using a 1-10μL automatic pipette. The two halves of the grinding jar were screw closed and the grinding jar was left undisturbed to rest for a set period of time to encourage the water to soak into the surface of the powder. This period of time ranged between 30 minutes and 24 hours, apparently making little difference on the outcome. On completion of the resting period, the two half part of the grinding jar were then firmly hand tightened and secured with insulating tape. The sealed grinding jar was milled at ambient temperature (19-22°C) for an extended period of time at 30 Hz in a Retsch MM400 Shaker Mill to ensure equilibrium was achieved. The required repeat grinding (i.e. 5h = 5 x 1h) was achieved by using an in-house automatic finger (see Section 3.2.2) allowing a rest of 5-10 minutes in between grinding period to prevent the motor from overheating. The resulting powder was analysed by HPLC for chemical composition and by PXRD for solid state composition as discussed in experimental procedure 1.

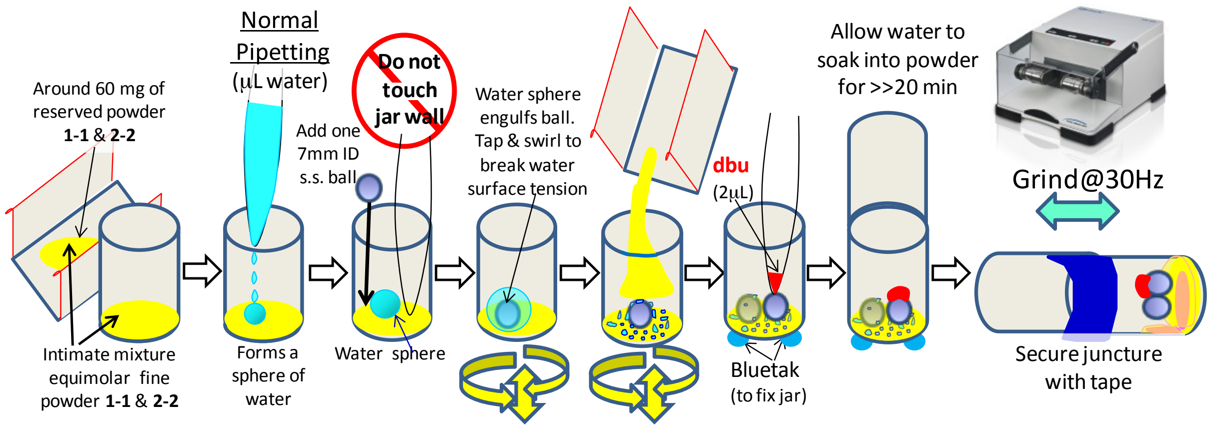

Figure S 19: Experimental Procedure for preparation of (ii) using for water as the LAG solvent

4.6 Procedures for preparation of Theophylline: Benzamide cocrystals

The method for the preparation of Form I and Form II was taken from the publication by Fischer et al.[26]

4.6.1 Preparation of Form I of Theophylline: Benzamide cocrystal

Theophylline anhydrous (3.32mmol; 598.0 mg] and Benzamide (3.32mmol; 402.0 mg], were weighed with a 5 decimal figure balance and transferred accurately to one half of a homemade 14.5mL snap closure stainless steel grinding jar. The total weight of the homodimers added up to around 1g. The solid chemicals were thoroughly mixed in the grinding jar with a microspatula until homogeneity of the powder was achieved. To the half of the grinding jar containing the weighed powders, two 10 mm-internal diameter hardened hardened stainless steel ball bearings (4.0g) were added. Nothing else or 250uL cyclohexane was added to the solid before snap closing the two halves of the grinding jars. The junction was secured with insulating tape. The mixture was then milled at 30 Hz in a Retsch MM400 Shaker Mill at ambient temperature (19-22°C) for period of time in excess to 25 minutes to ensure thermodynamic control was achieved resulting in quantitative formation of **Form I** cocrystal. The resulting mixture was checked by PXRD for homogeneity, as 1 g payload is 5 times the payload normally run by our group. PXRD samples were prepared from 5 different locations within the powder in the grinding jar.

Table S 6 Homogeneity of the formation of **Form I** cocrystal of 1:1 Theophylline: Benzamide by ball mill NG

Preparation of 1:1 Theophylline:Benzamide cocrystal **Form I**									
Sample homogeneity									
**Ball mill grinding** experimental conditions				**Starting coformers** weighing (mg)		**Reaction product of ball mill neat grinding** Phase composition			
ss balls	Payload	solvent µL	grinding time @30Hz	**tp 3.3 mmol 598mg**	**bzm 3.3 mmol 402mg**	**Form I %M**	e.s.d. mol%	**Form II %M**	e.s.d. mol%
2*10mm ID	1g	**Neat**	**25 min**	**598.00**	**401.78**				
Sample from **location 1** from 1 g of product inside same grinding jar						91.5	*0.6*	8.5	*0.6*
Sample from **location 2** from 1 g of product inside same grinding jar						91.3	*0.5*	8.7	*0.5*
Sample from **location 3** from 1 g of product inside same grinding jar						99.5	*0.2*	0.5	*0.2*
Sample from **location 4** from 1 g of product inside same grinding jar						99.5	*0.3*	0.5	*0.3*
Sample from **location 5** from 1 g of product inside same grinding jar						99.5	*0.2*	0.5	*0.2*
**CONCLUSION:**					**Average**	**96.3**	0.4	3.7	0.4
Sample is very homogenous					**%RSD**	**4.6**		119	

4.6.2 Preparation of Form II of Theophylline: Benzamide cocrystal

Theophylline anhydrous (3.32mmol; 598.0 mg] and Benzamide (3.32mmol; 402.0 mg], were weighed with a 5 decimal figure balance and transferred accurately to one half of a homemade 14.5mL snap closure stainless steel grinding jar. The total weight of the homodimers added up to around 1g. The solid chemicals were thoroughly mixed in the grinding jar with a microspatula until homogeneity of the powder was achieved. To the half of the grinding jar containing the weighed powders, two 10 mm-internal diameter hardened hardened stainless steel ball bearings (4.0g) were added. 250µL of polar solvents (H_2O, acetonitrile, ethanol or acetone) was added to the solid before snap closing the two halves of the grinding jars. The junction was secured with insulating tape. The mixture was then milled at 30 Hz in a Retsch MM400 Shaker Mill at ambient temperature (19-22°C) for period of time in excess to 25 minutes to ensure thermodynamic control was achieved resulting in quantitative formation of **Form II** cocrystal.

Table S 7 Homogeneity of the formation of **Form II** cocrystal of 1:1 Theophylline: Benzamide by ball mill LAG

Preparation of 1:1 Theophylline:Benzamide cocrystal **Form II**									
Homogeneity of ball mill grinding sample									
**Ball mill grinding** experimental conditions				**Starting coformers** weighing (mg)		**Reaction product of ball mill LAG** Phase composition			
ss balls	Payload	solvent µL	grinding time @30Hz	**tp 3.3 mmol 598mg**	**bzm 3.3 mmol 402mg**	**Form I %M**	e.s.d. mol%	**Form II %M**	e.s.d. mol%
2*10mm ID	1g	**250µL H_2O**	**25 min**	**598.05**	**402.15**				
Sample from **location 1** from 1 g of product inside same grinding jar						1.4	0.3	98.6	0.3
Sample from **location 2** from 1 g of product inside same grinding jar						0.4	0.2	99.6	0.2
Sample from **location 3** from 1 g of product inside same grinding jar						1.7	0.3	98.3	0.3
Sample from **location 4** from 1 g of product inside same grinding jar						2.2	0.3	97.8	0.3
Sample from **location 5** from 1 g of product inside same grinding jar						0.7	0.2	99.3	0.2
**CONCLUSION:**					**Average**	1.3	0.3	**98.7**	**0.3**
Sample is very homogenous					**%RSD**	57		0.7	

4.6.3 Investigation of the thermodynamic product of the Theophylline: Benzamide cocrystals under Neat grinding or LAG with apolar solvents conditions

The best way to determine if **Form I** or **Form II** cocrystal is the thermodynamic product under Neat grinding or LAG with apolar solvents, is to ball mill neat grind a 1:1 mixture of **Form I** and **Form II** under the given experimental conditions for extended periods of time. The resulting cocrystal form is the thermodynamic product under the given conditions. In this case is **Form I**.

We investigated the given conditions at 1g payload and at 200 mg payload. The ball mill grinding was performed neat or by adding an apolar solvent , 250 µL for a 1 g payload and 50µL for a 200 mg payload.

4.6.3.1 Procedure: Ball mill neat grinding or ball mill LAG with apolar solvent of equimolar amount of cocrystals tp:ba Form I and tp:ba Form II at 1g payload

1:1 tp:ba **Form I** cocrystal (500 mg) and 1:1 tp:bzm **Form II** cocrystal (500 mg), were weighed with a 5 decimal figure balance and transferred accurately to one half of a homemade 14.5mL snap closure stainless steel grinding jar. The total weight of the homodimers added up to around 1g. The solid chemicals were thoroughly mixed in the grinding jar with a microspatula until homogeneity of the powder was achieved. To the half of the grinding jar containing the weighed powders, two 10 mm-internal diameter hardened stainless steel ball bearings (4.0g) were added. Nothing else was added to the solid before snap closing the two halves of the grinding jars. The junction was secured with insulating tape. The mixture was then milled at 30 Hz in a Retsch MM400 Shaker Mill at ambient temperature (19-22°C) for the specified period of time (15 minutes, 3x50 minutes and 24x60 minutes). PXRD analysis of the sample was performed immediately after completion of the grinding. The phase composition was determined by Rietveld refinement of the PXRD pattern. The experimental data is documented in Section 14.4.

4.6.3.2 Procedure: Ball mill neat grinding or ball mill LAG with apolar solvent of equimolar amount of cocrystals tp:bzm Form I and tp:bzm Form II at 200 mg payload

1:1 tp:bzm **Form I** cocrystal (100 mg) and 1:1 tp:bzm **Form II** cocrystal (100 mg), were weighed with a 5 decimal figure balance and transferred accurately to one half of a homemade 14.5mL snap closure stainless steel grinding jar. The total weight of the homodimers added up to around 200 mg. The solid chemicals were thoroughly mixed in the grinding jar with a microspatula until homogeneity of the powder was achieved. To the half of the grinding jar containing the weighed powders, two 7 mm-internal diameter (1.37g) hardened stainless steel ball bearings were added. Nothing else was added to the solid before snap-closing the two halves of the grinding jars. The junction was secured with insulating tape. The mixture was then milled at 30 Hz in a Retsch MM400 Shaker Mill at ambient temperature (19-22°C) for the specified period of time (15 minutes, 42.5 minutes and 3x50 minutes). PXRD of the sample was performed immediately after completion of the grinding. The phase composition was determined by Rietveld refinement of the PXRD pattern. The experimental data is documented in Section 14.4.

4.6.3.3 Procedure: Ball mill LAG with polar solvent of equimolar amount of cocrystals tp:bzm Form I and tp:bzm Form II at 1g payload

1:1 tp:bzm **Form I** cocrystal (500 mg) and 1:1 tp:bzm **Form II** cocrystal (500 mg), were weighed with a 5 decimal figure balance and transferred accurately to one half of a homemade 14.5mL snap closure stainless steel grinding jar. The total weight of the homodimers added up to around 1g. The solid chemicals were thoroughly mixed in the grinding jar with a microspatula until homogeneity of the powder was achieved. To the half of the grinding jar containing the weighed powders, two 10 mm-internal diameter hardened stainless steel ball bearings (4.0g) were added. 250μL of polar solvent (water) was added to the solid before snap closing the two halves of the grinding jars. The junction was secured with insulating tape. The mixture was then milled at 30 Hz in a Retsch MM400 Shaker Mill at ambient temperature (19-22°C) for the specified period of time (1 minutes, 3x50 minutes and 24x60 minutes). PXRD of the sample was performed immediately after completion of the grinding. The phase composition was determined by Rietveld refinement of the PXRD pattern. The experimental data is documented in Section 14.5.

4.6.3.4 Procedure: Ball mill LAG with polar solvent of equimolar amount of cocrystals tp:bzm Form I and tp:bzm Form II at 200 mg payload

1:1 tp:bzm **Form I** cocrystal (100 mg) and 1:1 tp:bzm **Form II** cocrystal (100 mg), were weighed with a 5 decimal figure balance and transferred accurately to one half of a homemade 14.5mL snap closure stainless steel grinding jar. The total weight of the homodimers added up to around 200 mg. The solid chemicals were thoroughly mixed in the grinding jar with a microspatula until homogeneity of the powder was achieved. To the half of the grinding jar containing the weighed powders, two 7 mm-internal diameter hardened stainless steel ball bearings (1.37g) were added. 50μL of polar solvent (water or MeCN) was added to the solid before snap closing the two halves of the grinding jars. The junction was secured with insulating tape. The mixture was then milled at 30 Hz in a Retsch MM400 Shaker Mill at ambient temperature (19-22°C) for the specified period of time (15 minutes and 3x50 minutes). PXRD of the sample was performed immediately after completion of the grinding. The phase composition was determined by Rietveld refinement of the PXRD pattern. The experimental data is documented in Section 14.5.

4.6.4 Procedure: Kinetic studies of polymorph interconversion from Form I to Form II by ball mill LAG with polar solvent at 1g payload

The objective of this kinetic study was to determine the time required to reach thermodynamic equilibrium. The starting material for this kinetic study: **Form I** was prepared as described in Section 4.6.1 in the same grinding jar which was later used for the particular kinetic point. The expected polymorphic form of the starting material was confirmed by PXRD and the powder from the PXRD sample slide was quantitatively returned to the grinding jar. To around 1 g of **Form I** already containing the two 10 mm ID stainless steel balls, 250 μL of water was added. The two halves of the grinding jar were snap-closed and the junction secured with insulating tape. Ball mill LAG was performed for the period of time shown in Table S142. The experimental data is documented in Section 14.6.

4.6.5 Procedure: Kinetic studies of polymorph interconversion from Form II to Form I by ball mill neat grinding at 1g payload

The objective of this kinetic study was to determine the time required to reach thermodynamic equilibrium. The starting material for this kinetic study**, Form II**, was prepared as described in Section 4.6.2 with 250 μL water, Ethanol or Acetone using the same grinding jar which was later used for the particular kinetic point. The expected polymorphic form of the starting polymorph was confirmed by PXRD to be **Form II** and the powder from the PXRD sample slide was quantitatively returned to the grinding jar. The wet powder from the grinding jar was transferred to a large box assembled from weighing paper to spread the powder and left in the fumehood overnight to ensure that the powder was properly dried before use for kinetic studies. This dry **Form II** powder was transferred back to the grinding jar used before. To around 1 g of **Form II** already containing the two 10 mm ID stainless steel balls nothing else was added for ball mill neat grinding. The two halves of the grinding jar were snap closed and the junction secured with insulating tape. Ball mill neat grinding was performed for the period of time shown in Table 143. The experimental data are documented in Section 14.7.

4.6.6 Procedure: Turnover experiments of polymorph interconversion between Form I and Form II of the 1:1 tp:bzm cocrystals at 1g payload

Starting from 1g of dried **Form II** in a 14.5mL snap closed stainless steel grinding jar to which two 10 mm ID stainless ball bearings were added, the grinding jar was snap closed and the closure secured with insulating tape. The mixture was then milled at 30 Hz in a Retsch MM400 Shaker Mill at ambient temperature (19-22°C) for 4 x 60 minutes with 10 minutes rest in between grinding periods. On completing the grinding, the grinding jar was opened and the powder was transferred to a PXRD slide to confirm that the powder has reached equilibrium giving quantitative **Form I**. The powder was then returned to the grinding jar and 250 μL of water was added. The grinding jar was snap closed and the junction secured with insulating tape. Ball mill LAG was performed for 15 minutes at 30 Hz in a Retsch MM400 Shaker Mill at ambient temperature (19-22°C). On completion, the grinding jar was opened and some of the content transferred to a PXRD slide to confirm that the powder has reached equilibrium giving quantitative **Form II**. All the content of the grinding jar was transferred to a large box assembled from weighing paper to spread out the powder and it was allowed to dry overnight. The first turnover experiment has been completed now. Using the same grinding jar, four more turnover experiments were performed. The experimental data is documented in Section 14.8.

4.7 Procedure: Solubility studies in 15 solvents of $(2NO_2PhS)_2$, $(4ClPhS)_2$ and the 2 polymorphs of heterodimer in System ii: Form A and Form B

4.7.1 Design of the protocol for solubility studies

A preliminary investigation of the solubility of **1-1**, **2-2** and **Form B** in 15 solvents was performed. This assisted in designing the proper protocol to perform a detailed solubility study of **1-1** and **2-2** and the two polymorphs of the heterodimer, **Form A** and **Form B**. Summary data for **1-1, 2-2, Form A** and **Form B** is reported in Section 10.11, 10.12 and 10.13.

The design of this solubility investigation was such that it allowed at the same time to determine which of the two polymorphs was the thermodynamic and which was the metastable polymorph in slurry experiments.

1.8 mL HPLC screw-closure clear glass vials were used to prepare the slurry solution required for the solubility studies. The small size of the HPLC vials allowed the introduction of a 2mm long magnetic flee encased in Teflon while restricting the amount of powder/crystals of **1-1**, **2-2**, **Form A** and **Form B** required for the solubility experiments. These vials were tightly closed with a Bakelite cap, the Teflon liner inside the Bakelite cap preventing evaporation of low volatile solvents. The slurries were vigorously stirred with a magnetic stirrer during the solubility experiments. Enough excess of powder or crystals of the homodimers and heterodimers were added to the glass vials so as to have a solid residue of approximately 50 mg in the slurry even after vigorous stirring. This residual solid were used to investigate if a polymorph interconversion from **Form A** to **Form B** or vice versa could take place during the solubility studies.

After many failed trials, it was found that the study of just 5 solvent of similar solubility for each of the solid/crystals investigated gave the best and most reproducible protocol. As the solubility of the homodimers **1-1** and **2-2** are not critical for the outcome of this manuscript, only summary data is tabulated in Section 10.11 to 10.13.

A specific isocratic reverse phase HPLC method was developed for **1-1** (section 4.7.2.1), **2-2** (section 4.7.2.2) and **1-2** (section 4.7.2.3). Before starting the solubility studies, a linearity curve was performed for each HPLC method. Section 10.1.1 documents the linearity graph for **Form B** which is equally applicable to **Form A**. For the solubility studies, aliquots of the supernatant of the slurry formed were diluted with an aliquot of MeCN+0.2%v/v TFA to obtain a concentration of the samples to be within the demonstrated linearity range of the HPLC method. 0.2%v/v TFA was added to the MeCN used for the sample preparation for HPLC analysis to avoid disulfides from scrambling through disulfide exchange.[3, 20] To ensure that the determination of the concentration of the diluted supernatants was as accurate as possible, two independent external standards using crystal of **1-1**, **2-2** or **Form B**, as required, were freshly weighed and prepared as the two independent reference standard solutions. The duplication between the two freshly prepared independent external standard solutions is reported for each solubility point as (Std1/Std2 duplication). This protocol uses an injection program for each solubility point, the HPLC data being saved in just one HPLC file. Results of solubility determination can be found in Section 10. The investigation of the polymorphic stability of **Form A** and **Form B** in slurry experiments were carried out as an integral part of the solubility determination and it is reported in Section 12.

4.7.2 Methodology for solubility determination by HPLC

4.7.2.1 HPLC method for solubility determination of 1-1 $(2NO_2PhS)_2$

HPLC column: 1.8μm Zorbax XDB C18, (4.6mm ID × 50 mm length)
HPLC mobile phase: A(water); B(MeCN) 95% B; 2mL/min; 50°C; 0.5μL injection; 240nm (8nm bandwidth) with reference 550nm(100nm bandwidth).

Only summary results for **1-1** will be tabulated in this document in Table S87 and Figure S 83 in Section 10.11, Figure S 84 in Section 10.12 and Figure S 85 in Section 10.13.

4.7.2.2 HPLC method for solubility determination of 2-2 $(4ClPhS)_2$

HPLC column: 1.8μm Zorbax XDB C18, (4.6mm ID × 50 mm length)
HPLC mobile phase: A (water); B (MeCN) 80% B; 2mL/min; 50°C; 0.5 μL injection; 240nm (8nm bandwidth) with reference 550nm(100nm bandwidth).

Only summary results for **2-2** will be tabulated in this document in Table S87 and Figure S 83 in Section 10.11, Figure S 84 in Section 10.12 and Figure S 85 in Section 10.13.

4.7.2.3 HPLC method for solubility determination of $(4ClPhSSPh2NO_2)_2$ polymorphs Form A and Form B

HPLC column: 1.8μm Zorbax XDB C18, (4.6mm ID × 50 mm length)
HPLC mobile phase: A (water); B (MeCN) 92% B; 2mL/min; 50°C; 0.5 μL injection; 343nm (8nm bandwidth) with reference 550nm(100nm bandwidth).

Summary results for the solubility just of **Form A** in 15 solvents are reported in Table S 85 and Figure S81 in Section 10.9.
Summary results for the solubility just of **Form B** in 15 solvents are reported in Table S 81 and Figure S80 in Section 10.5.
Summary results for the solubility of **Form A** compared just to **Form B** in 15 solvents are reported in Table S 86 and Figure S82 in Section 10.10.
Summary results for the solubility of **Form A** compared to **Form B**, and to the homodimers **1-1** and **2-2** in 15 solvents are reported in Table S87 and Figure S 83 in Section 10.11, Figure S 84 in Section 10.12 and Figure S 85 in Section 10.13.

4.7.2.4 HPLC injector programme for determination of solubility

All HPLC method used for the solubility studies used an injection program; this consist of an injection sequence of blank samples, standard reference and sample solutions run in the following consecutive injections separated by a period of 0.7 minutes. This automated sequence has 11 and 12 minutes run time;

1) MeCN+0.2%TFA blank;
2) Std 1 solution;
3) Std 2 solution;
4)MeCN+0.2%TFA blank
5) **Solvent 1 supernatant**: aliquot diluted (if necessary) in MeCN+0.2%TFA
6) **Solvent 2 supernatant**: aliquot diluted (if necessary) in MeCN+0.2%TFA
7) **Solvent 3 supernatant**: aliquot diluted (if necessary) in MeCN+0.2%TFA
8) **Solvent 4 supernatant**: aliquot diluted (if necessary) in MeCN+0.2%TFA
9) **Solvent 5 supernatant**: aliquot diluted (if necessary) in MeCN+0.2%TFA
10) MeCN+0.2%TFA blank;
11) Std 1 solution;
12) Std 2 solution;
13) MeCN+0.2%TFA blank

4.7.3 Procedure for the solubility determination of Form A and Form B

Enough crystalline material of **Form B** and of **Form A** was added to the 1.8mL glass vials for the solubility experiments.

The experiments were divided in 3 groups based on similar solubility from the preliminary study.

Table S 8 Experimental design for the determination of the solubility for **Form B** and **Form A** in 15 solvents

Group 1		Group 2		Group 3	
low solubility		medium solubility		high solubility	
1	**Water** Note 1	6	**MeCN**	11	**Benzene**
2	**IPA**	7	**DMSO**	12	**DCM**
3	**MeOH**	8	**Acetone**	13	**DMF**
4	**EtOH**	9	**EtOAc**	14	**$CHCl_3$**
5	**Cyclohexane** Note 1	10	**Toluene**	15	**THF**

Note 1: supernatant from slurries in H_2O and c-hexane are filtered through Pasteur pipette with cotton wool plug

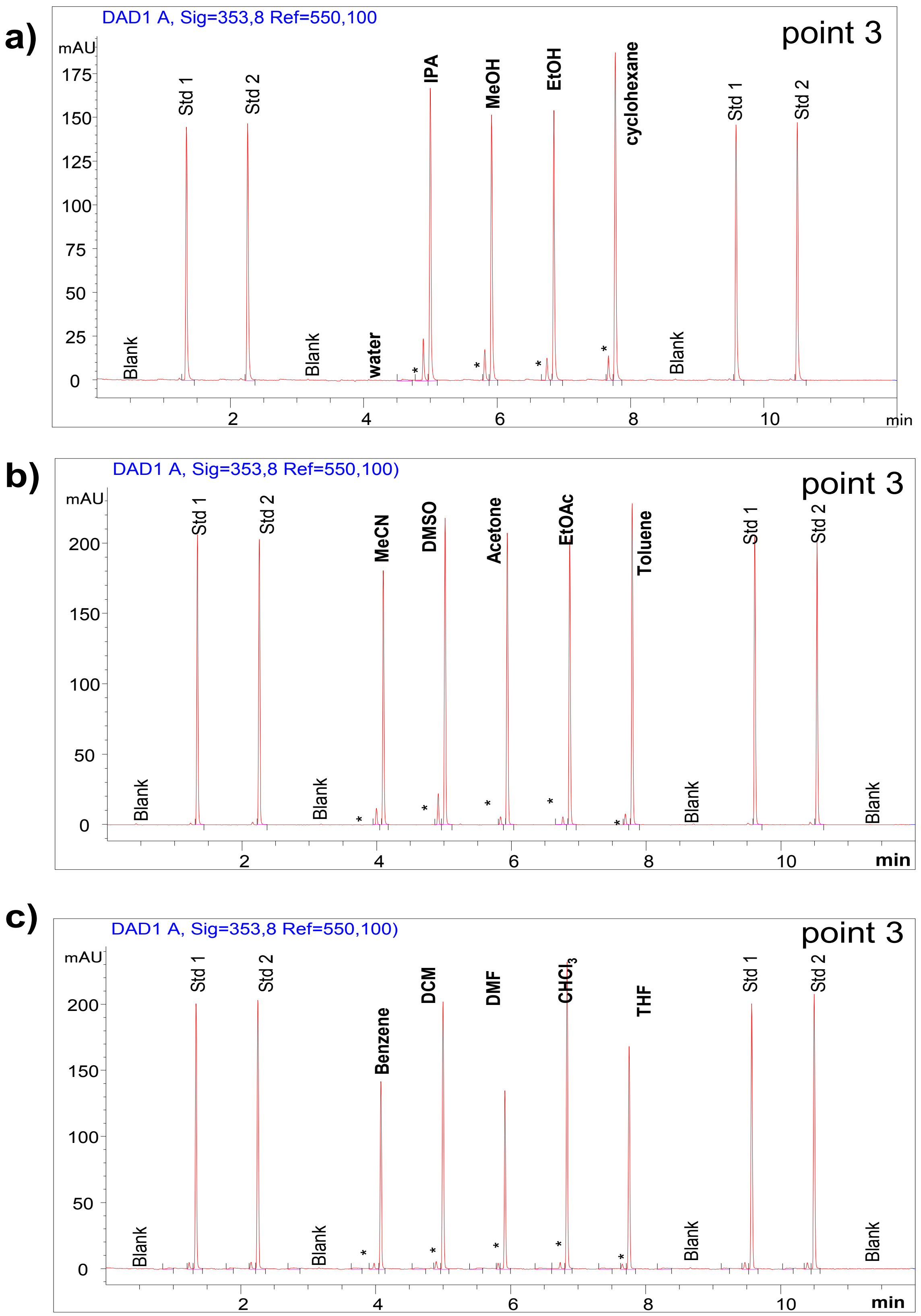

Figure S 20: examples of injections sequences for the determination of solubility of **Form B** in 3 groups of 5 solvents in, : a) poor solubility solvents: water, IPA, Methanol, Ethanol and cyclohexane; b) medium solubility solvents: MeCN, DMSO, acetone, EtOAc and Toluene and c) good solubility solvents: Benzene, DCM, DMF, chloroform and THF. The injection sequence is started and ends with the diluent used for sample preparation used as Blank injection. Std 1 and Std 2 solution of **Form B** has been prepared from independent weighings. An aliquot of the supernatant of the solubility experiments have been dissolved with the diluent to reach a similar response on HPLC.

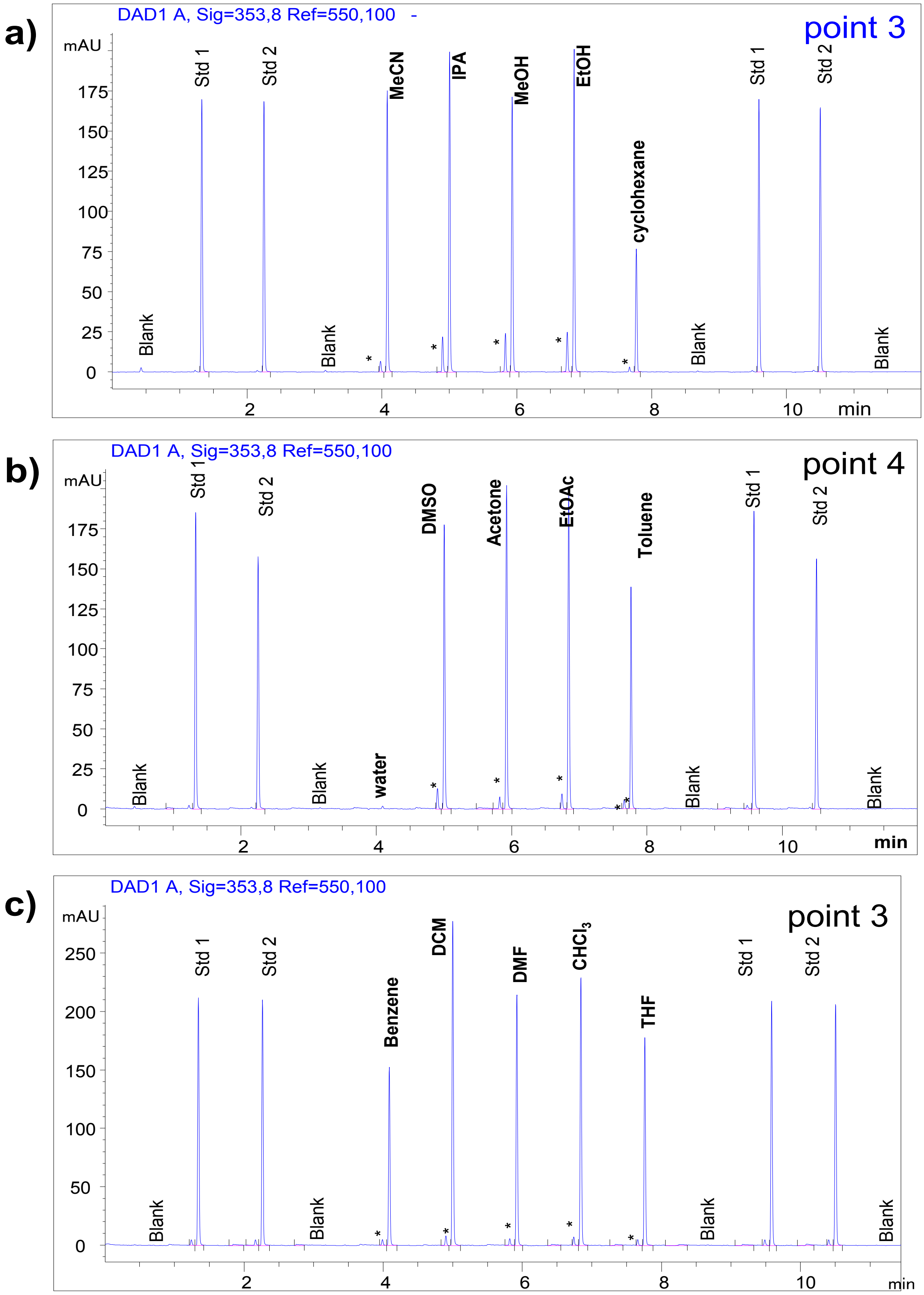

Figure S 21 examples of injections sequences for the determination of solubility of **Form A** in 3 groups of 5 solvents in, : a) poor solubility solvents: MeCN, IPA, Methanol, Ethanol and cyclohexane; b) medium solubility solvents: water (by mistake), DMSO, acetone, EtOAc and Toluene and c) good solubility solvents: Benzene, DCM, DMF, chloroform and THF. The injection sequence is started and ends with the diluent used for sample preparation used as Blank injection. Std 1 and Std 2 solutions of **Form A** have been prepared from independent weightings. An aliquot of the supernatant of the solubility experiments have been dissolved with the diluent to reach a similar response on HPLC.

5 Analysis of the solid state samples

5.1 Analysis by HPLC

The chemical composition of the DCC experiments performed by grinding, were analysed by reverse phase HPLC using an Agilent 1200 Series composed of a HPLC high pressure binary pump, autosampler, column oven and a Diode Array Detector. 1.8μm Zorbax XDB C18, (4.6mm ID × 50 mm length) was used as the HPLC column. The conditions of the HPLC method are as follows:

Solvent A: Water +0.1% Formic acid;

Solvent B: Acetonitrile +0.1% Formic acid;

Gradient of 0-2 minutes 75% - 85%B with re-equilibration time of 1 minutes.

Flowrate: 2 ml/min; Column temperature of 60°C;

Injection volume of 1 μL.

The signal was monitored at 259 nm (8 nm bandwidth) with reference at 550 nm (100 nm bandwidth)

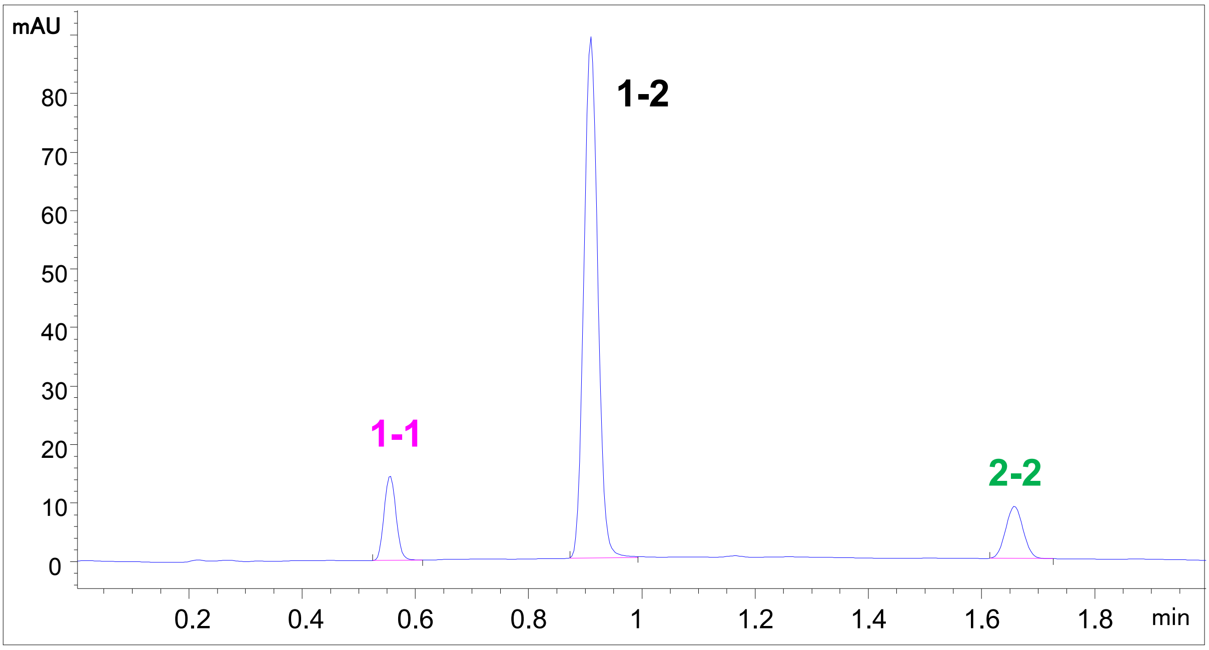

Figure S 22 Typical HPLC chromatogram of enriched heterodimer **1-2** (made up of **Form A** + **Form B**). HPLC run contains the two homodimers, **1-1** and **2-2**.

5.1.1 Preparation of HPLC samples from solid state DCC studies

HPLC samples were freshly prepared at a concentration of 1.0 mg/ml in acetonitrile containing 0.2%v/v of trifluoroacetic acid (TFA). The acid was added to quench the base catalysed reaction (see Section 4.1.2). In this way, preventing the disulfide exchange reaction from taking place in solution which would have resulted in the scrambling of the dynamic covalent chemistry library forming a statistical mixture of **1-1**, **2-2** and **1-2**, in a 1:1:2 proportion before HPLC analysis. The HPLC samples were then sonicated for a few minutes to bring them fully into solution before injecting them into the HPLC.

5.2 Analysis by PXRD

X-ray powder diffractograms in the 2θ range 4-45° (Cu Kα radiation, step size 0.03°, time/step 100 s, 0.04 rad soller, VxA 40x40) were collected either (1) on an X-Pert PRO MPD powder X-ray diffractometer or (2) on a Panalytical X'Pert Pro diffractometer both equipped with an X'Celerator detector. Both PXRD equipment were available at the Department of Chemistry, University of Cambridge.

5.2.1 Sample preparation for PXRD analysis

On completion of the grinding experiment, the grinding jar was immediately opened and the powder transferred to an agar mortar. The powder was gently ground with an agar pestle, and the powder transferred to the sample holder for Powder X-Ray diffractometry. The PXRD of the starting materials **1-1** and **2-2,** unreacted **1-1**+**2-2** and the two polymorph **Form A** and **Form B** are shown in Figure S23.

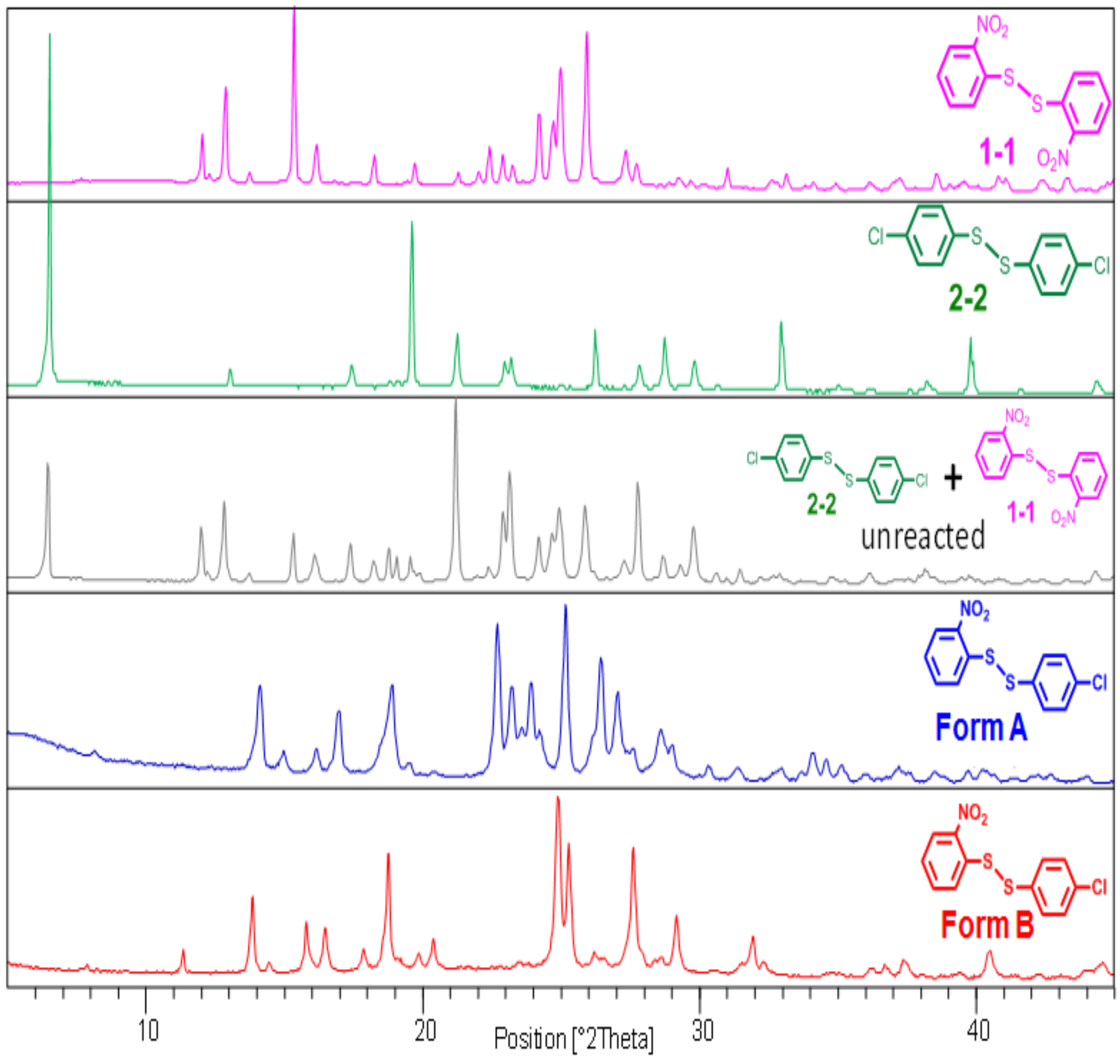

Figure S 23 PXRD scan of the starting materials **1-1** and **2-2** and the two polymorph **Form A** and **Form B** using a X-Pert PRO MPD powder X-ray diffractometer, using Cu Kα radiation (0.154184 nm) at 40 kV, 40 mA with a scanning rate of 10°/min, and a 2θ angle ranging from 4 to 45°

5.3 Rietveld quantitative analysis of solid state samples

The solid state composition of the samples was determined by Rietveld refinements from PXRD data. X-ray powder diffractograms in the 2θ range 4-45° (Cu Kα radiation, step size 0.03°, time/step 100 s, 0.04 rad soller, VxA 40x40) were collected either (1) on an X-Pert PRO MPD powder X-ray diffractometer or (2) on a Panalytical X'Pert Pro diffractometer both equipped with an X'Celerator detector. Both PXRD equipment were available at the Department of Chemistry, University of Cambridge. Rietveld refinements were performed with the software Topas V4.1.[1] For each of the six compounds the crystal structure retrieved from the Cambridge Structural Database[1] or directly from the papers by Fischer et al. [34,35] was refined on a dataset collected on a pure single phase specimen: this first step was useful for the identification of the crystallographic planes most affected by preferred orientation phenomena. These were (0 1 0) for **1-1**; (0 0 1) for **2-2**; (1 0 2) and (0 0 1) for **(1-2)A**; (0 1 0) for **(1-2)B**. The March-Dollase model for preferred orientation was applied on these crystal planes in the quantitative analysis. No correction for preferred orientation was necessary for the system (**i**) instead. No structural parameter was refined in the quantitative Rietveld refinements. The amorphous fractions was assumed to be neglectable for both systems. For system (**ii**), the **1-1** and **2-2** scale factors were constrained to be refined together (in order to have a **1-1**/**2-2** molar ratio of 1). This was found to be the most effective approach, after several trials and comparisons with the HPLC data: even if these assumptions may sound rather strong (the **1-1** amorphous fraction may be in principle different from the **2-2** amorphous fraction, leading to a ratio of the crystalline fractions different from 1), the excellent agreement with the HPLC data validates our approach.[33] Furthermore the peak shape and the parameters describing the diffractometer geometry were optimized using a LaB6 standard: only a Lorentzian Scherrer term (CS_L) for each phase was modeled in the Pseudo-Voigt functions for the quantitative analysis, the other parameters being fixed. A minimum limit of 30 nm for the crystal size was define to avoid correlations with the background. On the basis of tests performed on six replicates made on the same sample, we estimate an accuracy error of ±6%M absolute.[33] A shifted Chebyshev function with seven parameters was used to fit the background. R_{wp} and χ^2 values ranged typically from 6% to 12% and from 3 to 6 respectively.

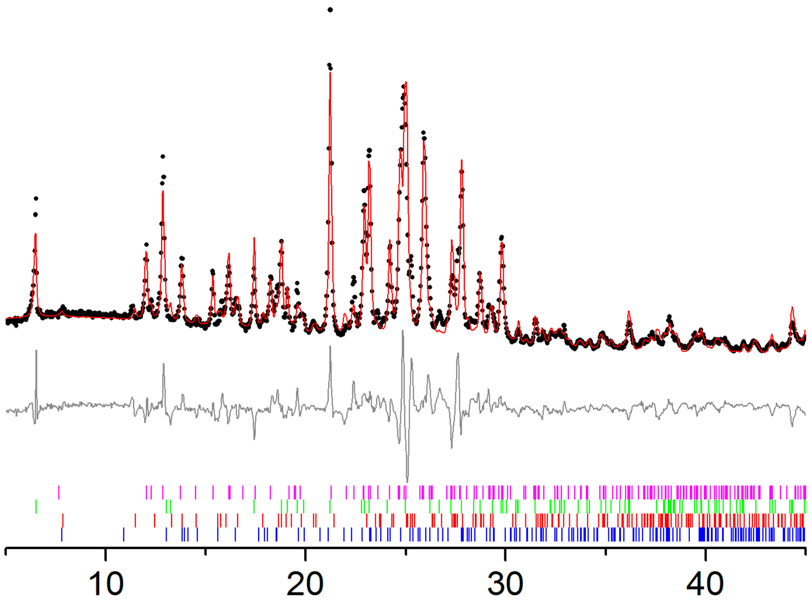

Figure S24a Plot of observed, calculated and difference curves from one of the refinements as an example for system **(ii)**.

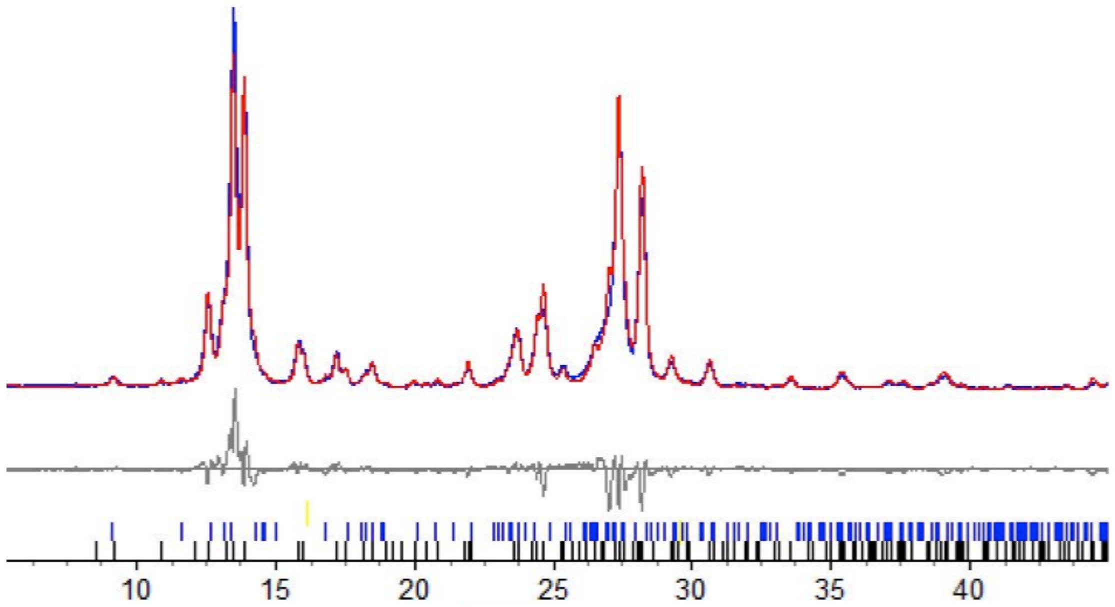

Figure S24b Plot of observed, calculated and difference curves from one of the refinements as an example for system **(i)**.

Reference:

[1] Coelho, A. (2007) TOPAS-Academic, Coelho Software, Brisbane, Australia

[2] F. H. Allen, Acta Crystallogr., Sect. B: Struct. Sci., 2002, 58, 380–388.

[3] A. M. Belenguer, G. I. Lampronti, D. J. Wales and J. K. M. Sanders, *Journal of the American Chemical Society*, 2014, 136, 16156-16166.

5.4 Quantification of amorphous phase using Rietveld refinement

The Rietveld method allows very accurate quantitative analysis of crystalline phases in a polycrystalline powder, with errors minor than 1%. Indeed, the more similar are the physical-chemical properties of the phases (X-ray microabsorption), the more accurate is the analysis. The relative weight quantity of a phase *i* is given by:

$$W_i = [S_i \cdot (M_i \cdot Z_i \cdot V_i)] / [\Sigma_j S_j \cdot (M_j \cdot Z_j \cdot V_j)]$$

where M_i is the asymmetric unit weight (the molecular weight in many cases) of phase *i*, Z_i is the multiplicity of the space group, and V_i is the cell volume, and $[\Sigma_j S_j \cdot (M_j \cdot Z_j \cdot V_j)] = 1$. The Rietveld method is generally more accurate than quantitative methods that are based on single peaks area or intensity, because the analysis takes into account the whole diffraction pattern minimizing any error due to local problems (*e.g.* preferred orientation and peak shape).

When an amorphous phase is present in the crystalline powder, quantification is not a straightforward analytical process, because Rietveld refinement is "blind" for amorphous phases: the scattering due to non-crystalline phases is added to the background scattering, *i.e.* the instrumental background. In these cases an "internal standard is used: a known quantity of a known phase is added to the crystalline powder. If an amorphous phase is present in the sample all the crystalline phases will be overestimated because $S_j \cdot (M_j \cdot Z_j \cdot V_j)] = 1$, and background is not considered. Real amorphous fraction in the sample can be calculated directly from:

$$W_i = [1 / (1 - W_s)] \cdot [1 - (W_s / W_{s,c})] \quad ,$$

where W_s is the experimental weight fraction of internal standard, while $W_{s,c}$ is the calculated weight fraction of internal standard from the refinement. Rilevability limit for amorphous phase is generally considered 10%.

The main problem for a quantification method with powder diffraction is choosing the internal standard, since it has to have the following features:

- it must have a microabsorption value as near to that of the sample as possible;
- it must have a good to optimum crystallinity;
- It must be inert in the analyzed substance (also in grinding);
- It is better if its main diffraction peaks don't overlap on those of the sample;
- It is better if it doesn't present strong preferred orientation.

In the case of system **(ii)**, corundum, α-Al_2O_3, was chosen as internal standard.

Diffractograms in the 2θ range 5-60° (Cu Kα radiation, step size 0.02°, time/step 100 s, 0.04 rad soller, VxA 40x40) were collected in Bragg-Brentano geometry on an X-Pert PRO MPD powder X-ray diffractometer equipped with an X'Celerator detector, available at the Department of Chemistry, University of Cambridge.

Sample **A1** was prepared by method (**a**) under LAG conditions with 21 μL of $CHCl_3$. Two specimens from the milling jar were weighted straight after the milling experiment was over. They were manually mixed with 20.6% (sample **A3**) and 35.9% (**sample-A2**) wt of internal standard and measured immediately by PXRD.

Topas V4.1 was utilized for Rietveld refinements.[27] Fundamental parameters[28] were determined with the NIST 660b LaB_6 standard, and then fixed. Refinement details are here listed: seven parameters were used for background; the homodimers (**1-1** and **2-2**) were found to be lower than 1.5 wt% and thus were excluded from the refinement; March-Dollase model was used to correct for preferred orientation on the crystallographic plane (0 1 0) of **Form A** and (1 0 2) and (0 0 1) of **Form B**; for **Form A** and Form **B**

crystal size contribution to peak broadening was assumed to be isotropic and Laurenzian, and microstrain contribution was supposed to be neglectable; for corundum microstrain contribution to peak broadening was assumed to be isotropic and gaussian, and crystal size contribution was supposed to be neglectable; structural parameters were all fixed except for cell parameters.

Table S 9: reports R_{wp} values, experimental weight fraction of internal standard, calculated weight fraction of internal standard from the refinement itself, estimated weight fraction of amorphous phase in the sample.

	R_{wp}	calc %wt Form A	calc %wt Form B	exp %wt Al_2O_3	calc %wt Al_2O_3	sample %wt amorphous	Figure
**Sample-A3**	8.45%	24.1(3)	51.7(4)	20.6%	24.2(3)%	1 9%	S 25
**Sample A2**	7.68%	16.2(3)	19.6(4)	35.9%	44.2(3)%	30%	S 26

The difference in amorphous quantification between sample **A2** and **A3** is relatively small considering that the accuracy in amorphous determination can be as large as 10% absolute.[29] The refinements show that a fraction of **A1** larger than 10 wt% is most likely amorphous.

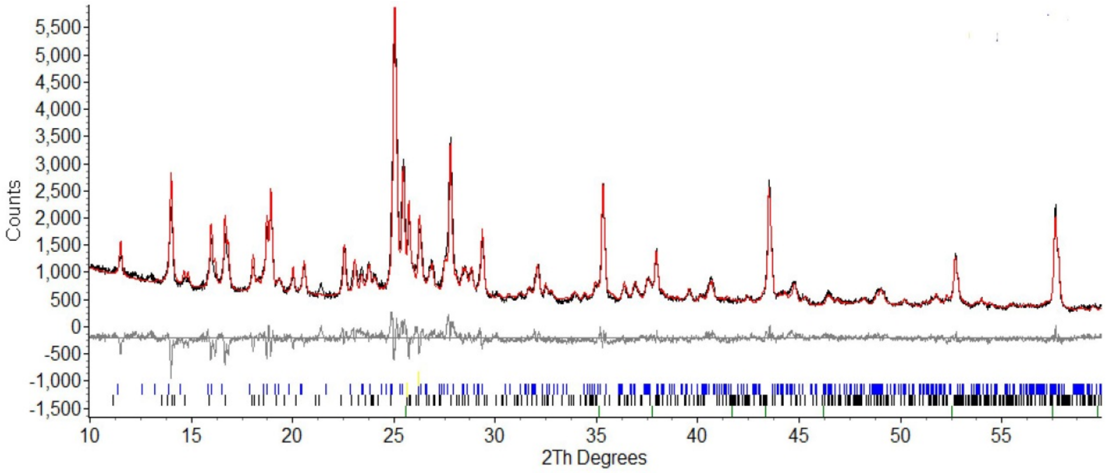

Figure S 24 . Experimental (black curve), calculated (red curve) and difference (grey curve) patterns of measurement sample-A3. **Form B** (blue), **Form A** (black) and corundum (green) peak marks are also indicated.

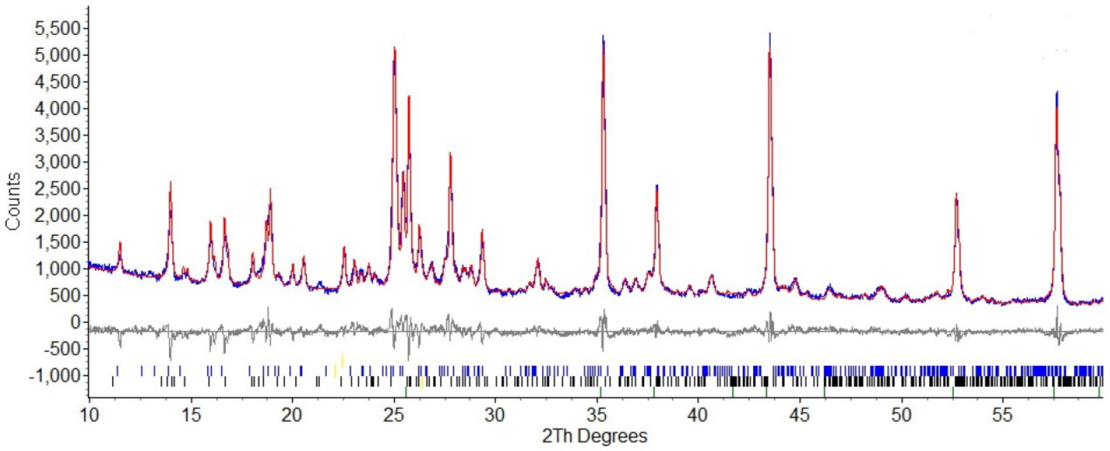

Figure S 25 *Experimental (blue curve), calculated (red curve) and difference (grey curve) patterns of measurement sample-A2.* ***Form B*** *(blue),* ***Form A*** *(black) and corundum (green) peak marks are also indicated.*

5.5 Crystal size determination from powder diffraction data

The crystal size was estimated by whole pattern Rietveld refinements which incorporate the Scherrer equation,

$$\text{crystal size [in nm]} = k\lambda/(10 \cdot \Delta\theta \cdot \cos\theta),$$

where k is a crystal shape factor most often assumed to be 0.9, λ is the radiation wavelength and θ is the diffraction angle, into the peak shape function in a given crystallographic direction (hkl) according to the following equation:

$$\text{fwhm}(2\theta, \text{hkl}) = (180/\pi)\ \lambda\ /\ (\cos\theta \cdot \text{crystal size}),$$

where fwhm(2θ) is the full width at half maximum of the peak at a given diffraction angle and a given crystallographic direction.[30]
The LaB_6 660b NIST standard[31] was used to model the instrumental contribution to peak broadening using a fundamental parameters approach with the software Topas v4.1.[32] Because of the nanocrystalline nature of the analysed powders and the absence of peaks above 45º in 2θ, the sample contribution to the peak broadening was assumed to be related to size only. A Lorentzian peak shape was found to fit better than a Gaussian one. In order to minimise the correlations and the e.s.d., the crystal size contribution to peak broadening was modelled as isotropic, i.e. using one single parameter for all crystallographic directions. We here remind that the e.s.d. from the Rietveld calculation has no bearing on the precision or accuracy, being merely related to the mathematical fit of the model. For what concerns the accuracy of the size determination, it is known that for a typical laboratory X-ray diffraction instrument the Scherrer analysis provides sensitivity to crystallite size in the 1–100 nm range, the upper limit being set by the instrumental broadening. This also means that the smaller the crystal size, the less the Scherrer size value is affected by how the instrumental broadening is defined.[30] In our experience related to the present case, a Scherrer crystal size of 100nm can vary by up to 30% relative of its value depending on the way the fundamental parameters are used to fit the LaB_6 660b NIST standard - e.g., depending on whether the size and microstrain contribution for the NIST standard are assumed to be zero or are allowed to give a contribution to the LaB_6 peak shape. However, if the crystal size is 60nm, this variation is no more than 10% relative. In other words, the smaller the crystal size, the more reliable the number. It is also important to point out that the peak shape tends to be dominated by the larger crystallites rather than the smaller ones, so the calculated size tends to be overestimated.[30]

6 Polymorph interconversion Form B and Form A in solid state dynamic covalent chemistry by ball mill grinding using base catalyst

6.1 Kinetic studies for polymorph transformation from Form B to Form A in solid state DCC under ball mill neat grinding conditions (contains dbu).

Table S 10 Ball mill neat grinding of quantitative **Form B** with dbu (HPLC analysis)
Chemical composition by HPLC analysis of the reaction mixture (%M) versus grinding time at 30 Hz. Each point in the graph is an individual experiment. This table includes the HPLC method used.

Ball mill **Neat grinding**: starting from **Form B** (97%M)

(Neat, 2%M dbu)

**Form B**	**Form B** is thermodynamic product	**HPLC conditions**
MW 296.97	obtained from ball mill LAG grinding	Zorbax SB C18, 1.8 μm; 4.6mm ID x30 mm
97%M	contains dbu as base catalyst and	A: H_2O+0.1% HCOOH; B: MeCN+0.1%HCOOH
0.38 mmol	around 1.5%M of 1-1 and 1.5%M of 2-2.	0-2 min 75-85%B; 2ml/min; 60°C; 259 nm (8nm)
200 mg		

**Form B** used as starting material			grinding @ 30 Hz time (min)	REACTION PRODUCT COMPOSITION		
Product weighed (mg)	(Purity by HPLC) 1-1: **1-2** : 2-2 1-2 = $2NO_2PhSSPh4Cl$	PXRD consistent with		$(2NO_2PhS)_2$ (1-1) %M	$2NO_2PhSSPh4Cl$ 1-2 %M	$(4ClPhS)_2$ (2-2) %M
Typical composition of **Form B**			**0**	1.5	**97.0**	1.5
197.65mg	1.1-98.2-0.6	**Form B**	**5**	2.9	**94.6**	2.5
199.80mg	1.9-96.8-1.4	**Form B**	**7**	3.5	**93.2**	3.3
188.83mg	1.9-96.7-1.3	**Form B**	**10**	2.2	**95.6**	2.3
197.85mg	1.5-97.0-1.5	**Form B**	**15**	1.6	**96.9**	1.5
199.67mg	1.5-97.0-1.5	**Form B**	**20**	2.5	**95.5**	2.0
199.89mg	1.9-98.1-0.9	**Form B**	**25**	2.1	**95.4**	2.5
200.37mg	2.6-96.0-1.4	**Form B**	**30**	1.8	**96.1**	2.5
199.67mg	5.4-91.4-0.6	**Form B**	**45**	2.1	**95.4**	2.5

Table S 11 Ball mill neat grinding of quantitative **Form B** with no dbu (PXRD analysis)
Tabulation of the Rietveld refinement of PXRD data calculated as %M of the phase composition of the mixture of homodimers **1-1** and **2-2** and the two polymorphs of the product **1-2**, together with its corresponding refinement error (esd) calculated as %mol, versus grinding time for each single point experiment.

Ball mill **NG**: 97%M **form B** contains 3%M 1:1 of 1-1 to 2-2

(2%M dbu)

grind time (min)	**1-1**		**2-2**		**Form B**		**Form A**	
	**%M**	e.s.d. (mol%)	**%M**	e.s.d. (mol%)	**%M**	e.s.d. (mol%)	**%M**	e.s.d. (mol%)
**0**	**1.5**		**1.5**		**97**		**0**	
**5**	**1.9**	0.8	**1.9**	0.8	**91.7**	1.3	**4.5**	0.8
**7**	3.2	0.7	3.3	0.8	**80.5**	1.4	**13.0**	1.1
**10**	1.5	0.8	1.6	0.9	**21.2**	1.2	**75.7**	1.4
**15**	2.5	0.9	2.6	0.9	**12.1**	1.3	**82.8**	1.6
**20**	1.6	0.5	1.7	0.6	**6.3**	1.3	**90.4**	1.4
**25**	1.3	0.4	1.2	0.5	**5.3**	1.2	**92.2**	1.3
**30**	2.1	0.6	2.1	0.6	**2.1**	0.4	**93.7**	0.9
**45**	0.9	0.4	0.7	0.3	**3.1**	0.4	**95.3**	0.6

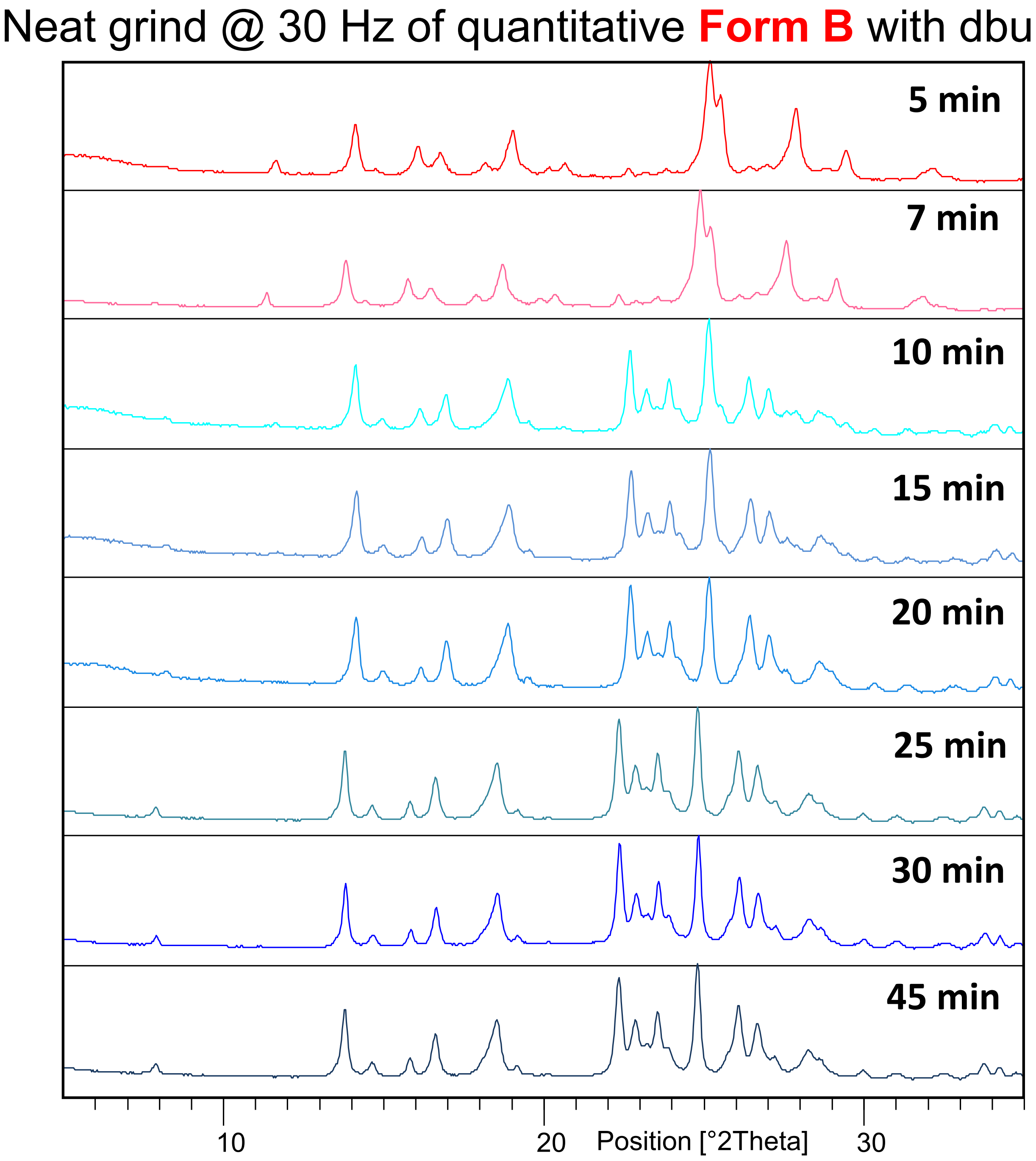

PXRD scan 1 Ball mill Neat grinding of Form B **(with dbu).** Each PXRD scan was obtained from a single point experiment at the specified time. The PXRD sample was prepared immediately after completion of the grinding experiment, and scanned between 4 and 45° (2θ).

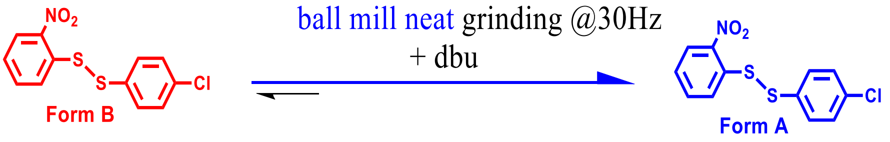

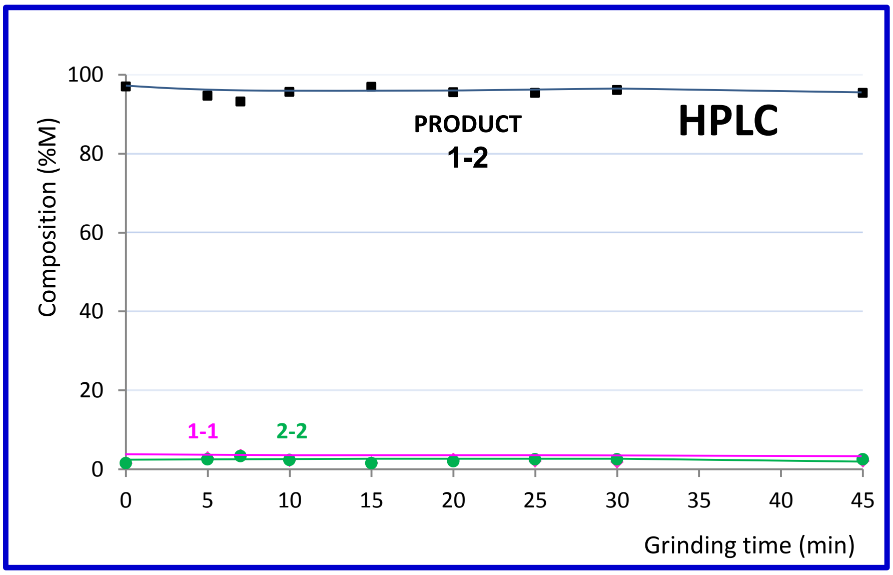

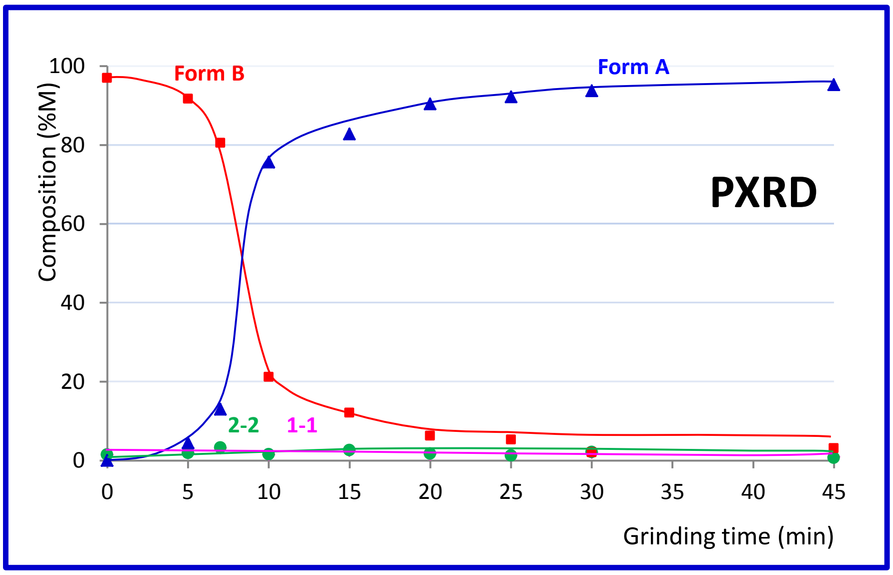

Figure S 26 Chemical and phase composition graph of ball mill neat grinding of quantitative **Form B (with dbu)**

6.2 Kinetic studies for polymorph transformation from Form A to Form B in solid state DCC under ball mill LAG conditions (contains dbu).

Table S 12 Ball mill LAG of quantitative **Form A** with dbu (HPLC analysis)
Chemical composition by HPLC analysis of the reaction mixture (%M) versus grinding time at 30 Hz. Each point in the graph is an individual experiment. This table includes the HPLC method used.

Ball mill **LAG** : starting from **Form A** (97%M)		
(LAG 50 µL MeCN, 2dbu)		
**Form A**	**Form A** is thermodynamic product	**HPLC conditions**
MW 296.97	obtained from ball mill LAG grinding	Zorbax SB C18, 1.8 µm; 4.6mm ID x30 mm
97%M	contains dbu as base catalyst .	A: H_2O+0.1% HCOOH; B: MeCN+0.1%HCOOH
0.38 mmol	and 1.5%M of **1-1** & 1.5%M of **2-2**.	0-2 min 75-85%B; 2ml/min; 60°C; 259 nm (8nm)
200 mg		

"in-situ" **Form A** as starting material			grinding @ 30 Hz time (min)	REACTION PRODUCT COMPOSITION		
In-situ product from solid-state DCC (mg)	(Purity by HPLC) 1-1: **1-2** : 2-2 1-2 = $2NO_2PhSSPh4Cl$	PXRD consistent with		$(2NO_2PhS)_2$ (1-1) %M	$2NO_2PhSSPh4Cl$ **1-2** %M	$(4ClPhS)_2$ (2-2) %M
~200 mg	used as t=0	**Form A**	**0**	1.2	**97.6**	1.2
~200 mg	used as t=0	**Form A**	**0**	1.4	**97.1**	1.5
~200 mg	used as t=0	**Form A**	**0**	1.3	**97.3**	1.4
in-situ 200mg	1.2-97.6-1.2	**Form A**	**10**	2.9	**93.7**	3.3
in-situ 200mg	no HPLC run	**Form A**	**15**	2.5	**94.4**	2.5
in-situ 200mg	1.4-97.1-1.5	**Form A**	**20**	4.3	**92.5**	3.2
in-situ 200mg	1.3-97.3-1.4	**Form A**	**25**	2.7	**93.7**	3.6
in-situ 200mg	no HPLC run	**Form A**	**45**	1.1	**97.8**	1.1
200.08	1.0-98.1-0.8	**Form A**	**60**	1.3	**97.6**	1.1
199.49	1.0-98.1-0.9	**Form A**	**70**	1.6	**96.6**	1.7
200.61	1.9-96.8-1.4	**Form A**	**80**	1.2	**97.8**	1.0
in-situ 200mg	no HPLC run	**Form A**	**90**	1.3	**97.8**	1.0
199.65	1.0-09.1-0.9	**Form A**	**105**	1.6	**97.2**	1.2
in-situ 200mg	1.7-96.5-1.8	**Form A**	**120**	1.4	**97.4**	1.2
in-situ 200mg	1.9-96.3-1.9	**Form A**	**150**	1.6	**96.9**	1.5

Table S 13 Tabulation of the Rietveld refinement of PXRD data calculated as %M of the phase composition of the mixture of homodimers 1-1 and 2-2 and the two polymorphs of the product 1-2, together with its corresponding refinement error (esd) calculated as %mol, versus grinding time for each single point experiment.

Ball mill LAG grinding: 97%M Form A (contains 3%M 1:1 of 1-1 to 2-2) (2%M dbu)								
grind time (min)	1-1		2-2		Form B		Form A	
	%M	e.s.d. (mol%)	%M	e.s.d. (mol%)	%M	e.s.d. (mol%)	%M	e.s.d. (mol%)
**0**	**1.5**		**1.5**		**97**		**0**	
**0**	**1.5**	0.3	**1.5**	0.3	**5.5**	0.7	**91.5**	0.8
**10**	**1.0**	0.3	**1.0**	0.2	**1.6**	0.5	**96.4**	0.6
**15**	**0.9**	0.3	**0.9**	0.2	**1.4**	0.3	**96.8**	0.5
**25**	**1.0**	0.3	**1.0**	0.2	**1.3**	0.3	**96.7**	0.5
**45**	**0.9**	0.3	**0.9**	0.2	**1.7**	0.4	**96.5**	0.6
**60**	**0.8**	0.4	**0.8**	0.3	**97.7**	0.6	**0.7**	0.3
**70**	**0.4**	0.2	**0.3**	0.2	**99.0**	0.3	**0.3**	0.1
**80**	**0.8**	0.4	**0.8**	0.5	**97.4**	0.7	**1.0**	0.4
**105**	**0.4**	0.3	**0.5**	0.2	**98.6**	0.4	**0.5**	0.2
**120**	**0.7**	0.3	**0.7**	0.3	**97.9**	0.5	**0.7**	0.2
**150**	**0.7**	0.3	**0.8**	0.3	**97.7**	0.5	**0.8**	0.2

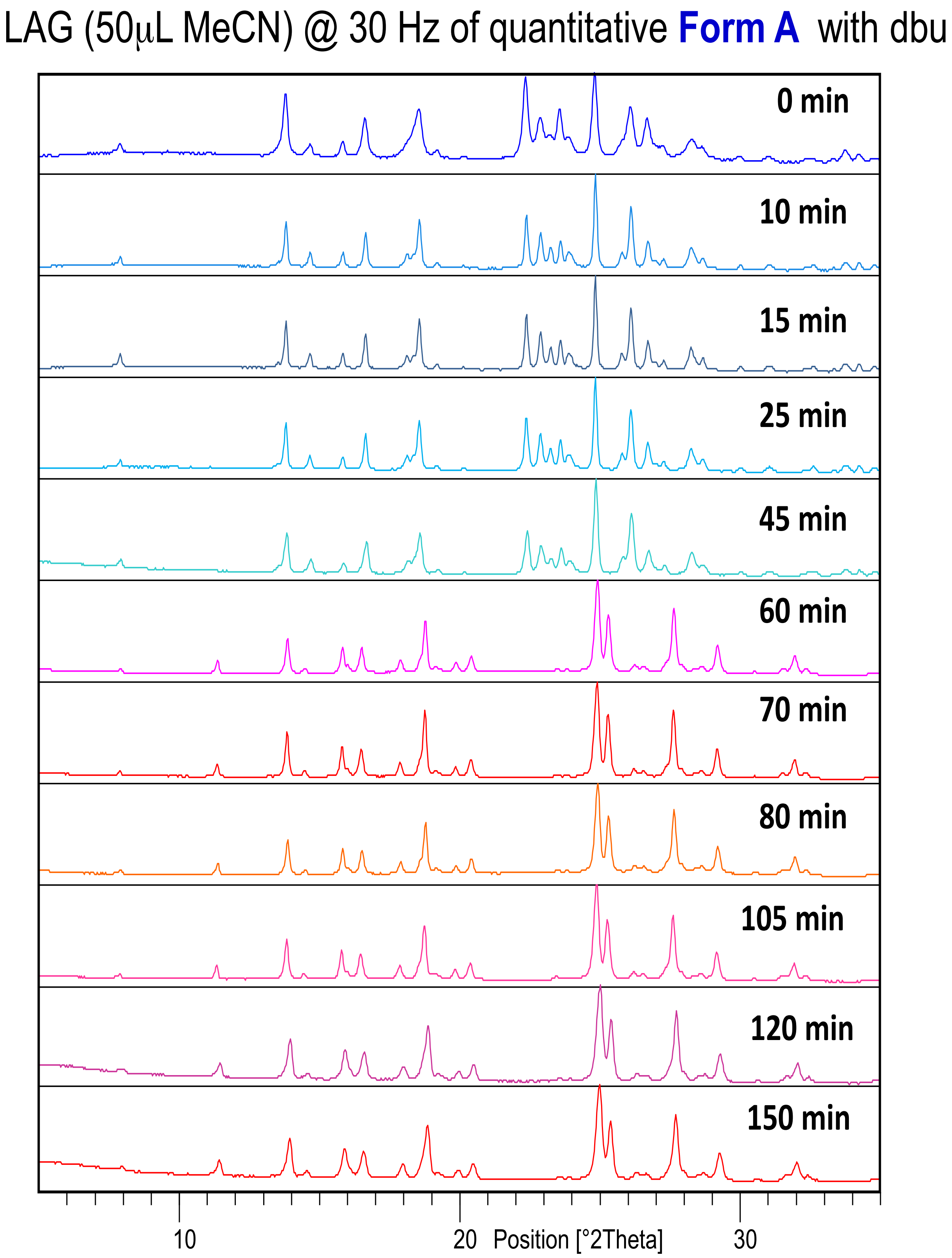

PXRD scan 2 Ball mill LAG of **Form A (with dbu);** Each PXRD scan was obtained from a single point experiment at the specified time. The PXRD sample was prepared immediately after completion of the grinding experiment, and scanned between 4 and 45° (2θ).

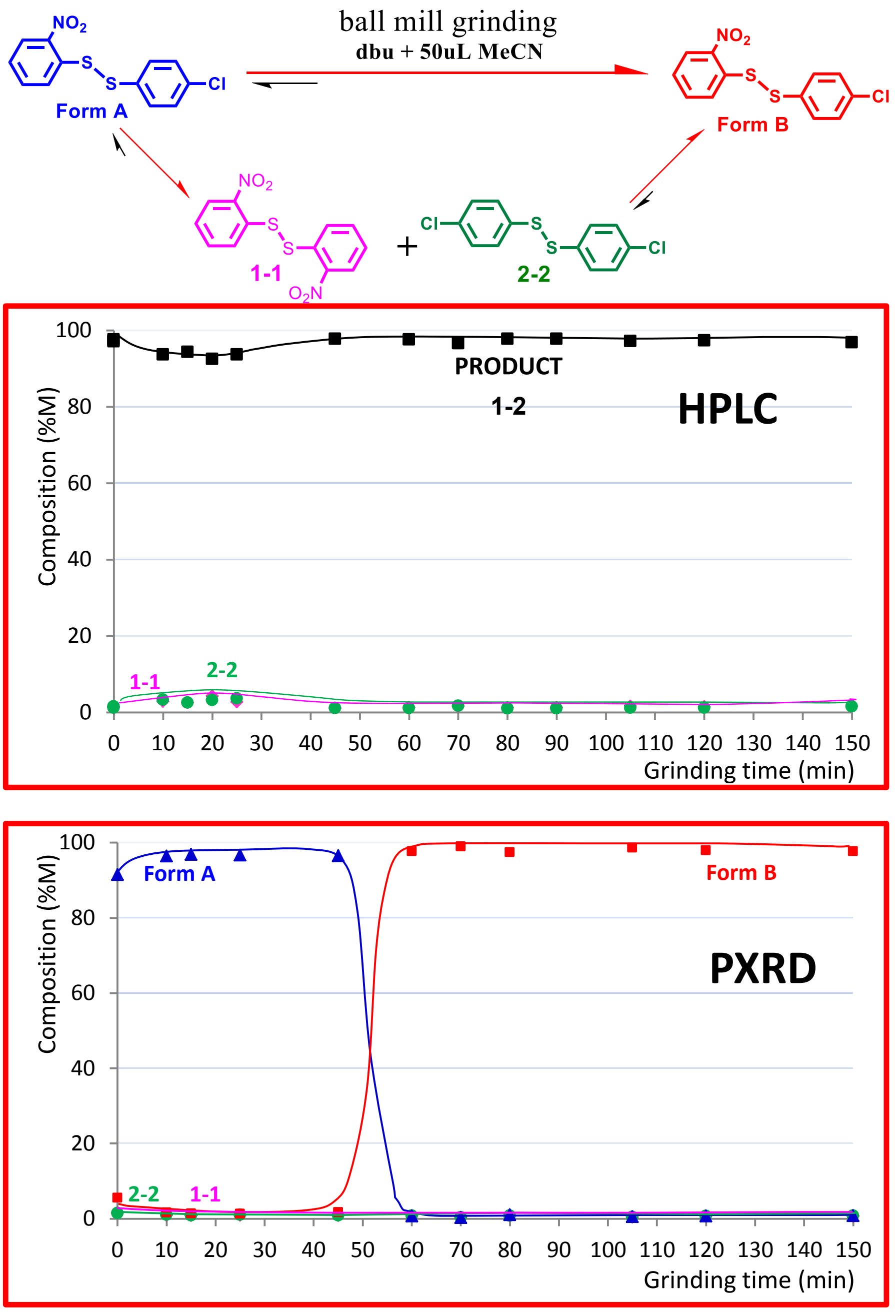

Figure S 27 Chemical and phase composition graph of ball mill LAG of quantitative **Form A** **(with dbu)**

6.3 Turnover experiments for polymorph interconversion between Form A and Form B in solid state DCC by ball mill grinding.

6.3.1 Example 1 (5 turnover experiments performed)

Table S 14 Turnover experiments of in situ polymorph interconversion between **Form B** and **Form A** in the presence of dbu, promoting dynamic covalent chemistry. The starting point is the formation of **Form B** by LAG (50uL MeCN) from equimolar amounts of the heterodimers **1-1** and **2-2** under base catalysed conditions (2%M dbu). In situ refers here to the sample being left in the grinding jar throughout the experiment. For neat grinding, the grinding jar containing **Form B** was left open overnight in fumehood to remove any traces of solvent.

Turnover polymorph interconversion between **Form A** & **Form B** in presence of dbu

Turnover experiments	grinding time @ 30Hz to equilibrium.	grinding conditions	**1-1**		**2-2**		**Form B**		**Form A**		[A]+[B]	R = [B] / [A]+[B]
		dbu present	%M	e.s.d. mol%	%M	e.s.d. mol%	%M	e.s.d. mol%	%M	e.s.d. mol%	total polymorph	% R
Starting material		2uL	2	0.8	1.8	0.7	**96**	1.1	0.7	0.3	96.2	**99.3**
Turnover-1	45 min	NEAT	2	0.6	2	0.5	4.2	0.6	**92**	0.9	95.7	**4.4**
Turnover-1	90 min	50 ul MeCN	4	0.7	3.7	0.6	**91**	0.9	1.1	0.4	92.3	**98.8**
Turnover-2	45 min	NEAT	3	0.7	2.9	0.6	4.1	0.6	**90**	1.1	93.8	**4.4**
Turnover-2	90 min	50 ul MeCN	2	0.7	2.1	0.6	**95**	0.9	0.4	0.2	95.7	**99.6**
Turnover-3	45 min	NEAT	3	0.8	2.7	0.7	3	0.6	**91**	1.1	94.3	**3.2**
Turnover-3	90 min	50 ul MeCN	2	0.8	1.9	0.7	**96**	1	0.4	0.2	96.0	**99.6**
Turnover-4	45 min	NEAT	4	0.8	3.5	0.7	2.2	0.6	**90**	1.1	92.5	**2.4**
Turnover-4	90 min	50 ul MeCN	2	0.5	1.4	0.5	**97**	0.7	0.4	0.2	97.1	**99.6**
Turnover-5	45 min	NEAT	4	0.6	3.4	0.6	1.7	0.5	**91**	1	92.8	**1.8**
Turnover-5	90 min	50 ul MeCN	2	0.5	1.4	0.5	**97**	0.7	0.4	0.2	97.1	**99.6**
Turnover-6	45 min	NEAT	5	0.7	4.5	0.6	3.5	0.7	**87**	1.1	90.4	**3.9**

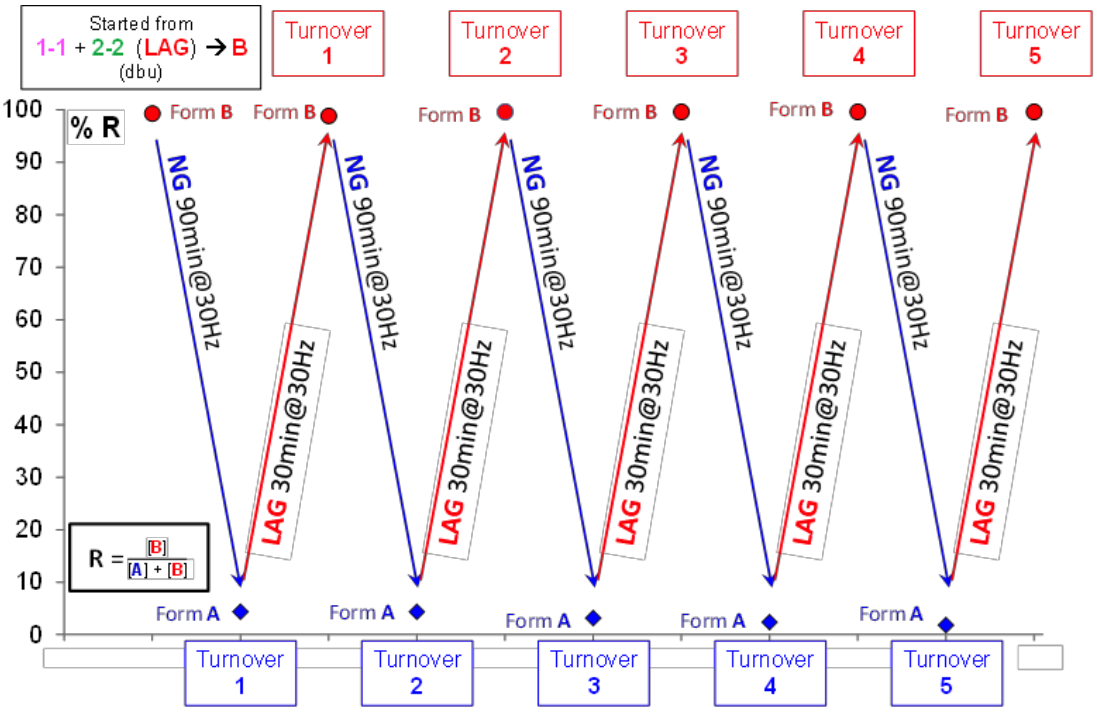

Figure S 28 %R versus polymorphic transformation step, NG followed by LAG followed by NG and so on in turnover experiments; The "turnover" polymorph conversion experiment has been repeated 5 times with dbu.

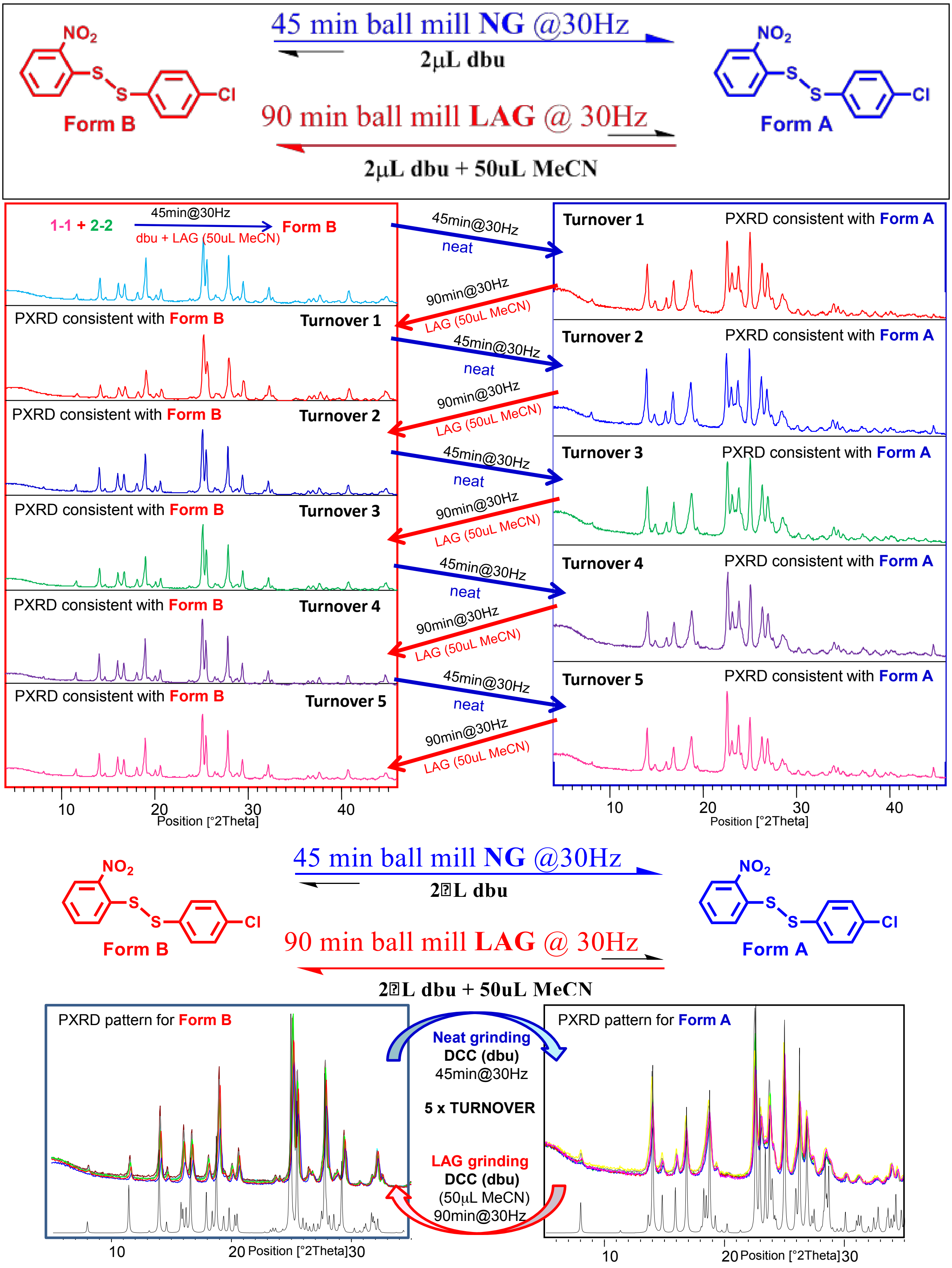

Figure S 29 Turnover experiments of in situ polymorph interconversion between **Form B** and **Form A**. The starting point is the formation of **Form B** by LAG (50mL MeCN) from equimolar amounts of the heterodimers **1-1** and **2-2** under base catalysed conditions (2%M dbu) promoting dynamic covalent chemistry. For neat grinding the grinding jar containing **Form B** was left open overnight in fumehood to remove any traces of solvent. Calculated patterns for **form B** (left) and **form A** (right) are shown in black.

6.3.2 Example 2 (3 turnover experiments performed)

Table S 15 Turnover experiments of in situ polymorph interconversion between **Form B** and **Form A** in the presence of dbu, promoting dynamic covalent chemistry. The starting point is the formation of **Form B** by LAG (50uL MeCN) from equimolar amounts of the heterodimers 1-1 and 2-2 under base catalyzed conditions (2%M dbu) . In situ refers here to the sample being left in the grinding jar throughout the experiment. For neat grinding the grinding jar containing **Form B** was left open overnight in fumehood to remove any traces of solvent.

Turnover polymorph interconversion between **Form A** & **Form B** in presence of dbu

Turnover experiments	grinding time @ 30Hz to equilibrium.	grinding conditions	1-1		2-2		Form B		Form A		[A]+[B]	R = [B] / ([A]+[B])
		dbu present	%M	e.s.d. mol%	%M	e.s.d. mol%	%M	e.s.d. mol%	%M	e.s.d. mol%	total polymorph	% R
Starting material : contains 2uL dbu			2.3	0.6	2.1	0.6	**95**	0.9	0.9	0.3	95.7	**99 %**
Turnover-1	45 min	NEAT	2.7	0.6	2.4	0.6	4.1	0.6	**91**	1	94.8	**4 %**
Turnover-1	90 min	50 ul MeCN	2.1	0.7	1.9	0.6	**96**	0.9	0.6	0.2	96.1	**99 %**
Turnover-2	45 min	NEAT	3.0	0.6	2.6	0.5	5.1	0.6	**89**	0.9	94.4	**5 %**
Turnover-2	90 min	50 ul MeCN	1.5	0.5	1.4	0.5	**97**	0.8	0.3	0.2	97.0	**100 %**
Turnover-3	45 min	NEAT	4.3	0.7	4.1	0.6	2.5	0.6	**89**	1.1	91.6	**3 %**
Turnover-3	90 min	50 ul MeCN	2.9	0.7	2.8	0.6	**94**	0.9	0.4	0.2	94.3	**100 %**

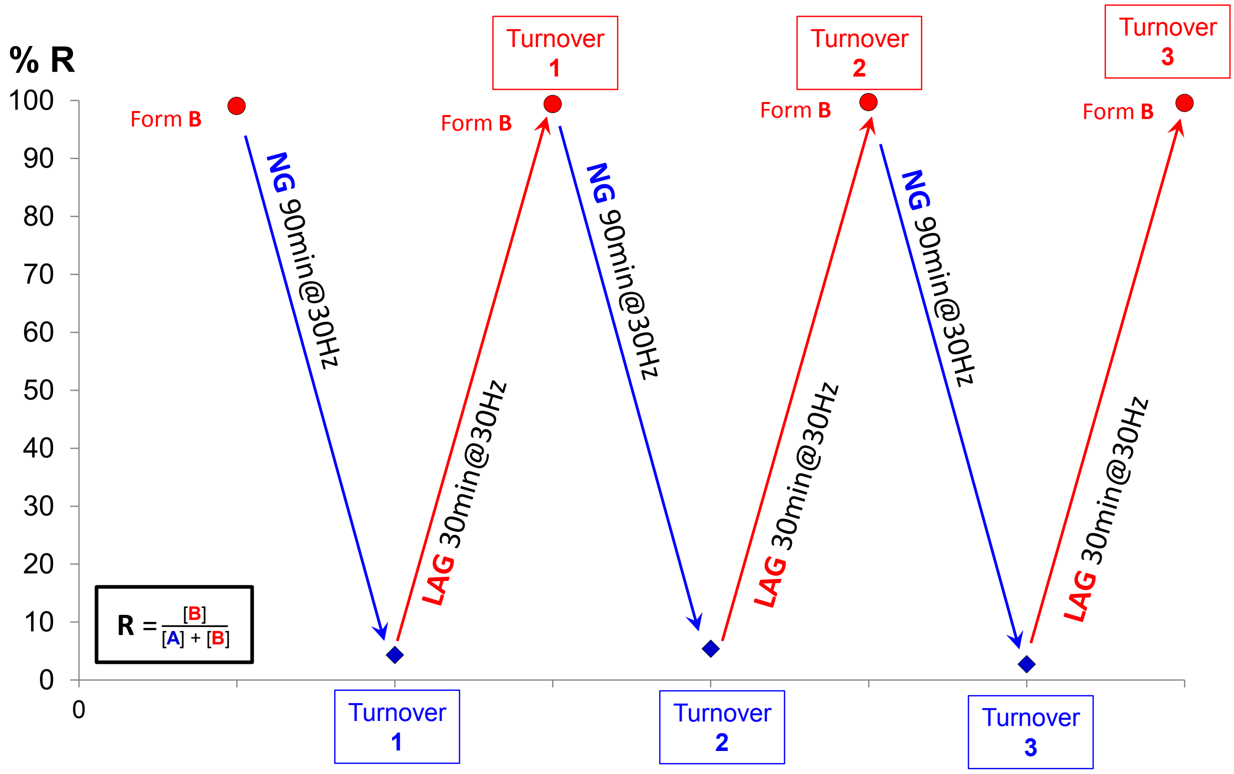

Figure S 30 %R versus polymorphic transformation step, NG followed by LAG followed by NG and so on in turnover experiments;. The "turnover" polymorph conversion experiment has been repeated 3 times with dbu.

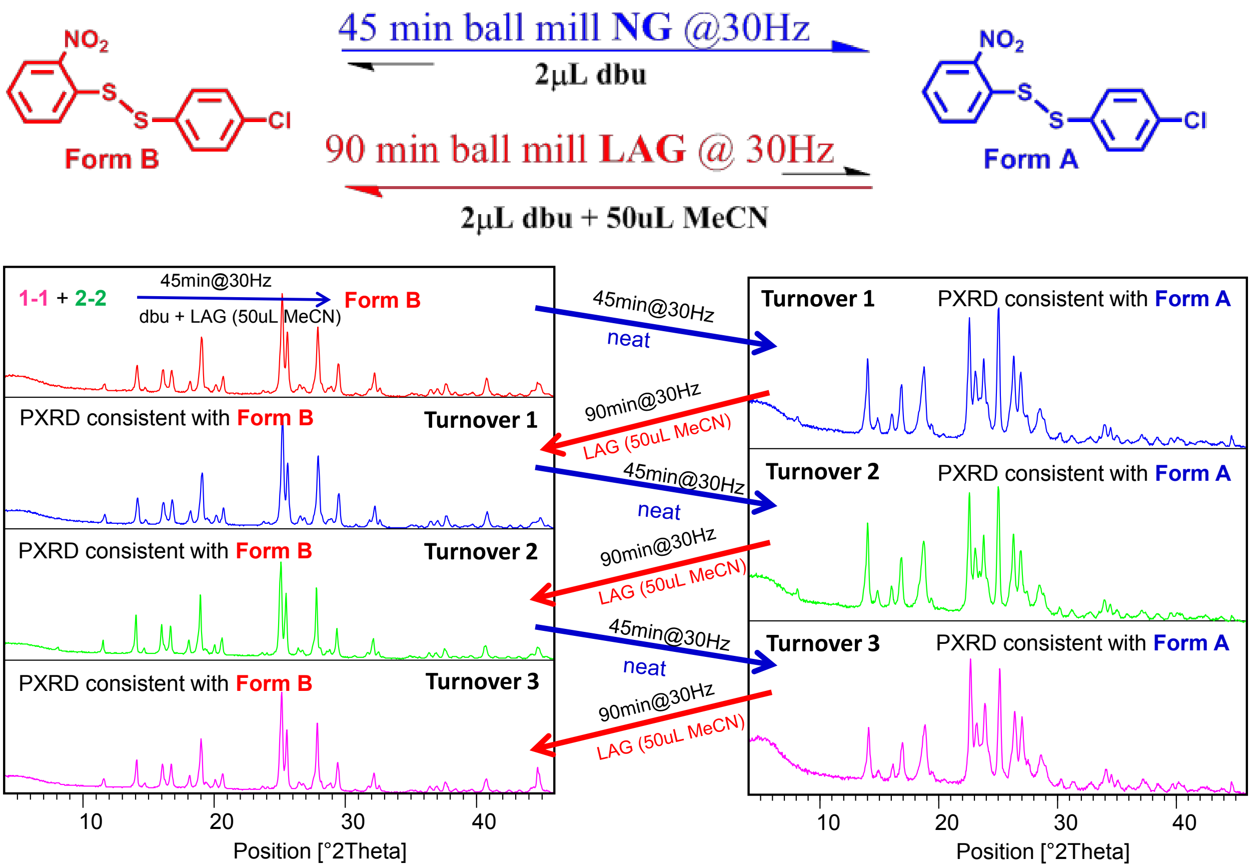

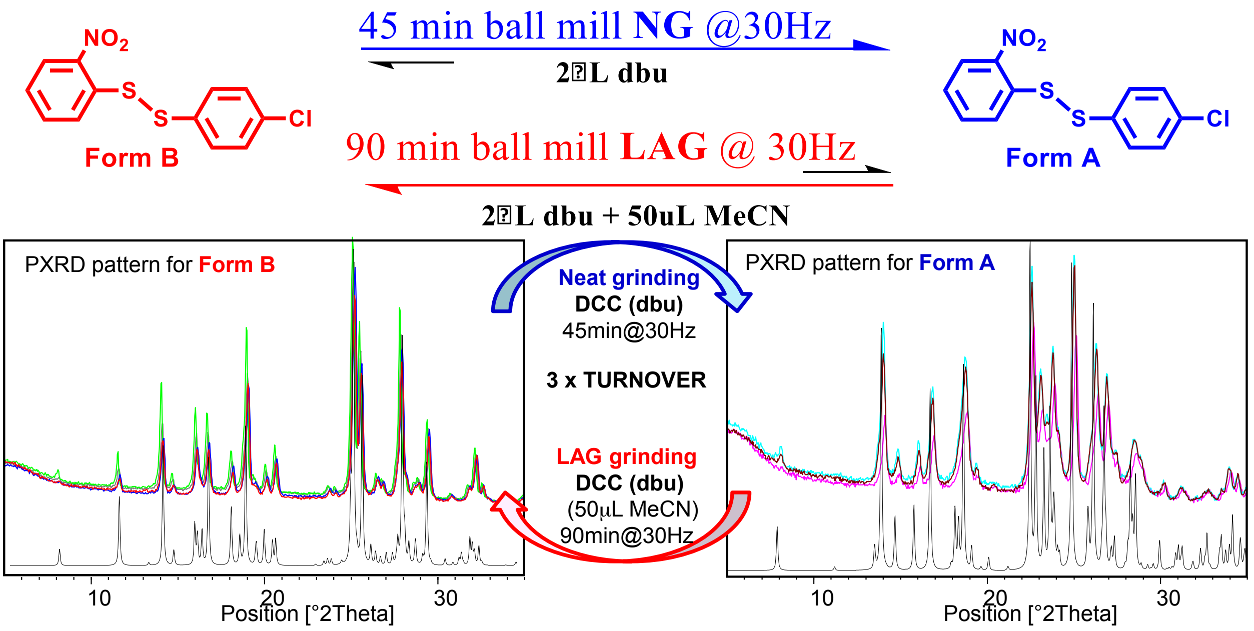

Figure S 31 Turnover experiments of in situ polymorph interconversion between **Form B** and **Form A**. The starting point is the formation of **Form B** by LAG (50uL MeCN) from equimolar amounts of the heterodimers **1-1** and **2-2** under base catalyzed conditions (2%M dbu) promoting dynamic covalent chemistry. For neat grinding the grinding jar containing **Form B** was left open overnight in fumehood to remove any traces of solvent. Calculated patterns for **form B** (left) and **form A** (right) are shown in black.

7 Direct Polymorph interconversion between Form B and Form A in the absence of base catalyst

7.1 Kinetic studies for direct polymorph transformation from Form B to Form A under ball mill neat grinding conditions (absence of catalyst).

Table S 16 Ball mill Neat grinding of quantitative **Form B** with no dbu (HPLC analysis)
Chemical composition by HPLC analysis of the reaction mixture (%M) versus grinding time at 30 Hz. Each point in the graph is an individual experiment. This table includes the HPLC method used.

Ball mill **neat grinding** : Starting from Form **B crystals** (98.5%M)						
no dbu						
Form **B**	Form **B** obtained from recrytallisation from IPA			**HPLC conditions**		
MW 296.97	obtained from recrytallisation from IPA			Zorbax SB C18, 1.8 μm; 4.6mm ID x30 mm		
97%M	from (1-2)B containing dbu			A: H_2O+0.1% HCOOH; B: MeCN+0.1%HCOOH		
0.38 mmol	Crystals are absent of dbu			0-2 min 75-85%B; 2ml/min; 60°C; 259 nm (8nm)		
200 mg	Crystals have 98.5%M **Form B** & 15%M of **1-1**.					
crystals of **Form B** as starting material			grinding @ 30 Hz time (min)	**REACTION PRODUCT COMPOSITION**		
In-situ product from solid-state DCC (mg)	(Purity by HPLC) 1-1: **1-2** : 2-2<br>1-2 = $2NO_2PhSSPh4Cl$	PXRD consistent with		**$(2NO_2PhS)_2$ (1-1) %M**	$2NO_2PhSSPh4Cl$ **1-2 %M**	**$(4ClPhS)_2$ (2-2) %M**
	1.5-98.5-0.0	Form B	0	1.4	**98.6**	0
200.18	1.5-98.5-0.0	Form B	45	1.3	**98.7**	0
200.14	1.5-98.5-0.0	Form B	90	1.3	**98.7**	0
198.29	1.5-98.5-0.0	Form B	300	1.3	**97.2**	0.2

Table S 17 Ball mill Neat grinding of quantitative **Form B** with no dbu (PXRD analysis)
Tabulation of the Rietveld refinement of PXRD data calculated as %M of the phase composition of the mixture of homodimers **1-1** and **2-2** and the two polymorphs of the product **1-2**, together with its corresponding refinement error (esd) calculated as %mol, versus grinding time for each single point experiment.

Ball mill **neat** grinding: Starting from 98.5%M **Form B crystals (no dbu)**								
			contains 1.5%M of **1-1**					
grind time	**1-1**		**2-2**		**Form B**		**Form A**	
@30Hz (min)	**%M**	e.s.d. (mol%)	**%M**	e.s.d. (mol%)	**%M**	e.s.d. (mol%)	**%M**	e.s.d. (mol%)
**0**	**1.5**		**1.5**		**97**		**0**	
45	**4.2**	1.0	**4.3**	1.0	**86.6**	1.5	**4.9**	0.8
150	**2.9**	0.7	**2.9**	0.7	**90.6**	1.1	**3.6**	0.6
300	**0.4**	0.2	**0.3**	0.2	**3.1**	0.4	**96.2**	0.5

Neat grind @ 30 Hz of quantitative crystals Form B

(contains no dbu)

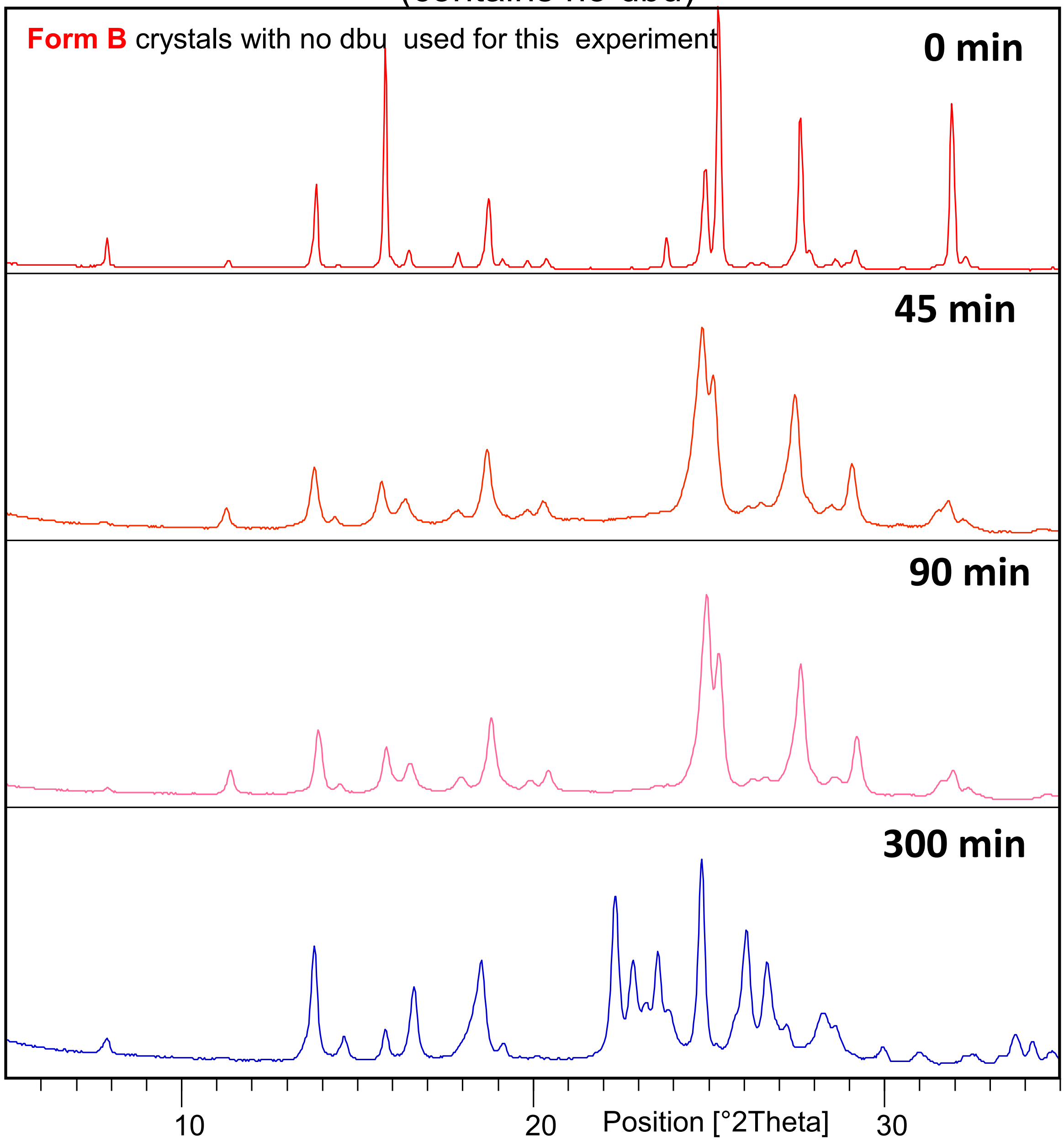

PXRD scan 3 Ball mill Neat grinding of **Form B (with no dbu).** Each PXRD scan was obtained from a single point experiment at the specified time. The PXRD sample was prepared immediately after completion of the grinding experiment, and scanned between 4 and 45° (2θ).

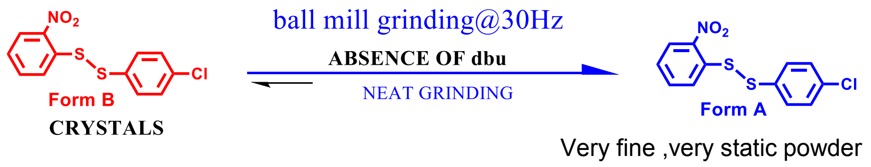

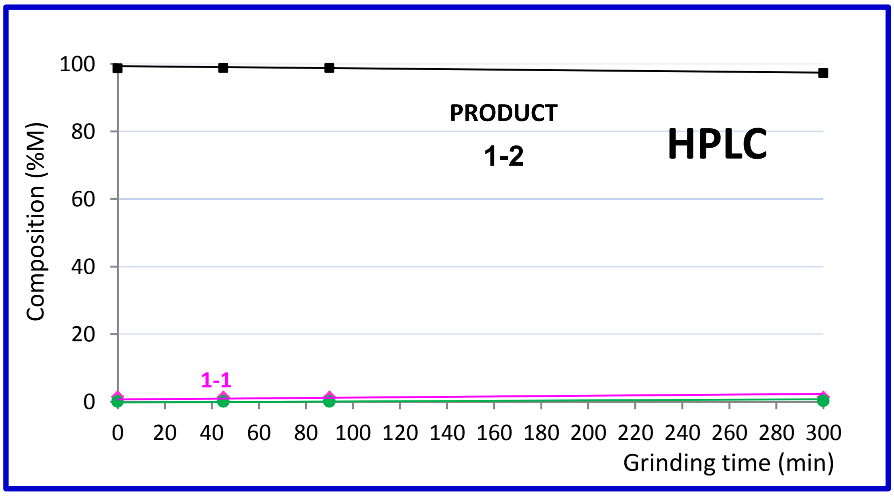

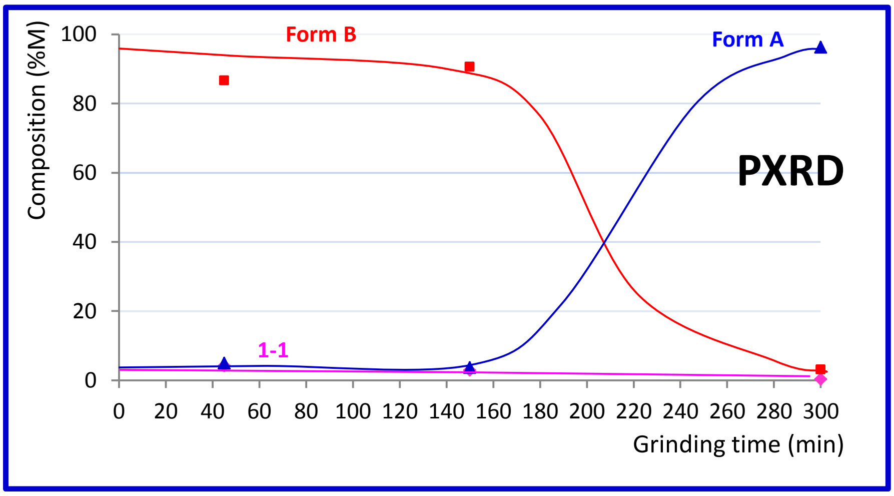

Figure S 32 Chemical and phase composition graph of ball mill neat grinding of quantitative Form B **(no dbu)**

7.2 Kinetic studies for the direct polymorph transformation from Form A to Form B under ball mill LAG conditions with 50μL MeCN (absence of catalyst).

Table S 18 Ball mill LAG of quantitative **Form A** with no dbu (HPLC analysis)
Chemical composition by HPLC analysis of the reaction mixture (%M) versus grinding time at 30 Hz. Each point in the graph is an individual experiment. This table includes the HPLC method used.

Ball mill LAG : starting from Form A (97%M)							
(LAG 50μL MeCN, absent of dbu as dbu has been quenched with HCl)							
Form A	Form A is thermodynamic product			HPLC conditions			
MW 296.97	obtained from ball mill LAG grinding			Zorbax SB C18, 1.8 μm; 4.6mm ID x30 mm			
97%M	contains dbu as base catalyst .			A: H_2O+0.1% HCOOH; B: MeCN+0.1%HCOOH			
0.38 mmol	dbu quenchd with HCl			0-2 min 75-85%B; 2ml/min; 60°C; 259 nm (8nm)			
200 mg	and 1.5%M of 1-1 & 1.5%M of 2-2.						
"in-situ" Form A as starting material			grinding @ 30 Hz time (min)	REACTION PRODUCT COMPOSITION			
In-situ product from solid-state DCC (mg)	(Purity by HPLC) 1-1: **1-2** : 2-2 1-2= $2NO_2PhSSPh4Cl$	PXRD consistent with		$(2NO_2PhS)_2$ (1-1) %M	$2NO_2PhSSPh4Cl$ 1-2 %M	$(4ClPhS)_2$ (2-2) %M	
			0	1.5	**97.0**	1.5	
199.37	1.9-96.2-1.9	Form A	15	2.8	**94.6**	2.5	
199.43	1.9-96.2-1.9	Form A	10	2.8	**94.7**	2.5	
200.35	1.1-98.3-0.7	Form A	20	1.9	**96.4**	1.7	
200.01	no HPL C	Form A	30	2.2	**95.8**	2.1	
200.1	no HPL C	Form A	25	1.0	**98.1**	0.9	
200.05	no HPL C	Form A	45	1.1	**97.8**	1.0	

Table S 19 Ball mill LAG of quantitative **Form A** with no dbu (PXRD analysis)
Tabulation of the Rietveld refinement of PXRD data calculated as %M of the phase composition of the mixture of homodimers **1-1** and **2-2** and the two polymorphs of the product **1-2**, together with its corresponding refinement error (esd) calculated as %mol, versus grinding time for each single point experiment.

Ball mill LAG: 97%M Form A (no dbu- quenched with HCl) contains 1.5%M of 1-1 & 1.5%M of 2-2)								
grind time	1-1		2-2		Form B		Form A	
(min)	%M	e.s.d. (mol%)	%M	e.s.d. (mol%)	%M	e.s.d. (mol%)	%M	e.s.d. (mol%)
**0**	**1.5**		**1.5**		**97**		**0**	
5	**1.3**	0.4	**1.4**	0.5	**3.9**	0.7	**93.4**	0.9
15	**2.3**	0.4	**2.4**	0.3	**9.3**	0.5	**86.0**	0.6
10	**2.4**	0.3	**2.4**	0.3	**3.6**	0.4	**91.6**	0.6
20	**0.7**	0.2	**0.8**	0.2	**36.8**	0.5	**61.7**	0.5
25	**1.1**	0.4	**1.1**	0.3	**95.6**	0.6	**2.2**	0.4
30	**0.8**	0.3	**0.8**	0.2	**98.0**	0.4	**0.4**	0.2
45	**1.1**	0.3	**1.1**	0.3	**97.1**	0.5	**0.7**	0.3

LAG (50μL MeCN) starting with quantitative **Form A**
with no dbu (quenched with HCl)

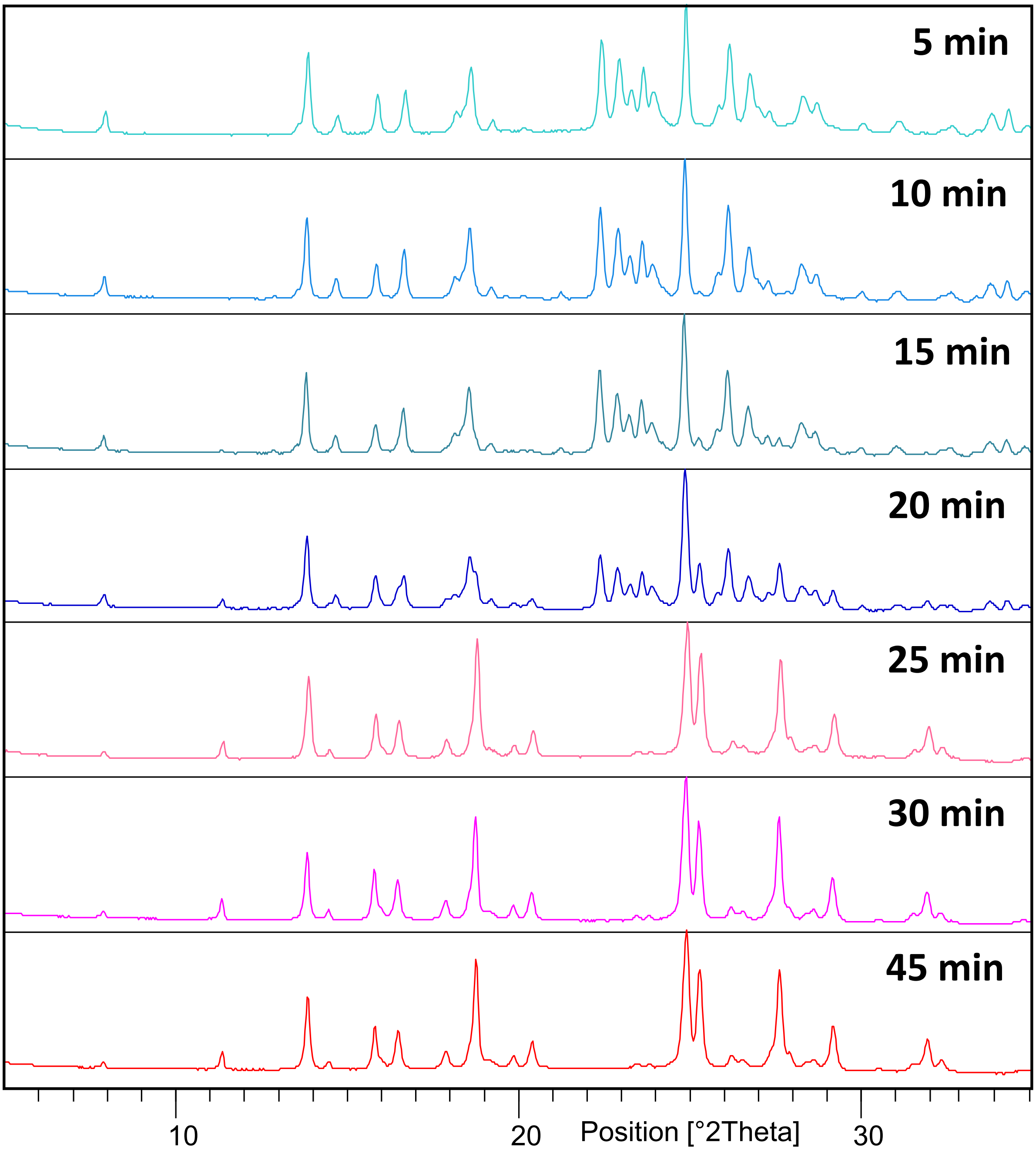

PXRD scan 4 Ball mill LAG of **Form A** **(with no dbu).** Each PXRD scan was obtained from a single point experiment at the specified time. The PXRD sample was prepared immediately after completion of the grinding experiment, and scanned between 4 and 45° (2θ).

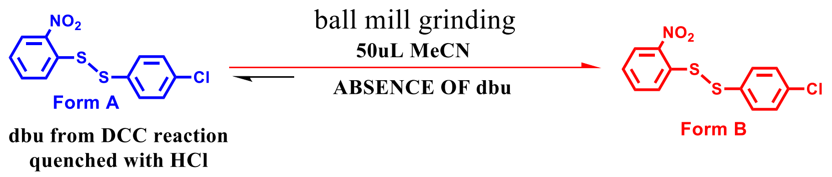

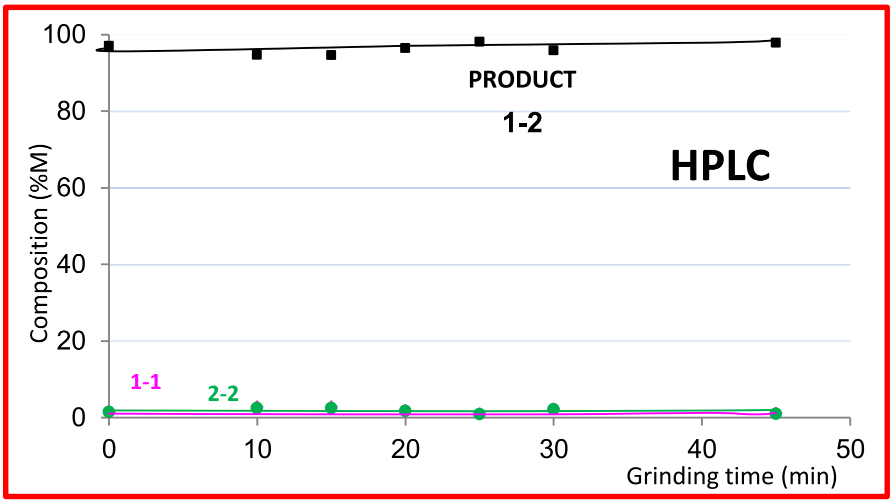

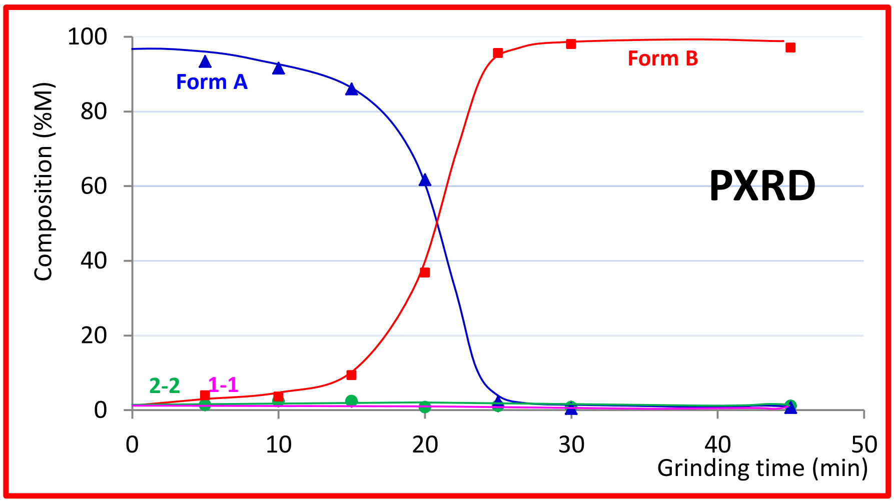

Figure S 33 Chemical and phase composition graph of ball mill LAG of quantitative **Form A** (no dbu). Kinetic curves of the reaction mixture (%M) versus grinding time at 30 Hz. Top:. chemical composition obtained from HPLC analysis; bottom: phase composition obtained from the Rietveld refinement of PXRD data. Each point in the graph is an individual experiment.

7.3 Turnover experiments for the direct polymorph interconversion between Form A and Form B in the absence of base catalysts.

Table S 20 Turnover experiments of in situ polymorph interconversion between **Form B** and **Form A**. The starting point manually ground crystals of Form B which does not contain the base catalyst dbu. In situ refers here to the sample being left in the grinding jar throughout the experiment. For neat grinding, the grinding jar containing **Form B** was left open overnight in the fumehood to remove any traces of solvent.

Turnover polymorph interconversion between **Form A** & **Form B** in the absence of dbu

Turnover experiments	grinding time @30Hz to equilibrium.	grinding conditions	1-1		2-2		Form B		Form A		[A]+[B]	R = [B]/([A]+[B])
		dbu absent	%M	e.s.d. mol%	%M	e.s.d. mol%	%M	e.s.d. mol%	%M	e.s.d. mol%	total polymorph	% R
Starting Material:crystals of **Form B**			1.5	0.5	0	0	**98.5**	1.0	0	0	98.5	**100 %**
Turnover-1	3x50 min	NEAT	1.8	0.3	1.7	0.3	3.8	0.3	**92.7**	0.5	96.5	**4 %**
Turnover-1	30 min	50 ul MeCN	1.8	0.3	1.6	0.3	**95.9**	0.4	0.7	0.2	96.6	**99 %**
Turnover-2	3x50 min	NEAT	1.7	0.3	1.7	0.3	3.6	0.4	**93.0**	0.6	96.6	**4 %**
Turnover-2	30 min	50 ul MeCN	1.6	0.3	1.5	0.3	**95.8**	0.5	1.1	0.2	96.9	**99 %**
Turnover-3	3x50 min	NEAT	1.0	0.3	0.9	0.2	3.1	0.4	**95.0**	0.5	98.1	**3 %**
Turnover-3	30 min	50 ul MeCN	1.4	0.3	1.3	0.3	**94.4**	0.5	1.7	0.3	96.1	**98 %**
Turnover-4	3x50 min	NEAT	1.4	0.3	1.3	0.3	4.1	0.5	**93.2**	0.6	97.3	**4 %**
Turnover-4	30 min	50 ul MeCN	1.4	0.3	1.3	0.3	**96.4**	0.5	1.0	0.3	97.4	**99 %**
Turnover-5	3x50 min	NEAT	2.1	0.4	1.3	0.3	4.1	0.5	**93.2**	0.6	97.3	**4 %**
Turnover-5	30 min	50 ul MeCN	2.4	0.6	2.1	0.6	**91.7**	1	3.7	0.6	95.4	**96 %**

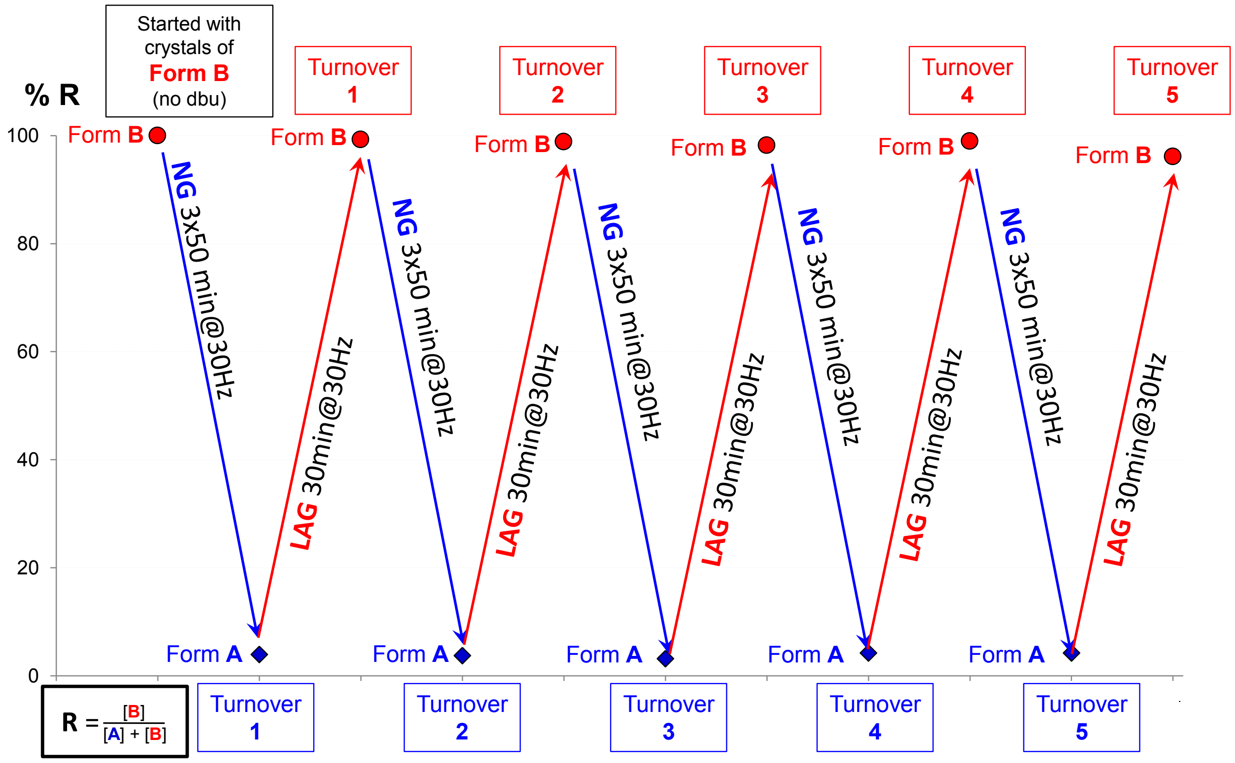

Figure S 34 graphical representation of %R versus each part of turnover experiments of polymorph interconversion between **Form B** (formed under ball mill LAG conditions to equilibrium in presence of 50μL MeCN) and **Form A** formed under ball mill neat grinding conditions to equilibrium. All grinding experiments performed in absence of dbu.

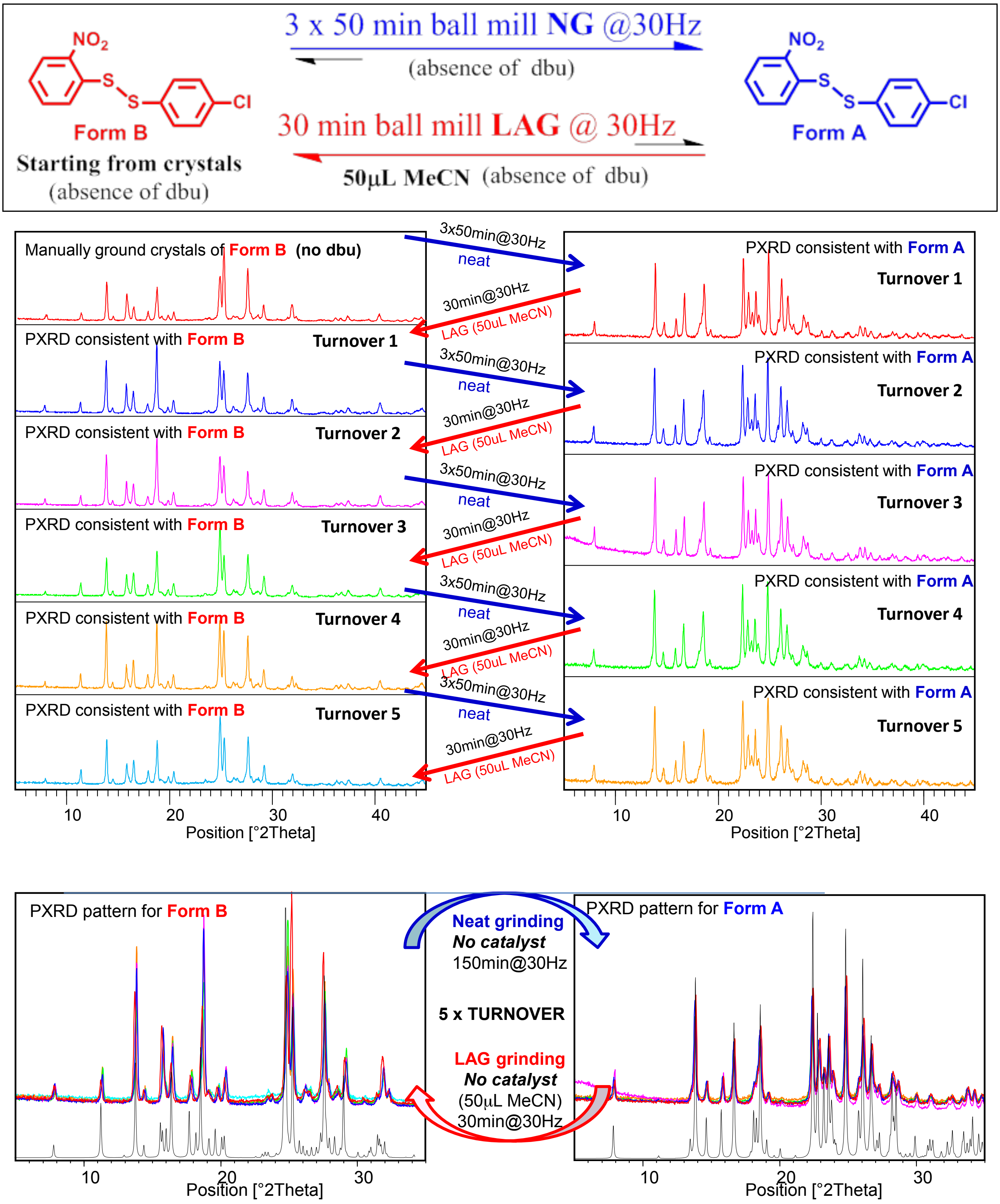

Figure S 35 30Turnover experiments of in situ polymorph interconversion between Form B and Form A. The starting point is the formation of Form B by LAG (50µL MeCN) from equimolar amounts of the heterodimers 1-1 and 2-2 under base catalyzed conditions (2%M dbu) promoting dynamic covalent chemistry. For neat grinding the grinding jar containing **Form B** was left open overnight in fumehood to remove any traces of solvent. Calculated patterns for **form B** (left) and **form A** (right) are shown in black.

8 Formation of disulfide polymorphs (Form A and Form B) in System i-i by ball mill LAG using 16 LAG solvents

8.1 Tabulation of the locations for each solvent system

Table S 21: list of location of data for each solvent.

Solvent used in solid state DCC reaction	Section	Procedure used for solid state DCC reaction	Accuracy & precision dispensing solvent	HPLC data for individual experimental points for thermodynamic curve	PXRD spectra for individual experimental points for thermodynamic curve	PXRD: Rietvelt refinement: Table	Thermodynamic graphs %R versus solvent added to solid state DCC reaction
**MeCN**	**8.2**	Proc. 1	Table S22 Fig. S 37	Table S 23	PXRD scan S-05	Table S 24	Fig. S 38
**Acetone**	**8.3**	Proc. 1	Table S25 Fig. S 39	Table S 26	PXRD scan S-06	Table S 27	Fig. S 40
**THF**	**8.4**	Proc. 1	Table S28 Fig. S 41	Table S 29	PXRD scan S-07	Table S 30	Fig. S 42
**DMF**	**8.5**	Proc. 1	Table S31 Fig. S 43	Table S 32	PXRD scan S-08	Table S 33	Fig. S 44
**EtOAc**	**8.6**	Proc. 1	Table S34 Fig. S 45	Table S 35	PXRD scan S-09	Table S 36	Fig. S 46
**$CHCl_3$**	**8.7**	Proc. 1	Table S37 Fig. S 47	Table S 38	PXRD scan S-10	Table S 39	Fig. S 48
**DCM**	**8.8**	Proc. 2	Table S40 Fig. S 49	Table S 41	PXRD scan S-11	Table S 42	Fig. S 50
**DMSO**	**8.9**	Proc. 4	Table S43 Fig. S 51	Table S 44	PXRD scan S-12	Table S 45	Fig. S 52
**MeOH**	**8.10**	Proc. 1		Table S 46	PXRD scan S-13	Table S 47	Fig. S 53
**MeOH**	**8.11**	Proc. 3	Table S48 Fig. S 54	Table S 49	PXRD scan S-14	Table S 50	Fig. S 55
**EtOH**	**8.12**	Proc. 4	Table S51 Fig. S 56	Table S 52	PXRD scan S-15	Table S 53	Fig. S 57
**IPA**	**8.13**	Proc. 1		Table S 54	PXRD scan S-16	Table S 55	Fig. S 58
**IPA**	**8.14**	Proc. 4	Table S56 Fig. S 59	Table S 57	PXRD scan S-17	Table S 58	Fig. S 60
**Water**	**8.15**	Proc. 5		Table S 59	PXRD scan S-18	Table S 60	Fig. S 61
**Cyclo-hexane**	**8.16**	Proc. 4		Table S 61	PXRD scan S-19	Table S 62	Fig. S 62
**Benzene**	**8.17**	Proc. 4		Table S 63	PXRD scan S-20	Table S 64	Fig. S 63
**Toluene**	**8.18**	Proc. 4		Table S 65	PXRD scan S-21	Table S 66	Fig. S 64
**Perfluoro decalin**	**8.19**	Proc. 4		Table S 67	PXRD scan S-22	Table S 68	Fig. S 65

MeCN (Acetonitrile);Acetone; THF(tetrahydrofuran); DMF (dimethylformamide); EtOAc (Ethyl Acetate); $CHCl_3$ (chloroform); DCM (dichloromethane); DMSO (dimethylsulfoxide); MeOH (methanol); EtOH (ethanol); IPA (isopropanol); water; cyclohexane; benzene; toluene; F18-decalin (perfluorodecalin)

8.2 Solid-state DCC reaction by ball mill LAG using MeCN as LAG solvent (Procedure 1)

These experiments were performed by adding the solvent by reverse pipetting using Procedure 1 (Section 4.5.1).

8.2.1 Accuracy and precision of dispensing MeCN (Procedure 1)

Table S 22 Tabulation of accuracy and precision of MeCN dispensed, calibrated by weighing experiments

Density:	0.786		24.0°C		bp:	82°C					
Dispensed volume (μL)	**REVERSE PIPETTING** aspiration speed = 1, dispensing speed = 1						Average weight (mg)	Std deviation	Relative Std dev	volume MeCN (μL)	Accuracy error (%)
	1	2	3	4							
10	7.58	7.72	7.98	9.17			8.11	0.72	8.93	10.32	3.11
20	15.38	15.81	16.10				15.76	0.36	2.30	20.06	0.27
21	16.72	16.71	16.58				16.67	0.08	0.47	21.21	0.98
22	17.45	17.40	17.55				17.47	0.08	0.44	22.22	1.00
23	18.45	18.28	18.10				18.28	0.18	0.96	23.25	1.09
24	18.80	18.84	19.07				18.90	0.15	0.77	24.05	0.21
25	19.11	19.86	19.88				19.62	0.44	2.24	24.96	-0.17
26	20.72	20.65	20.62				20.66	0.05	0.25	26.29	1.10
27	21.00	21.34	21.15				21.16	0.17	0.81	26.93	-0.28
30	23.56	23.50	23.73				23.60	0.12	0.51	30.02	0.07
40	31.91	31.60	31.5				31.67	0.21	0.68	40.29	0.73
50	38.99	39.16	39.61				39.25	0.32	0.82	49.94	-0.12
60	47.30	47.33	47.31				47.31	0.02	0.03	60.20	0.32
70	55.22	55.35	55.07				55.21	0.14	0.25	70.25	0.35
80	63.20	63.07	63.1				63.12	0.07	0.11	80.31	0.39
90	70.76	70.77	71.06				70.86	0.17	0.24	90.16	0.17
100	79.02	78.91	78.89				78.94	0.07	0.09	100.43	0.43

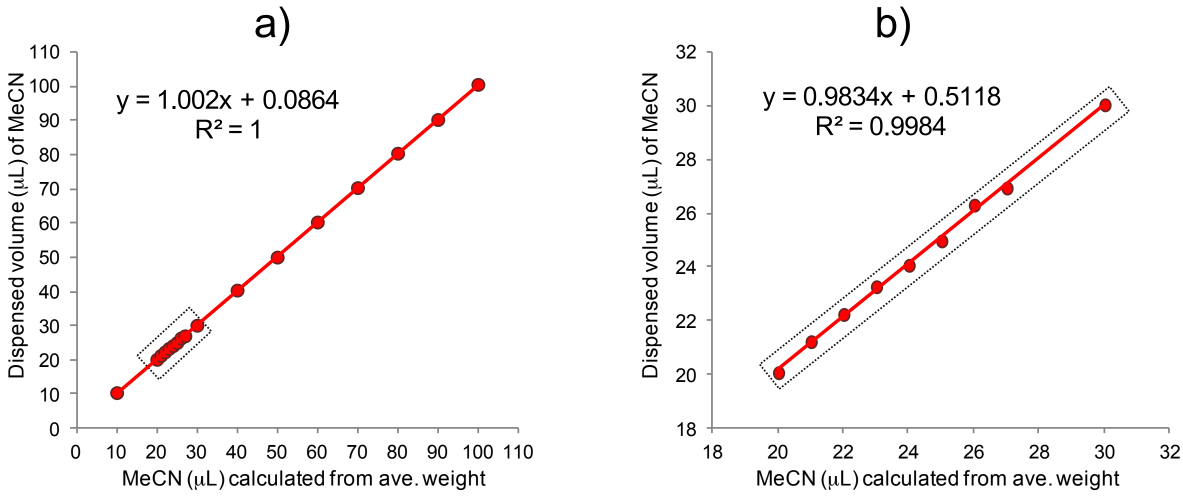

Figure S 36 Accuracy and precision of volume dispensed, calibrated by weighing experiments.
a) Range 10-100μL MeCN; b) expanded narrow range from 20-30μL MeCN

8.2.1 Experimental details and HPLC data (Procedure 1)

Table S 23 Experimental details of ball mill LAG experiments with **MeCN** as grinding solvent used to prepare the milling equilibrium curves and corresponding HPLC data. Tabulation of weights (mg) of starting materials (**1-1** & **2-2**), volume of **MeCN** added to the solid state DCC reaction and the corresponding grinding time used to ensure that thermodynamic equilibrium is achieved for each single experimental point. The chemical composition of the reaction product as %M obtained by HPLC analysis is tabulated. Details of HPLC method are included.

Ball mill LAG taken to thermodynamic equilibrium using Procedure 1

2%M dbu + μL **MeCN** added to 0.68 mmol **DCC** ([**1-1**] + [**2-2**] + [**1-2**])

Reagents:	**$(2NO_2PhS)_2$**	**$(4ClPhS)_2$**	MeCN added to powder		**HPLC conditions**		
MW:	308.33	287.23			Zorbax XDB C18, 1.8 μm; 4.6x50 mm		
%M initial	50%M	50%M	MW (g/mol)	Densiity (g/mL)	A: H_2O+0.1% FA; B: MeCN+0.1%FA		
mmol	0.34	0.34			0-2min 75-85%B;		
mg	104.8 mg	97.6 mg	41.05	0.7760	2ml/min; 60°C; 259 nm (8nm bandwidth)		
					**HPLC results**		
grinding time @ 30 Hz to equilibrium	$(2NO_2PhS)_2$ weighed (mg)	$(4ClPhS)_2$ weighed (mg)	MeCN μL	[MeCN]/[DCC] (mol/mol)	$(2NO_2PhS)_2$ (1-1) %M	Product **(1-2)** %M	$(4ClPhS)_2$ (2-2) %M
2h	104.80	97.69	**10**	**0.28**	0.7	**98.7**	0.6
2h	104.81	97.63	**15**	**0.42**	0.5	**98.9**	0.7
2h	104.76	97.67	**20**	**0.56**	0.8	**98.5**	0.7
2h	104.84	97.68	**21**	**0.58**	0.8	**98.3**	0.9
2h	104.85	97.61	**22**	**0.61**	0.8	**98.6**	0.6
2h	104.84	97.68	**23**	**0.64**	1.5	**97.3**	1.3
2h	104.88	97.65	**24**	**0.67**	1.9	**96.5**	1.6
2h	104.82	97.66	**25**	**0.69**	1.5	**97.3**	1.2
1h	104.82	97.63	**50**	**1.39**	2.2	**96.3**	1.5
3h	104.84	97.67	**75**	**2.08**	2.1	**96.2**	1.7

[DCC] is the concentration of the dynamic covalent chemistry reaction at any time =[1-1]+[2-2]+[1-2]

[1-2] = **Form A** + **Form B**

8.2.2 PXRD scans, tabulation of Rietveld refinement of PXRD data and corresponding experimental milling equilibrium curves (Procedure 1)

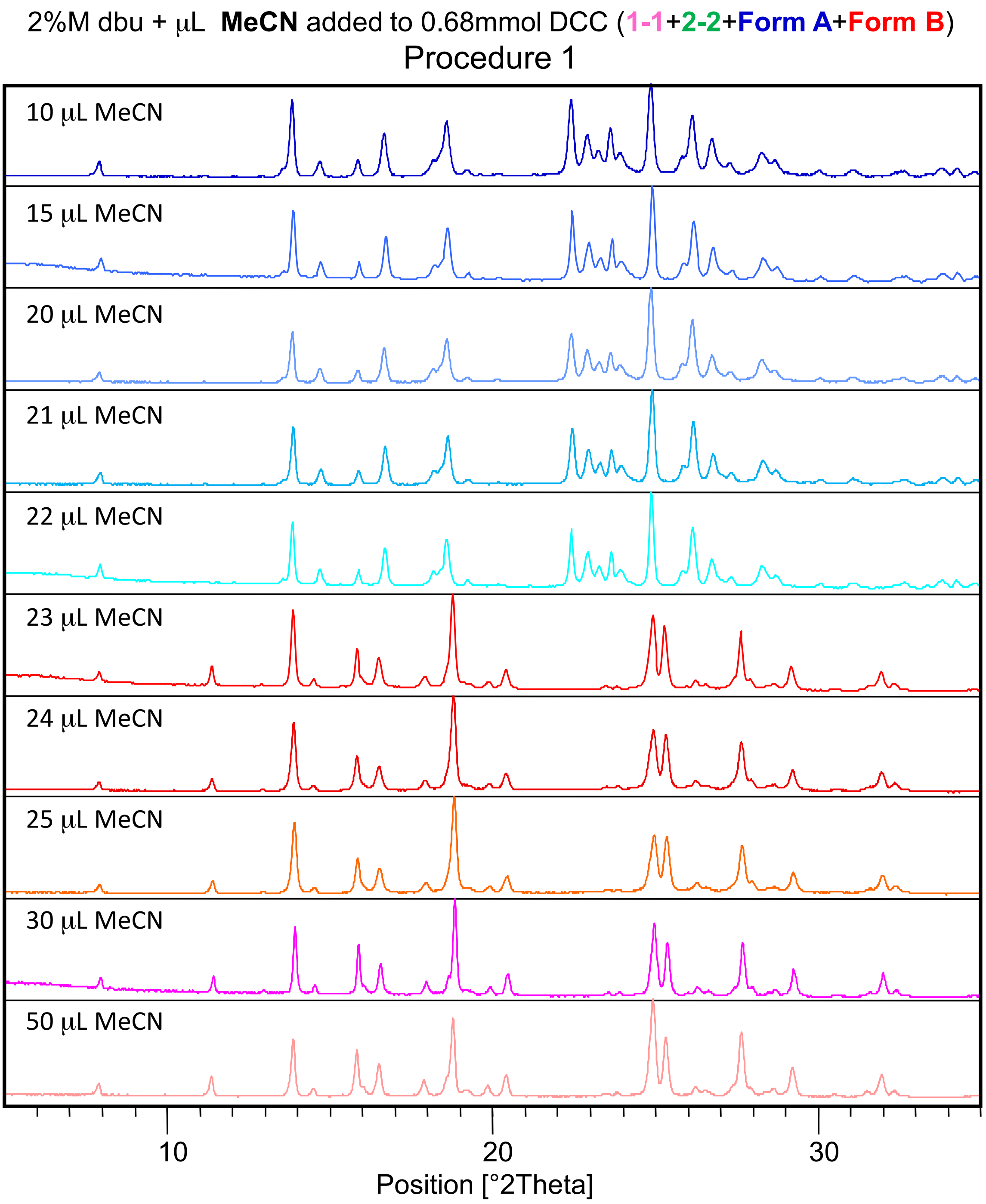

PXRD scan 5 Ball mill LAG reaction at equilibrium with different volumes of **MeCN**. Each PXRD scan was obtained at equilibrium from a single point experiment with the specified added volume of **MeCN**. The PXRD sample was prepared immediately after completion of the grinding experiment, and scanned between 5 and 45° (2θ).

Table S 24 Tabulation of the phase composition and corresponding R index used to prepare the milling equilibrium curve with **MeCN** as the LAG solvent using ball mill grinding **Procedure 1**. The phase composition was calculated by Rietveld refinement of PXRD data as %M of the mixture of starting materials (**1-1** and **2-2**) and the polymorphs of the product (**Form A** and **Form B**). The corresponding refinement errors (esd) have been calculated as %mol. The R index is calculated as the ratio between the concentration of **Form B** versus the total concentration of **Form B** plus **Form A**.

Ball mill LAG grinding reaction taken to thermodynamic equilibrium using Procedure 1												
2%M dbu+μL MeCN added to 0.68 mmol DCC ([1-1]+[2-2]+[Form A]+[Form B])												
grinding time @ 30Hz to equilib.	MeCN added to powder		1-1		2-2		Form B		Form A		[A]+[B]	R = [B] / ([A]+[B])
	MeCN μL	[MeCN]/[DCC] (mol/mol)	%M	e.s.d. mol%	%M	e.s.d. mol%	%M	e.s.d. mol%	%M	e.s.d. mol%	total polymorph	% R
2h	**10**	**0.28**	**1.1**	0.2	**0.9**	0.2	**3.9**	0.4	**94.1**	0.5	98.0	**4.0**
2h	**15**	**0.42**	**1.1**	0.3	**0.8**	0.2	**2.1**	0.6	**96.0**	0.7	98.1	**2.1**
2h	**20**	**0.56**	**1.0**	0.2	**0.8**	0.2	**2.2**	0.3	**96.0**	0.4	98.2	**2.2**
2h	**21**	**0.58**	**0.8**	0.2	**0.7**	0.1	**2.6**	0.3	**96.0**	0.4	98.6	**2.6**
2h	**22**	**0.61**	**1.1**	0.3	**0.9**	0.2	**2.5**	0.4	**95.6**	0.6	98.1	**2.5**
2h	**23**	**0.64**	**1.2**	0.3	**0.9**	0.2	**97.5**	0.4	**0.5**	0.2	98.0	**99.5**
2h	**24**	**0.67**	**1.6**	0.3	**1.3**	0.2	**95.7**	0.4	**1.4**	0.2	97.1	**98.6**
2h	**25**	**0.69**	**1.3**	0.2	**1.1**	0.2	**95.8**	0.4	**1.8**	0.3	97.6	**98.2**
2h	**50**	**1.39**	**1.6**	0.5	**1.3**	0.4	**96.3**	0.9	**0.8**	0.6	97.1	**99.2**
2h	**75**	**2.08**	**0.8**	0.2	**0.7**	0.2	**97.7**	0.3	**0.8**	0.2	98.5	**99.2**

[DCC] is the concentration of the dynamic covalent chemistry reaction at any time =[**1-1**]+[**2-2**]+[**Form A**]+[**Form B**]
[**1-2**] = **Form A** + **Form B**   [**A**] ≡ [**Form A**]   [**B**] ≡ [**Form B**]

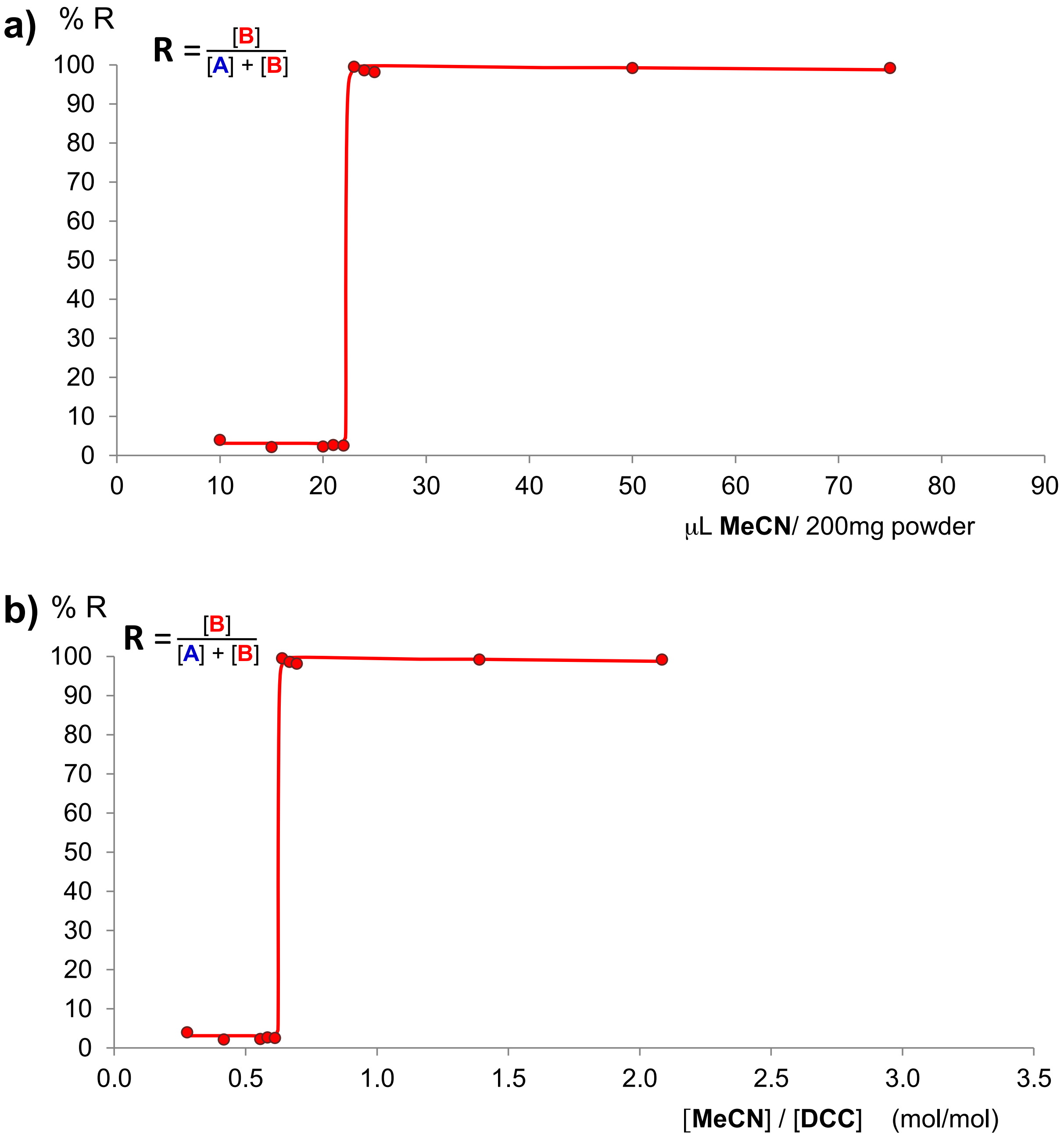

[**DCC**] is the concentration of the dynamic covalent chemistry reaction at any time = [**1-1**]+[**2-2**]+[**A**]+[**B**]
[**A**] is the concentration of **Form A** in DCC; [**B**] is the concentration of **Form B** in DCC

Figure S 37 Experimental milling equilibrium curves using “ball mill grinding procedure 1” plotted as the concentration of acetonitrile versus %R index. R is the ratio of the concentration of **Form B** with respect to the total concentration of **Form B** plus **Form A** formed in the solid state DCC reaction for each specific experimental point. **a)** Curve expressed as μL MeCN / 200 mg powder and **b)** curve expressed as mols of MeCN added to the reaction with respect to 0.68 mmol of DCC library; this library is composed by the homodimers **1-1** and **2-2** and the heterodimers **Form A** and **Form B**. R is calculated from the Rietveld refinement of PXRD data giving the phase composition of the solid-state DCC experiment at equilibrium as %M. Each point in the graph is an individual experiment. No fitting was performed and the curves drawn are only a guide to the eye.

8.3 LAG experiments using Acetone as grinding liquid on solid-state DCC reaction.

These experiments were performed by adding the solvent by reverse pipetting using Procedure 1 (Section 4.5.1).

8.3.1 Accuracy and precision of dispensing acetone (Procedure 1)

Table S 25 Tabulation of accuracy and precision of acetone dispensed, calibrated by weighing experiments

Density:	0.786		24.0°C		bp:	56°C					
Dispensed volume (µL)	**REVERSE PIPETTING** aspiration speed = 1, dispensing speed = 1						Average weight (mg)	Std deviation	Relative Std dev	volume Acetone (µL)	Accuracy error (%)
	1	2	3	4	5	6					
10	7.77	8.55	8.25	7.47	8.06		8.02	0.42	5.22	10.20	2.00
15	12.49	12.30	10.22	13.47	11.80	12.45	12.12	1.08	8.90	15.42	2.74
16	12.41	11.66	12.38	11.99	12.20	12.2	12.14	0.28	2.30	15.45	-3.59
17	13.09	13.56	13.42	13.38	12.87	12.84	13.19	0.30	2.30	16.79	-1.28
18	13.92	13.73	13.96	13.80			13.85	0.11	0.77	17.62	-2.13
19	14.85	14.39	14.98	15.13			14.84	0.32	2.15	18.88	-0.65
20	15.22	15.47	15.70	15.53			15.48	0.20	1.28	19.69	-1.55
21	16.25	15.20	16.02	15.88			15.84	0.45	2.85	20.15	-4.22
22	16.52	16.70	16.51	16.74	17.04	17.2	16.79	0.28	1.67	21.35	-3.02
23	17.54	17.86	18.46	17.39	17.33		17.72	0.46	2.62	22.54	-2.04
24	18.44	18.49	17.94	18.85	17.49		18.24	0.53	2.91	23.21	-3.41
25	18.84	18.95	19.6	19.13			19.13	0.34	1.75	24.34	-2.72
30	22.26	23.50	22.95	23.31	23.57		23.12	0.54	2.32	29.41	-2.00
40	28.78	31.18	30.99	31.12			30.52	1.16	3.80	38.83	-3.02
50	38.20	39.47	39.31	39.36			39.09	0.59	1.52	49.73	-0.55
60	46.96	46.52	47.62	47.7	47.18		47.20	0.49	1.03	60.05	0.08
70	55.24	55.82	56.05	55.95			55.77	0.36	0.65	70.95	1.34
80	64.05	63.60	63.6	63.53			63.70	0.24	0.38	81.04	1.28
90	68.79	69.86	70.04	69.97	70.86		69.90	0.74	1.06	88.94	-1.20
100	78.58	78.86	79.07	78.99			78.88	0.21	0.27	100.35	0.35

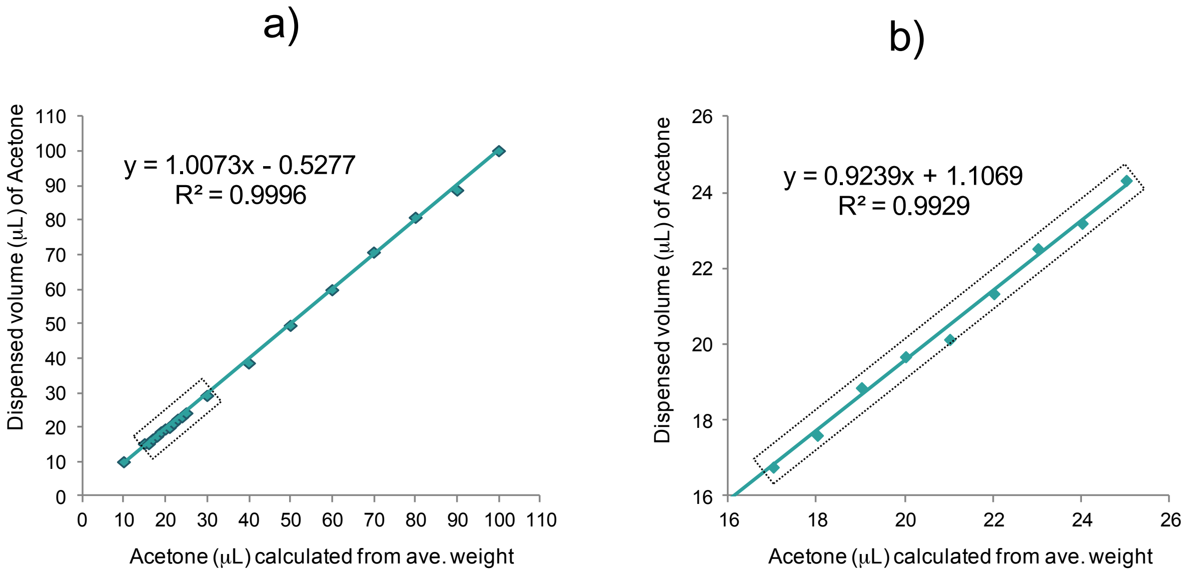

Figure S 38 Accuracy and precision of acetone dispensed, calibrated by weighing experiments.
a) Range 10-100µL acetone; b) expanded narrow range from 16-26 µL acetone

8.3.2 Experimental details and HPLC data (Procedure 1)

Table S 26 Experimental details of ball mill LAG experiments with **Acetone** as grinding solvent used to prepare the milling equilibrium curves and corresponding HPLC data. Tabulation of weights (mg) of starting materials (**1-1** & **2-2**), volume of **Acetone** added to the solid state DCC reaction and the corresponding grinding time used to ensure that thermodynamic equilibrium is achieved for each single experimental point. The chemical composition of the reaction product as %M obtained by HPLC analysis is tabulated. Details of HPLC method are included.

Ball mill LAG taken to thermodynamic equilibrium using Procedure 1							
2%M dbu + μL **acetone** added to 0.68 mmol **DCC** ([**1-1**] + [**2-2**] + [**1-2**])							
Reagents:	$(2NO_2PhS)_2$	$(4ClPhS)_2$	acetone added		**HPLC conditions**		
MW:	308.33	287.23	to powder		Zorbax XDB C18, 1.8 μm; 4.6x50 mm		
%M initial	50%M	50%M	MW	Densiity	A: H_2O+0.1% FA; B: MeCN+0.1%FA		
mmol	0.34	0.34	(g/mol)	(g/mL)	0-2min 75-85%B;		
mg	104.8 mg	97.6 mg	58.08	0.7849	2ml/min; 60°C; 259 nm (8nm bandwidth)		
					**HPLC results**		
grinding time @ 30 Hz to equilibrium	$(2NO_2PhS)_2$ weighed (mg)	$(4ClPhS)_2$ weighed (mg)	acetone μL	[acetone]/[DCC] (mol/mol)	$(2NO_2PhS)_2$ (1-1) %M	Product (1-2) %M	$(4ClPhS)_2$ (2-2) %M
45m	104.80	97.66	**10**	**0.20**	0.7	**98.6**	0.7
45m	104.81	97.66	**14**	**0.28**	0.9	**98.3**	0.8
45m	104.81	97.67	**15**	**0.30**	1.4	**97.9**	0.7
45m	104.84	97.68	**16**	**0.32**	1.0	**98.1**	0.9
45m	104.82	97.68	**17**	**0.34**	2.8	**94.8**	2.4
45m	104.81	97.65	**18**	**0.36**	3.7	**93.2**	3.1
45m	104.82	97.65	**20**	**0.40**	3.1	**94.1**	2.7
45m	104.84	97.67	**30**	**0.60**	4.1	**92.3**	3.6
45m	104.86	97.67	**50**	**0.99**	3.8	**93.0**	3.2
[DCC] is the concentration of the dynamic covalent chemistry reaction at any time =[1-1]+[2-2]+[1-2]							
[1-2] = **Form A** + **Form B**							

8.3.3 PXRD scans, tabulation of Rietveld refinement of PXRD data and corresponding experimental milling equilibrium curves (Procedure 1)

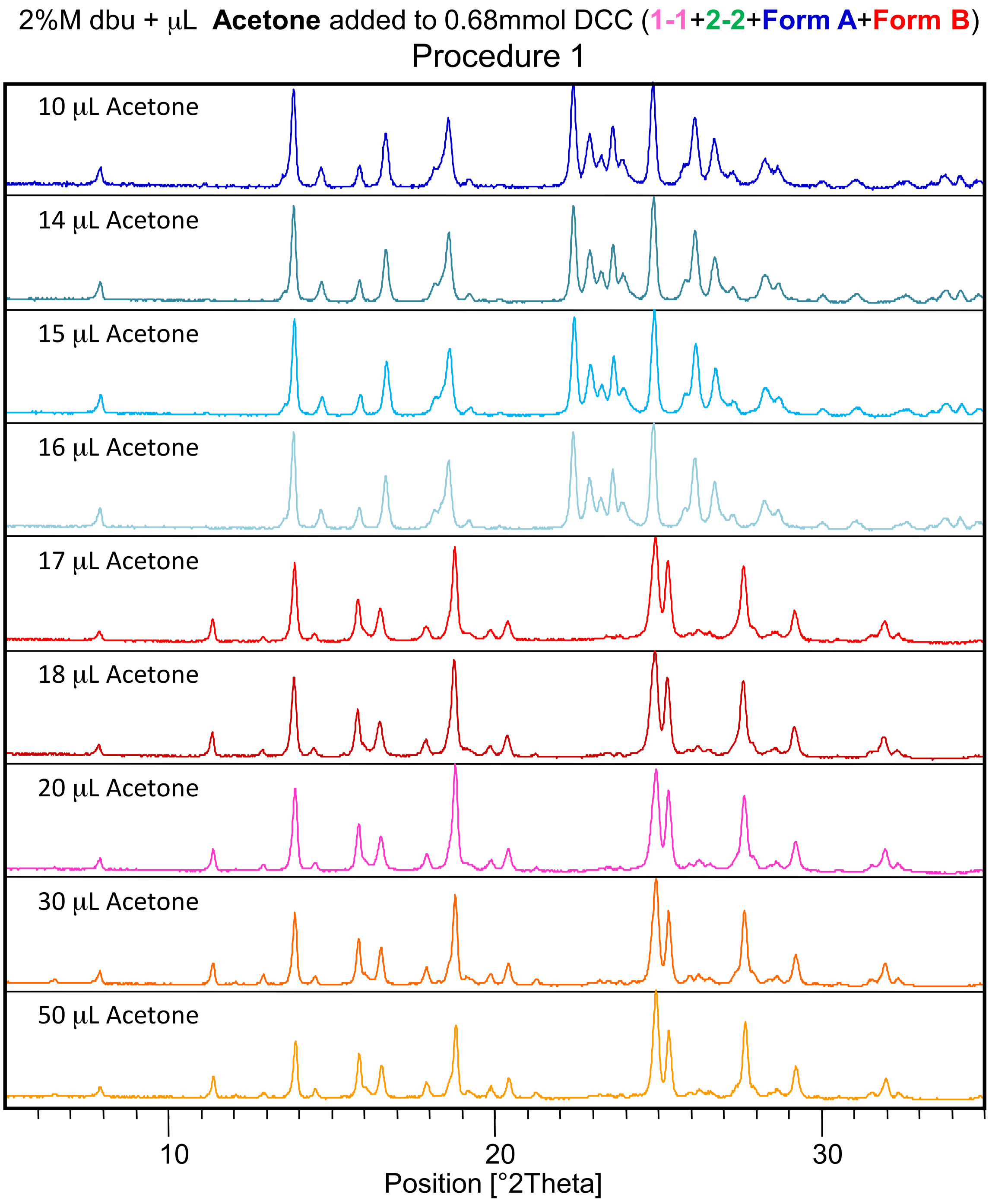

PXRD scan 6 Ball mill LAG reaction at equilibrium with different volumes of **Acetone**. Each PXRD scan was obtained at equilibrium from a single point experiment with the specified added volume of **Acetone**. The PXRD sample was prepared immediately after completion of the grinding experiment, and scanned between 5 and 45° (2θ).

Table S 27 Tabulation of the phase composition and corresponding R index used to prepare the milling equilibrium curve with **Acetone** as the LAG solvent using ball mill grinding **Procedure 1.** The phase composition was calculated by Rietveld refinement of PXRD data as %M of the mixture of starting materials (**1-1** and **2-2**) and the polymorphs of the product (**Form A** and **Form B**). The corresponding refinement errors (esd) have been calculated as %mol. The R index is calculated as the ratio between the concentration of **Form B** versus the total concentration of **Form B** plus **Form A**.

Ball mill LAG grinding reaction taken to thermodynamic equilibrium using Procedure 1												
2%M dbu+μL acetone added to 0.68 mmol DCC ([**1-1**]+[**2-2**]+[**Form A**]+[**Form B**])												
grinding time @ 30Hz to equilib.	acetone added to powder		**1-1**		**2-2**		**Form B**		**Form A**		[A]+[B]	R = [B] / ([A]+[B])
	acetone μL	[acetone]/[DCC] (mol/mol)	%M	e.s.d. mol%	%M	e.s.d. mol%	%M	e.s.d. mol%	%M	e.s.d. mol%	total polymorph	**% R**
45m	**10**	**0.20**	**1.1**	0.3	**0.9**	0.2	**2.1**	0.6	**95.9**	0.7	98.0	**2.1**
45m	**14**	**0.28**	**0.8**	0.2	**0.6**	0.1	**2.8**	0.4	**95.8**	0.4	98.6	**2.8**
45m	**15**	**0.30**	**0.7**	0.2	**0.6**	0.1	**2.1**	0.3	**96.6**	0.4	98.7	**2.1**
45m	**16**	**0.32**	**0.9**	0.2	**0.7**	0.1	**2.1**	0.3	**96.3**	0.4	98.4	**2.1**
45m	**17**	**0.34**	**2.3**	0.3	**1.8**	0.3	**92.9**	0.6	**3.0**	0.4	95.9	**96.9**
45m	**18**	**0.36**	**2.8**	0.3	**2.3**	0.2	**92.8**	0.5	**2.1**	0.3	94.9	**97.8**
45m	**20**	**0.40**	**3.1**	0.3	**2.5**	0.2	**92.3**	0.5	**2.1**	0.3	94.4	**97.8**
45m	**30**	**0.60**	**4.9**	0.3	**3.9**	0.2	**89.3**	0.5	**1.9**	0.4	91.2	**97.9**
45m	**50**	**0.99**	**4.4**	0.4	**3.6**	0.3	**90.8**	0.5	**1.2**	0.4	92.0	**98.7**

[DCC] is the concentration of the dynamic covalent chemistry reaction at any time =[**1-1**]+[**2-2**]+[**Form A**]+[**Form B**]

[**1-2**] = **Form A** + **Form B** [**A**] ≡ [**Form A**] [**B**] ≡ [**Form B**]

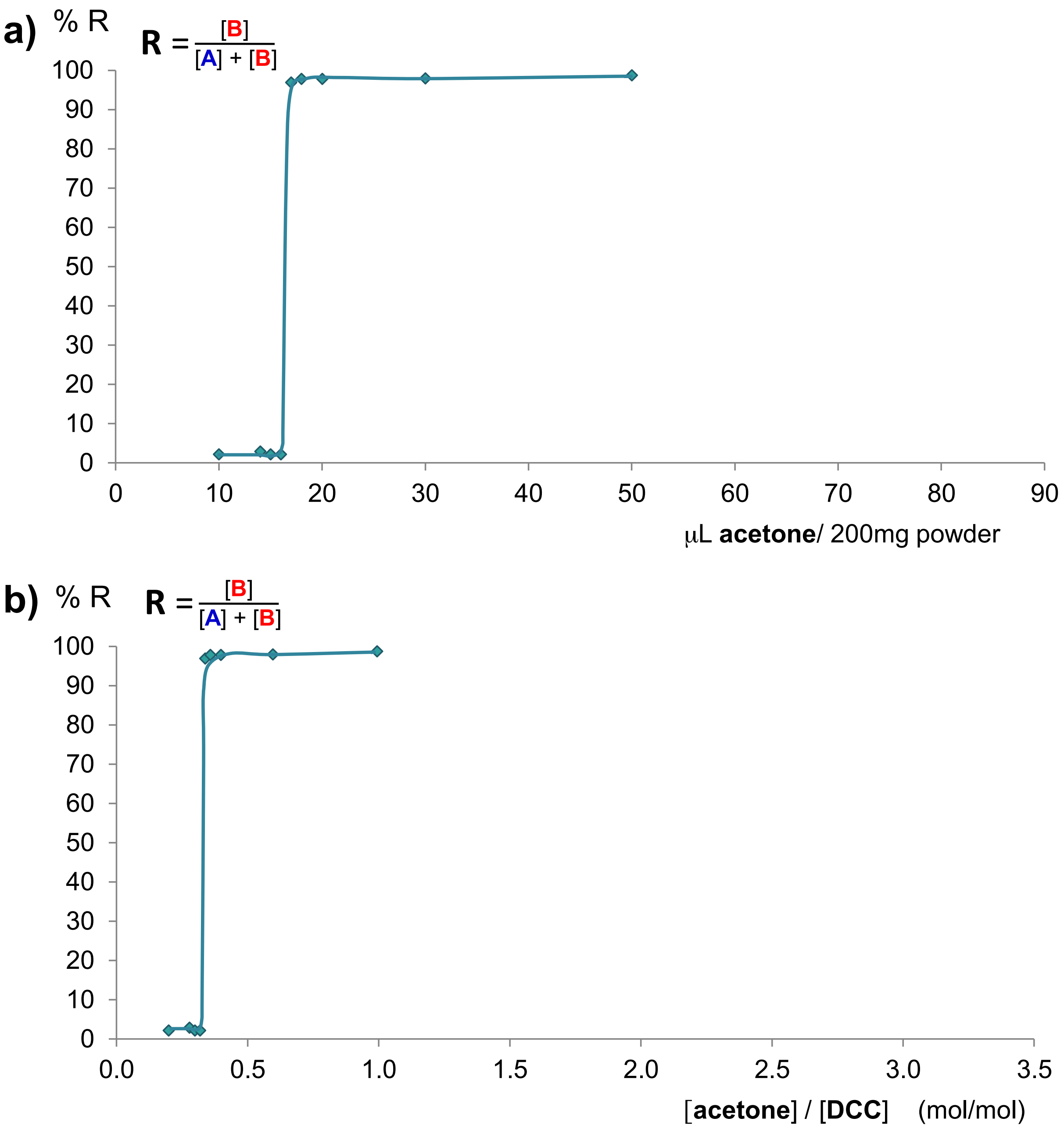

[**DCC**] is the concentration of the dynamic covalent chemistry reaction at any time = [**1-1**]+[**2-2**]+[**A**]+[**B**]

[**A**] is the concentration of **Form A** in DCC; [**B**] is the concentration of **Form B** in DCC

Figure S 39 Experimental milling equilibrium curves using "ball mill grinding procedure 1" plotted as the concentration of Acetone versus %R index. R is the ratio of the concentration of **Form B** with respect to the total concentration of **Form B** plus **Form A** formed in the solid state DCC reaction for each specific experimental point. **a)** Curve expressed as μL Acetone / 200 mg powder and **b)** curve expressed as mols of Acetone added to the reaction with respect to 0.68 mmol of DCC library; this library is composed by the homodimers **1-1** and **2-2** and the heterodimers **Form A** and **Form B**. R is calculated from the Rietveld refinement of PXRD data giving the phase composition of the solid-state DCC experiment at equilibrium as %M. Each point in the graph is an individual experiment. No fitting was performed and the curves drawn are only a guide to the eye.

8.4 LAG experiments using tetrahydrofuran as grinding liquid on solid-state DCC reaction.

These experiments were performed by adding the solvent by reverse pipetting using Procedure 1 (Section 4.5.1).

8.4.1 Accuracy and precision of dispensing THF (Procedure 1)

Table S 28 Tabulation of accuracy and precision of THF dispensed, calibrated by weighing experiments

Density	0.889		24.0°C		bp:	66°C					
Dispensed volume (μL)	**REVERSE PIPETTING** aspiration speed = 1, dispensing speed = 1						Average weight (mg)	Std deviation	Relative Std dev	volume THF (μL)	Accuracy error (%)
	1	2	3	4							
10	8.72	8.95	8.76	9.17			8.90	0.21	2.32	10.01	0.09
20	18.39	18.02	17.60				18.00	0.40	2.20	20.25	1.22
30	27.17	27.00	27.07				27.08	0.09	0.32	30.45	1.49
40	35.19	36.26	36.08				35.84	0.57	1.60	40.31	0.77
50	45.40	45.37	45.50				45.42	0.07	0.15	51.08	2.12
60	52.12	54.32	54.26	53.03			53.43	1.06	1.98	60.09	0.15
70	62.92	63.90	62.26	62.85			62.98	0.68	1.08	70.83	1.17
80	72.80	72.37	72.51				72.56	0.22	0.30	81.60	1.96
90	82.98	82.64	82.22				82.61	0.38	0.46	92.91	3.13
100	92.96	91.99	92.02				92.32	0.55	0.60	103.83	3.69

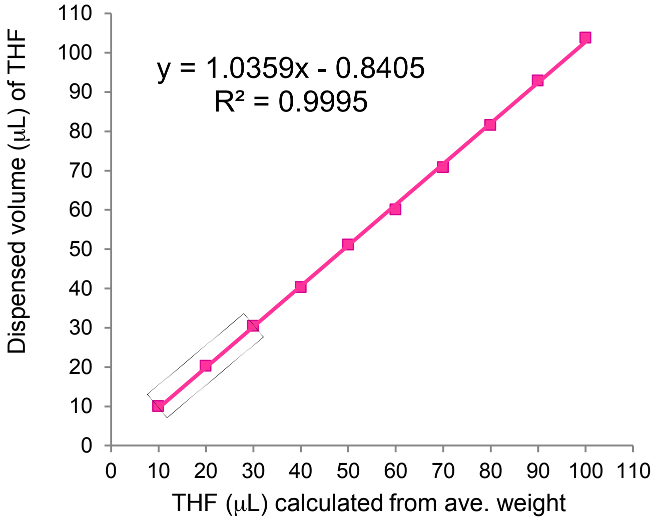

Figure S 40 Accuracy and precision of THF dispensed, calibrated by weighing experiments. Range 10-100μL THF

8.4.2 Experimental details and HPLC data (Procedure 1)

Table S 29 Experimental details of ball mill LAG experiments with **THF** as grinding solvent used to prepare the milling equilibrium curves and corresponding HPLC data. Tabulation of weights (mg) of starting materials (**1-1** & **2-2**), volume of **THF** added to the solid state DCC reaction and the corresponding grinding time used to ensure that thermodynamic equilibrium is achieved for each single experimental point. The chemical composition of the reaction product as %M obtained by HPLC analysis is tabulated. Details of HPLC method are included.

Ball mill LAG taken to thermodynamic equilibrium using Procedure 1

2%M dbu + µL **THF** added to 0.68 mmol **DCC** ([**1-1**] + [**2-2**] + [**1-2**])

Reagents:	**$(2NO_2PhS)_2$**	**$(4ClPhS)_2$**	THF added to powder		**HPLC conditions**		
MW:	308.33	287.23			Zorbax XDB C18, 1.8 µm; 4.6x50 mm		
%M initial	50%M	50%M	MW	Densiity	A: H_2O+0.1% FA; B: MeCN+0.1%FA		
mmol	0.34	0.34	(g/mol)	(g/mL)	0-2min 75-85%B;		
mg	104.8 mg	97.6 mg	72.11	0.8837	2ml/min; 60°C; 259 nm (8nm bandwidth)		
					**HPLC results**		
grinding time @ 30 Hz to equilibrium	$(2NO_2PhS)_2$ weighed (mg)	$(4ClPhS)_2$ weighed (mg)	THF µL	[THF]/[DCC] (mol/mol)	$(2NO_2PhS)_2$ **(1-1)** %M	Product **(1-2)** %M	$(4ClPhS)_2$ **(2-2)** %M
1h	104.83	97.65	**10**	**0.18**	0.6	**98.7**	0.7
1h	104.84	97.63	**15**	**0.27**	2.2	**95.5**	2.3
1h	104.82	97.68	**17**	**0.31**	2.5	**95.1**	2.3
1h	104.84	97.66	**18**	**0.32**	2.4	**94.7**	2.9
1h	104.85	97.67	**18**	**0.32**	4.0	**91.8**	4.2
1h30m	104.82	97.68	**19**	**0.34**	4.9	**90.8**	4.2
2h	104.86	97.68	**20**	**0.36**	7.6	**85.9**	6.4
1h	104.80	97.64	**21**	**0.38**	5.1	**90.4**	4.5
1h	104.82	97.66	**22**	**0.40**	4.9	**90.8**	4.4
1h	104.82	97.67	**23**	**0.41**	4.8	**90.6**	4.6
1h	104.84	97.66	**25**	**0.45**	4.4	**91.4**	4.2
1h	104.83	97.63	**30**	**0.54**	10.9	**79.6**	9.6

[DCC] is the concentration of the dynamic covalent chemistry reaction at any time =[1-1]+[2-2]+[1-2]

[1-2] = **Form A** + **Form B**

8.4.3 **PXRD scans, tabulation of Rietveld refinement of PXRD data and corresponding experimental milling equilibrium curves (Procedure 1)**

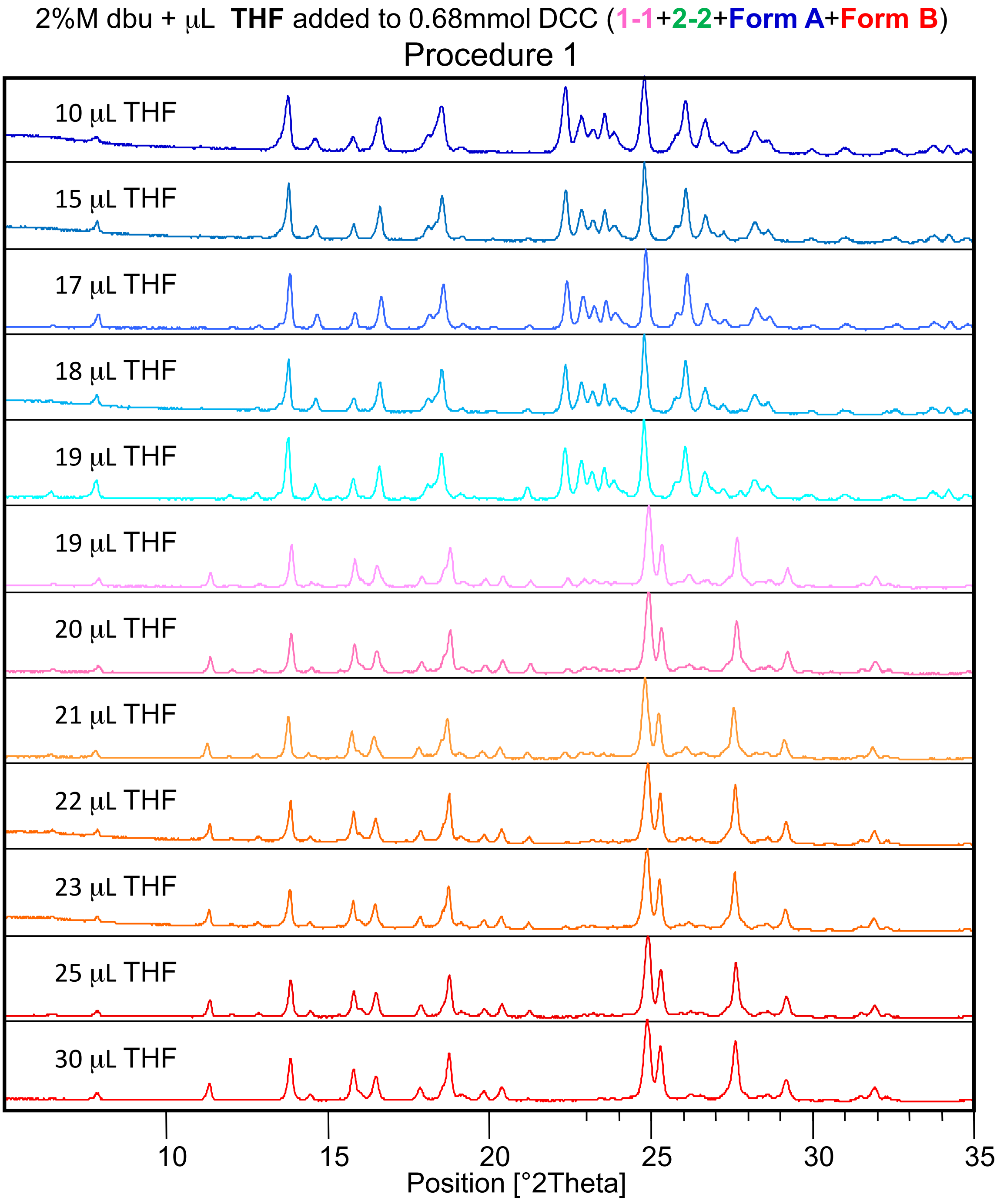

PXRD scan 7 Ball mill LAG reaction at equilibrium with different volumes of **THF**. Each PXRD scan was obtained at equilibrium from a single point experiment with the specified added volume of **THF**. The PXRD sample was prepared immediately after completion of the grinding experiment, and scanned between 5 and 45° (2θ).

Table S 30 Tabulation of the phase composition and corresponding R index used to prepare the milling equilibrium curve with **THF** as the LAG solvent using ball mill grinding Procedure 1. The phase composition was calculated by Rietveld refinement of PXRD data as %M of the mixture of starting materials (**1-1** and **2-2**) and the polymorphs of the product (**Form A** and **Form B**). The corresponding refinement errors (esd) have been calculated as %mol. The R index is calculated as the ratio between the concentration of **Form B** versus the total concentration of **Form B** plus **Form A**.

Ball mill LAG grinding reaction taken to thermodynamic equilibrium using Procedure 1												
2%M dbu + μL THF added to 0.68 mmol DCC ([**1-1**] + [**2-2**] + [**1-2**])												
grinding time @ 30Hz to equilib.	THF added to powder		**1-1**		**2-2**		**Form B**		**Form A**		[A]+[B]	**R =** [B] / ([A]+[B])
	THF μL	[THF]/[DCC] (mol/mol)	%M	e.s.d. mol%	%M	e.s.d. mol%	%M	e.s.d. mol%	%M	e.s.d. mol%	total polymorph	% R
1h	**10**	**0.18**	**1.7**	0.3	**1.4**	0.2	**3.3**	0.4	**93.6**	0.5	96.9	**3.4**
1h	**15**	**0.27**	**2.4**	0.3	**1.9**	0.3	**3.1**	0.4	**92.6**	0.6	95.7	**3.2**
1h	**17**	**0.31**	**3.8**	0.2	**3.0**	0.2	**3.5**	0.4	**89.7**	0.4	93.2	**3.8**
1h	**18**	**0.32**	**3.0**	0.3	**2.4**	0.3	**3.1**	0.5	**91.5**	0.6	94.6	**3.3**
1h	**18**	**0.32**	**7.3**	0.3	**5.9**	0.3	**4.2**	0.4	**82.5**	0.5	86.7	**4.8**
1h30m	**19**	**0.34**	**4.2**	0.2	**3.4**	0.2	**73.8**	0.4	**18.6**	0.4	92.4	**79.9**
2h	**20**	**0.36**	**7.5**	0.3	**6.0**	0.2	**77.9**	0.5	**8.6**	0.4	86.5	**90.1**
1h	**21**	**0.38**	**5.4**	0.3	**4.3**	0.2	**75.5**	0.5	**14.8**	0.4	90.3	**83.6**
1h	**22**	**0.40**	**5.8**	0.3	**4.6**	0.3	**86.7**	0.6	**2.9**	0.5	89.6	**96.8**
1h	**23**	**0.41**	**5.6**	0.3	**4.5**	0.3	**84.1**	0.5	**5.8**	0.4	89.9	**93.5**
1h	**25**	**0.45**	**5.7**	0.3	**4.6**	0.2	**89.1**	0.4	**0.6**	0.2	89.7	**99.3**
1h	**30**	**0.54**	**2.3**	0.3	**1.8**	0.3	**94.5**	0.5	**1.4**	0.3	95.9	**98.5**

[DCC] is the concentration of the dynamic covalent chemistry reaction at any time =[1-1]+[2-2]+[1-2]

[1-2] = **Form A** + **Form B**

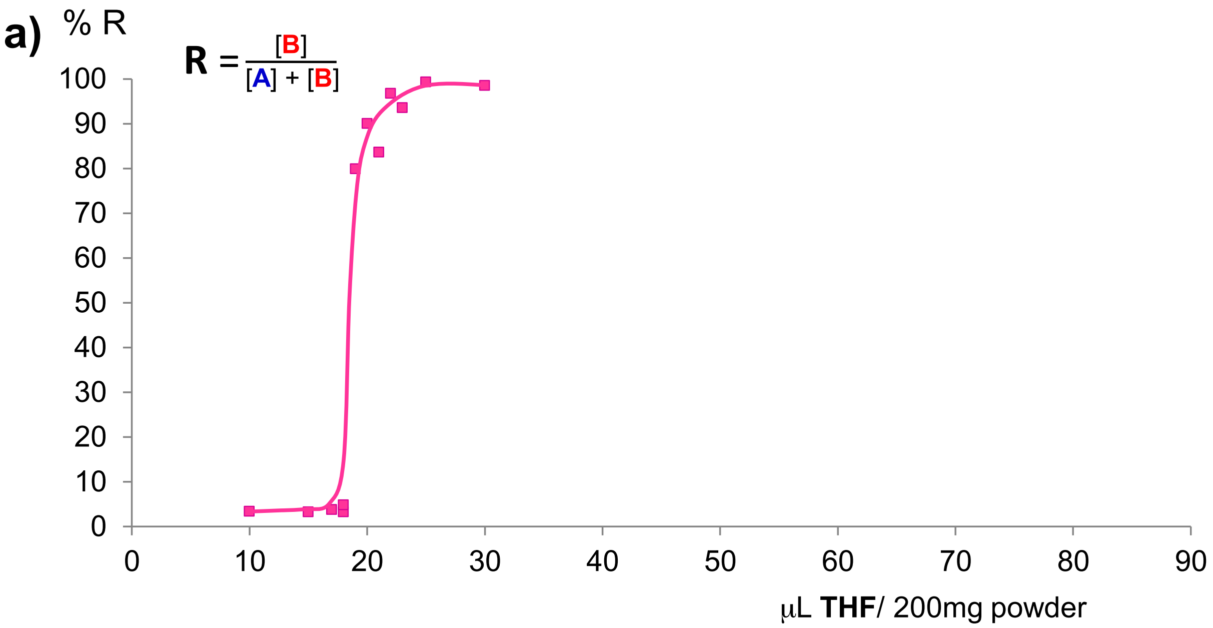

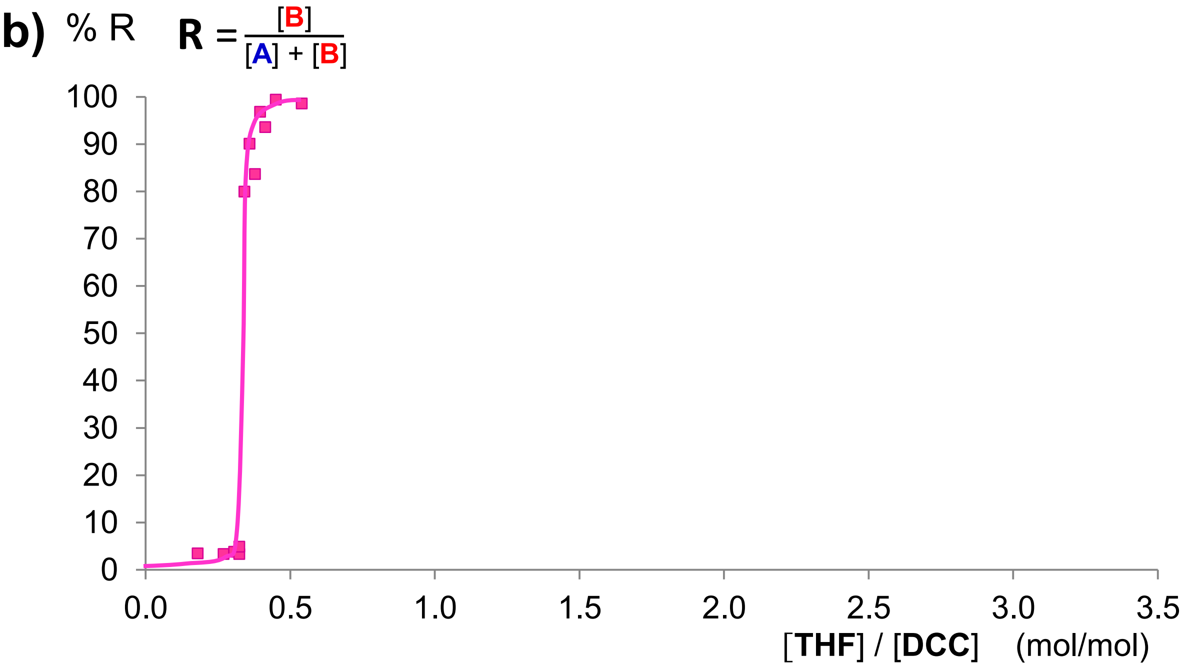

[**DCC**] is the concentration of the dynamic covalent chemistry reaction at any time = [**1-1**]+[**2-2**]+[**A**]+[**B**]
[**A**] is the concentration of **Form A** in DCC; [**B**] is the concentration of **Form B** in DCC

Figure S 41 Experimental milling equilibrium curves using “ball mill grinding procedure 1” plotted as the concentration of THF versus %R index. R is the ratio of the concentration of **Form B** with respect to the total concentration of **Form B** plus **Form A** formed in the solid state DCC reaction for each specific experimental point. **a)** Curve expressed as μL THF / 200 mg powder and **b)** curve expressed as mols of THF added to the reaction with respect to 0.68 mmol of DCC library; this library is composed by the homodimers **1-1** and **2-2** and the heterodimers **Form A** and **Form B**. R is calculated from the Rietveld refinement of PXRD data giving the phase composition of the solid-state DCC experiment at equilibrium as %M. Each point in the graph is an individual experiment. No fitting was performed and the curves drawn are only a guide to the eye.

8.5 LAG experiments using dimethylformamide as grinding liquid on solid-state DCC reaction.

These experiments were performed by adding the solvent by reverse pipetting using Procedure 1 (Section 4.5.1).

8.5.1 Accuracy and precision of dispensing DMF (Procedure 1)

Table S 31 Tabulation of accuracy and precision of DMF dispensed, calibrated by weighing experiments

Density:	0.940		24.0°C		bp:	56°C					
Dispensed volume (μL)	REVERSE PIPETTING aspiration speed = 1, dispensing speed = 1						Average weight (mg)	Std deviation	Relative Std dev	volume DMF (μL)	Accuracy error (%)
	1	2	3	4	5	6					
13	12.16	12.10	12.31	13.47	11.80	12.45	12.38	0.58	4.66	13.17	1.31
14	13.28	13.24	13.20	11.99	12.20	12.2	12.69	0.61	4.83	13.49	-3.74
15	14.22	14.28	14.15	13.38	12.87	12.84	13.62	0.68	4.98	14.49	-3.50
16	15.22	15.12	15.08	13.80			14.81	0.67	4.54	15.75	-1.59
17	16.17	16.05	16.22	15.13			15.89	0.51	3.23	16.91	-0.55
18	17.24	17.19	17.04	15.53			16.75	0.82	4.88	17.82	-1.01
19	17.98	18.16	18.01	15.88			17.51	1.09	6.21	18.63	-2.01
20	19.11	18.92	18.92	16.74	17.04	17.2	17.99	1.10	6.13	19.14	-4.51
21	20.09	19.79	20.02	17.39	17.33		18.92	1.43	7.57	20.13	-4.31
22	20.81	20.68	20.66	18.85	17.49		19.70	1.48	7.50	20.96	-4.99
23	21.89	21.55	21.69	19.13			21.07	1.30	6.16	22.41	-2.63

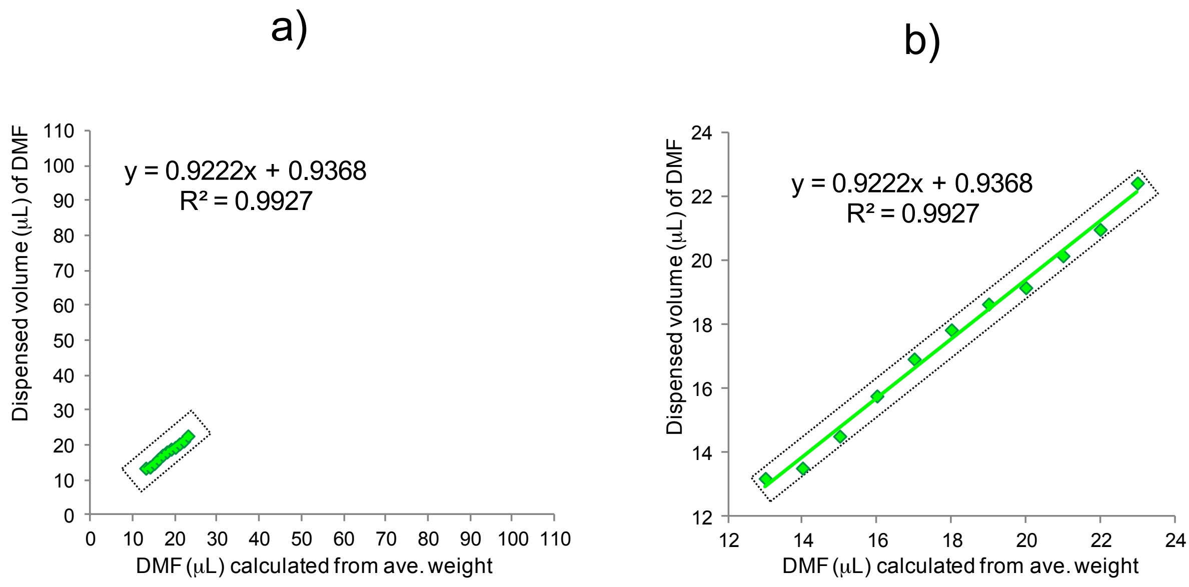

Figure S 42 Accuracy and precision of DMF dispensed, calibrated by weighing experiments.
a) on a range 0-100 μL DMF b) on an narrower expanded range from 13-23 μL DMF

8.5.2 Experimental details and HPLC data (Procedure 1)

Table S 32 Experimental details of ball mill LAG experiments with **DMF** as grinding solvent used to prepare the milling equilibrium curves and corresponding HPLC data. Tabulation of weights (mg) of starting materials (**1-1** & **2-2**), volume of **DMF** added to the solid state DCC reaction and the corresponding grinding time used to ensure that thermodynamic equilibrium is achieved for each single experimental point. The chemical composition of the reaction product as %M obtained by HPLC analysis is tabulated. Details of HPLC method are included.

Ball mill LAG taken to thermodynamic equilibrium using Procedure 1

2%M dbu + µL **DMF** added to 0.68 mmol **DCC** ([**1-1**] + [**2-2**] + [**1-2**])

Reagents:	**$(2NO_2PhS)_2$**	**$(4ClPhS)_2$**	DMF added to powder		HPLC conditions: Zorbax XDB C18, 1.8 µm; 4.6x50 mm; A: H_2O+0.1% FA; B: MeCN+0.1%FA; 0-2min 75-85%B; 2ml/min; 60°C; 259 nm (8nm bandwidth)		
MW:	308.33	287.23					
%M initial	50%M	50%M	MW (g/mol)	Densiity (g/mL)			
mmol	0.34	0.34					
mg	104.8 mg	97.6 mg	59.07	0.9995			
					**HPLC results**		
grinding time @ 30 Hz to equilibrium	$(2NO_2PhS)_2$ weighed (mg)	$(4ClPhS)_2$ weighed (mg)	DMF µL	[DMF]/[DCC] (mol/mol)	$(2NO_2PhS)_2$ **(1-1)** %M	Product **(1-2)** %M	$(4ClPhS)_2$ **(2-2)** %M
2h30m	104.81	97.66	**5**	**0.12**	0.8	**98.6**	0.6
3h	104.86	97.71	**10**	**0.25**	1.2	**97.4**	1.4
3h	104.83	97.64	**13**	**0.32**	1.4	**97.6**	1.0
2h30m	104.81	97.69	**14**	**0.35**	0.9	**98.2**	0.9
2h	104.85	97.70	**15**	**0.37**	1.3	**97.5**	1.1
3h	104.83	97.64	**16**	**0.40**	7.1	**86.6**	6.3
2h	104.84	97.66	**16**	**0.40**	2.1	**95.9**	2.0
3h	104.85	97.72	**17**	**0.42**	2.7	**95.2**	2.1
2h	104.82	97.68	**18**	**0.45**	4.8	**90.8**	4.2
3h	104.86	97.63	**19**	**0.47**	2.4	**95.7**	1.9
3h	104.87	97.63	**20**	**0.50**	2	**96.5**	1.4
3h	104.83	97.64	**21**	**0.52**	1.6	**97.2**	1.3
2h30m	104.84	97.65	**22**	**0.55**	2	**96.4**	1.5
3h	104.87	97.66	**25**	**0.62**	1.1	**97.9**	1
2h	104.84	97.67	**28**	**0.70**	2.2	**96.1**	1.8
2h	104.83	97.66	**30**	**0.75**	1.7	**96.9**	1.4
2h30m	104.83	97.69	**50**	**1.24**	1.3	**98.1**	0.6

[DCC] is the concentration of the dynamic covalent chemistry reaction at any time =[1-1]+[2-2]+[1-2]

[1-2] = **Form A** + **Form B**

8.5.3 **PXRD scans, tabulation of Rietveld refinement of PXRD data and corresponding experimental milling equilibrium curves (Procedure 1)**

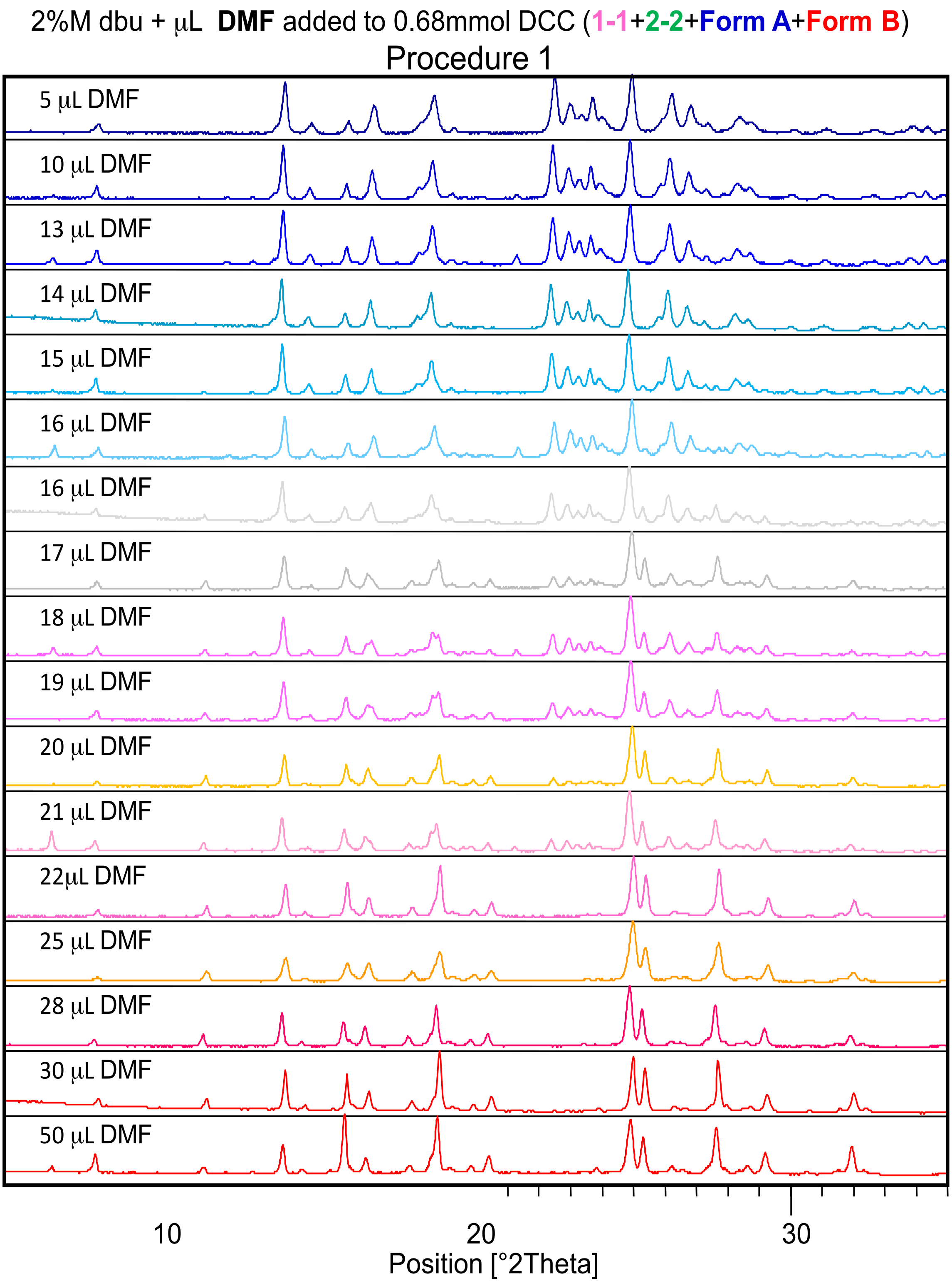

PXRD scan 8 Ball mill LAG reaction at equilibrium with different volumes of **DMF**. Each PXRD scan was obtained at equilibrium from a single point experiment with the specified added volume of **DMF**. The PXRD sample was prepared immediately after completion of the grinding experiment, and scanned between 5 and 45° (2θ).

Table S 33 Tabulation of the phase composition and corresponding R index used to prepare the milling equilibrium curve with **DMF** as the LAG solvent using ball mill grinding **Procedure 1**. The phase composition was calculated by Rietveld refinement of PXRD data as %M of the mixture of starting materials (**1-1** and **2-2**) and the polymorphs of the product (**Form A** and **Form B**). The corresponding refinement errors (esd) have been calculated as %mol. The R index is calculated as the ratio between the concentration of **Form B** versus the total concentration of **Form B** plus **Form A**.

Ball mill LAG grinding reaction taken to thermodynamic equilibrium using Procedure 1												
2%M dbu+μL DMF added to 0.68 mmol DCC ([1-1]+[2-2]+[Form A]+[Form B])												
grinding time @ 30Hz to equilib.	DMF added to powder		1-1		2-2		Form B		Form A		[A]+[B]	R = [B] / ([A]+[B])
	DMF μL	[DMF]/[DCC] (mol/mol)	%M	e.s.d. mol%	%M	e.s.d. mol%	%M	e.s.d. mol%	%M	e.s.d. mol%	total polymorph	% R
2h30m	**5**	**0.12**	**1.5**	0.3	**1.2**	0.2	**4.7**	0.6	**92.6**	0.7	97.3	**4.8**
3h	**10**	**0.25**	**3.0**	0.3	**2.4**	0.3	**1.9**	0.3	**92.7**	0.5	94.6	**2.0**
3h	**13**	**0.32**	**6.3**	0.3	**5.1**	0.2	**3.8**	0.5	**84.9**	0.5	88.7	**4.3**
2h30m	**14**	**0.35**	**1.0**	0.2	**0.8**	0.2	**3.0**	0.4	**95.3**	0.5	98.3	**3.1**
2h	**15**	**0.37**	**1.8**	0.3	**1.4**	0.3	**13.1**	0.6	**83.7**	0.6	96.8	**13.5**
3h	**16**	**0.40**	**8.8**	0.3	**7.1**	0.3	**13.0**	0.5	**71.1**	0.5	84.1	**15.5**
2h	**16**	**0.40**	**1.8**	0.4	**1.5**	0.3	**30.2**	0.5	**66.5**	0.5	96.7	**31.2**
3h	**17**	**0.42**	**0.8**	0.2	**0.7**	0.2	**67.4**	0.6	**31.1**	0.6	98.5	**68.4**
2h	**18**	**0.45**	**5.9**	0.3	**4.8**	0.2	**39.8**	0.5	**49.5**	0.5	89.3	**44.6**
3h	**19**	**0.47**	**1.6**	0.3	**1.3**	0.3	**54.9**	0.6	**42.1**	0.5	97.0	**56.6**
3h	**20**	**0.50**	**1.5**	0.3	**1.2**	0.2	**78.4**	0.5	**19.0**	0.4	97.4	**80.5**
3h	**21**	**0.52**	**6.5**	0.3	**5.2**	0.2	**59.7**	0.4	**28.6**	0.4	88.3	**67.6**
2h30m	**22**	**0.55**	**1.0**	0.3	**0.8**	0.2	**97.4**	0.4	**0.8**	0.3	98.2	**99.2**
3h	**25**	**0.62**	**1.2**	0.3	**1.0**	0.3	**93.7**	0.6	**4.0**	0.5	97.7	**95.9**
2h	**28**	**0.70**	**0.9**	0.5	**0.7**	0.4	**96.7**	0.8	**1.6**	0.6	98.3	**98.4**
2h	**30**	**0.75**	**1.7**	0.2	**1.4**	0.2	**96.5**	0.5	**0.4**	0.4	96.9	**99.6**
2h30m	**50**	**1.24**	**3.0**	0.3	**2.4**	0.2	**93.9**	0.5	**0.6**	0.4	94.5	**99.4**
[DCC] is the concentration of the dynamic covalent chemistry reaction at any time =[1-1]+[2-2]+[Form A]+[Form B]												
[**1-2**] = **Form A** + **Form B** [A] ≡ [**Form A**] [B] ≡ [**Form B**]												

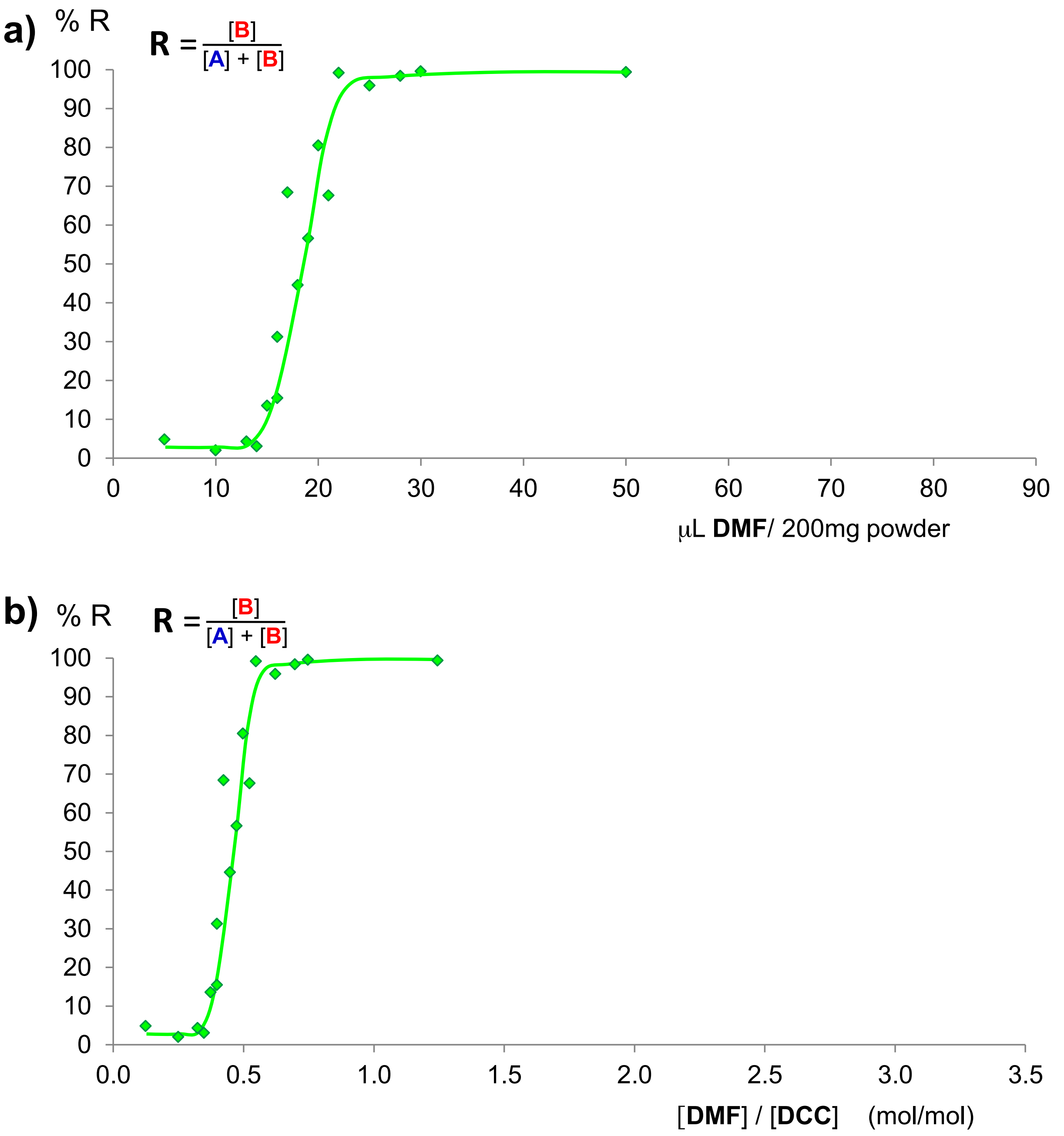

[**DCC**] is the concentration of the dynamic covalent chemistry reaction at any time = [**1-1**]+[**2-2**]+[**A**]+[**B**]
[**A**] is the concentration of **Form A** in DCC; [**B**] is the concentration of **Form B** in DCC

Figure S 43 Experimental milling equilibrium curves using “ball mill grinding procedure 1” plotted as the concentration of DMF versus %R index. R is the ratio of the concentration of **Form B** with respect to the total concentration of **Form B** plus **Form A** formed in the solid state DCC reaction for each specific experimental point. **a)** Curve expressed as μL DMF / 200 mg powder and **b)** curve expressed as mols of DMF added to the reaction with respect to 0.68 mmol of DCC library; this library is composed by the homodimers **1-1** and **2-2** and the heterodimers **Form A** and **Form B**. R is calculated from the Rietveld refinement of PXRD data giving the phase composition of the solid-state DCC experiment at equilibrium as %M. Each point in the graph is an individual experiment. No fitting was performed and the curves drawn are only a guide to the eye.

8.6 LAG experiments using ethyl acetate as grinding liquid on solid-state DCC reaction.

These experiments were performed by adding the solvent by reverse pipetting using Procedure 1 (Section 4.5.1).

8.6.1 Accuracy and precision of dispensing ethyl acetate (Procedure 1)

Table S 34 Tabulation of accuracy and precision of ethyl acetate dispensed, calibrated by weighing experiments

Density	0.895		24.0°C		bp:	77.1°C					
Dispensed volume (μL)	REVERSE PIPETTING aspiration speed = 1, dispensing speed = 1						Average weight (mg)	Std deviation	Relative Std dev	volume EtOAc (μL)	Accuracy error (%)
	1	2	3								
10	8.54	8.75	8.39				8.56	0.18	2.11	9.57	-4.50
20	18.13	17.60	18.13				17.95	0.31	1.70	20.07	0.35
30	27.04	26.93	26.93				26.97	0.06	0.24	30.15	0.49
40	36.25	36.05	36.76				36.35	0.37	1.01	40.64	1.58
50	45.58	45.51	45.48				45.52	0.05	0.11	50.89	1.75
60	54.29	54.48	54.38				54.38	0.10	0.17	60.80	1.31
70	61.68	63.02	63.10				62.60	0.80	1.27	69.98	-0.02
80	72.73	72.78	72.92				72.81	0.10	0.14	81.40	1.72
90	82.03	82.09	82.02				82.05	0.04	0.05	91.72	1.88
100	91.88	91.74	91.48				91.70	0.20	0.22	102.52	2.45

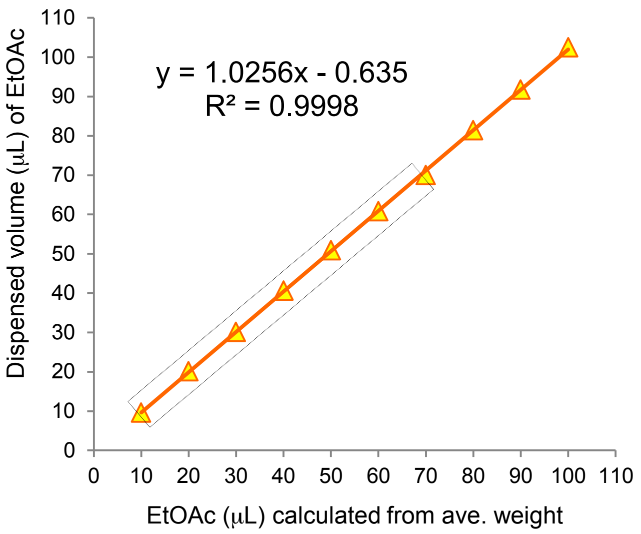

Figure S 44 Accuracy and precision of EtOAc dispensed, calibrated by weighing experiments.

8.6.2 Experimental details and HPLC data (Procedure 1)

Table S 35 Experimental details of ball mill LAG experiments with **EtOAc** as grinding solvent used to prepare the milling equilibrium curves and corresponding HPLC data. Tabulation of weights (mg) of starting materials (**1-1** & **2-2**), volume of **EtOAc** added to the solid state DCC reaction and the corresponding grinding time used to ensure that thermodynamic equilibrium is achieved for each single experimental point. The chemical composition of the reaction product as %M obtained by HPLC analysis is tabulated. Details of HPLC method are included.

Ball mill LAG taken to thermodynamic equilibrium using Procedure 1

2%M dbu + µL **EtOAc** added to 0.68 mmol **DCC** ([**1-1**] + [**2-2**] + [**1-2**])

Reagents:	**$(2NO_2PhS)_2$**	**$(4ClPhS)_2$**	EtOAc added to powder		**HPLC conditions**		
MW:	308.33	287.23			Zorbax XDB C18, 1.8 µm; 4.6x50 mm		
%M initial	50%M	50%M	MW	Densiity	A: H_2O+0.1% FA; B: MeCN+0.1%FA		
mmol	0.34	0.34	(g/mol)	(g/mL)	0-2min 75-85%B;		
mg	104.8 mg	97.6 mg	88.11	0.8945	2ml/min; 60°C; 259 nm (8nm bandwidth)		
					**HPLC results**		
grinding time @ 30 Hz to equilibrium	$(2NO_2PhS)_2$ weighed (mg)	$(4ClPhS)_2$ weighed (mg)	EtOAc µL	[EtOAc]/[DCC] (mol/mol)	$(2NO_2PhS)_2$ (1-1) %M	Product **(1-2)** %M	$(4ClPhS)_2$ (2-2) %M
3h	104.85	97.69	**10**	**0.15**	0.6	**98.7**	0.7
2h	104.81	97.64	**18**	**0.27**	2.6	**94.9**	2.5
4h	104.90	97.67	**20**	**0.30**	3.8	**92.7**	3.4
3h30m	104.83	97.69	**22**	**0.33**	5.2	**90.2**	4.6
3h30m	104.82	97.68	**23**	**0.34**	5.1	**90.4**	4.6
4h	104.80	97.69	**25**	**0.37**	6.8	**86.8**	6.4
3h	104.80	97.66	**26**	**0.39**	5	**90.6**	4.5
3h	104.78	97.63	**27**	**0.40**	5	**90.8**	4.2
3h	104.85	97.65	**28**	**0.42**	3.6	**93.7**	2.7
4h	104.87	97.74	**30**	**0.45**	4.4	**92.2**	3.5
2h	104.81	97.65	**31**	**0.46**	5.6	**89.6**	4.8
3h	104.82	97.66	**40**	**0.60**	5.8	**89.1**	5.1
3h	104.87	97.65	**50**	**0.75**	5.2	**91.2**	3.6
2h30m	104.84	97.67	**60**	**0.90**	6.8	**87.5**	5.7
2h	104.85	97.65	**70**	**1.05**	8	**84.9**	7.2

[DCC] is the concentration of the dynamic covalent chemistry reaction at any time =[1-1]+[2-2]+[1-2]

[1-2] = **Form A** + **Form B**

8.6.3 PXRD scans, tabulation of Rietveld refinement of PXRD data and corresponding experimental milling equilibrium curves (Procedure 1)

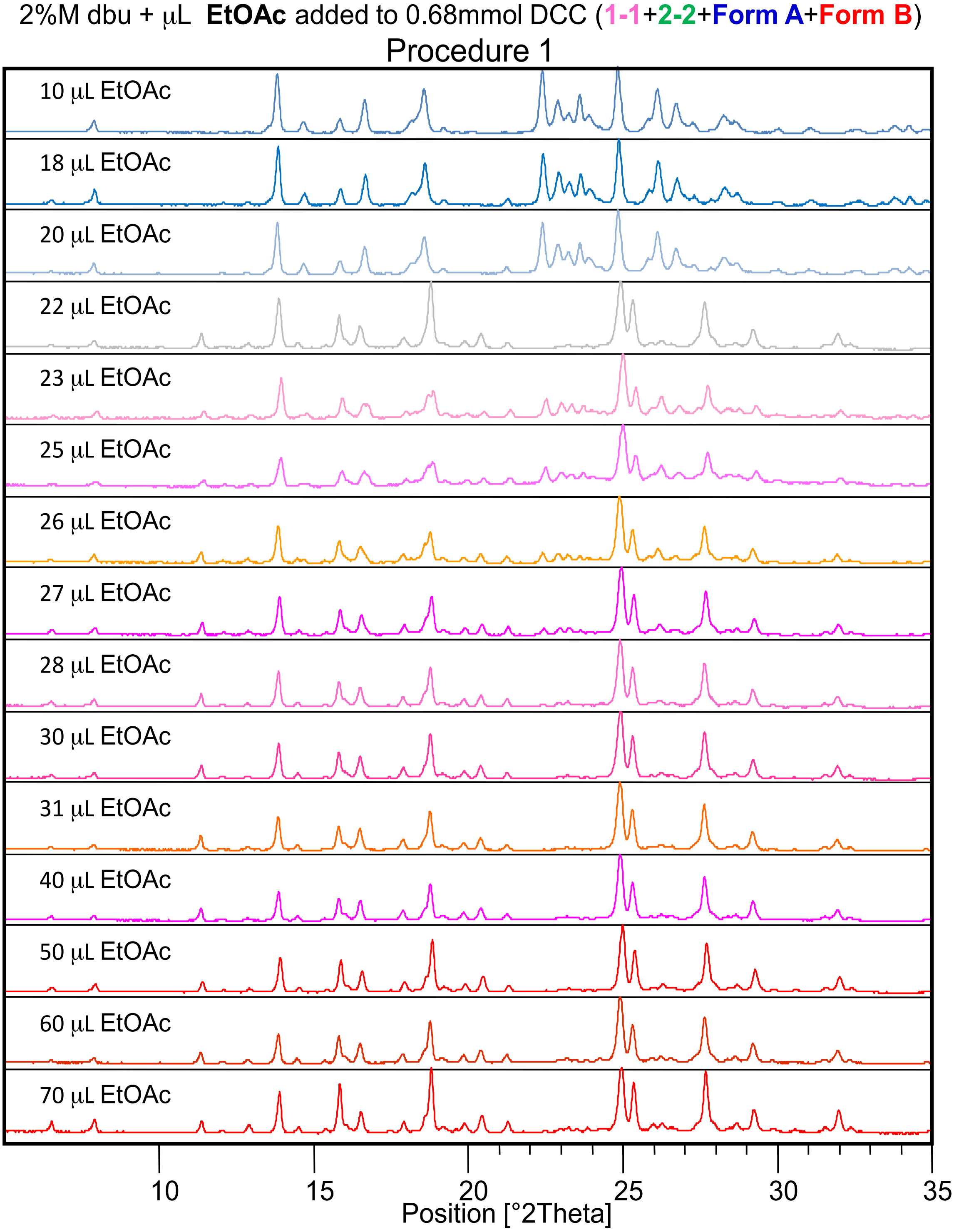

PXRD scan 9 Ball mill LAG reaction at equilibrium with different volumes of **EtOAc**. Each PXRD scan was obtained at equilibrium from a single point experiment with the specified added volume of **EtOAc**. The PXRD sample was prepared immediately after completion of the grinding experiment, and scanned between 5 and 45° (2θ).

Table S 36 Tabulation of the phase composition and corresponding R index used to prepare the milling equilibrium curve with **EtOAc** as the LAG solvent using ball mill grinding **Procedure 1**. The phase composition was calculated by Rietveld refinement of PXRD data as %M of the mixture of starting materials (**1-1** and **2-2**) and the polymorphs of the product (**Form A** and **Form B**). The corresponding refinement errors (esd) have been calculated as %mol. The R index is calculated as the ratio between the concentration of **Form B** versus the total concentration of **Form B** plus **Form A**.

Ball mill LAG grinding reaction taken to thermodynamic equilibrium using Procedure 1

2%M dbu+μL EtOAc added to 0.68 mmol DCC ([**1-1**]+[**2-2**]+[**Form A**]+[**Form B**])

grinding time @ 30Hz to equilib.	EtOAc added to powder		**1-1**		**2-2**		**Form B**		**Form A**		[A]+[B]	R = [B] / ([A]+[B])
	EtOAc μL	[EtOAc]/[DCC] (mol/mol)	%M	e.s.d. mol%	%M	e.s.d. mol%	%M	e.s.d. mol%	%M	e.s.d. mol%	total polymorph	% R
3h	**10**	**0.15**	**1.5**	0.3	**1.2**	0.3	**3.3**	0.5	**94.0**	0.6	97.3	**3.4**
2h	**18**	**0.27**	**4.6**	0.3	**3.7**	0.2	**3.8**	0.4	**87.9**	0.5	91.7	**4.1**
4h	**20**	**0.30**	**4.3**	0.3	**3.5**	0.2	**3.4**	0.4	**88.8**	0.5	92.2	**3.7**
3h30m	**22**	**0.33**	**5.9**	**0.3**	4.7	**0.2**	**87.7**	0.4	**1.6**	0.3	89.3	**98.2**
3h30m	**23**	**0.34**	**7.5**	0.4	**6.1**	0.3	**47.7**	0.6	**38.8**	0.6	86.5	**55.1**
4h	**25**	**0.37**	**6.3**	0.4	**5.1**	0.3	**52.0**	0.6	**36.6**	0.6	88.6	**58.7**
3h	**26**	**0.39**	**6.5**	0.3	**5.2**	0.3	**63.8**	0.5	**24.5**	0.4	88.3	**72.3**
3h	**27**	**0.40**	**7.5**	0.3	**6.0**	0.2	**72.3**	0.5	**14.1**	0.4	86.4	**83.7**
3h	**28**	**0.42**	**7.7**	0.3	**6.2**	0.2	**79.6**	0.4	**6.4**	0.4	86.0	**92.6**
4h	**30**	**0.45**	**6.1**	0.3	**4.9**	0.3	**87.9**	0.5	**1.0**	0.3	88.9	**98.9**
2h	**31**	**0.46**	**5.9**	0.3	**4.7**	0.2	**88.1**	0.4	**1.3**	0.3	89.4	**98.5**
3h	**40**	**0.60**	**6.4**	0.3	**5.2**	0.2	**86.8**	0.4	**1.6**	0.3	88.4	**98.2**
3h	**50**	**0.75**	**6.6**	0.2	**5.3**	0.2	**87.1**	0.3	**1.1**	0.2	88.2	**98.8**
2h30m	**60**	**0.90**	**7.4**	0.3	**6.0**	0.3	**85.0**	0.6	**1.6**	0.5	86.6	**98.2**
2h	**70**	**1.05**	**9.3**	0.3	**7.5**	0.2	**82.0**	0.5	**1.2**	0.4	83.2	**98.6**

[DCC] is the concentration of the dynamic covalent chemistry reaction at any time =[**1-1**]+[**2-2**]+[**Form A**]+[**Form B**]

[**1-2**] = **Form A** + **Form B** [A] ≡ [**Form A**] [B] ≡ [**Form B**]

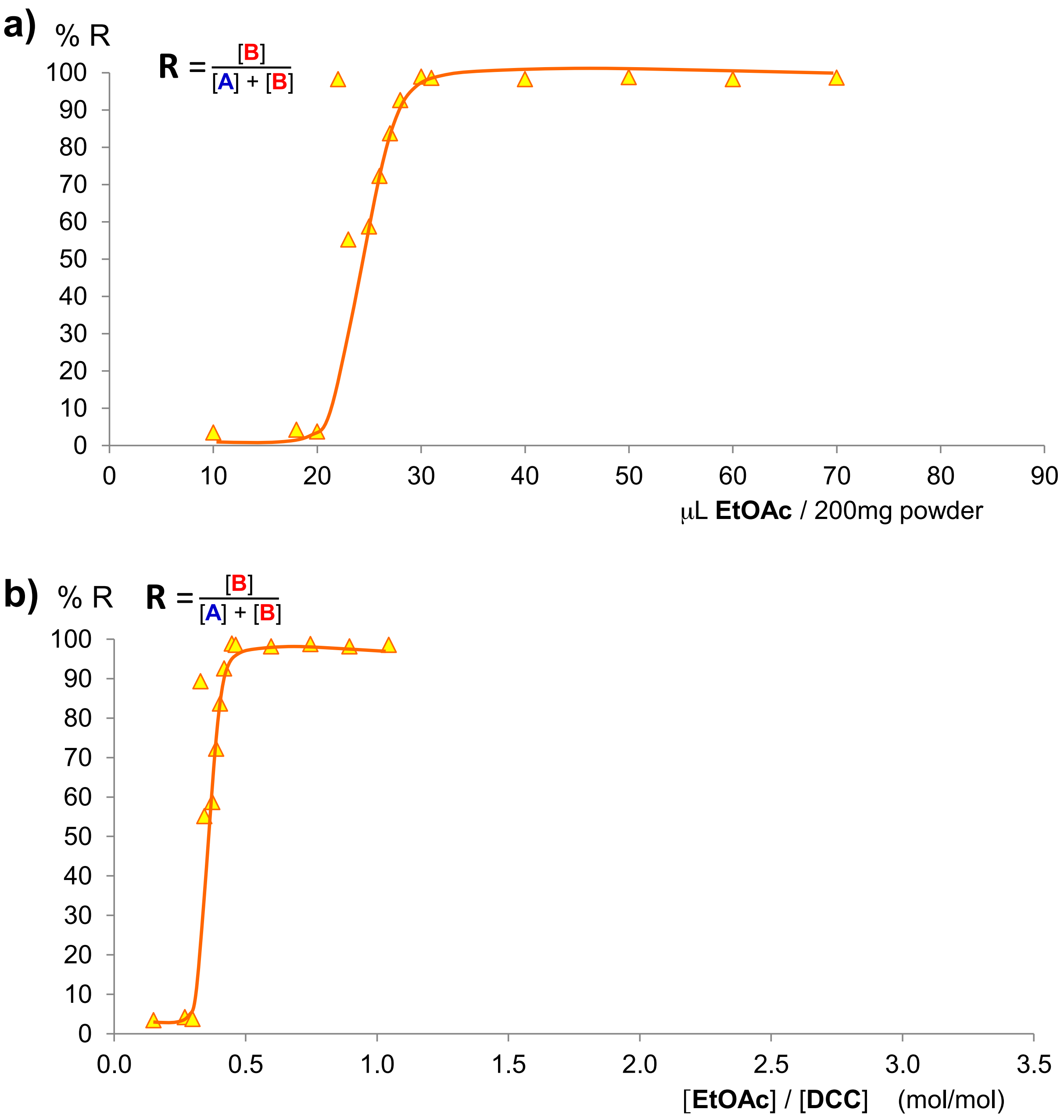

[**DCC**] is the concentration of the dynamic covalent chemistry reaction at any time = [**1-1**]+[**2-2**]+[**A**]+[**B**]
[**A**] is the concentration of **Form A** in DCC; [**B**] is the concentration of **Form B** in DCC

Figure S 45 Experimental milling equilibrium curves using "ball mill grinding procedure 1" plotted as the concentration of EtOAc versus %R index. R is the ratio of the concentration of **Form B** with respect to the total concentration of **Form B** plus **Form A** formed in the solid state DCC reaction for each specific experimental point. **a)** Curve expressed as μL EtOAc / 200 mg powder and **b)** curve expressed as mols of EtOAc added to the reaction with respect to 0.68 mmol of DCC library; this library is composed by the homodimers **1-1** and **2-2** and the heterodimers **Form A** and **Form B**. R is calculated from the Rietveld refinement of PXRD data giving the phase composition of the solid-state DCC experiment at equilibrium as %M. Each point in the graph is an individual experiment. No fitting was performed and the curves drawn are only a guide to the eye.

8.7 LAG experiments using Chloroform as grinding liquid on solid-state DCC reaction.

These experiments were performed by adding the solvent by reverse pipetting using Procedure 1 (Section 4.5.1).

8.7.1 Accuracy and precision of dispensing chloroform

Table S 37 Tabulation of accuracy and precision of chloroform dispensed, calibrated by weighing experiments

Density	1.4793		24.0°C		bp:	61.2°C					
Dispensed volume (μL)	REVERSE PIPETTING aspiration speed = 1, dispensing speed = 1						Average weight (mg)	Std deviation	Relative Std dev	volume $CHCl_3$ (μL)	Accuracy error (%)
	1	2	3	4	5	6					
10	15.53	14.20	14.11	14.94			14.70	0.67	4.56	9.93	-0.67
20	32.44	30.97	31.23				31.55	0.78	2.49	21.33	6.22
30	48.00	46.60	46.90	46.78			47.07	0.63	1.34	31.82	5.72
40	62.70	61.17	62.20				62.02	0.78	1.26	41.93	4.60
50	78.34	77.21	77.55	77.43	77.45	77.06	77.51	0.45	0.57	52.39	4.57
60	92.07	92.48	92.93	92.36			92.46	0.36	0.39	62.50	4.00
70	110.16	109.17	110.18				109.84	0.58	0.53	74.25	5.72
80	125.82	127.54	127.16	126.90			126.86	0.74	0.58	85.75	6.71
90	142.36	140.76	140.38	140.50			141.00	0.92	0.65	95.32	5.58
100	157.70	157.10	156.28				157.03	0.71	0.45	106.15	5.79

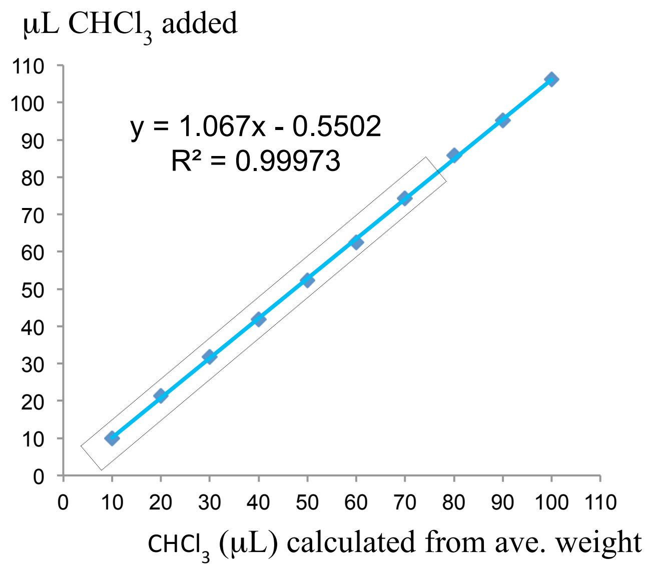

Figure S 46 Accuracy and precision of $CHCl_3$ dispensed, calibrated by weighing experiments. Range 10-100μL $CHCl_3$

8.7.2 Experimental details and HPLC data (Procedure 1)

Table S 38 Experimental details of ball mill LAG experiments with **$CHCl_3$** as grinding solvent used to prepare the milling equilibrium curves and corresponding HPLC data. Tabulation of weights (mg) of starting materials (**1-1** & **2-2**), volume of **$CHCl_3$** added to the solid state DCC reaction and the corresponding grinding time used to ensure that thermodynamic equilibrium is achieved for each single experimental point. The chemical composition of the reaction product as %M obtained by HPLC analysis is tabulated. Details of HPLC method are included.

Ball mill LAG taken to thermodynamic equilibrium using Procedure 1							
2%M dbu + **μL $CHCl_3$** added to 0.68 mmol **DCC** ([**1-1**] + [**2-2**] + [**1-2**])							
Reagents:	**$(2NO_2PhS)_2$**	**$(4ClPhS)_2$**	$CHCl_3$ added to powder		**HPLC conditions**		
MW:	308.33	287.23			Zorbax XDB C18, 1.8 μm; 4.6x50 mm		
%M initial	50%M	50%M	MW	Densiity	A: H_2O+0.1% FA; B: MeCN+0.1%FA		
mmol	0.34	0.34	(g/mol)	(g/mL)	0-2min 75-85%B;		
mg	104.8 mg	97.6 mg	119.30	1.4793	2ml/min; 60°C; 259 nm (8nm bandwidth)		
					**HPLC results**		
grinding time @ 30 Hz to equilibrium	$(2NO_2PhS)_2$ weighed (mg)	$(4ClPhS)_2$ weighed (mg)	$CHCl_3$ μL	$[CHCl_3]/[DCC]$ (mol/mol)	$(2NO_2PhS)_2$ (1-1) %M	Product **(1-2)** %M	$(4ClPhS)_2$ (2-2) %M
2h30m	104.84	97.68	**5**	**0.09**	0.7	**98.9**	0.4
2h30m	104.84	97.69	**10**	**0.18**	1.0	**98.2**	0.8
2h	104.81	97.65	**13**	**0.24**	0.7	**98.2**	1.1
1h15m	104.81	97.70	**15**	**0.27**	8.2	**84.8**	7.0
2h	104.80	97.70	**16**	**0.29**	1.1	**98.0**	0.9
1h	104.85	97.64	**17**	**0.31**	3.8	**92.9**	3.3
1h30m	104.86	97.65	**18**	**0.33**	3.5	**93.3**	3.3
2h	104.81	97.65	**19**	**0.35**	3.6	**93.0**	3.4
45m	104.82	97.69	**20**	**0.36**	5.2	**90.5**	4.2
2h	104.80	97.64	**20**	**0.36**	4.5	**92.0**	3.5
2h	104.84	97.67	**21**	**0.38**	3.6	**92.7**	3.7
2h	104.84	97.67	**22**	**0.40**	3.7	**92.9**	3.4
2h30m	104.81	97.66	**23**	**0.42**	3.2	**93.8**	3.0
2h	104.83	97.66	**24**	**0.44**	5.1	**90.6**	4.3
2h	104.84	97.70	**25**	**0.46**	3.3	**94.0**	2.7
2h	104.84	97.67	**27**	**0.49**	5.7	**90.8**	4.2
2h	104.82	97.67	**30**	**0.55**	4.2	**92.5**	3.3
3h	104.81	97.68	**32**	**0.58**	4.2	**92.7**	3.1
3h30m	104.80	97.67	**35**	**0.64**	6.8	**87.9**	5.3
2h30m	104.81	97.69	**50**	**0.91**	10.3	**80.6**	9.1
2h45m	104.80	97.68	**50**	**0.91**	11.3	**79.0**	9.8
3h	104.82	97.67	**75**	**1.37**	49.5	**6.1**	44.4
[DCC] is the concentration of the dynamic covalent chemistry reaction at any time =[1-1]+[2-2]+[1-2]							
[1-2] = **Form A** + **Form B**							

8.7.3 **PXRD scans, tabulation of Rietveld refinement of PXRD data and corresponding experimental milling equilibrium curves (Procedure 1)**

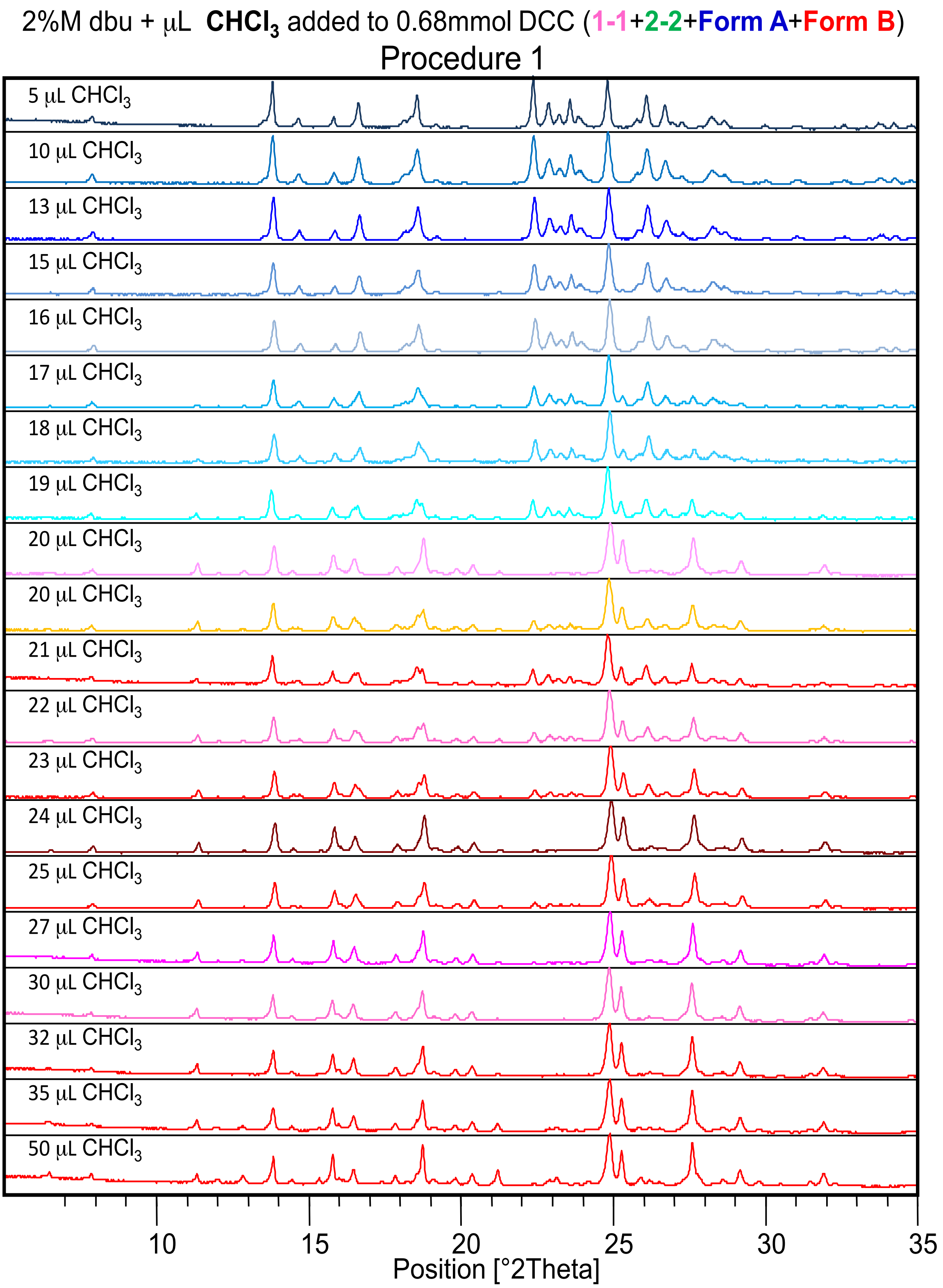

PXRD scan 10 Ball mill LAG reaction at equilibrium with different volumes of **$CHCl_3$**. Each PXRD scan was obtained at equilibrium from a single point experiment with the specified added volume of **$CHCl_3$**. The PXRD sample was prepared immediately after completion of the grinding experiment, and scanned between 5 and 45° (2θ).

Table S 39 Tabulation of the phase composition and corresponding R index used to prepare the milling equilibrium curve with **$CHCl_3$** as the LAG solvent using ball mill grinding **Procedure 1**. The phase composition was calculated by Rietveld refinement of PXRD data as %M of the mixture of starting materials (**1-1** and **2-2**) and the polymorphs of the product (**Form A** and **Form B**). The corresponding refinement errors (esd) have been calculated as %mol. The R index is calculated as the ratio between the concentration of **Form B** versus the total concentration of **Form B** plus **Form A**.

Ball mill LAG grinding reaction taken to thermodynamic equilibrium using Procedure 1													
2%M dbu + µL $CHCl_3$ added to 0.68 mmol DCC ([1-1]+[2-2]+[Form A]+[Form B])													
grinding time @ 30Hz to equilib.	$CHCl_3$ added to powder		**1-1**		**2-2**		**Form B**		**Form A**		[A]+[B]	R = [B] / ([A]+[B])
	$CHCl_3$ µL	[$CHCl_3$]/[DCC] (mol/mol)	%M	e.s.d. mol%	%M	e.s.d. mol%	%M	e.s.d. mol%	%M	e.s.d. mol%	total polymorph	% R
2h30m	**5**	**0.09**	**0.5**	0.2	**0.4**	0.1	**2.8**	0.4	**96.3**	0.5	99.1	**2.8**
2h30m	**10**	**0.18**	**0.7**	0.2	**0.6**	0.1	**2.2**	0.3	**96.6**	0.4	98.8	**2.2**
2h	**13**	**0.24**	**0.8**	0.2	**0.6**	0.1	**2.7**	0.3	**95.9**	0.4	98.6	**2.7**
1h15m	**15**	**0.27**	**3.0**	0.3	**2.4**	0.3	**7.5**	0.5	**87.1**	0.6	94.6	**7.9**
2h	**16**	**0.29**	**1.1**	0.2	**0.9**	0.2	**2.5**	0.3	**95.5**	0.4	98.0	**2.6**
1h	**17**	**0.31**	**3.2**	0.3	**2.6**	0.2	**26.5**	0.4	**67.7**	0.4	94.2	**28.1**
1h30m	**18**	**0.33**	**3.0**	0.5	**2.4**	0.4	**27.9**	0.7	**66.8**	0.8	94.7	**29.5**
2h	**19**	**0.35**	**3.5**	0.5	**2.8**	0.4	**41.6**	0.7	**52.0**	0.7	93.6	**44.4**
45m	**20**	**0.36**	**5.0**	0.2	**4.0**	0.2	**89.2**	0.4	**1.9**	0.3	91.1	**97.9**
2h	**20**	**0.36**	**3.1**	0.4	**2.5**	0.3	**63.0**	0.5	**31.4**	0.5	94.4	**66.7**
2h	**21**	**0.38**	**3.4**	0.4	**2.7**	0.3	**44.4**	0.6	**49.5**	0.6	93.9	**47.3**
2h	**22**	**0.40**	**4.2**	0.3	**3.4**	0.2	**56.6**	0.4	**35.8**	0.4	92.4	**61.3**
2h30m	**23**	**0.42**	**1.6**	0.3	**1.3**	0.2	**67.7**	0.5	**29.4**	0.4	97.1	**69.7**
2h	**24**	**0.44**	**4.9**	0.3	**3.9**	0.3	**86.5**	0.6	**4.7**	0.4	91.2	**94.8**
2h	**25**	**0.46**	**2.8**	0.3	**2.2**	0.2	**77.8**	0.5	**17.2**	0.4	95.0	**81.9**
2h	**27**	**0.49**	**3.7**	0.4	**3.0**	0.3	**88.0**	0.6	**5.2**	0.5	93.2	**94.4**
2h	**30**	**0.55**	**2.9**	0.4	**2.4**	0.3	**93.1**	0.6	**1.6**	0.4	94.7	**98.3**
3h	**32**	**0.58**	**4.1**	0.3	**3.3**	0.2	**91.2**	0.5	**1.4**	0.3	92.6	**98.5**
3h30m	**35**	**0.64**	**9.5**	0.3	**7.7**	0.2	**81.8**	0.4	**1.0**	0.3	82.8	**98.8**
2h30m	**50**	**0.91**	**14.5**	0.3	**11.7**	0.3	**73.0**	0.4	**0.9**	0.3	73.9	**98.8**

[DCC] is the concentration of the dynamic covalent chemistry reaction at any time =[1-1]+[2-2]+[Form A]+[Form B]

[**1-2**] = **Form A** + **Form B** [A] ≡ [**Form A**] [B] ≡ [**Form B**]

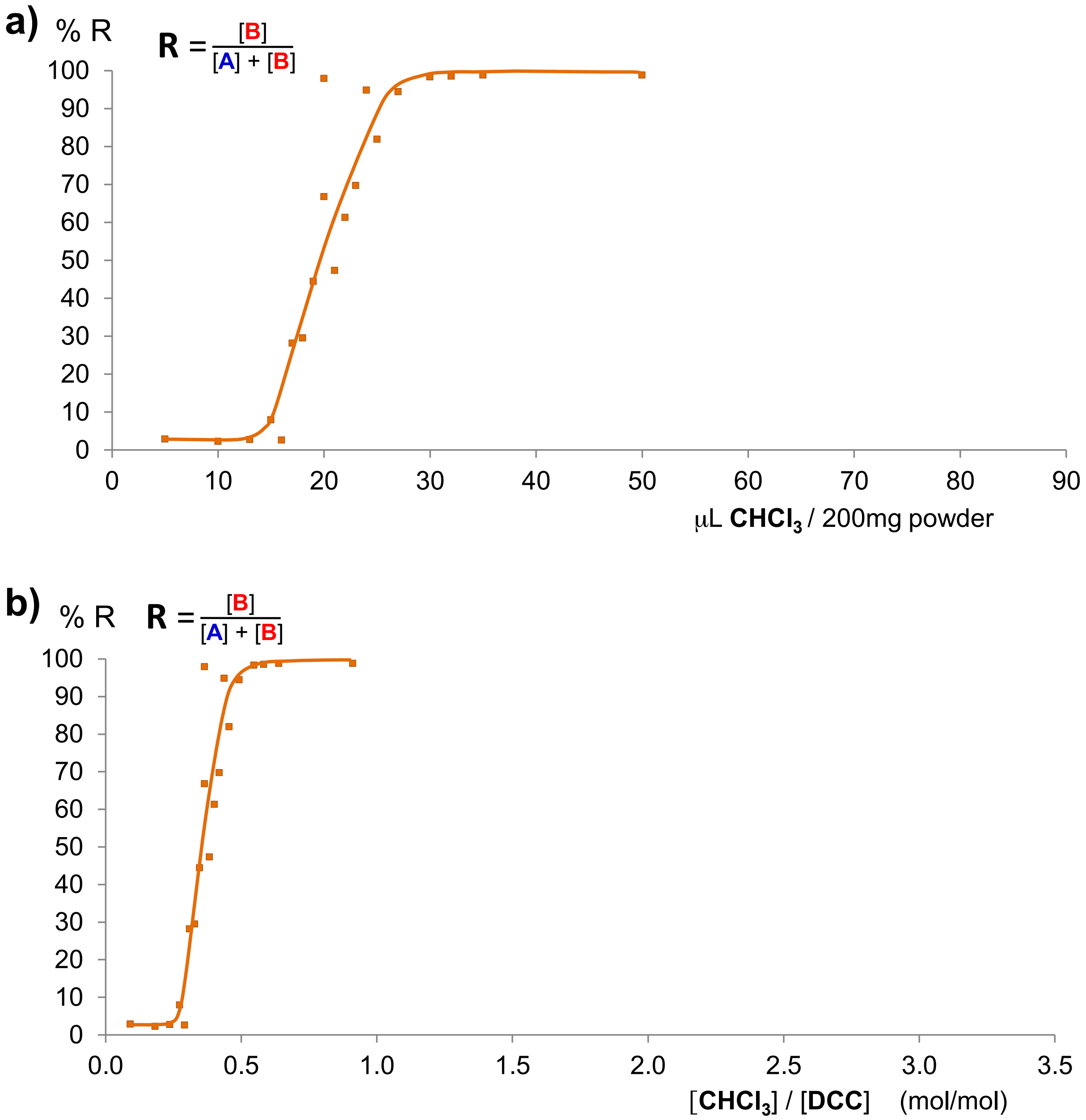

[**DCC**] is the concentration of the dynamic covalent chemistry reaction at any time = [**1-1**]+[**2-2**]+[**A**]+[**B**]
[**A**] is the concentration of **Form A** in DCC; [**B**] is the concentration of **Form B** in DCC

Figure S 47 Experimental milling equilibrium curves using "ball mill grinding procedure 1" plotted as the concentration of $CHCl_3$ versus %R index. R is the ratio of the concentration of **Form B** with respect to the total concentration of **Form B** plus **Form A** formed in the solid state DCC reaction for each specific experimental point. **a)** Curve expressed as μL $CHCl_3$ / 200 mg powder and **b)** curve expressed as mols of $CHCl_3$ added to the reaction with respect to 0.68 mmol of DCC library; this library is composed by the homodimers **1-1** and **2-2** and the heterodimers **Form A** and **Form B**. R is calculated from the Rietveld refinement of PXRD data giving the phase composition of the solid-state DCC experiment at equilibrium as %M. Each point in the graph is an individual experiment. No fitting was performed and the curves drawn are only a guide to the eye.

8.8 LAG experiments using dichloromethane as grinding liquid on solid-state DCC reaction. (Procedure 2)

These experiments were performed by adding the solvent with a 25uL and 50uL syringe using Procedure 2 (Section 4.5.2).

8.8.1 Accuracy and precision of dispensing DCM

Table S 40 Tabulation of accuracy and precision of DCM dispensed, calibrated by weighing experiments

Density:	1.3943		24.0°C		bp:	39.62°C					
Dispensed volume (µL)	**Hamilton syringe** 10-25µL (25µL syringe) 25-50µL (50µL syringe)						Average weight (mg)	Std deviation	Relative Std dev	volume DCM (µL)	Accuracy error (%)
	1	2	3								
10	12.95	12.78	12.35				12.69	0.31	2.44	9.10	-9.85
15	19.43	19.41	19.49				19.44	0.04	0.21	13.94	-7.57
20	25.66	25.42	25.88				25.65	0.23	0.90	18.40	-8.70
21	27.06	27.07	27.18				27.10	0.07	0.25	19.44	-8.03
22	28.23	28.39	28.38				28.33	0.09	0.32	20.32	-8.26
23	29.63	29.51	29.73				29.62	0.11	0.37	21.25	-8.26
24	31.11	30.52	31.12				30.92	0.34	1.11	22.17	-8.24
25	32.23	32.13	32.40				32.25	0.14	0.42	23.13	-8.07
25	32.38	32.44	32.49				32.44	0.06	0.17	23.26	-7.46
26	33.21	33.12	33.57				33.30	0.24	0.72	23.88	-8.86
27	34.49	34.34	34.75				34.53	0.21	0.60	24.76	-9.03
28	36.23	36.00	35.95				36.06	0.15	0.41	25.86	-8.27
30	38.99	38.45	39.18				38.87	0.38	0.97	27.88	-7.60
35	45.34	45.35	45.05				45.25	0.17	0.38	32.45	-7.85
40	51.50	50.60	51.65				51.25	0.57	1.11	36.76	-8.82
50	63.09	62.50	64.59				63.39	1.08	1.70	45.47	-9.97

a)

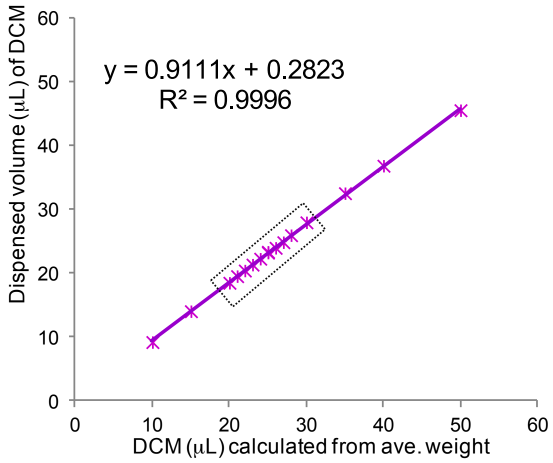

b)

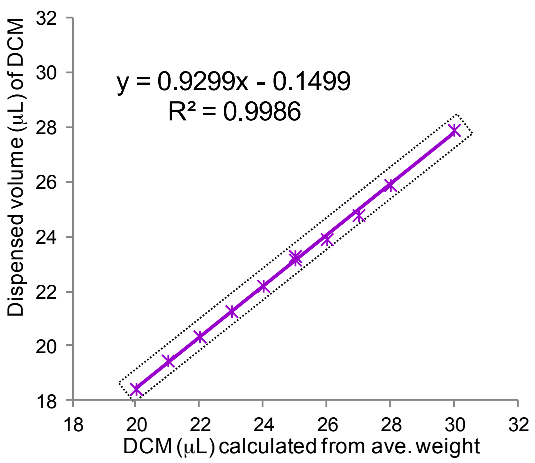

Figure S 48 Accuracy and precision of DCM dispensed, calibrated by weighing experiments.
a) Range 10-100µL DCM; b) expanded narrow range from 20-30 µL DCM

8.8.2 Experimental details and HPLC data (Procedure 2)

Table S 41 Experimental details of ball mill LAG experiments with **DCM** as grinding solvent used to prepare the milling equilibrium curves and corresponding HPLC data. Tabulation of weights (mg) of starting materials (**1-1** & **2-2**), volume of **DCM** added to the solid state DCC reaction and the corresponding grinding time used to ensure that thermodynamic equilibrium is achieved for each single experimental point. The chemical composition of the reaction product as %M obtained by HPLC analysis is tabulated. Details of HPLC method are included.

Ball mill LAG taken to thermodynamic equilibrium using Procedure 2							
2%M dbu + μL **DCM** added to 0.68 mmol **DCC** ([**1-1**] + [**2-2**] + [**1-2**])							
Reagents:	**$(2NO_2PhS)_2$**	**$(4ClPhS)_2$**	DCM added		**HPLC conditions**		
MW:	308.33	287.23	to powder		Zorbax XDB C18, 1.8 μm; 4.6x50 mm		
%M initial	50%M	50%M	MW	Densiity	A: H_2O+0.1% FA; B: MeCN+0.1%FA		
mmol	0.34	0.34	(g/mol)	(g/mL)	0-2min 75-85%B;		
mg	104.8 mg	97.6 mg	89.93	1.3943	2ml/min; 60°C; 259 nm (8nm bandwidth)		
					**HPLC results**		
grinding time @ 30 Hz to equilibrium	$(2NO_2PhS)_2$ weighed (mg)	$(4ClPhS)_2$ weighed (mg)	DCM μL	[DCM]/[DCC] (mol/mol)	$(2NO_2PhS)_2$ (1-1) %M	Product (**1-2**) %M	$(4ClPhS)_2$ (2-2) %M
3h	104.80	97.67	**15**	**0.34**	1.4	**97.1**	1.5
3h	104.80	97.64	**18**	**0.41**	2.7	**95.2**	2.1
3h	104.84	97.68	**20**	**0.46**	6.2	**88.9**	4.9
4h	104.82	97.64	**22**	**0.50**	5.2	**90.6**	4.2
4h	104.84	97.67	**23**	**0.52**	5.1	**90.8**	4.2
4h	104.84	97.66	**24**	**0.55**	4.4	**91.8**	3.7
3h30m	104.81	97.64	**25**	**0.57**	8.1	**85.3**	6.6
4h	104.86	97.65	**26**	**0.59**	8.3	**88.6**	5.1
4h	104.81	97.67	**27**	**0.62**	8.7	**84.0**	7.3
4h	104.82	97.65	**28**	**0.64**	7.8	**91.9**	6.6
4h	104.81	97.64	**30**	**0.68**	8.4	**84.8**	6.8
4h	104.84	97.68	**35**	**0.80**	9.0	**83.2**	7.7
5h	104.81	97.65	**50**	**1.14**	12.1	**76.4**	11.5
2h	104.85	97.69	**60**	**1.37**	11.3	**78.2**	10.5
[DCC] is the concentration of the dynamic covalent chemistry reaction at any time =[1-1]+[2-2]+[1-2]							
[1-2] = **Form A** + **Form B**							

8.8.3 **PXRD scans, tabulation of Rietveld refinement of PXRD data and corresponding experimental milling equilibrium curves (Procedure 2)**

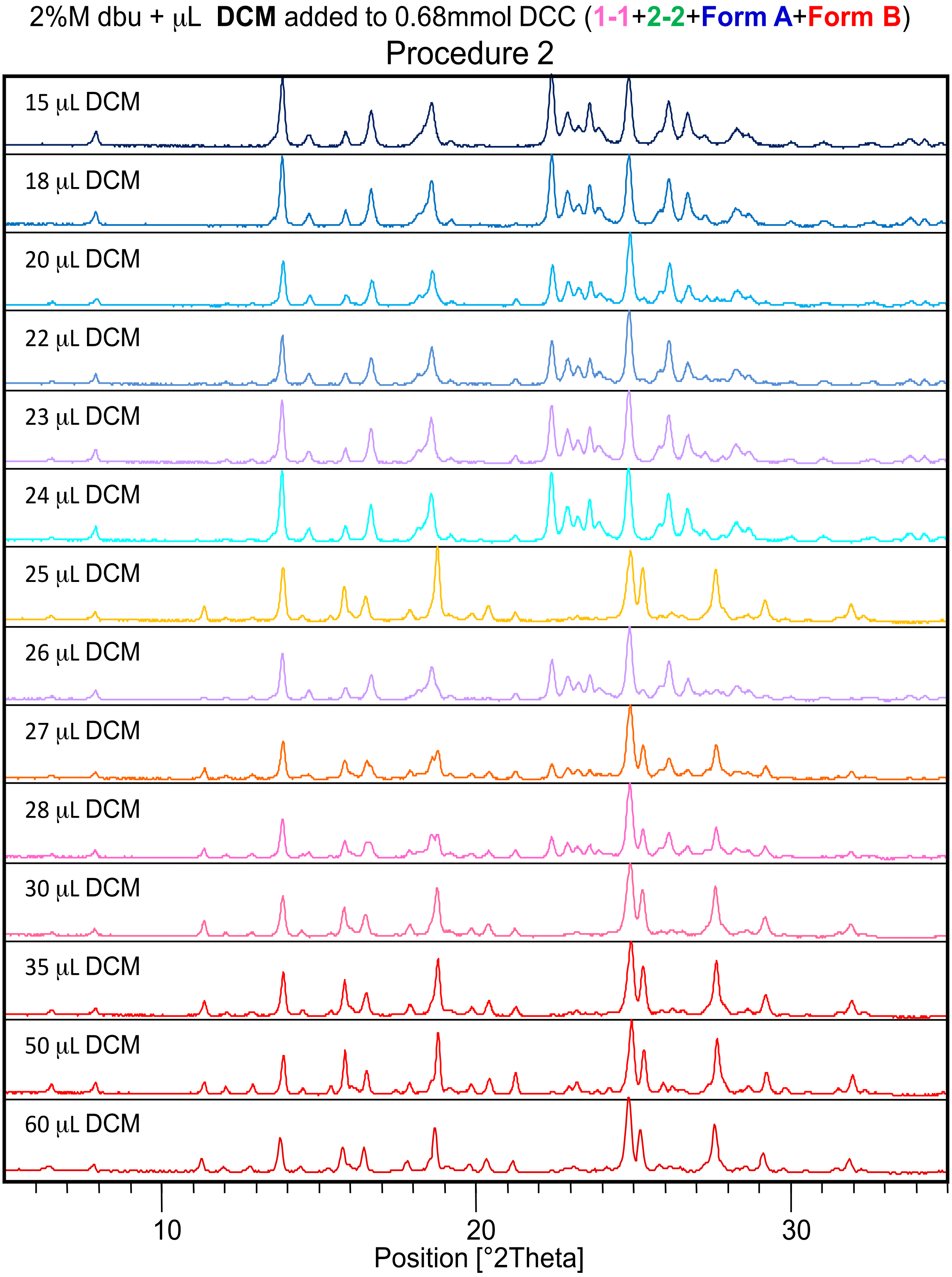

PXRD scan 11 Ball mill LAG reaction at equilibrium with different volumes of **DCM**. Each PXRD scan was obtained at equilibrium from a single point experiment with the specified added volume of **DCM**. The PXRD sample was prepared immediately after completion of the grinding experiment, and scanned between 5 and 45° (2θ).

Table S 42 Tabulation of the phase composition and corresponding R index used to prepare the milling equilibrium curve with **DCM** as the LAG solvent using ball mill grinding **Procedure 2**. The phase composition was calculated by Rietveld refinement of PXRD data as %M of the mixture of starting materials (**1-1** and **2-2**) and the polymorphs of the product (**Form A** and **Form B**). The corresponding refinement errors (esd) have been calculated as %mol. The R index is calculated as the ratio between the concentration of **Form B** versus the total concentration of **Form B** plus **Form A**.

Ball mill LAG grinding reaction taken to thermodynamic equilibrium using Procedure 2												
2%M dbu+μL DCM added to 0.68 mmol DCC ([1-1]+[2-2]+[Form A]+[Form B])												
grinding time @ 30Hz to equilib.	DCM added to powder		1-1		2-2		Form B		Form A		[A]+[B]	R = [B] / ([A]+[B])
	DCM μL	[DCM]/[DCC] (mol/mol)	%M	e.s.d. mol%	%M	e.s.d. mol%	%M	e.s.d. mol%	%M	e.s.d. mol%	total polymorph	% R
3h	**15**	**0.34**	**1.1**	0.2	**0.9**	0.2	**5.3**	0.5	**92.6**	0.5	97.9	**5.4**
3h	**18**	**0.41**	**1.9**	0.3	**1.5**	0.2	**4.1**	0.4	**92.4**	0.5	96.5	**4.2**
3h	**20**	**0.46**	**5.3**	0.3	**4.3**	0.3	**10.9**	0.4	**79.5**	0.5	90.4	**12.1**
4h	**22**	**0.50**	**4.7**	0.3	**3.8**	0.3	**8.9**	0.5	**82.6**	0.6	91.5	**9.7**
4h	**23**	**0.52**	**4.7**	0.3	**3.8**	0.2	**3.8**	0.4	**87.7**	0.5	91.5	**4.2**
4h	**24**	**0.55**	**3.7**	0.3	**3.0**	0.2	**3.6**	0.4	**89.7**	0.5	93.3	**3.9**
3h30m	**25**	**0.57**	**6.6**	0.3	**5.3**	0.2	**86.4**	0.5	**1.6**	0.3	88.0	**98.2**
4h	**26**	**0.59**	**5.5**	0.3	**4.4**	0.3	**15.9**	0.4	**74.3**	0.5	90.2	**17.6**
4h	**27**	**0.62**	**6.9**	0.3	**5.6**	0.3	**55.9**	0.5	**31.6**	0.4	87.5	**63.9**
4h	**28**	**0.64**	**6.4**	0.3	**5.2**	0.3	**45.8**	0.5	**42.6**	0.5	88.4	**51.8**
4h	**30**	**0.68**	**6.6**	0.3	**5.3**	0.3	**85.9**	0.5	**2.1**	0.4	88.0	**97.6**
4h	**35**	**0.80**	**8.1**	0.3	**6.5**	0.2	**84.2**	0.4	**1.1**	0.3	85.3	**98.7**
5h	**50**	**1.14**	**14.5**	0.3	**11.7**	0.2	**73.0**	0.4	**0.7**	0.3	73.7	**99.1**
2h	**60**	**1.37**	**10.8**	0.6	**8.7**	0.5	**79.0**	0.9	**1.6**	0.7	80.6	**98.0**
[DCC] is the concentration of the dynamic covalent chemistry reaction at any time =[1-1]+[2-2]+[Form A]+[Form B]												
[**1-2**] = **Form A** + **Form B** [A] ≡ [**Form A**] [B] ≡ [**Form B**]												

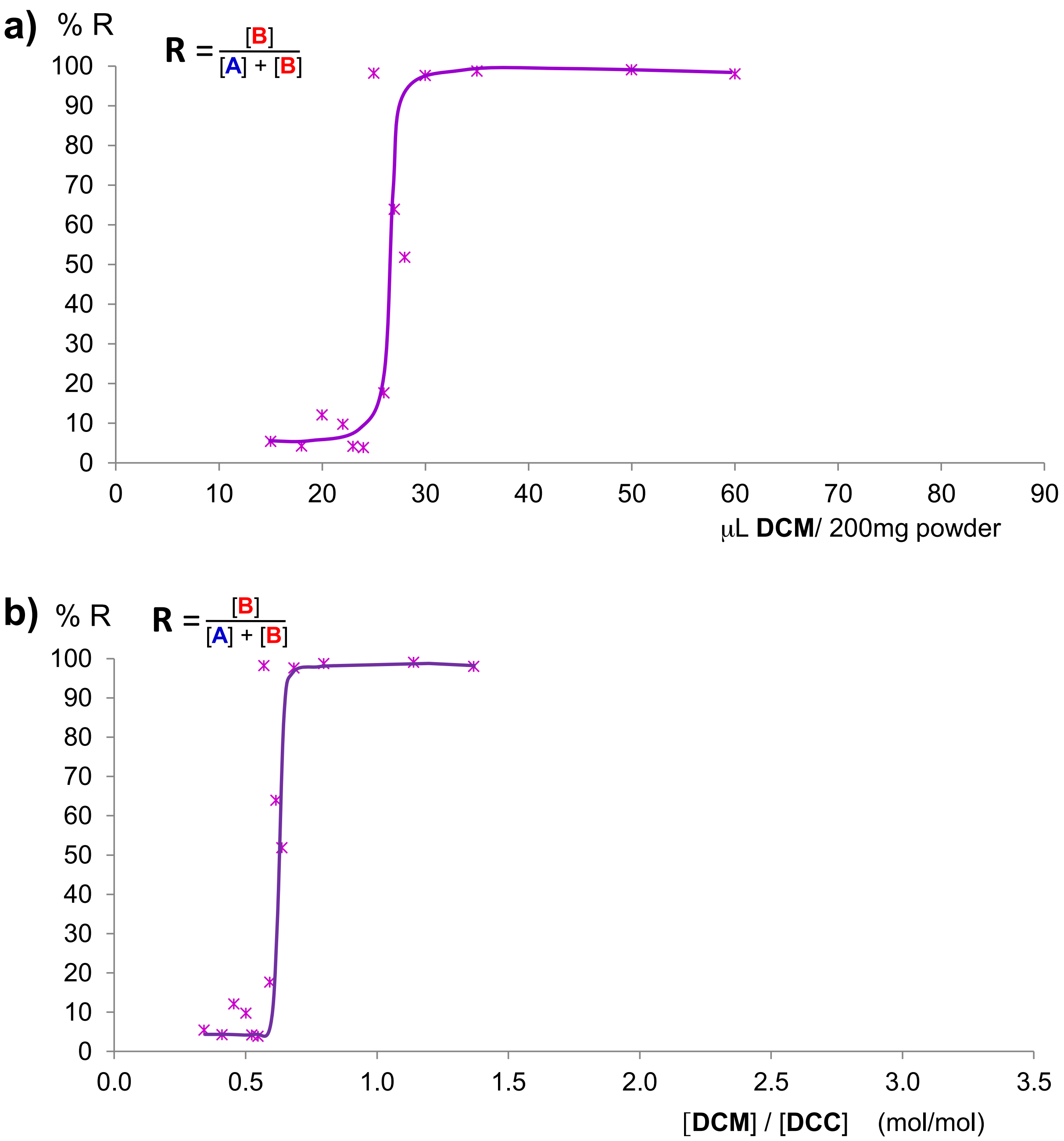

[**DCC**] is the concentration of the dynamic covalent chemistry reaction at any time = [**1-1**]+[**2-2**]+[**A**]+[**B**]
[**A**] is the concentration of **Form A** in DCC; [**B**] is the concentration of **Form B** in DCC

Figure S 49 Experimental milling equilibrium curves using "ball mill grinding procedure 2" plotted as the concentration of DCM versus %R index. R is the ratio of the concentration of **Form B** with respect to the total concentration of **Form B** plus **Form A** formed in the solid state DCC reaction for each specific experimental point. **a)** Curve expressed as µL DCM / 200 mg powder and **b)** curve expressed as mols of DCM added to the reaction with respect to 0.68 mmol of DCC library; this library is composed by the homodimers **1-1** and **2-2** and the heterodimers **Form A** and **Form B**. R is calculated from the Rietveld refinement of PXRD data giving the phase composition of the solid-state DCC experiment at equilibrium as %M. Each point in the graph is an individual experiment. No fitting was performed and the curves drawn are only a guide to the eye.

8.9 LAG experiments using dimethyl sulfoxide as grinding liquid on solid-state DCC reaction (Procedure 4)

These experiments were performed by adding the solvent by normal pipetting using Procedure 4 (Section 4.5.4).

8.9.1 Accuracy and precision of dispensing DMSO (Procedure 4)

Table S 43 Tabulation of accuracy and precision of DMSO dispensed, calibrated by weighing experiments

Density:	1.096		24.0°C		bp:	189°C					
Dispensed volume (μL)	**NORMAL PIPETTING** aspiration speed = 1, dispensing speed = 1						Average weight (mg)	Std deviation	Relative Std dev	volume DMSO (μL)	Accuracy error (%)
	1	2	3	4							
10	11.25	11.49	11.38				11.37	0.12	1.06	10.38	3.65
20	22.21	22.19	23.35				22.58	0.66	2.94	20.61	2.95
30	33.34	33.08	33.22				33.21	0.13	0.39	30.31	1.02
38	42.10	42.08	42.99				42.39	0.52	1.23	38.68	1.77
39	44.00	43.09	43.13				43.41	0.51	1.18	39.61	1.54
40	44.49	44.43	44.18				44.37	0.16	0.37	40.49	1.21
41	45.02	46.16	45.74				45.64	0.58	1.26	41.65	1.56
42	47.52	47.31	47.27				47.37	0.13	0.28	43.23	2.84
43	48.34	47.72	47.56				47.87	0.41	0.86	43.69	1.57
44	48.85	48.92	48.64				48.80	0.15	0.30	44.54	1.21
45	49.49	49.77	49.91				49.72	0.21	0.43	45.38	0.83
46	51.28	50.77	52.01	51.02			51.27	0.54	1.04	46.79	1.68
47	51.86	51.90	51.90				51.89	0.02	0.04	47.35	0.74
48	53.19	52.89	52.88				52.99	0.18	0.33	48.35	0.73
50	56.34	55.31	56.04				55.90	0.53	0.95	51.01	1.98
60	66.42	66.01	66.30				66.24	0.21	0.32	60.45	0.75
70	77.78	77.27	77.11				77.39	0.35	0.45	70.62	0.88
80	88.49	88.35	88.14				88.33	0.18	0.20	80.60	0.75
90	99.00	99.29	99.30				99.20	0.17	0.17	90.52	0.58
100	111.16	110.18	110.18				110.51	0.57	0.51	100.85	0.84

a) b)

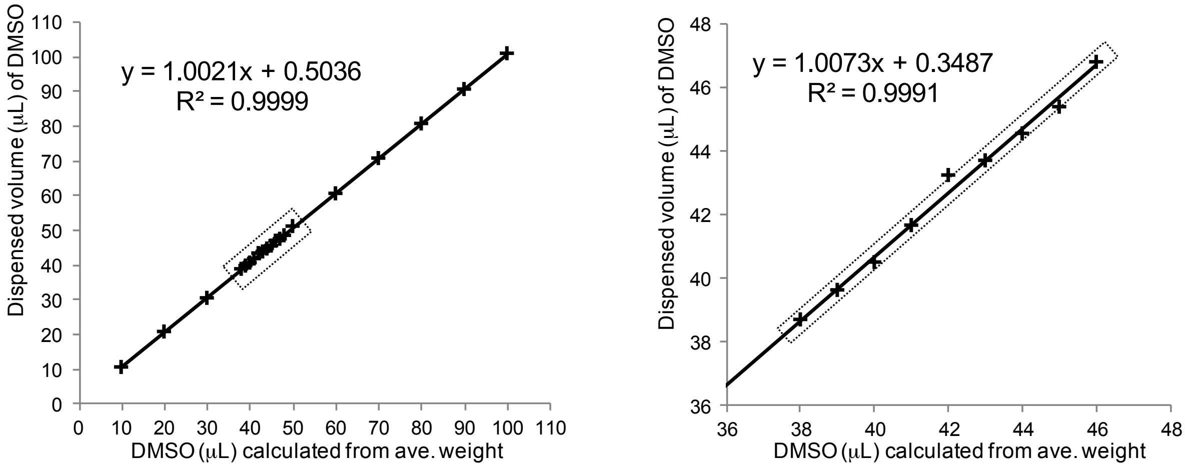

Figure S 50 Accuracy and precision of DMSO dispensed, calibrated by weighing experiments.
a) Range 10-100μL DMSO; b) expanded narrow range from 36-48 μL DMSO

8.9.2 Experimental details and HPLC data (Procedure 4)

Table S 44 Experimental details of ball mill LAG experiments with **DMSO** as grinding solvent used to prepare the milling equilibrium curves and corresponding HPLC data. Tabulation of weights (mg) of starting materials (**1-1** & **2-2**), volume of **DMSO** added to the solid state DCC reaction and the corresponding grinding time used to ensure that thermodynamic equilibrium is achieved for each single experimental point. The chemical composition of the reaction product as %M obtained by HPLC analysis is tabulated. Details of HPLC method are included.

Ball mill LAG grinding reaction taken to thermodynamic equilibrium using Procedure 4

2%M dbu + µL **DMSO** added to 0.68 mmol **DCC** ([**1-1**] + [**2-2**] + [**1-2**])

Reagents:		**$(2NO_2PhS)_2$**	**$(4ClPhS)_2$**	DMSO added to powder		**HPLC conditions**		
MW:		308.33	287.23			Zorbax XDB C18, 1.8 µm; 4.6x50 mm		
%M initial		50%M	50%M	MW	Densiity	A: H_2O+0.1% FA; B: MeCN+0.1%FA		
mmol		0.34	0.34	(g/mol)	(g/mL)	0-2min 75-85%B;		
mg		104.8 mg	97.6 mg	78.13	1.0958	2ml/min; 60°C; 259 nm (8nm bandwidth)		
						**HPLC results**		
pre-soaking time	grinding time @ 30 Hz to equilibrium	$(2NO_2PhS)_2$ weighed (mg)	$(4ClPhS)_2$ weighed (mg)	DMSO µL	[DMSO]/[DCC] (mol/mol)	$(2NO_2PhS)_2$ **(1-1)** %M	Product **(1-2)** %M	$(4ClPhS)_2$ **(2-2)** %M
4h	50 m	104.84	97.64	25	0.52	4.6	**92.7**	3.4
4h	1h	104.83	97.64	35	0.72	3.9	**93.0**	3.1
4h	1h	104.81	97.65	37	0.76	2.0	**95.9**	2.1
4.5h	1h	104.83	97.65	39	0.80	3.7	**92.5**	3.8
5h	1h	104.83	97.66	41	0.85	6.4	**88.2**	5.4
4h45m	1h30m	104.83	97.66	42	0.87	0.9	**98.2**	0.9
5.5h	1h	104.82	97.67	43	0.89	6.7	**86.9**	6.4
5h15m	1h30m	104.85	97.68	44	0.91	3.2	**93.8**	3.0
5h	1h	104.80	97.64	45	0.93	3.8	**92.8**	3.4
5.5h	1h30m	104.81	97.64	47	0.97	6.2	**87.2**	6.6

[DCC] is the concentration of the dynamic covalent chemistry reaction at any time =[1-1]+[2-2]+[1-2]

[1-2] = **Form A** + **Form B**

8.9.3 **PXRD scans, tabulation of Rietveld refinement of PXRD data and corresponding experimental milling equilibrium curves (Procedure 4)**

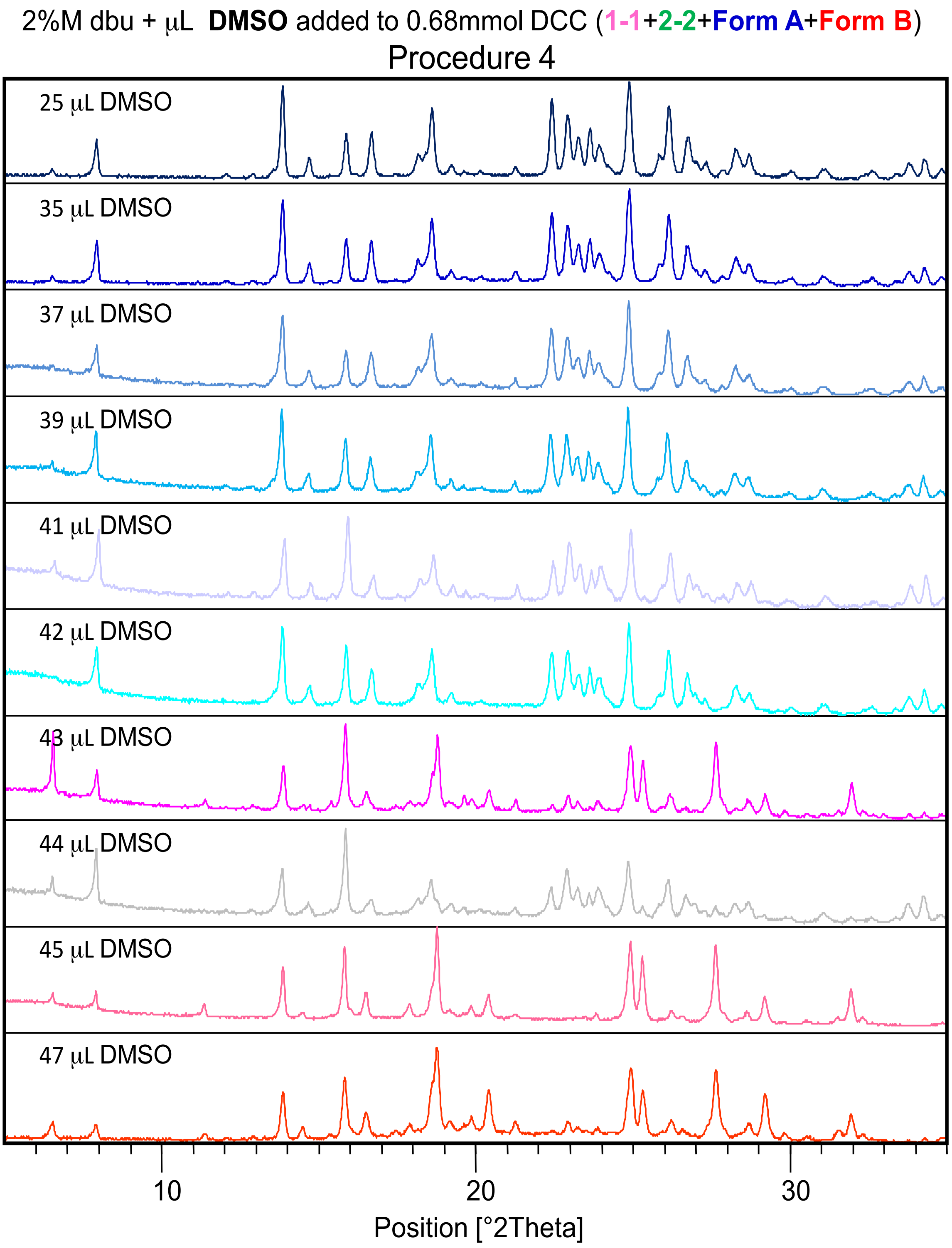

PXRD scan 12 Ball mill LAG reaction at equilibrium with different volumes of **DMSO**. Each PXRD scan was obtained at equilibrium from a single point experiment with the specified added volume of **DMSO**. The PXRD sample was prepared immediately after completion of the grinding experiment, and scanned between 5 and 45° (2θ).

Table S 45 Tabulation of the phase composition and corresponding R index used to prepare the milling equilibrium curve with **DMSO** as the LAG solvent using ball mill grinding **Procedure 4**. The phase composition was calculated by Rietveld refinement of PXRD data as %M of the mixture of starting materials (**1-1** and **2-2**) and the polymorphs of the product (**Form A** and **Form B**). The corresponding refinement errors (esd) have been calculated as %mol. The R index is calculated as the ratio between the concentration of **Form B** versus the total concentration of **Form B** plus **Form A**.

Ball mill LAG grinding reaction taken to thermodynamic equilibrium using Procedure 4												
2%M dbu+μL DMSO added to 0.68 mmol DCC ([1-1]+[2-2]+[Form A]+[Form B])												
grinding time @ 30Hz to equilib.	DMSO added to powder		1-1		2-2		Form B		Form A		[A]+[B]	R = [B] / ([A]+[B])
	DMSO μL	[DMSO]/[DCC] (mol/mol)	%M	e.s.d. mol%	%M	e.s.d. mol%	%M	e.s.d. mol%	%M	e.s.d. mol%	total polymorph	% R
4h	**25**	**0.52**	**3.9**	0.3	**3.1**	0.3	**3.8**	0.5	**89.2**	0.6	93.0	**4.1**
4h	**35**	**0.72**	**4.0**	0.4	**3.2**	0.3	**5.0**	0.5	**87.8**	0.6	92.8	**5.4**
4h	**37**	**0.76**	**3.8**	0.5	**3.1**	0.4	**4.3**	0.6	**88.8**	0.8	93.1	**4.6**
4.5h	**39**	**0.80**	**4.7**	0.5	**3.8**	0.4	**6.3**	0.7	**85.2**	0.9	91.5	**6.9**
5h	**41**	**0.85**	**8.5**	0.6	**6.8**	0.5	**13.0**	0.8	**71.7**	1.0	84.7	**15.3**
4h45m	**42**	**0.87**	**2.6**	0.7	**2.0**	0.6	**7.4**	0.8	**88.0**	1.1	95.4	**7.8**
5.5h	**43**	**0.89**	**9.5**	0.4	**7.7**	0.4	**75.4**	0.7	**7.4**	0.6	82.8	**91.1**
5h15m	**44**	**0.91**	**8.2**	0.7	**6.6**	0.6	**20.1**	0.9	**65.1**	1.0	85.2	**23.6**
5h	**45**	**0.93**	**3.9**	0.4	**3.2**	0.4	**92.3**	0.6	**0.7**	0.2	93.0	**99.2**
5.5h	**47**	**0.97**	**7.1**	0.5	**5.8**	0.4	**84.4**	0.9	**2.8**	0.7	87.2	**96.8**
[DCC] is the concentration of the dynamic covalent chemistry reaction at any time =[1-1]+[2-2]+[Form A]+[Form B]												
[**1-2**] = **Form A** + **Form B** [A] ≡ [Form A] [B] ≡ [Form B]												

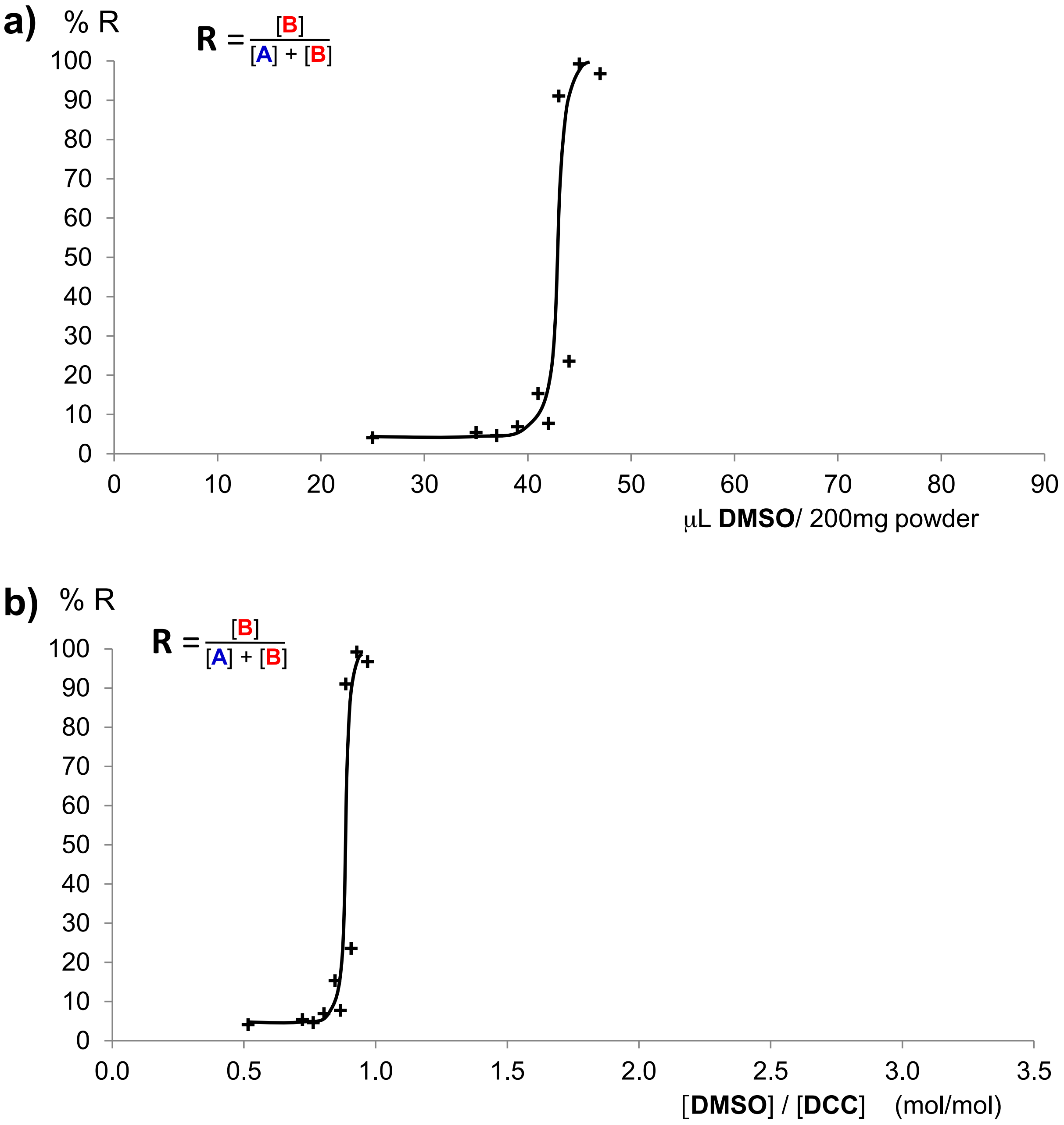

[**DCC**] is the concentration of the dynamic covalent chemistry reaction at any time = [**1-1**]+[**2-2**]+[**A**]+[**B**]
[**A**] is the concentration of **Form A** in DCC; [**B**] is the concentration of **Form B** in DCC

Figure S 51 Experimental milling equilibrium curves using "ball mill grinding procedure 4" plotted as the concentration of DMSO versus %R index. R is the ratio of the concentration of **Form B** with respect to the total concentration of **Form B** plus **Form A** formed in the solid state DCC reaction for each specific experimental point. **a)** Curve expressed as µL DMSO / 200 mg powder and **b)** curve expressed as mols of DMSO added to the reaction with respect to 0.68 mmol of DCC library; this library is composed by the homodimers **1-1** and **2-2** and the heterodimers **Form A** and **Form B**. R is calculated from the Rietveld refinement of PXRD data giving the phase composition of the solid-state DCC experiment at equilibrium as %M. Each point in the graph is an individual experiment. No fitting was performed and the curves drawn are only a guide to the eye.

8.10 LAG experiments using methanol as grinding liquid on solid-state DCC reaction (Procedure 1)

These experiments were performed by adding the solvent by reverse pipetting using Procedure 1 (Section 4.5.1).

8.10.1 Experimental details and HPLC data (Procedure 1)

Table S 46 Experimental details of ball mill LAG experiments with **MeOH** as grinding solvent used to prepare the milling equilibrium curves and corresponding HPLC data. Tabulation of weights (mg) of starting materials (**1-1** & **2-2**), volume of **MeOH** added to the solid state DCC reaction and the corresponding grinding time used to ensure that thermodynamic equilibrium is achieved for each single experimental point. The chemical composition of the reaction product as %M obtained by HPLC analysis is tabulated. Details of HPLC method are included.

Ball mill LAG taken to thermodynamic equilibrium using Procedure 1

2%M dbu + μL **MeOH** added to 0.68 mmol **DCC** ([**1-1**] + [**2-2**] + [**1-2**])

Reagents:	$(2NO_2PhS)_2$	$(4ClPhS)_2$	MeOH added to powder		**HPLC conditions** Zorbax XDB C18, 1.8 μm; 4.6x50 mm; A: H_2O+0.1% FA; B: MeCN+0.1%FA; 0-2min 75-85%B; 2ml/min; 60°C; 259 nm (8nm bandwidth)		
MW:	308.33	287.23					
%M initial	50%M	50%M	MW	Densiity			
mmol	0.34	0.34	(g/mol)	(g/mL)			
mg	104.8 mg	97.6 mg	32.04	0.7872			
					**HPLC results**		
grinding time @ 30 Hz to equilibrium	$(2NO_2PhS)_2$ weighed (mg)	$(4ClPhS)_2$ weighed (mg)	MeOH μL	[MeOH]/[DCC] (mol/mol)	$(2NO_2PhS)_2$ (1-1) %M	Product **(1-2)** %M	$(4ClPhS)_2$ (2-2) %M
5h	104.83	97.66	**30**	**1.08**	1.20	**97.5**	1.3
5h	104.83	97.69	**50**	**1.81**	1.30	**97.5**	1.2
5h	104.83	97.71	**55**	**1.99**	0.40	**99.1**	0.5
5h	104.84	97.73	**60**	**2.17**	3.20	**93.6**	3.3
5h	104.84	97.66	**65**	**2.35**	2.00	**96.3**	1.7
5h	104.82	97.64	**70**	**2.53**	1.10	**97.7**	1.3
4h	104.85	97.68	**80**	**2.89**	1.00	**98.2**	0.8
5h	104.84	97.64	**80**	**2.89**	2.20	**96.3**	1.5
5h	104.82	97.67	**90**	**3.25**	2.60	**94.9**	2.5
5h	104.83	97.71	**100**	**3.61**	1.60	**97.2**	1.2

[DCC] is the concentration of the dynamic covalent chemistry reaction at any time =[1-1]+[2-2]+[1-2]

[1-2] = **Form A** + **Form B**

8.10.2 PXRD scans, tabulation of Rietveld refinement of PXRD data and corresponding experimental milling equilibrium curves (Procedure 1)

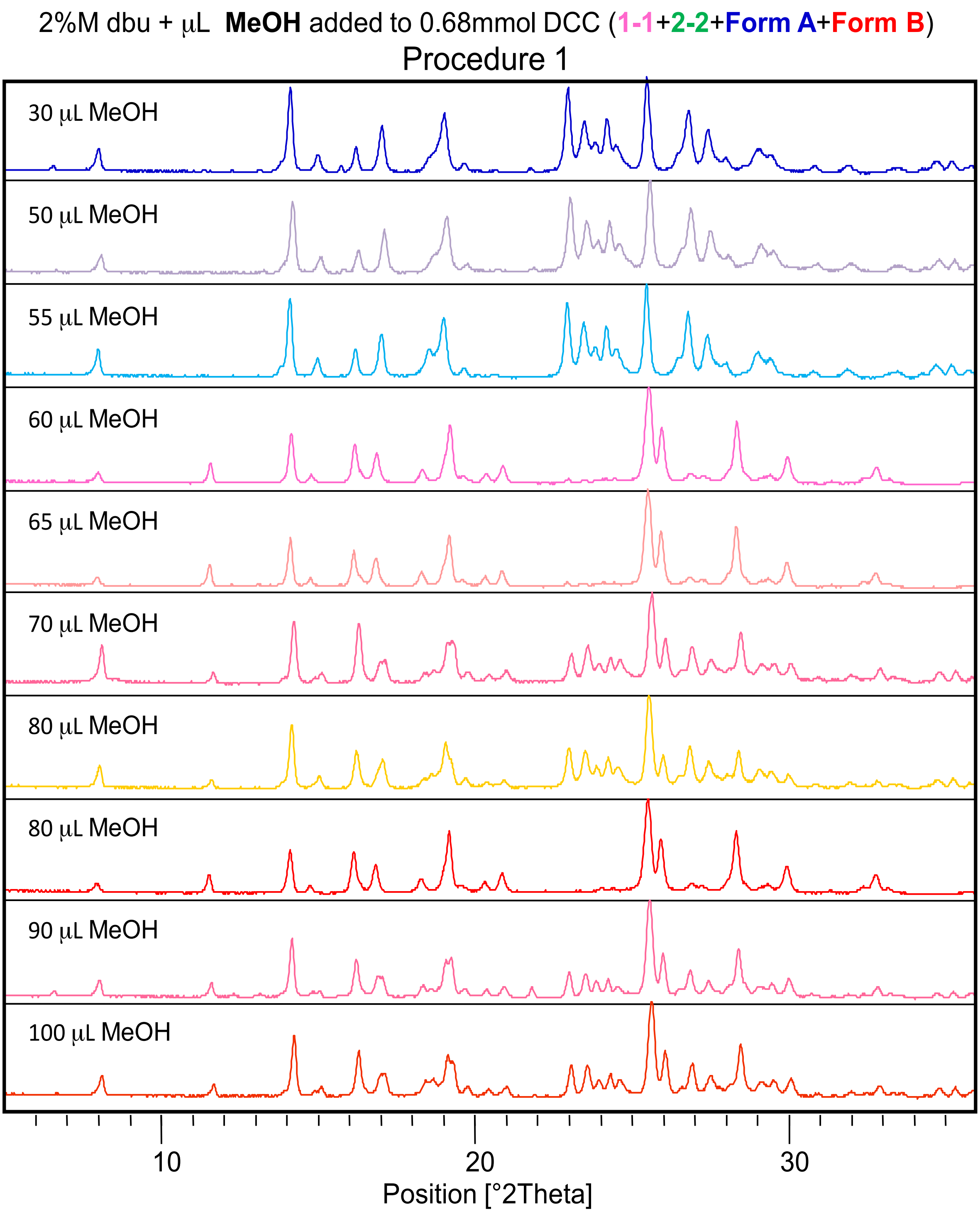

PXRD scan 13 Ball mill LAG reaction at equilibrium with different volumes of **MeOH**. Each PXRD scan was obtained at equilibrium from a single point experiment with the specified added volume of **MeOH**. The PXRD sample was prepared immediately after completion of the grinding experiment, and scanned between 5 and 45° (2θ).

Table S 47 Tabulation of the phase composition and corresponding R index used to prepare the milling equilibrium curve with **MeOH** as the LAG solvent using ball mill grinding **Procedure 1**. The phase composition was calculated by Rietveld refinement of PXRD data as %M of the mixture of starting materials (**1-1** and **2-2**) and the polymorphs of the product (**Form A** and **Form B**). The corresponding refinement errors (esd) have been calculated as %mol. The R index is calculated as the ratio between the concentration of **Form B** versus the total concentration of **Form B** plus **Form A**.

Ball mill LAG grinding reaction taken to thermodynamic equilibrium using Procedure 1													
2%M dbu+μL MeOH added to 0.68 mmol DCC ([1-1]+[2-2]+[Form A]+[Form B])													
grinding time @ 30Hz to equilib.	MeOH added to powder		1-1		2-2		Form B		Form A		[A]+[B]	R = [B] / [A]+[B]
	MeOH μL	[MeOH]/[DCC] (mol/mol)	%M	e.s.d. mol%	%M	e.s.d. mol%	%M	e.s.d. mol%	%M	e.s.d. mol%	total polymorph	% R
5 h	**30**	**1.08**	**2.4**	0.2	**1.9**	0.2	**4.3**	0.5	**91.4**	0.5	95.7	**4.5**
5 h	**50**	**1.81**	**2.9**	0.4	**2.3**	0.3	**3.6**	0.5	**91.2**	0.7	94.8	**3.8**
5 h	**55**	**1.99**	**0.8**	0.2	**0.6**	0.2	**3.9**	0.4	**94.6**	0.5	98.5	**4.0**
5 h	**60**	**2.17**	**1.3**	0.3	**1.1**	0.3	**89.7**	0.6	**7.9**	0.5	97.6	**91.9**
5 h	**65**	**2.35**	**1.3**	0.3	**1.1**	0.2	**89.3**	0.5	**8.3**	0.4	97.6	**91.5**
5 h	**70**	**2.53**	**1.6**	0.4	**1.3**	0.3	**50.6**	0.6	**46.5**	0.6	97.1	**52.1**
5 h	**80**	**2.89**	**0.9**	0.4	**0.7**	0.3	**35.6**	0.5	**62.8**	0.6	98.4	**36.2**
5 h	**80**	**2.89**	**1.1**	0.3	**0.9**	0.3	**96.0**	0.6	**2.1**	0.5	98.1	**97.9**
5 h	**90**	**3.25**	**5.0**	0.3	**4.0**	0.2	**54.5**	0.4	**36.5**	0.4	91.0	**59.9**
5 h	**100**	**3.61**	**1.4**	0.3	**1.1**	0.2	**50.3**	0.5	**47.1**	0.5	97.4	**51.6**
[DCC] is the concentration of the dynamic covalent chemistry reaction at any time =[1-1]+[2-2]+[Form A]+[Form B]												
[1-2] = Form A + Form B [A] ≡ [Form A] [B] ≡ [Form B]												

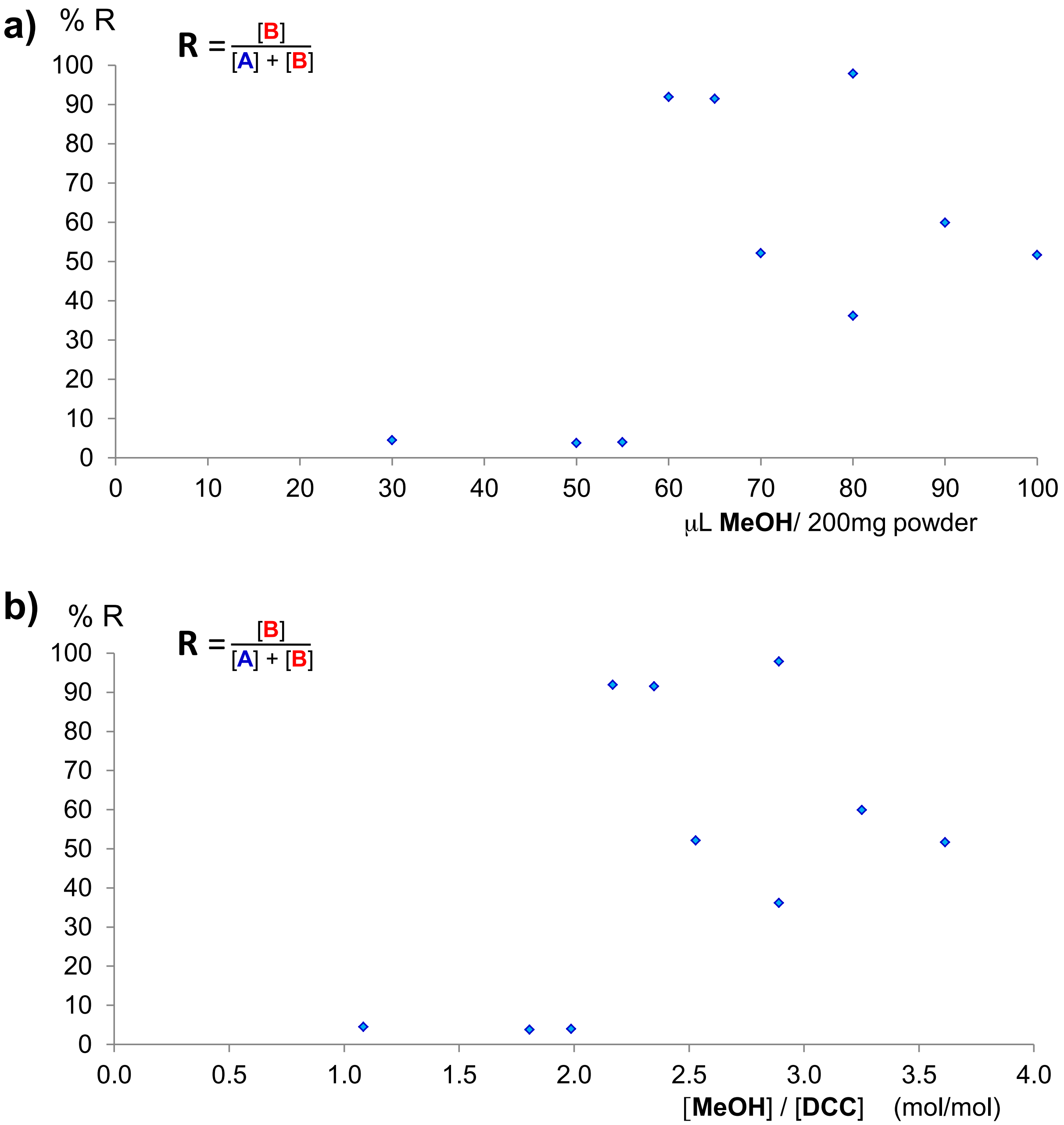

[**DCC**] is the concentration of the dynamic covalent chemistry reaction at any time = [**1-1**]+[**2-2**]+[**A**]+[**B**]
[**A**] is the concentration of **Form A** in DCC; [**B**] is the concentration of **Form B** in DCC

Figure S 52 Experimental milling equilibrium curves using "ball mill grinding procedure 1" plotted as the concentration of MeOH versus %R index. R is the ratio of the concentration of **Form B** with respect to the total concentration of **Form B** plus **Form A** formed in the solid state DCC reaction for each specific experimental point. **a)** Curve expressed as μL MeOH / 200 mg powder and **b)** curve expressed as mols of MeOH added to the reaction with respect to 0.68 mmol of DCC library; this library is composed by the homodimers **1-1** and **2-2** and the heterodimers **Form A** and **Form B**. R is calculated from the Rietveld refinement of PXRD data giving the phase composition of the solid-state DCC experiment at equilibrium as %M. Each point in the graph is an individual experiment.

8.11 LAG experiments using methanol as grinding liquid on solid-state DCC reaction (Procedure 3)

These experiments were performed by adding the solvent by normal pipetting using Procedure 3 (Section 4.5.3).

8.11.1 Accuracy and precision of dispensing volume of MeOH (normal pipetting)

Table S 48 Tabulation of accuracy and precision of dispensed MeOH (normal pipetting), calibrated by weighing experiments

Density:	0.787		24.0°C		bp:	64.7°C					
Dispensed volume (μL)	**NORMAL PIPETTING** aspiration speed = 1, dispensing speed = 1						Average weight (mg)	Std deviation	Relative Std dev	volume MeOH (μL)	Accuracy error (%)
	1	2	3	4							
20	15.32	15.17	15.16	14.72			15.09	0.26	1.72	19.17	-4.32
30	22.73	22.15	22.67	22.99			22.64	0.35	1.55	28.75	-4.33
40	30.65	30.52	30.61	30.59			30.59	0.05	0.18	38.86	-2.93
50	38.34	38.39	38.27	38.42	38.57	38.3	38.38	0.11	0.28	48.76	-2.55
60	45.75	45.84	46.32	46.23	46.54	46.31	46.17	0.31	0.66	58.64	-2.31
70	52.75	54.08	54.09	54.02			53.74	0.66	1.22	68.26	-2.55
80	62.13	62.03	61.91				62.02	0.11	0.18	78.79	-1.54
90	70.13	69.72	69.47				69.77	0.33	0.48	88.63	-1.54
100	77.63	77.68	77.84				77.72	0.11	0.14	98.73	-1.29

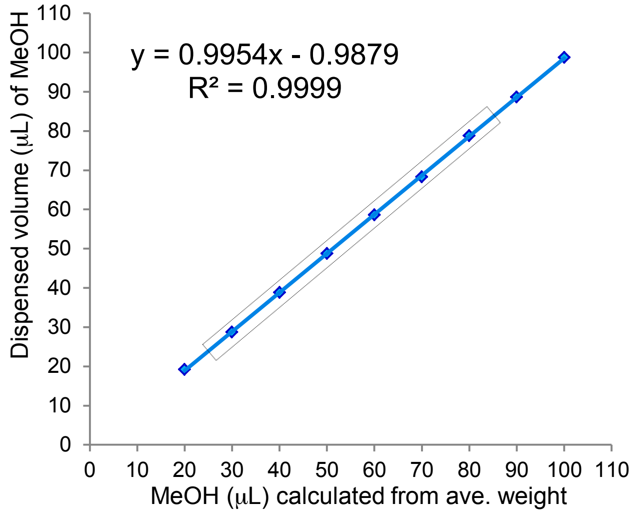

Figure S 53 Accuracy and precision of MeOH dispensed, calibrated by weighing experiments. Range 20-100μL MeOH;

8.11.2 Experimental details and HPLC data (Procedure 3)

Table S 49 Experimental details of ball mill LAG experiments with **MeOH** as grinding solvent used to prepare the milling equilibrium curves and corresponding HPLC data. Tabulation of weights (mg) of starting materials (**1-1** & **2-2**), volume of **MeOH** added to the solid state DCC reaction and the corresponding grinding time used to ensure that thermodynamic equilibrium is achieved for each single experimental point. The chemical composition of the reaction product as %M obtained by HPLC analysis is tabulated. Details of HPLC method are included.

Ball mill LAG grinding reaction taken to thermodynamic equilibrium using Procedure 3

2%M dbu + μL **MeOH** added to 0.68 mmol **DCC** ([**1-1**] + [**2-2**] + [**1-2**])

Reagents:		$(2NO_2PhS)_2$	$(4ClPhS)_2$	MeOH added to powder		**HPLC conditions**		
MW:		308.33	287.23			Zorbax XDB C18, 1.8 μm; 4.6x50 mm		
%M initial		50%M	50%M	MW	Densiity	A: H_2O+0.1% FA; B: MeCN+0.1%FA		
mmol		0.34	0.34	(g/mol)	(g/mL)	0-2min 75-85%B;		
mg		104.8 mg	97.6 mg	32.04	0.7872	2ml/min; 60°C; 259 nm (8nm bandwidth)		
						**HPLC results**		
pre-soaking time	grinding time @ 30 Hz to equilibrium	$(2NO_2PhS)_2$ weighed (mg)	$(4ClPhS)_2$ weighed (mg)	MeOH μL	[MeOH]/[DCC] (mol/mol)	$(2NO_2PhS)_2$ (1-1) %M	Product (**1-2**) %M	$(4ClPhS)_2$ (2-2) %M
1h45m	4h	104.84	97.67	25	0.90	0.3	**99.0**	0.6
20 m	5h	104.85	97.70	50	1.81	0.7	**98.6**	0.7
20 m	4h	104.81	97.66	50	1.81	0.8	**98.1**	1.1
20 m	4h30m	104.83	97.66	60	2.17	0.5	**98.6**	0.9
20 m	4h20m	104.83	97.68	63	2.28	1.3	**97.5**	1.2
20 m	5h	104.85	97.66	64	2.31	0.9	**97.8**	1.3
20 m	5h	104.81	97.67	65	2.35	0.6	**98.5**	0.8
20 m	5h	104.82	97.62	66	2.38	1.2	**97.8**	1.0
20 m	5h	104.81	97.70	67	2.42	2.4	**95.2**	2.4
20 m	5h30m	104.84	97.70	68	2.46	2.6	**94.9**	2.4
20 m	5h	104.84	97.67	69	2.49	1.6	**96.8**	1.5
20 m	5h	104.84	97.66	69	2.49	0.6	**98.6**	0.8
20 m	4h30m	104.82	97.65	70	2.53	2.4	**95.7**	1.9
20 m	5h	104.81	97.63	75	2.71	2.2	**96.0**	1.8
20 m	5h	104.79	97.64	80	2.89	1.6	**96.8**	1.5
20 m	5h	104.86	97.65	85	3.07	1.9	**96.8**	1.3

[DCC] is the concentration of the dynamic covalent chemistry reaction at any time =[1-1]+[2-2]+[1-2]

[1-2] = **Form A** + **Form B**

8.11.3 PXRD scans, tabulation of Rietveld refinement of PXRD data and corresponding experimental milling equilibrium curves (Procedure 3)

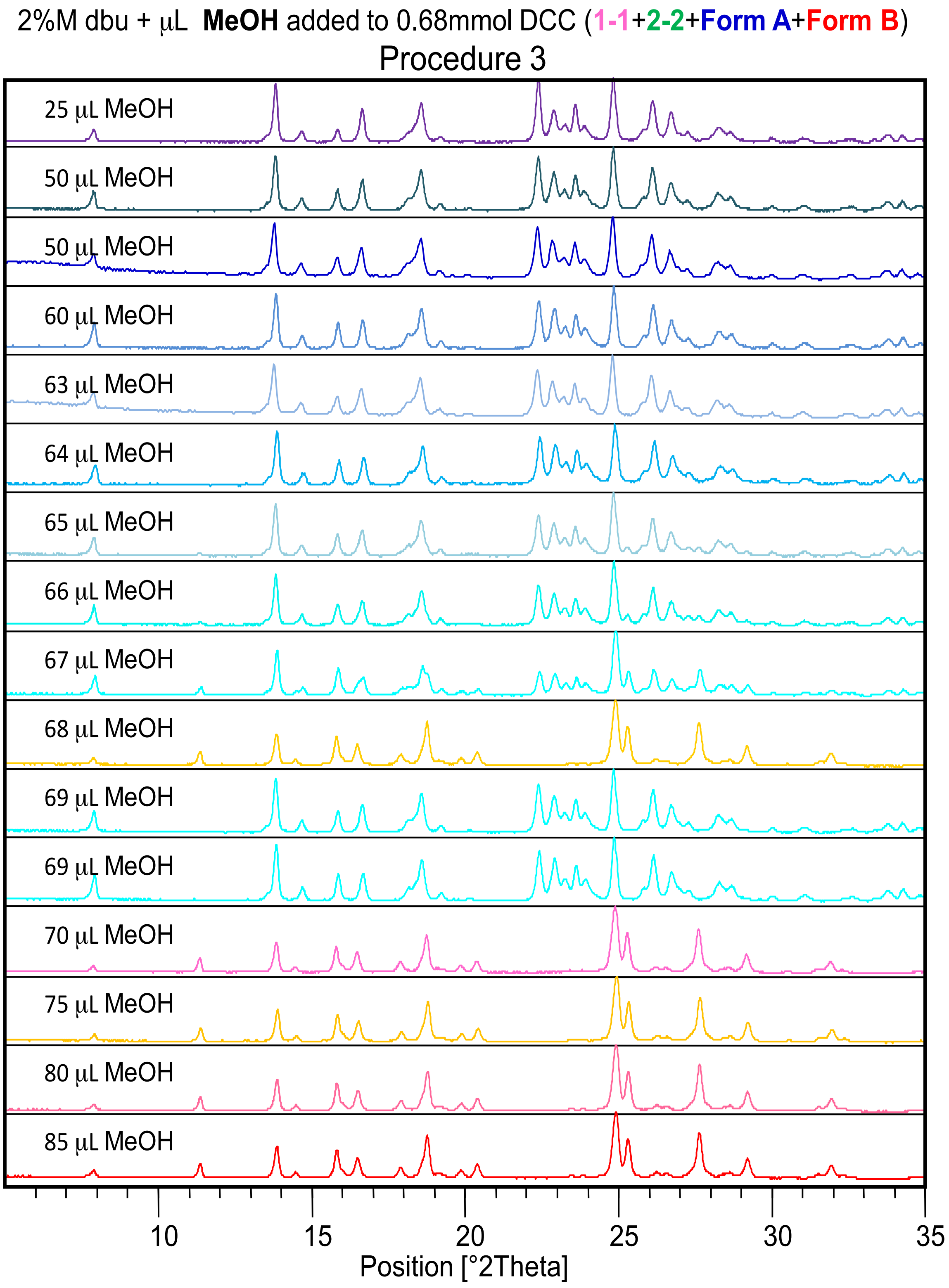

PXRD scan 14 Ball mill LAG reaction at equilibrium with different volumes of **MeOH**. Each PXRD scan was obtained at equilibrium from a single point experiment with the specified added volume of **MeOH**. The PXRD sample was prepared immediately after completion of the grinding experiment, and scanned between 5 and 45° (2θ).

Table S 50 Tabulation of the phase composition and corresponding R index used to prepare the milling equilibrium curve with **MeOH** as the LAG solvent using ball mill grinding **Procedure 3**. The phase composition was calculated by Rietveld refinement of PXRD data as %M of the mixture of starting materials (**1-1** and **2-2**) and the polymorphs of the product (**Form A** and **Form B**). The corresponding refinement errors (esd) have been calculated as %mol. The R index is calculated as the ratio between the concentration of **Form B** versus the total concentration of **Form B** plus **Form A**.

Ball mill LAG grinding reaction taken to thermodynamic equilibrium using Procedure 3

2%M dbu+μL MeOH added to 0.68 mmol DCC ([**1-1**]+[**2-2**]+[**Form A**]+[**Form B**])

pre-soaking time	grinding time @ 30Hz to equilib.	MeOH added to powder		1-1		2-2		(1-2)B		(1-2)A		(1-2)A+(1-2)B	R = (1-2)B / ((1-2)A+(1-2)B)
		MeOH μL	[MeOH]/[DCC] (mol/mol)	%M	e.s.d. mol%	%M	e.s.d. mol%	%M	e.s.d. mol%	%M	e.s.d. mol%	total polymorph	% R
1h45m	4h	**25**	**0.90**	**0.7**	0.2	**0.6**	0.1	**4.2**	0.4	**94.5**	0.5	98.7	**4.3**
20 m	5h	**50**	**1.81**	**0.9**	0.2	**0.7**	0.2	**3.4**	0.5	**95.0**	0.5	98.4	**3.5**
20 m	4h	**50**	**1.81**	**1.8**	0.5	**1.4**	0.4	**3.2**	0.6	**93.6**	0.9	96.8	**3.3**
20 m	4h30m	**60**	**2.17**	**0.8**	0.2	**0.6**	0.2	**4.4**	0.5	**94.1**	0.6	98.5	**4.5**
20 m	4h20m	**63**	**2.28**	**1.9**	0.6	**1.5**	0.5	**2.9**	0.6	**93.7**	0.9	96.6	**3.0**
20 m	5h	**64**	**2.31**	**1.7**	0.4	**1.3**	0.3	**5.8**	0.6	**91.3**	0.8	97.1	**6.0**
20 m	5h	**65**	**2.35**	**0.9**	0.2	**0.7**	0.2	**13.8**	0.5	**84.5**	0.6	98.3	**14.0**
20 m	5h	**66**	**2.38**	**1.1**	0.3	**0.8**	0.2	**15.6**	0.5	**82.5**	0.6	98.1	**15.9**
20 m	5h	**67**	**2.42**	**1.0**	0.3	**0.8**	0.3	**42.1**	0.5	**56.1**	0.5	98.2	**42.9**
20 m	5h30m	**68**	**2.46**	**0.8**	0.3	**0.6**	0.2	**96.3**	0.5	**2.2**	0.4	98.5	**97.8**
20 m	5h	**69**	**2.49**	**1.0**	0.3	**0.8**	0.2	**4.3**	0.5	**93.9**	0.6	98.2	**4.4**
20 m	5h	**69**	**2.49**	**1.1**	0.3	**0.9**	0.2	**5.1**	0.6	**92.8**	0.7	97.9	**5.2**
20 m	4h30m	**70**	**2.53**	**1.0**	0.3	**0.8**	0.2	**96.2**	0.5	**2.0**	0.4	98.2	**98.0**
20 m	5h	**75**	**2.71**	**0.8**	0.2	**0.7**	0.2	**96.7**	0.5	**1.8**	0.4	98.5	**98.2**
20 m	5h	**80**	**2.89**	**0.8**	0.2	**0.6**	0.2	**97.5**	0.4	**1.1**	0.3	98.6	**98.9**
20 m	5h	**85**	**3.07**	**0.8**	0.3	**0.6**	0.2	**97.4**	0.4	**1.2**	0.3	98.6	**98.8**

[DCC] is the concentration of the dynamic covalent chemistry reaction at any time =[**1-1**]+[**2-2**]+[**Form A**]+[**Form B**]

[**1-2**] = **Form A** + **Form B** [**A**] ≡ [**Form A**] [**B**] ≡ [**Form B**]

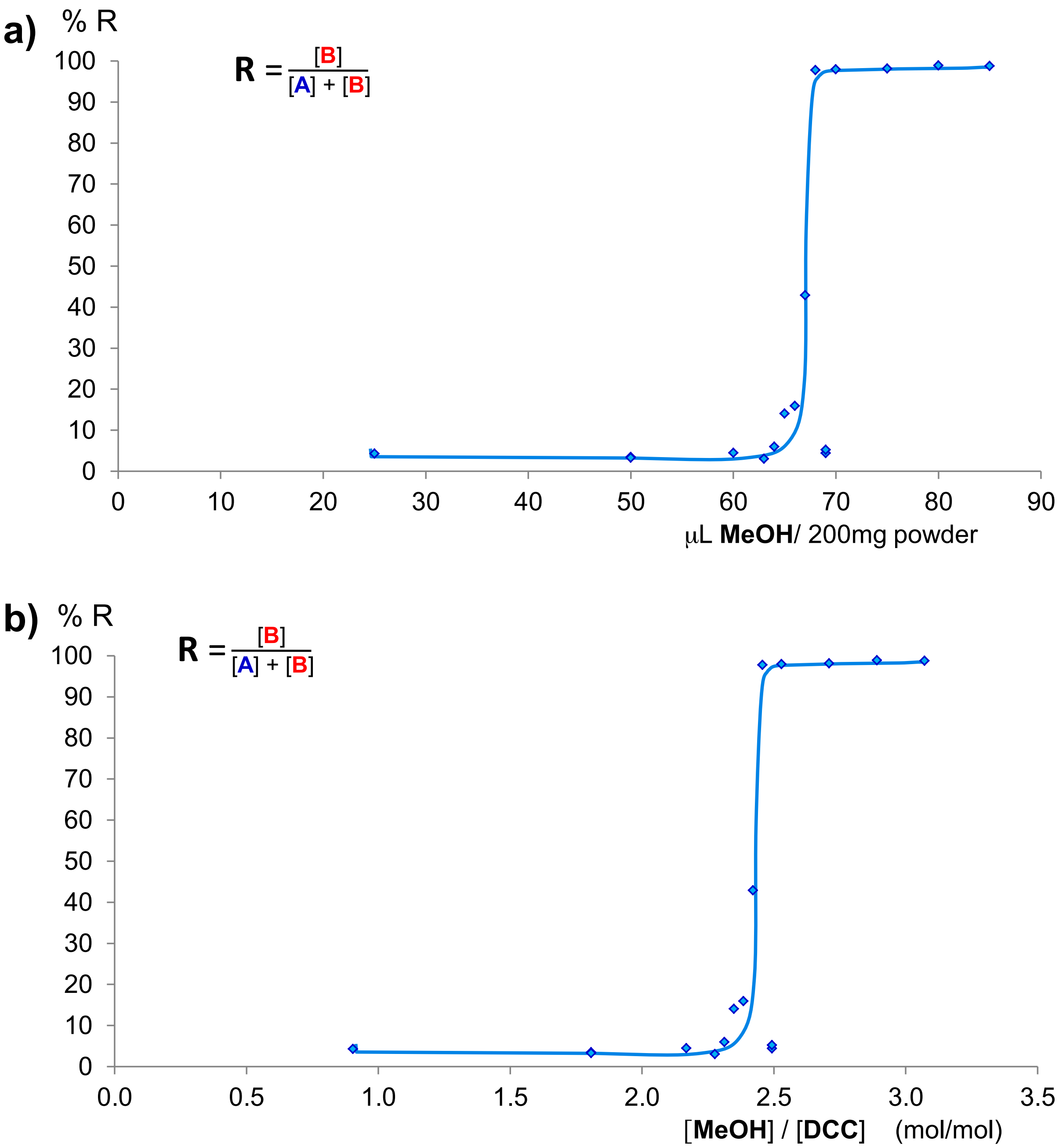

[**DCC**] is the concentration of the dynamic covalent chemistry reaction at any time = [**1-1**]+[**2-2**]+[**A**]+[**B**]
[**A**] is the concentration of **Form A** in DCC; [**B**] is the concentration of **Form B** in DCC

Figure S 54 Experimental milling equilibrium curves using "ball mill grinding procedure 3" plotted as the concentration of MeOH versus %R index. R is the ratio of the concentration of **Form B** with respect to the total concentration of **Form B** plus **Form A** formed in the solid state DCC reaction for each specific experimental point. **a)** Curve expressed as μL MeOH / 200 mg powder and **b)** curve expressed as mols of MeOH added to the reaction with respect to 0.68 mmol of DCC library; this library is composed by the homodimers **1-1** and **2-2** and the heterodimers **Form A** and **Form B**. R is calculated from the Rietveld refinement of PXRD data giving the phase composition of the solid-state DCC experiment at equilibrium as %M. Each point in the graph is an individual experiment. No fitting was performed and the curves drawn are only a guide to the eye.

8.12 LAG experiments using ethanol as grinding liquid on solid-state DCC reaction (Procedure 4)

These experiments were performed by adding the solvent by normal pipetting using Procedure 4 (Section 4.5.4).

8.12.1 Accuracy and precision of dispensing volume of EtOH (Procedure 4)

Table S 51 Tabulation of accuracy and precision of EtOH dispensed, calibrated by weighing experiments

Density:	0.785		24.0°C		bp:	78.4°C					
Dispensed volume (μL)	**NORMAL PIPETTING** aspiration speed = 1, dispensing speed = 1						Average weight (mg)	Std deviation	Relative Std dev	volume EtOH (μL)	Accuracy error (%)
	1	2	3	4							
40	31.54	31.61	31.71				31.62	0.09	0.27	40.29	0.72
50	39.56	39.46	39.70				39.57	0.12	0.30	50.42	0.84
60	47.31	47.30	47.36				47.32	0.03	0.07	60.30	0.50
65	51.40	51.27	51.15				51.27	0.13	0.24	65.33	0.51
66	52.12	51.98	51.65				51.92	0.24	0.46	66.15	0.23
67	53.12	53.00	53.41				53.18	0.21	0.40	67.76	1.12
68	54.17	53.95	54.04				54.05	0.11	0.20	68.88	1.27
69	54.68	54.63	54.94				54.75	0.17	0.30	69.76	1.09
70	55.74	55.49	55.28				55.50	0.23	0.41	70.72	1.02
71	55.82	56.38	55.97				56.06	0.29	0.52	71.43	0.60
72	57.07	57.43	56.8				57.10	0.32	0.55	72.76	1.04
73	57.77	57.59	57.75				57.70	0.10	0.17	73.53	0.72
74	57.96	58.62	58.07				58.22	0.35	0.61	74.18	0.24
75	59.22	59.20	58.95				59.12	0.15	0.25	75.34	0.45
80	62.63	63.06	62.91	63.35			62.99	0.30	0.48	80.26	0.32
90	70.94	70.82	70.6	71.68			71.01	0.47	0.66	90.48	0.53
100	79.46	79.42	79.36				79.41	0.05	0.06	101.19	1.18

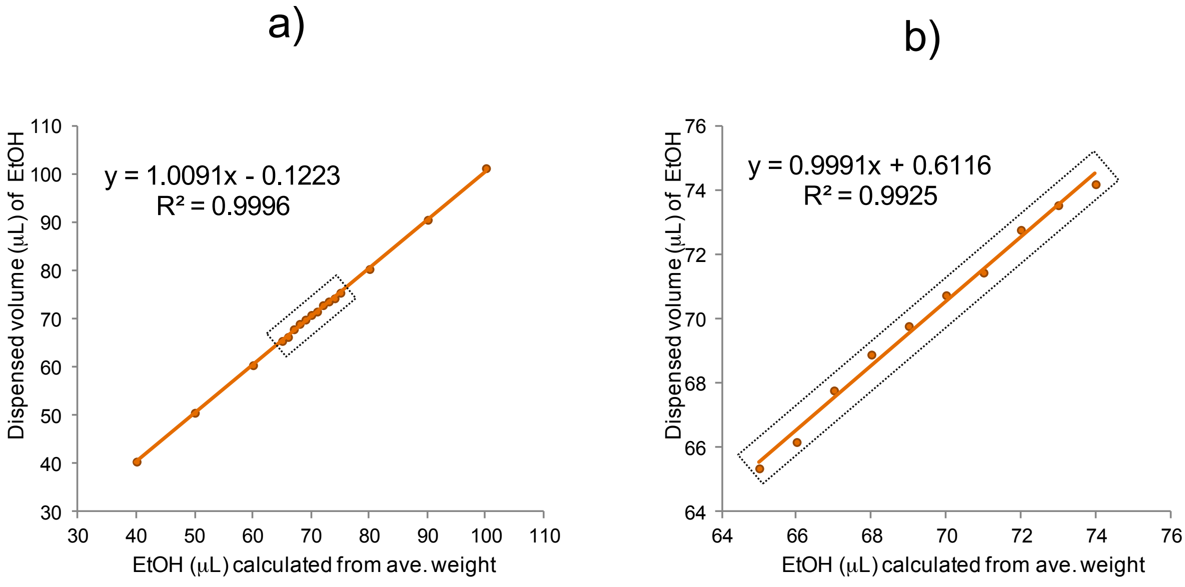

Figure S 55 Accuracy and precision of EtOH dispensed, calibrated by weighing experiments. a) range 40-100μL EtOH; b) expanded narrow range from 65-75 μL EtOH

8.12.2 Experimental details and HPLC data (Procedure 4)

Table S 52 Experimental details of ball mill LAG experiments with **EtOH** as grinding solvent used to prepare the milling equilibrium curves and corresponding HPLC data. Tabulation of weights (mg) of starting materials (**1-1** & **2-2**), volume of **EtOH** added to the solid state DCC reaction and the corresponding grinding time used to ensure that thermodynamic equilibrium is achieved for each single experimental point. The chemical composition of the reaction product as %M obtained by HPLC analysis is tabulated. Details of HPLC method are included.

Ball mill LAG grinding reaction taken to thermodynamic equilibrium using Procedure 4								
2%M dbu + µL **EtOH** added to 0.68 mmol **DCC** ([**1-1**] + [**2-2**] + [**1-2**])								
Reagents:		$(2NO_2PhS)_2$	$(4ClPhS)_2$	EtOH added		**HPLC conditions**		
MW:		308.33	287.23	to powder		Zorbax XDB C18, 1.8 µm; 4.6x50 mm		
%M initial		50%M	50%M	MW	Densiity	A: H_2O+0.1% FA; B: MeCN+0.1%FA		
mmol		0.34	0.34	(g/mol)	(g/mL)	0-2min 75-85%B;		
mg		104.8 mg	97.6 mg	46.07	0.7848	2ml/min; 60°C; 259 nm (8nm bandwidth)		
						**HPLC results**		
pre-soaking time	grinding time @ 30 Hz to equilibrium	$(2NO_2PhS)_2$ weighed (mg)	$(4ClPhS)_2$ weighed (mg)	EtOH µL	[EtOH]/[DCC] (mol/mol)	$(2NO_2PhS)_2$ (1-1) %M	Product (**1-2**) %M	$(4ClPhS)_2$ (2-2) %M
45m	4h	104.82	97.67	**25**	**0.63**	0.8	**98.7**	0.6
45m	3h	104.83	97.68	**50**	**1.25**	1.6	**96.9**	1.5
1h	4h	104.81	97.64	**55**	**1.38**	1.5	**97.0**	1.4
1h	4h	104.82	97.66	**60**	**1.50**	1.2	**97.3**	1.5
45m	3.5h	104.81	97.64	**64**	**1.60**	1.4	**97.1**	1.5
48m	3h20m	104.85	97.66	**65**	**1.63**	1.1	**98.0**	1.0
51m	4h20m	104.83	97.65	**66**	**1.65**	1.0	**97.0**	1.6
51m	4h	104.81	97.64	**67**	**1.68**	2.6	**94.9**	2.6
48m	3h	104.82	97.67	**68**	**1.70**	1.5	**97.2**	1.3
38m	5h	104.81	97.67	**69**	**1.73**	1.7	**97.1**	1.2
39m	3h	104.84	97.69	**70**	**1.75**	2.1	**96.4**	1.5
33m	5h20m	104.82	97.69	**71**	**1.78**	0.9	**98.4**	0.7
38m	4h	104.82	97.68	**72**	**1.80**	1.4	**97.6**	1.3
27m	4h	104.83	97.68	**73**	**1.83**	1.7	**96.7**	1.6
45m	4	104.83	97.66	**75**	**1.88**	2.2	**96.7**	2.2
1h20m	4h	104.84	97.68	**77**	**1.93**	1.7	**96.8**	1.4
1h10m	4h	104.81	97.65	**80**	**2.00**	1.8	**96.9**	1.4
>1h	8h	104.84	97.65	**90**	**2.25**	1.8	**96.7**	1.5
[DCC] is the concentration of the dynamic covalent chemistry reaction at any time =[1-1]+[2-2]+[1-2]								
[1-2] = **Form A** + **Form B**								

8.12.3 **PXRD scans, tabulation of Rietveld refinement of PXRD data and corresponding experimental milling equilibrium curves (Procedure 4)**

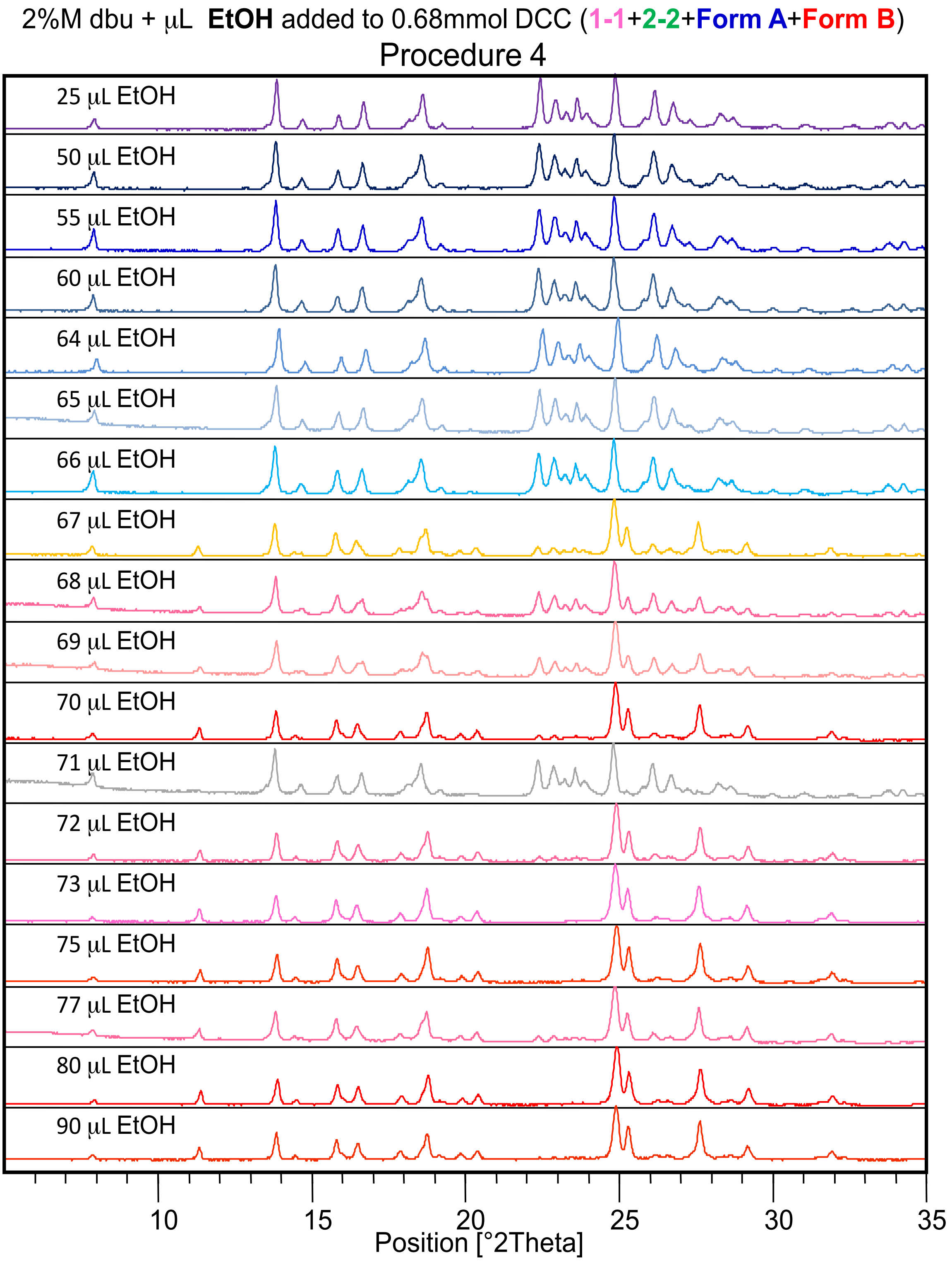

PXRD scan 15 Ball mill LAG reaction at equilibrium with different volumes of **EtOH**. Each PXRD scan was obtained at equilibrium from a single point experiment with the specified added volume of **EtOH**. The PXRD sample was prepared immediately after completion of the grinding experiment, and scanned between 5 and 45° (2θ).

Table S 53 Tabulation of the phase composition and corresponding R index used to prepare the milling equilibrium curve with **EtOH** as the LAG solvent using ball mill grinding **Procedure 4**. The phase composition was calculated by Rietveld refinement of PXRD data as %M of the mixture of starting materials (**1-1** and **2-2**) and the polymorphs of the product (**Form A** and **Form B**). The corresponding refinement errors (esd) have been calculated as %mol. The R index is calculated as the ratio between the concentration of **Form B** versus the total concentration of **Form B** plus **Form A**.

Ball mill LAG grinding reaction taken to thermodynamic equilibrium using Procedure 4

2%M dbu+μL EtOH added to 0.68 mmol DCC ([**1-1**]+[**2-2**]+[**Form A**]+[**Form B**])

pre-soaking time	grinding time @ 30Hz to equilib.	EtOH added to powder		1-1		2-2		Form B		Form A		[A]+[B]	R = [B] / [A]+[B]
		EtOH μL	[EtOH]/[DCC] (mol/mol)	%M	e.s.d. mol%	%M	e.s.d. mol%	%M	e.s.d. mol%	%M	e.s.d. mol%	total polymorph	% R
45m	4h	**25**	**0.63**	**1.0**	0.3	**0.8**	0.3	**2.0**	0.4	**96.3**	0.6	98.3	**2.0**
45m	3h	**50**	**1.25**	**2.1**	0.3	**1.7**	0.3	**4.3**	0.5	**91.9**	0.6	96.2	**4.5**
1h	4h	**55**	**1.38**	**1.9**	0.3	**1.5**	0.2	**4.5**	0.4	**92.1**	0.5	96.6	**4.7**
1h	4h	**60**	**1.50**	**1.0**	0.2	**0.8**	0.2	**3.5**	0.4	**94.7**	0.5	98.2	**3.6**
45m	3.5h	**64**	**1.60**	**1.1**	0.3	**0.9**	0.2	**2.8**	0.4	**95.2**	0.6	98.0	**2.9**
48m	3h20m	**65**	**1.63**	**1.9**	0.4	**1.5**	0.3	**3.8**	0.5	**92.8**	0.6	96.6	**3.9**
51m	4h20m	**66**	**1.65**	**1.0**	0.3	**0.8**	0.2	**7.6**	0.6	**90.6**	0.7	98.2	**7.7**
51m	4h	**67**	**1.68**	**0.9**	0.3	**0.7**	0.3	**71.6**	0.6	**26.9**	0.5	98.5	**72.7**
48m	3h	**68**	**1.70**	**1.5**	0.3	**1.2**	0.3	**36.6**	0.5	**60.7**	0.5	97.3	**37.6**
38m	5h	**69**	**1.73**	**1.6**	0.4	**1.3**	0.3	**48.0**	0.5	**49.1**	0.5	97.1	**49.4**
39m	3h	**70**	**1.75**	**1.2**	0.3	**1.0**	0.2	**83.6**	0.5	**14.2**	0.4	97.8	**85.5**
33m	5h20m	**71**	**1.78**	**2.1**	0.4	**1.7**	0.3	**10.6**	0.5	**85.6**	0.7	96.2	**11.0**
38m	4h	**72**	**1.80**	**0.6**	0.2	**0.5**	0.2	**84.2**	0.5	**14.8**	0.4	99.0	**85.1**
27m	4h	**73**	**1.83**	**0.8**	0.3	**0.6**	0.2	**96.7**	0.5	**1.9**	0.3	98.6	**98.1**
45m	4	**75**	**1.88**	**1.3**	0.3	**1.0**	0.2	**95.6**	0.5	**2.1**	0.4	97.7	**97.9**
1h20m	4h	**77**	**1.93**	**1.6**	0.4	**1.2**	0.3	**79.1**	0.6	**18.1**	0.5	97.2	**81.4**
1h10m	4h	**80**	**2.00**	**0.5**	0.2	**0.4**	0.2	**97.2**	0.4	**1.8**	0.3	99.0	**98.2**
1h15m	8h	**90**	**2.25**	**1.8**	0.5	**1.5**	0.4	**91.1**	1.0	**5.6**	0.7	96.7	**94.2**

[DCC] is the concentration of the dynamic covalent chemistry reaction at any time =[**1-1**]+[**2-2**]+[**Form A**]+[**Form B**]

[**1-2**] = **Form A** + **Form B** [**A**] ≡ [**Form A**] [**B**] ≡ [**Form B**]

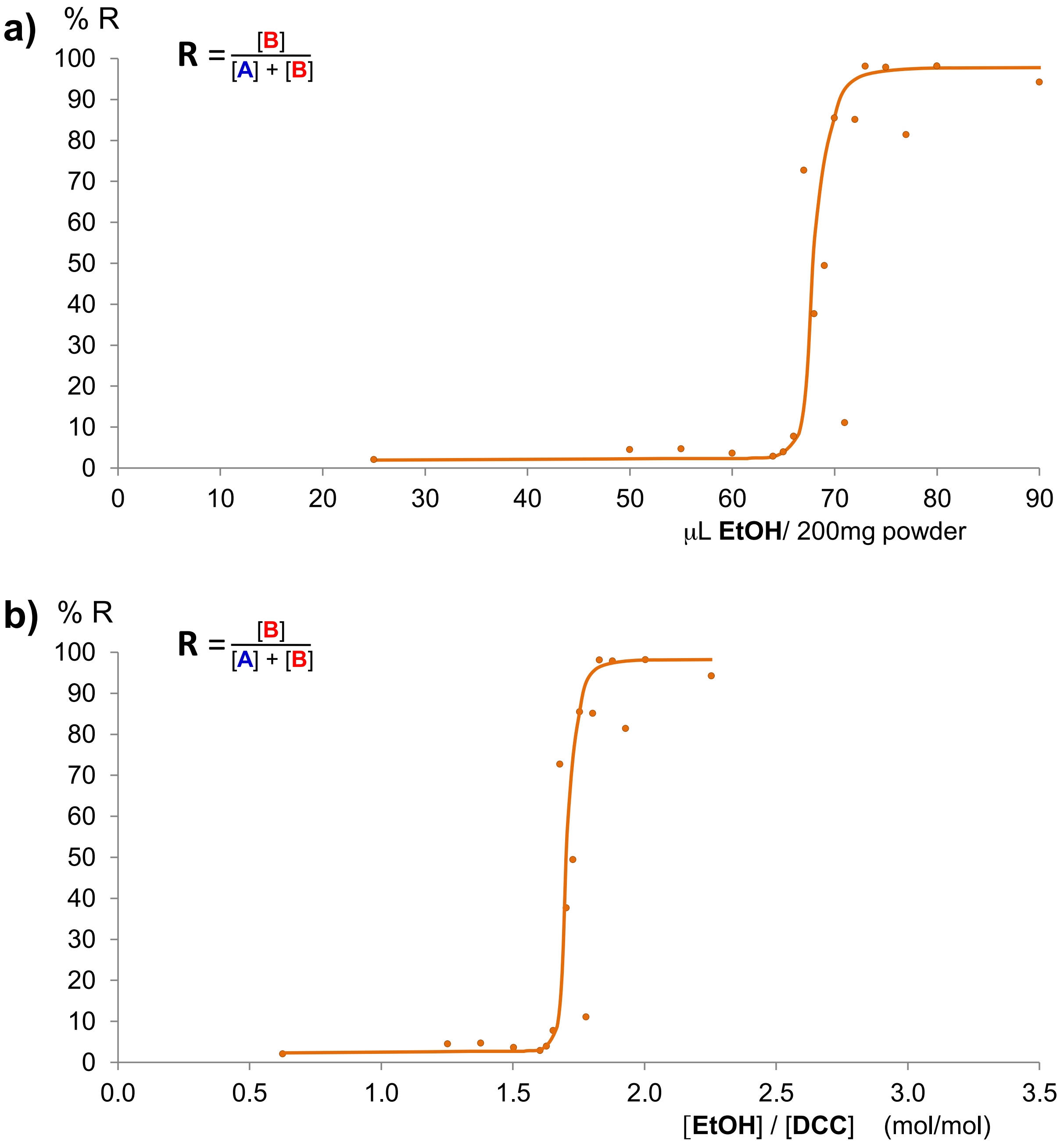

[**DCC**] is the concentration of the dynamic covalent chemistry reaction at any time = [**1-1**]+[**2-2**]+[**A**]+[**B**]
[**A**] is the concentration of **Form A** in DCC; [**B**] is the concentration of **Form B** in DCC

Figure S 56 Experimental milling equilibrium curves using "ball mill grinding procedure 4" plotted as the concentration of ethanol versus %R index. R is the ratio of the concentration of **Form B** with respect to the total concentration of **Form B** plus **Form A** formed in the solid state DCC reaction for each specific experimental point. **a)** Curve expressed as μL Ethanol / 200 mg powder and **b)** curve expressed as mols of ethanol added to the reaction with respect to 0.68 mmol of DCC library; this library is composed by the homodimers **1-1** and **2-2** and the heterodimers **Form A** and **Form B**. R is calculated from the Rietveld refinement of PXRD data giving the phase composition of the solid-state DCC experiment at equilibrium as %M. Each point in the graph is an individual experiment. No fitting was performed and the curves drawn are only a guide to the eye.

8.13 LAG experiments using isopropanol as grinding liquid on solid-state DCC reaction (Procedure 1)

These experiments were performed by adding the solvent by reverse pipetting using Procedure 1 (Section 4.5.1).

8.13.1 Experimental details and HPLC data (Procedure 1)

Table S 54 Experimental details of ball mill LAG experiments with **IPA** as grinding solvent used to prepare the milling equilibrium curves and corresponding HPLC data. Tabulation of weights (mg) of starting materials (**1-1** & **2-2**), volume of **IPA** added to the solid state DCC reaction and the corresponding grinding time used to ensure that thermodynamic equilibrium is achieved for each single experimental point. The chemical composition of the reaction product as %M obtained by HPLC analysis is tabulated. Details of HPLC method are included.

Ball mill LAG taken to thermodynamic equilibrium using Procedure 1

2%M dbu + µL **IPA** added to 0.68 mmol **DCC** ([**1-1**] + [**2-2**] + [**1-2**])

Reagents:	$(2NO_2PhS)_2$	$(4ClPhS)_2$	IPA added		**HPLC conditions**		
MW:	308.33	287.23	to powder		Zorbax XDB C18, 1.8 µm; 4.6x50 mm		
%M initial	50%M	50%M	MW	Densiity	A: H_2O+0.1% FA; B: MeCN+0.1%FA		
mmol	0.34	0.34	(g/mol)	(g/mL)	0-2min 75-85%B;		
mg	104.8 mg	97.6 mg	60.10	0.7815	2ml/min; 60°C; 259 nm (8nm bandwidth		
					**HPLC results**		
grinding time @ 30 Hz to equilibrium	$(2NO_2PhS)_2$ weighed (mg)	$(4ClPhS)_2$ weighed (mg)	IPA µL	[IPA]/[DCC] (mol/mol)	$(2NO_2PhS)_2$ (1-1) %M	Product (1-2) %M	$(4ClPhS)_2$ (2-2) %M
4h	104.77	97.69	**50**	**0.96**	1.40	**97.3**	1.3
4h	104.83	97.65	**70**	**1.34**	3.60	**92.3**	4.1
5h	104.81	97.66	**75**	**1.43**	1.70	**97.0**	1.3
3h	104.79	97.70	**80**	**1.53**	2.80	**94.7**	2.5
5h	104.85	97.72	**85**	**1.63**	1.80	**96.7**	1.5
5h	104.86	97.70	**90**	**1.72**	2.00	**96.3**	1.7
5h	104.82	97.69	**95**	**1.82**	0.60	**98.7**	0.7
4h	104.80	97.60	**100**	**1.91**	3.00	**94.4**	1.8
5h	104.82	97.67	**105**	**2.01**	2.00	**96.2**	1.8
4h	104.76	97.67	**110**	**2.10**	3.00	**94.5**	4.5
4h	104.80	97.63	**120**	**2.29**	4.9	**90.5**	1.6
5h	104.86	97.71	**140**	**2.68**	1.3	**97.1**	1.6

[DCC] is the concentration of the dynamic covalent chemistry reaction at any time =[1-1]+[2-2]+[1-2]

[1-2] = **Form A** + **Form B**

8.13.2 **PXRD scans, tabulation of Rietveld refinement of PXRD data and corresponding experimental milling equilibrium curves (Procedure 1)**

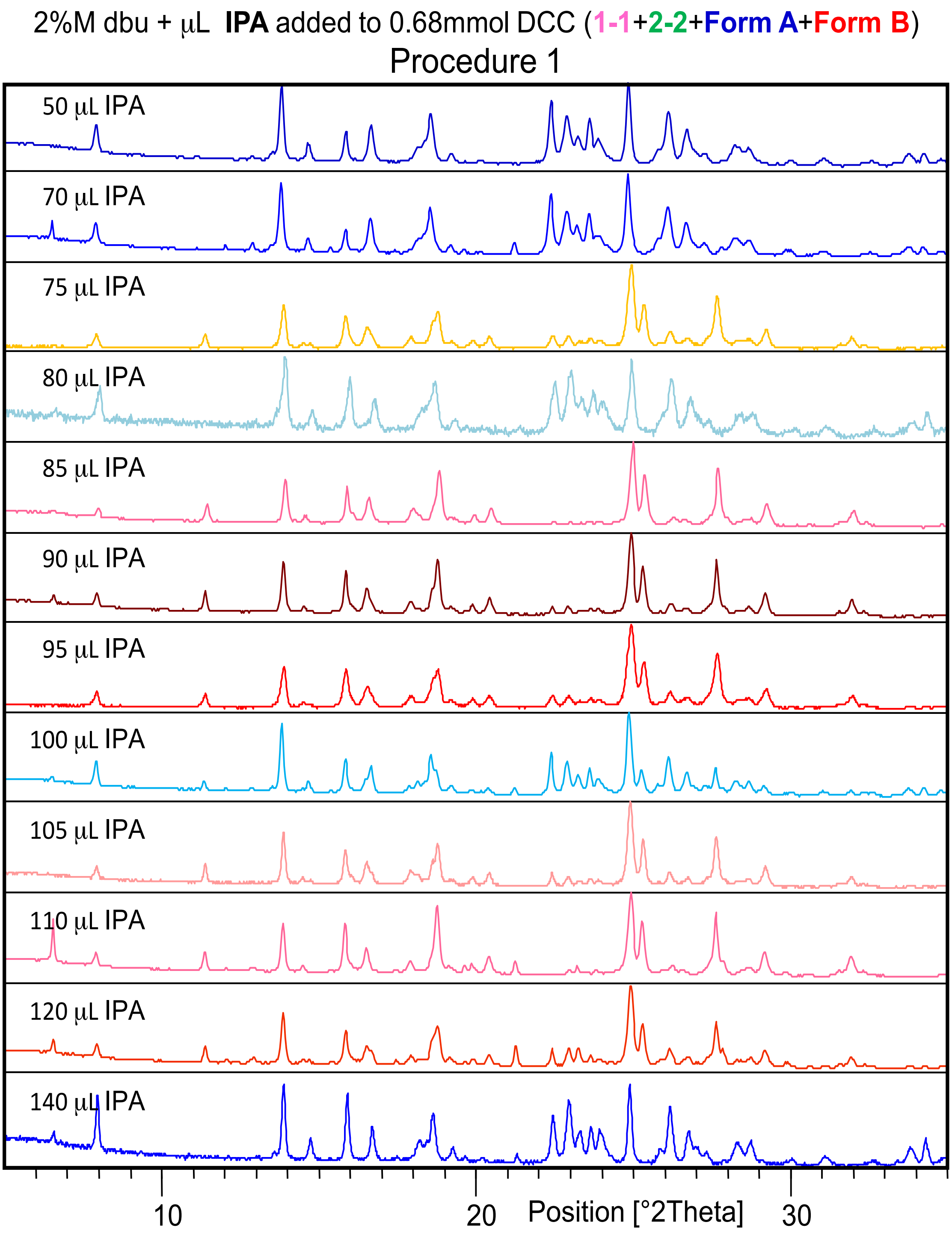

PXRD scan 16 Ball mill LAG reaction at equilibrium with different volumes of **IPA**. Each PXRD scan was obtained at equilibrium from a single point experiment with the specified added volume of **IPA**. The PXRD sample was prepared immediately after completion of the grinding experiment, and scanned between 5 and 45° (2θ).

Table S 55 Tabulation of the phase composition and corresponding R index used to prepare the milling equilibrium curve with **IPA** as the LAG solvent using ball mill grinding **Procedure 1**. The phase composition was calculated by Rietveld refinement of PXRD data as %M of the mixture of starting materials (**1-1** and **2-2**) and the polymorphs of the product (**Form A** and **Form B**). The corresponding refinement errors (esd) have been calculated as %mol. The R index is calculated as the ratio between the concentration of **Form B** versus the total concentration of **Form B** plus **Form A**.

Ball mill LAG grinding reaction taken to thermodynamic equilibrium using Procedure 1

2%M dbu+μL IPA added to 0.68 mmol DCC ([1-1]+[2-2]+[Form A]+[Form B])

grinding time @ 30Hz to equilib.	IPA added to powder		1-1		2-2		Form B		Form A		[A]+[B]	R = [B] / ([A]+[B])
	IPA μL	[IPA]/[DCC] (mol/mol)	%M	e.s.d. mol%	%M	e.s.d. mol%	%M	e.s.d. mol%	%M	e.s.d. mol%	total polymorph	% R
4h	**50**	**0.96**	**1.6**	0.4	**1.3**	0.3	**3.5**	0.5	**93.7**	0.7	97.2	**3.6**
4h	**70**	**1.34**	**6.2**	0.3	**5.0**	0.3	**3.0**	0.4	**85.8**	0.6	88.8	**3.4**
5h	**75**	**1.43**	**1.5**	0.3	**1.2**	0.3	**71.5**	0.5	**25.7**	0.5	97.2	**73.6**
3h	**80**	**1.53**	**5.4**	1.2	**4.3**	0.9	**6.9**	1.5	**83.5**	1.9	90.4	**7.6**
5h	**85**	**1.63**	**1.3**	0.7	**1.1**	0.6	**94.7**	1.2	**2.9**	0.9	97.6	**97.0**
5h	**90**	**1.72**	**1.6**	0.4	**1.3**	0.3	**84.8**	0.7	**12.4**	0.6	97.2	**87.2**
5h	**95**	**1.82**	**1.5**	0.3	**1.2**	0.3	**74.4**	0.6	**22.9**	0.5	97.3	**76.5**
4h	**100**	**1.91**	**3.5**	0.4	**2.8**	0.3	**32.2**	0.5	**61.4**	0.5	93.6	**34.4**
5h	**105**	**2.01**	**1.3**	0.5	**1.0**	0.4	**76.3**	0.7	**21.4**	0.6	97.7	**78.1**
4h	**110**	**2.10**	**7.5**	0.3	**6.1**	0.3	**85.8**	0.4	**0.6**	0.2	86.4	**99.3**
4h	**120**	**2.29**	**9.2**	0.4	**7.4**	0.3	**60.9**	0.6	**22.4**	0.5	83.3	**73.1**
5h	**140**	**2.68**	**3.6**	0.5	**2.8**	0.4	**6.2**	0.8	**87.4**	0.9	93.6	**6.6**

[DCC] is the concentration of the dynamic covalent chemistry reaction at any time =[**1-1**]+[**2-2**]+[**Form A**]+[**Form B**]

[**1-2**] = **Form A** + **Form B** [**A**] ≡ [**Form A**] [**B**] ≡ [**Form B**]

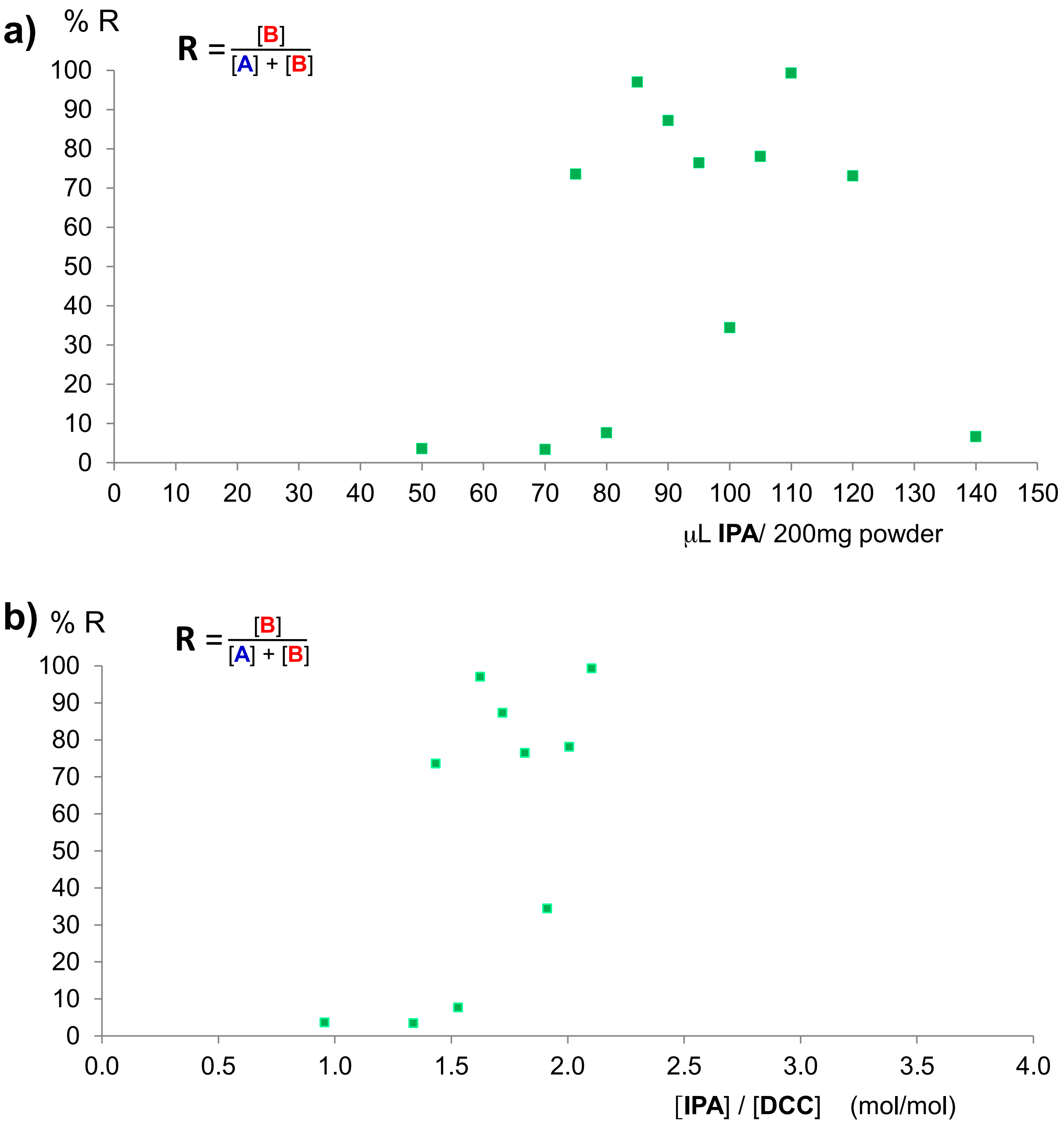

[**DCC**] is the concentration of the dynamic covalent chemistry reaction at any time = [**1-1**]+[**2-2**]+[**A**]+[**B**]

[**A**] is the concentration of **Form A** in DCC; [**B**] is the concentration of **Form B** in DCC

Figure S 57 Experimental milling equilibrium curves using "ball mill grinding procedure 1" plotted as the concentration of IPA versus %R index. R is the ratio of the concentration of **Form B** with respect to the total concentration of **Form B** plus **Form A** formed in the solid state DCC reaction for each specific experimental point. **a)** Curve expressed as μL IPA / 200 mg powder and **b)** curve expressed as mols of IPA added to the reaction with respect to 0.68 mmol of DCC library; this library is composed by the homodimers **1-1** and **2-2** and the heterodimers **Form A** and **Form B**. R is calculated from the Rietveld refinement of PXRD data giving the phase composition of the solid-state DCC experiment at equilibrium as %M. Each point in the graph is an individual experiment.

8.14 LAG experiments using isopropanol as grinding liquid on solid-state DCC reaction (Procedure 4)

These experiments were performed by adding the solvent by normal pipetting using Procedure 4 (Section 4.5.4).

8.14.1 Accuracy and precision of dispensing volume of IPA (Procedure 4)

Table S 56 Tabulation of accuracy and precision of IPA dispensed, calibrated by weighing experiments

Density:	0.7815		24.0°C		bp:	82.6°C					
Dispensed volume (μL)	**NORMAL PIPETTING** aspiration speed = 1, dispensing speed = 1						Average weight (mg)	Std deviation	Relative Std dev	volume IPA (μL)	Accuracy error (%)
	1	2	3	4							
10	7.75	8.15	7.96	7.96	7.69		7.90	0.18	2.34	10.11	1.10
20	15.72	15.88	15.75				15.78	0.09	0.54	20.20	0.97
30	23.78	23.76	23.65				23.73	0.07	0.29	30.36	1.20
40	31.52	31.52	31.18	31.38			31.40	0.16	0.51	40.18	0.45
50	39.49	39.30	39.67				39.49	0.19	0.47	50.53	1.04
60	47.36	47.20	47.40				47.32	0.11	0.22	60.55	0.91
70	55.27	55.29	55.14				55.23	0.08	0.15	70.68	0.96

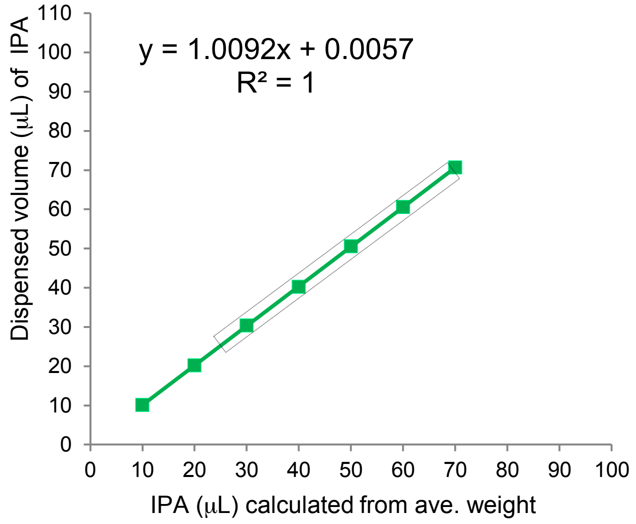

Figure S 58 Accuracy and precision of IPA dispensed, calibrated by weighing experiments. Range 10-70μL IPA

8.14.2 Experimental details and HPLC data (Procedure 4)

Table S 57 Experimental details of ball mill LAG experiments with **IPA** as grinding solvent used to prepare the milling equilibrium curves and corresponding HPLC data. Tabulation of weights (mg) of starting materials (**1-1** & **2-2**), volume of **IPA** added to the solid state DCC reaction and the corresponding grinding time used to ensure that thermodynamic equilibrium is achieved for each single experimental point. The chemical composition of the reaction product as %M obtained by HPLC analysis is tabulated. Details of HPLC method are included.

Ball mill LAG grinding reaction taken to thermodynamic equilibrium using Procedure 4								
2%M dbu + **μL IPA** added to 0.68 mmol **DCC** ([**1-1**] + [**2-2**] + [**1-2**])								
Reagents:		**$(2NO_2PhS)_2$**	**$(4ClPhS)_2$**	IPA added to powder		**HPLC conditions**		
MW:		308.33	287.23			Zorbax XDB C18, 1.8 μm; 4.6x50 mm		
%M initial		50%M	50%M	MW	Densiity	A: H_2O+0.1% FA; B: MeCN+0.1%FA		
mmol		0.34	0.34	(g/mol)	(g/mL)	0-2min 75-85%B;		
mg		104.8 mg	97.6 mg	60.10	0.7815	2ml/min; 60°C; 259 nm (8nm bandwidth)		
						**HPLC results**		
pre-soaking time	grinding time @ 30 Hz to equilibrium	$(2NO_2PhS)_2$ weighed (mg)	$(4ClPhS)_2$ weighed (mg)	IPA μL	[IPA]/[DCC] (mol/mol)	$(2NO_2PhS)_2$ **(1-1)** %M	Product **(1-2)** %M	$(4ClPhS)_2$ **(2-2)** %M
1h45m	4h	104.81	97.64	**25**	**0.48**	1.2	**97.9**	1.4
45 m	3	104.81	97.67	**50**	**0.96**	1.2	**97.7**	1.1
45 m	5h	104.86	97.65	**65**	**1.24**	1.1	**97.7**	1.3
45 m	3.5h	104.83	97.66	**70**	**1.34**	0.6	**99.0**	0.4
45 m	3.5h	104.80	97.70	**75**	**1.43**	0.6	**98.6**	0.8
45 m	3h	104.81	97.65	**76**	**1.45**	0.9	**98.2**	0.9
45 m	3h	104.85	97.68	**77**	**1.47**	0.8	**98.4**	0.8
45 m	3h	104.82	97.63	**78**	**1.49**	1.5	**96.9**	1.6
45 m	3h	104.86	97.63	**79**	**1.51**	1.5	**97.0**	1.5
45 m	3h30m	104.82	97.65	**80**	**1.53**	2.0	**96.4**	1.6
[DCC] is the concentration of the dynamic covalent chemistry reaction at any time =[1-1]+[2-2]+[1-2]								
[1-2] = **Form A** + **Form B**								

8.14.3 PXRD scans, tabulation of Rietveld refinement of PXRD data and corresponding experimental milling equilibrium curves (Procedure 4)

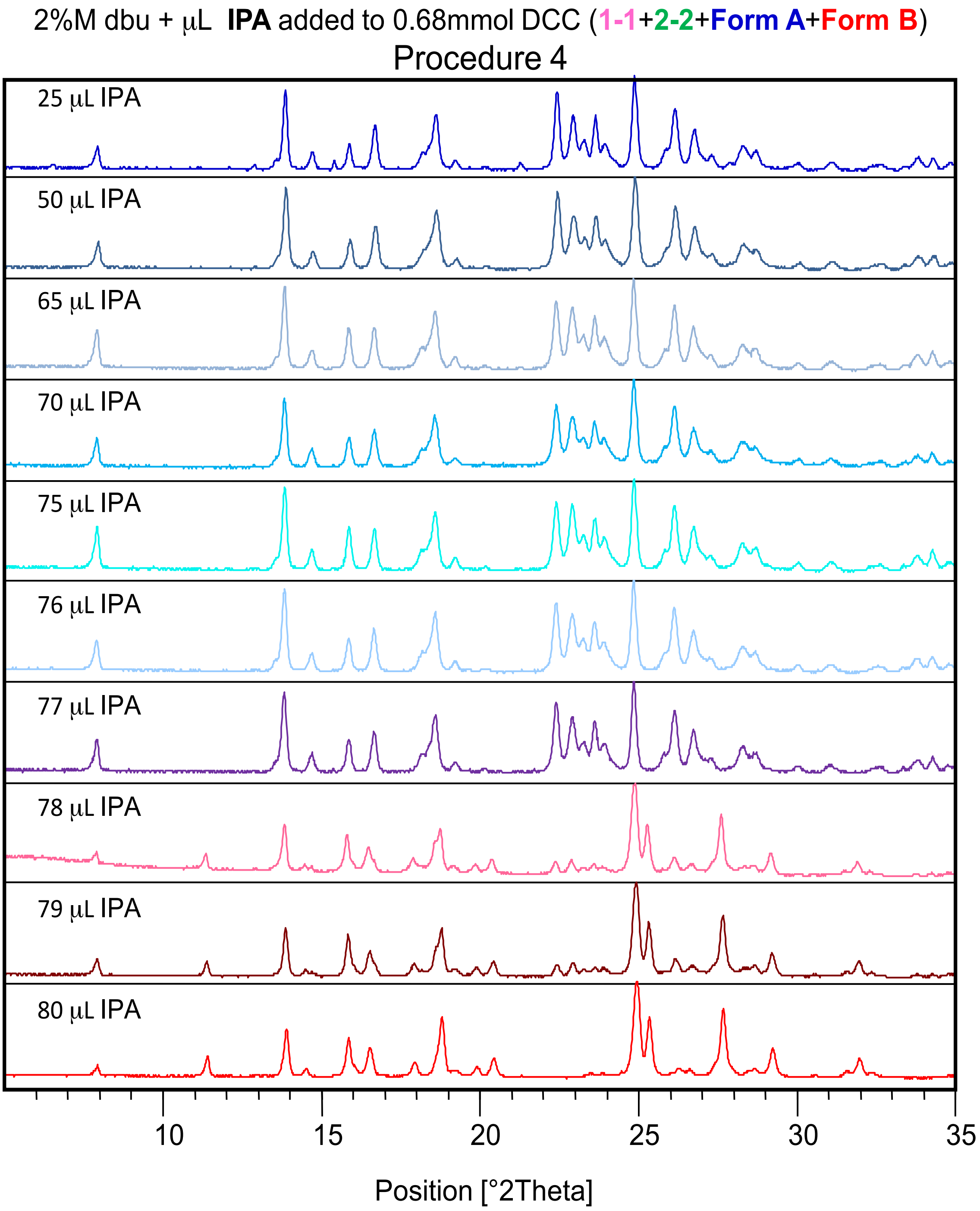

PXRD scan 17 Ball mill LAG reaction at equilibrium with different volumes of **IPA**. Each PXRD scan was obtained at equilibrium from a single point experiment with the specified added volume of **IPA**. The PXRD sample was prepared immediately after completion of the grinding experiment, and scanned between 5 and 45° (2θ).

Table S 58 Tabulation of the phase composition and corresponding R index used to prepare the milling equilibrium curve with **IPA** as the LAG solvent using ball mill grinding **Procedure 4**. The phase composition was calculated by Rietveld refinement of PXRD data as %M of the mixture of starting materials (**1-1** and **2-2**) and the polymorphs of the product (**Form A** and **Form B**). The corresponding refinement errors (esd) have been calculated as %mol. The R index is calculated as the ratio between the concentration of **Form B** versus the total concentration of **Form B** plus **Form A**.

Ball mill LAG grinding reaction taken to thermodynamic equilibrium using Procedure 4													
2%M dbu+μL IPA added to 0.68 mmol DCC ([1-1]+[2-2]+[Form A]+[Form B])													
pre-soaking time	grinding time @ 30Hz to equilib.	IPA added to powder		1-1		2-2		Form B		Form A		[A]+[B]	R = [B] / ([A]+[B])
		IPA μL	[IPA]/[DCC] (mol/mol)	%M	e.s.d. mol%	%M	e.s.d. mol%	%M	e.s.d. mol%	%M	e.s.d. mol%	total polymorph	% R
1h45m	4h	**25**	**0.48**	**3.5**	0.3	**2.8**	0.2	**2.6**	0.4	**91.1**	0.5	93.7	**2.8**
45 m	3	**50**	**0.96**	**1.9**	0.5	**1.5**	0.4	**3.1**	0.4	**93.4**	0.7	96.5	**3.2**
45 m	5h	**65**	**1.24**	**1.9**	0.4	**1.5**	0.3	**4.3**	0.5	**92.3**	0.7	96.6	**4.5**
45 m	3.5h	**70**	**1.34**	**2.0**	0.7	**1.6**	0.6	**4.9**	0.6	**91.5**	1.0	96.4	**5.1**
45 m	3.5h	**75**	**1.43**	**2.3**	0.5	**1.8**	0.4	**3.9**	0.4	**91.9**	0.8	95.8	**4.1**
45 m	3h	**76**	**1.45**	**2.0**	0.5	**1.6**	0.4	**3.3**	0.4	**93.1**	0.7	96.4	**3.4**
45 m	3h	**77**	**1.47**	**1.9**	0.8	**1.5**	0.7	**3.6**	0.7	**92.9**	1.2	96.5	**3.7**
45 m	3h	**78**	**1.49**	**2.2**	0.7	**1.8**	0.5	**72.7**	0.8	**23.3**	0.5	96.0	**75.7**
45 m	3h	**79**	**1.51**	**2.1**	0.5	**1.8**	0.4	**73.7**	0.6	**22.4**	0.5	96.1	**76.7**
45 m	3h30m	**80**	**1.53**	**2.6**	0.5	**2.1**	0.4	**93.7**	0.7	**1.6**	0.3	95.3	**98.3**

[DCC] is the concentration of the dynamic covalent chemistry reaction at any time =[**1-1**]+[**2-2**]+[**Form A**]+[**Form B**]

[**1-2**] = **Form A** + **Form B** [**A**] ≡ [**Form A**] [**B**] ≡ [**Form B**]

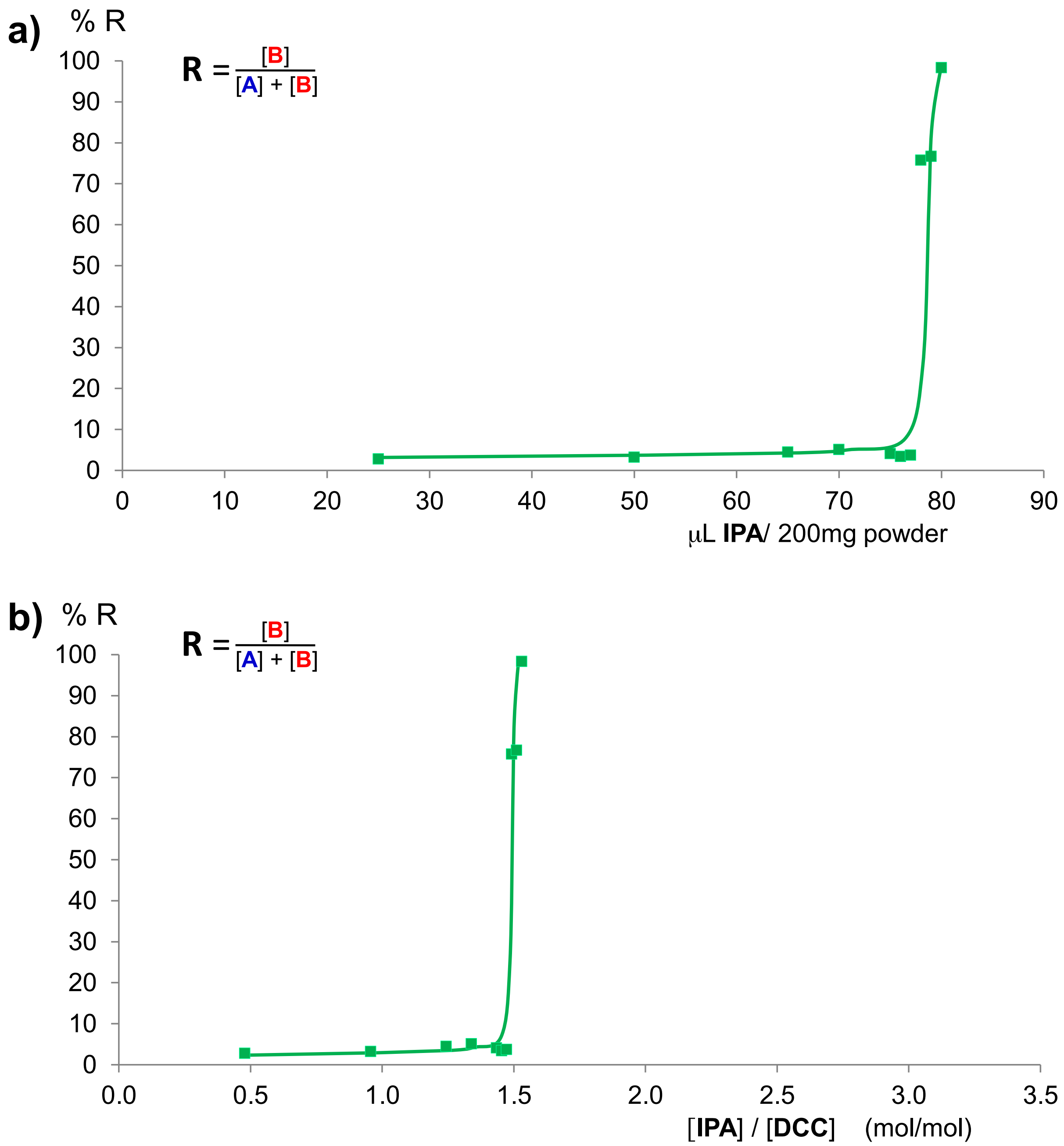

[**DCC**] is the concentration of the dynamic covalent chemistry reaction at any time = [**1-1**]+[**2-2**]+[**A**]+[**B**]
[**A**] is the concentration of **Form A** in DCC; [**B**] is the concentration of **Form B** in DCC

Figure S 59 Experimental milling equilibrium curves using "ball mill grinding procedure 4" plotted as the concentration of IPA versus %R index. R is the ratio of the concentration of **Form B** with respect to the total concentration of **Form B** plus **Form A** formed in the solid state DCC reaction for each specific experimental point. **a)** Curve expressed as μL IPA / 200 mg powder and **b)** curve expressed as mols of IPA added to the reaction with respect to 0.68 mmol of DCC library; this library is composed by the homodimers **1-1** and **2-2** and the heterodimers **Form A** and **Form B**. R is calculated from the Rietveld refinement of PXRD data giving the phase composition of the solid-state DCC experiment at equilibrium as %M. Each point in the graph is an individual experiment. No fitting was performed and the curves drawn are only a guide to the eye.

8.15 LAG experiments using water as grinding liquid on solid-state DCC reaction (Procedure 5)

These experiments were performed by adding the solvent by normal pipetting using Procedure 5 (Section 4.5.5).

8.15.1 Experimental details and HPLC data (Procedure 5)

Table S 59 Experimental details of ball mill LAG experiments with **water** as grinding solvent used to prepare the milling equilibrium curves and corresponding HPLC data. Tabulation of weights (mg) of starting materials (**1-1** & **2-2**), volume of **water** added to the solid state DCC reaction and the corresponding grinding time used to ensure that thermodynamic equilibrium is achieved for each single experimental point. The chemical composition of the reaction product as %M obtained by HPLC analysis is tabulated. Details of HPLC method are included.

Ball mill LAG grinding reaction taken to thermodynamic equilibrium using Procedure 5								
2%M dbu + µL H_2O added to 0.68 mmol **DCC** ([**1-1**] + [**2-2**] + [**1-2**])								
Reagents:		**$(2NO_2PhS)_2$**	**$(4ClPhS)_2$**	H_2O added		**HPLC conditions**		
MW:		308.33	287.23	to powder		Zorbax XDB C18, 1.8 µm; 4.6x50 mm		
%M initial		50%M	50%M	MW	Densiity	A: H_2O+0.1% FA; B: MeCN+0.1%FA		
mmol		0.34	0.34	(g/mol)	(g/mL)	0-2min 75-85%B;		
mg		104.8 mg	97.6 mg	18.02	0.9974	2ml/min; 60°C; 259 nm (8nm bandwidth)		
						**HPLC results**		
pre-soaking time	grinding time @ 30 Hz to equilibrium	$(2NO_2PhS)_2$ weighed (mg)	$(4ClPhS)_2$ weighed (mg)	H_2O µL	$[H_2O]/[DCC]$ (mol/mol)	$(2NO_2PhS)_2$ (**1-1**) %M	Product (**1-2**) %M	$(4ClPhS)_2$ (**2-2**) %M
60 m	1h20m	104.83	97.65	**25**	**2.03**	0.5	**98.5**	1.0
45 m	1h30m	104.83	97.68	**35**	**2.85**	0.9	**97.7**	1.4
2h30m	3h	104.81	97.67	**50**	**4.07**	8.4	**83.5**	8.1
1h15m	2h	104.84	97.71	**70**	**5.70**	3.9	**92.4**	3.7
60 m	3h	104.84	97.65	**90**	**7.33**	3.1	**93.9**	3.0
50 m	3h	104.86	97.68	**100**	**8.14**	2.1	**96.1**	1.8
1h30m	3h	104.86	97.65	**130**	**10.58**	4.3	**91.8**	3.9
1h30m	3h30m	104.81	97.69	**160**	**13.02**	15.0	**71.6**	13.5
3h15m	5h	104.80	97.68	**200**	**16.28**	20.6	**60.2**	19.2
[DCC] is the concentration of the dynamic covalent chemistry reaction at any time =[1-1]+[2-2]+[1-2]								
[1-2] = **Form A** + **Form B**								

8.15.2 PXRD scans, tabulation of Rietveld refinement of PXRD data and corresponding experimental milling equilibrium curves (Procedure 5)

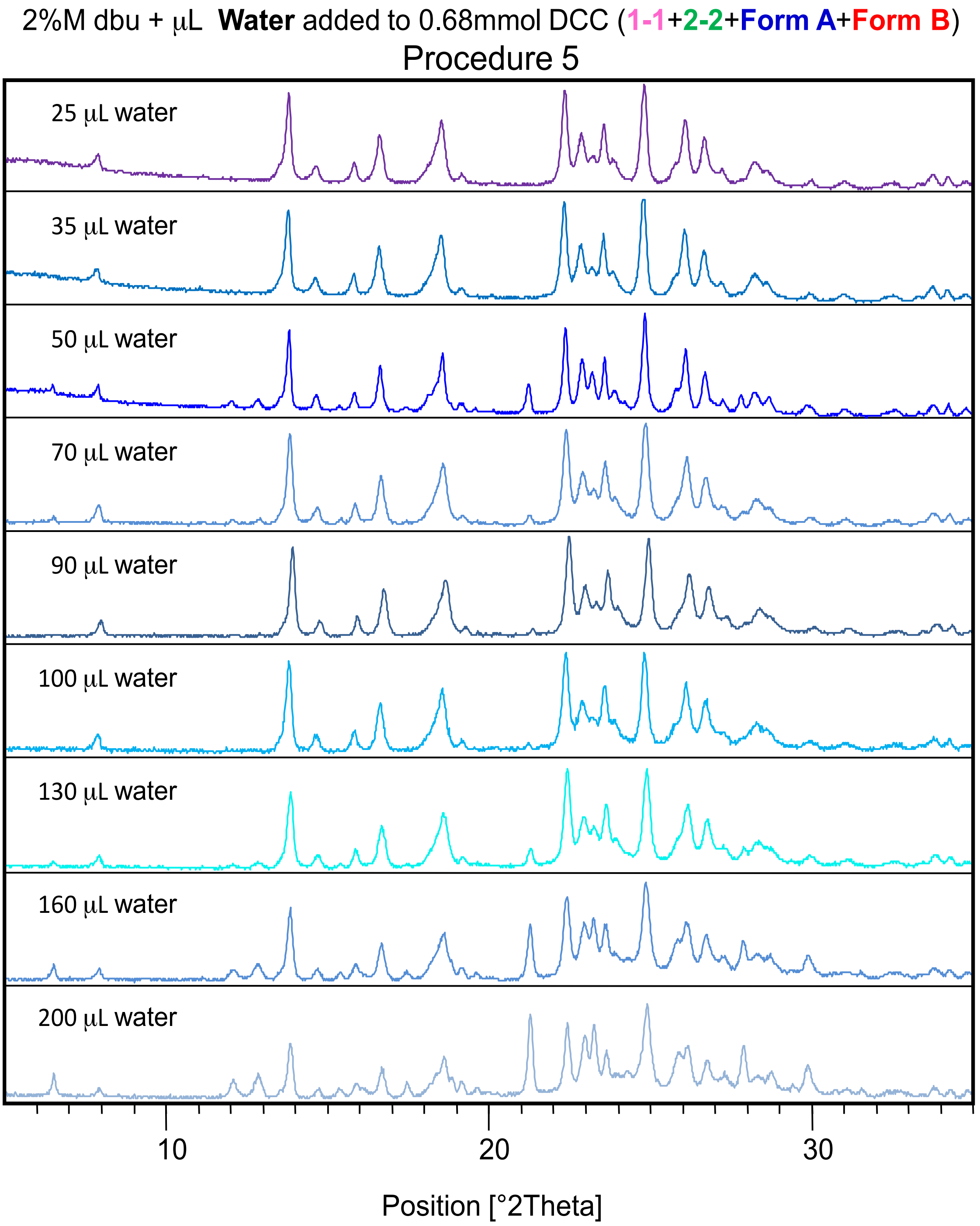

PXRD scan 18 Ball mill LAG reaction at equilibrium with different volumes of **water**. Each PXRD scan was obtained at equilibrium from a single point experiment with the specified added volume of **water**. The PXRD sample was prepared immediately after completion of the grinding experiment, and scanned between 5 and 45° (2θ).

Table S 60 Tabulation of the phase composition and corresponding R index used to prepare the milling equilibrium curve with **water** as the LAG solvent using ball mill grinding **Procedure 5**. The phase composition was calculated by Rietveld refinement of PXRD data as %M of the mixture of starting materials (**1-1** and **2-2**) and the polymorphs of the product (**Form A** and **Form B**). The corresponding refinement errors (esd) have been calculated as %mol. The R index is calculated as the ratio between the concentration of **Form B** versus the total concentration of **Form B** plus **Form A**.

Ball mill LAG grinding reaction taken to thermodynamic equilibrium using Procedure 5													
2%M dbu+μL water added to 0.68 mmol DCC ([1-1]+[2-2]+[Form A]+[Form B])													
pre-soaking time	grinding time @ 30Hz to equilib.	water added to powder		1-1		2-2		Form B		Form A		[A]+[B]	R = [B] / ([A]+[B])
		water μL	[water]/[DCC] (mol/mol)	%M	e.s.d. mol%	%M	e.s.d. mol%	%M	e.s.d. mol%	%M	e.s.d. mol%	total polymorph	% R
60 m	1h20m	**25**	**2.03**	**1.5**	0.4	**1.2**	0.3	**5.8**	0.6	**91.5**	0.8	97.3	**6.0**
45 m	1h30m	**35**	**2.85**	**1.6**	0.4	**1.3**	0.3	**4.7**	0.6	**92.5**	0.8	97.2	**4.8**
2h30m	3h	**50**	**4.07**	**10.3**	0.4	**8.3**	0.3	**2.8**	0.5	**78.6**	0.6	81.4	**3.4**
1h15m	2h	**70**	**5.70**	**3.6**	0.3	**2.9**	0.2	**3.7**	0.4	**89.8**	0.5	93.5	**4.0**
60 m	3h	**90**	**7.33**	**1.9**	0.3	**1.6**	0.2	**3.8**	0.4	**92.7**	0.5	96.5	**3.9**
50 m	3h	**100**	**8.14**	**1.2**	0.3	**0.9**	0.2	**3.5**	0.7	**94.5**	0.8	98.0	**3.6**
1h30m	3h	**130**	**10.58**	**6.1**	0.3	**4.9**	0.3	**2.3**	0.4	**86.7**	0.5	89.0	**2.6**
1h30m	3h30m	**160**	**13.02**	**17.4**	0.5	**14.0**	0.4	**3.8**	0.6	**64.7**	0.7	68.5	**5.5**
3h15m	5h	**200**	**16.28**	**25.5**	0.6	**20.5**	0.5	**1.9**	0.6	**52.1**	0.7	54.0	**3.5**
[DCC] is the concentration of the dynamic covalent chemistry reaction at any time =[1-1]+[2-2]+[Form A]+[Form B]													
[1-2] = Form A + Form B [A] ≡ [Form A] [B] ≡ [Form B]													

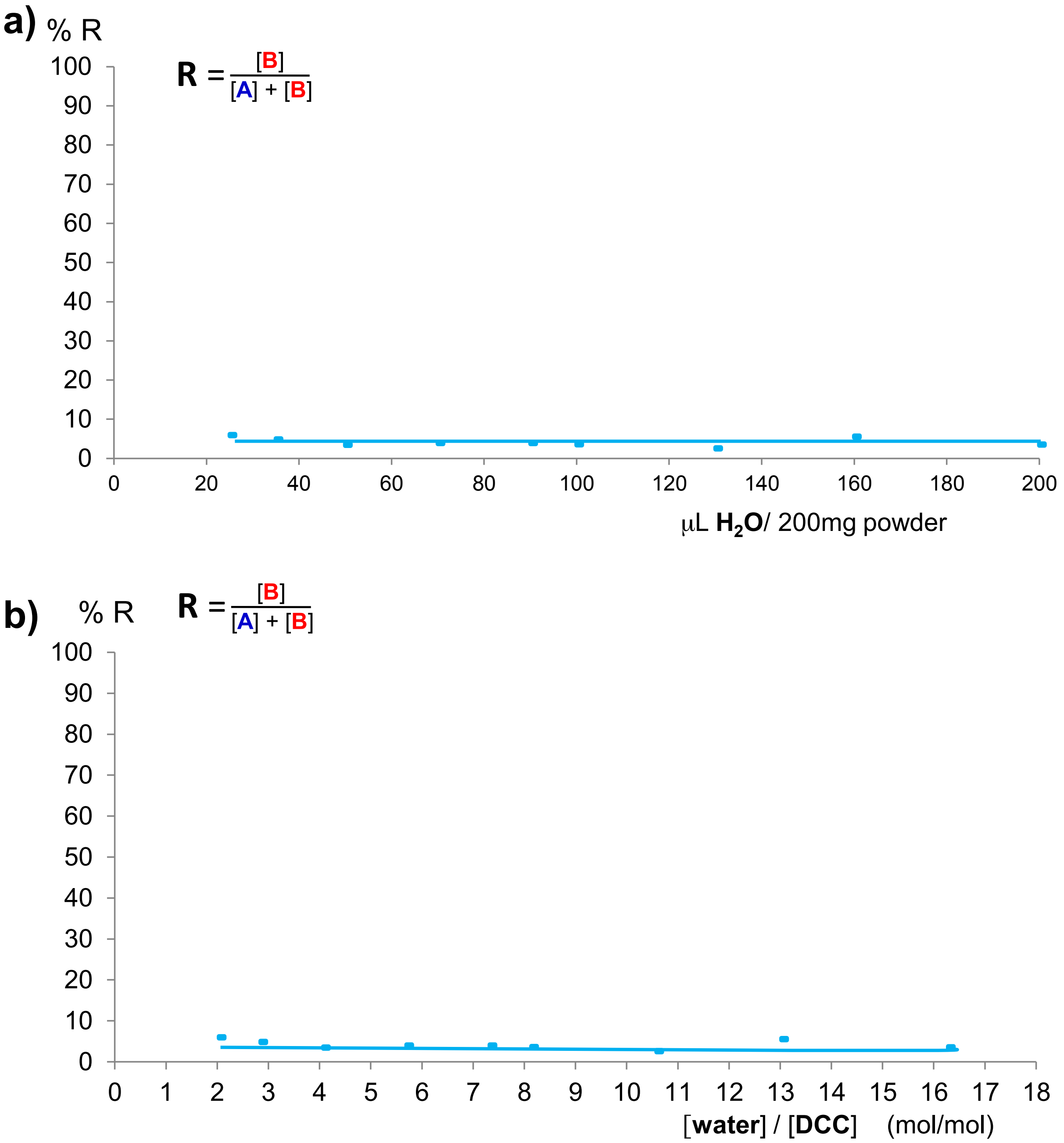

[**DCC**] is the concentration of the dynamic covalent chemistry reaction at any time = [**1-1**]+[**2-2**]+[**A**]+[**B**]
[**A**] is the concentration of **Form A** in DCC; [**B**] is the concentration of **Form B** in DCC

Figure S 60 Experimental milling equilibrium curves using "ball mill grinding procedure 5" plotted as the concentration of water versus %R index. R is the ratio of the concentration of **Form B** with respect to the total concentration of **Form B** plus **Form A** formed in the solid state DCC reaction for each specific experimental point. **a)** Curve expressed as μL water / 200 mg powder and **b)** curve expressed as mols of water added to the reaction with respect to 0.68 mmol of DCC library; this library is composed by the homodimers **1-1** and **2-2** and the heterodimers **Form A** and **Form B**. R is calculated from the Rietveld refinement of PXRD data giving the phase composition of the solid-state DCC experiment at equilibrium as %M. Each point in the graph is an individual experiment. No fitting was performed and the curves drawn are only a guide to the eye.
.

8.16 LAG experiments using cyclohexane as grinding liquid on solid-state DCC reaction (Procedure 4)

These experiments were performed by adding the solvent by normal pipetting using Procedure 4 (Section 4.5.4).

8.16.1 Experimental details and HPLC data (Procedure 4)

Table S 61 Experimental details of ball mill LAG experiments with **cyclohexane** as grinding solvent used to prepare the milling equilibrium curves and corresponding HPLC data. Tabulation of weights (mg) of starting materials (**1-1** & **2-2**), volume of **cyclohexane** added to the solid state DCC reaction and the corresponding grinding time used to ensure that thermodynamic equilibrium is achieved for each single experimental point. The chemical composition of the reaction product as %M obtained by HPLC analysis is tabulated. Details of HPLC method are included.

Ball mill LAG grinding reaction taken to thermodynamic equilibrium using Procedure 4

2%M dbu + µL **cyclohexane** added to 0.68 mmol **DCC** ([**1-1**] + [**2-2**] + [**1-2**])

Reagents:		$(2NO_2PhS)_2$	$(4ClPhS)_2$	cyclohexane added to powder		**HPLC conditions**		
MW:		308.33	287.23			Zorbax XDB C18, 1.8 µm; 4.6x50 mm		
%M initial		50%M	50%M	MW	Densiity	A: H_2O+0.1% FA; B: MeCN+0.1%FA		
mmol		0.34	0.34	(g/mol)	(g/mL)	0-2min 75-85%B;		
mg		104.8 mg	97.6 mg	84.16	0.7739	2ml/min; 60°C; 259 nm (8nm bandwidth)		
						**HPLC results**		
pre-soaking time	grinding time @ 30 Hz to equilibrium	$(2NO_2PhS)_2$ weighed (mg)	$(4ClPhS)_2$ weighed (mg)	cHexane µL	[cHexane]/[DCC] (mol/mol)	$(2NO_2PhS)_2$ (1-1) %M	Product (1-2) %M	$(4ClPhS)_2$ (2-2) %M
1h	1h	104.81	97.68	**25**	**0.34**	0.5	**98.8**	0.7
40 m	1h10m	104.83	97.67	**35**	**0.47**	0.7	**98.3**	1.0
1h	3h	104.83	97.67	**50**	**0.68**	0.6	**97.8**	1.5
1h	1h	104.86	97.65	**70**	**0.95**	4.2	**91.4**	4.3
1h	2h	104.80	97.70	**70**	**0.95**	1.9	**96.8**	1.3
14h	5h	104.81	97.65	**100**	**1.35**	4.1	**91.6**	4.3
1h	3h	104.83	97.66	**100**	**1.35**	0.9	**98.6**	0.6
3h	5h	104.84	97.68	**200**	**2.70**	4.2	**93.2**	2.6

[DCC] is the concentration of the dynamic covalent chemistry reaction at any time =[1-1]+[2-2]+[1-2]

[1-2] = **Form A** + **Form B**

8.16.2 **PXRD scans, tabulation of Rietveld refinement of PXRD data and corresponding experimental milling equilibrium curves (Procedure 4)**

2%M dbu + μL **cyclohexane** added to 0.68mmol DCC (**1-1**+**2-2**+**Form A**+**Form B**)

Procedure 4

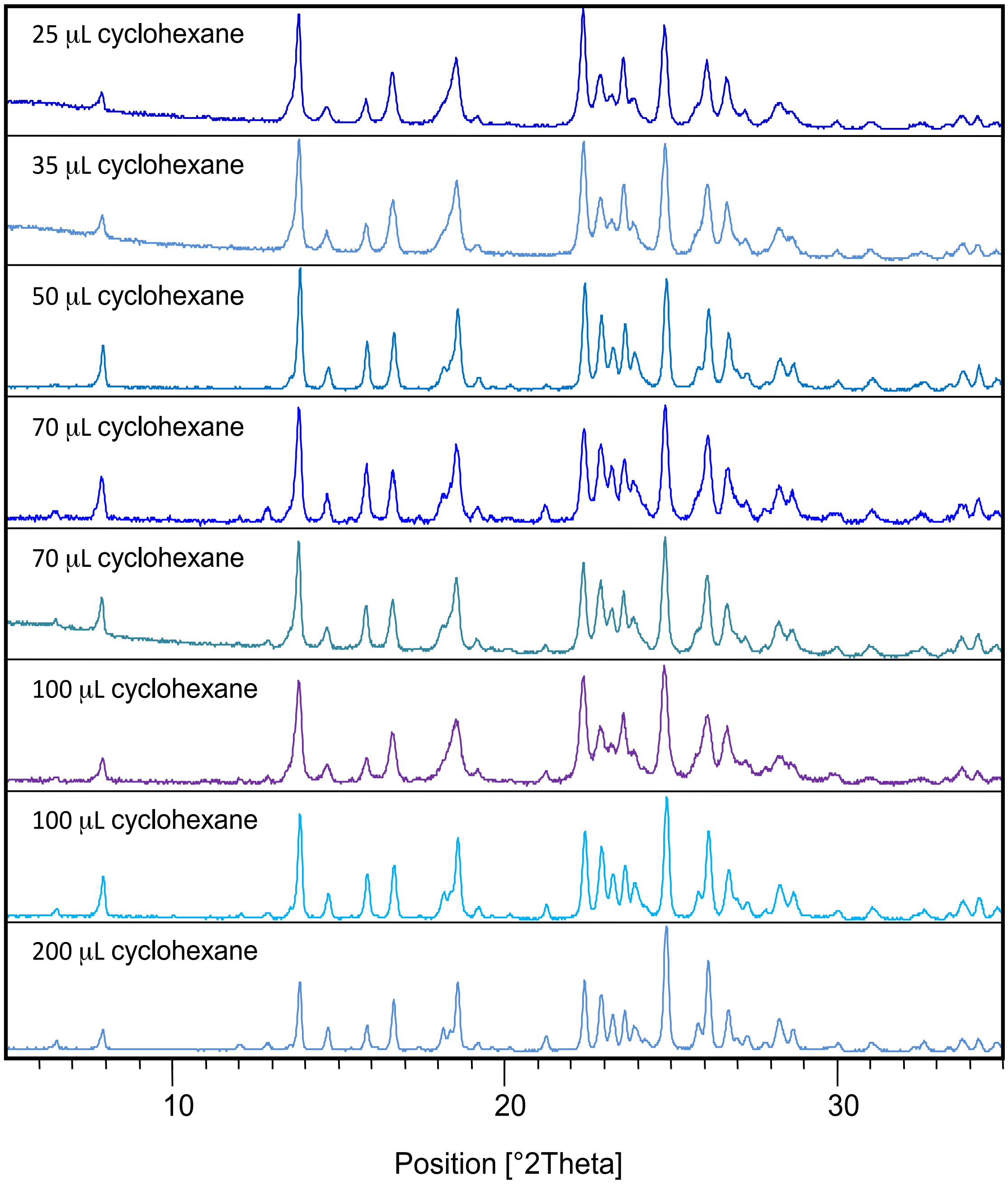

PXRD scan 19 Ball mill LAG reaction at equilibrium with different volumes of **cyclohexane**. Each PXRD scan was obtained at equilibrium from a single point experiment with the specified added volume of **cyclohexane**. The PXRD sample was prepared immediately after completion of the grinding experiment, and scanned between 5 and 45° (2θ).

Table S 62 Tabulation of the phase composition and corresponding R index used to prepare the milling equilibrium curve with **cyclohexane** as the LAG solvent using ball mill grinding **Procedure 4**. The phase composition was calculated by Rietveld refinement of PXRD data as %M of the mixture of starting materials (**1-1** and **2-2**) and the polymorphs of the product (**Form A** and **Form B**). The corresponding refinement errors (esd) have been calculated as %mol. The R index is calculated as the ratio between the concentration of **Form B** versus the total concentration of **Form B** plus **Form A**.

Ball mill LAG grinding reaction taken to thermodynamic equilibrium using Procedure 4													
2%M dbu+μL cyclohexane added to 0.68 mmol DCC ([1-1]+[2-2]+[Form A]+[Form B])													
pre-soaking time	grinding time @ 30Hz to equilib.	cyclohexane added to powder		1-1		2-2		Form B		Form A		[A]+[B]	R = [B] / ([A]+[B])
		cyclo-Hexane μL	[cHexane]/[DCC] (mol/mol)	%M	e.s.d. mol%	%M	e.s.d. mol%	%M	e.s.d. mol%	%M	e.s.d. mol%	total polymorph	% R
1h	1h	**25**	**0.34**	**1.2**	0.3	**0.9**	0.2	**4.1**	0.6	**93.9**	0.7	98.0	**4.2**
40 m	1h10m	**35**	**0.47**	**1.3**	0.4	**1.1**	0.3	**4.3**	0.6	**93.3**	0.7	97.6	**4.4**
1h	3h	**50**	**0.68**	**1.7**	0.3	**1.4**	0.3	**3.5**	0.4	**93.4**	0.6	96.9	**3.6**
1h	1h	**70**	**0.95**										
1h	2h	**70**	**0.95**	**4.2**	0.4	**3.4**	0.4	**3.7**	0.5	**88.7**	0.7	92.4	**4.0**
14h	5h	**100**	**1.35**	**4.2**	0.6	**3.4**	0.5	**7.9**	0.8	**84.4**	1.0	92.3	**8.6**
1h	3h	**100**	**1.35**	**4.5**	0.3	**3.6**	0.2	**3.4**	0.4	**88.5**	0.5	91.9	**3.7**
3h	5h	**200**	**2.70**	**7.1**	0.3	**5.7**	0.2	**1.9**	0.3	**85.2**	0.4	87.1	**2.2**

[DCC] is the concentration of the dynamic covalent chemistry reaction at any time =[**1-1**]+[**2-2**]+[**Form A**]+[**Form B**]

[**1-2**] = **Form A** + **Form B** [**A**] ≡ [**Form A**] [**B**] ≡ [**Form B**]

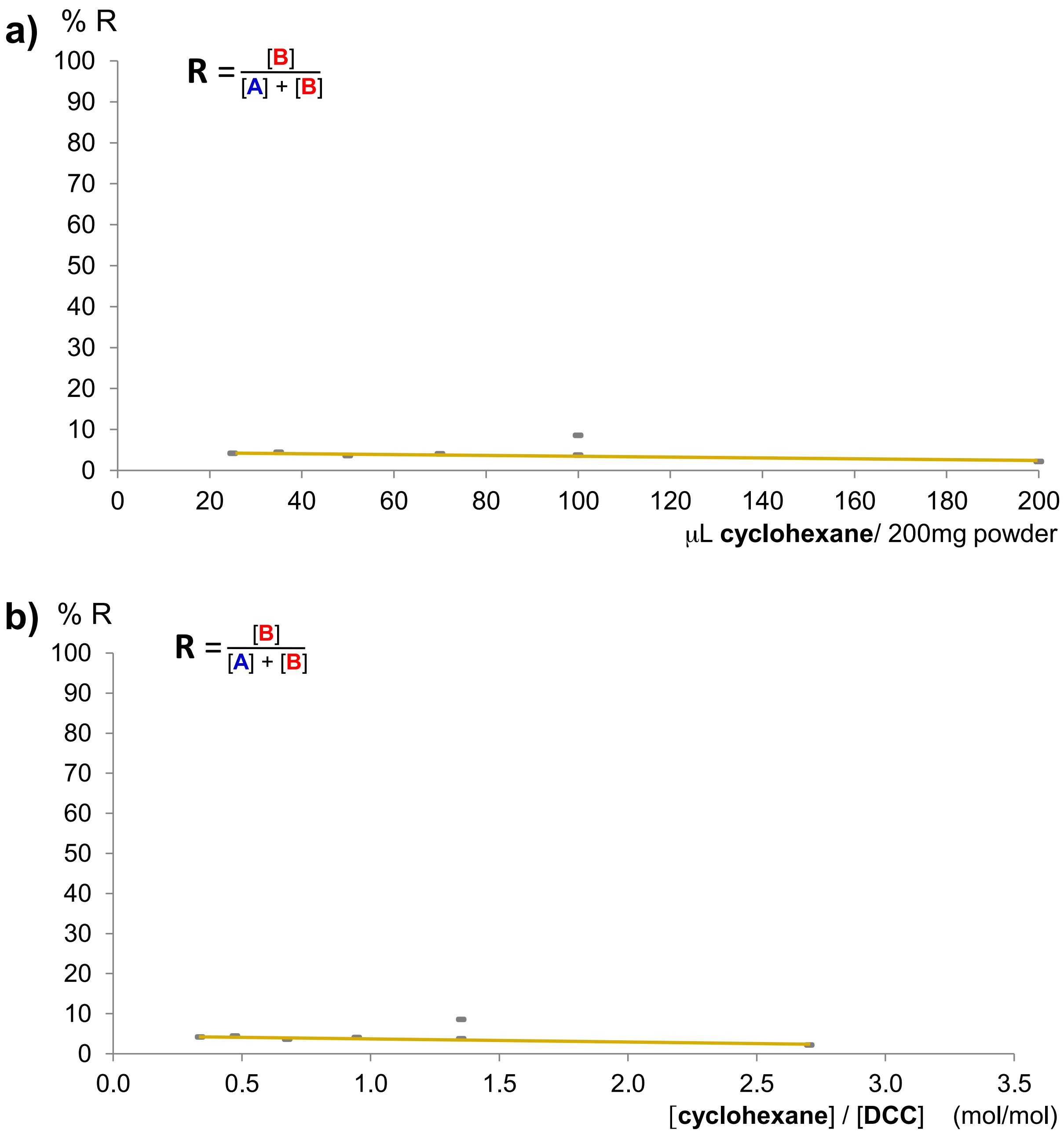

[**DCC**] is the concentration of the dynamic covalent chemistry reaction at any time = [**1-1**]+[**2-2**]+[**A**]+[**B**]
[**A**] is the concentration of **Form A** in DCC; [**B**] is the concentration of **Form B** in DCC

Figure S 61 Experimental milling equilibrium curves using "ball mill grinding procedure 4" plotted as the concentration of cyclohexane versus %R index. R is the ratio of the concentration of **Form B** with respect to the total concentration of **Form B** plus **Form A** formed in the solid state DCC reaction for each specific experimental point. **a)** Curve expressed as μL cyclohexane / 200 mg powder and **b)** curve expressed as mols of cyclohexane added to the reaction with respect to 0.68 mmol of DCC library; this library is composed by the homodimers **1-1** and **2-2** and the heterodimers **Form A** and **Form B**. R is calculated from the Rietveld refinement of PXRD data giving the phase composition of the solid-state DCC experiment at equilibrium as %M. Each point in the graph is an individual experiment. No fitting was performed and the curves drawn are only a guide to the eye.

8.17 LAG experiments using benzene as grinding liquid on solid-state DCC reaction (Procedure 4)

These experiments were performed by adding the solvent by normal pipetting using Procedure 4 (Section 4.5.4).

8.17.1 Experimental details and HPLC data (Procedure 4)

Table S 63 Experimental details of ball mill LAG experiments with **benzene** as grinding solvent used to prepare the milling equilibrium curves and corresponding HPLC data. Tabulation of weights (mg) of starting materials (**1-1** & **2-2**), volume of **benzene** added to the solid state DCC reaction and the corresponding grinding time used to ensure that thermodynamic equilibrium is achieved for each single experimental point. The chemical composition of the reaction product as %M obtained by HPLC analysis is tabulated. Details of HPLC method are included.

Ball mill LAG grinding reaction taken to thermodynamic equilibrium using Procedure 4								
2%M dbu + µL **Benzene** added to 0.68 mmol **DCC** ([**1-1**] + [**2-2**] + [**1-2**])								
Reagents:		**$(2NO_2PhS)_2$**	**$(4ClPhS)_2$**	Benzene added		**HPLC conditions**		
MW:		308.33	287.23	to powder		Zorbax XDB C18, 1.8 µm; 4.6x50 mm		
%M initial		50%M	50%M	MW	Densiity	A: H_2O+0.1% FA; B: MeCN+0.1%FA		
mmol		0.34	0.34	(g/mol)	(g/mL)	0-2min 75-85%B;		
mg		104.8 mg	97.6 mg	78.12	0.8690	2ml/min; 60°C; 259 nm (8nm bandwidth)		
						**HPLC results**		
pre-soaking time	grinding time @ 30 Hz to equilibrium	$(2NO_2PhS)_2$ weighed (mg)	$(4ClPhS)_2$ weighed (mg)	Benzene µL	[Benzene]/[DCC] (mol/mol)	$(2NO_2PhS)_2$ (1-1) %M	Product **(1-2)** %M	$(4ClPhS)_2$ (2-2) %M
30m	45m	104.81	97.65	25	0.41	1.5	**96.9**	1.5
1h	45m	104.81	97.66	25	0.41	5.0	**90.8**	4.2
45m	1h	104.81	97.67	50	0.82	14.7	**72.1**	13.1
45m	2h	104.84	97.65	50	0.82	13.9	**73.3**	12.9
1h	4h	104.81	97.70	60	0.98	19.0	**63.2**	17.8
[DCC] is the concentration of the dynamic covalent chemistry reaction at any time =[1-1]+[2-2]+[1-2]								
[1-2] = **Form A** + **Form B**								

8.17.2 PXRD scans, tabulation of Rietveld refinement of PXRD data and corresponding experimental milling equilibrium curves (Procedure 4)

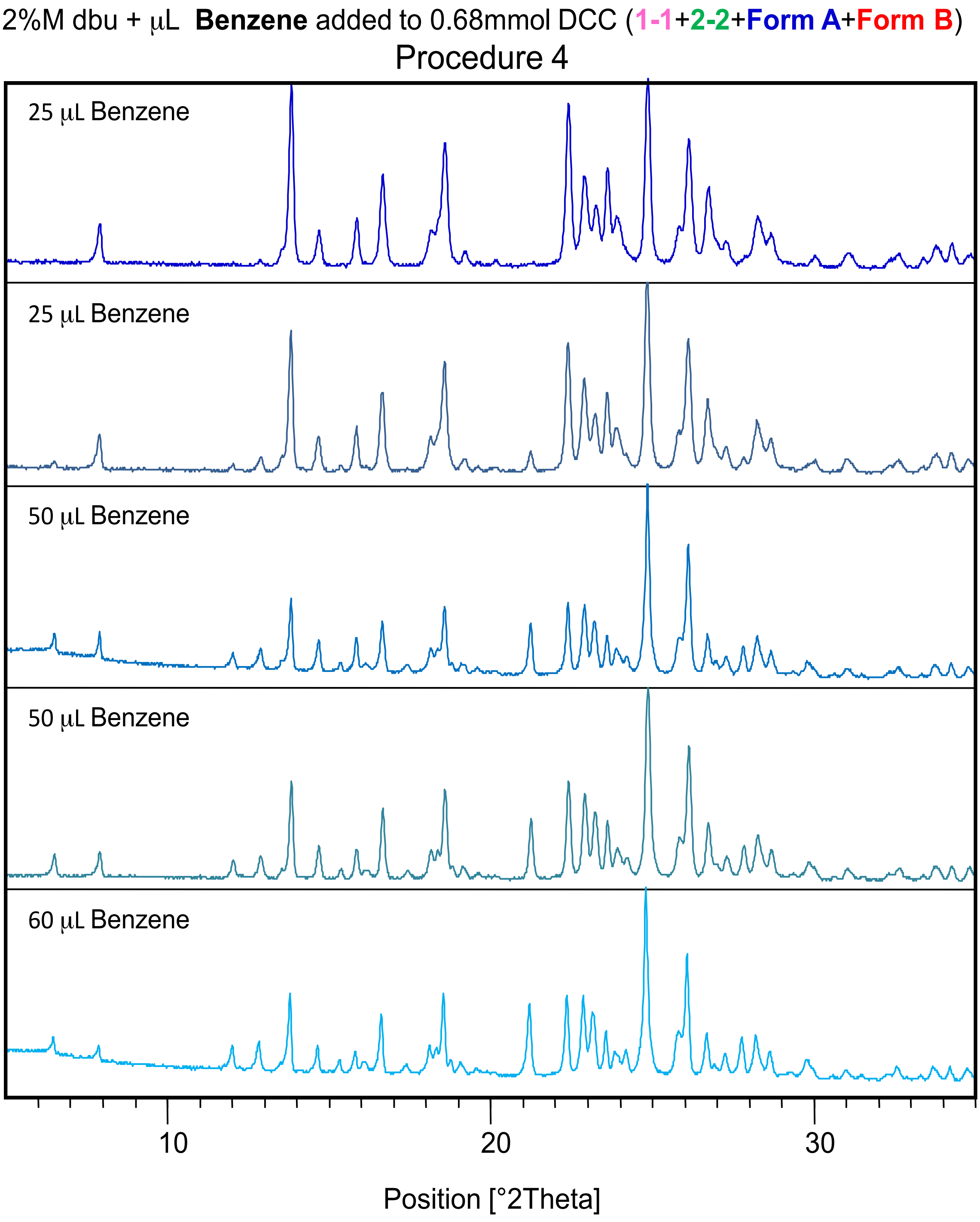

PXRD scan 20 Ball mill LAG reaction at equilibrium with different volumes of **benzene**. Each PXRD scan was obtained at equilibrium from a single point experiment with the specified added volume of **benzene**. The PXRD sample was prepared immediately after completion of the grinding experiment, and scanned between 5 and 45° (2θ).

Table S 64 Tabulation of the phase composition and corresponding R index used to prepare the milling equilibrium curve with **benzene** as the LAG solvent using ball mill grinding **Procedure 4**. The phase composition was calculated by Rietveld refinement of PXRD data as %M of the mixture of starting materials (**1-1** and **2-2**) and the polymorphs of the product (**Form A** and **Form B**). The corresponding refinement errors (esd) have been calculated as %mol. The R index is calculated as the ratio between the concentration of **Form B** versus the total concentration of **Form B** plus **Form A**.

Ball mill LAG grinding reaction taken to thermodynamic equilibrium using Procedure 4													
2%M dbu+μL Benzene added to 0.68 mmol DCC ([1-1]+[2-2]+[Form A]+[Form B])													
pre-soaking time	grinding time @ 30Hz to equilib.	Benzene added to powder		1-1		2-2		Form B		Form A		[A]+[B]	R = [B] / ([A]+[B])
		Benzene μL	[Benzene]/[DCC] (mol/mol)	%M	e.s.d. mol%	%M	e.s.d. mol%	%M	e.s.d. mol%	%M	e.s.d. mol%	total polymorph	% R
30m	45m	**25**	**0.41**	**1.4**	0.3	**1.1**	0.2	**2.6**	0.4	**94.9**	0.5	97.5	**2.7**
1h	45m	**25**	**0.41**	**5.0**	0.3	**4.0**	0.3	**2.1**	0.4	**88.8**	0.5	90.9	**2.3**
45m	1h	**50**	**0.82**	**15.7**	0.4	**12.6**	0.3	**1.5**	0.3	**70.2**	0.5	71.7	**2.1**
45m	2h	**50**	**0.82**	**14.9**	0.3	**12.0**	0.3	**1.3**	0.3	**71.8**	0.4	73.1	**1.8**
1h	4h	**60**	**0.98**	**20.1**	0.3	**16.2**	0.3	**1.2**	0.3	**62.6**	0.4	63.8	**1.9**

[DCC] is the concentration of the dynamic covalent chemistry reaction at any time =[**1-1**]+[**2-2**]+[**Form A**]+[**Form B**]

[**1-2**] = **Form A** + **Form B** [**A**] ≡ [**Form A**] [**B**] ≡ [**Form B**]

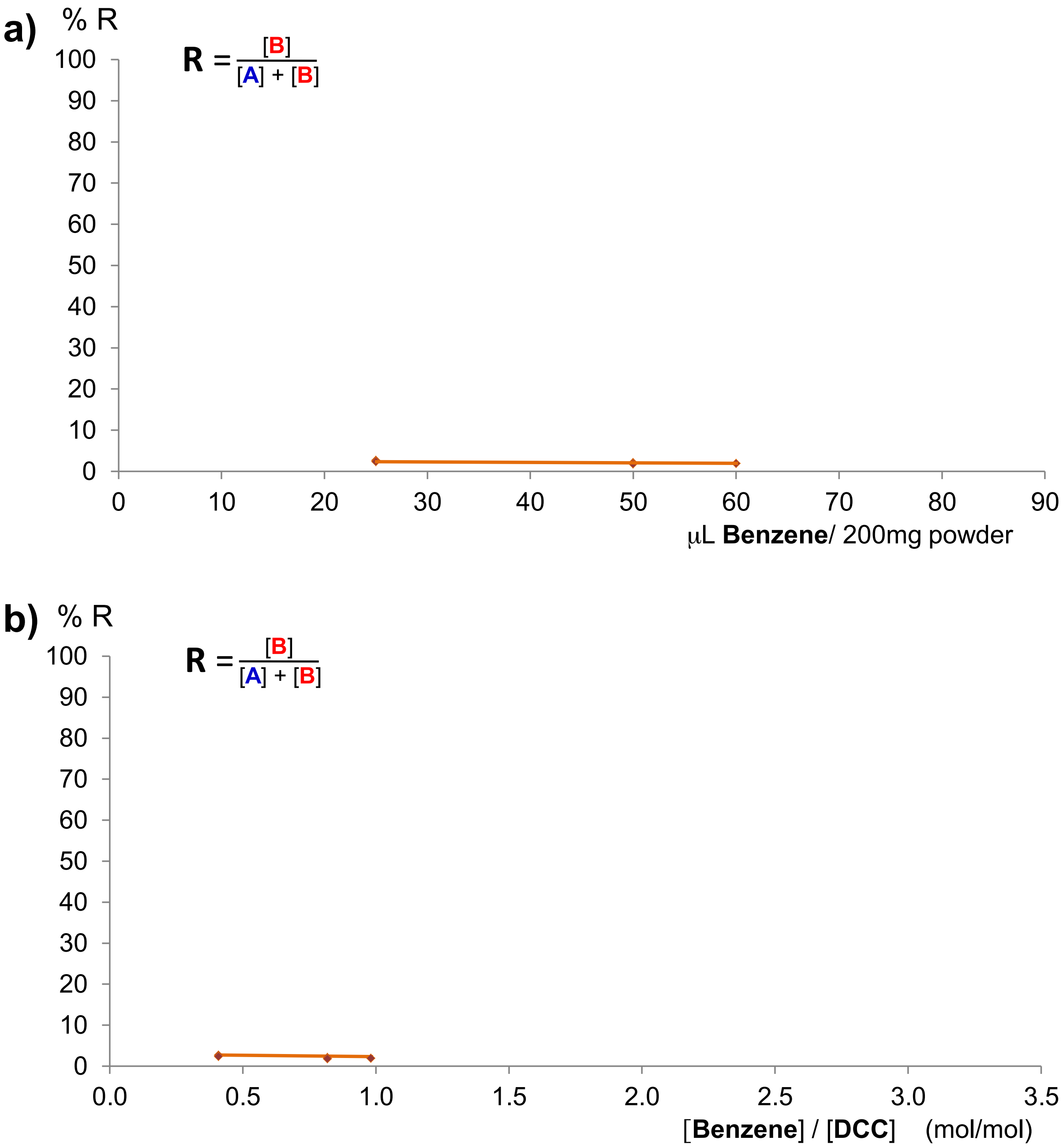

[**DCC**] is the concentration of the dynamic covalent chemistry reaction at any time = [**1-1**]+[**2-2**]+[**A**]+[**B**]
[**A**] is the concentration of **Form A** in DCC; [**B**] is the concentration of **Form B** in DCC

Figure S 62 Experimental milling equilibrium curves using “ball mill grinding procedure 4” plotted as the concentration of benzene versus %R index. R is the ratio of the concentration of **Form B** with respect to the total concentration of **Form B** plus **Form A** formed in the solid state DCC reaction for each specific experimental point. **a)** Curve expressed as μL benzene / 200 mg powder and **b)** curve expressed as mols of benzene added to the reaction with respect to 0.68 mmol of DCC library; this library is composed by the homodimers **1-1** and **2-2** and the heterodimers **Form A** and **Form B**. R is calculated from the Rietveld refinement of PXRD data giving the phase composition of the solid-state DCC experiment at equilibrium as %M. Each point in the graph is an individual experiment. No fitting was performed and the curves drawn are only a guide to the eye.

8.18 LAG experiments using toluene as grinding liquid on solid-state DCC reaction (Procedure 4)

These experiments were performed by adding the solvent by normal pipetting using Procedure 4 (Section 4.5.4).

8.18.1 Experimental details and HPLC data (Procedure 4)

Table S 65 Experimental details of ball mill LAG experiments with **toluene** as grinding solvent used to prepare the milling equilibrium curves and corresponding HPLC data. Tabulation of weights (mg) of starting materials (**1-1** & **2-2**), volume of **toluene** added to the solid state DCC reaction and the corresponding grinding time used to ensure that thermodynamic equilibrium is achieved for each single experimental point. The chemical composition of the reaction product as %M obtained by HPLC analysis is tabulated. Details of HPLC method are included.

Ball mill LAG grinding reaction taken to thermodynamic equilibrium using Procedure 4

2%M dbu + µL **Toluene** added to 0.68 mmol **DCC** ([**1-1**] + [**2-2**] + [**1-2**])

Reagents:		$(2NO_2PhS)_2$	$(4ClPhS)_2$	Toluene added		**HPLC conditions**		
MW:		308.33	287.23	to powder		Zorbax XDB C18, 1.8 µm; 4.6x50 mm		
%M initial		50%M	50%M	MW	Densiity	A: H_2O+0.1% FA; B: MeCN+0.1%FA		
mmol		0.34	0.34	(g/mol)	(g/mL)	0-2min 75-85%B;		
mg		104.8 mg	97.6 mg	92.14	0.8619	2ml/min; 60°C; 259 nm (8nm bandwidth)		
						**HPLC results**		
pre-soaking time	grinding time @ 30 Hz to equilibrium	$(2NO_2PhS)_2$ weighed (mg)	$(4ClPhS)_2$ weighed (mg)	Toluene µL	[Toluene]/[DCC] (mol/mol)	$(2NO_2PhS)_2$ (1-1) %M	Product (1-2) %M	$(4ClPhS)_2$ (2-2) %M
30 m	1h	104.81	97.67	15	0.21	1.0	**97.9**	1.1
30 m	1h	104.82	97.64	18	0.25	2.2	**95.9**	1.9
30 m	1h20m	104.80	97.67	20	0.28	1.8	**96.2**	1.9
30 m	1h	104.80	97.67	25	0.34	2.9	**94.4**	2.7
30 m	1h20m	104.81	97.66	30	0.41	2.7	**95.7**	1.6
0m	2h	104.83	97.66	50	0.69	13.4	**74.6**	12.0
1h	4h	104.82	97.66	60	0.83	15.7	**71.4**	12.8

[DCC] is the concentration of the dynamic covalent chemistry reaction at any time =[1-1]+[2-2]+[1-2]

[1-2] = **Form A** + **Form B**

8.18.2 **PXRD scans, tabulation of Rietveld refinement of PXRD data and corresponding experimental milling equilibrium curves (Procedure 4)**

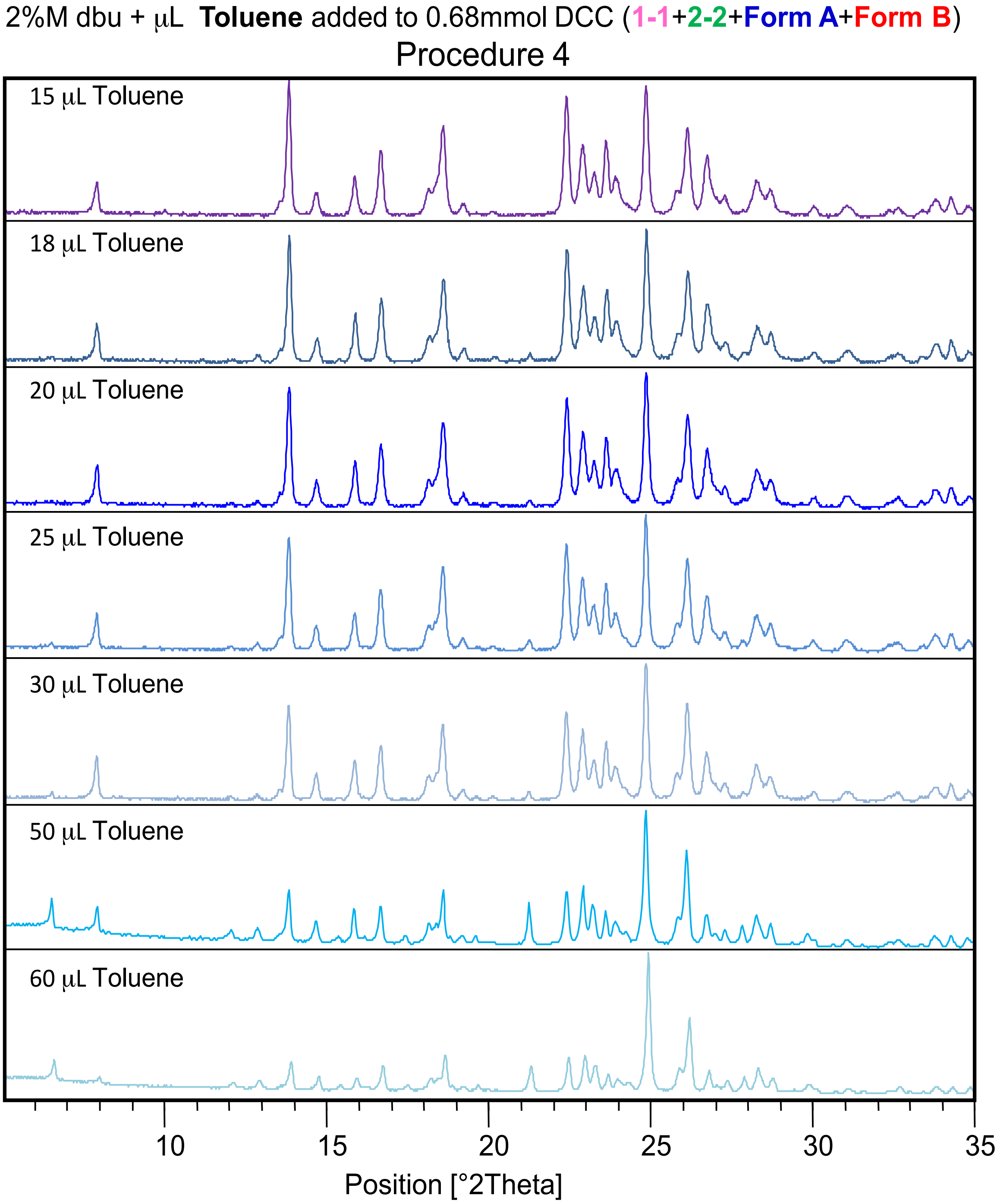

PXRD scan 21 Ball mill LAG reaction at equilibrium with different volumes of **toluene**. Each PXRD scan was obtained at equilibrium from a single point experiment with the specified added volume of **toluene**. The PXRD sample was prepared immediately after completion of the grinding experiment, and scanned between 5 and 45° (2θ).

Table S 66 Tabulation of the phase composition and corresponding R index used to prepare the milling equilibrium curve with **toluene** as the LAG solvent using ball mill grinding **Procedure 4**. The phase composition was calculated by Rietveld refinement of PXRD data as %M of the mixture of starting materials (**1-1** and **2-2**) and the polymorphs of the product (**Form A** and **Form B**). The corresponding refinement errors (esd) have been calculated as %mol. The R index is calculated as the ratio between the concentration of **Form B** versus the total concentration of **Form B** plus **Form A**.

Ball mill LAG grinding reaction taken to thermodynamic equilibrium using Procedure 4													
2%M dbu+µL Toluene added to 0.68 mmol DCC ([1-1]+[2-2]+[Form A]+[Form B])													
pre-soaking time	grinding time @ 30Hz to equilib.	Toluene added to powder		1-1		2-2		Form B		Form A		[A]+[B]	R = [B] / ([A]+[B])
		Toluene µL	[Toluene]/[DCC] (mol/mol)	%M	e.s.d. mol%	%M	e.s.d. mol%	%M	e.s.d. mol%	%M	e.s.d. mol%	total polymorph	% R
30 m	1h	**15**	**0.21**	**1.0**	0.3	**0.8**	0.3	**2.3**	0.4	**95.9**	0.6	98.2	**2.3**
30 m	1h	**18**	**0.25**	**2.9**	0.4	**2.3**	0.3	**3.3**	0.5	**91.5**	0.7	94.8	**3.5**
30 m	1h20m	**20**	**0.28**	**2.3**	0.4	**1.9**	0.3	**2.9**	0.5	**92.9**	0.7	95.8	**3.0**
30 m	1h	**25**	**0.34**	**3.4**	0.3	**2.8**	0.2	**2.5**	0.4	**91.3**	0.5	93.8	**2.7**
30 m	1h20m	**30**	**0.41**	**3.3**	0.4	**2.7**	0.3	**3.7**	0.5	**90.3**	0.7	94.0	**3.9**
0m	2h	**50**	**0.69**	**15.6**	0.5	**12.5**	0.4	**2.7**	0.6	**69.3**	0.7	72.0	**3.8**
1h	4h	**60**	**0.83**	**18.0**	0.3	**14.5**	0.3	**2.2**	0.4	**65.3**	0.4	67.5	**3.3**

[DCC] is the concentration of the dynamic covalent chemistry reaction at any time =[**1-1**]+[**2-2**]+[**Form A**]+[**Form B**]

[**1-2**] = **Form A** + **Form B** [**A**] ≡ [**Form A**] [**B**] ≡ [**Form B**]

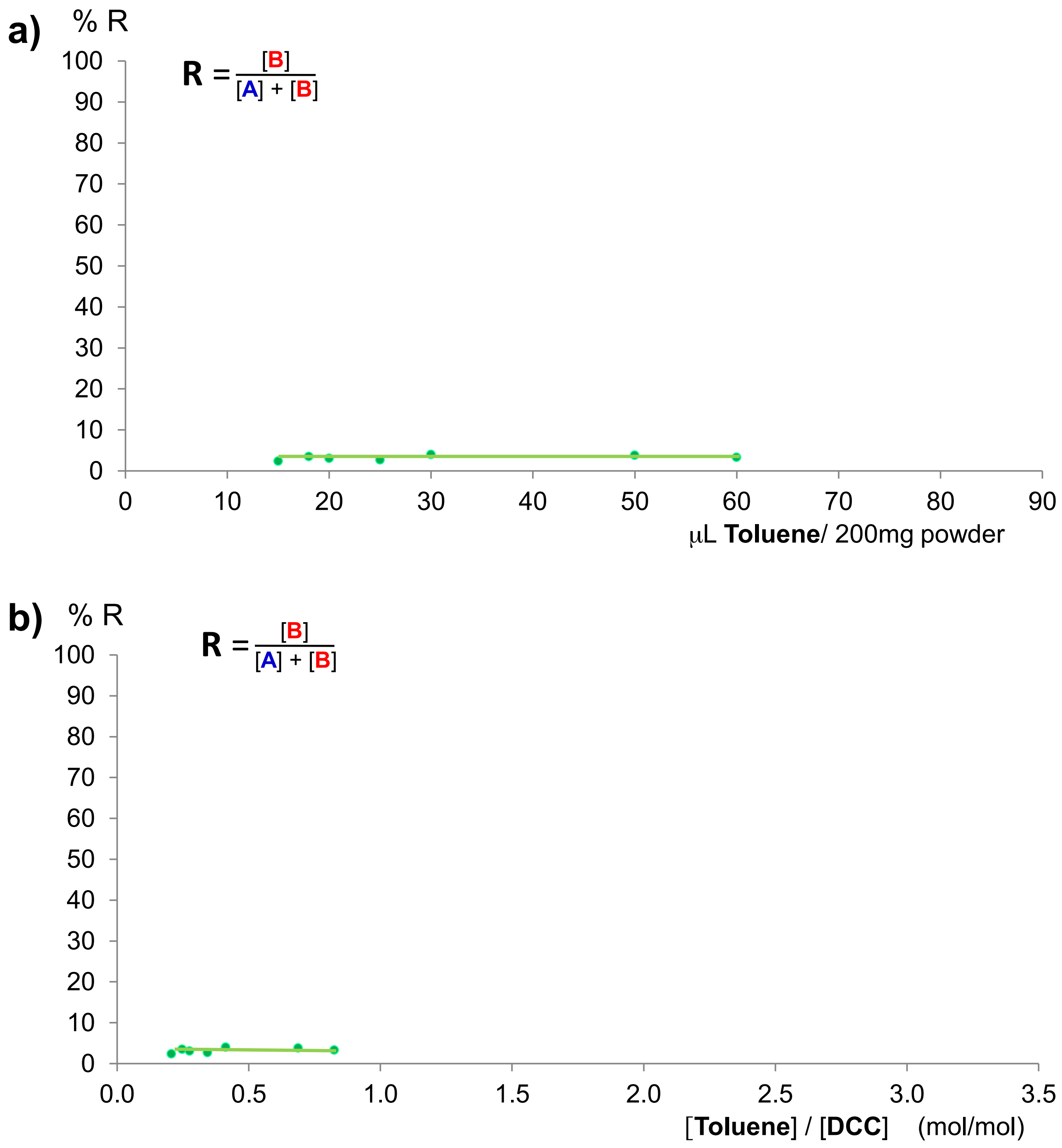

[**DCC**] is the concentration of the dynamic covalent chemistry reaction at any time = [**1-1**]+[**2-2**]+[**A**]+[**B**]
[**A**] is the concentration of **Form A** in DCC; [**B**] is the concentration of **Form B** in DCC

Figure S 63: Experimental milling equilibrium curves using "ball mill grinding procedure 4" plotted as the concentration of toluene versus %R index. R is the ratio of the concentration of **Form B** with respect to the total concentration of **Form B** plus **Form A** formed in the solid state DCC reaction for each specific experimental point. **a)** Curve expressed as μL toluene / 200 mg powder and **b)** curve expressed as mols of toluene added to the reaction with respect to 0.68 mmol of DCC library; this library is composed by the homodimers **1-1** and **2-2** and the heterodimers **Form A** and **Form B**. R is calculated from the Rietveld refinement of PXRD data giving the phase composition of the solid-state DCC experiment at equilibrium as %M. Each point in the graph is an individual experiment. No fitting was performed and the curves drawn are only a guide to the eye.

8.19 LAG experiments using perfluorodecalin as grinding liquid on solid-state DCC reaction (Procedure 4)

These experiments were performed by adding the solvent by normal pipetting using Procedure 4 (Section 4.5.4).

8.19.1 Experimental details and HPLC data (Procedure 4)

Table S 67 Experimental details of ball mill LAG experiments with **perfluorodecalin** as grinding solvent used to prepare the milling equilibrium curves and corresponding HPLC data. Tabulation of weights (mg) of starting materials (**1-1** & **2-2**), volume of **perfluorodecalin** added to the solid state DCC reaction and the corresponding grinding time used to ensure that thermodynamic equilibrium is achieved for each single experimental point. The chemical composition of the reaction product as %M obtained by HPLC analysis is tabulated. Details of HPLC method are included.

Ball mill LAG grinding reaction taken to thermodynamic equilibrium using Procedure 4								
2%M dbu + µL **F18-decalin** added to 0.68 mmol **DCC** ([**1-1**] + [**2-2**] + [**1-2**])								
Reagents:		**$(2NO_2PhS)_2$**	**$(4ClPhS)_2$**	F18-decalin added		**HPLC conditions**		
MW:		308.33	287.23	to powder		Zorbax XDB C18, 1.8 µm; 4.6x50 mm		
%M initial		50%M	50%M	MW	Densiity	A: H_2O+0.1% FA; B: MeCN+0.1%FA		
mmol		0.34	0.34	(g/mol)	(g/mL)	0-2min 75-85%B;		
mg		104.8 mg	97.6 mg	462.10	1.9465	2ml/min; 60°C; 259 nm (8nm bandwidth)		
						**HPLC results**		
pre-soaking time	grinding time @ 30 Hz to equilibrium	$(2NO_2PhS)_2$ weighed (mg)	$(4ClPhS)_2$ weighed (mg)	F18-decalin µL	[F18-decalin] /[DCC] (mol/mol)	$(2NO_2PhS)_2$ **(1-1)** %M	Product **(1-2)** %M	$(4ClPhS)_2$ **(2-2)** %M
45m	2h	104.83	97.64	**25**	**0.15**	21.3	**57.8**	20.9
45m	2h	104.83	97.66	**50**	**0.31**	1.4	**96.9**	1.7
45m	3h	104.79	97.65	**75**	**0.46**	0.7	**98.5**	0.8
[DCC] is the concentration of the dynamic covalent chemistry reaction at any time =[1-1]+[2-2]+[1-2]								
[1-2] = **Form A** + **Form B**								

8.19.2 PXRD scans, tabulation of Rietveld refinement of PXRD data and corresponding experimental milling equilibrium curves (Procedure 4)

2%M dbu + μL **F18-decalin** added to 0.68mmol DCC (**1-1**+**2-2**+**Form A**+**Form B**)

Procedure 4

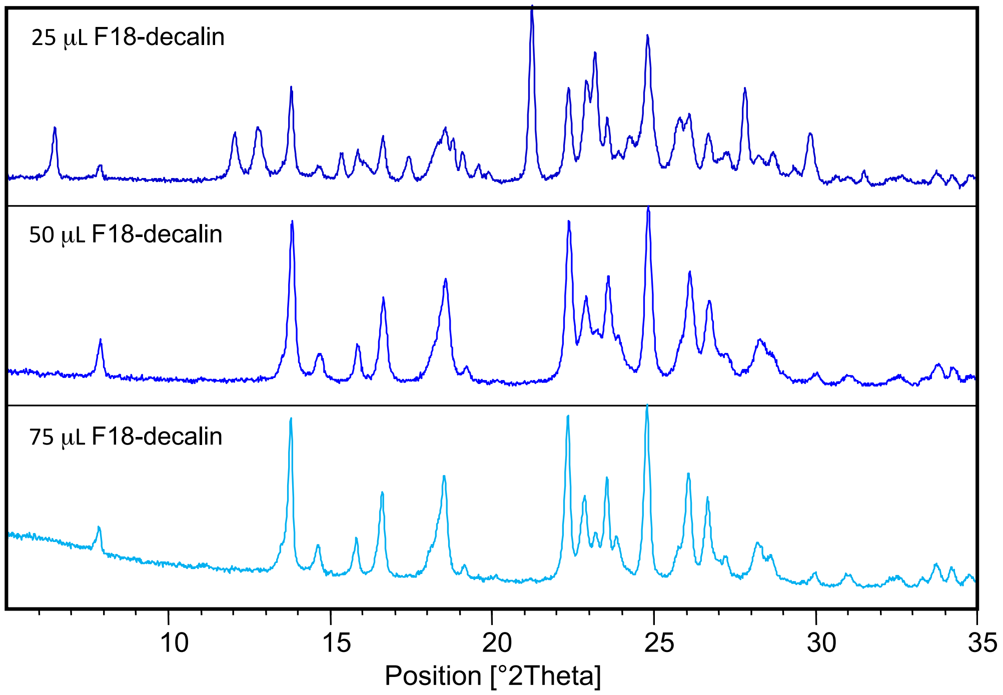

PXRD scan 22 Ball mill LAG reaction at equilibrium with different volumes of **perfluorodecalin**. Each PXRD scan was obtained at equilibrium from a single point experiment with the specified added volume of **perfluorodecalin**. The PXRD sample was prepared immediately after completion of the grinding experiment, and scanned between 5 and 45° (2θ).

Table S 68 Tabulation of the phase composition and corresponding R index used to prepare the milling equilibrium curve with **perfluorodecalin** as the LAG solvent using ball mill grinding **Procedure 4**. The phase composition was calculated by Rietveld refinement of PXRD data as %M of the mixture of starting materials (**1-1** and **2-2**) and the polymorphs of the product (**Form A** and **Form B**). The corresponding refinement errors (esd) have been calculated as %mol. The R index is calculated as the ratio between the concentration of **Form B** versus the total concentration of **Form B** plus **Form A**.

Ball mill LAG grinding reaction taken to thermodynamic equilibrium using Procedure 4

2%M dbu+μL F18-decalin added to 0.68 mmol DCC ([**1-1**]+[**2-2**]+[**Form A**]+[**Form B**])

pre-soaking time	grinding time @ 30Hz to equilib.	F18-decalin added to powder		**1-1**		**2-2**		**Form B**		**Form A**		[A]+[B]	R = [B] / ([A]+[B])
		F18-decalin μL	[F18-decalin] /[DCC] (mol/mol)	%M	e.s.d. mol%	%M	e.s.d. mol%	%M	e.s.d. mol%	%M	e.s.d. mol%	total polymorph	% R
45m	2h	**25**	**0.15**	**30.9**	0.5	**24.8**	0.5	**3.1**	0.6	**41.2**	0.6	44.3	**7.0**
45m	2h	**50**	**0.31**	**1.9**	0.5	**1.5**	0.4	**7.0**	0.7	**89.6**	0.9	96.6	**7.2**
45m	3h	**75**	**0.46**	**1.5**	0.4	**1.2**	0.3	**5.2**	0.7	**92.2**	0.8	97.4	**5.3**

[DCC] is the concentration of the dynamic covalent chemistry reaction at any time =[**1-1**]+[**2-2**]+[**Form A**]+[**Form B**]

[**1-2**] = **Form A** + **Form B** [A] ≡ [**Form A**] [B] ≡ [**Form B**]

a)

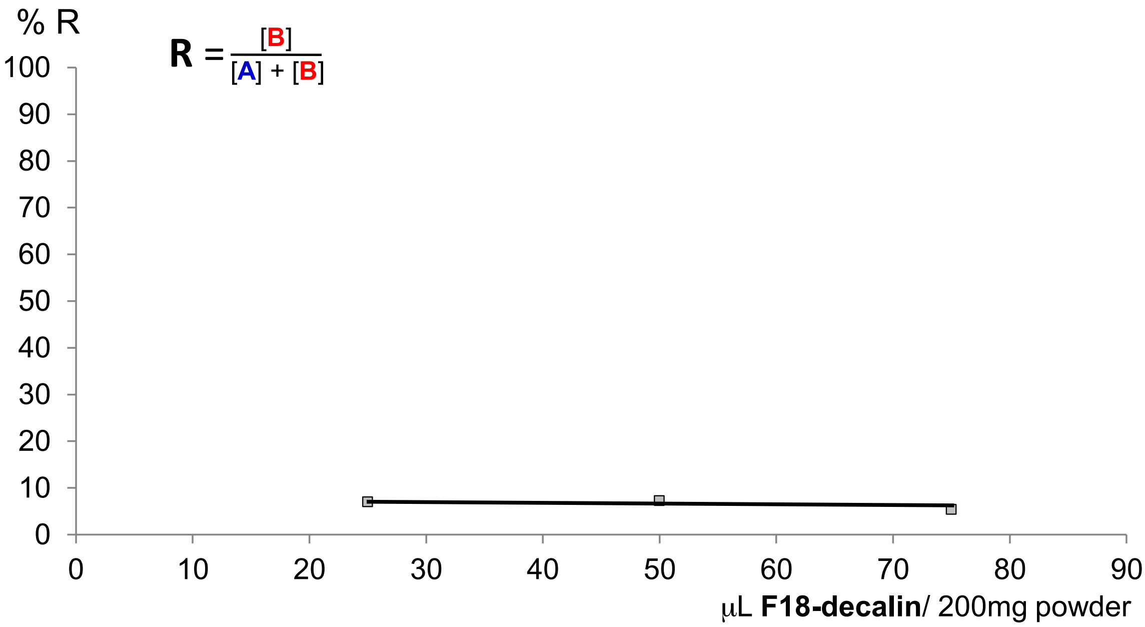

b)

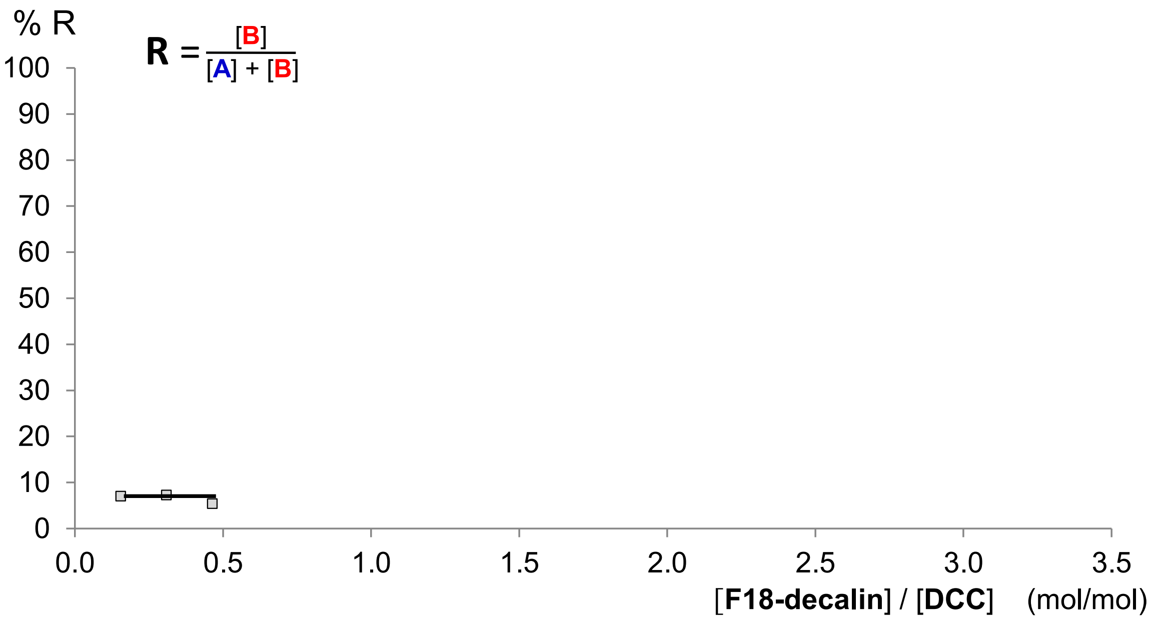

[**DCC**] is the concentration of the dynamic covalent chemistry reaction at any time = [**1-1**]+[**2-2**]+[**A**]+[**B**]
[**A**] is the concentration of **Form A** in DCC; [**B**] is the concentration of **Form B** in DCC

Figure S 64: Experimental milling equilibrium curves using "ball mill grinding procedure 4" plotted as the concentration of perfluorodecalin versus %R index. R is the ratio of the concentration of **Form B** with respect to the total concentration of **Form B** plus **Form A** formed in the solid state DCC reaction for each specific experimental point. **a)** Curve expressed as µL perfluorodecalin / 200 mg powder and **b)** curve expressed as mols of perfluorodecalin added to the reaction with respect to 0.68 mmol of DCC library; this library is composed by the homodimers **1-1** and **2-2** and the heterodimers **Form A** and **Form B**. R is calculated from the Rietveld refinement of PXRD data giving the phase composition of the solid-state DCC experiment at equilibrium as %M. Each point in the graph is an individual experiment. No fitting was performed and the curves drawn are only a guide to the eye.

8.20 Summary of results of polymorph compositions (%R) at thermodynamic equilibrium versus type and volume of solvent used in ball mill LAG

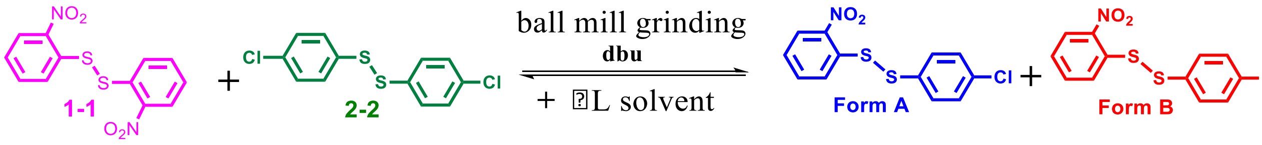

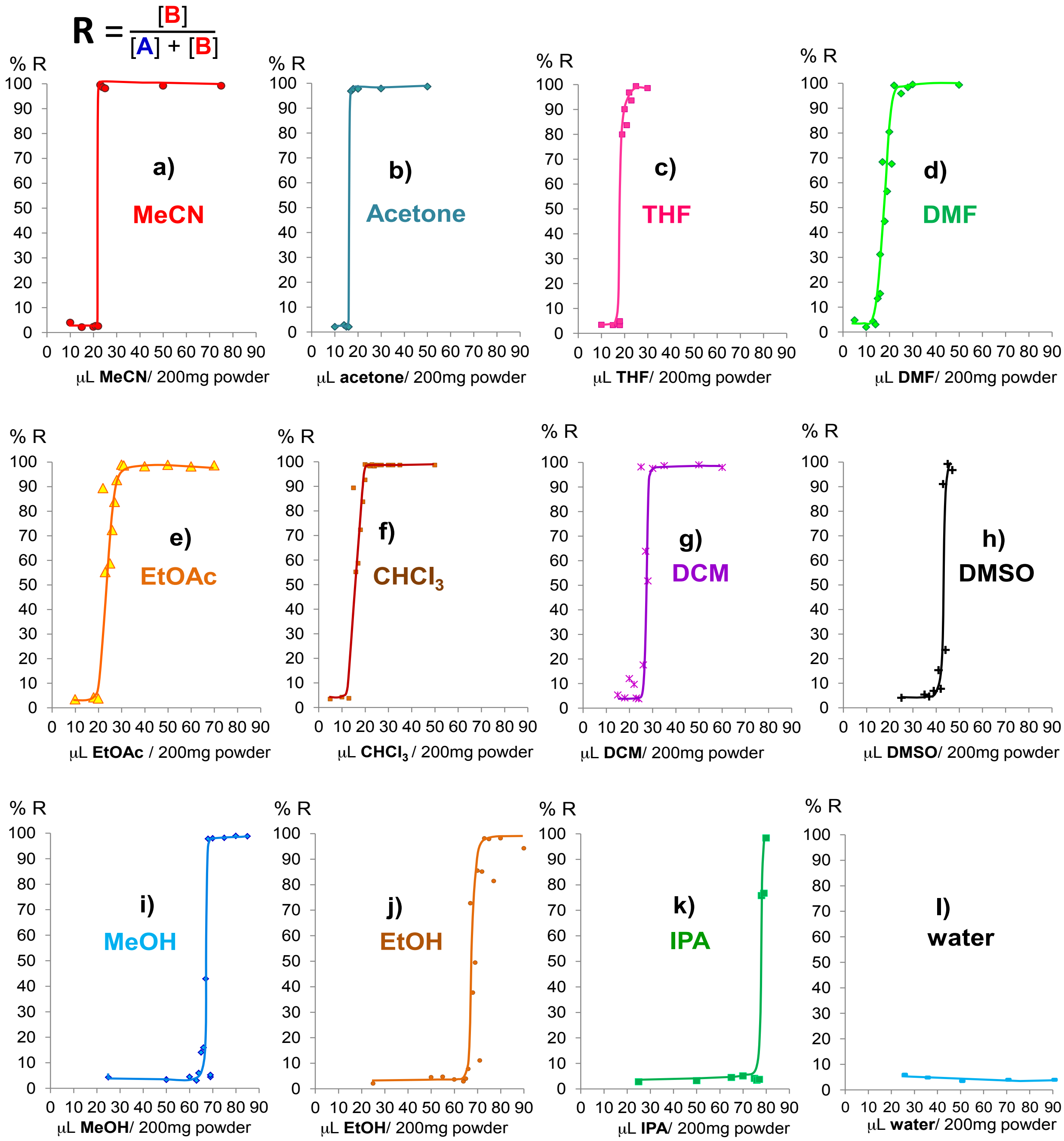

Figure S 65 Individual milling equilibrium curves prepared from the Rietveld refinement of PXRD data showing %R with respect to µL of each solvent added to 200 mg of powder. **R** is the ratio of **Form B** polymorph to total 1-2 (**Form A** +**Form B)** formed in solid state DCC reaction. The ball mill LAG reaction is started from 200 mg of equimolar amounts of homodimers **1-1** & **2-2** and 2%M dbu (base catalyst). 0% R is obtained when the solid state DCC reaction at equilibrium results in the quantitative formation of **Form A**, while 100%R is obtained when quantitative **Form B** is formed at equilibrium.

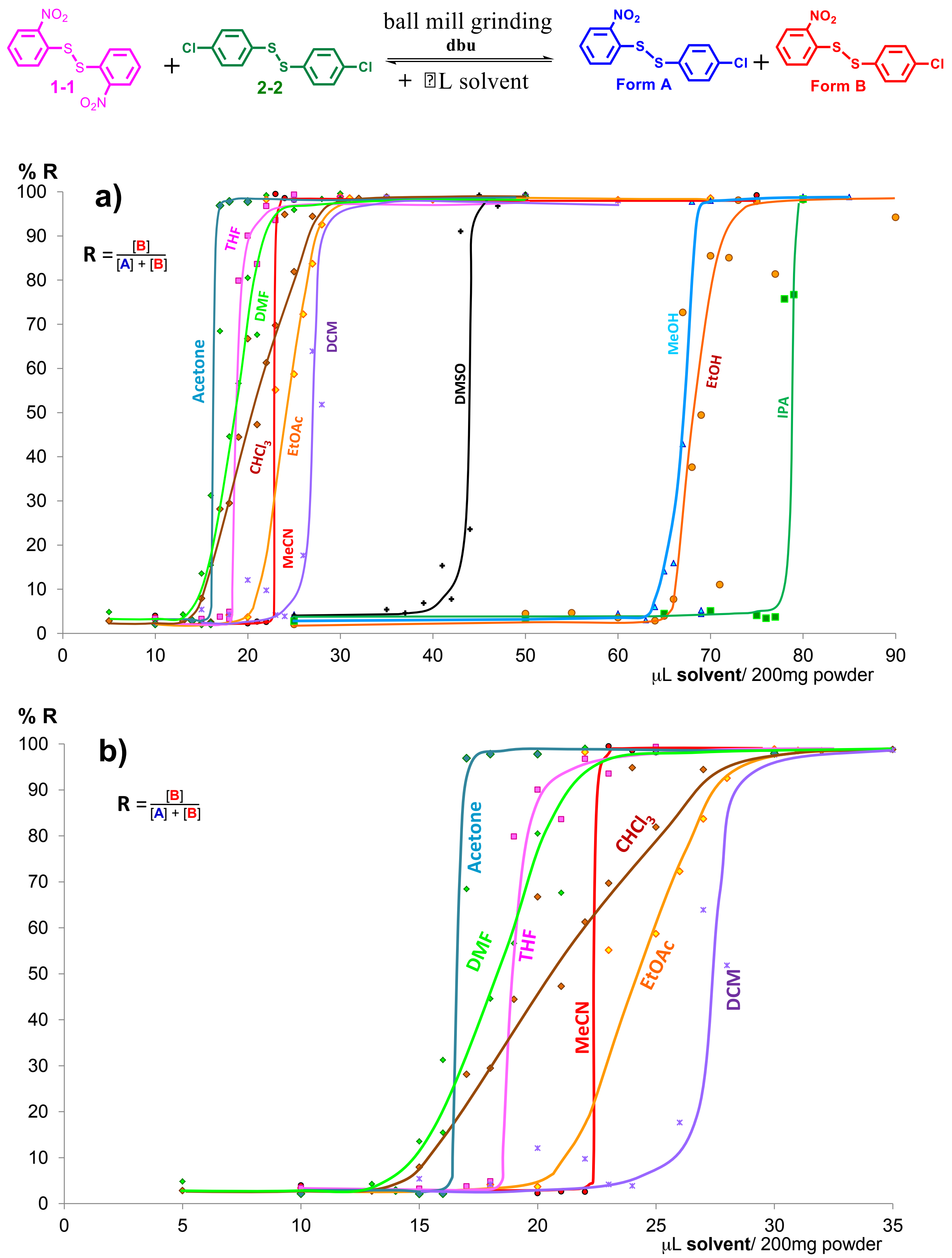

Figure S 66 Comparative equilibrium curves prepared from PXRD data showing %R with respect to µL of each solvent added to 200 mg of powder. **R** is the ratio of **Form B** polymorph to total 1-2 (**Form A** +**Form B)** formed in solid state DCC reaction. The ball mill LAG reaction is started from 200 mg of equimolar amounts of homodimers **1-1** & **2-2** and 2%M dbu (base catalyst). 0% R is obtained when the solid state DCC reaction at equilibrium results in the quantitative formation of **Form A**, while 100%R is obtained when quantitative **Form B** is formed at equilibrium. a) milling equilibrium curves for all 11 solvents forming **Form B** with high volume of solvent; b) expanded milling equilibrium curves bunched between 13 & 28µL solvent (Acetone, THF, EtOAc, $CHCl_3$, DMF, DCM & MeCN).

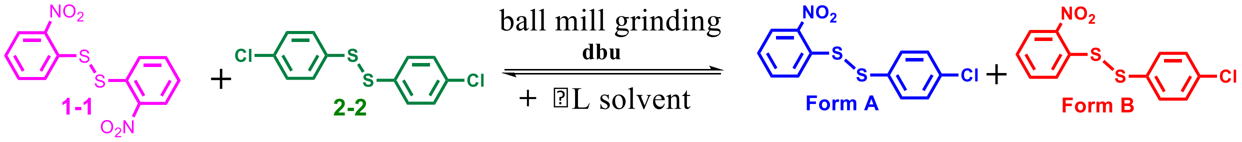

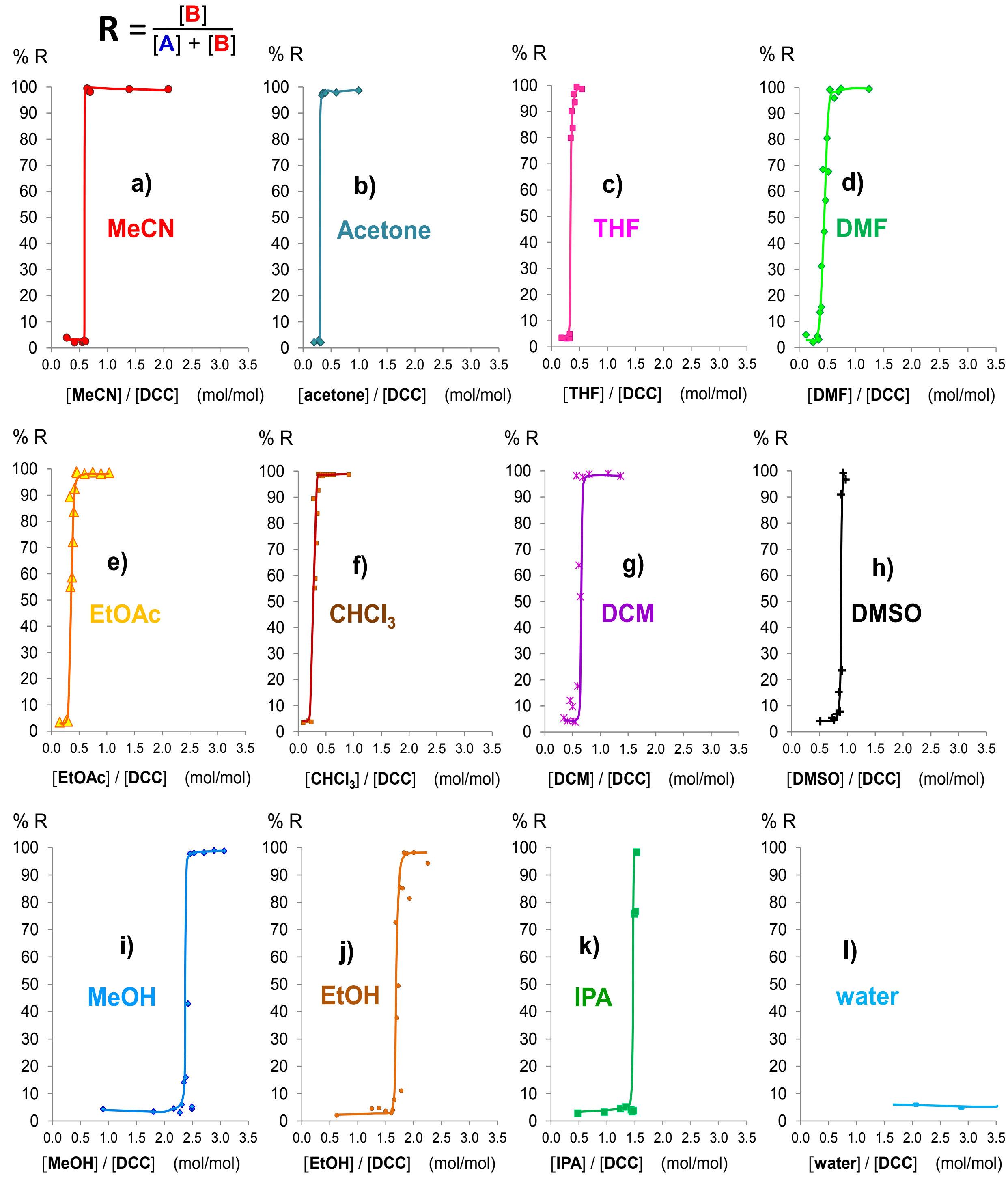

Figure S 67 Individual milling equilibrium curves prepared from PXRD data showing **%R** with respect to mol of each solvent added per mol of powder. **R** is the ratio of **Form B** polymorph to total 1-2 (**Form A** +**Form B)** formed in solid state DCC reaction. The ball mill LAG reaction is started from 200 mg of equimolar amounts of homodimers **1-1** & **2-2** and 2%M dbu (base catalyst). 0% R is obtained when the solid state DCC reaction at equilibrium results in the quantitative formation of **Form A**, while 100%R is obtained when quantitative **Form B** is formed at equilibrium.

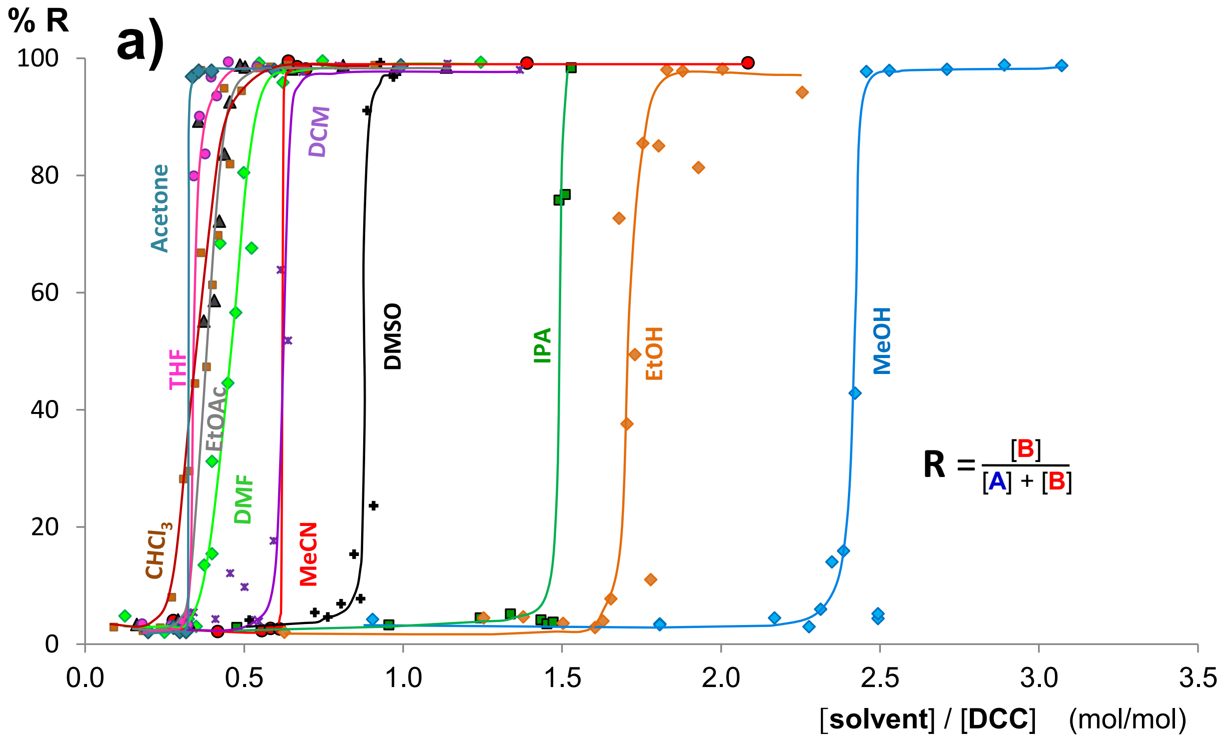

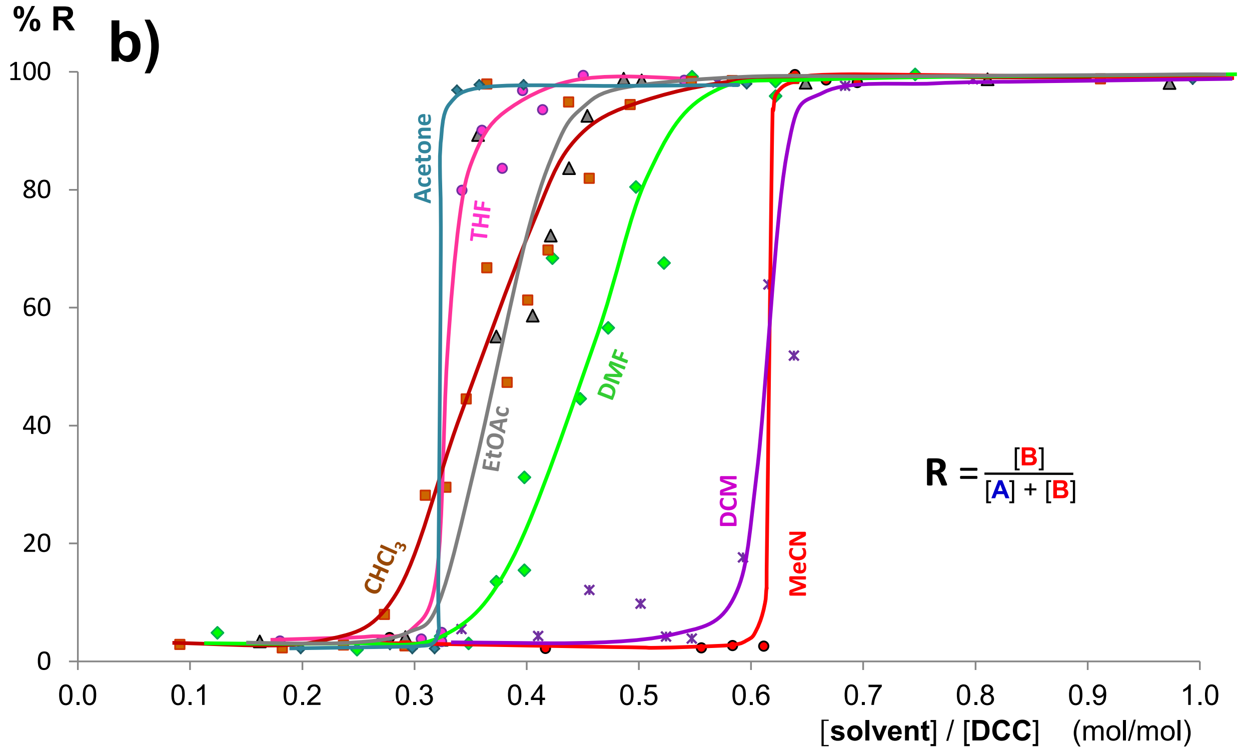

Figure S 68 Comparative equilibrium curves prepared from PXRD data showing %R with respect to mol of each solvent added per mol of powder. **R** is the ratio of **Form B** polymorph to total 1-2 (**Form A** +**Form B)** formed in solid state DCC reaction. The ball mill LAG reaction is started from 200mg of equimolar amounts of homodimers **1-1** & **2-2** and 2%M dbu (base catalyst). 0% R is obtained when the solid state DCC reaction at equilibrium results in the quantitative formation of **Form A**, while 100%R is obtained when quantitative **Form B** is formed at equilibrium.
a) experimental milling equilibrium curves for all 11 solvents forming **Form B** with high volume of solvent;
b) expanded milling equilibrium curves bunched between 0.3 and 0.5 mol/mol (Acetone, THF, EtOAc, $CHCl_3$, DMF, DCM & MeCN)

9 Preliminary investigation of phase equilibria and chemical equilibria on ball mill LAG of system ii

For each solvent, equilibration times were assessed by means of kinetic milling experiments. The materials obtained by these milling experiments were all characterized by HPLC, to confirm that the product had formed quantitatively and to check that the compound had not degraded with extensive grinding. These results allowed us to establish for each solvent milling time ranges in which the heterodimer (compound **ii**) is obtained quantitatively and does not degrade. We are aware of the fact that chemical equilibrium (i.e. equilibrium among chemical species) does not necessarily imply phase equilibrium (equilibrium among polymorphs). In the case of neat grinding and acetonitrile we know that phase equilibrium and chemical equilibrium are achieved at the same time within minutes.[20] For this paper this was also tested for four extra solvents: acetone (25 μL, section 9.1), ethyl acetate (30 μL, section 9.2), chloroform (25 μL, section 9.3) and dichloromethane (60 μL, section 9.4) . In all these cases we proved that phase and chemical equilibrium were reached within experimental error.

9.1 Chemical and phase equilibria of ball mill LAG with 25 μL Acetone of system ii at different grinding times

Acetone was used as a LAG solvent in ball mill LAG grinding of system ii as shown in Section 8.3. 25μL Acetone leads to the formation by ball mill LAG to a mixture of **Form A** and **Form B**. We found that 45 minutes grinding was sufficient to reach chemical (See Table S 26) and phase equilibrium (See Table S27).

Table S 69: Experimental details and HPLC results of the chemical composition of LAG reaction with 25 μL Acetone at different grinding times

Study of chemical equilibrium in ball mill LAG using Procedure 1							
2%M dbu + **25 μL acetone** added to 0.68 mmol **DCC** ([**1-1**] + [**2-2**] + [**1-2**])							
Reagents:	$(2NO_2PhS)_2$	$(4ClPhS)_2$	acetone added		**HPLC conditions**		
MW:	308.33	287.23	to powder		Zorbax XDB C18, 1.8 μm; 4.6x50 mm		
%M initial	50%M	50%M	MW	Densiity	A: H_2O+0.1% FA; B: MeCN+0.1%FA		
mmol	0.34	0.34	(g/mol)	(g/mL)	0-2min 75-85%B;		
mg	104.8 mg	97.6 mg	58.08	0.7849	2ml/min; 60°C; 259 nm (8nm bandwidth)		
					**HPLC results**		
grinding time @ 30 Hz to equilibrium	$(2NO_2PhS)_2$ weighed (mg)	$(4ClPhS)_2$ weighed (mg)	acetone μL	[acetone]/[DCC] (mol/mol)	$(2NO_2PhS)_2$ (1-1) %M	Product **(1-2)** %M	$(4ClPhS)_2$ (2-2) %M
**30**	104.81	97.65	25	0.50	4.3	**92.1**	3.6
**45**	104.86	97.67	25	0.50	3.8	**93.0**	3.2
**60**	104.81	97.68	25	0.50	5.6	**89.9**	4.4
**120**	104.83	97.66	25	0.50	7.1	**87.1**	5.9
[DCC] is the concentration of the dynamic covalent chemistry reaction at any time =[1-1]+[2-2]+[1-2]							
[1-2] = **Form A** + **Form B**							

Table S 70 Tabulation of Rietveld refinement of PXRD data of the phase composition of LAG reaction with 25 μL Acetone at different grinding times

Study of phase equilibrium in ball mill LAG using Procedure 1												
2%M dbu+25μL acetone added to 0.68 mmol DCC ([**1-1**]+[**2-2**]+[**Form A**]+[**Form B**])												
grinding time @ 30Hz to equilib.	Acetone added to powder		**1-1**		**2-2**		**Form B**		**Form A**		[A]+[B]	**R =** [B] / ([A]+[B])
	Acetone μL	[Acetone]/[DCC] (mol/mol)	%M	e.s.d. mol%	%M	e.s.d. mol%	%M	e.s.d. mol%	%M	e.s.d. mol%	total polymorph	**% R**
**30 min**	25	**0.50**	4.7	0.3	3.8	0.2	**90.7**	0.4	**0.9**	0.2	91.6	**99 %**
**45 min**	25	**0.50**	4.4	0.3	3.5	0.2	**91**	0.4	**1**	0.3	92.0	**99 %**
**60 min**	25	**0.50**	5.3	0.2	4.3	0.2	**89.8**	0.3	**0.6**	0.1	90.4	**99 %**
**120 min**	25	**0.50**	7.1	0.2	5.7	0.2	**86.8**	0.3	**0.4**	0.1	87.2	**100 %**
[DCC] is the concentration of the dynamic covalent chemistry reaction at any time =[**1-1**]+[**2-2**]+[**Form A**]+[**Form B**]												
[**1-2**] = **Form A** + **Form B** [A] ≡ [**Form A**] [B] ≡ [**Form B**]												

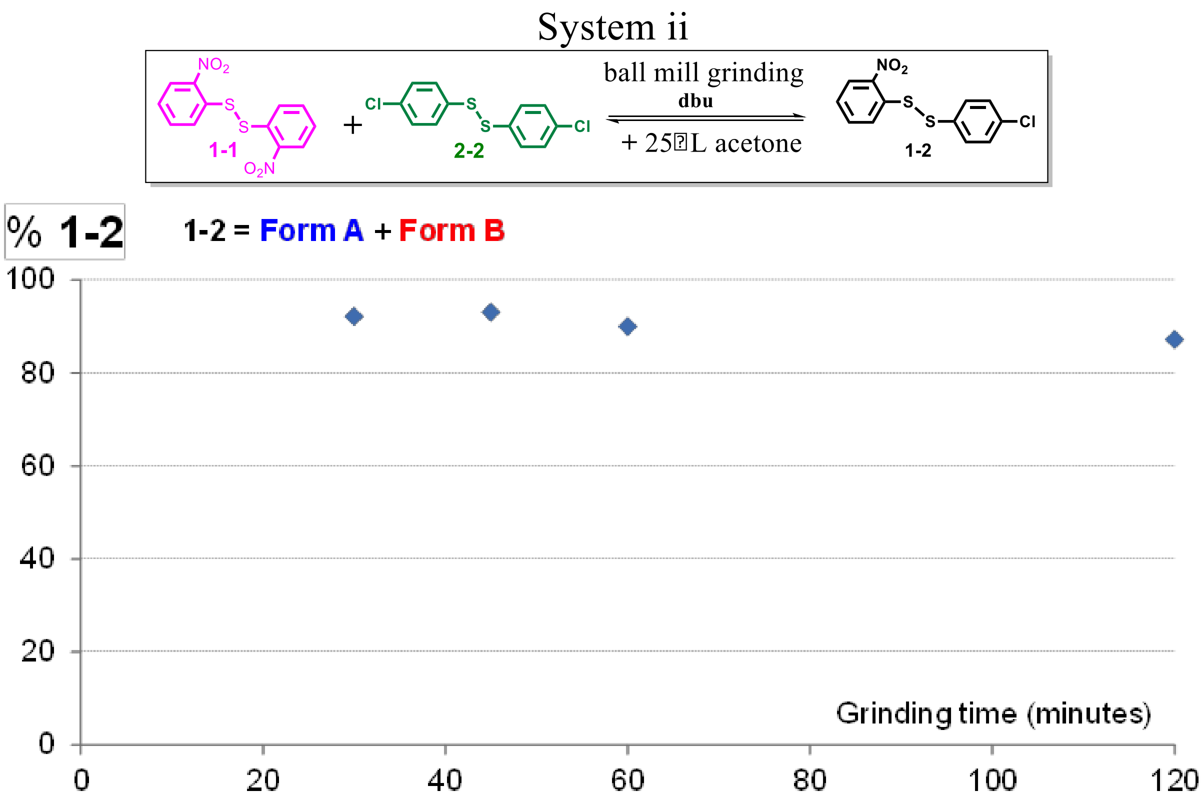

Figure S 69   Chemical equilibria of LAG reaction of system ii with 25 µL Acetone at different grinding times

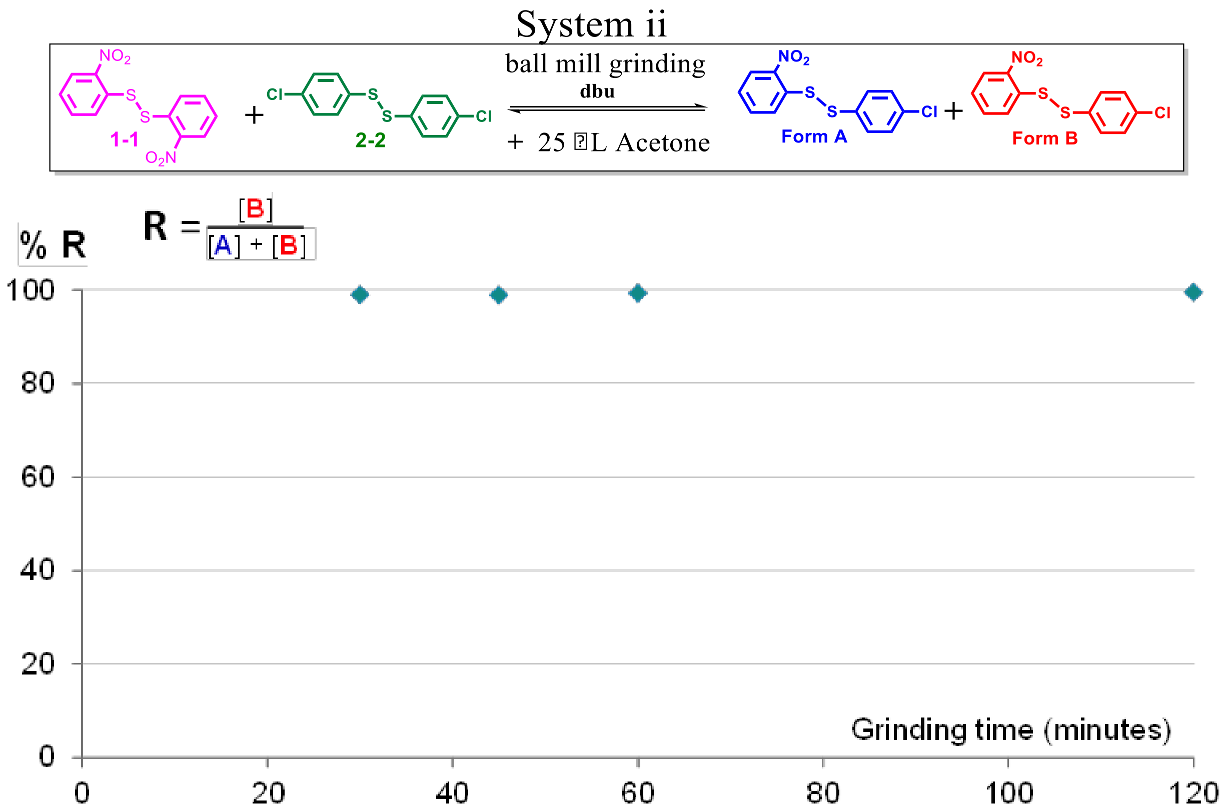

Figure S 70   Phase equilibria of LAG reaction of system ii with 25 µL Acetone at different grinding times

9.2 Chemical and phase equilibria of ball mill LAG with 30μL EtOAc of system ii at different grinding times

Ethyl acetate was used as a LAG solvent in ball mill LAG grinding of system ii as shown in Section 8.6. 30μL EtOAc leads to the formation by ball mill LAG of **Form B** predominantly. We found that 2 or more hours of grinding were sufficient to reach chemical (See Table S 35) and phase equilibrium (See Table S36).

Table S 71: Experimental details and HPLC results of the chemical composition of LAG reaction with 30μL EtOAc at different grinding times

Study of chemical equilibrium in ball mill LAG using Procedure 1							
2%M dbu + **30 μL EtOAc** added to 0.68 mmol **DCC** ([1-1] + [2-2] + [**1-2**])							
Reagents:	**$(2NO_2PhS)_2$**	**$(4ClPhS)_2$**	EtOAc added to powder		**HPLC conditions**		
MW:	308.33	287.23			Zorbax XDB C18, 1.8 μm; 4.6x50 mm		
%M initial	50%M	50%M	MW	Density	A: H_2O+0.1% FA; B: MeCN+0.1%FA		
mmol	0.34	0.34	(g/mol)	(g/mL)	0-2min 75-85%B;		
mg	104.8 mg	97.6 mg	88.11	0.8945	2ml/min; 60°C; 259 nm (8nm bandwidth)		
					**HPLC results**		
grinding time @ 30 Hz to equilibrium	$(2NO_2PhS)_2$ weighed (mg)	$(4ClPhS)_2$ weighed (mg)	EtOAc μL	[EtOAc]/[DCC] (mol/mol)	$(2NO_2PhS)_2$ (1-1) %M	Product (**1-2**) %M	$(4ClPhS)_2$ (2-2) %M
**1.0 h**	104.85	97.66	**30**	0.45	4.0	**92.4**	3.6
**1.5 h**	104.86	97.64	**30**	0.45	3.9	**92.4**	3.8
**2.0 h**	104.84	97.67	**30**	0.45	3.3	**93.8**	2.9
**3.0 h**	104.87	97.65	**30**	0.45	4.8	**91.1**	4.1
**4.0 h**	104.87	97.68	**30**	0.45	3.7	**93.4**	2.9
[DCC] is the concentration of the dynamic covalent chemistry reaction at any time =[1-1]+[2-2]+[1-2]							
[1-2] = **Form A** + **Form B**							

Table S 72 Tabulation of Rietveld refinement of PXRD data of the phase composition of LAG reaction with 30μL EtOAc at different grinding times

2%M dbu+**30μL EtOAc** added to 0.68 mmol DCC ([1-1]+[2-2]+[**Form A**]+[**Form B**])													
grinding time @ 30Hz to equilib.	EtOAc added to powder		**1-1**		**2-2**		**Form B**		**Form A**		[A]+[B]	**R = [B] / ([A]+[B])**
	EtOAc μL	[EtOAc]/[DCC] (mol/mol)	%M	e.s.d. mol%	%M	e.s.d. mol%	%M	e.s.d. mol%	%M	e.s.d. mol%	total polymorph	**% R**
**1.0 h**	30	**0.45**	4.3	0.3	3.5	0.2	**90.9**	0.4	**1.3**	0.3	92.2	**99 %**
**1.5 h**	30	**0.45**	3.5	0.3	2.8	0.3	**81.6**	0.6	**12.2**	0.5	93.8	**87 %**
**2.0 h**	30	**0.45**	2.7	0.4	2.2	0.3	**84.4**	0.7	**10.7**	0.5	95.1	**89 %**
**3.0 h**	30	**0.45**	4.8	0.2	3.9	0.2	**75.5**	0.4	**15.8**	0.3	91.3	**83 %**
**4.0 h**	30	**0.45**	3.6	0.2	2.9	0.2	**70.2**	0.3	**23.4**	0.3	93.6	**75 %**
[DCC] is the concentration of the dynamic covalent chemistry reaction at any time =[1-1]+[2-2]+[**Form A**]+[**Form B**]												
[**1-2**] = **Form A** + **Form B** [A] ≡ [**Form A**] [B] ≡ [**Form B**]												

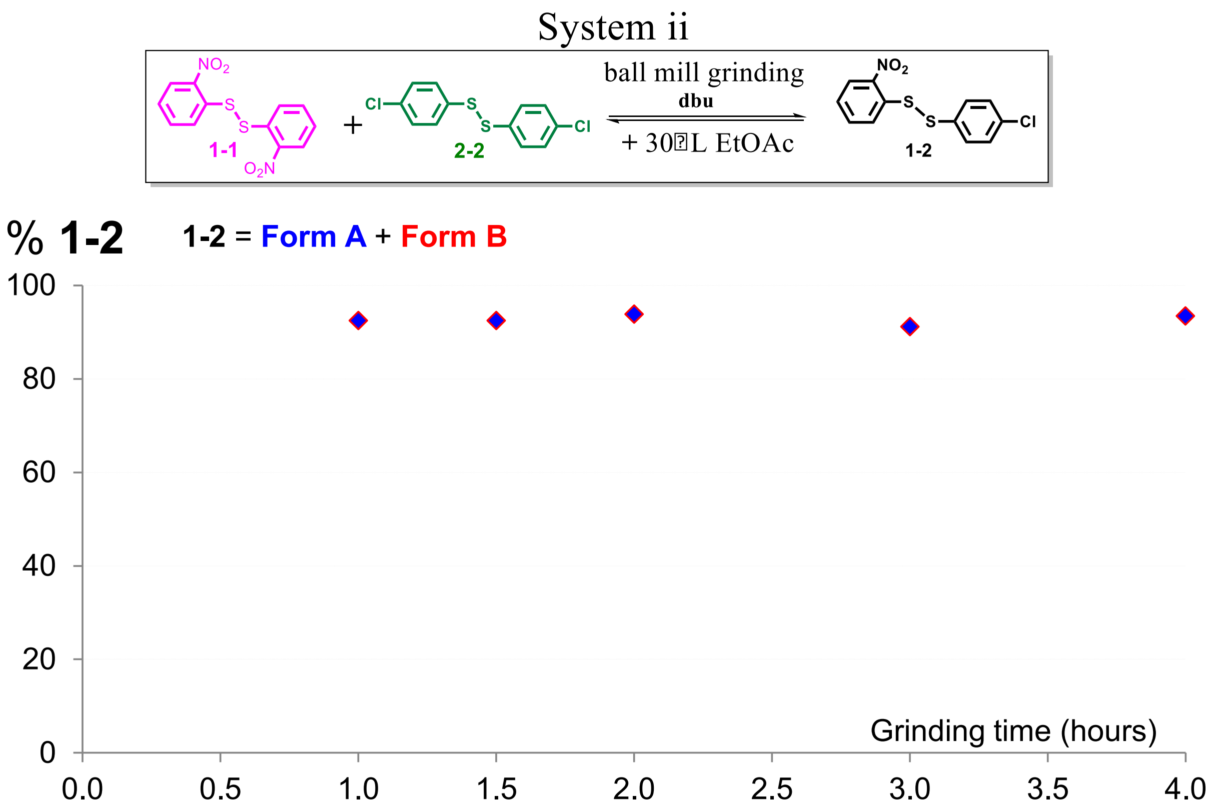

Figure S 71 Chemical equilibria of LAG reaction of system ii with 30 μL EtOAc at different grinding times

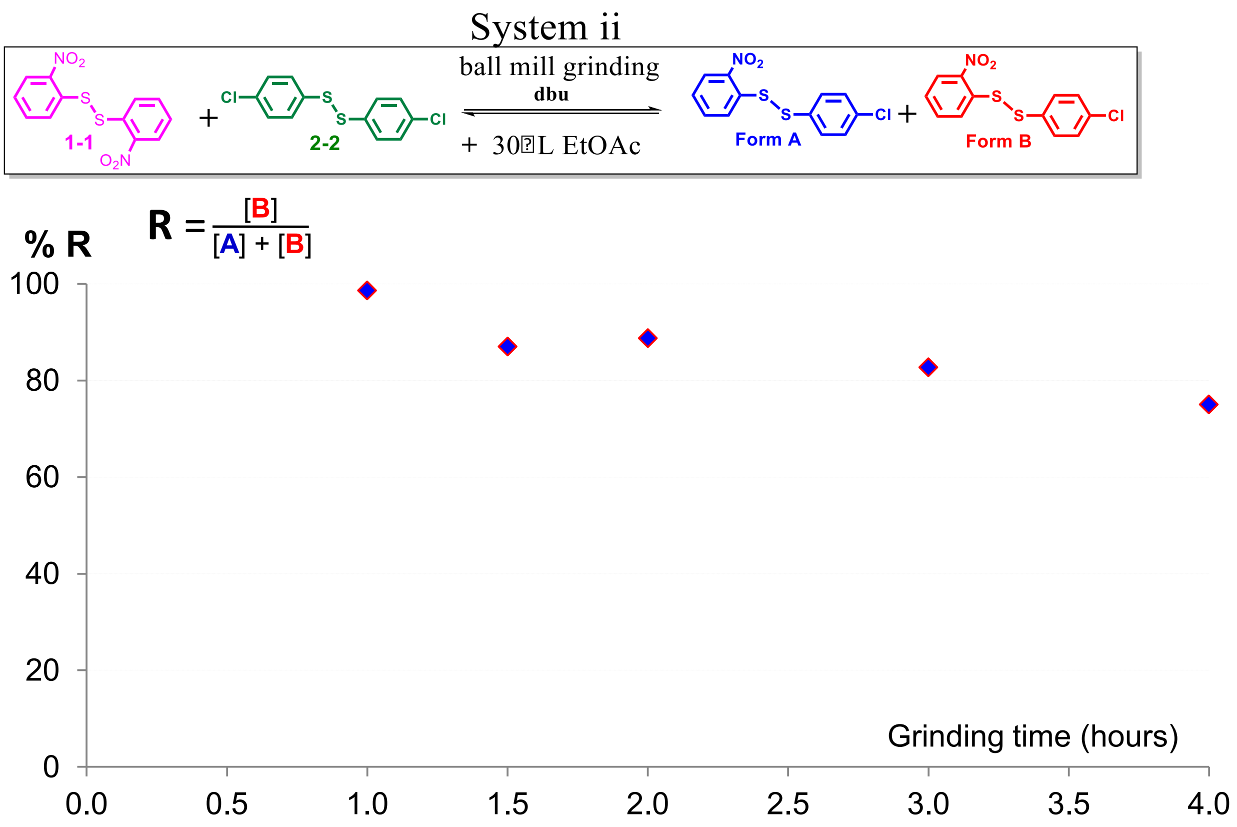

Figure S 72 Phase equilibria of LAG reaction of system ii with 30 μL EtOAc at different grinding times

9.3 Chemical and phase equilibria of ball mill LAG with 25μL Chloroform of system ii at different grinding times

Chloroform was used as a LAG solvent in ball mill LAG grinding of system ii as shown in Section 8.7. 25μL $CHCl_3$ leads to the formation by ball mill LAG of a mixture of **Form B** (78%M) and **Form A** (28%M). We found that 1.5 hours of ball mill grinding were sufficient to reach chemical (See Table S 38) and phase equilibrium (See Table S39).

Table S 73: Experimental details and HPLC results of the chemical composition of LAG reaction with 25μL $CHCl_3$ at different grinding times

Study of chemical equilibrium in ball mill LAG using Procedure 1

2%M dbu + **25 μL $CHCl_3$** added to 0.68 mmol **DCC** ([**1-1**] + [**2-2**] + [**1-2**])

Reagents:	$(2NO_2PhS)_2$	$(4ClPhS)_2$	$CHCl_3$ added to powder		**HPLC conditions**		
MW:	308.33	287.23			Zorbax XDB C18, 1.8 μm; 4.6x50 mm		
%M initial	50%M	50%M	MW (g/mol)	Density (g/mL)	A: H_2O+0.1% FA; B: MeCN+0.1%FA		
mmol	0.34	0.34			0-2min 75-85%B;		
mg	104.8 mg	97.6 mg	119.30	1.4793	2ml/min; 60°C; 259 nm (8nm bandwidth)		
					**HPLC results**		
grinding time @ 30 Hz to equilibrium	$(2NO_2PhS)_2$ weighed (mg)	$(4ClPhS)_2$ weighed (mg)	$CHCl_3$ μL	$[CHCl_3]/[DCC]$ (mol/mol)	$(2NO_2PhS)_2$ (1-1) %M	Product (1-2) %M	$(4ClPhS)_2$ (2-2) %M
**0.5 h**	104.81	97.70	**25**	0.46	5.8	**89.3**	4.9
**1.0 h**	104.83	97.68	**25**	0.46	2.8	**93.6**	3.5
**1.5 h**	104.83	97.71	**25**	0.46	5.2	**90.4**	4.3
**2.0 h**	104.84	97.70	**25**	0.46	3.3	**94.0**	2.7

[DCC] is the concentration of the dynamic covalent chemistry reaction at any time =[1-1]+[2-2]+[1-2]

[1-2] = **Form A** + **Form B**

Table S 74 Tabulation of Rietveld refinement of PXRD data of the phase composition of LAG reaction with 25μL $CHCl_3$ at different grinding times

2%M dbu+**25μL $CHCl_3$** added to 0.68 mmol DCC ([**1-1**]+[**2-2**]+[**Form A**]+[**Form B**])

grinding time @ 30Hz to equilib.	$CHCl_3$ added to powder		1-1		2-2		Form B		Form A		[A]+[B]	R = [B] / ([A]+[B])
	$CHCl_3$ μL	$[CHCl_3]/[DCC]$ (mol/mol)	%M	e.s.d. mol%	%M	e.s.d. mol%	%M	e.s.d. mol%	%M	e.s.d. mol%	total polymorph	% R
**0.5 h**	**25**	**0.46**	5	0.2	4.3	0.2	**88.9**	0.3	**1.5**	0.2	90.4	**98.3**
**1.0 h**	**25**	**0.46**	4	0.4	2.8	0.3	**74.8**	0.7	**18.9**	0.6	93.7	**79.8**
**1.5 h**	**25**	**0.46**	5	0.3	3.9	0.2	**90.1**	0.3	**1.2**	0.1	91.3	**98.7**
**2.0 h**	**25**	**0.46**	3	0.2	2.2	0.2	**78.2**	0.4	**17**	0.3	95.2	**82.1**

[DCC] is the concentration of the dynamic covalent chemistry reaction at any time =[**1-1**]+[**2-2**]+[**Form A**]+[**Form B**]

[**1-2**] = **Form A** + **Form B** [**A**] ≡ [**Form A**] [**B**] ≡ [**Form B**]

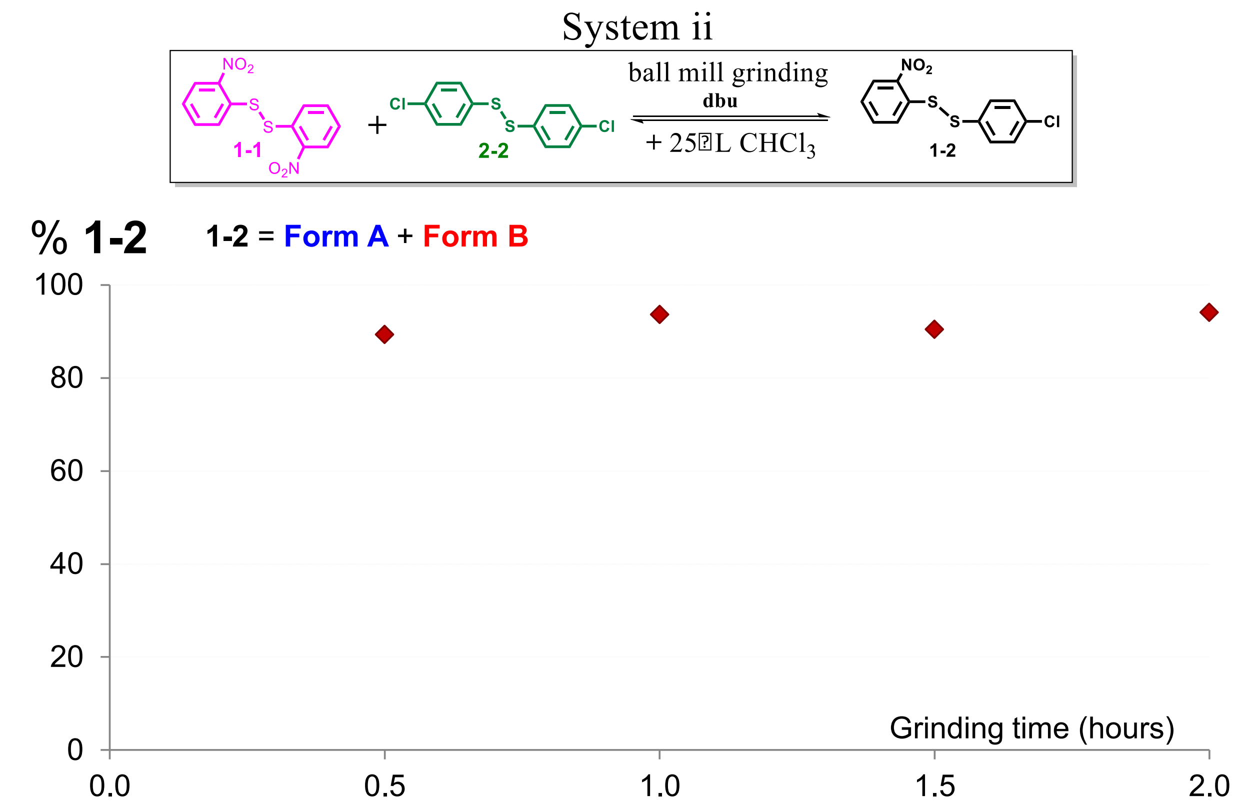

Figure S 73 Chemical equilibria of LAG reaction of system ii with 25 μL $CHCl_3$ at different grinding times

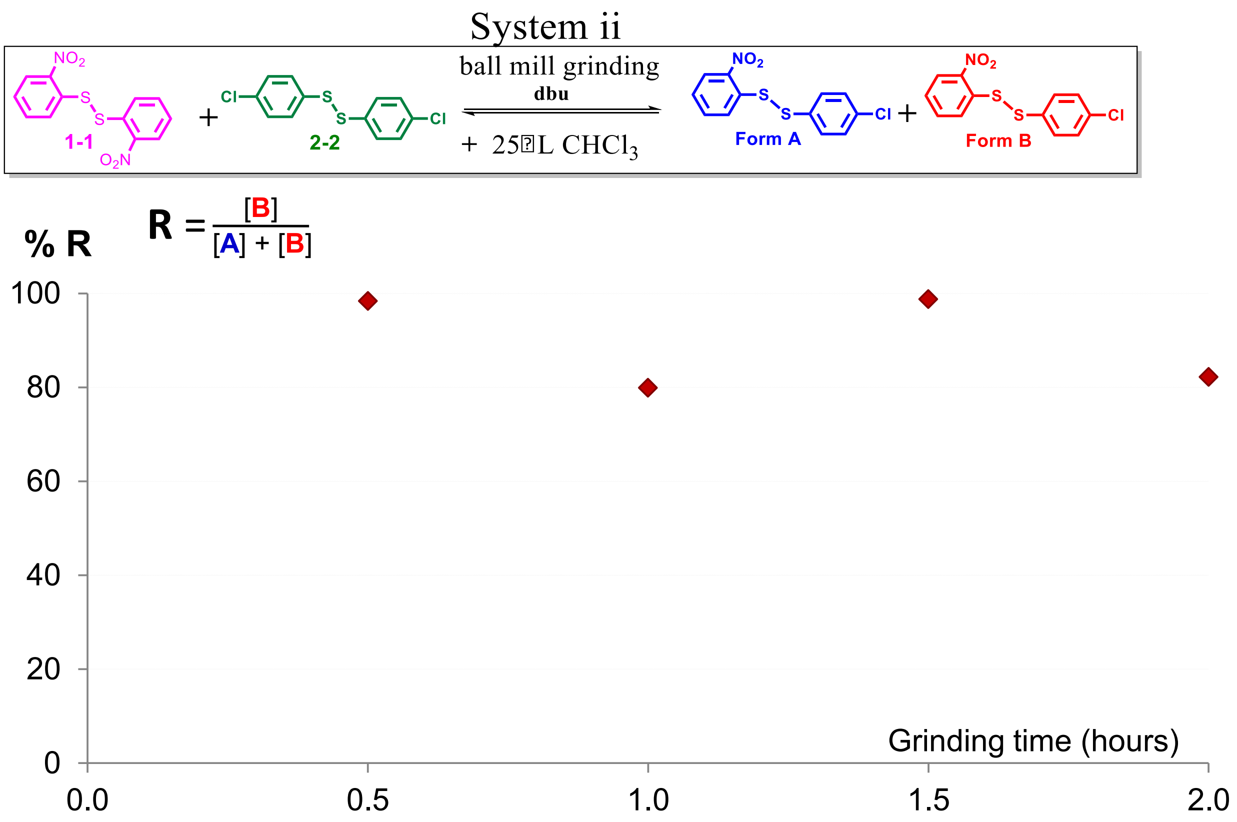

Figure S 74 Phase equilibria of LAG reaction of system ii with 25 μL $CHCl_3$ at different grinding times

9.4 Chemical and phase equilibria of ball mill LAG with 60μL DCM of system ii at different grinding times

Dichloromethane was used as a LAG solvent in ball mill LAG grinding of system ii as shown in Section 8.8. 60μL DCM leads to the formation by ball mill LAG of **Form B** predominantly. We found that 4 hours of ball mill grinding was excessive to reach chemical (See Table S 41) and phase equilibrium (See Table S42), leading to some degradation. Shorter ball mill grinding like 1 hour is probably sufficient to achieve chemical and phase equilibrium avoiding unnecessary degradation.

Table S 75: Experimental details and HPLC results of the chemical composition of LAG reaction with 60μL DCM at different grinding times

Study of chemical equilibrium in ball mill LAG using Procedure 2							
2%M dbu + 60 μL **DCM** added to 0.68 mmol **DCC** ([**1-1**] + [**2-2**] + [**1-2**])							
Reagents:	**$(2NO_2PhS)_2$**	**$(4ClPhS)_2$**	DCM added		**HPLC conditions**		
MW:	308.33	287.23	to powder		Zorbax XDB C18, 1.8 μm; 4.6x50 mm		
%M initial	50%M	50%M	MW	Densiity	A: H_2O+0.1% FA; B: MeCN+0.1%FA		
mmol	0.34	0.34	(g/mol)	(g/mL)	0-2min 75-85%B;		
mg	104.8 mg	97.6 mg	89.93	1.3943	2ml/min; 60°C; 259 nm (8nm bandwidth)		
					**HPLC results**		
grinding time @ 30 Hz to equilibrium	$(2NO_2PhS)_2$ weighed (mg)	$(4ClPhS)_2$ weighed (mg)	DCM μL	[DCM]/[DCC] (mol/mol)	$(2NO_2PhS)_2$ (1-1) %M	Product **(1-2)** %M	$(4ClPhS)_2$ (2-2) %M
**1.0 h**	104.82	97.69	60	1.37	11	**78.6**	10.5
**2.0 h**	104.85	97.69	60	1.37	11.3	**78.2**	10.5
[DCC] is the concentration of the dynamic covalent chemistry reaction at any time =[1-1]+[2-2]+[1-2]							
[1-2] = **Form A** + **Form B**							

Table S 76 Tabulation of Rietveld refinement of PXRD data of the phase composition of LAG reaction with 60μL DCM at different grinding times

Study of phase equilibrium in ball mill LAG using Procedure 2												
2%M dbu+**60μL DCM** added to 0.68 mmol DCC ([**1-1**]+[**2-2**]+[**Form A**]+[**Form B**])												
grinding time @ 30Hz to equilib.	DCM added to powder		**1-1**		**2-2**		**Form B**		**Form A**		[A]+[B]	**R = [B] / ([A]+[B])**
	DCM μL	[DCM]/[DCC] (mol/mol)	%M	e.s.d. mol%	%M	e.s.d. mol%	%M	e.s.d. mol%	%M	e.s.d. mol%	total polymorph	**% R**
**1 h**	60	**1.37**	14	0.2	11	0.2	**75**	0.3	**0.4**	0.1	75.1	**99 %**
**2 h**	60	**1.37**	11	0.5	8.9	0.4	**79**	0.7	**1.1**	0.3	80.2	**99 %**
[DCC] is the concentration of the dynamic covalent chemistry reaction at any time =[**1-1**]+[**2-2**]+[**Form A**]+[**Form B**]												
[**1-2**] = **Form A** + **Form B** [**A**] ≡ [**Form A**] [**B**] ≡ [**Form B**]												

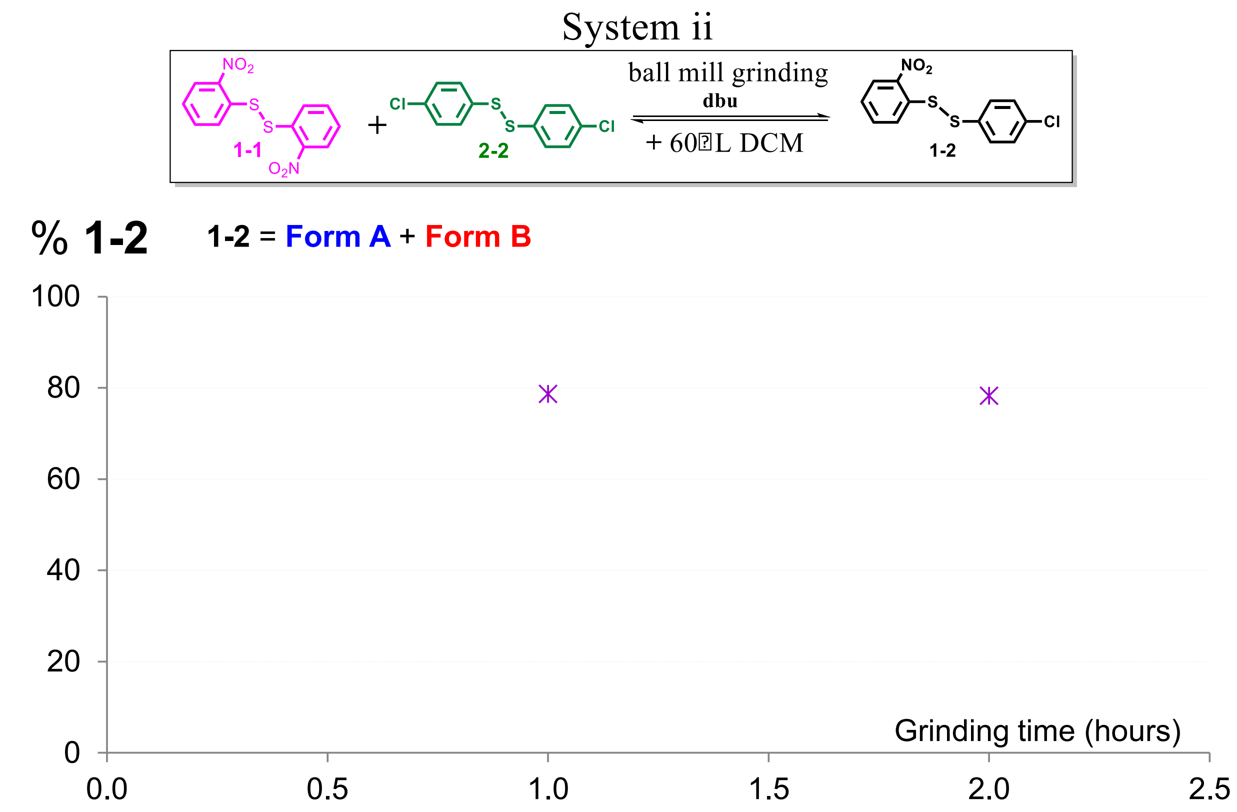

Figure S 75 Chemical equilibria of LAG reaction of system ii with 60μL DCM at different grinding times

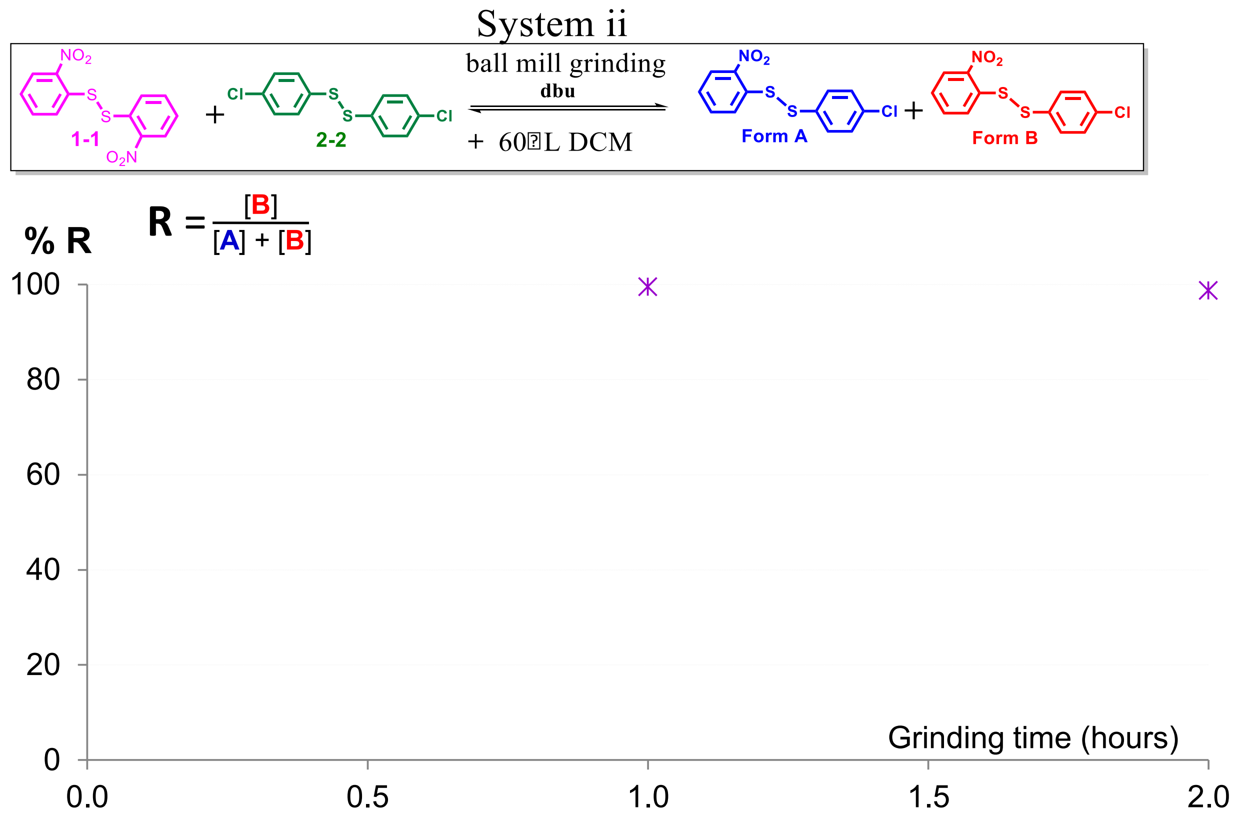

Figure S 76 Phase equilibria of LAG reaction of system ii with 60 μL DCM at different grinding times

10 Determination of the solubility of Form A and Form B in System ii in 15 solvents

The procedure to determine the solubility of **Form B** and **Form A** in 15 solvents in described in Section 4.7.

10.1 HPLC Method used for the quantitative determination of saturated solutions of Form A &Form B

HPLC column: 1.8μm Zorbax XDB C18, (4.6 × 50 mm)

HPLC mobile phase: A(water); B(MeCN) 95% B; 2mL/min; 50°C; 0.5uL injection; 240nm (8nm bandwidth) with reference 550nm(100nm bandwidth).

10.1.1 Linearity of HPLC method for Form B; applicable to Form A

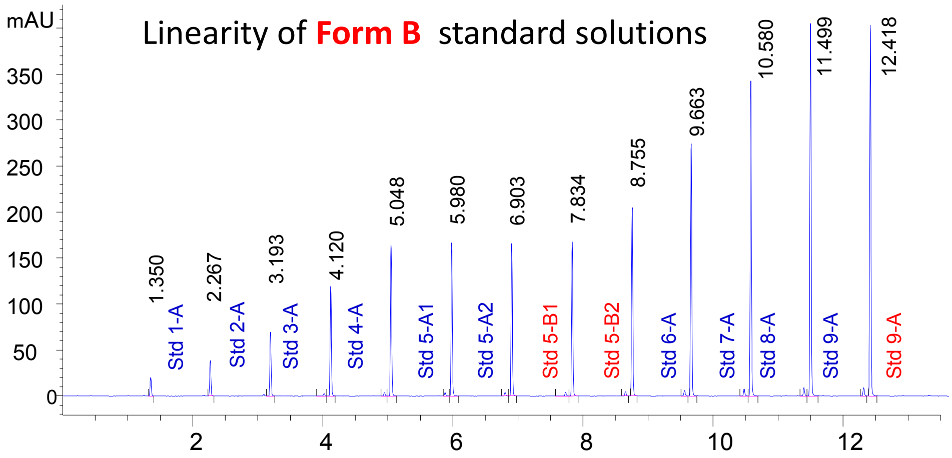

Figure S 77 HPLC chromatogram for the linearity experiment for **Form B**; applicable to **Form A**
Programmed injections of solutions of **1-2** in MeCN from Std 1=0.19 mg/ mL; Std 2= 0.37 mg/ mL; Std 3= 0.68 mg/ mL; Std 4= 1.15 mg/ mL; Std 5= 1.52 mg/ mL; Std 6= 2.03 mg/ mL; Std 7= 2.70mg/ mL; Std 8= 3.38mg/ mL; Std 9= 4.05mg/ mL. Two different stock solution of **1-2** used: Stock Std A: 4.056mg/mL; Stock Std B: 4.023mg/mL;

Table S 77 Tabulation of Peak Areas for linearity experiment for Form B; applicable to **Form A**

	Linearity of $2NO_2PhSSPh4Cl$ **Form B**					
	Std A= 25.80mg/10 mL MeCN			Std B= 25.64mg/10 mL MeCN		
Standard solution	Form **B** stock used (mg/mL)	**Form B** Aliquot (µL)	solvent MeCN (µL added)	**Form B** concentration mg/mL	HPLC time (min)	**form B** HPLC Peak Area
Std 1-A	2.580	50	1000	**0.123**	2.138	**27.3**
Std 2-A	2.580	100	1000	**0.235**	2.954	**52.1**
Std 3-A	2.580	200	1000	**0.430**	3.777	**94.3**
Std 4-A	2.580	400	1000	**0.737**	4.594	**161.9**
Std 5-A1	2.580	600	900	**1.032**	5.426	**224.7**
Std 5-A2	2.580	600	900	**1.032**	6.246	**227.3**
Std 5-B1	2.564	600	900	**1.026**	7.071	**225.4**
Std 5-B2	2.564	600	900	**1.026**	7.898	**220.1**
Std 6-A	2.580	600	600	**1.290**	8.716	**281.2**
Std 7-A	2.580	1000	500	**1.720**	9.522	**374.3**
Std 8-A	2.580	1000	200	**2.150**	10.341	**466.2**
Std 9-A	2.580	1000	0	**2.580**	11.16	**555.0**
Std 9-B	2.564	1000	0	**2.564**	11.16	**551.4**

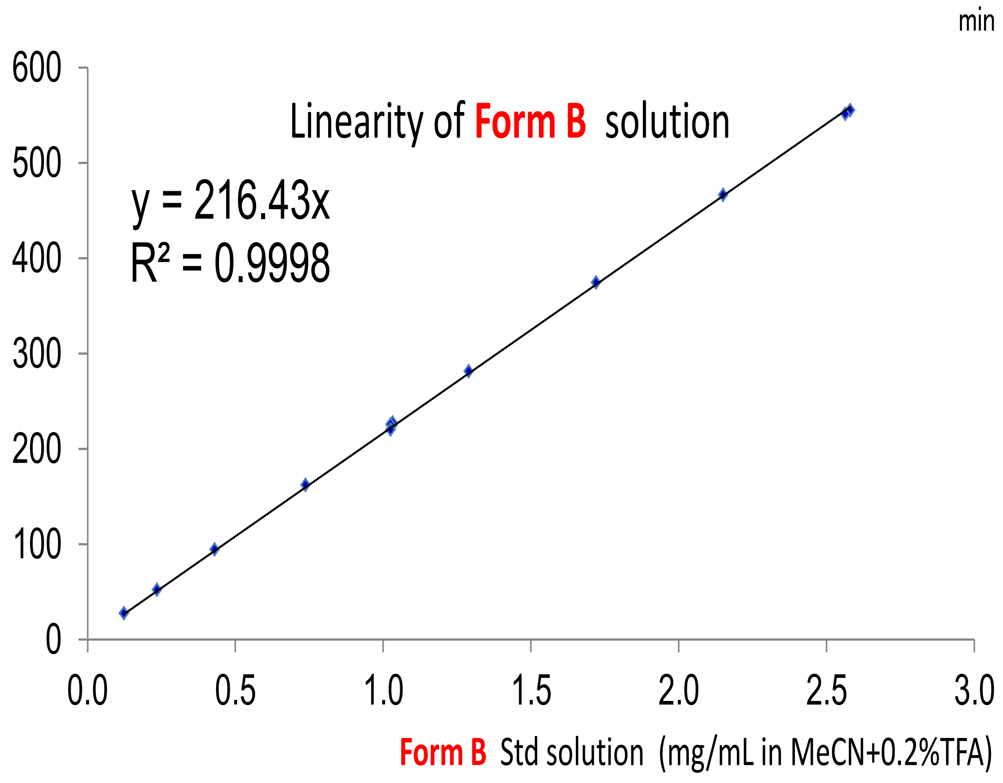

Figure S 78 Linearity curve for the determination of solubility of **Form B** by HPLC; applies to **Form A**

10.2 Solubility of Form B in water, MeOH, EtOH, IPA and cyclohexane

Table S 78 Tabulation of experimental data to determine solubility of Form B in water, MeOH, EtOH, IPA and cyclohexane (Solvents of low solubility of Form B: ≤ 6mg/mL)

Solubility of Form B in water, MeOH, EtOH, IPA and cycloHexane

Std 1 stock	10.20	mg/mL	Standard Stock and all HPLC solution prepared in MeCN+0.2%TFA							
Std 2 stock	10.06	mg/mL								
	**Std 1** dilution	**Std 2** dilution	**water** supernat	**MeOH** supernat	**EtOH** supernat	**IPA** supernat	**cHexane** supernat	**Std 1** dilution	**Std 2** dilution	**Std** dilution Average
Aliquot. Std stock or supntant (ul)	1000	1000	50	25	25	25	25	1000	1000	Duppl. Std1/Std2
to µL MeCN	0	0	0	50	100	50	100	0	0	**101.5%**
**Peak Area**	**219**	**218**	**0**	**257**	**225**	**238**	**252**	**218**	**219**	**219**
conc. suptnant (mg/mL)	**Point 1**		**0.0**	**3.3**	**3.6**	**5.2**	**5.8**	slurry stirred for		10 min
Aliquot. Std stock or supntant (ul)	1000	1000	50	25	25	25	25	1000	1000	Duppl. Std1/Std2
to µL MeCN	0	0	0	50	100	50	100	0	0	**101.7%**
**Peak Area**	**215**	**215**	**0**	**242**	**222**	**227**	**261**	**215**	**217**	**215**
conc. suptnant (mg/mL)	**Point 2**		**0.0**	**3.2**	**3.4**	**5.2**	**6.1**	slurry stirred for		10 min
Aliquot. Std stock or supntant (ul)	1000	1000	50	25	25	25	25	1000	1000	Duppl. Std1/Std2
to µL MeCN	0	0	0	50	100	50	100	0	0	**102.6%**
**Peak Area**	**214**	**218**	**0**	**224**	**230**	**248**	**276**	**215**	**217**	**216**
conc. suptnant (mg/mL)	**Point 3**		**0.0**	**3.5**	**3.1**	**5.4**	**6.5**	slurry stirred for		10 min
Aliquot. Std stock or supntant (ul)	1000	1000	50	25	25	25	25	1000	1000	Duppl. Std1/Std2
to µL MeCN	0	0	0	50	100	50	100	0	0	**100.9%**
**Peak Area**	**215**	**215**	**0**	**253**	**223**	**247**	**296.6**	**215**	**213**	**214**
conc. suptnant (mg/mL)	**Point 4**		**0.0**	**3.5**	**3.6**	**5.3**	**7.0**	slurry stirred for		10 min
Aliquot. Std stock or supntant (ul)	1000	1000	50	25	25	25	25	1000	1000	Duppl. Std1/Std2
to µL MeCN	0	0	0	50	100	50	100	0	0	**101.3%**
**Peak Area**	**215.2**	**216.5**	**0**	**247.6**	**226.8**	**234.7**	**265.9**	**216.7**	**215**	**215.9**
conc. suptnant (mg/mL)	**Point 5**		**0.0**	**3.3**	**3.5**	**5.3**	**6.2**	slurry stirred for		10 min
Aliquot. Std stock or supntant (ul)	1000	1000	50	25	25	25	25	1000	1000	Duppl. Std1/Std2
to µL MeCN	0	0	0	50	100	50	100	0	0	**100.4%**
**Peak Area**	**217.4**	**215.8**	**0**	**247.3**	**224.4**	**232.2**	**267.6**	**217.1**	**214.5**	**216.2**
conc. suptnant (mg/mL)	**Point 6**		**0.0**	**3.3**	**3.5**	**5.3**	**6.3**	slurry stirred for		10 min

Solubility (mg/mL) of Form B in in given solvents

	Points 1-6 averaged	Points 1-6 averaged	Points 1-6 averaged	Points 1-6 averaged	Points 1-6 averaged
	**water**	**MeOH**	**EtOH**	**IPA**	**cyclo-Hexane**
**mg/mL solvent**	**0.0**	**3.3**	**3.4**	**5.3**	**6.3**
**mmol/mol solvent**	**0.0**	**0.5**	**0.7**	**1.4**	**2.3**

The duplication of the 2 independently prepared standard solutions is good, between 100.4-102.6%.

10.3 Solubility of Form B in MeCN, DMSO, Acetone, EtOAc and Toluene

Table S 79 Tabulation of experimental data to determine solubility of Form B in MeCN, DMSO, Acetone, EtOAc and Toluene (Solvents of medium solubility of Form B: 68-130 mg/mL excluding MeCN (19 mg/mL))

Solubility of Form B in MeCN, DMSO, Acetone, Ethyl Acetate and Toluene

Std 1 stock	12.34	mg/mL	Standard Stock and all HPLC solution prepared in MeCN+0.2%TFA							
Std 2 stock	12.16	mg/mL								
	**Std 1 dilution**	**Std 2 dilution**	**MeCN supernat**	**DMSO supernat**	**Acetone supernat**	**EtOAc supernat**	**Toluene supernat**	**Std 1 dilution**	**Std 2 dilution**	**Std dilution Average**
Aliquot. Std stock or supntant (ul)	1000	1000	25	25	25	25	10	1000	1000	Duppl. Std1/Std2
to µL MeCN	0	0	500	1500	1500	1500	1000	0	0	**101.5%**
**Peak Area**	**273**	**268**	**163**	**202**	**235**	**237**	**381**	**273**	**270**	**271**
conc. supntant (mg/mL)	**Point 1**		**15**	**56**	**65**	**65**	**174**	slurry stirred for		10 min
Aliquot. Std stock or supntant (ul)	1000	1000	25	25	25	25	10	1000	1000	Duppl. Std1/Std2
to µL MeCN	0	0	400	1300	1500	1500	1500	0	0	**99.8%**
**Peak Area**	**277**	**271**	**229**	**260**	**257**	**285**	**168**	**273**	**270**	**273**
conc. supntant (mg/mL)	**Point 2**		**17**	**62**	**70**	**78**	**114**	slurry stirred for		10 min
Aliquot. Std stock or supntant (ul)	1000	1000	25	25	25	25	10	1000	1000	Duppl. Std1/Std2
to µL MeCN	0	0	400	1300	1500	1500	1300	0	0	**100.3%**
**Peak Area**	**278**	**271**	**250**	**289**	**271**	**275**	**199**	**273**	**274**	**274**
conc. supntant (mg/mL)	**Point 3**		**19**	**68**	**74**	**75**	**116**	slurry stirred for		10 min
Aliquot. Std stock or supntant (ul)	1000	1000	25	25	25	25	10	1000	1000	Duppl. Std1/Std2
to µL MeCN	0	0	400	1300	1500	1500	1000	0	0	**102.2%**
**Peak Area**	**277**	**280**	**237**	**283**	**279**	**278**	**303.7**	**276**	**277**	**278**
conc. supntant (mg/mL)	**Point 4**		**18**	**66**	**75**	**75**	**135**	slurry stirred for		10 min
**Aliquot. Std stock or supntant (ul)**	**1000**	**1000**	**25**	**25**	**25**	**25**	**10**	**1000**	**1000**	**Duppl. Std1/Std2**
**to µL MeCN**	**0**	**0**	**400**	**1300**	**1500**	**1500**	**1000**	**0**	**0**	**100.6%**
**Peak Area**	**280.3**	**276.6**	**246.5**	**295.6**	**282.6**	**277.9**	**311.5**	**278.9**	**278**	**278.5**
**conc. supntant (mg/mL)**	**Point 5**		**18**	**69**	**76**	**75**	**138**	**slurry stirred fo**		**10 min**
**Aliquot. Std stock or supntant (ul)**	**1000**	**1000**	**25**	**25**	**25**	**25**	**10**	**1000**	**1000**	**Duppl. Std1/Std2**
**to µL MeCN**	**0**	**0**	**400**	**1300**	**1500**	**1500**	**1000**	**0**	**0**	**102.1%**
**Peak Area**	**272.7**	**277.8**	**254.1**	**296.9**	**293.4**	**279.8**	**323.8**	**276.8**	**274.8**	**275.5**
**conc. supntant (mg/mL)**	**Point 6**		**19**	**70**	**80**	**76**	**145**	**slurry stirred fo**		**10 min**

Solubility (mg/mL) of Form B in in given solvents

	Points 2-6 averaged	Points 2-6 averaged	Points 2-6 averaged	Points 2-6 averaged	Points 2-6 averaged
	**MeCN**	**DMSO**	**Acetone**	**EtOAc**	**Toluene**
**mg/mL solvent**	**19**	**68**	**76**	**75**	**130**
**mmol/mol solvent**	**3.3**	**16**	**19**	**25**	**47**

The duplication of the 2 independently prepared standard solutions is good, between 99.8-102.2%.

10.4 Solubility of Form B in Benzene, DCM, DMF, $CHCl_3$ and THF

Table S 80 Tabulation of experimental data to determine solubility of Form B in Benzene, DCM, DMF, $CHCl_3$ and THF (Solvents of high solubility of Form B: 140-330 mg/mL)

Solubility of Form B in Benzene, DCM, DMF, $CHCl_3$ and THF

Std 1 stock	12.74	mg/mL	Standard Stock and all HPLC solution prepared in MeCN+0.2%TFA							
Std 2 stock	13.02	mg/mL								
	**Std 1 dilution**	**Std 2 dilution**	**Benzene supernat**	**DCM supernat**	**DMF supernat**	**$CHCl_3$ supernat**	**THF supernat**	**Std 1 dilution**	**Std 2 dilution**	**Std dil. Average**
Aliquot. Std stock or supntant (ul)	1000	1000	10	25	10	10	10	1000	1000	Duppl. Std1/Std2
to µL MeCN	0	0	1500	5000	1500	1500	3000	0	0	**99.1%**
**Peak Area**	**274**	**276**	**176**	**257**	**283**	**344**	**224**	**274**	**278**	**275**
conc. supntant (mg/mL)	**Point 1**		**125**	**242**	**200**	**243**	**315**	slurry stirred for		10 min
Aliquot. Std stock or supntant (ul)	1000	1000	10	25	10	10	10	1000	1000	Duppl. Std1/Std2
to µL MeCN	0	0	1500	5000	1500	1700	3000	0	0	**99.1%**
**Peak Area**	**272**	**278**	**186**	**265**	**275**	**293**	**229**	**273**	**275**	**274**
conc. supntant (mg/mL)	**Point 2**		**131**	**249**	**195**	**234**	**322**	slurry stirred for		10 min
Aliquot. Std stock or supntant (ul)	1000	1000	10	25	10	10	10	1000	1000	Duppl. Std1/Std2
to µL MeCN	0	0	1500	5000	1500	1700	3000	0	0	**100.1%**
**Peak Area**	**275**	**277**	**192**	**275**	**302**	**314**	**228**	**273**	**283**	**277**
conc. supntant (mg/mL)	**Point 3**		**136**	**258**	**212**	**251**	**320**	slurry stirred for		10 min
Aliquot. Std stock or supntant (ul)	1000	1000	10	25	10	10	10	1000	1000	Duppl. Std1/Std2
to µL MeCN	0	0	1500	5000	1500	1700	3000	0	0	**100.0%**
**Peak Area**	**273**	**281**	**201**	**292**	**303**	**310**	**238**	**271**	**276**	**275**
conc. supntant (mg/mL)	**Point 4**		**142**	**274**	**214**	**248**	**334**	slurry stirred for		10 min
Aliquot. Std stock or supntant (ul)	1000	1000	10	25	10	10	10	1000	1000	Duppl. Std1/Std2
to µL MeCN	0	0	1500	5000	1500	1700	3000	0	0	**98.6%**
**Peak Area**	**274**	**278**	**208**	**281**	**316**	**312**	**242**	**274**	**275**	**275**
conc. supntant (mg/mL)	**Point 5**		**147**	**264**	**223**	**249**	**340**	slurry stirred for		10 min
Aliquot. Std stock or supntant (ul)	1000	1000	10	25	10	10	10	1000	1000	Duppl. Std1/Std2
to µL MeCN	0	0	1500	5000	1500	1700	3000	0	0	**101.1%**
**Peak Area**	**272**	**276**	**202**	**281**	**299**	**308**	**239**	**263**	**276**	**272**
conc. supntant (mg/mL)	**Point 6**		**142**	**264**	**214**	**246**	**337**	slurry stirred for		10 min

Solubility (mg/mL) of Form B in in given solvents

	Benzene	DCM	DMF	$CHCl_3$	THF
	Points 2-6 averaged	Points 2-6 averaged	Points 2-6 averaged	Points 2-6 averaged	Points 2-6 averaged
**mg/mL solvent**	**141**	**266**	**215**	**248**	**334**
**mmol/mol solvent**	**42**	**58**	**43**	**67**	**91**

The duplication of the 2 independently prepared standard solutions is good, between 98.6-101.1%.

10.5 Summary of solubility data: Form B in 15 solvents

Table S 81: Tabulation of the summary of the solubility of Form B in 15 solvents

Solvent investigated	water	MeOH	EtOH	IPA	MeCN	DMSO	cyclo-Hexane	Acetone	EtOAc	DMF	Toluene	Benzene	$CHCl_3$	DCM	THF
MW Solvent g/mol	18.02	32.04	46.07	60.10	41.05	78.13	84.16	58.08	88.11	59.07	92.14	78.12	119.30	89.93	72.11
density Solvent g/mL	0.9974	0.7872	0.7848	0.7815	0.7760	1.0958	0.7781	0.7849	0.8945	0.9995	0.8619	0.8690	1.4793	1.3943	0.8837
**Form B** (mg/mL)	**0.0**	**3.3**	**3.4**	**5.3**	**19**	**68**	**6.3**	**76**	**75**	**215**	**130**	**141**	**248**	**266**	**334**
**Form B** mmol /mol **solvent**	**0.0**	**0.5**	**0.7**	**1.4**	**3.3**	**16.4**	**2.3**	**18.9**	**24.8**	**42.7**	**46.6**	**42.5**	**67.2**	**57.5**	**91.4**

MW of Form B is 297.77 g/mol.

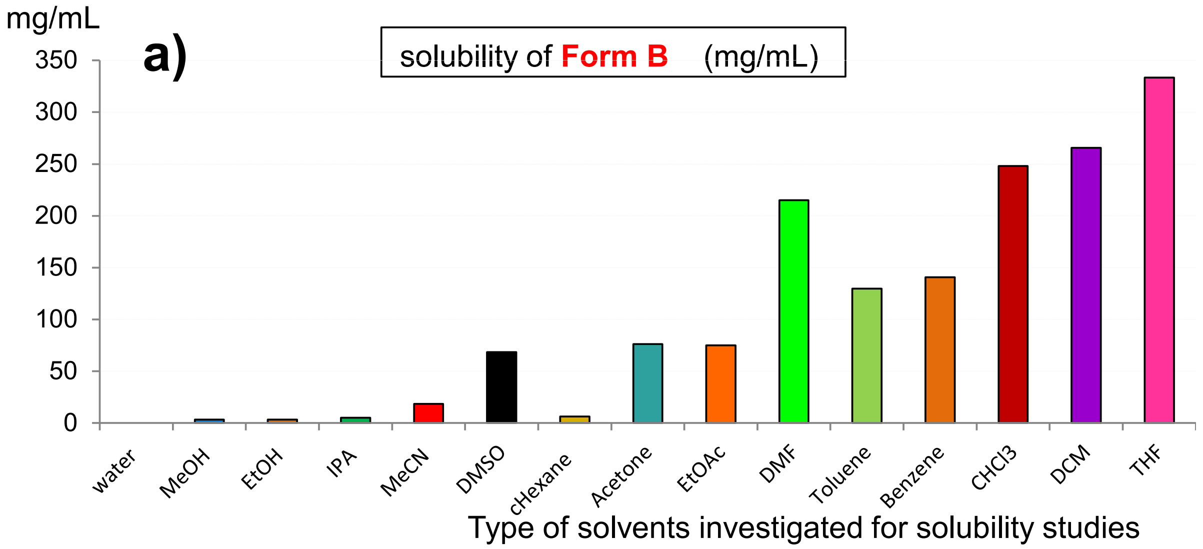

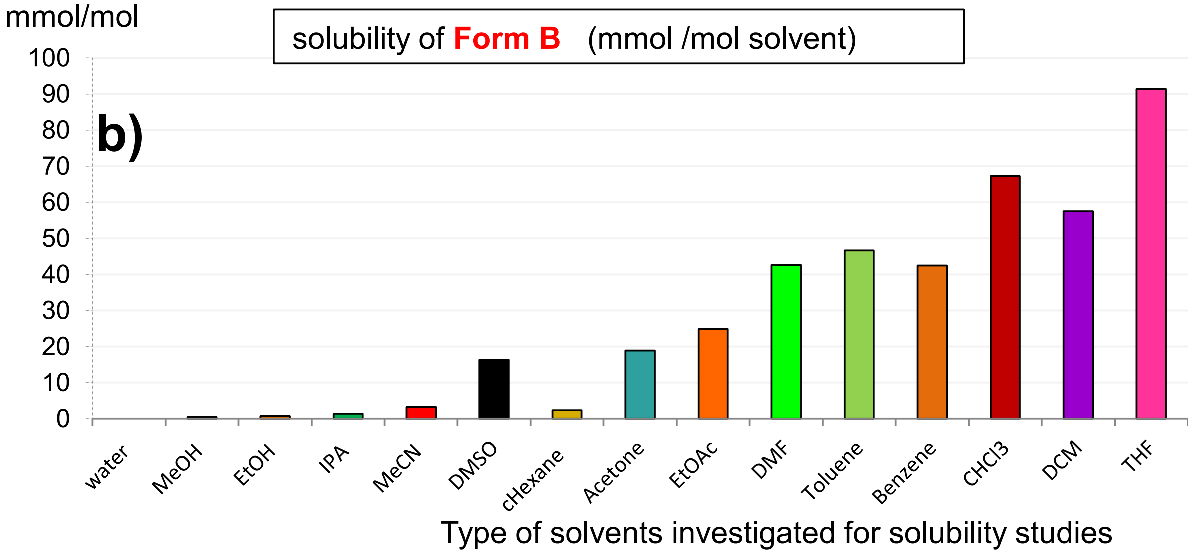

Figure S 79: Comparison of the solubility of Form B in 15 solvents, a) mg /mL and b) mmol /mol solvent

10.6 Solubility of Form A in MeCN, MeOH, EtOH, IPA and cyclohexane

Table S 82 Tabulation of experimental data to determine solubility of **Form A** in MeCN, MeOH, EtOH, IPA and cyclohexane (Solvents of low solubility of Form A: ≤ 3 mg /mL).
Note: **Form A** is transformed to **Form B** (thermodynamic stable polymorph at ambient conditions) within a few hours for MeCN and cyclohexane. For alcohols full transformation takes over a few hours but less than 1 week. See Section 12.2. So, the solubility determined is really that of **Form B**.

Solubility of Form A in MeCN, MeOH, EtOH, IPA and cycloHexane										
Std 1 stock	10.33	mg/mL	Standard Stock and all HPLC solution prepared in MeCN+0.2%TFA							
Std 2 stock	10.14	mg/mL								
	Std 1 dilution	Std 2 dilution	MeCN supernat	IPA supernat	MeOH supernat	EtOH supernat	cHexane supernat	Std 1 dilution	Std 2 dilution	Std dilution Average
Aliquot. Std stock or supntant (ul)	1000	1000	25	25	25	25	25	1000	1000	Duppl. Std1/Std2
to µL MeCN	0	0	250	50	50	100	100	0	0	100.4%
Peak Area	231	229	325	256	174	258	604	230	226	229
conc. supntant (mg/mL)	Point 1		16.0	3.4	2.3	5.8	13.5	slurry stirred for		10 min
Aliquot. Std stock or supntant (ul)	1000	1000	25	25	25	25	25	1000	1000	Duppl. Std1/Std2
to µL MeCN	0	0	500	50	70	100	500	0	0	102.5%
Peak Area	231	229	172	276	233	273	69	229	233	215
conc. supntant (mg/mL)	Point 2		16.1	3.7	4.0	6.1	6.5	slurry stirred for		10 min
Aliquot. Std stock or supntant (ul)	1000	1000	25	25	25	25	25	1000	1000	Duppl. Std1/Std2
to µL MeCN	0	0	400	50	70	100	300	0	0	100.1%
Peak Area	232	230	238	272	234	274	103	231	225	216
conc. supntant (mg/mL)	Point 3		18.1	3.6	4.0	6.1	6.0	slurry stirred for		10 min
Aliquot. Std stock or supntant (ul)	1000	1000	25	25	25	25	25	1000	1000	Duppl. Std1/Std2
to µL MeCN	0	0	400	50	70	100	200	0	0	100.5%
Peak Area	233	227	238	294	253	295	138.5	230	230	214
conc. supntant (mg/mL)	Point 4		18.1	3.9	4.3	6.6	5.6	slurry stirred for		10 min
Aliquot. Std stock or supntant (ul)	1000	1000	25	25	25	25	25	1000	1000	Duppl. Std1/Std2
to µL MeCN	0	0	400	50	70	100	200	0	0	101.8%
Peak Area	233	231	228	307	271	297	171	231	233	216
conc. supntant (mg/mL)	Point 5		17.3	4.1	4.6	6.6	6.9	slurry stirred for		10 min
Aliquot. Std stock or supntant (ul)	1000	1000	25	25	25	25	25	1000	1000	Duppl. Std1/Std2
to µL MeCN	0	0	400	50	70	100	200	0	0	101.7%
Peak Area	230	231	236	302	262	293	162	231	228	216
conc. supntant (mg/mL)	Point 6		17.9	4.0	4.5	6.5	6.5	slurry stirred for		10 min

Solubility (mg/mL) of Form A in in given solvents					
	Points 2-6 averaged	Points 2-6 averaged	Points 2-6 averaged	Points 2-6 averaged	Points 2-6 averaged
	MeCN	MeOH	EtOH	IPA	cyclo-Hexane
mg/mL solvent	17.5	3.9	4.3	6.4	6.3
mmol/mol solvent	3.1	0.5	0.8	1.7	2.3

The duplication of the 2 independently prepared standard solutions is good, between 100.1-102.5%.

10.7 Solubility of Form A in Water, DMSO, Acetone, EtOAc and Toluene

Table S 83 Tabulation of experimental data to determine solubility of **Form A** in Water, DMSO, Acetone, EtOAc and Toluene (Solvents of medium solubility of **Form A**: 60-125 mg/mL) with the exception of water which is not soluble at all.

Note: **Form A** is quickly transformed to **Form B** (thermodynamic stable polymorph at ambient conditions) except for water, which is transformed being very slow. See 12.2 . So, the solubility determined if really that of **Form B**.

Solubility of Form A in Water, DMSO, Acetone, Ethyl Acetate and Toluene

Std 1 stock	12.19	mg/mL	Standard Stock and all HPLC solution prepared in MeCN+0.2%TFA							
Std 2 stock	10.07	mg/mL								
	**Std 1 dilution**	**Std 2 dilution**	**Water supernat**	**DMSO supernat**	**Acetone supernat**	**EtOAc supernat**	**Toluene supernat**	**Std 1 dilution**	**Std 2 dilution**	**Std dil Average**
Aliquot. Std stock or supntant (ul)	1000	1000	50	25	25	25	10	1000	1000	Duppl. Std1/Std2
to µL MeCN	0	0	0	1300	1500	1500	1000	0	0	**101.7%**
**Peak Area**	**259**	**220**	**0**	**249**	**263**	**248**	**292**	**259**	**215**	**238**
conc. supntant (mg/mL)	**Point 1**		**0**	**62**	**75**	**71**	**138**	slurry stirred for		10 min
Aliquot. Std stock or supntant (ul)	1000	1000	50	25	25	25	10	1000	1000	Duppl. Std1/Std2
to µL MeCN	0	0	0	1300	1500	1500	1300	0	0	**102.2%**
**Peak Area**	**253**	**216**	**3**	**241**	**263**	**269**	**183**	**256**	**215**	**235**
conc. supntant (mg/mL)	**Point 2**		**0**	**61**	**76**	**78**	**114**	slurry stirred for		10 min
Aliquot. Std stock or supntant (ul)	1000	1000	50	25	25	25	10	1000	1000	conc Std
to µL MeCN	0	0	0	1300	1500	1500	1300	0	0	**103.2%**
**Peak Area**	**254**	**218**	**2**	**244**	**274**	**262**	**192**	**254**	**215**	**235**
conc. supntant (mg/mL)	**Point 3**		**0**	**61**	**79**	**76**	**119**	slurry stirred for		10 min
Aliquot. Std stock or supntant (ul)	1000	1000	50	25	25	25	10	1000	1000	Duppl. Std1/Std2
to µL MeCN	0	0	0	1300	1500	1500	1300	0	0	**102.2%**
**Peak Area**	**254**	**216**	**3**	**246**	**276**	**269**	**189.2**	**255**	**215**	**235**
conc. supntant (mg/mL)	**Point 4**		**0**	**62**	**80**	**78**	**117**	slurry stirred for		10 min
Aliquot. Std stock or supntant (ul)	1000	1000	50	25	25	25	10	1000	1000	Duppl. Std1/Std2
to µL MeCN	0	0	0	1300	1500	1500	1300	0	0	**102.0%**
**Peak Area**	**254**	**214**	**7**	**257**	**293**	**289**	**199**	**252**	**213**	**233**
conc. supntant (mg/mL)	**Point 5**		**0**	**65**	**85**	**84**	**124**	slurry stirred for		10 min
Aliquot. Std stock or supntant (ul)	1000	1000	50	25	25	25	10	1000	1000	conc Std
to µL MeCN	0	0	0	1300	1500	1500	1300	0	0	**101.1%**
**Peak Area**	**256**	**215**	**0**	**255**	**276**	**284**	**219**	**256**	**212**	**235**
conc. supntant (mg/mL)	**Point 6**		**0**	**64**	**80**	**82**	**136**	slurry stirred for		10 min

Solubility (mg/mL) of Form A in in given solvents

	Points 1-6 averaged	Points 1-6 averaged	Points 1-6 averaged	Points 1-6 averaged	Points 1-6 averaged
	**water**	**DMSO**	**Acetone**	**EtOAc**	**Toluene**
**mg/mL solvent**	**0**	**62**	**79**	**78**	**125**
**mmol/mol solvent**	**0.0**	**15**	**20**	**26**	**45**

The duplication of the 2 independently prepared standard solutions is good, between 101.1-103.2%.

10.8 Solubility of Form A in Benzene, DCM, DMF, $CHCl_3$ and THF

Table S 84 Tabulation of experimental data to determine solubility of **Form A** in Benzene, DCM, DMF, $CHCl_3$ and THF (Solvents of high solubility of **Form A**: 140-340 mg/mL.
Note: **Form A** is quickly transformed to **Form B** (thermodynamic stable polymorph at ambient conditions). See Section 12.2 . So, the solubility determined is really that of **Form B**.

Solubility of Form A in Benzene, DCM, DMF, $CHCl_3$ and THF

Std 1 stock	10.33	mg/mL	Standard Stock and all HPLC solution prepared in MeCN+0.2%TFA							
Std 2 stock	10.14	mg/mL								
	**Std 1** dilution	**Std 2** dilution	**Benzene** supernat	**DCM** supernat	**DMF** supernat	**$CHCl_3$** supernat	**THF** supernat	**Std 1** dilution	**Std 2** dilution	**Std** dilution Average
Aliquot. Std stock or supntant (ul)	1000	1000	10	10	10	10	10	1000	1000	Duppl. Std1/Std2
to µL MeCN	0	0	1500	1500	1500	1700	3000	0	0	**100.1%**
**Peak Area**	**286**	**284**	**180**	**329**	**254**	**323**	**232**	**286**	**281**	**284**
conc. supntant (mg/mL)	**Point 1**		**126**	**230**	**178**	**255**	**323**	slurry stirred for		10 min
Aliquot. Std stock or supntant (ul)	1000	1000	10	10	10	10	10	1000	1000	Duppl. Std1/Std2
to µL MeCN	0	0	1500	1500	1500	1700	3000	0	0	**100.7%**
**Peak Area**	**286**	**292**	**197**	**370**	**269**	**309**	**251**	**288**	**279**	**215**
conc. supntant (mg/mL)	**Point 2**		**136**	**257**	**187**	**243**	**347**	slurry stirred for		10 min
Aliquot. Std stock or supntant (ul)	1000	1000	10	10	10	10	10	1000	1000	Duppl. Std1/Std2
to µL MeCN	0	0	1500	1500	1500	1700	3000	0	0	**96.8%**
**Peak Area**	**295**	**281**	**212**	**415**	**269**	**322**	**249**	**297**	**284**	**216**
conc. supntant (mg/mL)	**Point 3**		**146**	**285**	**185**	**251**	**341**	slurry stirred for		10 min
Aliquot. Std stock or supntant (ul)	1000	1000	10	10	10	10	10	1000	1000	Duppl. Std1/Std2
to µL MeCN	0	0	1500	1500	1500	1700	3000	0	0	**100.4%**
**Peak Area**	**233**	**227**	**208**	**378**	**266**	**314**	**240**	**230**	**230**	**214**
conc. supntant (mg/mL)	**Point 4**		**145**	**264**	**185**	**248**	**333**	slurry stirred for		10 min
Aliquot. Std stock or supntant (ul)	1000	1000	10	10	10	10	10	1000	1000	Duppl. Std1/Std2
to µL MeCN	0	0	1500	1500	1500	1700	3000	0	0	**100.9%**
**Peak Area**	**233**	**231**	**204**	**391**	**253**	**331**	**250**	**231**	**233**	**216**
conc. supntant (mg/mL)	**Point 5**		**141**	**270**	**175**	**258**	**345**	slurry stirred for		10 min
Aliquot. Std stock or supntant (ul)	1000	1000	10	10	10	10	10	1000	1000	Duppl. Std1/Std2
to µL MeCN	0	0	1500	1500	1500	1700	3000	0	0	**99.6%**
**Peak Area**	**230**	**231**	**216**	**377**	**270**	**317**	**246**	**231**	**228**	**216**
conc. supntant (mg/mL)	**Point 6**		**150**	**262**	**187**	**249**	**339**	slurry stirred for		10 min

Solubility (mg/mL) of Form A in in given solvents

	Points 2-6 averaged	Points 2-6 averaged	Points 2-6 averaged	Points 2-6 averaged	Points 2-6 averaged
	**Benzene**	**DCM**	**DMF**	**$CHCl_3$**	**THF**
**mg/mL solvent**	**144**	**267**	**183**	**250**	**341**
**mmol/mol solvent**	**43**	**58**	**36**	**68**	**94**

The duplication of the 2 independently prepared standard solutions is good, between 96.8-100.7%.

10.9 Summary of solubility data: Form A in 15 solvents

Table S 85: Tabulation of the summary of the solubility of **Form A** in 15 solvents

MW (solvent)	18.02	32.04	46.07	60.10	41.05	78.13	84.16	58.08	88.11	59.07	92.14	78.12	119.30	89.93	72.11
density Solvent g/mL	0.9974	0.7872	0.7848	0.7815	0.7760	1.0958	0.7781	0.7849	0.8945	0.9995	0.8619	0.8690	1.4793	1.3943	0.8837
Solvent investigated	**water**	**MeOH**	**EtOH**	**IPA**	**MeCN**	**DMSO**	**cyclo-Hexane**	**Acetone**	**EtOAc**	**DMF**	**Toluene**	**Benzene**	**$CHCl_3$**	**DCM**	**THF**
**Form A** (mg/mL)	**0.0**	**3.9**	**4.3**	**6.4**	**18**	**62**	**6.3**	**79**	**78**	**183**	**125**	**144**	**250**	**267**	**341**
**Form A** mmol/mol solvent	**0.0**	**0.5**	**0.8**	**1.7**	**3.1**	**15**	**2**	**20**	**26**	**36**	**45**	**43**	**68**	**58**	**94**

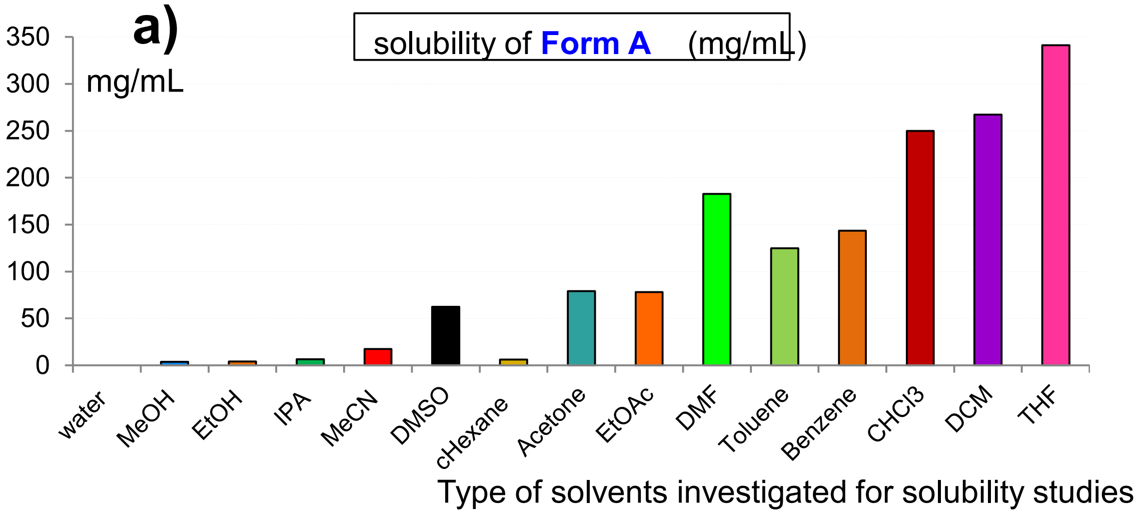

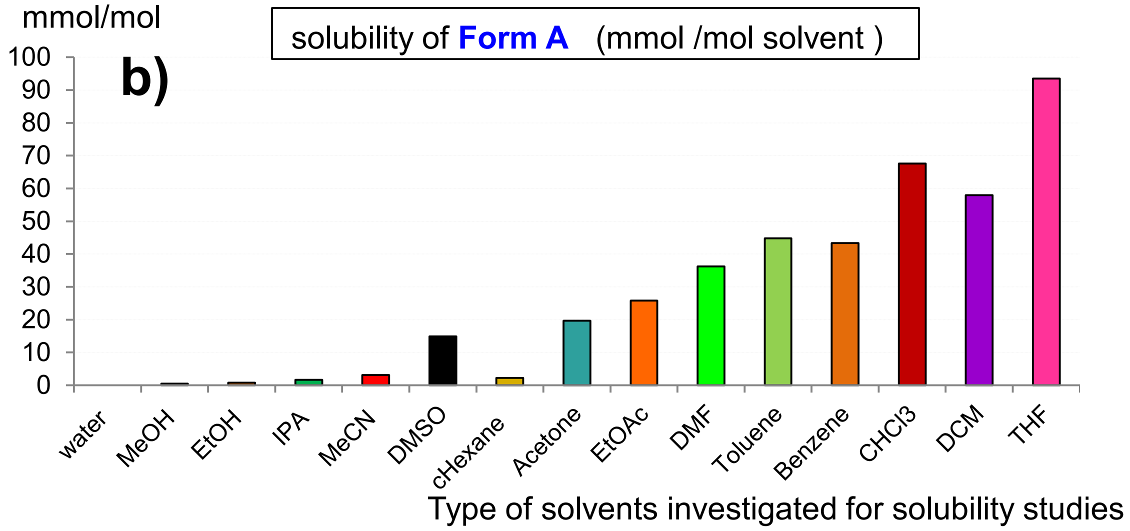

Figure S 80 Comparison of solubility of **Form A** & **Form B** determined in 15 solvents, a) mg /mL and b) mmol /mol solvent

10.10 Summary solubility data for Form A & Form B

Table S 86 Summary table of solubility of **Form A** & **Form** B; expressed as mg/ml & mmol/mol solvent

MW (solvent)	18.02	32.04	46.07	60.10	41.05	78.13	84.16	58.08	88.11	59.07	92.14	78.12	119.30	89.93	72.11
density Solvent g/mL	0.9974	0.7872	0.7848	0.7815	0.7760	1.0958	0.7781	0.7849	0.8945	0.9995	0.8619	0.8690	1.4793	1.3943	0.8837
Solvent investigated	**water**	**MeOH**	**EtOH**	**IPA**	**MeCN**	**DMSO**	**cyclo-Hexane**	**Acetone**	**EtOAc**	**DMF**	**Toluene**	**Benzene**	**$CHCl_3$**	**DCM**	**THF**
Form B (mg/mL)	**0.0**	**3.3**	**3.4**	**5.3**	**19**	**68**	**6.3**	**76**	**75**	**215**	**130**	**141**	**248**	**266**	**334**
Form A (mg/mL)	**0.0**	**3.9**	**4.3**	**6.4**	**18**	**62**	**6.3**	**79**	**78**	**183**	**125**	**144**	**250**	**267**	**341**
Form B mmol /mol solvent	**0.0**	**0.5**	**0.7**	**1.4**	**3.3**	**16**	**2**	**19**	**25**	**43**	**47**	**42**	**67**	**58**	**91**
Form A mmol /mol solvent	**0.0**	**0.5**	**0.8**	**1.7**	**3.1**	**15**	**2**	**20**	**26**	**36**	**45**	**43**	**68**	**58**	**94**

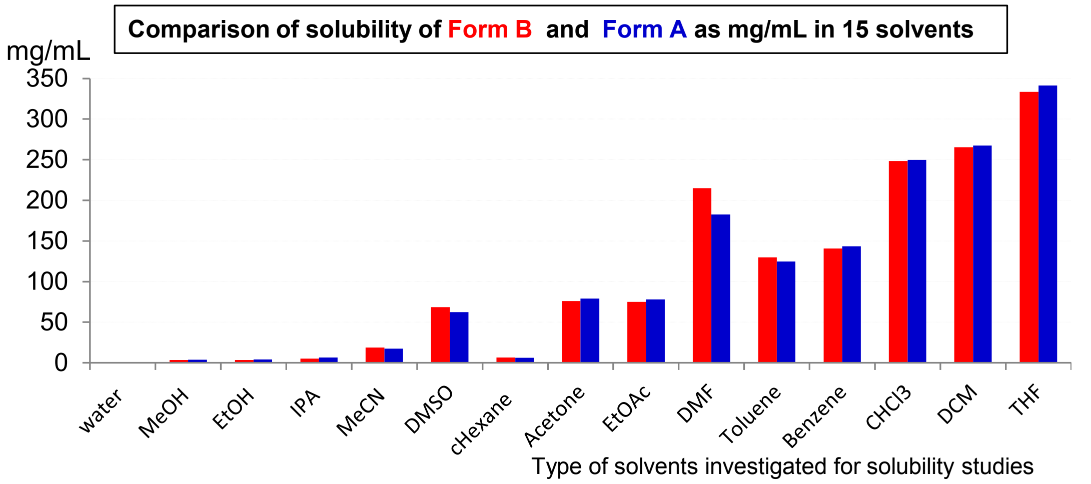

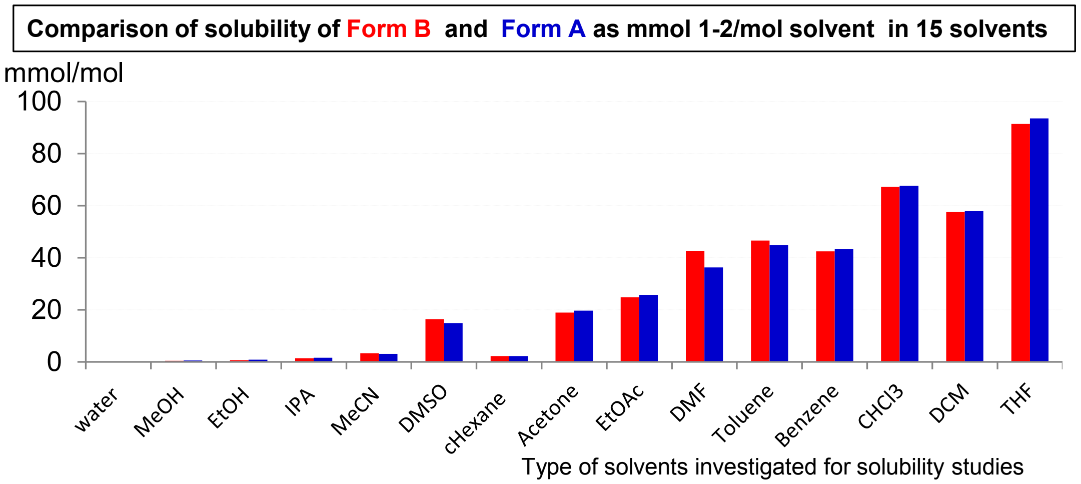

Figure S 81 Comparison of solubility of **Form A** & **Form B** determined in 15 solvents, expressed as: top: as mg/mL; bottom: as mmol/mol solvent

10.11 Summary solubility data for Form A & Form B in comparison with 1-1 & 2-2

Table S 87 Summary table of solubility of **Form A** & Form B; expressed as mg/ml & mmol/mol solvent

MW (solvent)	18.02	32.04	46.07	60.10	41.05	78.13	84.16	58.08	88.11	59.07	92.14	78.12	119.30	89.93	72.11
density Solvent g/mL	0.9974	0.7872	0.7848	0.7815	0.7760	1.0958	0.7781	0.7849	0.8945	0.9995	0.8619	0.8690	1.4793	1.3943	0.8837
Solvent investigated	**water**	**MeOH**	**EtOH**	**IPA**	**MeCN**	**DMSO**	**cyclo-Hexane**	**Acetone**	**EtOAc**	**DMF**	**Toluene**	**Benzene**	**CHCl3**	**DCM**	**THF**
Form B (mg/mL)	**0.0**	**3.3**	**3.4**	**5.3**	**19**	**68**	**6.3**	**76**	**75**	**215**	**130**	**141**	**248**	**266**	**334**
Form A (mg/mL)	**0.0**	**3.9**	**4.3**	**6.4**	**18**	**62**	**6.3**	**79**	**78**	**183**	**125**	**144**	**250**	**267**	**341**
1-1 (mg/mL)	**0.1**	**2.3**	**2.6**	**1.5**	**3.4**	**7.9**	**1.0**	**7.4**	**4.5**	**16**	**4.4**	**5.6**	**17**	**28**	**29**
2-2 (mg/mL)	**0.0**	**14**	**28**	**19**	**30**	**128**	**145**	**266**	**352**	**302**	**516**	**566**	**609**	**659**	**697**
Form B mmol /mol solvent	**0.0**	**0.5**	**0.7**	**1.4**	**3.3**	**16**	**2**	**19**	**25**	**43**	**47**	**42**	**67**	**58**	**91**
Form A mmol /mol solvent	**0.0**	**0.5**	**0.8**	**1.7**	**3.1**	**15**	**2**	**20**	**26**	**36**	**45**	**43**	**68**	**58**	**94**
1-1 mmol /mol solvent	**0.0**	**0.3**	**0.5**	**0.4**	**0.6**	**1.8**	**0.4**	**1.8**	**1.4**	**3.1**	**1.5**	**1.6**	**4.5**	**5.8**	**7.8**
2-2 mmol /mol solvent	**0.0**	**2.0**	**5.6**	**5.0**	**5.4**	**32**	**55**	**69**	**121**	**62**	**192**	**177**	**171**	**148**	**198**

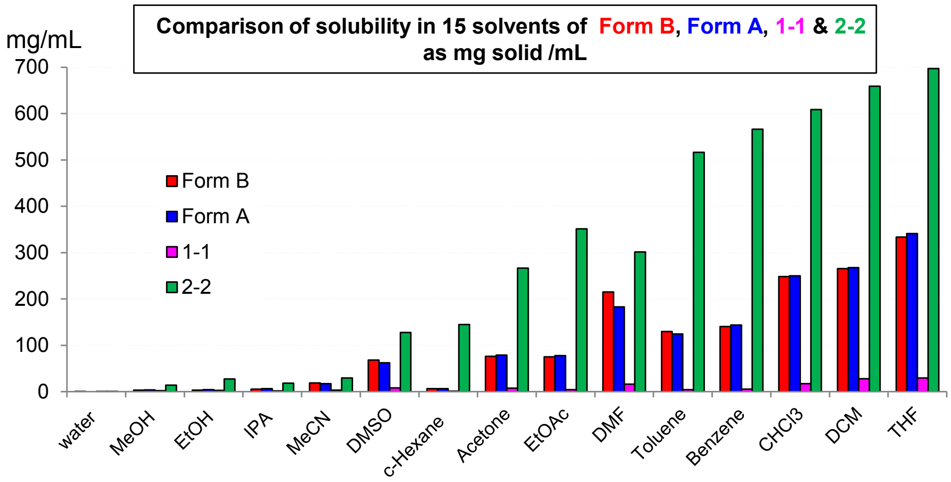

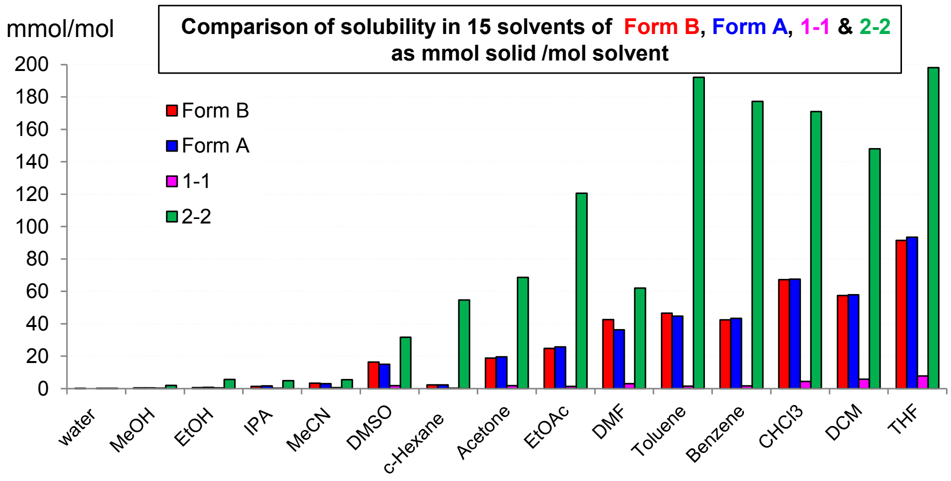

Figure S 82 Comparison of solubility of **1-1**, **2-2**, F**orm A** & **Form B** determined in 15 solvents, expressed as: top: as mg/mL; bottom: as mmol/mol solvent

10.12 Summary solubility data for individual graphs for Form A & Form B in comparison with 1-1 & 2-2 expressed as mg/mL

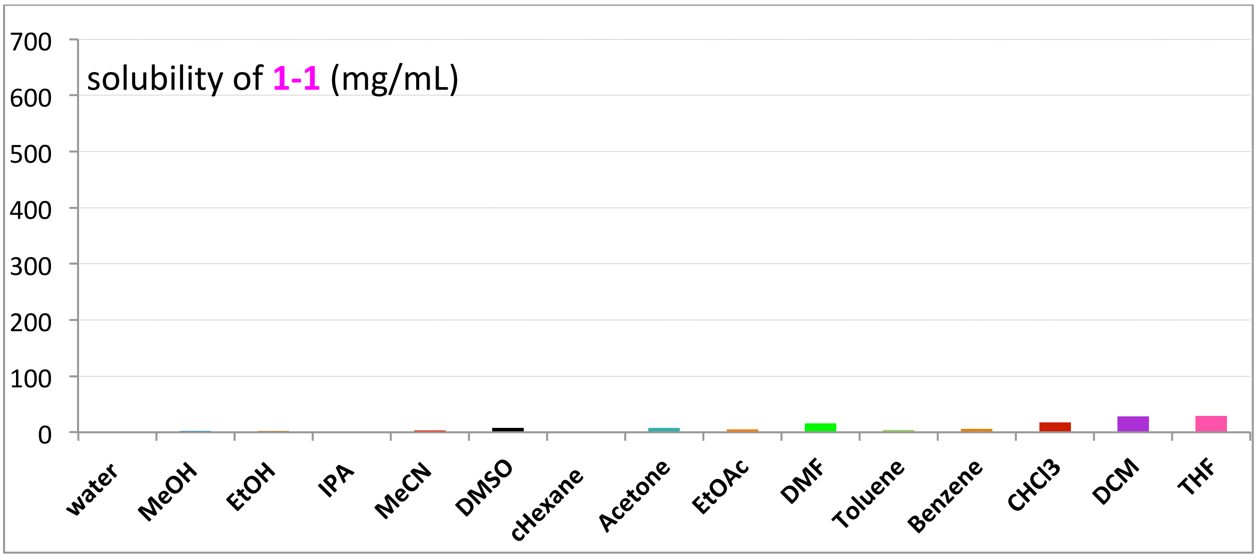

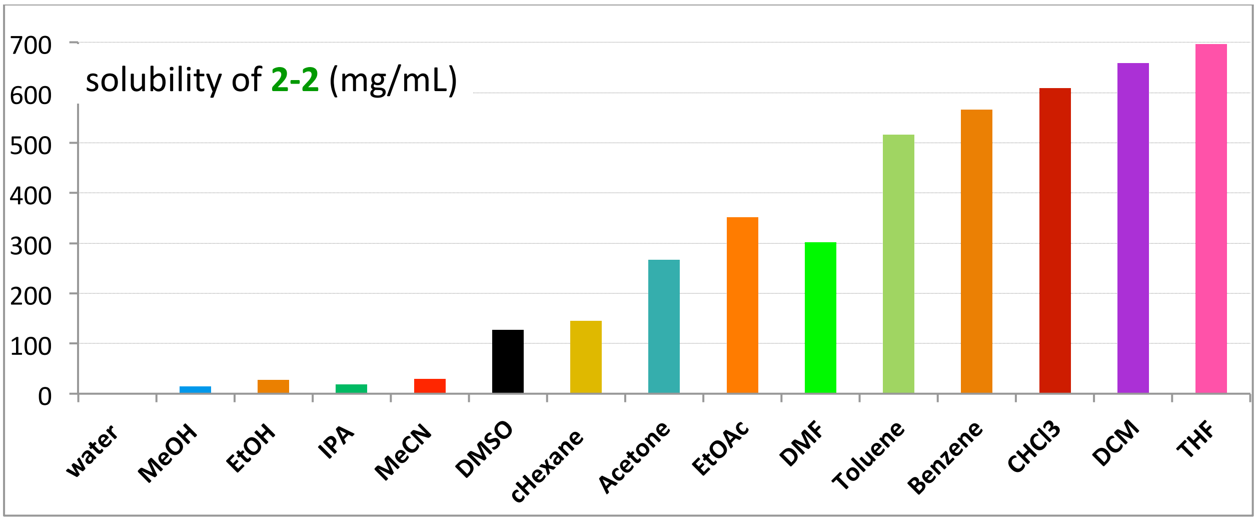

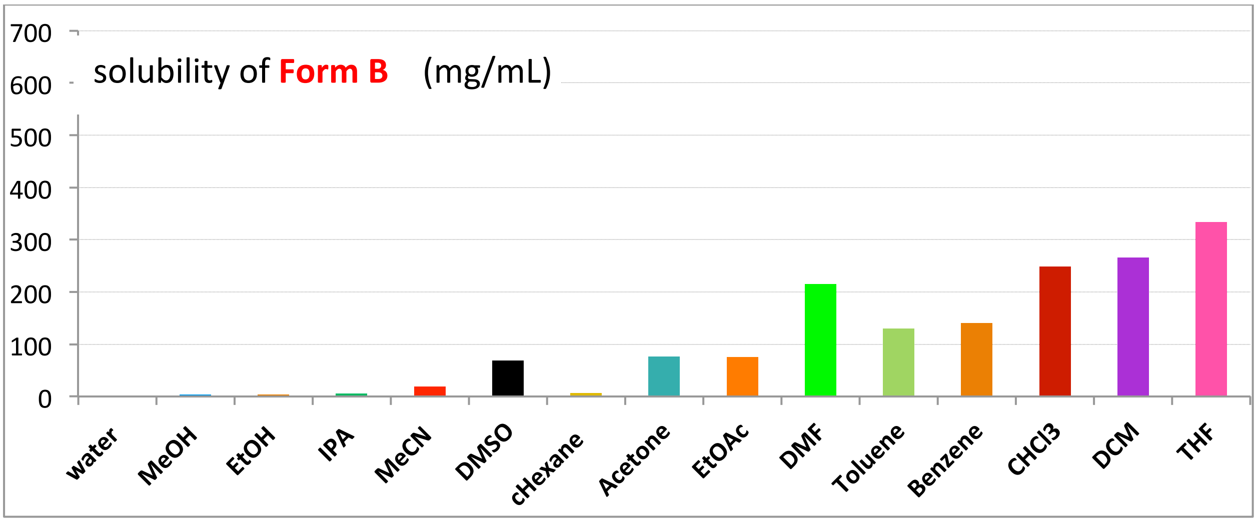

Figure S 83: Graphical representation of the solubility of 1-1, 2-2 and Form B in 15 solvents expressed as mg/mL. **As Form A transformed rapidly to Form B; the solubility of Form A is taken as that of Form B**

10.13 Summary solubility data for individual graphs for Form A & Form B in comparison with 1-1 & 2-2 expressed as mmol/mol solvent

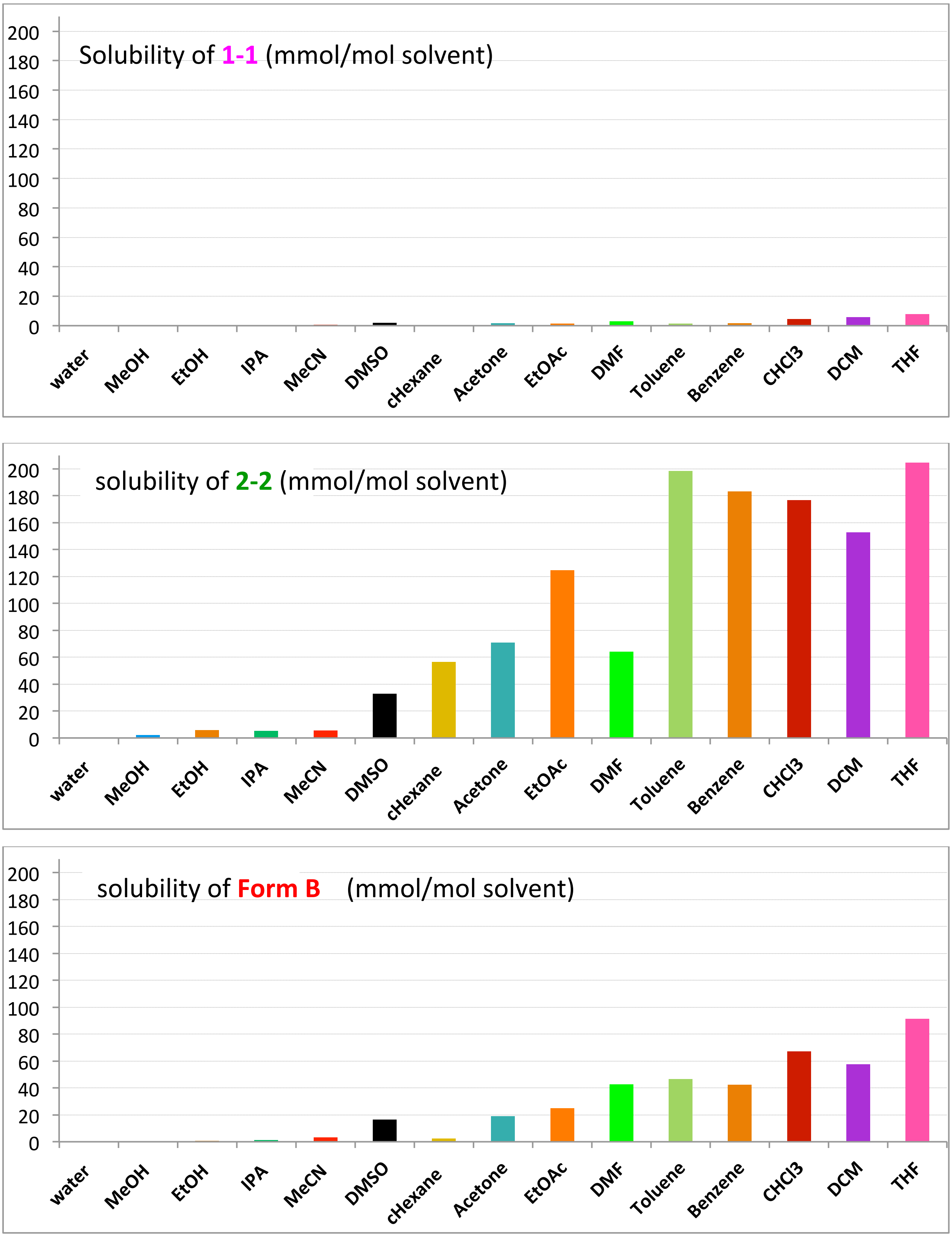

Figure S 84 Graphical representation of the solubility of 1-1, 2-2 and Form B in 15 solvents expressed as mmol/mol. **As Form A transformed rapidly to Form B; the solubility of Form A is taken as that of Form B**

11 Investigating which of the 2 polymorphs of system ii (Form A and Form B) is the thermodynamic product under ambient conditions (slurry experiment)

A good way to investigate which of the polymorphs of system ii (**Form A** and **Form B**) is the thermodynamic product under ambient conditions (ambient temperature and pressure) a slurry solution is prepared in any solvent with an equimolar mixture of the two polymorphs. This slurry is allowed to stir until equilibrium is achieved.

In this case we selected MeCN as the solvent. However, any other solvent would have done just as well although the kinetic of reaching equilibrium may differ.

We demonstrate here that **Form B** is the thermodynamic product under ambient conditions as it is formed in quantitative yield at equilibrium while **Form A** is the metastable product as it is consumed while being transformed to the more stable polymorph **Form B**.

For **Form B** we used crystals of **Form B** (See Section 4.2.3) which were manually ground with mortar and pestle to increase the surface area exposed to the solvents. **Form A** was obtained from ball mill neat grinding crystals of **Form B** to equilibrium (See Section 4.2.4).

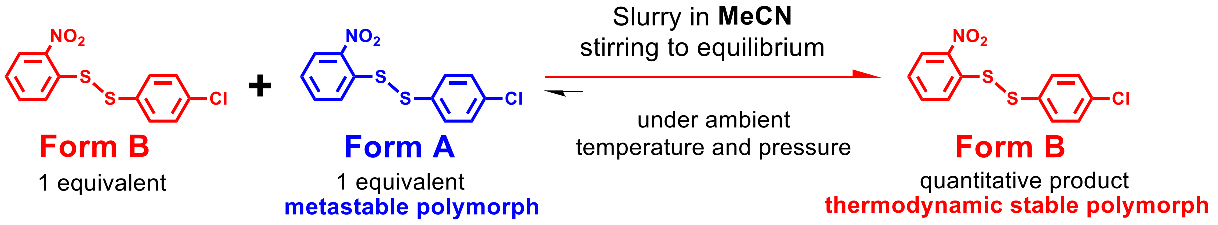

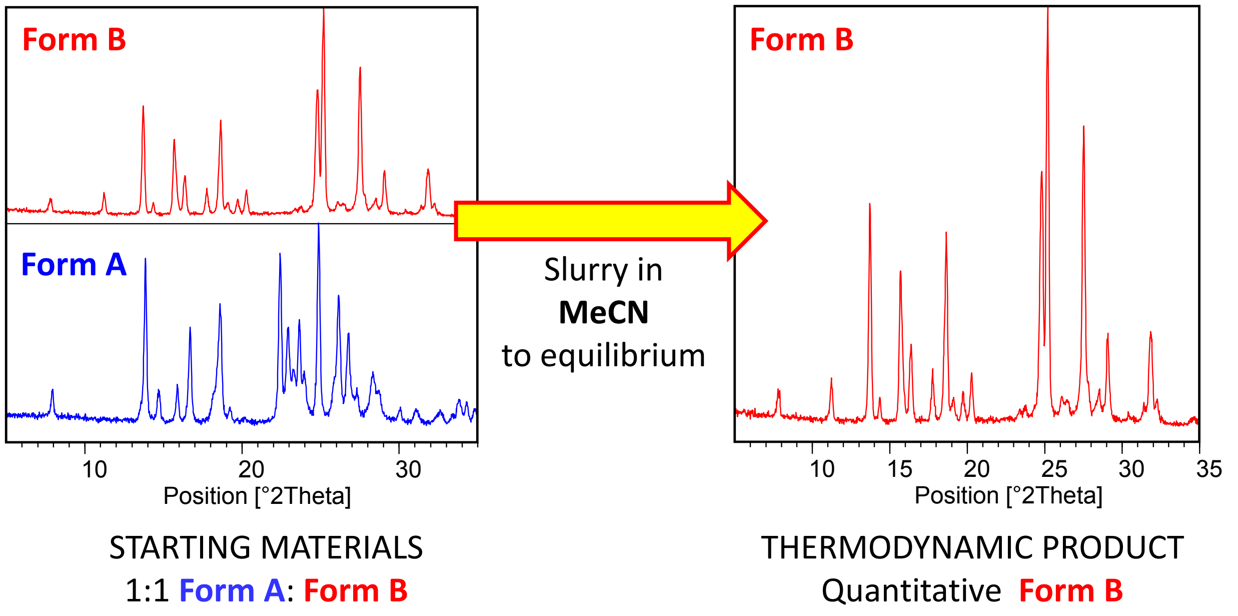

Figure S 85 determining which polymorph is the thermodynamic stable product under slurry conditions (solvent used is MeCN)

12 Determination of polymorph stability of Form A and Form B under slurry conditions performed in 15 solvents

12.1 Determination of polymorph stability of Form B under slurry conditions

12.1.1 Slurry experiment: Form B in MeCN

Table S 88: Composition of **Form B** slurry in MeCN after stirring for different periods of time

Determining the thermodynamic stable polymorph at ambient temperature and pressure

Slurry of **Form B** in Acetonitrile

Slurry stirring for	1-1 %M	1-1 e.s.d. (mol%)	2-2 %M	2-2 e.s.d. (mol%)	Form B %M	Form B e.s.d. (mol%)	Form A %M	Form A e.s.d. (mol%)	total polymorp [A]+[B]	R = [B] / [A]+[B]	Stable polymorph under ambient conditions
Starting material	Consistent with quantitative **Form B**: no Rietveld refinement performed.										
3 hours	2.6	2.2	2.2	1.8	**94**	3.3	**1.2**	1.9	95.2	**99%**	
3 weeks	1.9	0.5	1.7	0.4	**95**	1	**1.4**	0.8	96.4	**99%**	**Form B**

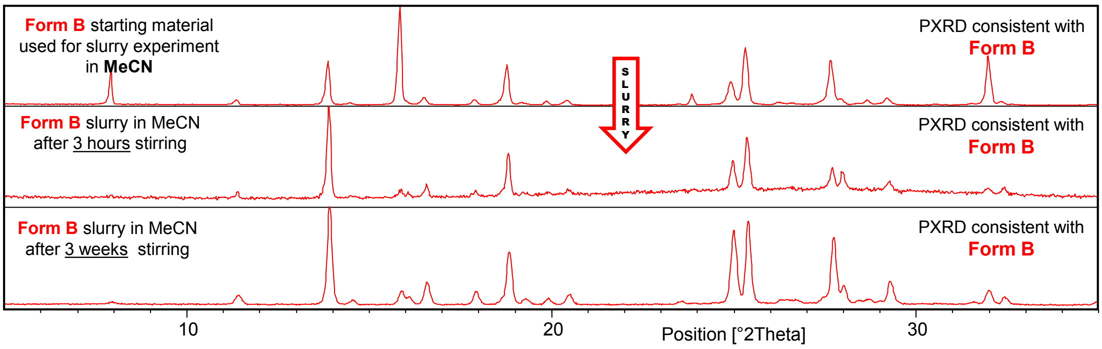

PXRD scan 23 PXRD scan of the residue of **Form B**, continuously stirring in a slurry in MeCN for 3 h and 3 weeks

12.1.2 Slurry experiment of Form B in Acetone

Table S 89: Composition of **Form B** slurry in Acetone after stirring for different periods of time

Determining the thermodynamic stable polymorph at ambient temperature and pressure

Slurry of **Form B** in Acetone

Slurry stirring for	1-1 %M	1-1 e.s.d. (mol%)	2-2 %M	2-2 e.s.d. (mol%)	Form B %M	Form B e.s.d. (mol%)	Form A %M	Form A e.s.d. (mol%)	total polymorp [A]+[B]	R = [B] / [A]+[B]	Stable polymorph under ambient conditions
Starting material	Consistent with quantitative **Form B**: no Rietveld refinement performed.										
3 hours	8	4.9	6.6	4.1	**81.2**	8.5	**4.2**	6.8	85.4	**95%**	
3 weeks	1.3	0.5	1.2	0.5	**97.1**	0.7	**0.5**	0.3	97.6	**99%**	**Form B**

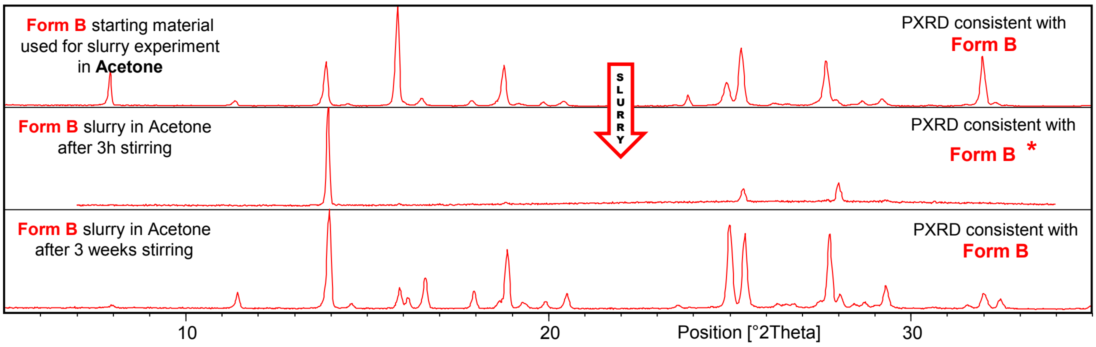

* **Form B** displays strong preferred orientation because of the crystallinity of the PXRD sample as it was not ground to powder.

PXRD scan 24 PXRD scan of the residue of **Form B**, continuously stirring in slurry in Acetone for 3 h and 3 weeks

12.1.3 Slurry experiment of Form B in Tetrahydrofuran

Table S 90: Composition of **Form B** slurry in Tetrahydrofuran after stirring for different periods of time

Determining the thermodynamic stable polymorph at ambient temperature and pressure											
Slurry of **Form B** in Tetrahydrofuran											
	1-1		2-2		Form B		Form A		total polymorp	R =	Stable polymorph under ambient conditions
Slurry stirring for	%M	e.s.d. (mol%)	%M	e.s.d. (mol%)	%M	e.s.d. (mol%)	%M	e.s.d. (mol%)	[A]+[B]	[B] / [A]+[B]	
Starting material	Consistent with quantitative **Form B**: no Rietveld refinement performed.										
3 hours	0.9	0.5	0.8	0.4	**97.8**	0.7	**0.5**	0.3	98.3	**99%**	
3 weeks	1.9	0.5	1.7	0.4	**92.8**	1	**3.6**	0.8	98.3	**99%**	**Form B**

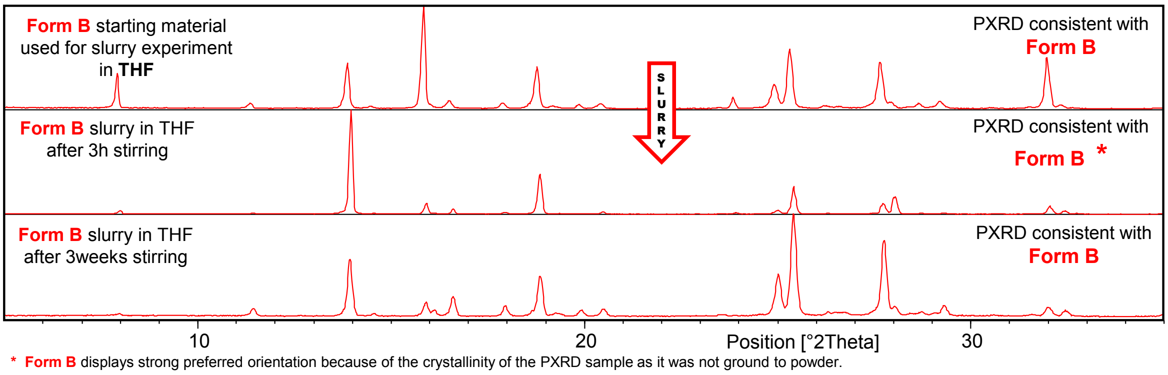

* **Form B** displays strong preferred orientation because of the crystallinity of the PXRD sample as it was not ground to powder.

PXRD scan 25 PXRD scan of the residue of **Form B**, continuously stirring in a slurry in THF for 3 h and 3 weeks

12.1.4 Slurry experiment of Form B in Ethyl Acetate

Table S 91 Composition of **Form B** slurry in EtOAc after stirring for different periods of time

Determining the thermodynamic stable polymorph at ambient temperature and pressure											
Slurry of **Form B** in Ethyl Acetate											
	1-1		2-2		Form B		Form A		total polymorp	R =	Stable polymorph under ambient conditions
Slurry stirring for	%M	e.s.d. (mol%)	%M	e.s.d. (mol%)	%M	e.s.d. (mol%)	%M	e.s.d. (mol%)	[A]+[B]	[B] / [A]+[B]	
Starting material	Consistent with quantitative **Form B**: no Rietveld refinement performed.										
3 hours	4.5	4.8	3.7	4	**83.5**	7.9	**8.3**	5.9	91.8	**91%**	
3 weeks	1.4	0.6	1.3	0.5	**95**	1.1	**2.2**	0.8	91.8	**91%**	**Form B**

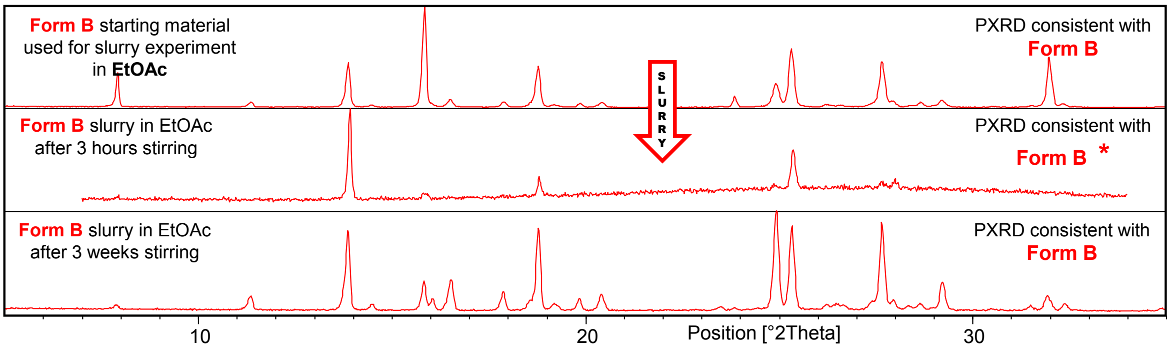

* **Form B** displays strong preferred orientation because of the crystallinity of the PXRD sample as it was not ground to powder.

PXRD scan 26 PXRD scan of the residue of **Form B**, continuously stirring in a slurry in EtOAc for 3 h and 3 weeks

12.1.5 Slurry experiment of Form B in Chloroform

Table S 92 Composition of **Form B** slurry in $CHCl_3$ after stirring for different periods of time

Determining the thermodynamic stable polymorph at ambient temperature and pressure											
Slurry of **Form B** in Chloroform											
	1-1		2-2		Form B		Form A		total polymorp	R =	Stable polymorph
Slurry stirring for	%M	e.s.d. (mol%)	%M	e.s.d. (mol%)	%M	e.s.d. (mol%)	%M	e.s.d. (mol%)	[A]+[B]	[B] / ([A]+[B])	under ambient conditions
Starting material	Consistent with quantitative **Form B**: no Rietveld refinement performed.										
3 hours	4.2	1.2	3.4	0.9	**83.3**	1.9	**9.2**	1.5	92.5	**90%**	
3 weeks	1.1	0.5	1	0.5	**95.5**	1	**2.4**	0.7	92.5	**90%**	**Form B**

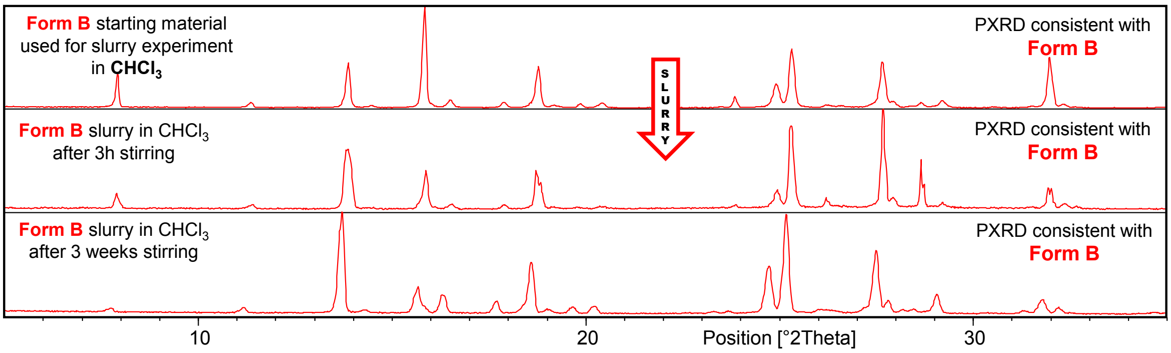

PXRD scan 27 PXRD scan of the residue of **Form B**, continuously stirring in a slurry in $CHCl_3$ for 3 h and 3 weeks

12.1.6 Slurry experiment of Form B in Dichloromethane

Table S 93 Composition of **Form B** slurry in DCM after stirring for different periods of time

Determining the thermodynamic stable polymorph at ambient temperature and pressure											
Slurry of **Form B** in Dichloromethane											
	1-1		2-2		Form B		Form A		total polymorp	R =	Stable polymorph
Slurry stirring for	%M	e.s.d. (mol%)	%M	e.s.d. (mol%)	%M	e.s.d. (mol%)	%M	e.s.d. (mol%)	[A]+[B]	[B] / ([A]+[B])	under ambient conditions
Starting material	Consistent with quantitative **Form B**: no Rietveld refinement performed.										
3 hours	1.8	0.6	1.4	0.5	**94.4**	1	**2.5**	0.7	96.9	**97%**	
3 weeks	2.2	0.4	2	0.4	**92.4**	0.8	**3.4**	0.6	96.9	**97%**	**Form B**

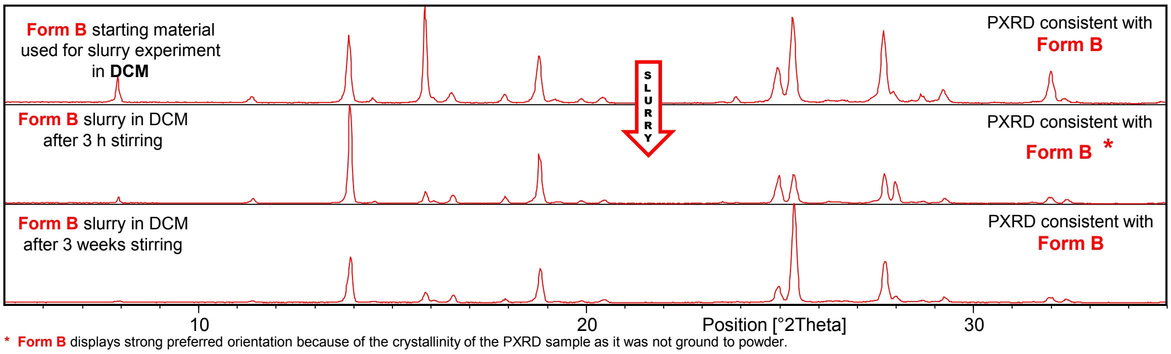

* **Form B** displays strong preferred orientation because of the crystallinity of the PXRD sample as it was not ground to powder.

PXRD scan 28 PXRD scan of the residue of **Form B**, continuously stirring in a slurry in DCM for 3 h and 3 weeks

12.1.7 Slurry experiment of Form B in Dimethylformamide

Table S 94 Composition of **Form B** slurry in DMF after stirring for different periods of time

Determining the thermodynamic stable polymorph at ambient temperature and pressure

Slurry of **Form B** in Dimethylformamide

	1-1		2-2		Form B*		Form A		total polymorp	R =	Stable polymorph under ambient conditions
Slurry stirring for	%M	e.s.d. (mol%)	%M	e.s.d. (mol%)	%M	e.s.d. (mol%)	%M	e.s.d. (mol%)	[A]+[B]	[B] / [A]+[B]	
Starting material	Consistent with quantitative **Form B**: no Rietveld refinement performed.										
3 hours	2.4	0.9	2	0.8	**94.4**	1.3	**1.2**	0.5	95.6	**99%**	
3 weeks	2.3	0.8	2.2	0.7	**95.2**	1.2	**0.4**	0.5	95.6	**99%**	**Form B**

* **Form B** decomposes with time in DMF.

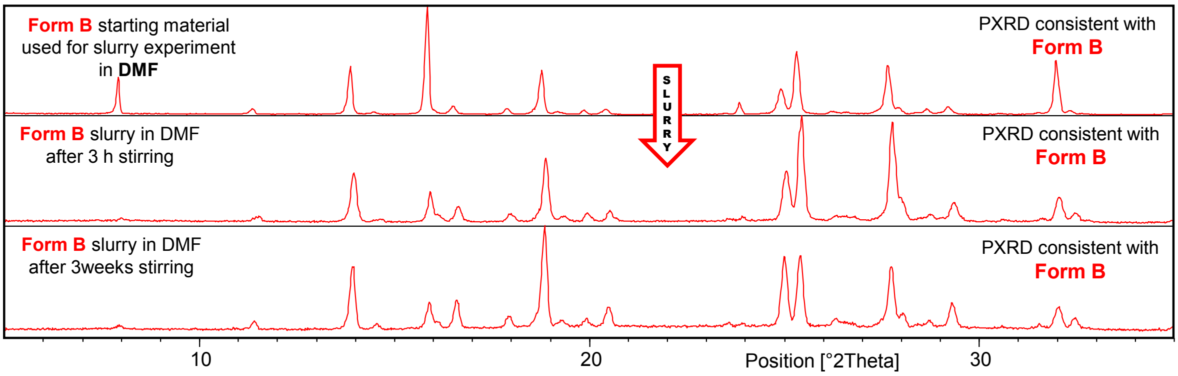

PXRD scan 29 PXRD scan of the residue of **Form B**, continuously stirring in a slurry in DMF for 3 h and 3 weeks

12.1.8 Slurry experiment of Form B in Dimethylsulphoxide

Table S 95 Composition of **Form B** slurry in DMSO after stirring for different periods of time

Determining the thermodynamic stable polymorph at ambient temperature and pressure

Slurry of **Form B** in Dimethylsulphoxide

	1-1		2-2		Form B*		Form A		total polymorp	R =	Stable polymorph under ambient conditions
Slurry stirring for	%M	e.s.d. (mol%)	%M	e.s.d. (mol%)	%M	e.s.d. (mol%)	%M	e.s.d. (mol%)	[A]+[B]	[B] / [A]+[B]	
Starting material	Consistent with quantitative **Form B**: no Rietveld refinement performed.										
3 hours	44.1	17.5	35	15.1	**4.3**	25.7	**16.6**	11.5	20.9	**21%**	
3 weeks	44.5	2	36	1.8	**7.4**	2.2	**12**	1.9	20.9	**21%**	**Form B**

* **Form B** decomposes with time in DMSO.

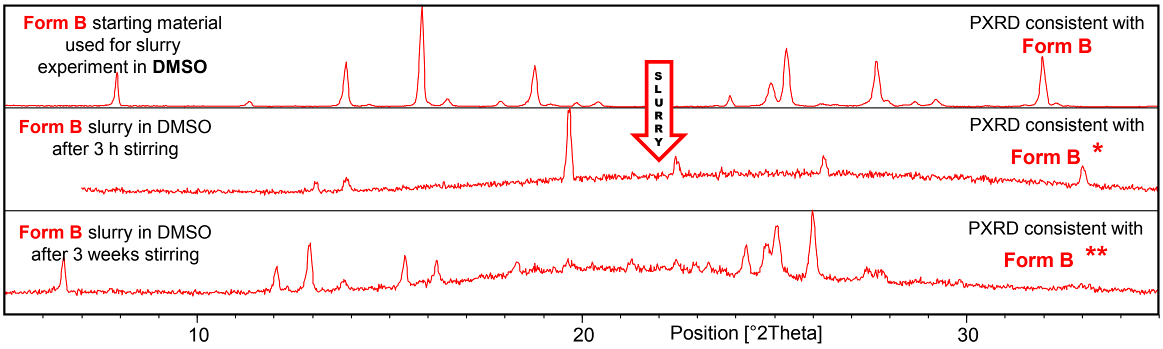

* **Form B** displays strong preferred orientation because of the crystallinity of the PXRD sample as it was not ground to powder.
** **Form B** decomposes with time in DMSO.

PXRD scan 30 PXRD scan of the residue of **Form B**, continuously stirring in a slurry in DMSO for 3 h and 3 weeks

12.1.9 Slurry experiment of Form B in Cyclohexane

Table S 96 Composition of **Form B** slurry in cyclohexane after stirring for different periods of time

Determining the thermodynamic stable polymorph at ambient temperature and pressure

Slurry of **Form B** in Cyclohexane

Slurry stirring for	1-1 %M	1-1 e.s.d. (mol%)	2-2 %M	2-2 e.s.d. (mol%)	Form B %M	Form B e.s.d. (mol%)	Form A %M	Form A e.s.d. (mol%)	total polymorp [A]+[B]	R = [B] / [A]+[B]	Stable polymorph under ambient conditions
Starting material	Consistent with quantitative **Form B**: no Rietveld refinement performed.										
3 hours	2.5	0.9	2	0.7	**93.1**	1.4	**2.4**	0.9	95.5	**97%**	
3 weeks	1.7	0.6	1.6	0.6	**92.7**	1	**3.9**	0.6	95.5	**97%**	**Form B**

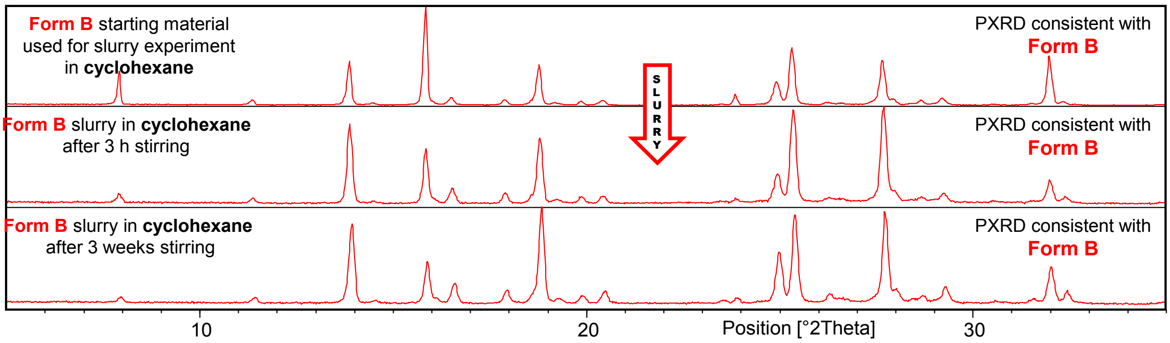

PXRD scan 31 PXRD scan of the residue of **Form B**, continuously stirring in a slurry in cyclohexane for 3 h and 3 weeks

12.1.10 Slurry experiment of Form B in Benzene

Table S 97 Composition of **Form B** slurry in benzene after stirring for different periods of time

Determining the thermodynamic stable polymorph at ambient temperature and pressure

Slurry of **Form B** in Benzene

Slurry stirring for	1-1 %M	1-1 e.s.d. (mol%)	2-2 %M	2-2 e.s.d. (mol%)	Form B %M	Form B e.s.d. (mol%)	Form A %M	Form A e.s.d. (mol%)	total polymorp [A]+[B]	R = [B] / [A]+[B]	Stable polymorph under ambient conditions
Starting material	Consistent with quantitative **Form B**: no Rietveld refinement performed.										
3 hours	1.3	0.7	1.1	0.5	**96.6**	1.1	**1.1**	0.7	97.7	**99%**	
3 weeks	1.5	0.4	1.4	0.4	**93.5**	0.9	**3.6**	0.7	97.7	**99%**	**Form B**

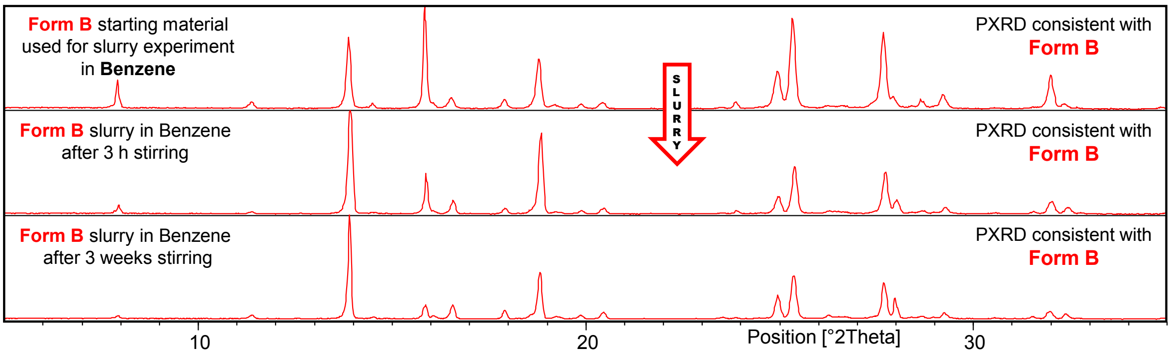

PXRD scan 32 PXRD scan of the residue of **Form B**, continuously stirring in a slurry in Benzene for 3 h and 3 weeks

12.1.11 Slurry experiment of Form B in Toluene

Table S 98 Composition of **Form B** slurry in Toluene after stirring for different periods of time

Determining the thermodynamic stable polymorph at ambient temperature and pressure

Slurry of **Form B** in Toluene

Slurry stirring for	1-1 %M	1-1 e.s.d. (mol%)	2-2 %M	2-2 e.s.d. (mol%)	Form B %M	Form B e.s.d. (mol%)	Form A %M	Form A e.s.d. (mol%)	total polymorp [A]+[B]	R = [B] / [A]+[B]	Stable polymorph under ambient conditions
Starting material	Consistent with quantitative **Form B**: no Rietveld refinement performed.										
3 hours	2.7	1	2.2	0.9	**87.4**	1.9	**7.7**	1.5	95.1	**92%**	
3 weeks	2.1	0.7	1.9	0.7	**88.4**	1.2	**7.5**	0.7	95.1	**92%**	**Form B**

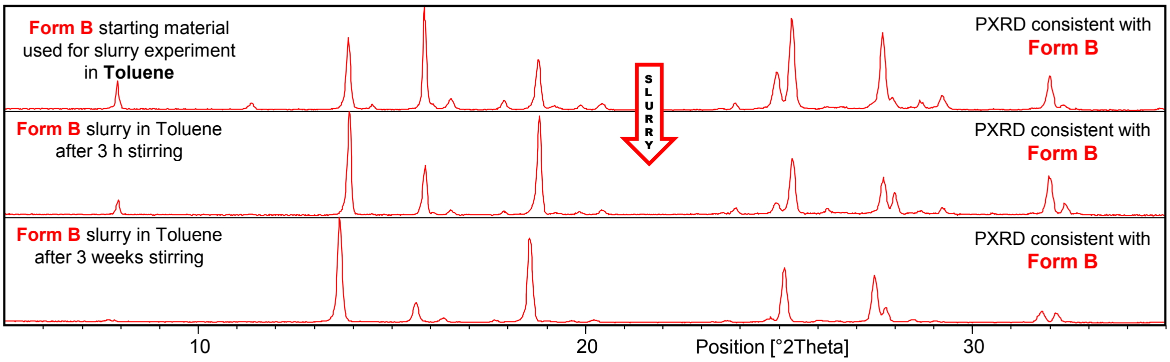

PXRD scan 33 PXRD scan of the residue of **Form B**, continuously stirring in a slurry in Toluene for 3 h and 3 weeks

12.1.12 Slurry experiment of Form B in Methanol

Table S 99 Composition of **Form B** slurry in MeOH after stirring for different periods of time

Determining the thermodynamic stable polymorph at ambient temperature and pressure

Slurry of **Form B** in Methanol

Slurry stirring for	1-1 %M	1-1 e.s.d. (mol%)	2-2 %M	2-2 e.s.d. (mol%)	Form B %M	Form B e.s.d. (mol%)	Form A %M	Form A e.s.d. (mol%)	total polymorp [A]+[B]	R = [B] / [A]+[B]	Stable polymorph under ambient conditions
Starting material	Consistent with quantitative **Form B**: no Rietveld refinement performed.										
3 hours	1.4	0.6	1.2	0.5	**96**	0.9	**1.5**	0.4	97.5	**98%**	
3 weeks	1.7	0.5	1.6	0.5	**94.9**	1.1	**1.8**	0.9	97.5	**98%**	**Form B**

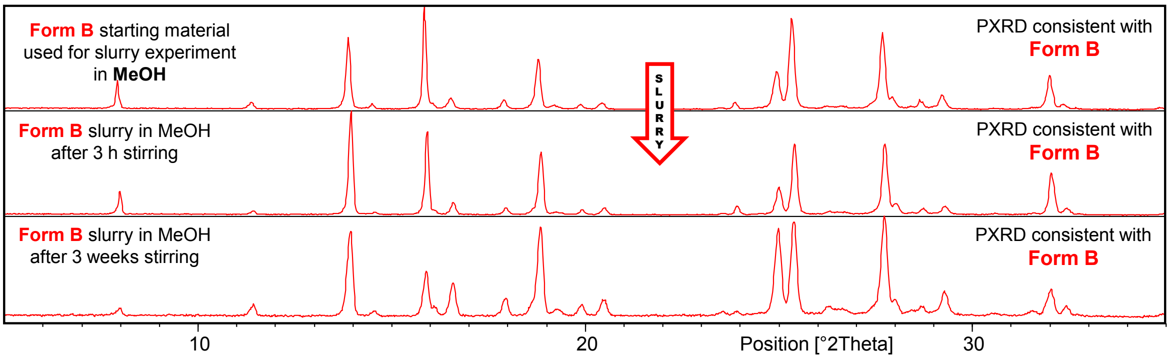

PXRD scan 34 PXRD scan of the residue of **Form B**, continuously stirring in a slurry in MeOH for 3 h and 3 weeks

12.1.13 Slurry experiment of Form B in Ethanol

Table S 100 Composition of **Form B** slurry in EtOH after stirring for different periods of time

<u>Determining the thermodynamic stable polymorph at ambient temperature and pressure</u>

Slurry of **Form B** in Ethanol

	1-1		2-2		Form B		Form A		total polymorp	R =	Stable polymorph
Slurry stirring for	%M	e.s.d. (mol%)	%M	e.s.d. (mol%)	%M	e.s.d. (mol%)	%M	e.s.d. (mol%)	[A]+[B]	[B] / [A]+[B]	under ambient conditions
Starting material	Consistent with quantitative **Form B**: no Rietveld refinement performed.										
3 hours	1.9	0.7	1.5	0.6	**95.4**	1	**1.2**	0.5	96.6	**99%**	
3 weeks	1.8	0.7	1.7	0.6	**96.5**	1	**0**	0.4	96.6	**99%**	**Form B**

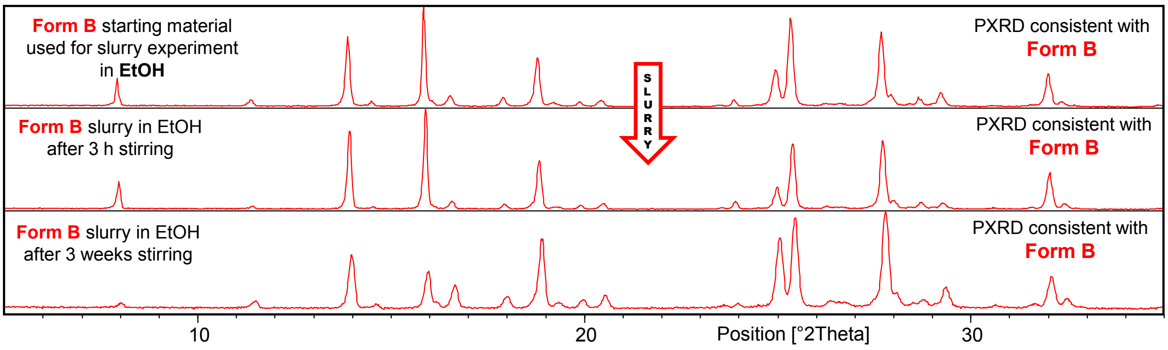

PXRD scan 35 PXRD scan of the residue of **Form B**, continuously stirring in a slurry in EtOH for 3 h and 3 weeks

12.1.14 Slurry experiment of Form B in Isopropanol

Table S 101 Composition of **Form B** slurry in IPA after stirring for different periods of time

<u>Determining the thermodynamic stable polymorph at ambient temperature and pressure</u>

Slurry of **Form B** in Isopropanol

	1-1		2-2		Form B		Form A		total polymorp	R =	Stable polymorph
Slurry stirring for	%M	e.s.d. (mol%)	%M	e.s.d. (mol%)	%M	e.s.d. (mol%)	%M	e.s.d. (mol%)	[A]+[B]	[B] / [A]+[B]	under ambient conditions
Starting material	Consistent with quantitative **Form B**: no Rietveld refinement performed.										
3 hours	4	1	3.3	0.8	**86.1**	1.8	**6.6**	1.4	92.7	**93%**	
3 weeks	7.4	3.7	6.5	3.3	**80.4**	6.1	**5.7**	4	92.7	**93%**	**Form B**

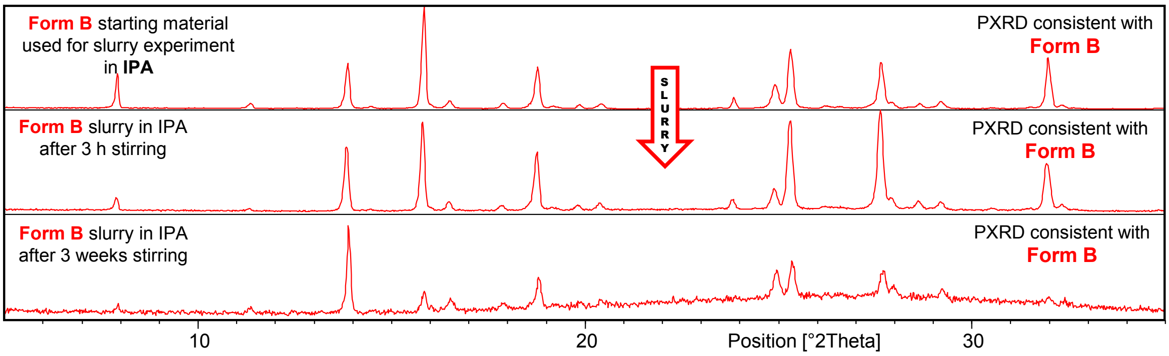

PXRD scan 36 PXRD scan of the residue of **Form B**, continuously stirring in a slurry in IPA for 3 h and 3 weeks

12.1.15 Slurry experiment of Form B in Water

Table S 102 Composition of **Form B** slurry in Water after stirring for different periods of time

Determining the thermodynamic stable polymorph at ambient temperature and pressure											
Slurry of **Form B** in Water											
	1-1		2-2		Form B		Form A		total polymorph	R =	Stable polymorph
Slurry stirring for	%M	e.s.d. (mol%)	%M	e.s.d. (mol%)	%M	e.s.d. (mol%)	%M	e.s.d. (mol%)	[A]+[B]	[B] / [A]+[B]	under ambient conditions
Starting material	Consistent with quantitative **Form B**: no Rietveld refinement performed.										
1month	Consistent with quantitative **Form B**: no Rietveld refinement performed.										**Form B**

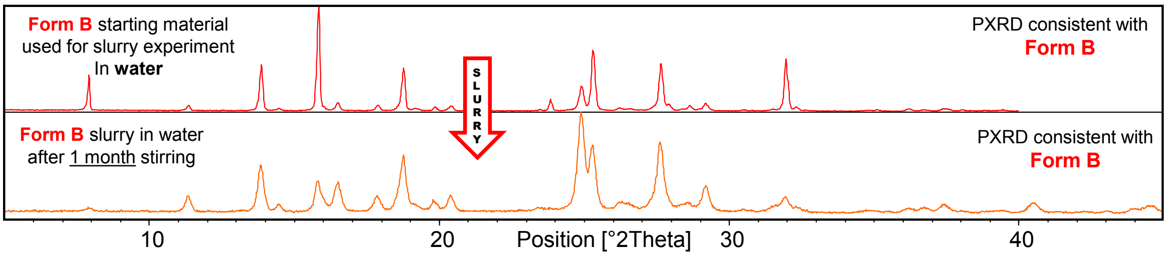

PXRD scan 37 PXRD scan of the residue of **Form B**, continuously stirring in a slurry in water for 1 month

12.1.16 Discussion and conclusions on slurry experiments of Form B in 15 solvents

Form B is the thermodynamic stable polymorph in any solvent and after 3 weeks there is no polymorph transformation. **Form B** is very unstable in DMF and DMSO and after 3 hours (1st point measured) the compound is quite degraded.

12.2 Determination of polymorph stability of Form A under slurry conditions

12.2.1 Slurry experiment Form A in MeCN

Table S 103: Composition of **Form A** slurry in MeCN after stirring for different periods of time

Determining the thermodynamic stable polymorph at ambient temperature and pressure											
Slurry of **Form A** in Acetonitrile											
	**1-1**		**2-2**		**Form B**		**Form A**		total polymorp	**R =**	Stable polymorph
Slurry stirring for	**%M**	e.s.d. (mol%)	**%M**	e.s.d. (mol%)	**%M**	e.s.d. (mol%)	**%M**	e.s.d. (mol%)	[A]+[B]	**[B]** / **[A]+[B]**	under ambient conditions
Starting material											
3 hours	0.9	0.4	0.7	0.3	**97.5**	0.5	**0.9**	0.2	98.4	**99%**	**Form B**

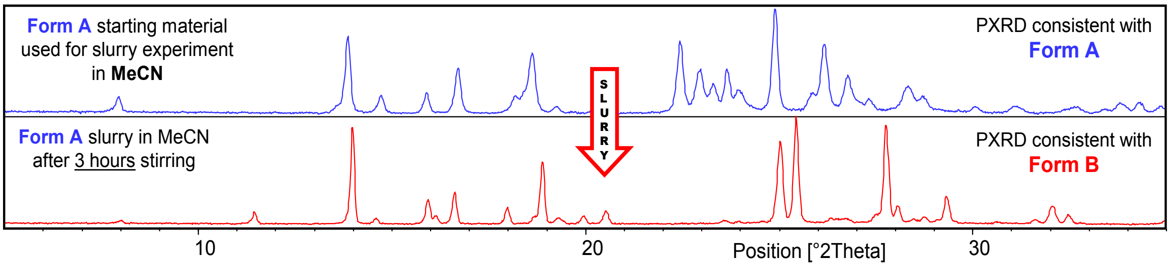

PXRD scan 38 PXRD scan of the residue of **Form A**, continuously stirring in a slurry in MeCN for 3 h

12.2.2 Slurry experiment of Form A in Acetone

Table S 104: Composition of **Form A** slurry in Acetone after stirring for different periods of time

Determining the thermodynamic stable polymorph at ambient temperature and pressure											
Slurry of **Form A** in Acetone											
	**1-1**		**2-2**		**Form B**		**Form A**		total polymorp	**R =**	Stable polymorph
Slurry stirring for	**%M**	e.s.d. (mol%)	**%M**	e.s.d. (mol%)	**%M**	e.s.d. (mol%)	**%M**	e.s.d. (mol%)	[A]+[B]	**[B]** / **[A]+[B]**	under ambient conditions
Starting material	Consistent with quantitative **Form A**: no Rietveld refinement performed.										
1 hour	3.8	1.9	3.1	1.6	**90.5**	3.3	**2.5**	2.5	93.0	**97%**	
3 hours	1.5	0.3	1.2	0.3	**96.6**	0.5	**0.8**	0.2	97.4	**99%**	**Form B**

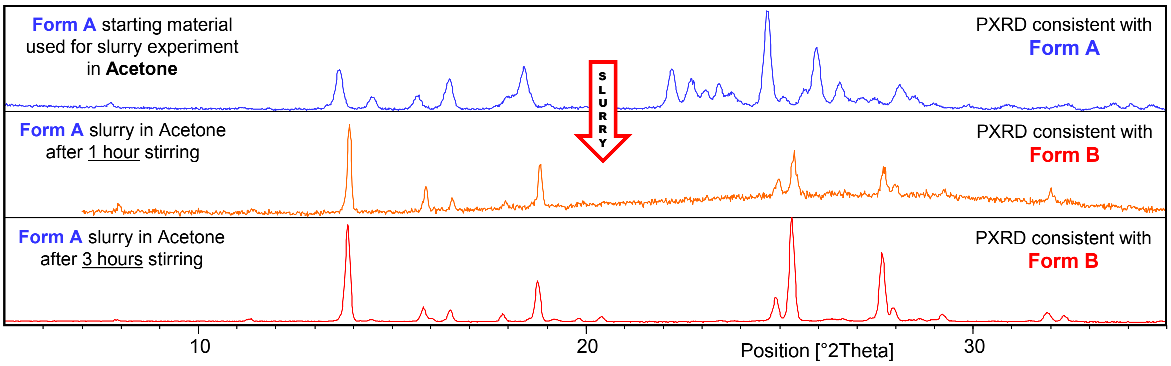

PXRD scan 39 PXRD scan of the residue of **Form A**, continuously stirring in a slurry in Acetone for 1 h and 3 hours

12.2.3 Slurry experiment of Form A in Tetrahydrofuran

Table S 105: Composition of **Form A** slurry in Tetrahydrofuran after stirring for different periods of time

Determining the thermodynamic stable polymorph at ambient temperature and pressure											
Slurry of **Form A** in Tetrahydrofuran											
	**1-1**		**2-2**		**Form B**		**Form A**		total polymorp	**R =**	Stable polymorph under ambient conditions
Slurry stirring for	**%M**	e.s.d. (mol%)	**%M**	e.s.d. (mol%)	**%M**	e.s.d. (mol%)	**%M**	e.s.d. (mol%)	[A]+[B]	[B] / [A]+[B]	
Starting material	Consistent with quantitative **Form A**: no Rietveld refinement performed.										
1 hour	1.8	2.4	1.5	2	**89**	3.7	**7.7**	2.3	96.7	**92%**	
3 hours	1.5	0.3	1.2	0.3	**96.4**	0.5	**1**	0.2	96.7	**92%**	**Form B**

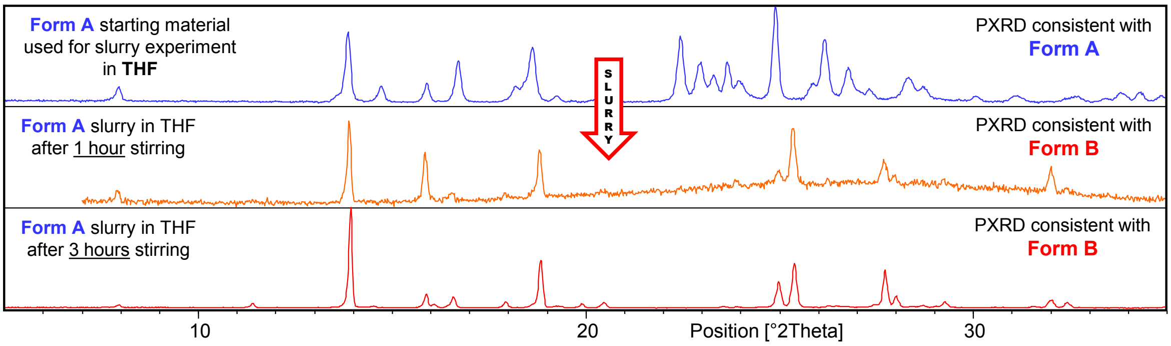

PXRD scan 40 PXRD scan of the residue of **Form A**, continuously stirring in a slurry in tetrahydrofuran for 1 and 3 hours.

12.2.4 Slurry experiment of Form A in Ethyl Acetate

Table S 106 Composition of **Form A** slurry in EtOAc after stirring for different periods of time

Determining the thermodynamic stable polymorph at ambient temperature and pressure											
Slurry of **Form A** in Ethyl Acetate											
	**1-1**		**2-2**		**Form B**		**Form A**		total polymorp	**R =**	Stable polymorph under ambient conditions
Slurry stirring for	**%M**	e.s.d. (mol%)	**%M**	e.s.d. (mol%)	**%M**	e.s.d. (mol%)	**%M**	e.s.d. (mol%)	[A]+[B]	[B] / [A]+[B]	
Starting material	Consistent with quantitative **Form A**: no Rietveld refinement performed.										
3 hours	1.1	0.3	0.9	0.3	**97.5**	0.5	**0.5**	0.2	98.0	**99%**	**Form B**

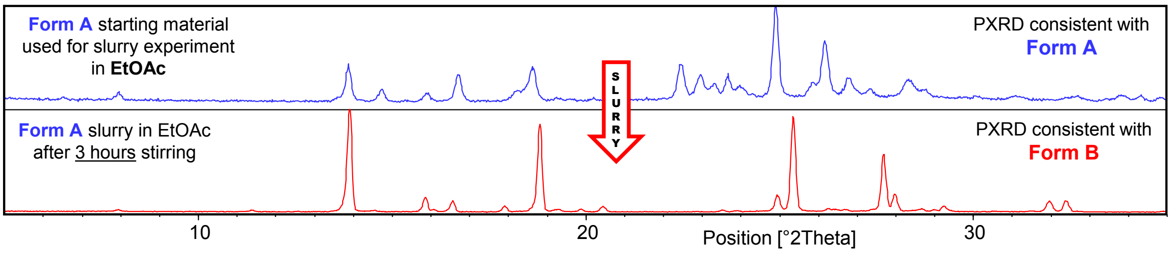

PXRD scan 41 PXRD scan of the residue of **Form A**, continuously stirring in a slurry in EtOAc for 3 hours.

12.2.5 Slurry experiment of Form A in Chloroform

Table S 107 Composition of **Form A** slurry in $CHCl_3$ after stirring for different periods of time

Determining the thermodynamic stable polymorph at ambient temperature and pressure											
Slurry of **Form A** in Chloroform											
	1-1		2-2		Form B		Form A		total polymorp	R =	Stable polymorph under ambient conditions
Slurry stirring for	%M	e.s.d. (mol%)	%M	e.s.d. (mol%)	%M	e.s.d. (mol%)	%M	e.s.d. (mol%)	[A]+[B]	[B] / ([A]+[B])	
Starting material	Consistent with quantitative **Form A**: no Rietveld refinement performed.										
1 hour	4.6	3	3.8	2.5	**87.8**	4	**3.9**	1.8	91.7	**96%**	
3 hours	0.9	0.3	0.8	0.2	**97.2**	0.4	**1.1**	0.2	98.3	**99%**	**Form B**

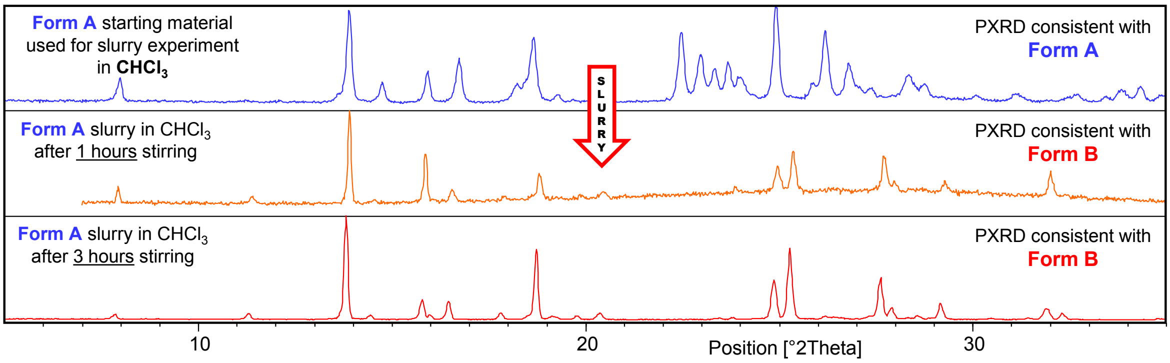

PXRD scan 42 PXRD scan of the residue of **Form A**, continuously stirring in a slurry in $CHCl_3$ for 1 and 3 hours

12.2.6 Slurry experiment of Form A in Dichloromethane

Table S 108 Composition of **Form A** slurry in DCM after stirring for different periods of time

Determining the thermodynamic stable polymorph at ambient temperature and pressure											
Slurry of **Form A** in Dichloromethane											
	1-1		2-2		Form B		Form A		total polymorp	R =	Stable polymorph under ambient conditions
Slurry stirring for	%M	e.s.d. (mol%)	%M	e.s.d. (mol%)	%M	e.s.d. (mol%)	%M	e.s.d. (mol%)	[A]+[B]	[B] / ([A]+[B])	
Starting material	Consistent with quantitative **Form A**: no Rietveld refinement performed.										
1 hour	2	1.4	1.6	1.1	**91.7**	2.4	**4.7**	1.9	96.4	**95%**	
3 hours	1.2	0.3	1	0.2	**96.7**	0.4	**1.1**	0.2	97.8	**99%**	**Form B**

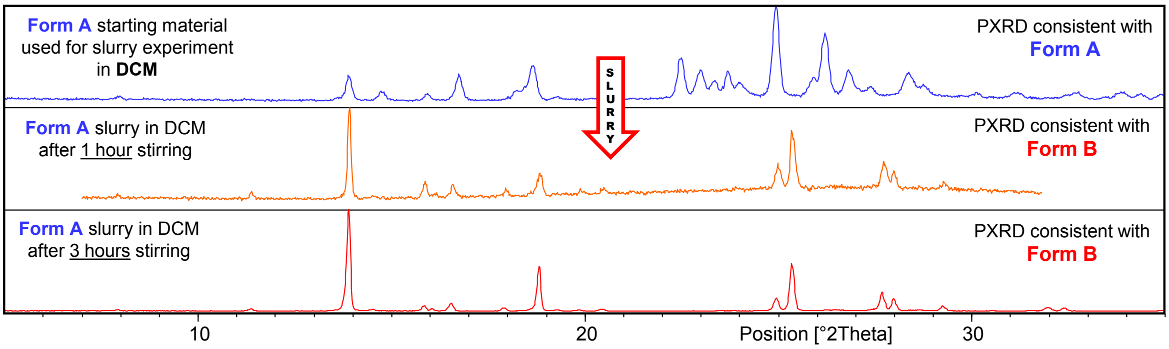

PXRD scan 43 PXRD scan of the residue of **Form A**, continuously stirring in a slurry in DCM for 1 and 3 hours

12.2.7 Slurry experiment of Form A in Dimethylformamide

Table S 109 Composition of **Form A** slurry in DMF after stirring for different periods of time

Determining the thermodynamic stable polymorph at ambient temperature and pressure

Slurry of **Form A** in Dimethylformamide

	1-1		2-2		Form B*		Form A		total polymorp	R =	Stable polymorph under ambient conditions
Slurry stirring for	%M	e.s.d. (mol%)	%M	e.s.d. (mol%)	%M	e.s.d. (mol%)	%M	e.s.d. (mol%)	[A]+[B]	[B] / [A]+[B]	
Starting material	Consistent with quantitative **Form A**: no Rietveld refinement performed.										
1 hour	6.5	3.6	5	2.8	**76.5**	5.7	**12**	5	88.5	**86%**	
3 hours	5.7	2	4.4	1.6	**63.2**	2.8	**26.7**	2.5	89.9	**70%**	**Form B**

* **Form B** decomposes with time in DMF. See Section xx

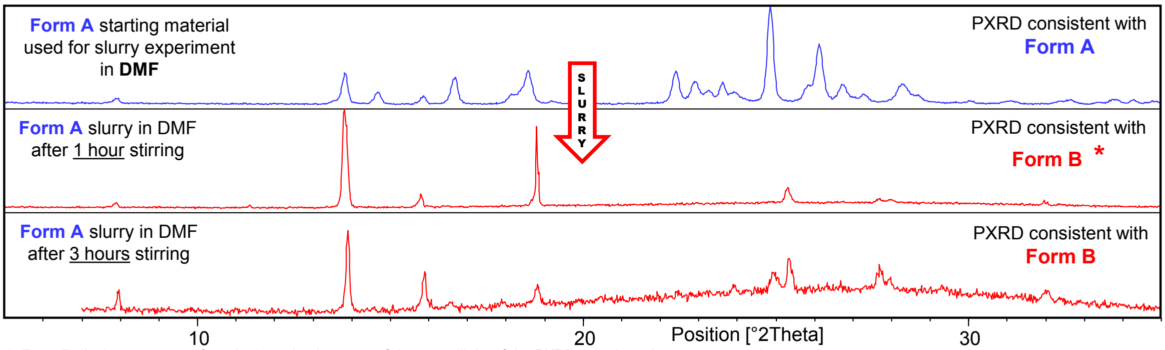

* **Form B** displays strong preferred orientation because of the crystallinity of the PXRD sample as it was not ground to powder.

PXRD scan 44 PXRD scan of the residue of **Form A**, continuously stirring in a slurry in DMF for 1 and 3 hours.

12.2.8 Slurry experiment of Form A in Dimethylsulphoxide

Table S 110 Composition of **Form A** slurry in DMSO after stirring for different periods of time

Determining the thermodynamic stable polymorph at ambient temperature and pressure

Slurry of **Form A** in Dimethylsulphoxide

	1-1		2-2		Form B *		Form A		total polymorp	R =	Stable polymorph under ambient conditions
Slurry stirring for	%M	e.s.d. (mol%)	%M	e.s.d. (mol%)	%M	e.s.d. (mol%)	%M	e.s.d. (mol%)	[A]+[B]	[B] / [A]+[B]	
Starting material	Consistent with quantitative **Form A**: no Rietveld refinement performed.										
1 hour	2.3	1.4	1.9	1.2	**91.2**	2.2	**4.6**	1.3	95.8	**95%**	
3 hours	7	2.7	5.7	2.3	**74.6**	4.3	**12.7**	3.7	87.3	**85%**	**Form B**

* **Form B** decomposes with time in DMSO.

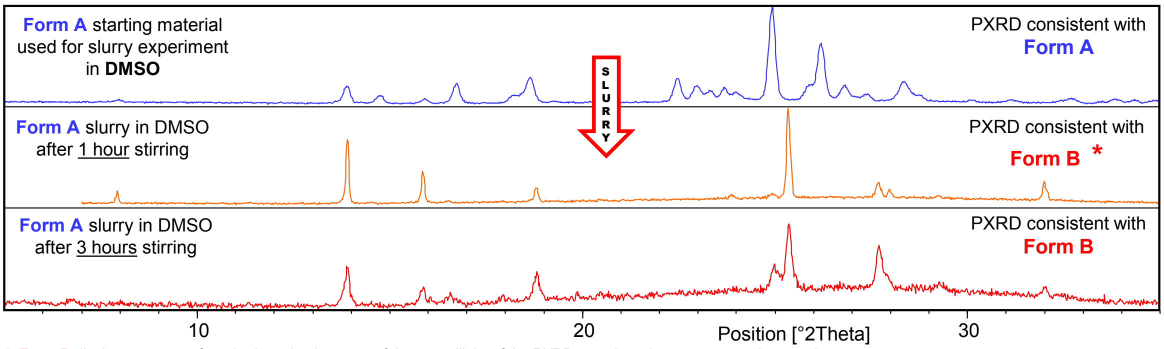

* **Form B** displays strong preferred orientation because of the crystallinity of the PXRD sample as it was not ground to powder.

PXRD scan 45 PXRD scan of the residue of **Form A**, continuously stirring in a slurry in DMSO for 1 and 3 hours

12.2.9 Slurry experiment of Form A in Cyclohexane

Table S 111 Composition of **Form A** slurry in cyclohexane after stirring for different periods of time

Determining the thermodynamic stable polymorph at ambient temperature and pressure											
Slurry of **Form A** in Cyclohexane											
	**1-1**		**2-2**		**Form B**		**Form A**		total polymorp	**R =**	Stable polymorph
Slurry stirring for	**%M**	e.s.d. (mol%)	**%M**	e.s.d. (mol%)	**%M**	e.s.d. (mol%)	**%M**	e.s.d. (mol%)	[A]+[B]	[B] / [A]+[B]	under ambient conditions
Starting material	Consistent with quantitative **Form A**: no Rietveld refinement performed.										
3 hours	0.7	0.4	0.6	0.3	**97.5**	0.7	**1.2**	0.4	98.7	**99%**	**Form B**

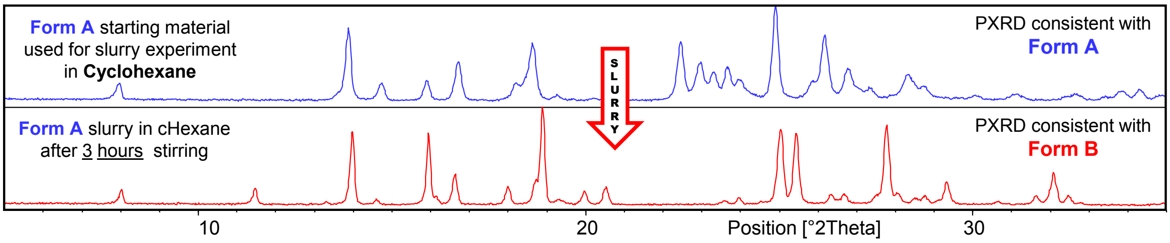

PXRD scan 46 PXRD scan of the residue of **Form A**, continuously stirring in a slurry in cyclohexane for 3 hours.

12.2.10 Slurry experiment of Form A in Benzene

Table S 112 Composition of **Form A** slurry in benzene after stirring for different periods of time

Determining the thermodynamic stable polymorph at ambient temperature and pressure											
Slurry of **Form A** in Benzene											
	**1-1**		**2-2**		**Form B**		**Form A**		total polymorp	**R =**	Stable polymorph
Slurry stirring for	**%M**	e.s.d. (mol%)	**%M**	e.s.d. (mol%)	**%M**	e.s.d. (mol%)	**%M**	e.s.d. (mol%)	[A]+[B]	[B] / [A]+[B]	under ambient conditions
Starting material	Consistent with quantitative **Form A**: no Rietveld refinement performed.										
1 hour	13.2	4.3	10.7	3.6	**43.4**	4.4	**32.7**	5.2	76.1	**57%**	
3 hours	1.3	0.5	1	0.4	**96.6**	0.7	**1.1**	0.3	97.7	**99%**	**Form B**

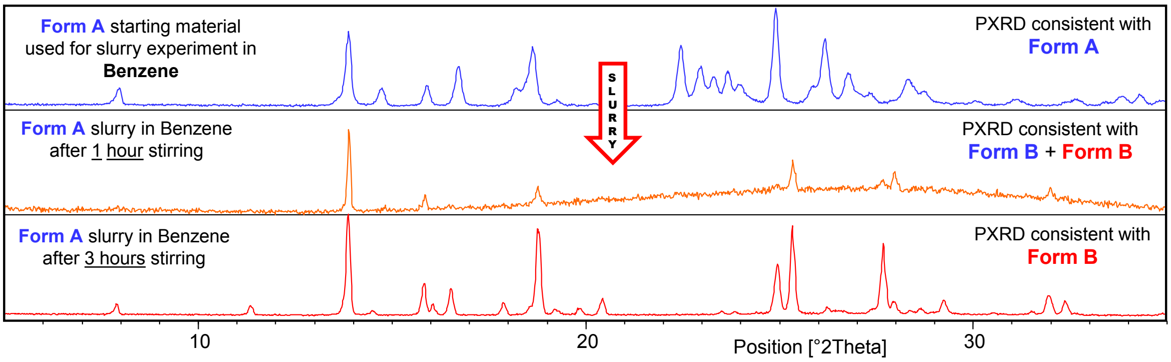

PXRD scan 47 PXRD scan of the residue of **Form A**, continuously stirring in a slurry in Benzene for 1 and 3 hours.

12.2.11 Slurry experiment of Form A in Toluene

Table S 113 Composition of **Form A** slurry in Toluene after stirring for different periods of time

Determining the thermodynamic stable polymorph at ambient temperature and pressure											
Slurry of **Form A** in Toluene											
	**1-1**		**2-2**		**Form B** $		**Form A**		total polymorp	R =	Stable polymorph
Slurry stirring for	**%M**	e.s.d. (mol%)	**%M**	e.s.d. (mol%)	**%M**	e.s.d. (mol%)	**%M**	e.s.d. (mol%)	[A]+[B]	[B] / [A]+[B]	under ambient conditions
Starting material	Consistent with quantitative **Form A**: no Rietveld refinement performed.										
1 hour	6	3.8	4.7	3.1	**63.3**	5.1	**26**	4.6	89.3	**71%**	**Form B**

$ **Form A** required >>1 hour to transform fully to **Form B**

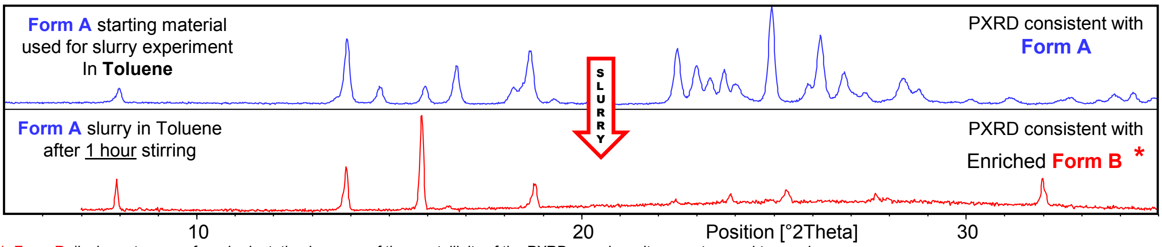

* **Form B** displays strong preferred orientation because of the crystallinity of the PXRD sample as it was not ground to powder.

PXRD scan 48 PXRD scan of the residue of **Form A**, continuously stirring in a slurry in Toluene for 1 hour.

12.2.12 Slurry experiment of Form A in Methanol

Table S 114 Composition of **Form A** slurry in Methanol after stirring for different periods of time

Determining the thermodynamic stable polymorph at ambient temperature and pressure											
Slurry of **Form A** in Methanol											
	**1-1**		**2-2**		**Form B**		**Form A**		total polymorp	R =	Stable polymorph
Slurry stirring for	**%M**	e.s.d. (mol%)	**%M**	e.s.d. (mol%)	**%M**	e.s.d. (mol%)	**%M**	e.s.d. (mol%)	[A]+[B]	[B] / [A]+[B]	under ambient conditions
Starting material	Consistent with quantitative **Form A**: no Rietveld refinement performed.										
3 hours	1	0.4	0.8	0.3	1	0.4	**97.2**	0.6	98.2	**1%**	
1 week	1.9	1.5	1.6	1.3	**94.9**	2.1	**1.6**	0.9	96.5	**98%**	**Form B**

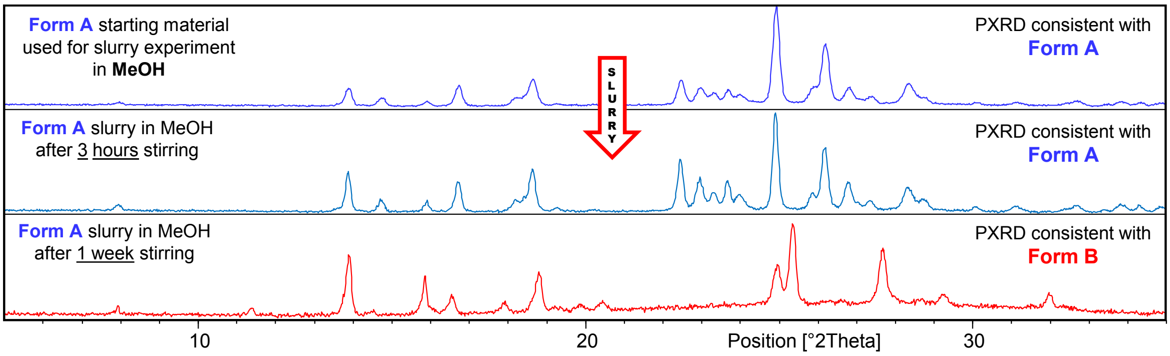

PXRD scan 49 PXRD scan of the residue of **Form A**, continuously stirring in a slurry in Methanol for 3 hours and 1 week.

12.2.13 Slurry experiment of Form A in Ethanol

Table S 115 Composition of **Form A** slurry in Ethanol after stirring for different periods of time

Determining the thermodynamic stable polymorph at ambient temperature and pressure

Slurry of **Form A** in Ethanol

	1-1		2-2		Form B		Form A		total polymorp	R =	Stable polymorph under ambient conditions
Slurry stirring for	%M	e.s.d. (mol%)	%M	e.s.d. (mol%)	%M	e.s.d. (mol%)	%M	e.s.d. (mol%)	[A]+[B]	[B] / [A]+[B]	
Starting material	Consistent with quantitative **Form A**: no Rietveld refinement performed.										
3 hours	1.6	1	1.2	0.8	**5.6**	1.4	**91.6**	1.8	97.2	**6%**	
1 week	3.5	0.8	2.8	0.6	**89.3**	1.2	**4.4**	0.9	93.7	**95%**	**Form B**

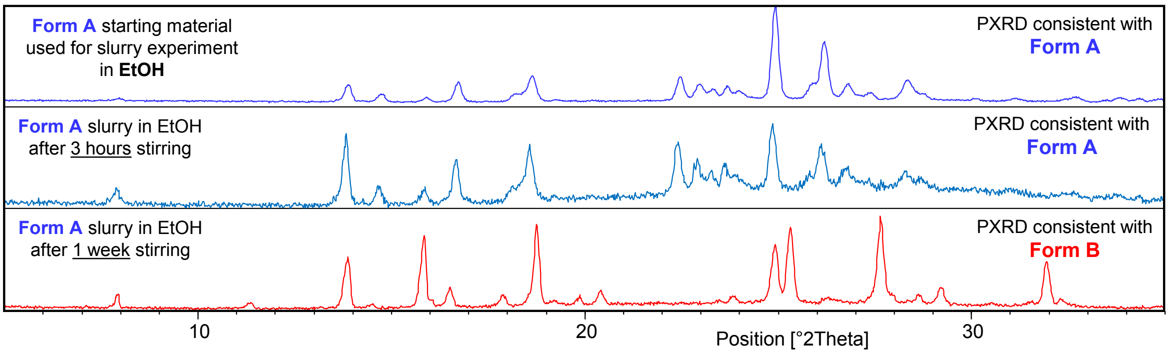

PXRD scan 50 PXRD scan of the residue of **Form A**, continuously stirring in a slurry in Ethanol for 3 hours and 1 week.

12.2.14 Slurry experiment of Form A in Isopropanol

Table S 116 Composition of **Form A** slurry in Isopropanol after stirring for different periods of time

Determining the thermodynamic stable polymorph at ambient temperature and pressure

Slurry of **Form A** in Isopropanol

	1-1		2-2		Form B $		Form A		total polymorp	R =	Stable polymorph under ambient conditions
Slurry stirring for	%M	e.s.d. (mol%)	%M	e.s.d. (mol%)	%M	e.s.d. (mol%)	%M	e.s.d. (mol%)	[A]+[B]	[B] / [A]+[B]	
Starting material	Consistent with quantitative **Form A**: no Rietveld refinement performed.										
3 hours	0.7	0.4	0.5	0.3	**1.7**	0.5	**97.1**	0.7	98.8	**2%**	
1 week	6.3	2.4	4.9	1.9	**74.2**	3.7	**14.6**	3.2	88.8	**84%**	**Form B**
$ **Form A** required >>1 week to transform fully to **Form B**											

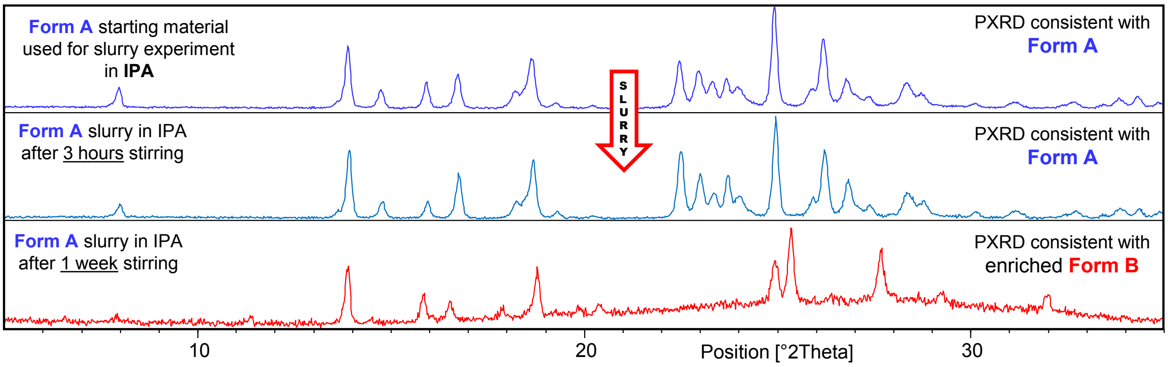

PXRD scan 51 PXRD scan of the residue of **Form A**, continuously stirring in a slurry in Isopropanol for 3 hours and 1 weeks

12.2.15 Slurry experiment of Form A in Water

Table S 117 Composition of **Form A** slurry in water after stirring for different periods of time

Determining the thermodynamic stable polymorph at ambient temperature and pressure

Slurry of **Form A** in Water

Slurry stirring for	1-1 %M	1-1 e.s.d. (mol%)	2-2 %M	2-2 e.s.d. (mol%)	Form B $ %M	Form B $ e.s.d. (mol%)	Form A %M	Form A e.s.d. (mol%)	total polymorp [A]+[B]	R = [B] / ([A]+[B])	Stable polymorph under ambient conditions
Starting material	Consistent with quantitative **Form A**: no Rietveld refinement performed.										
1 hour	1.8	0.6	1.4	0.5	**15**	1.2	**81.8**	1.4	96.8	**15%**	
3 hours	1.1	0.5	0.8	0.4	**15.3**	0.8	**82.8**	1	98.1	**16%**	
1 week	6.2	2.5	4.8	2	**16.5**	2.8	**72.5**	3.6	89.0	**19%**	
3 weeks	2	0.6	1.7	0.6	**23.3**	1	**73.0**	1.2	96.3	**24%**	**Form B**
2 months	1.8	0.7	1.7	0.7	**23.9**	1.7	**72.6**	1.8	96.5	**25%**	increases

$ **Form A** requires >>2 months to transform fully to **Form B**

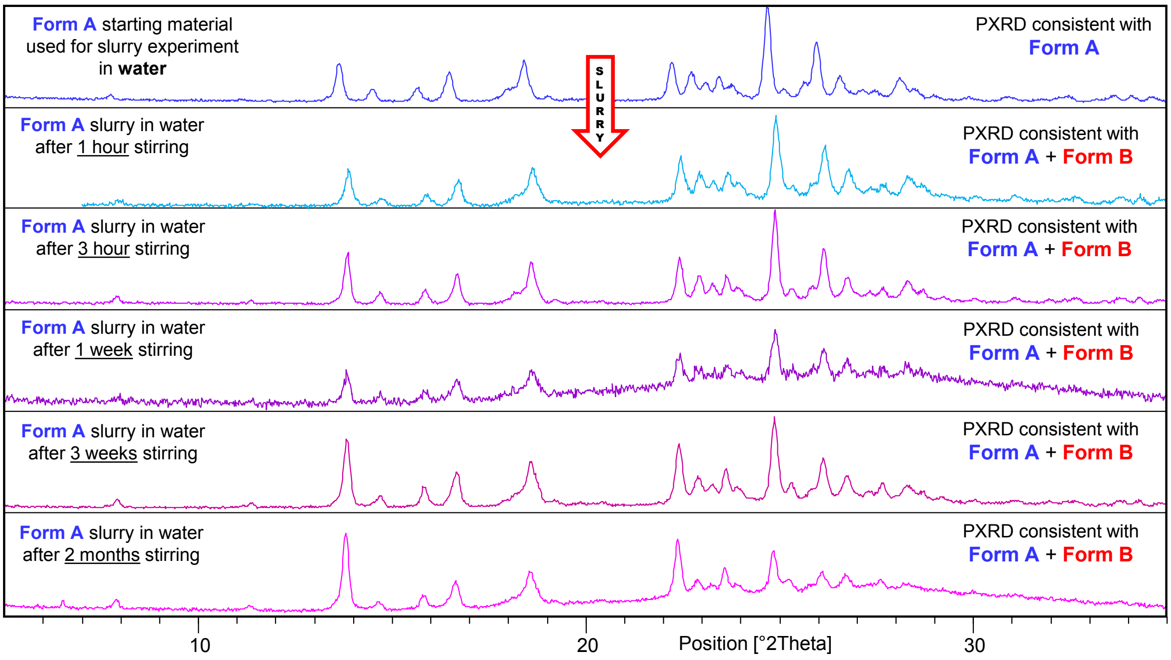

PXRD scan 52 PXRD scan of the residue of **Form A**, continuously stirring in a slurry in Water for 1 hour to 2 months.

12.2.16 Discussion and conclusions on slurry experiments of Form A in 15 solvents

Form A is transformed in all solvent under slurry conditions to **Form B**. Therefore **form A** is the metastable polymorph. It takes a few weeks for the alcohols to fully transform to **Form B**. Due to its poor solubility in water, **Form A** may take even years to fully transform to **Form B**; within 2 months **Form A** has already transformed 25% to **form B**. **Form A** just as **Form B** are quite unstable in DMF and DMSO; after 30 minutes to 1 hour, degradation is observed.

12.3 Overall conclusions of slurry experiments

Form B is the thermodynamic polymorph under slurry conditions in any solvent while **Form A** is the metastable polymorph and transforms with time to **Form B**.

12.3.1 Summary of solubility determination and slurry experiments of 1-1, 2-2, Form A and Form B in 15 solvents expressed as mg/mL

Table S 118: Summary of solubility obtained for 1-1, 2-2, Form A & Form B expressed mg/mL

Solubility experiments in following solvents	Solubility of 2-2 (mg/mL)	Solubility of 1-1 (mg/mL)	Solubility of (1-2)B (mg/mL)	(1-2)B PXRD slurry residue end solub exp. consistent with	Solubility of (1-2)A (mg/mL)	(1-2)A PXRD slurry residue 10 min stir. consistent with: (Note 1)
Water	0	0.0	0.0	(1-2)B	0	10 min stirr (1-2)A<br>1week stirr some (1-2)B<br>>1month stirr (1-2)B
MeOH	14	0.2	3.5	(1-2)B	4.3	10 min stirr (1-2)A<br>1week stirr (1-2)B
EtOH	27	0.3	5.3	(1-2)B	6.4	10 min stirr (1-2)A<br>1week stirr (1-2)B
IPA	18	0.2	3.4	(1-2)B	3.9	10 min stirr (1-2)A<br>1week stirr (1-2)B
MeCN	29	3.4	19	(1-2)B	18	(1-2)B
DMSO	128	7.9	70	(1-2)B	62	(1-2)B
cyclohexane	145	0.2	6.4	(1-2)B	6.3	(1-2)B
Acetone	266	7.4	78	(1-2)B	78	(1-2)B
EtOAc	352	4.5	77	(1-2)B	77	(1-2)B
DMF	304	16.0	129	(1-2)B	211	(1-2)B
Toluene	522	4.4	132	(1-2)B	123	(1-2)B
Benzene	565	5.6	141	(1-2)B	145	(1-2)B
$CHCl_3$	603	17.3	248	(1-2)B	252	(1-2)B
DCM	659	27.9	266	(1-2)B	270	(1-2)B
THF	684	29.4	334	(1-2)B	344	(1-2)B

Note 1: the solid residue of Form A of the slurry experiment after stirring in water over 8 weeks was analysed by PXRD and found to have been partially converted from Form A to Form B .

12.3.2 Summary of solubility determination and slurry experiments of 1-1, 2-2, Form A and Form B in 15 solvents expressed as mmol/mol solvent

Table S 119: Summary of solubility obtained for 1-1, 2-2, Form A & Form B expressed mmol/mol_solvent

Solubility experiments in following solvents	Solubility of 2-2 (mmol/mol)	Solubility of 1-1 (mmol/mol)	Solubility of (1-2)B (mmol/mol)	(1-2)B PXRD slurry residue end solub exp. consistent with	Solubility of (1-2)A (mmol/mol)	(1-2)A PXRD slurry residue 10 min stir. consistent with: (Note 1)
Water	0	0.0	0.0	(1-2)B	0.0	10 min stirr (1-2)A<br>1week stirr some (1-2)B<br>>1month stirr (1-2)B
MeOH	2	0.0	0.5	(1-2)B	0.6	10 min stirr (1-2)A<br>1week stirr (1-2)B
EtOH	6	0.1	1.0	(1-2)B	1.3	10 min stirr (1-2)A<br>1week stirr (1-2)B
IPA	5	0.0	0.9	(1-2)B	1.0	10 min stirr (1-2)A<br>1week stirr (1-2)B
MeCN	5	0.6	3.4	(1-2)B	3.1	(1-2)B
DMSO	32	1.8	16.7	(1-2)B	14.8	(1-2)B
cyclo-hexane	55	0.1	2.3	(1-2)B	2.3	(1-2)B
Acetone	69	1.8	19.3	(1-2)B	19.4	(1-2)B
EtOAc	121	1.4	25.3	(1-2)B	25.5	(1-2)B
DMF	62	3.1	25.6	(1-2)B	42.0	(1-2)B
Toluene	194	1.5	47.5	(1-2)B	44.2	(1-2)B
Benzene	177	1.6	42.5	(1-2)B	43.8	(1-2)B
$CHCl_3$	169	4.5	67.2	(1-2)B	68.3	(1-2)B
DCM	148	5.8	57.5	(1-2)B	58.5	(1-2)B
THF	194	7.8	91.4	(1-2)B	94.4	(1-2)B

Note1: solid residue of the slurry after stirring in water over 8 weeks was analysed by PXRD and found to have been fully converted from Form A to Form B.

Note2: The solubility experiments were also performed for 1-1 and 2-2, however in this document we only report the results as they are not important for the thermodynamic outcome of the polymorphs.

13 Determination of the particle size of Form A and From B from PXRD scans by Scherrer calculations

13.1 Determination of particle size of Form A and From B from turnover experiments of polymorph interconversion of solid state DCC (contains dbu)

Table S 120: Tabulation of the phase composition and particle size (nm) of the consecutive formation of **Form A** from NG of dried powder of **Form B** and **Form B** by LAG (50µL MeCN) from **Form A**. Phase composition was obtained by Rietveld refinement while particle size was obtained from Scherrer calculation from the PXRD scans.

Particle size calculation for **Form A** and **Form B** using Scherrer equation on PXRD scans

Turnover polymorph interconversion between **Form A** & **Form B** in presence of **dbu**

Turn-over experi-ments	grinding time @30Hz to equilibrium.	grinding conditions (solvent) / dbu is present	1-1 %M	2-2 %M	Form B %M	Form A %M	[A]+[B] total polymorph	R = [B]/([A]+[B]) % R	Scherrer size Form B crystal size (nm)	Form B e.s.d. crystal size (nm)	Form A crystal size (nm)	Form A e.s.d. crystal size (nm)
Starting material		2uL dbu + 50µL MeCN	2	1.8	**95.5**	0.7	96.2	**99%**	**67 nm**	1		
Tover-1	45 min	NEAT	2.3	2	4.2	**91.5**	95.7	**4%**			**40 nm**	0.7
Tover-1	90 min	50 ul MeCN	4.1	3.7	**91.2**	1.1	92.3	**99%**	**47 nm**	0.8		
Tover-2	45 min	NEAT	3.3	2.9	4.1	**89.7**	93.8	**4%**			**43 nm**	0.8
Tover-2	90 min	50 ul MeCN	2.2	2.1	**95.3**	0.4	95.7	**100%**	**72 nm**	1		
Tover-3	45 min	NEAT	3	2.7	3	**91.3**	94.3	**3%**			**40 nm**	0.8
Tover-3	90 min	50 ul MeCN	2.1	1.9	**95.6**	0.4	96.0	**100%**	**79 nm**	1		
Tover-4	45 min	NEAT	4	3.5	2.2	**90.3**	92.5	**2%**			**40 nm**	0.9
Tover-4	90 min	50 ul MeCN	1.5	1.4	**96.7**	0.4	97.1	**100%**	**81 nm**	1		
Tover-5	45 min	NEAT	3.8	3.4	1.7	**91.1**	92.8	**2%**			**45 nm**	9
Tover-5	90 min	50 ul MeCN	1.5	1.4	**96.7**	0.4	97.1	**100%**	**79 nm**	1		
Tover-6	45 min	NEAT	5.1	4.5	3.5	**86.9**	90.4	**4%**			**47 nm**	0.9

Note: In the presence of dbu (base catalyst) the polymorph interconversion between **Form A** and **Form B** goes via solid state Dynamic Covalent Chemistry (DCC) involving bond breaking and bond forming. Polymorph interconversion by solid state DCC can involve the homodimers **1-1** and **2-2** as intermediates.[20]

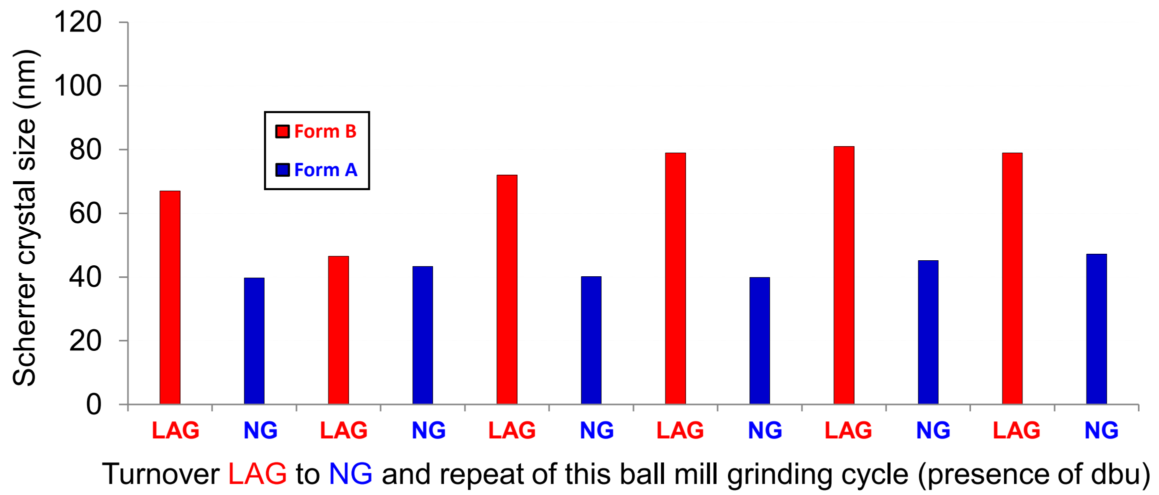

Figure S 86 Comparison of particle size of **Form B** (obtained by NG of **Form A**) and **Form A** (obtained by LAG from dried **Form B**) in 5 polymorph interconversion turnover experiments (with dbu)

13.2 Determination of particle size of Form A and From B from turnover experiments of direct polymorph interconversion (absence of dbu)

Table S 121: Tabulation of the phase composition and particle size (nm) of the consecutive formation of **Form A** from NG of dried powder of **Form B** and **Form B** by LAG (50µL MeCN) from **Form A**. Phase composition was obtained by Rietveld refinement while particle size was obtained by Scherrer calculation from the PXRD scans.

Particle size calculation for **Form A** and **Form B** using Scherrer equation on PXRD scans

Turnover of direct polymorph interconversion between **Form A** & **Form B** (absence of **dbu**)

Turn-over experi-ments	Grinding time @30Hz to equilibrium.	grinding conditions (solvent)	1-1	2-2	Form B	Form A	[A]+[B]	R = [B]/([A]+[B])	Scherrer size: Form B		Scherrer size: Form A	
		**dbu** is absent	%M	%M	%M	%M	total polymorph	% R	crystal size (nm)	e.s.d. crystal size (nm)	crystal size (nm)	e.s.d. crystal size (nm)
Tover-1	3x50 min	NEAT	1.8	1.7	3.8	**92.7**	96.5	**4%**			**77 nm**	0.9
Tover-1	30 min	50 ul MeCN	1.8	1.6	**95.9**	0.7	96.6	**99%**	**76 nm**	0.9		
Tover-2	3x50 min	NEAT	1.7	1.7	3.6	**93.0**	96.6	**4%**			**69 nm**	0.8
Tover-2	30 min	50 ul MeCN	1.6	1.5	**95.8**	1.1	96.9	**99%**	**76 nm**			
Tover-3	3x50 min	NEAT	1.0	0.9	3.1	**95.0**	98.1	**3%**			**72 nm**	0.8
Tover-3	30 min	50 ul MeCN	1.4	1.3	**94.4**	1.7	96.1	**98%**	**74 nm**	0.8		
Tover-4	3x50 min	NEAT	1.4	1.3	4.1	**93.2**	97.3	**4%**			**62 nm**	0.8
Tover-4	30 min	50 ul MeCN	1.4	1.3	**96.4**	1.0	97.4	**99%**	**98 nm**	1		
Tover-5	3x50 min	NEAT	2.1	1.3	4.1	**93.2**	97.3	**4%**			**58 nm**	0.8
Tover-5	30 min	50 ul MeCN	2.4	2.1	**91.7**	3.7	95.4	**96%**	**87 nm**	1		

Note 1: In the absence of dbu, the disulfide bond cannot break or reform. Therefore here we have a direct polymorph interconversion between **Form A** and **Form B**.

Note 2: After extensive grinding of the dried powder of **Form B** to obtain the thermodynamic product, **Form A** is formed as a very fine and extremely static powder making transferring this powder to a PXRD slide very laborious. To overcome this problem, IPA or other solvents are added to the powder at the final stage and ground for a few seconds (20 seconds) at 20 Hz, just to wet the powder. This powder is much easier to handle as far as it is worked immediately on opening the grinding jar to avoid the powder from drying again. This wetness of the **Form A** powder when running the PXRD may account for the particle size for **Form A** having higher values than those obtained under solid state DCC (see Table S120) where **Form A** can be easily transferred dry to the PXRD slide.

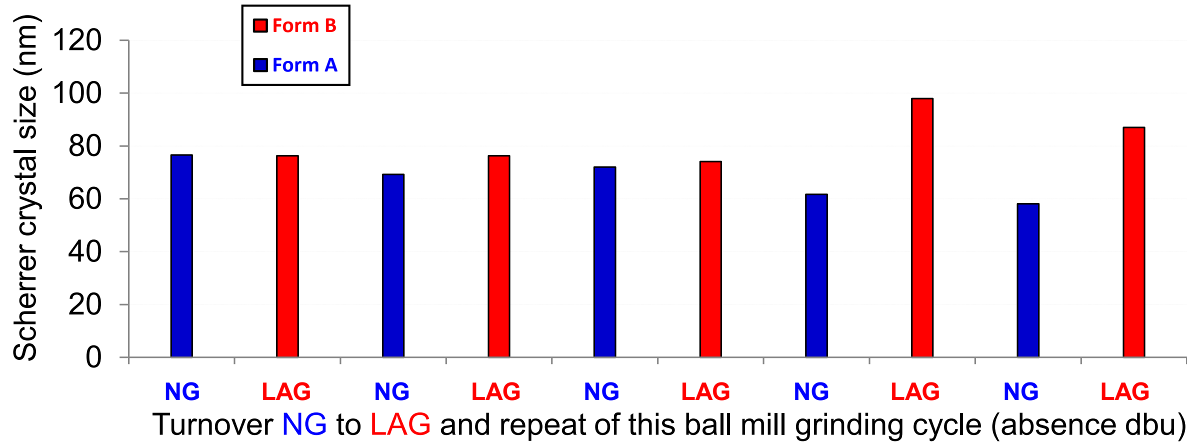

Figure S 87 Comparison of particle size of **Form A** (obtained by LAG from dried **Form B**) and **Form B** (obtained by NG of **Form A**) in 5 polymorph interconversion turnover experiments (absence of dbu)

13.3 Determination of particle size of Form A and From B from LAG experiments with different LAG solvents in solid state DCC (presence of dbu)

13.3.1 Particle size determination for Form A & Form B with Acetone as LAG solvent

Table S 122 Calculation of particle size using Scherrer equation on PXRD data using Acetone as solvent in LAG

Scherrer crystal size (nm) for **Form A** and **Form B**													
Ball mill LAG reaction taken to thermodynamic equilibrium using Procedure 1													
2%M dbu+µL **Acetone** added to 0.68 mmol DCC ([1-1]+[2-2]+[Form A]+[Form B])													
grinding time @ 30Hz to equilib.	acetone added to powder		1-1	2-2	Form B	Form A	[A]+[B]	R = [B] / [A]+[B]	Scherrer size Form B		Scherrer size Form A		
	acetone µL	[acetone]/[DCC] (mol/mol)	%M	%M	%M	%M	total polymorph	% R	crystal size (nm)	e.s.d. crystal size (nm)	crystal size (nm)	e.s.d. crystal size (nm)	
45m	**10**	**0.20**	1.1	0.9	**2.1**	**95.9**	98.0	**2 %**			**70 nm**	1.0	
45m	**14**	**0.28**	0.8	0.6	**2.8**	**95.8**	98.6	**3 %**			**78 nm**	0.9	
45m	**15**	**0.30**	0.7	0.6	**2.1**	**96.6**	98.7	**2 %**			**74 nm**	0.8	
45m	**16**	**0.32**	0.9	0.7	**2.1**	**96.3**	98.4	**2 %**			**79 nm**	0.9	
45m	**17**	**0.34**	2.3	1.8	**92.9**	**3.0**	95.9	**97 %**	**75 nm**	1.0			
45m	**18**	**0.36**	2.8	2.3	**92.8**	**2.1**	94.9	**98 %**	**76 nm**	0.9			
45m	**20**	**0.40**	3.1	2.5	**92.3**	**2.1**	94.4	**98 %**	**81 nm**	0.9			
45m	**30**	**0.60**	4.9	3.9	**89.3**	**1.9**	91.2	**98 %**	**90 nm**	1.0			
45m	**50**	**0.99**	4.4	3.6	**90.8**	**1.2**	92.0	**99 %**	**109 nm**	2.0			
[DCC] is the concentration of the dynamic covalent chemistry reaction at any time =[1-1]+[2-2]+[1-2]													
[1-2] = **Form A** + **Form B**													

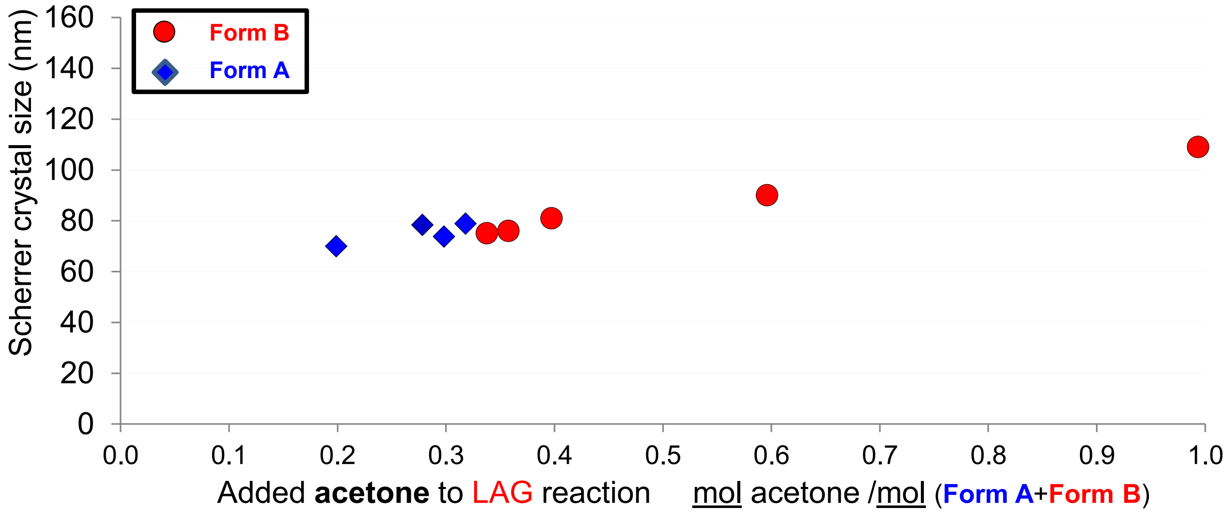

Figure S 88 Plot of Scherrer crystal size expressed as nm versus amount acetone added to LAG experiment expressed as mol Acetone per mol of **1-2** (**Form A**+**Form B**).

13.3.2 Particle size determination for Form A & Form B with THF as LAG solvent

Table S 123 Calculation of particle size using Scherrer equation on PXRD data using THF as solvent in LAG

Scherrer crystal size (nm) for **Form A** and **Form B**												
Ball mill LAG reaction taken to thermodynamic equilibrium using Procedure 1												
2%M dbu+µL **THF** added to 0.68 mmol DCC ([**1-1**]+[**2-2**]+[**Form A**]+[**Form B**])												
grinding time @ 30Hz to equilib.	THF added to powder		**1-1**	**2-2**	**Form B**	**Form A**	[**A**]+[**B**]	R = [**B**] / ([**A**]+[**B**])	Scherrer size **Form B**		Scherrer size **Form A**	
	THF µL	[THF]/[DCC] (mol/mol)	%M	%M	%M	%M	total polymorph	% R	crystal size (nm)	e.s.d. crystal size (nm)	crystal size (nm)	e.s.d. crystal size (nm)
1h	**15**	**0.27**	2.4	1.9	**3.1**	**92.6**	95.7	**3 %**			**79 nm**	1
1h	**17**	**0.31**	3.8	3.0	**3.5**	**89.7**	93.2	**4 %**			**88 nm**	1
1h	**18**	**0.32**	3.0	2.4	**3.1**	**91.5**	94.6	**3 %**			**86 nm**	1
1h	**18**	**0.32**	7.3	5.9	**4.2**	**82.5**	86.7	**5 %**			**81 nm**	1
1h30m	**19**	**0.34**	4.2	3.4	**73.8**	**18.6**	92.4	**80 %**	**100 nm**	2	**89 nm**	4
2h	**20**	**0.36**	7.5	6.0	**77.9**	**8.6**	86.5	**90 %**	**94 nm**	2	**89 nm**	9
1h	**21**	**0.38**	5.4	4.3	**75.5**	**14.8**	90.3	**84 %**				
1h	**22**	**0.40**	5.8	4.6	**86.7**	**2.9**	89.6	**97 %**				
1h	**23**	**0.41**	5.6	4.5	**84.1**	**5.8**	89.9	**94 %**				
1h	**25**	**0.45**	5.7	4.6	**89.1**	**0.6**	89.7	**99 %**	**94 nm**	1		
1h	**30**	**0.54**	2.3	1.8	**94.5**	**1.4**	95.9	**99 %**				

[DCC] is the concentration of the dynamic covalent chemistry reaction at any time =[**1-1**]+[**2-2**]+[**Form A**]+[**Form B**]

[**1-2**] = **Form A** + **Form B** [**A**] ≡ [**Form A**] [**B**] ≡ [**Form B**]

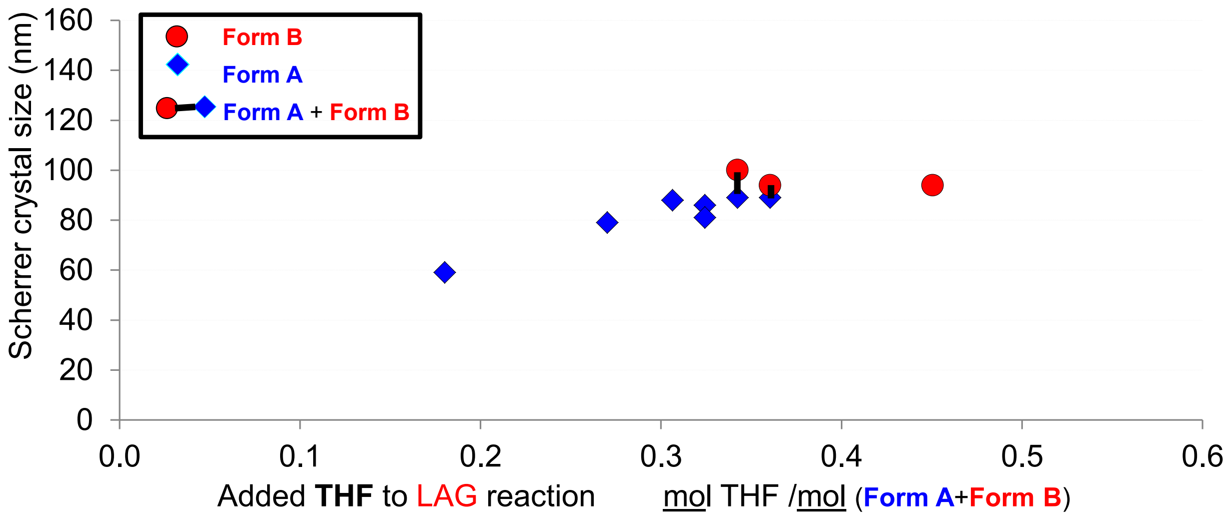

Figure S 89 Plot of Scherrer crystal size expressed as nm versus amount acetone added to LAG experiment expressed as mol THF per mol of **1-2** (**Form A**+**Form B**).

13.3.3 Particle size determination for Form A & Form B with DMF as LAG solvent

Table S 124 Calculation of particle size using Scherrer equation on PXRD data using DMF as solvent in LAG

Scherrer crystal size (nm) for **Form A** and **Form B**

Ball mill LAG reaction taken to thermodynamic equilibrium using Procedure 1

2%M dbu+µL DMF added to 0.68 mmol DCC ([**1-1**]+[**2-2**]+[**Form A**]+[**Form B**])

grinding time @ 30Hz to equilib.	DMF added to powder		**1-1**	**2-2**	**Form B**	**Form A**	[A]+[B]	R = [B] / ([A]+[B])	**Scherrer size** Form B		**Scherrer size** Form A	
	DMF µL	[DMF]/[DCC] (mol/mol)	%M	%M	%M	%M	total polymorph	% R	crystal size (nm)	e.s.d. crystal size (nm)	crystal size (nm)	e.s.d. crystal size (nm)
2h30m	**5**	**0.12**	**1.5**	**1.2**	**4.7**	**92.6**	97.3	**5 %**				
3h	**10**	**0.25**	**3.0**	**2.4**	**1.9**	**92.7**	94.6	**2 %**				
3h	**13**	**0.32**	**6.3**	**5.1**	**3.8**	**84.9**	88.7	**4 %**				
2h30m	**14**	**0.35**	**1.0**	**0.8**	**3.0**	**95.3**	98.3	**3 %**				
2h	**15**	**0.37**	**1.8**	**1.4**	**13.1**	**83.7**	96.8	**14 %**				
3h	**16**	**0.40**	**8.8**	**7.1**	**13.0**	**71.1**	84.1	**15 %**				
2h	**16**	**0.40**	**1.8**	**1.5**	**30.2**	**66.5**	96.7	**31 %**				
3h	**17**	**0.42**	**0.8**	**0.7**	**67.4**	**31.1**	98.5	**68 %**				
2h	**18**	**0.45**	**5.9**	**4.8**	**39.8**	**49.5**	89.3	**45 %**				
3h	**19**	**0.47**	**1.6**	**1.3**	**54.9**	**42.1**	97.0	**57 %**				
3h	**20**	**0.50**	**1.5**	**1.2**	**78.4**	**19.0**	97.4	**80 %**				
3h	**21**	**0.52**	**6.5**	**5.2**	**59.7**	**28.6**	88.3	**68 %**	**102 nm**	2	**100 nm**	3
2h30m	**22**	**0.55**	**1.0**	**0.8**	**97.4**	**0.8**	98.2	**99 %**				
3h	**25**	**0.62**	**1.2**	**1.0**	**93.7**	**4.0**	97.7	**96 %**				
2h	**28**	**0.70**	**0.9**	**0.7**	**96.7**	**1.6**	98.3	**98 %**	**111 nm**	2		
2h	**30**	**0.75**	**1.7**	**1.4**	**96.5**	**0.4**	96.9	**100 %**				
2h30m	**50**	**1.24**	**3.0**	**2.4**	**93.9**	**0.6**	94.5	**99 %**				

[DCC] is the concentration of the dynamic covalent chemistry reaction at any time =[**1-1**]+[**2-2**]+[**Form A**]+[**Form B**]

[**1-2**] = **Form A** + **Form B** [**A**] ≡ [**Form A**] [**B**] ≡ [**Form B**]

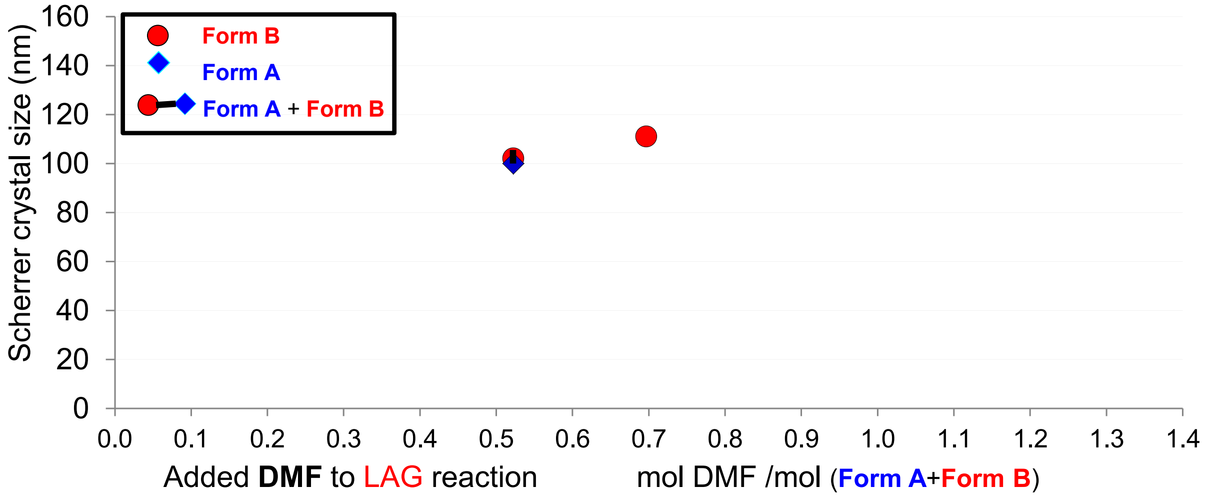

Figure S 90 Plot of Scherrer crystal size expressed as nm versus amount acetone added to LAG experiment expressed as mol DMF per mol of **1-2** (**Form A**+**Form B**).

13.3.4 Particle size determination for Form A & Form B with EtOAc as LAG solvent

Table S 125 Calculation of particle size using Scherrer equation on PXRD data using EtOAc as solvent in LAG

Scherrer crystal size (nm) for Form A and Form B												
Ball mill LAG reaction taken to thermodynamic equilibrium using Procedure 1												
2%M dbu+µL EtOAc added to 0.68 mmol DCC ([1-1]+[2-2]+[Form A]+[Form B])												
								R =	Scherrer size			
grinding time @ 30Hz to equilib.	EtOAc added to powder		1-1	2-2	Form B	Form A	[A]+[B]	[B] / [A]+[B]	Form B		Form A	
	EtOAc µL	[EtOAc]/[DCC] (mol/mol)	%M	%M	%M	%M	total polymorph	% R	crystal size (nm)	e.s.d. crystal size (nm)	crystal size (nm)	e.s.d. crystal size (nm)
3h	**10**	**0.15**	1.5	1.2	**3.3**	**94.0**	97.3	**3.4**				
2h	**18**	**0.27**	4.6	3.7	**3.8**	**87.9**	91.7	**4.1**			**78 nm**	0.9
4h	**20**	**0.30**	4.3	3.5	**3.4**	**88.8**	92.2	**3.7**				
3h30m	**22**	**0.33**	5.9	4.7	**87.7**	**1.6**	89.3	**98.2**				
3h30m	**23**	**0.34**	7.5	6.1	**47.7**	**38.8**	86.5	**55.1**				
4h	**25**	**0.37**	6.3	5.1	**52.0**	**36.6**	88.6	**58.7**				
3h	**26**	**0.39**	6.5	5.2	**63.8**	**24.5**	88.3	**72.3**				
3h	**27**	**0.40**	7.5	6.0	**72.3**	**14.1**	86.4	**83.7**				
3h	**28**	**0.42**	7.7	6.2	**79.6**	**6.4**	86.0	**92.6**	**101 nm**	1		
4h	**30**	**0.45**	6.1	4.9	**87.9**	**1.0**	88.9	**98.9**				
2h	**31**	**0.46**	5.9	4.7	**88.1**	**1.3**	89.4	**98.5**	**97 nm**	1.0		
3h	**40**	**0.60**	6.4	5.2	**86.8**	**1.6**	88.4	**98.2**				
3h	**50**	**0.75**	6.6	5.3	**87.1**	**1.1**	88.2	**98.8**				
2h30m	**60**	**0.90**	7.4	6.0	**85.0**	**1.6**	86.6	**98.2**				
2h	**70**	**1.05**	9.3	7.5	**82.0**	**1.2**	83.2	**98.6**				
[DCC] is the concentration of the dynamic covalent chemistry reaction at any time =[1-1]+[2-2]+[Form A]+[Form B]												
[1-2] = Form A + Form B [A] ≡ [Form A] [B] ≡ [Form B]												

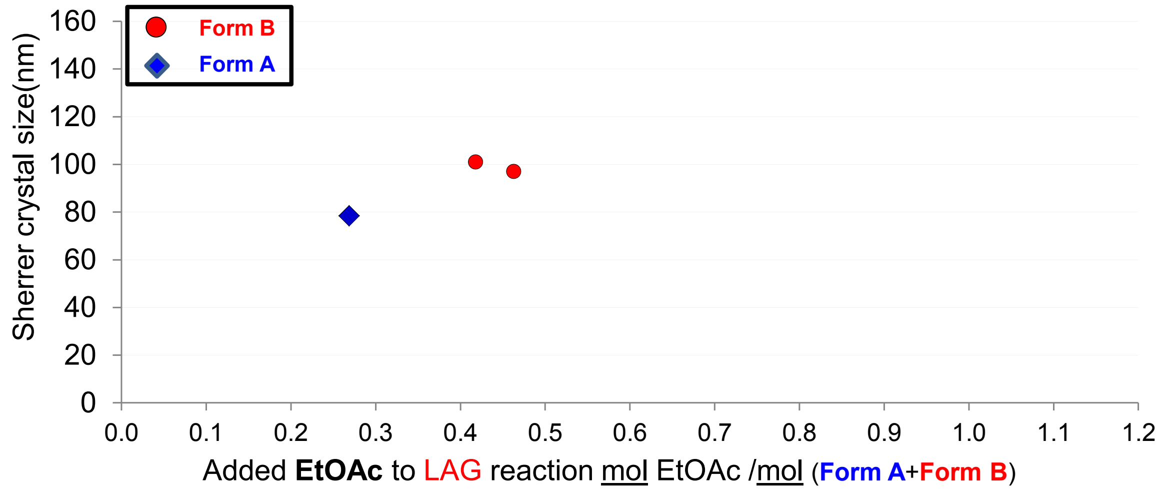

Figure S 91 Plot of Scherrer crystal size expressed as nm versus amount acetone added to LAG experiment expressed as mol EtOAc per mol of **1-2** (**Form A**+**Form B**).

13.3.5 Particle size determination for Form A & Form B with $CHCl_3$ as LAG solvent

Table S 126 Particle size determination: Scherrer equation on PXRD data using $CHCl_3$ as LAG solvent

Scherrer crystal size (nm) for **Form A** and **Form B**

Ball mill LAG reaction taken to thermodynamic equilibrium using Procedure 1

2%M dbu + μL **$CHCl_3$** added to 0.68 mmol DCC ([**1-1**]+[**2-2**]+[**Form A**]+[**Form B**])

grinding time @ 30Hz to equilib.	$CHCl_3$ added to powder: $CHCl_3$ μL	$CHCl_3$ added to powder: [$CHCl_3$]/[DCC] (mol/mol)	**1-1** %M	**2-2** %M	**Form B** %M	**Form A** %M	[A]+[B] total polymorph	R = [B]/([A]+[B]) % R	Scherrer size **Form B** crystal size (nm)	Scherrer size **Form B** e.s.d. crystal size (nm)	Scherrer size **Form A** crystal size (nm)	Scherrer size **Form A** e.s.d. crystal size (nm)
2h30m	**5**	**0.09**	0.5	0.4	**2.8**	**96.3**	99.1	**3 %**			**116 nm**	2.0
2h30m	**10**	**0.18**	0.7	0.6	**2.2**	**96.6**	98.8	**2 %**				
2h	**13**	**0.24**	0.8	0.6	**2.7**	**95.9**	98.6	**3 %**			**81 nm**	0.9
1h15m	**15**	**0.27**	3.0	2.4	**7.5**	**87.1**	94.6	**8 %**			**78 nm**	1
2h	**16**	**0.29**	1.1	0.9	**2.5**	**95.5**	98.0	**3 %**				
1h	**17**	**0.31**	3.2	2.6	**26.5**	**67.7**	94.2	**28 %**				
1h30m	**18**	**0.33**	3.0	2.4	**27.9**	**66.8**	94.7	**29 %**				
2h	**19**	**0.35**	3.5	2.8	**41.6**	**52.0**	93.6	**44 %**	**98 nm**	4	**87 nm**	3
45m	**20**	**0.36**	5.0	4.0	**89.2**	**1.9**	91.1	**98 %**	**86 nm**	1		
2h	**20**	**0.36**	3.1	2.5	**63.0**	**31.4**	94.4	**67 %**	**89 nm**	**2**	**84 nm**	3
2h	**21**	**0.38**	3.4	2.7	**44.4**	**49.5**	93.9	**47 %**	**105 nm**	**3**	**85 nm**	2
2h	**22**	**0.40**	4.2	3.4	**56.6**	**35.8**	92.4	**61 %**	**91 nm**	**3**	**77 nm**	1
2h30m	**23**	**0.42**	1.6	1.3	**67.7**	**29.4**	97.1	**70 %**	**95 nm**	**2**	**85 nm**	3
2h	**24**	**0.44**	4.9	3.9	**86.5**	**4.7**	91.2	**95 %**	**99 nm**	**6**	**81 nm**	2
2h	**25**	**0.46**	2.8	2.2	**77.8**	**17.2**	95.0	**82 %**	**95 nm**	**1**	**79 nm**	4
2h	**27**	**0.49**	3.7	3.0	**88.0**	**5.2**	93.2	**94 %**	**74 nm**			0.8
2h	**30**	**0.55**	2.9	2.4	**93.1**	**1.6**	94.7	**98 %**	**79 nm**			0.8
3h	**32**	**0.58**	4.1	3.3	**91.2**	**1.4**	92.6	**98 %**				
3h30m	**35**	**0.64**	9.5	7.7	**81.8**	**1.0**	82.8	**99 %**				
2h30m	**50**	**0.91**	14.5	11.7	**73.0**	**0.9**	73.9	**99 %**	**132 nm**	3		

[DCC] is the concentration of the dynamic covalent chemistry reaction at any time =[**1-1**]+[**2-2**]+[**Form A**]+[**Form B**]

[**1-2**] = **Form A** + **Form B** [**A**] ≡ [**Form A**] [**B**] ≡ [**Form B**]

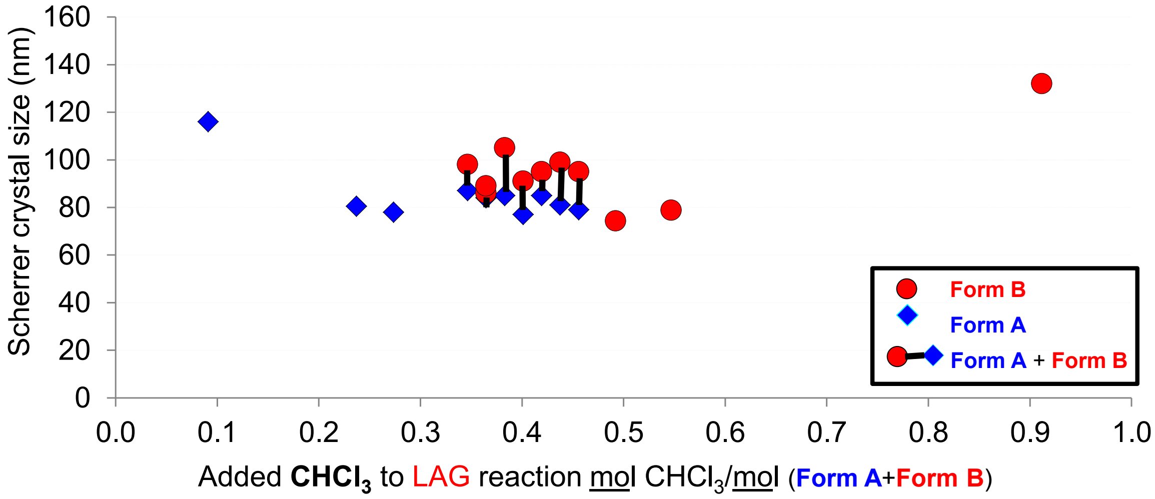

Figure S 92 Plot of Scherrer crystal size expressed as nm versus amount acetone added to LAG experiment expressed as mol $CHCl_3$ per mol of **1-2** (**Form A**+**Form B**).

13.3.6 Particle size determination for Form A & Form B with DCM as LAG solvent

Table S 127 Particle size determination: Scherrer equation on PXRD data using DCM as LAG solvent

Scherrer crystal size (nm) for Form A and Form B

Ball mill LAG reaction taken to thermodynamic equilibrium using Procedure 2

2%M dbu+µL DCM added to 0.68 mmol DCC ([1-1]+[2-2]+[Form A]+[Form B])

grinding time @ 30Hz to equilib.	DCM added to powder: DCM µL	DCM added to powder: [DCM]/[DCC] (mol/mol)	1-1 %M	2-2 %M	Form B %M	Form A %M	[A]+[B] total polymorph	R = [B]/([A]+[B]) % R	Scherrer size Form B crystal size (nm)	Scherrer size Form B e.s.d. crystal size (nm)	Scherrer size Form A crystal size (nm)	Scherrer size Form A e.s.d. crystal size (nm)
3h	**15**	**0.34**	1.1	0.9	**5.3**	**92.6**	97.9	**5 %**			**60 nm**	0.7
3h	**18**	**0.41**	1.9	1.5	**4.1**	**92.4**	96.5	**4 %**			**72 nm**	0.8
3h	**20**	**0.46**	5.3	4.3	**10.9**	**79.5**	90.4	**12 %**	**90 nm**	7	**79 nm**	1
4h	**22**	**0.50**	4.7	3.8	**8.9**	**82.6**	91.5	**10 %**	**106 nm**	11	**81 nm**	1
4h	**23**	**0.52**	4.7	3.8	**3.8**	**87.7**	91.5	**4 %**			**77 nm**	0.9
4h	**24**	**0.55**	3.7	3.0	**3.6**	**89.7**	93.3	**4 %**				
3h30m	**25**	**0.57**	6.6	5.3	**86.4**	**1.6**	88.0	**98 %**	**82 nm**	1		
4h	**26**	**0.59**	5.5	4.4	**15.9**	**74.3**	90.2	**18 %**				
4h	**27**	**0.62**	6.9	5.6	**55.9**	**31.6**	87.5	**64 %**				
4h	**28**	**0.64**	6.4	5.2	**45.8**	**42.6**	88.4	**52 %**				
4h	**30**	**0.68**	6.6	5.3	**85.9**	**2.1**	88.0	**98 %**	**70 nm**	1.0		
4h	**35**	**0.80**	8.1	6.5	**84.2**	**1.1**	85.3	**99 %**	**88 nm**	1.0		
5h	**50**	**1.14**	14.5	11.7	**73.0**	**0.7**	73.7	**99 %**				
2h	**60**	**1.37**	10.8	8.7	**79.0**	**1.6**	80.6	**98 %**	**120 nm**	4		

[DCC] is the concentration of the dynamic covalent chemistry reaction at any time =[1-1]+[2-2]+[Form A]+[Form B]

[**1-2**] = **Form A** + **Form B** [A] ≡ [**Form A**] [B] ≡ [**Form B**]

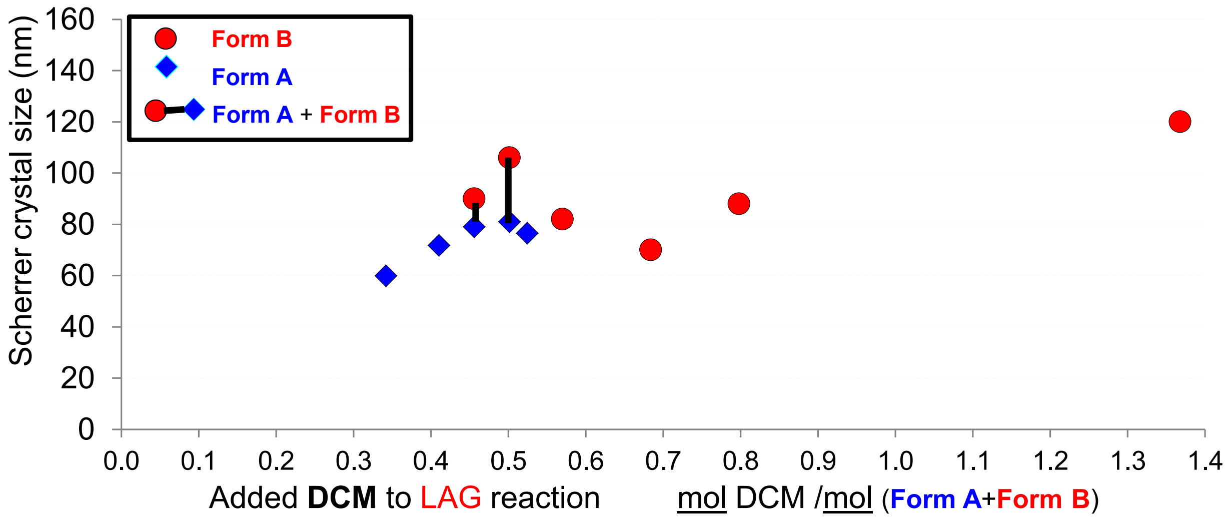

Figure S 93 Plot of Scherrer crystal size expressed as nm versus amount acetone added to LAG experiment expressed as mol DCM per mol of **1-2** (**Form A**+**Form B**).

13.3.7 Particle size determination for Form A & Form B with DMSO as LAG solvent

Table S 128 Particle size determination: Scherrer equation on PXRD data using DMSO as LAG solvent

Scherrer crystal size (nm) for Form A and Form B													
Ball mill LAG reaction taken to thermodynamic equilibrium using Procedure 4													
2%M dbu+μL DMSO added to 0.68 mmol DCC ([1-1]+[2-2]+[Form A]+[Form B])													
grinding time @ 30Hz to equilib.	DMSO added to powder		1-1	2-2	Form B	Form A	[A]+[B]	R = [B] / [A]+[B]	Scherrer size Form B		Scherrer size Form A		
	DMSO μL	[DMSO]/[DCC] (mol/mol)	%M	%M	%M	%M	total polymorph	% R	crystal size (nm)	e.s.d. crystal size (nm)	crystal size (nm)	e.s.d. crystal size (nm)	
4h	**25**	**0.52**	3.9	3.1	3.8	89.2	93.0	**4 %**			98 nm	2	
4h	**35**	**0.72**	4.0	3.2	5.0	87.8	92.8	**5 %**			92 nm	2	
4h	**37**	**0.76**	3.8	3.1	4.3	88.8	93.1	**5 %**			76 nm	2	
4.5h	**39**	**0.80**	4.7	3.8	6.3	85.2	91.5	**7 %**			81 nm	2	
5h	**41**	**0.85**	8.5	6.8	13.0	71.7	84.7	**15 %**			81 nm	2	
4h45m	**42**	**0.87**	2.6	2.0	7.4	88.0	95.4	**8 %**					
5.5h	**43**	**0.89**	9.5	7.7	75.4	7.4	82.8	**91 %**	101 nm	3			
5h15m	**44**	**0.91**	8.2	6.6	20.1	65.1	85.2	**24 %**					
5h	**45**	**0.93**	3.9	3.2	92.3	0.7	93.0	**99 %**	106 nm	2			
5.5h	**47**	**0.97**	7.1	5.8	84.4	2.8	87.2	**97 %**					

[DCC] is the concentration of the dynamic covalent chemistry reaction at any time =[1-1]+[2-2]+[Form A]+[Form B]

[**1-2**] = Form A + Form B   [A] ≡ [Form A]   [B] ≡ [Form B]

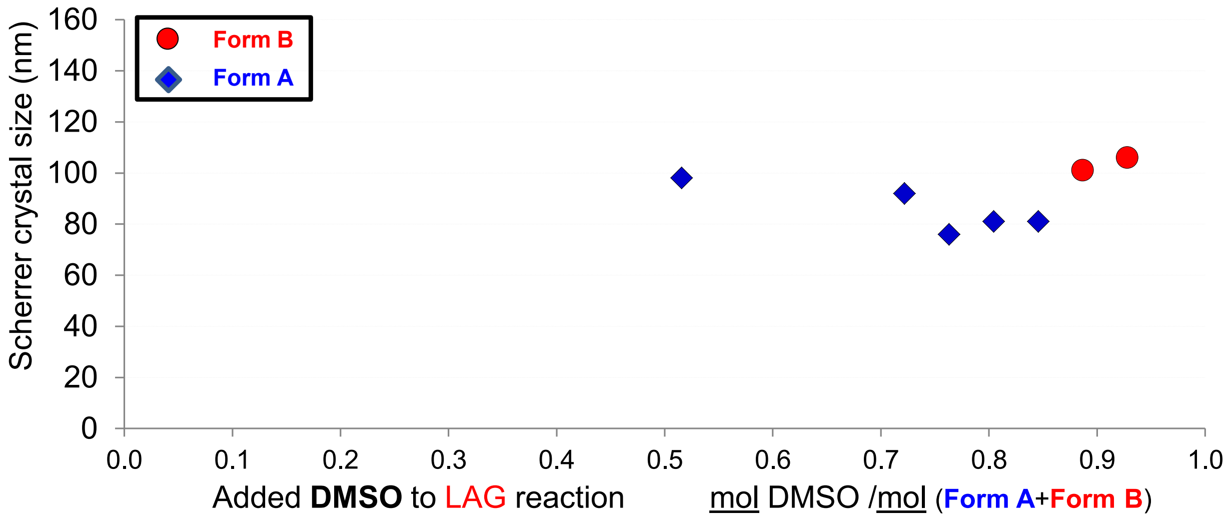

Figure S 94 Plot of Scherrer crystal size expressed as nm versus amount acetone added to LAG experiment expressed as mol DMSO per mol of **1-2** (Form A+Form B).

13.3.8 Particle size determination for Form A & Form B with MeOH as LAG solvent

Table S 129 Particle size determination: Scherrer equation on PXRD data using MeOH as LAG solvent

Scherrer crystal size (nm) for **Form A** and **Form B**

Ball mill LAG reaction taken to thermodynamic equilibrium using Procedure 3

2%M dbu+μL MeOH added to 0.68 mmol DCC ([1-1]+[2-2]+[Form A]+[Form B])

grinding time @ 30Hz to equilib.	MeOH added to powder		1-1	2-2	Form B	Form A	[A]+[B]	R = [B] / ([A]+[B])	Scherrer size Form B		Scherrer size Form A	
	MeOH μL	[MeOH]/[DCC] (mol/mol)	%M	%M	%M	%M	total polymorph	% R	crystal size (nm)	e.s.d. crystal size (nm)	crystal size (nm)	e.s.d. crystal size (nm)
4h	**25**	**0.90**	0.7	0.6	**4.2**	**94.5**	98.7	**4 %**				
5h	**50**	**1.81**	0.9	0.7	**3.4**	**95.0**	98.4	**3 %**			**68 nm**	0.8
4h	**50**	**1.81**	1.8	1.4	**3.2**	**93.6**	96.8	**3 %**			**65 nm**	1
4h30m	**60**	**2.17**	0.8	0.6	**4.4**	**94.1**	98.5	**4 %**			**69 nm**	0.9
4h20m	**63**	**2.28**	1.9	1.5	**2.9**	**93.7**	96.6	**3 %**			**65 nm**	1
5h	**64**	**2.31**	1.7	1.3	**5.8**	**91.3**	97.1	**6 %**				
5h	**65**	**2.35**	0.9	0.7	**13.8**	**84.5**	98.3	**14 %**	**115 nm**	10	**67 nm**	0.9
5h	**66**	**2.38**	1.1	0.8	**15.6**	**82.5**	98.1	**16 %**	**102 nm**	8	**68 nm**	0.9
5h	**67**	**2.42**	1.0	0.8	**42.1**	**56.1**	98.2	**43 %**	**86 nm**	2.0	**71 nm**	1.0
5h30m	**68**	**2.46**	0.8	0.6	**96.3**	**2.2**	98.5	**98 %**	**77 nm**	1.0		
5h	**69**	**2.49**	1.0	0.8	**4.3**	**93.9**	98.2	**4 %**			**68 nm**	0.9
5h	**69**	**2.49**	1.1	0.9	**5.1**	**92.8**	97.9	**5 %**			**68 nm**	0.9
4h30m	**70**	**2.53**	1.0	0.8	**96.2**	**2.0**	98.2	**98 %**	**78 nm**	1.0		
5h	**75**	**2.71**	0.8	0.7	**96.7**	**1.8**	98.5	**98 %**	**81 nm**	1.0		
5h	**80**	**2.89**	0.8	0.6	**97.5**	**1.1**	98.6	**99 %**	**81 nm**	0.9		
5h	**85**	**3.07**	0.8	0.6	**97.4**	**1.2**	98.6	**99 %**	**84 nm**	1.0		

[DCC] is the concentration of the dynamic covalent chemistry reaction at any time =[1-1]+[2-2]+[**Form A**]+[**Form B**]

[**1-2**] = **Form A** + **Form B** [A] ≡ [**Form A**] [B] ≡ [**Form B**]

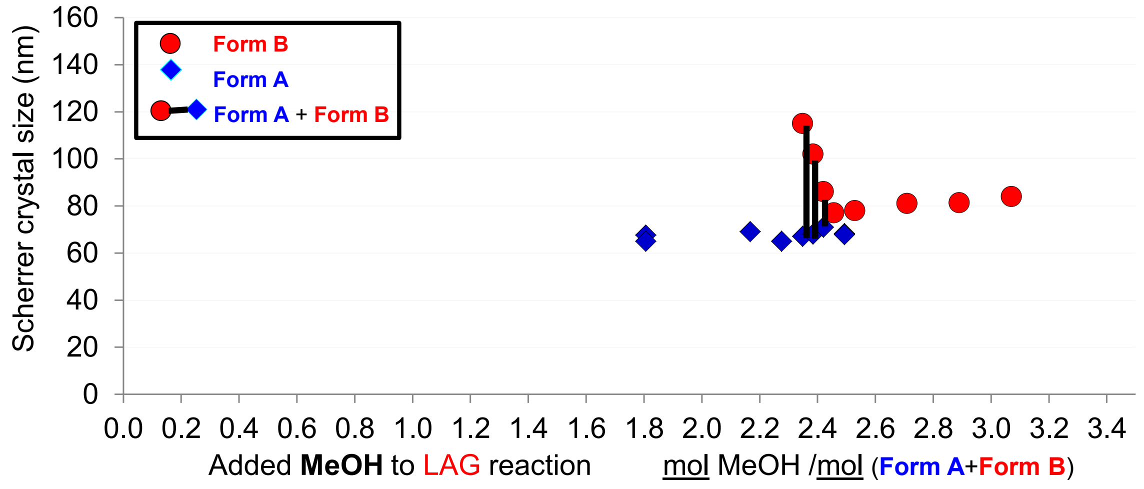

Figure S 95 Plot of Scherrer crystal size expressed as nm versus amount acetone added to LAG experiment expressed as mol MeOH per mol of **1-2** (**Form A**+**Form B**).

13.3.9 Particle size determination for Form A & Form B with EtOH as LAG solvent

Table S 130 Particle size determination: Scherrer equation on PXRD data using EtOH as LAG solvent

Scherrer crystal size (nm) for **Form A** and **Form B**

Ball mill LAG reaction taken to thermodynamic equilibrium using Procedure 4

2%M dbu+µL EtOH added to 0.68 mmol DCC ([**1-1**]+[**2-2**]+[**Form A**]+[**Form B**])

grinding time @ 30Hz to equilib.	EtOH added to powder		1-1	2-2	Form B	Form A	[A]+[B]	R = [B] / [A]+[B]	Scherrer size			
									Form B		Form A	
	EtOH µL	[EtOH]/[DCC] (mol/mol)	%M	%M	%M	%M	total polymorph	% R	crystal size (nm)	e.s.d. crystal size (nm)	crystal size (nm)	e.s.d. crystal size (nm)
3h	**50**	**1.25**	2.1	1.7	**4.3**	**91.9**	96.2	**4 %**			**65 nm**	0.9
4h	**55**	**1.38**	1.9	1.5	**4.5**	**92.1**	96.6	**5 %**			**68 nm**	0.8
4h	**60**	**1.50**	1.0	0.8	**3.5**	**94.7**	98.2	**4 %**			**67 nm**	0.8
3.5h	**64**	**1.60**	1.1	0.9	**2.8**	**95.2**	98.0	**3 %**			**68 nm**	0.8
3h20m	**65**	**1.63**	1.9	1.5	**3.8**	**92.8**	96.6	**4 %**			**76 nm**	0.8
4h20m	**66**	**1.65**	1.0	0.8	**7.6**	**90.6**	98.2	**8 %**			**62 nm**	0.8
4h	**67**	**1.68**	0.9	0.7	**71.6**	**26.9**	98.5	**73 %**	**82 nm**	2	**74 nm**	3
3h	**68**	**1.70**	1.5	1.2	**36.6**	**60.7**	97.3	**38 %**	**84 nm**	3	**67 nm**	1
5h	**69**	**1.73**	1.6	1.3	**48.0**	**49.1**	97.1	**49 %**	**71 nm**	1	**66 nm**	1
3h	**70**	**1.75**	1.2	1.0	**83.6**	**14.2**	97.8	**85 %**	**86 nm**	1	**80 nm**	5
5h20m	**71**	**1.78**	2.1	1.7	**10.6**	**85.6**	96.2	**11 %**				
4h	**72**	**1.80**	0.6	0.5	**84.2**	**14.8**	99.0	**85 %**				
4h	**73**	**1.83**	0.8	0.6	**96.7**	**1.9**	98.6	**98 %**				
4	**75**	**1.88**	1.3	1.0	**95.6**	**2.1**	97.7	**98 %**	**76 nm**	1		
4h	**77**	**1.93**	1.6	1.2	**79.1**	**18.1**	97.2	**81 %**				
4h	**80**	**2.00**	0.5	0.4	**97.2**	**1.8**	99.0	**98 %**				
8h	**90**	**2.25**	1.8	1.5	**91.1**	**5.6**	96.7	**94 %**				

[DCC] is the concentration of the dynamic covalent chemistry reaction at any time =[**1-1**]+[**2-2**]+[**Form A**]+[**Form B**]

[**1-2**] = **Form A** + **Form B** [**A**] ≡ [**Form A**] [**B**] ≡ [**Form B**]

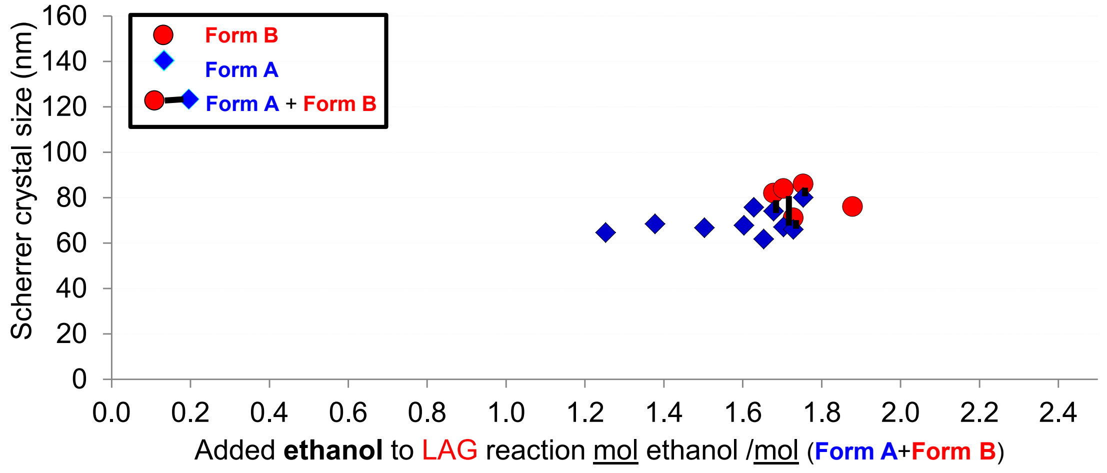

Figure S 96 Plot of Scherrer crystal size expressed as nm versus amount acetone added to LAG experiment expressed as mol EtOH per mol of **1-2** (**Form A**+**Form B**).

13.3.10 Particle size determination for Form A & Form B with IPA as LAG solvent

Table S 131 Particle size determination: Scherrer equation on PXRD data using IPA as LAG solvent

Scherrer crystal size (nm) for **Form A** and **Form B**												
Ball mill LAG reaction taken to thermodynamic equilibrium using Procedure 4												
2%M dbu+µL IPA added to 0.68 mmol DCC ([**1-1**]+[**2-2**]+[**Form A**]+[**Form B**])												
								R =	Scherrer size			
grinding time @ 30Hz to equilib.	IPA added to powder		**1-1**	**2-2**	**Form B**	**Form A**	[**A**]+[**B**]	[**B**] / [**A**]+[**B**]	Form **B**		Form **A**	
	IPA µL	[IPA]/[DCC] (mol/mol)	%M	%M	%M	%M	total polymorph	% R	crystal size (nm)	e.s.d. crystal size (nm)	crystal size (nm)	e.s.d. crystal size (nm)
1h45m	**25**	**0.48**	3.5	2.8	**2.6**	**91.1**	93.7	**3 %**				
45 m	**50**	**0.96**	1.9	1.5	**3.1**	**93.4**	96.5	**3 %**			**57 nm**	0.7
45 m	**65**	**1.24**	1.9	1.5	**4.3**	**92.3**	96.6	**4 %**			**64 nm**	0.9
45 m	**70**	**1.34**	2.0	1.6	**4.9**	**91.5**	96.4	**5 %**			**59 nm**	1
45 m	**75**	**1.43**	2.3	1.8	**3.9**	**91.9**	95.8	**4 %**			**65 nm**	0.9
45 m	**76**	**1.45**	2.0	1.6	**3.3**	**93.1**	96.4	**3 %**			**63 nm**	0.8
45 m	**77**	**1.47**	1.9	1.5	**3.6**	**92.9**	96.5	**4 %**			**76 nm**	1
45 m	**78**	**1.49**	2.2	1.8	**72.7**	**23.3**	96.0	**76 %**	**78 nm**	4	**73 nm**	4
45 m	**79**	**1.51**	2.1	1.8	**73.7**	**22.4**	96.1	**77 %**	**81 nm**	2	**72 nm**	4
45 m	**80**	**1.53**	2.6	2.1	**93.7**	**1.6**	95.3	**98 %**	**78 nm**	1		

[DCC] is the concentration of the dynamic covalent chemistry reaction at any time =[**1-1**]+[**2-2**]+[**Form A**]+[**Form B**]

[**1-2**] = **Form A** + **Form B** [**A**] ≡ [**Form A**] [**B**] ≡ [**Form B**]

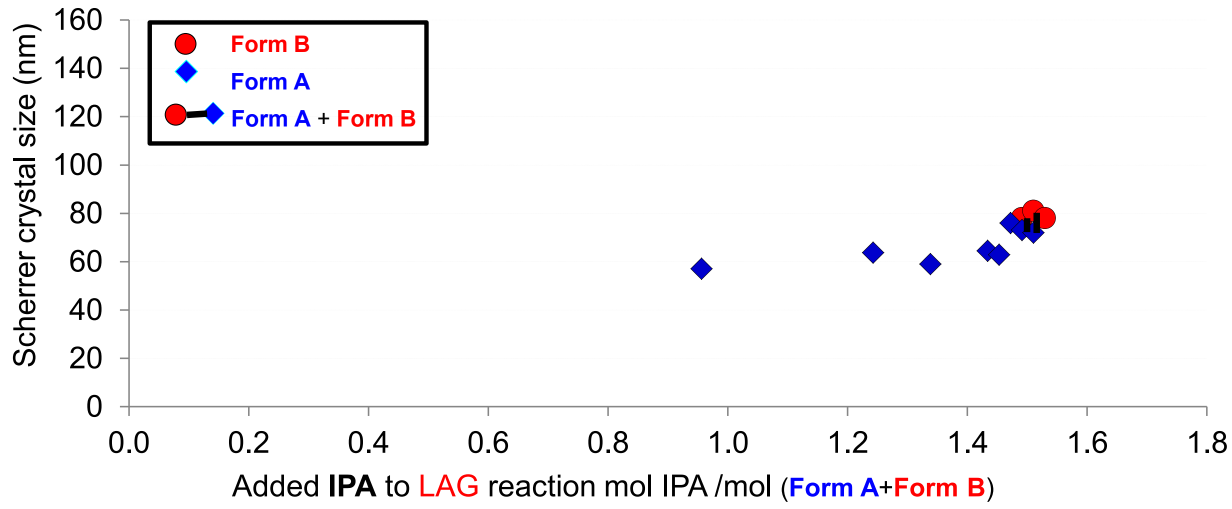

Figure S 97 Plot of Scherrer crystal size expressed as nm versus amount acetone added to LAG experiment expressed as mol IPA per mol of **1-2** (**Form A**+**Form B**).

13.3.11 Particle size determination for Form A & Form B with Water as LAG solvent

Table S 132 Particle size determination: Scherrer equation on PXRD data using Water as LAG solvent

Scherrer crystal size (nm) for **Form A** and **Form B**

Ball mill LAG reaction taken to thermodynamic equilibrium using Procedure 5

2%M dbu+µL **water** added to 0.68 mmol DCC ([**1-1**]+[**2-2**]+[**Form A**]+[**Form B**])

grinding time @ 30Hz to equilib.	water added to powder		1-1	2-2	Form B	Form A	[A]+[B]	R = [B]/([A]+[B])	Scherrer size Form B		Scherrer size Form A	
	water µL	[water]/[DCC] (mol/mol)	%M	%M	%M	%M	total polymorph	% R	crystal size (nm)	e.s.d. crystal size (nm)	crystal size (nm)	e.s.d. crystal size (nm)
1h20m	**25**	**2.03**	1.5	1.2	**5.8**	**91.5**	97.3	**6 %**			**54 nm**	0.6
1h30m	**35**	**2.85**	1.6	1.3	**4.7**	**92.5**	97.2	**5 %**			**53 nm**	0.6
3h	**50**	**4.07**	10.3	8.3	**2.8**	**78.6**	81.4	**3 %**				
2h	**70**	**5.70**	3.6	2.9	**3.7**	**89.8**	93.5	**4 %**			**49 nm**	0.5
3h	**90**	**7.33**	1.9	1.6	**3.8**	**92.7**	96.5	**4 %**			**49 nm**	0.6
3h	**100**	**8.14**	1.2	0.9	**3.5**	**94.5**	98.0	**4 %**			**49 nm**	0.8
3h	**130**	**10.58**	6.1	4.9	**2.3**	**86.7**	89.0	**3 %**			**47 nm**	0.6
3h30m	**160**	**13.02**	17.4	14.0	**3.8**	**64.7**	68.5	**6 %**				
5h	**200**	**16.28**	25.5	20.5	**1.9**	**52.1**	54.0	**4 %**				

[DCC] is the concentration of the dynamic covalent chemistry reaction at any time =[**1-1**]+[**2-2**]+[**Form A**]+[**Form B**]

[**1-2**] = **Form A** + **Form B** [**A**] ≡ [**Form A**] [**B**] ≡ [**Form B**]

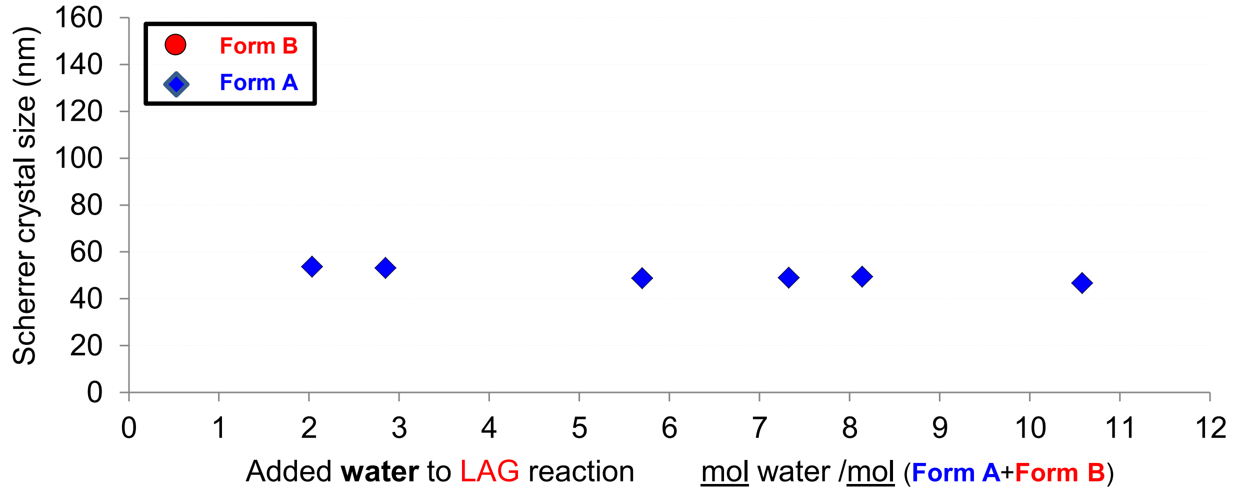

Figure S 98 Plot of Scherrer crystal size expressed as nm versus amount acetone added to LAG experiment expressed as mol Water per mol of **1-2** (**Form A**+**Form B**).

13.3.12 Particle size determination for Form A & Form B with cyclohexane as LAG solvent

Table S 133 Particle size determination: Scherrer equation on PXRD data using cyclohexane as LAG solvent

Scherrer crystal size (nm) for **Form A** and **Form B**

Ball mill LAG grinding reaction taken to thermodynamic equilibrium using Procedure 4

2%M dbu+µL **cyclohexane** added to 0.68 mmol DCC ([**1-1**]+[**2-2**]+[**Form A**]+[**Form B**])

grinding time @ 30Hz to equilib.	cyclohexane added to powder: cyclo-Hexane µL	cyclohexane added to powder: [cHexane]/[DCC] (mol/mol)	**1-1** %M	**2-2** %M	**Form B** %M	**Form A** %M	[A]+[B] total polymorph	R = [B]/([A]+[B]) % R	Scherrer size Form **B** crystal size (nm)	Scherrer size Form **B** e.s.d. crystal size (nm)	Scherrer size Form **A** crystal size (nm)	Scherrer size Form **A** e.s.d. crystal size (nm)
1h	**25**	**0.34**	1.2	0.9	**4.1**	**93.9**	98.0	**4.2**			**56**	0.8
1h10m	**35**	**0.47**	1.3	1.1	**4.3**	**93.3**	97.6	**4.4**			**57**	0.8
3h	**50**	**0.68**	1.7	1.4	**3.5**	**93.4**	96.9	**3.6**				
1h	**70**	**0.95**										
2h	**70**	**0.95**	4.2	3.4	**3.7**	**88.7**	92.4	**4.0**				
5h	**100**	**1.35**	4.2	3.4	**7.9**	**84.4**	92.3	**8.6**				
3h	**100**	**1.35**	4.5	3.6	**3.4**	**88.5**	91.9	**3.7**				
5h	**200**	**2.70**	7.1	5.7	**1.9**	**85.2**	87.1	**2.2**			**137**	2.0

[DCC] is the concentration of the dynamic covalent chemistry reaction at any time =[**1-1**]+[**2-2**]+[**Form A**]+[**Form B**]

[**1-2**] = **Form A** + **Form B** [**A**] ≡ [**Form A**] [**B**] ≡ [**Form B**]

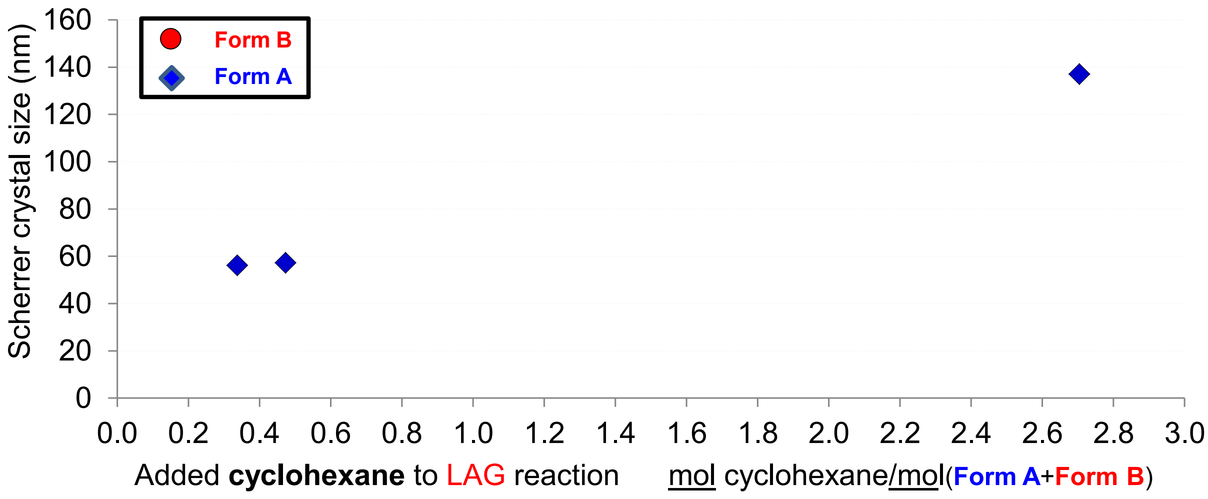

Figure S 99 Plot of Scherrer crystal size expressed as nm versus amount acetone added to LAG experiment expressed as mol cyclohexane per mol of **1-2** (**Form A**+**Form B**).

13.3.13 Particle size determination for Form A & Form B with Benzene as LAG solvent

Table S 134 Particle size determination: Scherrer equation on PXRD data using Benzene as LAG solvent

Scherrer crystal size (nm) for Form A and Form B													
Ball mill LAG reaction taken to thermodynamic equilibrium including Scherrer size using Procedure 4													
2%M dbu+µL Benzene added to 0.68 mmol DCC ([1-1]+[2-2]+[Form A]+[Form B])													
grinding time @ 30Hz to equilib.	Benzene added to powder		1-1	2-2	Form B	Form A	[A]+[B]	R = [B] / ([A]+[B])	Scherrer size Form B		Scherrer size Form A		
	Benzene µL	[Benzene]/[DCC] (mol/mol)	%M	%M	%M	%M	total polymorph	% R	crystal size (nm)	e.s.d. crystal size (nm)	crystal size (nm)	e.s.d. crystal size (nm)	
45m	**25**	**0.41**	1.4	1.1	**2.6**	**94.9**	97.5	**2.7**			**78**	2	
45m	**25**	**0.41**	5.0	4.0	**2.1**	**88.8**	90.9	**2.3**			**89**	1	
1h	**50**	**0.82**	15.7	12.6	**1.5**	**70.2**	71.7	**2.1**			**125**	3	
2h	**50**	**0.82**	14.9	12.0	**1.3**	**71.8**	73.1	**1.8**					
4h	**60**	**0.98**	20.1	16.2	**1.2**	**62.6**	63.8	**1.9**			**150**	3	

[DCC] is the concentration of the dynamic covalent chemistry reaction at any time =[1-1]+[2-2]+[Form A]+[Form B]

[1-2] = Form A + Form B [A] ≡ [Form A] [B] ≡ [Form B]

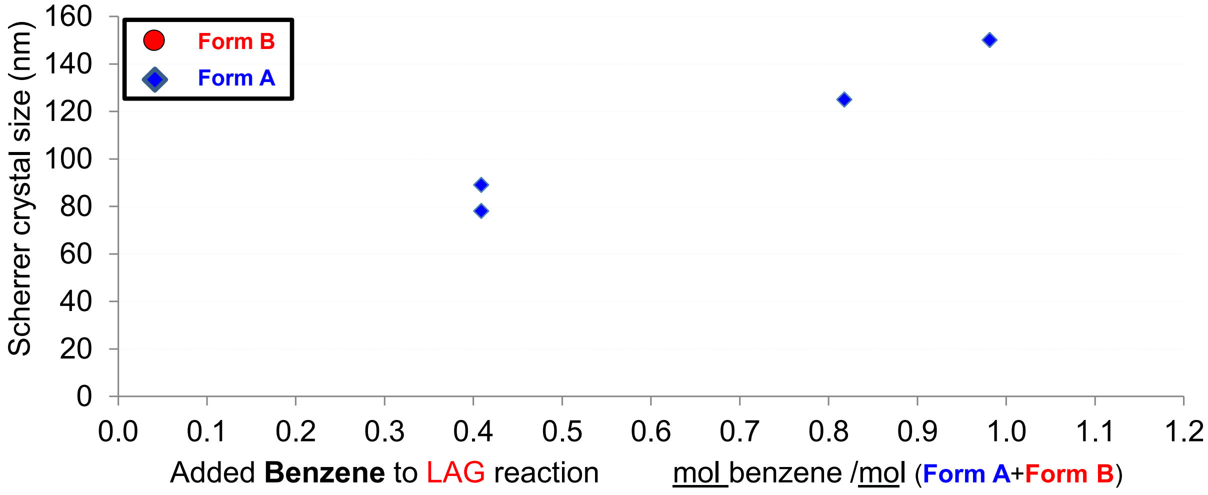

Figure S 100 Plot of Scherrer crystal size expressed as nm versus amount acetone added to LAG experiment expressed as mol Benzene per mol of **1-2** (**Form A**+**Form B**).

13.3.14 Particle size determination for Form A & Form B with Toluene as LAG solvent

Table S 135 Particle size determination: Scherrer equation on PXRD data using Toluene as LAG solvent

Scherrer crystal size (nm) for **Form A** and **Form B**

Ball mill LAG reaction taken to thermodynamic equilibrium including Scherrer size using Procedure 4

2%M dbu+μL Toluene added to 0.68 mmol DCC ([**1-1**]+[**2-2**]+[**Form A**]+[**Form B**])

grinding time @ 30Hz to equilib.	Toluene added to powder: Toluene μL	Toluene added to powder: [Toluene]/[DCC] (mol/mol)	1-1 %M	2-2 %M	Form B %M	Form A %M	[A]+[B] total polymorph	R = [B]/([A]+[B]) % R	Scherrer size Form B crystal size (nm)	Scherrer size Form B e.s.d. crystal size (nm)	Scherrer size Form A crystal size (nm)	Scherrer size Form A e.s.d. crystal size (nm)
1h	**15**	**0.21**	1.0	0.8	**2.3**	**95.9**	98.2	**2.3**			**81**	1
1h	**18**	**0.25**	2.9	2.3	**3.3**	**91.5**	94.8	**3.5**			**79**	1
1h20m	**20**	**0.28**	2.3	1.9	**2.9**	**92.9**	95.8	**3.0**			**80**	1
1h	**25**	**0.34**	3.4	2.8	**2.5**	**91.3**	93.8	**2.7**			**85**	1
1h20m	**30**	**0.41**	3.3	2.7	**3.7**	**90.3**	94.0	**3.9**			**93**	2
2h	**50**	**0.69**	15.6	12.5	**2.7**	**69.3**	72.0	**3.8**				
4h	**60**	**0.83**	18.0	14.5	**2.2**	**65.3**	67.5	**3.3**			**135**	3

[DCC] is the concentration of the dynamic covalent chemistry reaction at any time =[**1-1**]+[**2-2**]+[**Form A**]+[**Form B**]

[**1-2**] = **Form A** + **Form B** [**A**] ≡ [**Form A**] [**B**] ≡ [**Form B**]

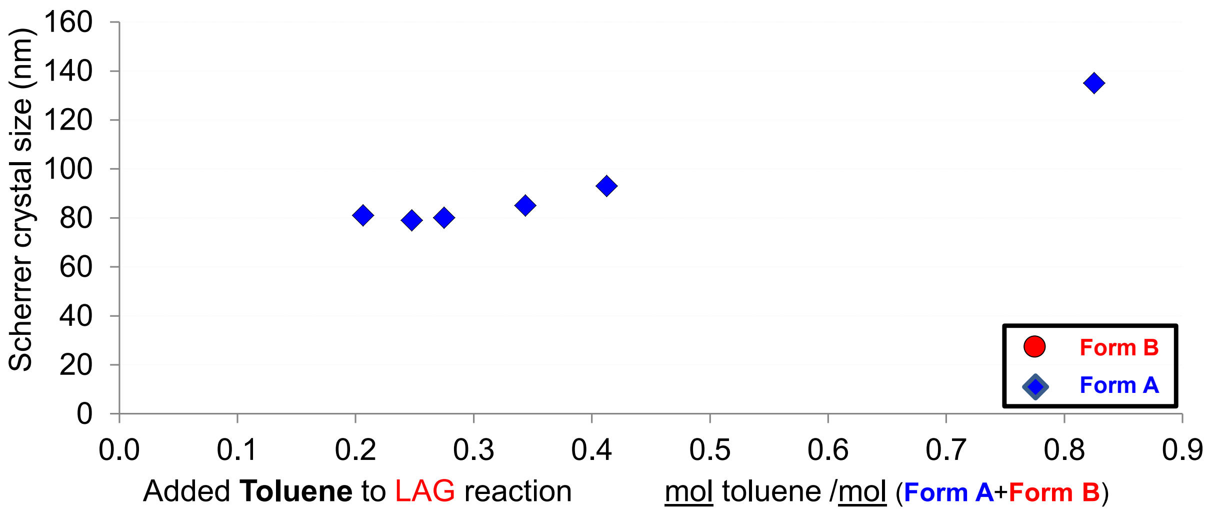

Figure S 101 Plot of Scherrer crystal size expressed as nm versus amount acetone added to LAG experiment expressed as mol Toluene per mol of **1-2** (**Form A**+**Form B**).

13.4 Conclusion of particle size determination by Scherrer equation of PXRD scans

Scherrer size conclusions

All particles have an average crystal size smaller than 150nm and in most cases in the order of tens of nm. All measurements were made ex-situ, and it is reasonable to assume that the actual equilibrium size under milling conditions will be equal or smaller than the one registered ex-situ. It is important to remember that the error in accuracy is larger for larger Scherrer size, with a possible bias towards larger calculated size. There is a general tendency of **form A** particles to be smaller than form B particles. We believe that the solvent has to play a role on the rates of growth of **form A** and **form B** particles. An interesting detail is that **Form A** seems to have larger crystal size in the turnover experiments in the absence of **dbu** catalyst than when the catalyst is present (see sections 13.1 and 13.2). This could be due to the way that the milled sample is recovered from the jar at the end of the experiment: since the sample is very static, some drops of IPA are used to help with the powder recovery. In a few experiments for which no IPA was used for the sample recovery, the size ranges from 45 to 56nm - the same range as for the experiments in the presence of catalyst.

We have computational evidence that the particle size has an effect on the polymorph relative stabilities. On the other hand the non infinite slope **R** index versus solvent concentration equilibrium curves gives experimental evidence that the surface solvation has to play a role on the relative stabilities too. It is difficult to separate these two effects. We are currently investigating this issue.

14 Thermodynamic experiments with 1:1 Theophilline: Benzamide cocrystals

14.1 Introduction

We want here to demonstrate that polymorph cocrystal formation and polymorph cocrystal interconversion by ball mill grinding is driven by thermodynamics. To this aim, we will be using **Form I** and **Form II** of the 1:1 theophylline and benzamide (tp and bzm) cocrystals as prepared by Fisher et al.[26] As in the example of the disulfide previously discussed, preparation of the cocrystals of 1:1 tp:bzm by ball mill grinding can lead to the formation of different polymorphs, these being the thermodynamically most stable products depending on the experimental conditions. Under ball mill LAG conditions, we will demonstrate that Form II is the stable polymorph while Form I is the metastable polymorph. Similarly, under ball mill NG conditions, Form I is the stable polymorph while Form II is the metastable polymorph.

Section 14.2 demonstrates that **Form I** is the stable polymorph formed from equimolar amounts of the conformers **tp** and **bzm**, under ball mill NG once equilibrium is achieved (>35 minutes) under the given experimental conditions (14.5mL stainless steel grinding jar, two 10 mm ID stainless steel ball bearings, 1g payload of total powder and grinding at 30Hz). The same polymorph is obtained if we use ball mill LAG with 250 μL cyclohexane (apolar solvent). Similarly, Section 14.3 demonstrates that **Form II** is the stable polymorph formed from equimolar amounts of the conformers **tp** and **bzm**, under ball mill LAG conditions once equilibrium is achieved (>25 minutes) using the same grinding jars, ball bearing, payload and grinding frequency as with the ball mill NG experiments. LAG can be achieved with 250μL of various polar solvents such as water, ethanol, acetonitrile or acetone as suggested by Fisher et all.[26]

The easiest way to demonstrate whether Form I or Form II is the thermodynamic product by ball mill grinding under given experimental conditions is to use under these ball mill grinding experimental conditions an equimolar mixture of Form I and Form II. The metastable polymorph will be consumed and transformed to the stable polymorph on ball mill grinding once equilibrium is achieved, obtaining only the stable polymorph at equilibrium in a quantitative yield.

Section 14.4 demonstrates that **Form I** is the stable polymorph under ball mill NG while Section 14.5 demonstrates that **Form II** is the stable polymorph under ball mill LAG (250μL water). We also demonstrate in Section 14.4 and Section 14.5 for ball mill NG and ball mill LAG, respectively that we can grind in a similar experimental setup as with the disulfide chemistry (14.5mL stainless steel grinding jar, two 7 mm ID stainless steel ball bearings, 200 mg payload of total powder and grinding at 30Hz). While the kinetics is different to the 1g payload and two 10 mm ID ball bearing, the thermodynamic products are the same: **Form I** is the stable form under ball mill NG and **Form II** is the stable form under ball mill LAG with polar solvents. For LAG we use 50μL water or MeCN. We show in Section 14.6 and 14.7 for ball mill LAG and ball mill NG respectively, that polymorph conversion follows a nucleation kinetic event to reach thermodynamic equilibrium. In Section 14.6, starting with **Form I** under ball mill LAG with polar solvents, **Form I** remains unchanged up to 2 minutes, between 2 and before 5 minutes it follows a sigmoidal nucleation step. After 5 minutes only the stable polymorph, **Form II**, is formed in quantitative yield. Similarly, in Section 14.7 starting with **Form II** under ball mill NG, **Form II** remains unchanged probably for the first 10 minutes, followed by a sigmoidal nucleation step. After 15 minutes and clearly after 30 minutes only the stable polymorph, **Form I**, is formed in quantitative yield. Section 14.8 shows the turnover experiments of polymorph interconversion. Starting from **Form I**, under ball mill LAG (250μL water) it is transformed after 15 minutes to the stable polymorph **Form II**. After drying **Form II** and subjecting it to ball mill NG, **Form I** is obtained on achieving equilibrium after 4 hours grinding. This turnover of polymorph interconversion is repeated 5 times, demonstrating that the thermodynamic form depends only on the experimental condition of ball mill grinding.

Section 14.9 demonstrates that under LAG conditions, different thermodynamic outcomes can be achieved depending on the volume of solvent used for the 1g payload with two 10 mm ID ball bearings. When starting from **Form I**, it remains unchanged when adding up to 9 μL water. **Form II** starts getting

formed after adding 10 μL and 11 μL water, the thermodynamic product being a mixture of **Form I** and **Form II**. After the addition of 12 μL and all the way up to 250 μL water, **Form II** is obtained in quantitative yield. Section 14.10 uses slurry experiments to demonstrate that **Form II** is the thermodynamic polymorph at ambient conditions. In this case we use water, but any other solvent will lead to the same thermodynamic outcome, however the kinetics is expected to be different.

14.2 Cocrystals of 1:1 Theophilline: Benzamide: preparation of Form I

Objective of this experiment:

To find a procedure to reproducibly prepare Form I from an equimolar mixture of **tp** and **bzm** by ball mill NG. Demonstrate that **Form I** is the thermodynamic form under the ball mill neat grinding experimental conditions used.

Table S 136 Demonstration of the homogeneity of the ball mill NG experiment to equilibrium of a 1g payload of equimolar mixture of the coformers tp and bzm when using two 10 mm ID ball bearings. The phase composition of the product is obtained by Rietveld refinement of the PXRD scan.

Preparation of 1:1 Theophylline:Benzamide cocrystal **Form I**									
Sample homogeneity									
**Ball mill grinding** experimental conditions				**Starting coformers** weighing (mg)		**Reaction product of ball mill neat grinding** Phase composition			
ss balls	Payload	solvent μL	grinding time @30Hz	**tp 3.3 mmol 598mg**	**bzm 3.3 mmol 402mg**	**Form I %M**	e.s.d. mol%	**Form II %M**	e.s.d. mol%
2*10mm ID	1g	**Neat**	**25 min**	**598.00**	**401.78**				
Sample from **location 1** from 1 g of product inside same grinding jar						91.5	*0.6*	8.5	*0.6*
Sample from **location 2** from 1 g of product inside same grinding jar						91.3	*0.5*	8.7	*0.5*
Sample from **location 3** from 1 g of product inside same grinding jar						99.5	*0.2*	0.5	*0.2*
Sample from **location 4** from 1 g of product inside same grinding jar						99.5	*0.3*	0.5	*0.3*
Sample from **location 5** from 1 g of product inside same grinding jar						99.5	*0.2*	0.5	*0.2*
**CONCLUSION:**					**Average**	**96.3**	0.4	3.7	0.4
Sample is very homogenous					**%RSD**	**4.6**		119	

Table S 137: Preparation of cocrystal Form I by ball mill NG and by LAG with apolar solvents (250 uLcyclohexane) of an equimolar mixture tp and bzm at 1g payload when using two 10 mm ID ball bearings. The phase composition of the product is obtained by Rietveld refinement of the PXRD scan.

Preparation of 1:1 Theophylline:Benzamide cocrystal **Form I**									
**Ball mill grinding** experimental conditions				**Starting coformers** weighing (mg)		**Reaction product of ball mill neat grinding** Phase composition			
ss balls	Payload	solvent μL	grinding time @30Hz	**tp 3.3 mmol 598mg**	**bzm 3.3 mmol 402mg**	**Form I %M**	e.s.d. mol%	**Form II %M**	e.s.d. mol%
2*10mm ID	1g	**Neat**	**25 min**	**598.00**	**401.78**	**96.3**	*0.36*	3.7	0.36
2*10mm ID	1g	**Neat**	**35 min**	**598.07**	**402.02**	**97.4**	0.4	2.6	*0.4*
2*10mm ID	1g	**Neat**	**35 min**	**598.25**	**402.04**	**97.1**	0.5	2.9	*0.5*
2*10mm ID	1g	Neat	**8x60 min**	**598.12**	**401.99**	**97.2**	0.5	2.8	0.5
2*10mm ID	1g	250μL cHexane	**8x60 min**	**598.03**	**402.02**	**99.3**	0.2	0.7	0.2
2*10mm ID	1g	Neat	**35 min**	**597.97**	**402.01**	**96.8**	0.4	3.2	0.4
2*10mm ID	1g	Neat	**35 min**	**598.22**	**401.84**	**96.9**	0.5	3.1	0.5
2*10mm ID	1g	Neat	**35 min**	**599.91**	**401.95**	**97.4**	0.6	2.6	0.6

Preparation of **Form I** cocrystal of 1:1 Theophylline: Benzamide

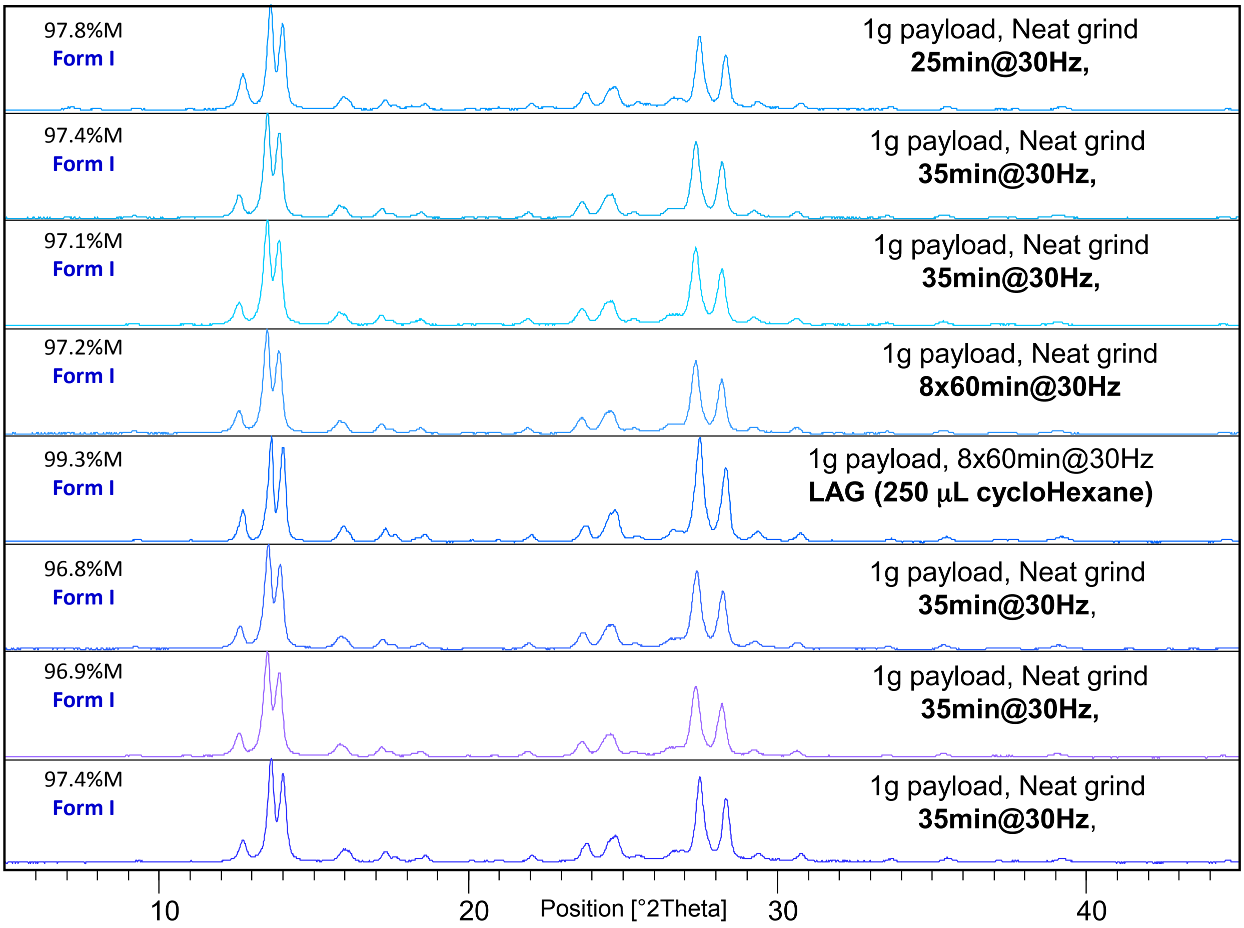

PXRD scan 53: Product of the ball mill NG or ball mill LAG with apolar solvents (cyclohexane) of equimolar mixture of theophylline and benzamide. Experimental conditions are documented in Table S1376.

<u>Conclusion of this experiment:</u>

Form I is the thermodynamic product by ball mill NG of equimolar mixture of tp and bzm when grinding to equilibrium (>35 minutes). The sample is homogeneous. The same outcome is achieved when using ball mill LAG with apolar solvents such as 250 µL cyclohexane.

14.3 Cocrystals of 1:1 Theophilline: Benzamide. Preparation of Form II

Objective of this experiment:

To find a procedure to reproducibly prepare **Form II** from an equimolar mixture of tp and bzm by ball mill LAG. Demonstrate that **Form II** is the thermodynamic form under the ball mill grinding experimental conditions used.

Table S 138 Demonstration of the homogeneity of the ball mill LAG experiment to equilibrium of a 1g payload of equimolar mixture of the coformers tp and bzm using two 10 mm ID ball bearings. The phase composition of the product is obtained by Rietveld refinement of the PXRD scan.

Preparation of 1:1 Theophylline:Benzamide cocrystal **Form II**									
Homogeneity of ball mill grinding sample									
**Ball mill grinding** experimental conditions				**Starting coformers** weighing (mg)		**Reaction product of ball mill LAG** Phase composition			
ss balls	Payload	solvent µL	grinding time @30Hz	**tp 3.3 mmol 598mg**	**bzm 3.3 mmol 402mg**	**Form I %M**	e.s.d. mol%	**Form II %M**	e.s.d. mol%
2*10mm ID	1g	**250µL H_2O**	**25 min**	**598.05**	**402.15**				
Sample from **location 1** from 1 g of product inside same grinding jar						1.4	0.3	98.6	0.3
Sample from **location 2** from 1 g of product inside same grinding jar						0.4	0.2	99.6	0.2
Sample from **location 3** from 1 g of product inside same grinding jar						1.7	0.3	98.3	0.3
Sample from **location 4** from 1 g of product inside same grinding jar						2.2	0.3	97.8	0.3
Sample from **location 5** from 1 g of product inside same grinding jar						0.7	0.2	99.3	0.2
**CONCLUSION:**					**Average**	1.3	0.3	**98.7**	**0.3**
Sample is very homogenous					**%RSD**	57		0.7	

Table S 139 Preparation of cocrystal **Form II** by ball mill LAG with 250 µL polar solvents such as water, EtOH, MeCN or acetone of an equimolar mixture tp and bzm at 1g payload using two 10 mm ID ball bearings. The phase composition of the product is obtained by Rietveld refinement of the PXRD scan.

Preparation of 1:1 Theophylline:Benzamide cocrystal **Form II**									
**Ball mill grinding** experimental conditions				**Starting coformers** weighing (mg)		**Reaction product of ball mill LAG** Phase composition			
ss balls	Payload	solvent µL	grinding time @30Hz	**tp 3.3 mmol 598mg**	**bzm 3.3 mmol 402mg**	**Form I %M**	e.s.d. mol%	**Form II %M**	e.s.d. mol%
2*10mm ID	1g	**250µL H_2O**	**25 min**	**598.05**	**402.15**	1.3	0.3	**98.7**	0.26
2*10mm ID	1g	**250µL H_2O**	**25 min**	**598.02**	**402.24**	0	0.2	**100.0**	0.2
2*10mm ID	1g	**250µL H_2O**	**25 min**	**598.32**	**402.67**	1.4	0.3	**98.6**	0.3
2*10mm ID	1g	**250µLMeCN**	**3x60 min**	**598.12**	**401.62**	2.3	0.3	**97.7**	0.3
2*10mm ID	1g	**250µLEtOH**	**3x60 min**	**598.13**	**402.09**	0.4	0.2	**99.6**	0.2
2*10mm ID	1g	**250µL Acetone**	**25 min**	**598.30**	**402.16**	0.2	0.1	**99.8**	0.1
2*10mm ID	1g	**250µL Acetone**	**25 min**	**598.30**	**402.16**	0	0.5	**100.0**	0.5
2*10mm ID	1g	**250µL Acetone**	**25 min**	**598.47**	**401.92**	0.7	0.1	**99.3**	0.1

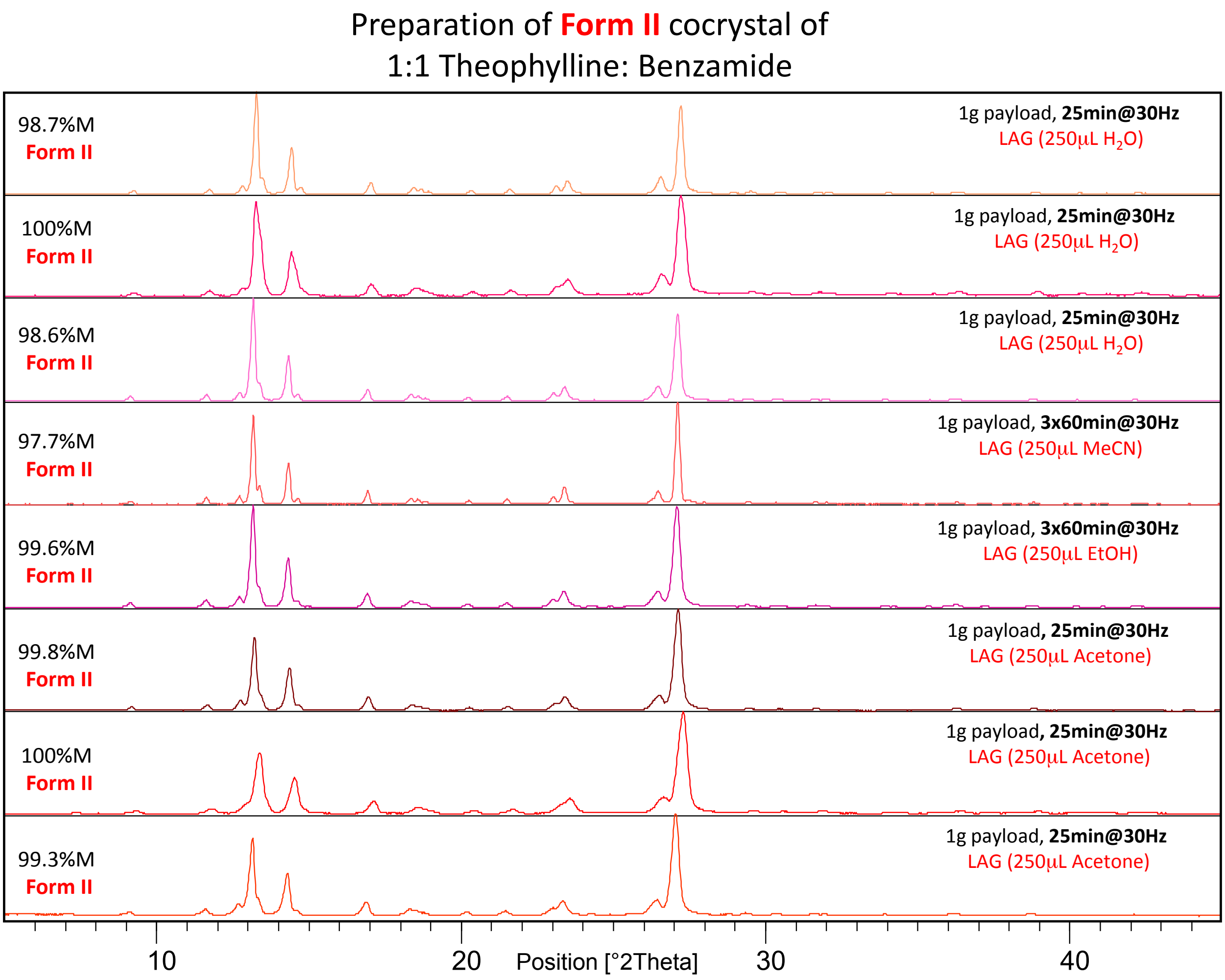

PXRD scan 54 PXRD of cocrystal **Form II** obtained by LAG with polar solvents (water, EtOH, MeCN, Acetone) an equimolar mixture of theophylline and benzamide at 1g payload.

<u>Conclusion of this experiment:</u>

Form II is the thermodynamic product by ball mill LAG of equimolar mixture of tp and bzm when grinding to equilibrium (>25 minutes) using 250 μL of polar solvents (water, MeCN, EtOH and acetone). The samples from ball mill LAG are homogenous.

14.4 Determining which of the cocrystals of 1:1 tp:bzm, Form I or Form II, is the thermodynamic product under ball mill neat grinding conditions

Objective of this experiment:

To determine whether **Form I** or **Form II** of the 1:1 tp:bzm cocrystals is the thermodynamic product under ball mill NG conditions at equilibrium.

An easy way to determine whether **Form I** or **Form II** is the thermodynamic product under ball mill NG conditions, is to perform this experiment with a 1:1 mixture of **Form I** : **Form II**. The metastable cocrystal will be transformed to the stable cocrystal under these experimental conditions. At equilibrium only the thermodynamic cocrystal form will remain.

Table S 140 Thermodynamic product under ball mill NG conditions of 1:1 tp:bzm cocrystals: **Form I** or **Form II** (1g and 200 mg payload). The phase composition of the product is obtained by Rietveld refinement of the PXRD scan.

Ball mill neat grinding of 1:1 Form I : Form II of (1:1 tp:bzm cocrystals)

**Ball mill grinding** experimental conditions				**Starting cocrystals** weighing (mg)			**Reaction product of ball mill neat grinding** Phase composition			
ss balls	Payload	solvent µL	grinding time @30Hz	**Form I 500mg**	**Form II 500mg**		**Form I %M**	e.s.d. mol%	**Form II %M**	e.s.d. mol%
2*10mm ID	1g	**Neat**	**15 min**	500.42	500.26	→	**99.3**	0.2	0.7	0.2
2*10mm ID	1g	**Neat**	**150 min**	500.06	500.26	→	**97.8**	0.3	2.2	0.3
2*10mm ID	1g	**Neat**	**24x60 min**	500.17	500.40	→	**97.9**	0.3	2.1	0.3
ss balls	Payload	solvent µL	grinding time @30Hz	**Form I 100mg**	**Form II 100mg**		**Form I %M**	e.s.d. mol%	**Form II %M**	e.s.d. mol%
**2*7mm ID**	**200mg**	**Neat**	**15 min**	99.93	100.25	→	**96.4**	0.4	3.6	0.4
**2*7mm ID**	**200mg**	**Neat**	**42.5 min**	100.13	99.89	→	**95.6**	0.8	4.4	0.8
**2*7mm ID**	**200mg**	**Neat**	**150 min**	100.04	100.40	→	**98.8**	0.2	1.2	0.2

Determining which Form of 1:1 tp:bzm cocrystal is the thermodynamic product under **ball mill neat grinding** condition (1g payload, 2x10mm ss balls)

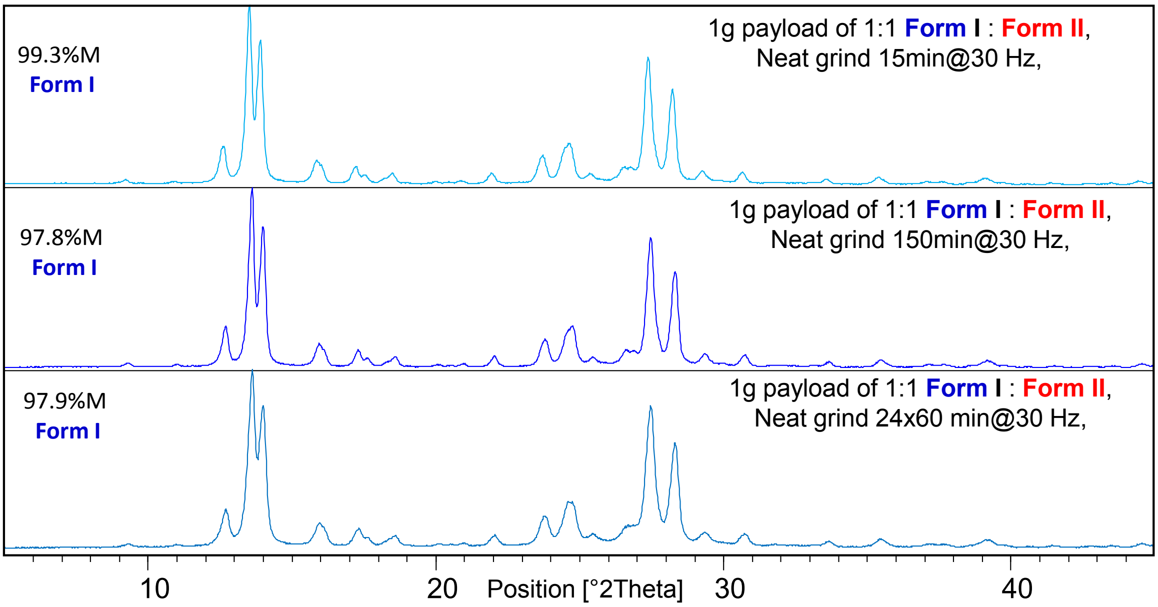

Determining which Form of 1:1 tp:bzm cocrystal is the thermodynamic product under **ball mill neat grinding** condition (200mg payload, 2x7mm ss balls)

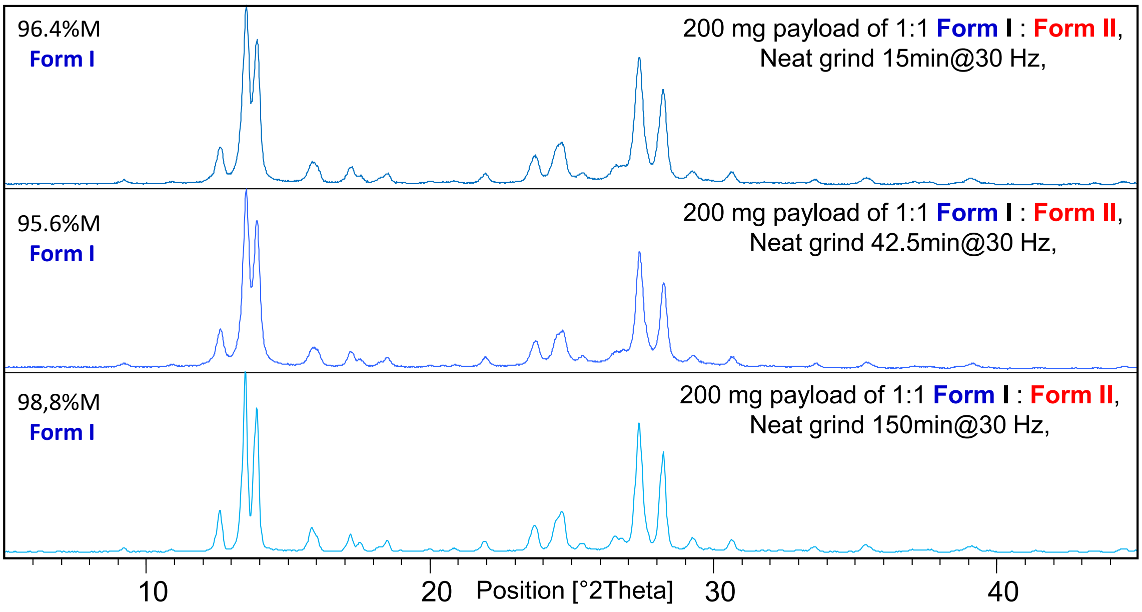

PXRD scan 55 PXRD scans of the product of ball mill NG of an equimolar mixture of **Form I** and **Form II** taken to equilibrium under the following experimental setup; top) 14.5mL stainless steel grinding jar, two 10 mm ID stainless steel ball bearings, 1g payload of total powder and grinding at 30Hz and bottom) 14.5mL stainless steel jar, two 7 mm ID stainless steel jar ball bearings, 200 mg payload of total powder and grinding at 30Hz.

Conclusion of this experiment:

Form I is the thermodynamic product under ball mill neat grinding conditions at equilibrium. **Form II** is the metastable form. Equilibrium for ball mill neat grinding when using 1:1 mixture of **Form I** : **Form II** is achieved before 15 minutes for a payload of 1 g and grinding @ 30Hz with two 10 mm ID stainless steel ball bearing. For a payload of 200 mg and grinding @ 30Hz with two 7 mm ID stainless steel ball bearing, equilibrium is achieved before 15 minutes.

14.5 Determining which of the cocrystals of 1:1 tp:bzm, Form I or Form II, is the thermodynamic product under ball mill LAG conditions with polar solvents

Objective of this experiment:

An easy way to determine which of the cocrystals of 1:1 tp:bzm is the thermodynamic product under ball mill LAG conditions , we performed this experiment with a 1:1 mixture of **Form I** : **Form II**. The metastable product will be transformed to the stable form, therefore at equilibrium only the thermodynamic form remains.

Table S 141 Thermodynamic product under ball mill LAG conditions of 1:1 tp:bzm cocrystals: **Form I** or **Form II** (1g and 200 mg payload). The phase composition of the product is obtained by Rietveld refinement of the PXRD scan.

Ball mill LAG of 1:1 Form I : Form II of (1:1 tp:bzm cocrystals)

**Ball mill grinding** experimental conditions				**Starting cocrystals** weighing (mg)			**Reaction product of ball mill LAG** Phase composition			
ss balls	Payload	solvent µL	grinding time @30Hz	**Form I 500mg**	**Form II 500mg**		**Form I %M**	e.s.d. mol%	**Form II %M**	e.s.d. mol%
2*10mm ID	1g	250µL H_2O	1 min	500.26	500.56	→	0.3	0.1	**99.7**	0.1
2*10mm ID	1g	250µL H_2O	150 min	500.37	500.32	→	0	0.2	**100**	0.2
2*10mm ID	1g	250µL H_2O	24x60min	500.04	500.31	→	0.3	0.2	**99.7**	0.2
ss balls	Payload	solvent µL	grinding time @30Hz	**Form I 100mg**	**Form II 100mg**		**Form I %M**	e.s.d. mol%	**Form II %M**	e.s.d. mol%
2*7mm ID	200mg	50µL H_2O	15 min	100.22	100.02	→	consistent with quantitative **Form II**			
2*7mm ID	200mg	50µL H_2O	150 min	100.36	100.1	→	3.3	0.4	**96.7**	0.4
2*7mm ID	200mg	50µL MeCN	15 min	100.13	100.52	→	1.5	0.2	**98.5**	0.2

a) Determining which Form of 1:1 tp:bzm cocrystal is the thermodynamic product under **ball mill LAG** conditions (1 g payload, 2x10mm ss balls)

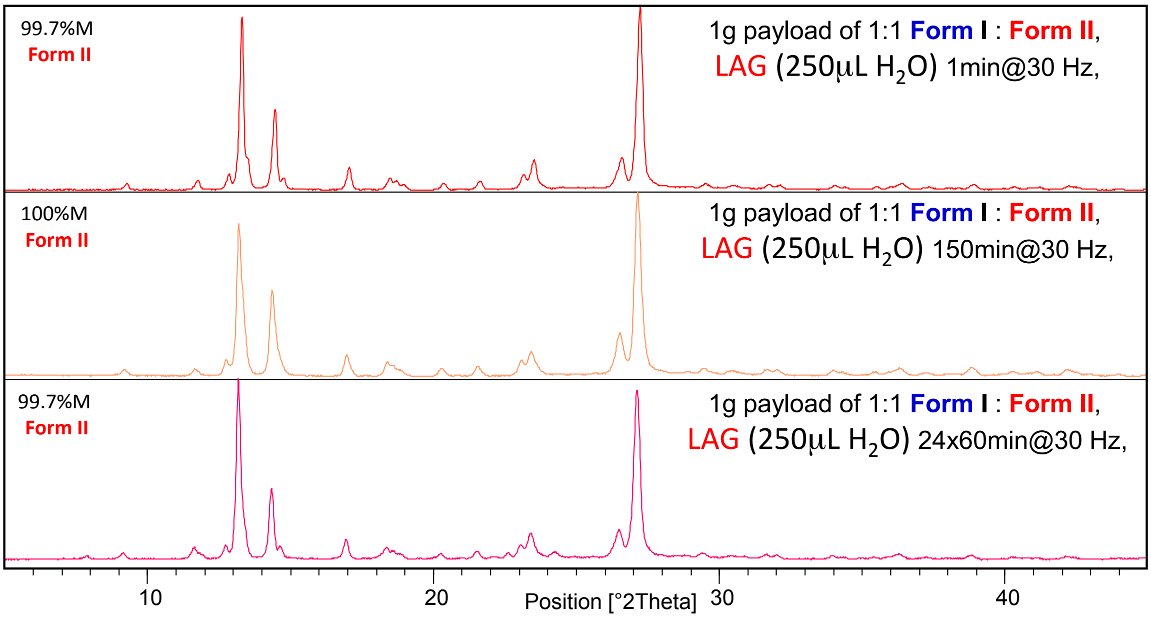

b) Determining which Form of 1:1 tp:bzm cocrystal is the thermodynamic product under **ball mill LAG** conditions (200 mg payload, 2x7mm ss balls)

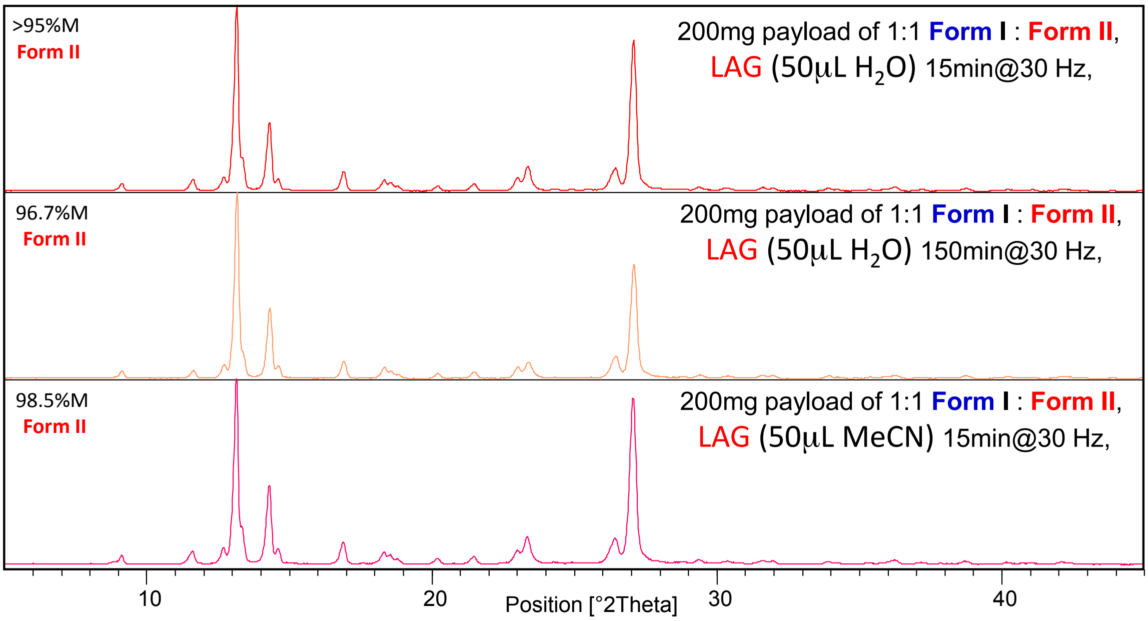

PXRD scan 56 PXRD scans of the product of ball mill LAG of an equimolar mixture of **Form I** and **Form II** taken to equilibrium under the following experimental setup; a) 14.5mL stainless steel grinding jar, two 10 mm ID stainless steel ball bearing, 1g payload of total powder, 250 µL polar solvent and grinding at 30Hz and b) 14.5mL stainless steel grinding jar, two 7 mm ID stainless steel ball bearing, 200 mg payload of total powder, 50 µL polar solvent and grinding at 30Hz.

<u>Conclusion of this experiment:</u>

Form II is the thermodynamic product under ball mill LAG to equilibrium. **Form I** is the metastable form. Equilibrium for ball mill LAG is achieved after just 1 minute for a payload of 1 g and grinding @ 30Hz with two 10 mm ID stainless steel ball bearings. For a payload of 200 mg and grinding @ 30Hz with two 7 mm ID stainless steel ball bearing, equilibrium is achieved before 15 minutes.

14.6 Polymorph transformation of Form I to Form II under ball mill LAG conditions

Objective of this experiment:

Kinetic study of ball mill LAG (250 μL water) to equilibrium of 1g of **Form I** cocrystal of 1:1 tp:bzm .

Table S 142: Kinetic study: Transformation of 1g of **Form I** to **Form II** under ball mill LAG conditions with 250 μL of water. The phase composition of the starting material and the product is obtained by Rietveld refinement of the PXRD scan.

Kinetic study: polymorph transformation **Form I** to **Form II** under **ball mill LAG** (250 μL water)

Preparation **Form I** (starting material) from 1:1 tp:bzm (1g payload) 35 min@30Hz	**Starting Material Form I** Phase composition					Starting Material **Form I** **+ 250 μL H_2O**	**Reaction product of ball mill LAG** Phase composition			
LAG Solvent	**Form I %M**	e.s.d. mol%	**Form II %M**	e.s.d. mol%		grinding time @30Hz	**Form I %M**	e.s.d. mol%	**Form II %M**	e.s.d. mol%
**Neat**	**99.3**	0.3	0.7	0.3	→	**1 min**	**98.4**	0.3	1.6	0.3
**Neat**	**98.3**	0.4	1.7	0.4	→	**3 min**	**66.1**	0.5	**33.9**	0.5
**Neat**	**96.8**	0.4	3.2	0.4	→	**5 min**	0.0	0.2	**100.0**	0.2
**Neat**	**96.9**	0.5	3.1	0.5	→	**10 min**	0.4	0.2	**99.6**	0.2

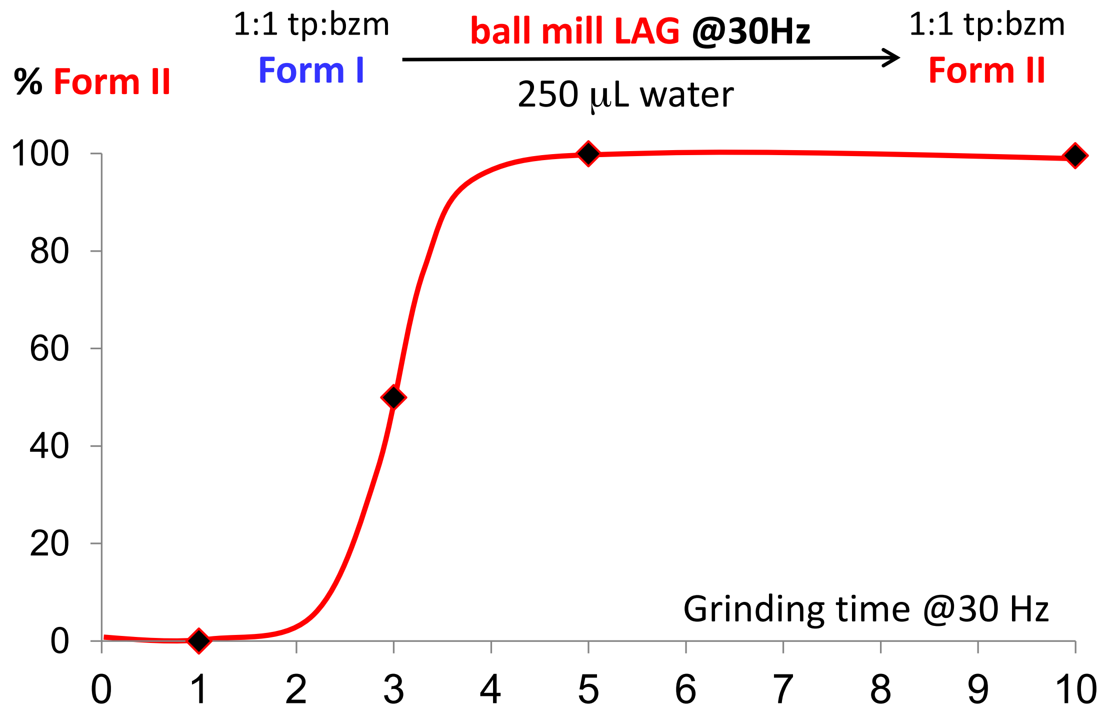

Figure S 102 Kinetic graph of the thermodynamic driven polymorph interconversion starting from **Form I** under ball mill LAG conditions. No fitting was performed and the curves drawn are only a guide to the eye.

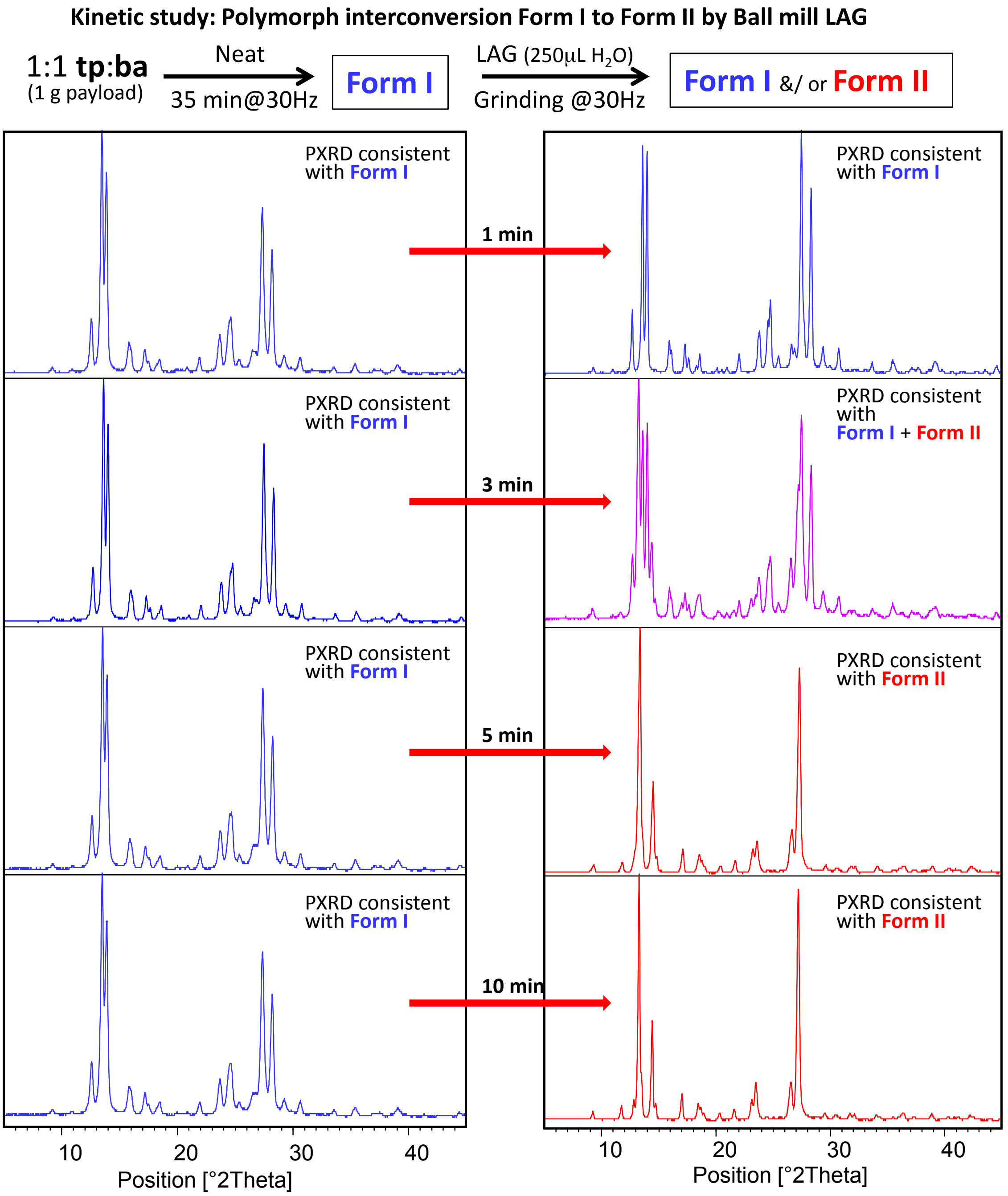

PXRD scan 57 Polymorph transformation of 1 g of **Form I** (PXRD scans shown on left) by ball mill LAG @ 30 Hz with 250μL water at different grinding times. The PXRD on the right shows the kinetic and/or thermodynamic products of the thermodynamic driven polymorph transformation to the stable **Form II**.

Conclusion of this experiment:

Form II is the thermodynamic product while **Form I** is the metastable polymorph under ball mill LAG conditions to equilibrium under the experimental conditions used (250μL water). Equilibrium is achieved between 3 and 5 minutes. After 5 minutes only **Form II** is formed. Before 1 minutes, **Form I** is unaltered: the polymorph transformation by ball mill neat grinding presents a delay period of around 1-2 minutes followed by a sigmoidal curve. .

14.7 Polymorph transformation of Form II to Form I under ball mill neat grinding conditions

Objective of this experiment:

Kinetic study of ball mill neat grinding to equilibrium of 1g of **Form II** cocrystal of 1:1 tp:bzm .

Table S 143 Kinetic study of in-situ polymorph transformation from **Form II** to **Form I** under ball mill neat grinding conditions. The phase composition of the starting material and the product is obtained by Rietveld refinement of the PXRD scan.

Kinetic study: polymorph transformation **Form II** to **Form I** under **ball mill neat grinding**										
Preparation **Form II** (starting material) from 1:1 tp:bzm (1g payload) 25 min@30Hz	**Starting Material Form II** Phase composition					Starting Material **Form II** is dried before polymorph transformation	**Reaction product of ball mill neat grinding** Phase composition			
LAG Solvent	**Form I** %M	e.s.d. mol%	**Form II** %M	e.s.d. mol%		grinding time @30Hz	**Form I** %M	e.s.d. mol%	**Form II** %M	e.s.d. mol%
**250µL Acetone**	0.7	0.2	**99.3**	0.2	→	**10 min**	**17.5**	0.5	**82.5**	0.5
**250µL Acetone**	0.4	0.1	**99.6**	0.1	→	**12 min**	**89.6**	0.3	10.4	0.3
**250µL H_2O**	0.4	0.2	**99.6**	0.2	→	**15 min**	**89.6**	0.3	10.4	0.3
**250µL H_2O**	0.0	0.2	**100.0**	0.2	→	**30 min**	**97.0**	0.3	3	0.3
**250µL Acetone**	0	0.5	**100.0**	0.5	→	**60 min**	**99.2**	0.2	0.8	0.2
**250µL Acetone**	0.2	0.1	**99.8**	0.1	→	**120 min**	**97.4**	0.3	2.6	0.3
**250µL Acetone**	0.7	0.1	**99.3**	0.1	→	**180 min**	**97.2**	0.4	2.8	0.4
**250µL EtOH**	0.4	0.2	**99.6**	0.2	→	**240 min**	**98.5**	0.3	1.5	0.3

Note: Form II can be prepared from different polar solvents. It is only important to make sure that the powder is totally dry before performing the experiment. When using water, the sample has to be dried overnight.

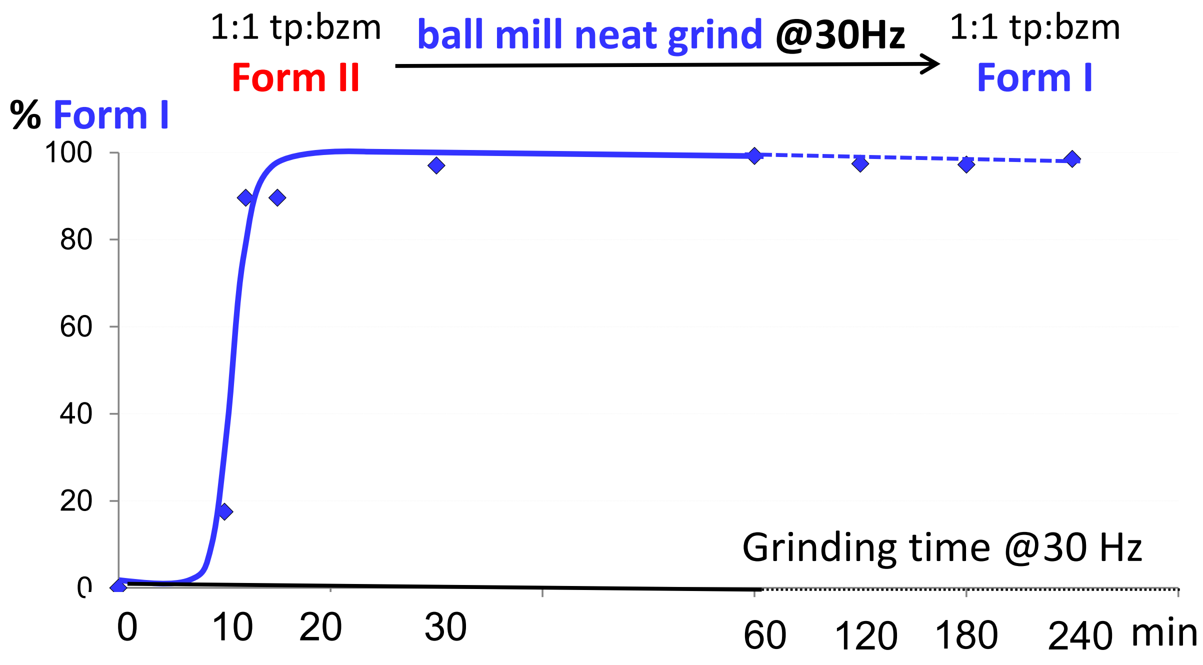

Figure S 103 Kinetic graph of the thermodynamic driven polymorph interconversion starting with **Form II** under ball mill NG conditions. No fitting was performed and the curves drawn are only a guide to the eye.

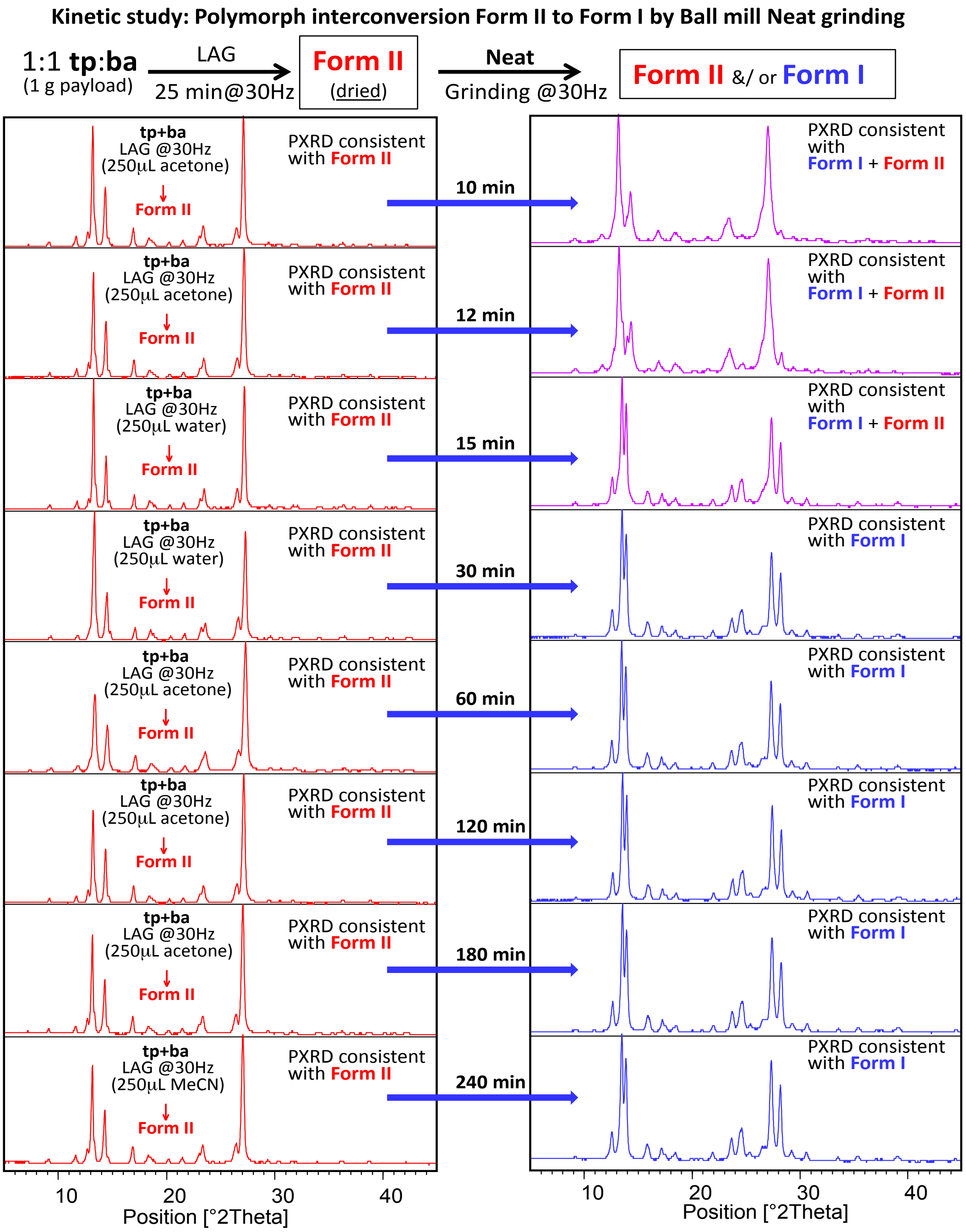

PXRD scan 58 Polymorph transformation of 1 g of dried **Form II** (PXRD scans shown on left) by ball mill NG @ 30 Hz. The PXRD on the right shows the kinetic and /or thermodynamic products of the thermodynamic driven polymorph transformation to the stable **Form I**.

Conclusion of this experiment:

Form I is the thermodynamic product while **Form II** is the metastable polymorph under ball mill neat grinding conditions to equilibrium under the experimental conditions used. Equilibrium is achieved between 15 and 30 minutes. After 30 minutes only **Form I** is formed. Before 10 minutes, **Form II** is unaltered: the polymorph transformation by ball mill neat grinding presents a delay period of around 10 minutes followed by a sigmoidal curve. .

14.8 Turnover experiments: polymorph interconversion between Form I and Form II cocrystals of 1:1 tp:bzm by ball mill grinding

Objective of this experiment:

To demonstrate that **Form I** is always formed as the thermodynamic product under ball mill neat grinding conditions when grinding is taken to equilibrium. Equally to demonstrate that **Form II** is always formed as the thermodynamic product under ball mill LAG conditions (using in this case 250μL water as the LAG solvent for 1 g payload) when grinding is taken to equilibrium.

Table S 144; Experimental conditions for the turnover experiment of polymorph interconversion starting from **Form I** of 1:1 tp:bzm demonstrating thermodynamic driving force. The phase composition of the starting material and the product is obtained by Rietveld refinement of the PXRD scan.

Turnover experiment of polymorph interconversion by ball mill grinding between **Form I** and **Form II** cocrystals of 1:1 tp:bzm

**Ball mill grinding** experimental conditions				**Starting cocrystals**			**Reaction product** Phase composition			
transformation	turnover	solvent μL	grinding time @30Hz	**Form I 1g**	**Form II 1g**		**Form I %M**	e.s.d. mol%	**Form II %M**	e.s.d. mol%
**Form II --> Form I**	1	**Neat**	**4x60 min**		dried *in-situ*	→	**97.4**	0.3	2.6	0.3
**Form I --> Form II**	1	**250μL H_2O**	**15 min**	*in-situ*		→	0.6	0.2	**99.4**	0.2
**Form II --> Form I**	2	**Neat**	**4x60 min**		dried *in-situ*	→	**96.8**	0.6	3.2	0.6
**Form I --> Form II**	2	**250μL H_2O**	**15 min**	*in-situ*		→	1.1	0.2	**98.9**	0.2
**Form II --> Form I**	3	**Neat**	**4x60 min**		dried *in-situ*	→	**99.4**	0.3	0.6	0.3
**Form I --> Form II**	3	**250μL H_2O**	**15 min**	*in-situ*		→	3.7	0.4	**96.3**	0.4
**Form II --> Form I**	4	**Neat**	**4x60 min**		dried *in-situ*	→	**97.2**	0.5	2.8	0.5
**Form I --> Form II**	4	**250μL H_2O**	**15 min**	*in-situ*		→	1.4	0.3	**98.6**	0.3
**Form II --> Form I**	5	**Neat**	**4x60 min**		dried *in-situ*	→	**99.5**	0.3	0.5	0.3
**Form I --> Form II**	5	**250μL H_2O**	**15 min**	*in-situ*		→	0.7	0.2	**99.3**	0.2

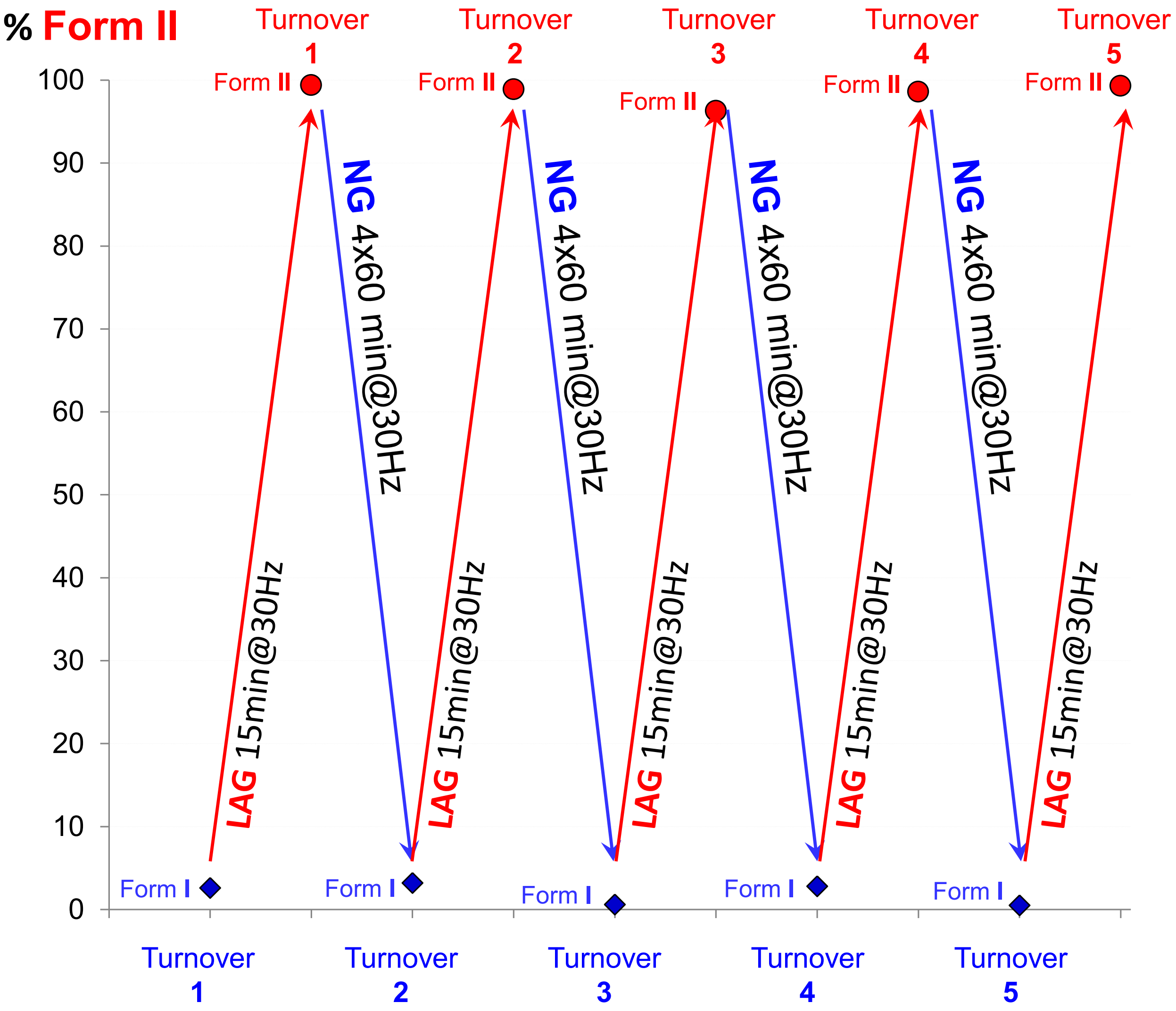

Figure S 104 Polymorph interconversion starting from **Form I** cocrystal prepared from 1:1 tp:bzm under ball mill NG (35 minutes at Hz) . **Form I** is transformed to **Form II** by LAG (250μL water, 15 minutes@30Hz). **Form II** is allowed to dry overnight; dried **Form II** is transformed to **Form I** by ball mill NG 4x60 minutes@30Hz). 4 additional turnover experiments are performed with the same material.

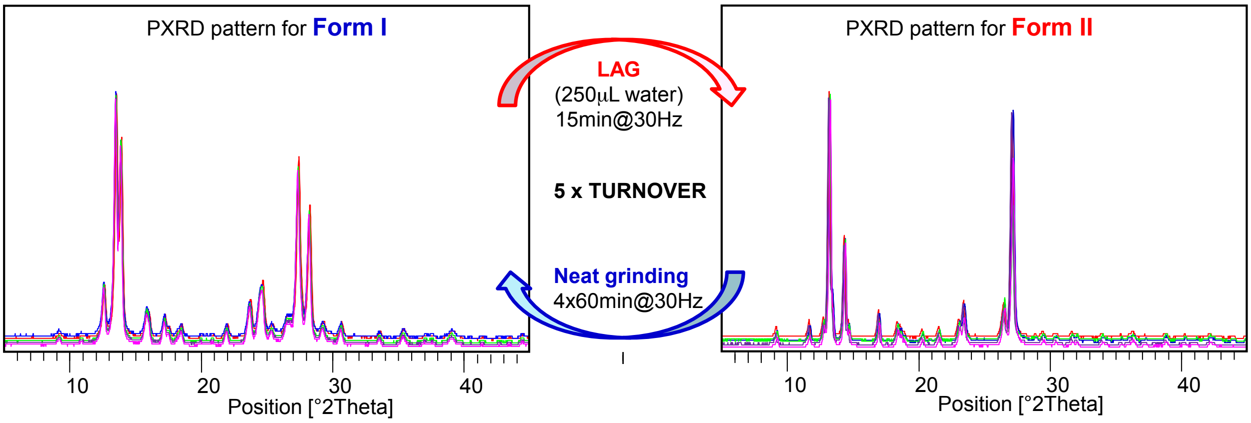

Figure S 105 On the left: superimposed PXRD scans of **Form I** prepared by ball mill NG of dried **Form II**; on the right : superimposed PXRD scans of **Form II** formed by ball mill LAG of Form I with 250 µl water.

Cocrystals o f 1:1 Theophylline : Benzamide

in situ polymorph interconversion between **Form I** and **Form II**

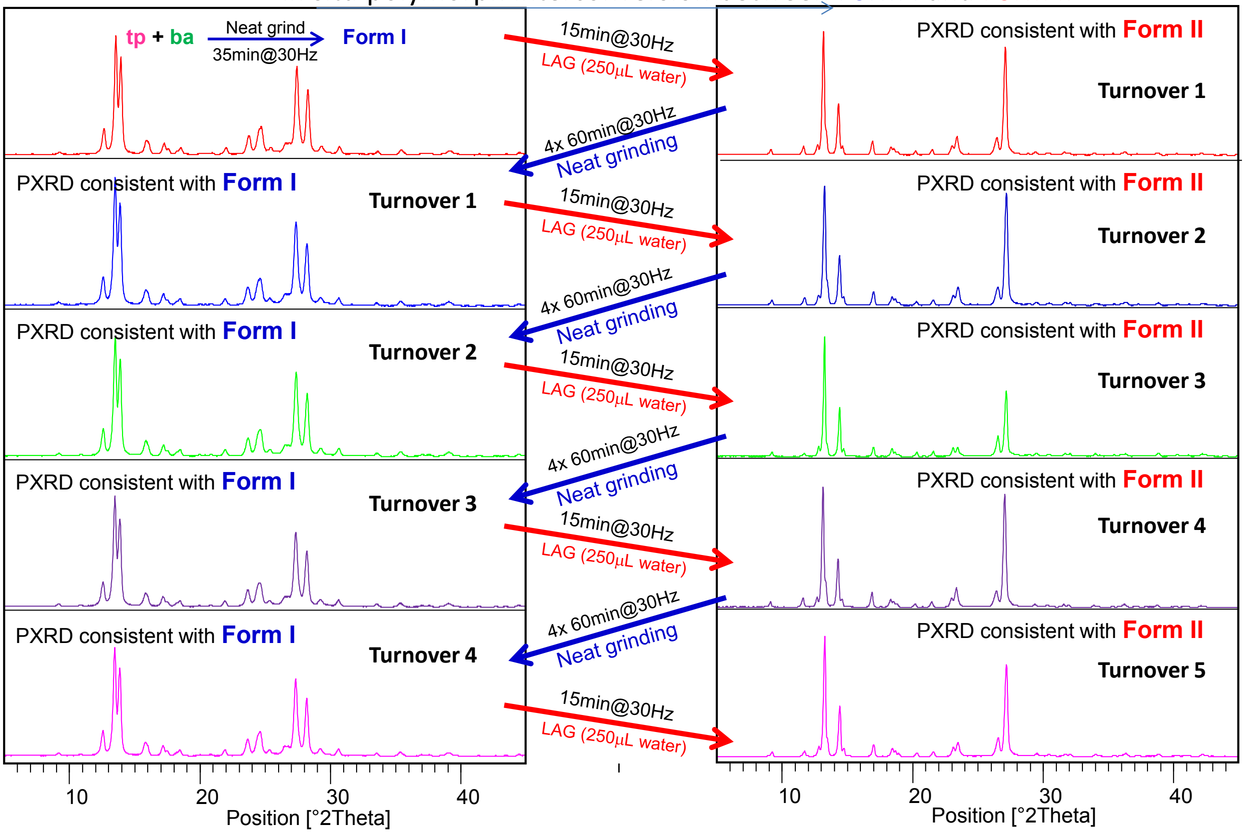

PXRD scan 59 Turnover experiments of polymorph interconversions between **Form I** and **Form II** of the 1:1 theophylline: benzamide cocrystals. The starting point is the formation of **Form I** cocrystal by 35 minutes at 30 Hz neat grinding from equimolar amount of the coformers Theophylline and Benzamide making a total weight of 1g. For neat grinding the grinding jar containing **Form II** was left open overnight in the fumehood to remove any traces of water.

Conclusion of this experiment:

Ball mill NG to equilibrium with a 14.5 mL stainless steel grinding jar and two 10 mm ID stainless steel ball bearings, **Form I** is the thermodynamic product while **Form II** is the metastable polymorph. Therefore dry powder of **Form II** will be transformed to **Form I** under ball mill neat grinding to equilibrium. Equally, **Form II** is the thermodynamic product while **Form I** is the metastable polymorph under ball mill LAG conditions (250 µL water) to equilibrium. Therefore **Form I** will be transformed to **Form II** under ball mill LAG conditions to equilibrium). In conclusion, **Form II** can be transformed to **Form I** under ball mill neat grinding and **Form I** can be transformed back to **Form II** under ball mill LAG; this transformation can be taken through many turnover experiments with the same starting material.

14.9 Thermodynamic study: ball mill LAG conditions taken to equilibrium to 1g of Form I with increasing volume of water as LAG solvent.

Objective of this experiment:

To investigate how the volume of solvent under ball mill LAG taken to equilibrium conditions affects the composition of **Form I** and **Form II**.

Table S 145; left columns: experimental conditions & phase composition of the starting material **Form I**; right columns: experimental conditions and thermodynamic product of ball mill LAG of **Form I**. The phase composition of the starting material and the product is obtained by Rietveld refinement of the PXRD scan.

Thermodynamic study: polymorph transformation **Form I** to **Form II** under **ball mill LAG** with increasing μL water

Preparation **Form I** (starting material) from 1:1 tp:bzm (1g payload) 35 min@30Hz	Starting Material **Form I** Phase composition					**Ball mill LAG 4x15 min@30Hz)** Starting Material **Form I**		**Reaction product of ball mill LAG** Phase composition			
LAG Solvent	**Form I %M**	e.s.d. mol%	**Form II %M**	e.s.d. mol%		water added μL	water added mmol	**Form I %M**	e.s.d. mol%	**Form II %M**	e.s.d. mol%
**Neat**	**97.7**	0.3	**2.3**	0.3	→	5 μL	0.3mmol	**99.2**	0.1	**0.8**	0.1
**Neat**	**97.9**	0.4	**2.1**	0.4	→	9 μL	0.5mmol	**98.6**	0.2	**1.4**	0.2
**Neat**	**97.9**	0.4	**2.1**	0.4	→	10 μL	0.6mmol	**16.5**	0.3	**83.5**	0.3
**Neat**	**99.5**	0.2	**0.5**	0.2	→	10 μL	0.6mmol	**97.8**	0.2	**2.2**	0.2
**Neat**	**98.3**	0.4	**1.7**	0.4	→	10 μL	0.6mmol	**98.7**	0.2	**1.3**	0.2
**Neat**	**97.8**	0.4	**2.2**	0.4	→	11 μL	0.6mmol	**88.5**	0.3	**11.5**	0.3
**Neat**	**99.4**	0.2	**0.6**	0.2	→	11 μL	0.6mmol	**77.8**	0.3	**22.2**	0.3
**Neat**	**99.5**	0.2	**0.5**	0.2	→	12 μL	0.7mmol	**0.5**	0.1	**99.5**	0.1
**Neat**	**97.5**	0.4	**2.5**	0.4	→	12 μL	0.7mmol	**0.2**	0.1	**99.8**	0.1
**Neat**	**96.6**	0.4	**3.4**	0.4	→	15 μL	0.8mmol	**0.4**	0.1	**99.6**	0.1
**Neat**	**97.1**	0.4	**2.9**	0.4	→	30 μL	1.7mmol	**0.8**	0.1	**99.2**	0.1
**Neat**	**97.4**	0.4	**2.6**	0.4	→	50 μL	2.8mmol	**1.3**	0.2	**98.7**	0.2
**Neat**	**97.6**	0.4	**2.4**	0.4	→	100 μL	5.6mmol	**0.5**	0.1	**99.5**	0.1
**Neat**	**97.4**	0.3	**2.6**	0.3	→	250 μL	13.9mmol	**0.6**	0.2	**99.4**	0.2

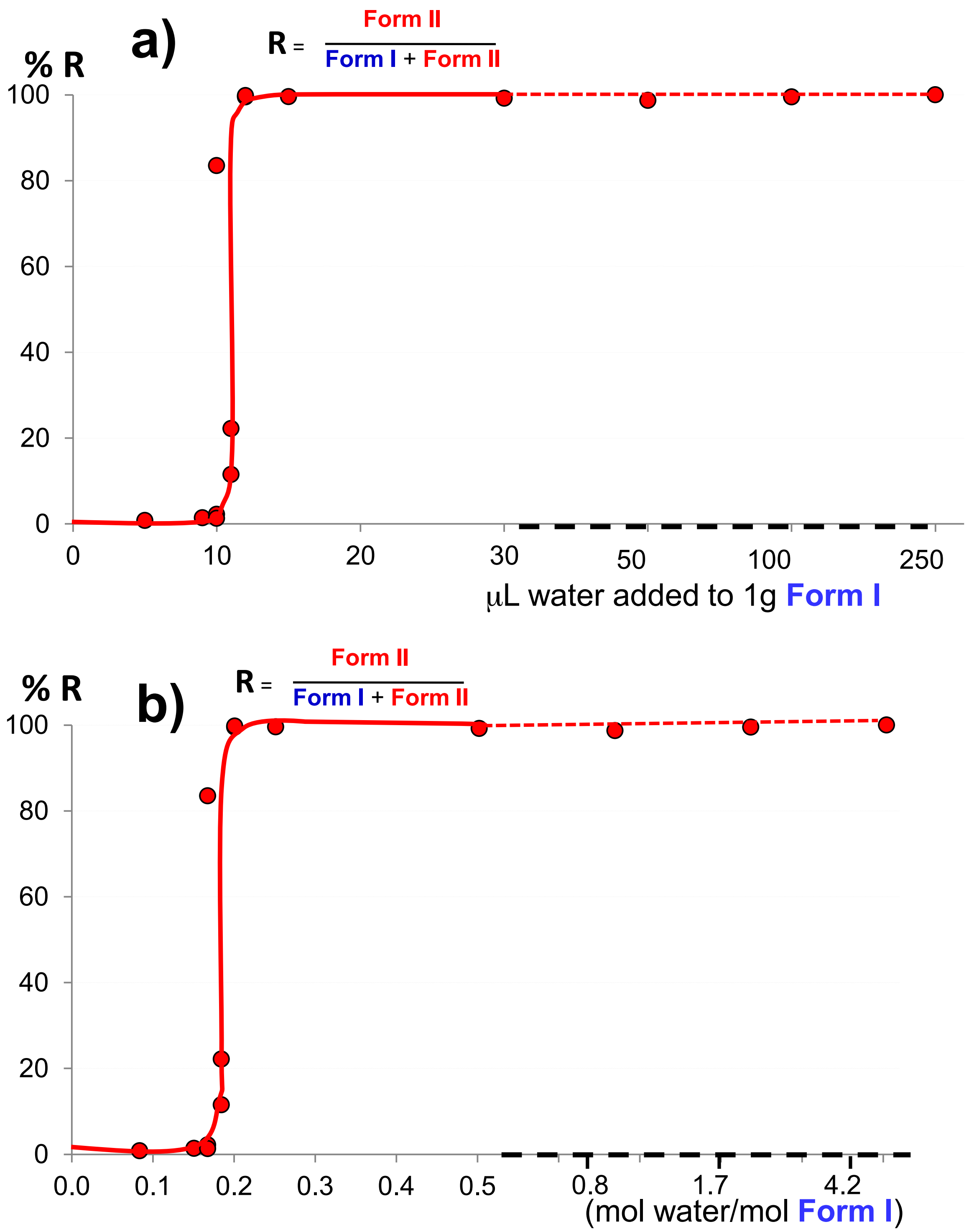

Figure S 106 Experimental milling curve obtained from the Rietveld refinement of PXRD data calculated as %R, R being the ratio between **Form II** and the total amount of 1:1 tp:bzm cocrystal = **Form I** +**Form II** versus a) μL water added to 1g **Form I** and b) mol water/mol total cocrystal. The Rietveld refinement of the PXRD data for these graphs is tabulated on Table S 145 and the PXRD spectra used for these graph are shown on in PXRD spectra 60.

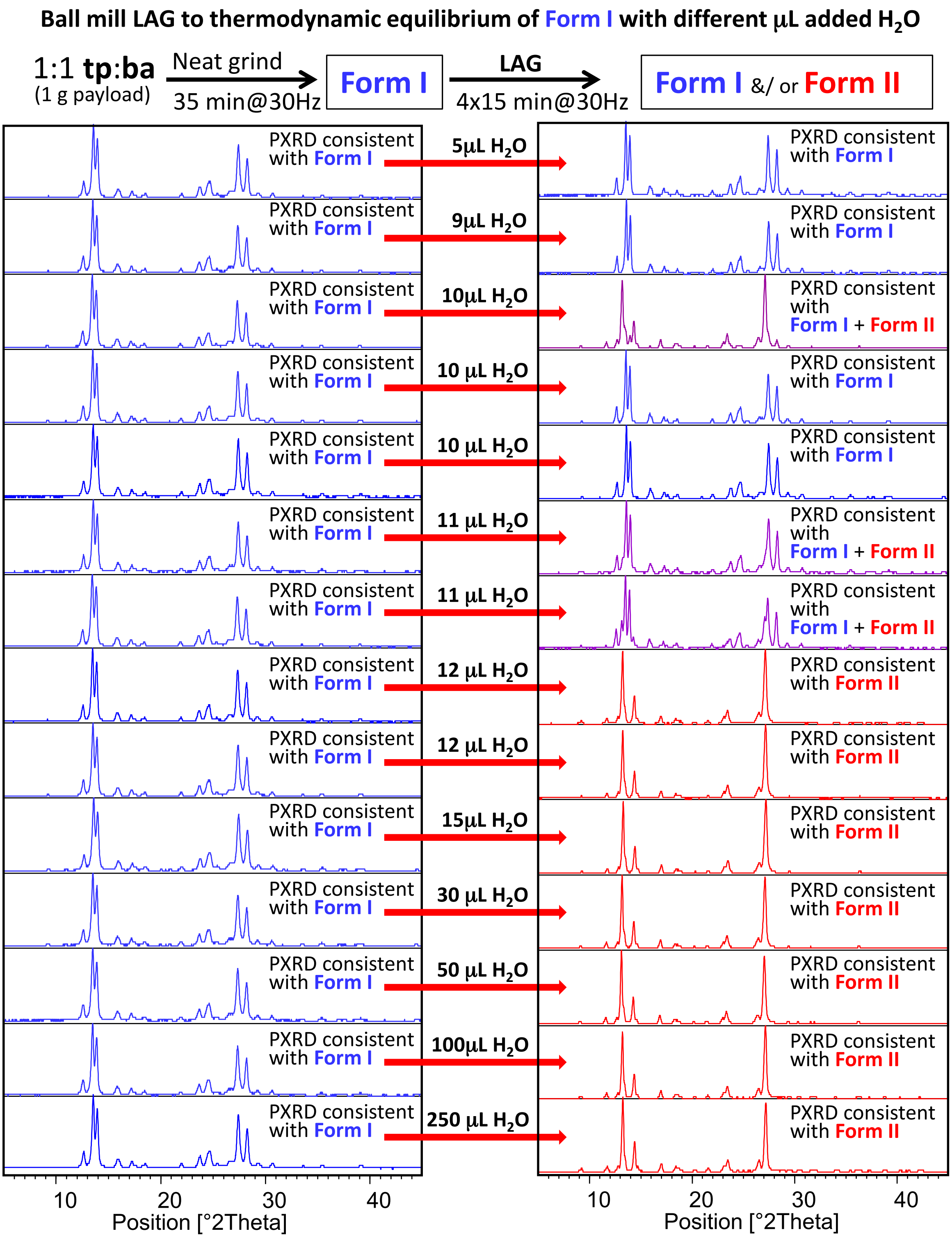

PXRD scan 60 Left: PXRD scan of **Form I** Starting material : Right: PXRD scan of the thermodynamic product of ball mill LAG starting from **Form I** with different volumes of water added to the grinding jar.

<u>Conclusion of this experiment:</u>

Form II is the thermodynamic product under ball mill LAG when ≥ 12 μL water is used to grind 1g of **Form I** with two 10 mm ID stainless steel ball bearing at 30 Hz. Below the addition of 9μL water, **Form I** is the thermodynamic product. Between 10 μL and 11 μL water added, the thermodynamic product is composed of a mixture of Form I and **Form II.**

14.10 Determination if Form I or Form II of 1:1 tp:bzm cocrystals is the thermodynamic product under slurry conditions

Objective of this experiment:

To determine whether **Form I** or **Form II** of the 1:1 tp:bzm cocrystals is the thermodynamic product at ambient temperature and pressure. An easy way to determine the thermodynamic form at ambient temperature and pressure is to prepare a slurry of a 1:1 mixture of **Form I** and **Form II** in a solvent. The metastable form will be consumed and transformed into the stable form, the phase composition at equilibrium being formed by just the stable polymorph. All solvents should lead to the same stable form, the kinetics of this polymorph transformation may be slower if the sample is not very soluble in the solvent selected.

Table S 146: Experimental conditions and phase composition of the outcome of the slurry experiment using 1:1 mixture of **Form I** and **Form II** in water. The phase composition of the product is obtained by Rietveld refinement of the PXRD scan.

Determining which Form of 1:1 tp:bzm is the thermodynamic product under slurry conditions								
**Slurry** experimental conditions	**Starting cocrystals** weighing (mg)			**Thermodynamic product of slurry experiment** Phase composition				
solvent	**Form I** 200 mg	**Form II** 200 mg		**Form I** %M	e.s.d. mol%	**Form II** %M	e.s.d. mol%
1 mL Water	200.88	200.34	→	1	0.3	99	0.3

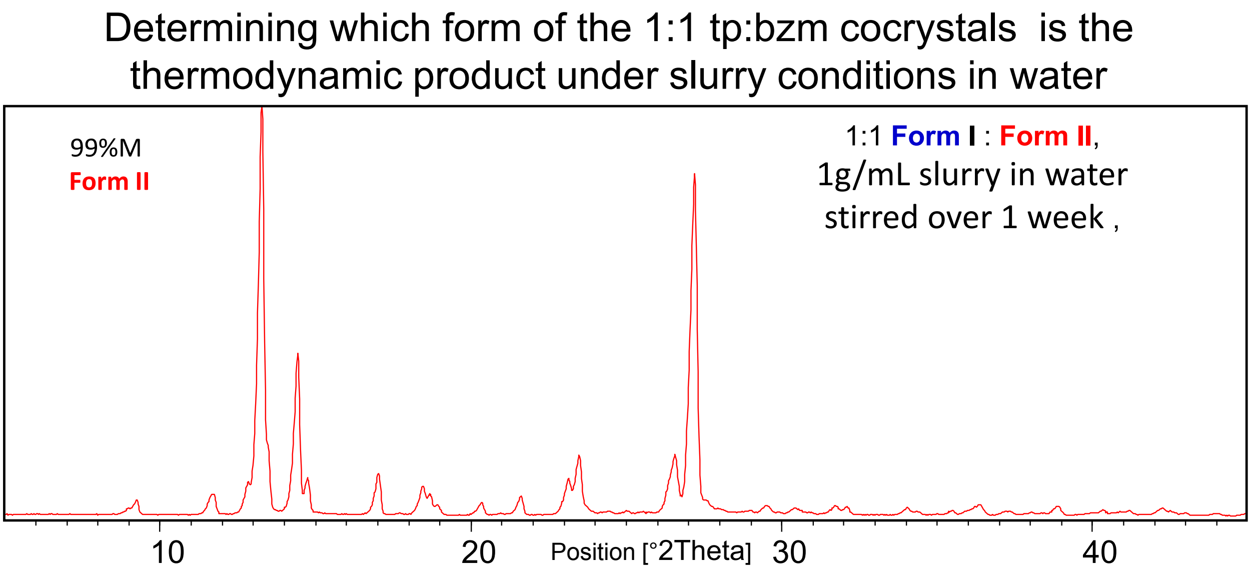

PXRD scan 61: PXRD scan of the dried solid filtrate of a slurry of a 1:1 mixture of **Form I** and **Form II** (1g/ml water) which had been continuously stirred over 1 week.

14.10.1 Conclusion of this experiment:

Form II is the thermodynamic product at ambient temperature and pressure. **Form I** is the metastable form.

15 Scanning Electron Microscopy (SEM)

SEM secondary electron analyses were performed on a FEI Quanta650F instrument operating at 2kV (spot size 1). Milled powder specimens were sprinkled through a mesh on a conductive tape on top of an aluminum stub and were gold coated for the analysis.

16 References

1. Groom, C. R., Bruno, I. J., Lightfoot, M. P. & Ward, S. C. The Cambridge Structural Database. *Acta Crystallogr. Sect. B Struct. Sci. Cryst. Eng. Mater.* **72,** 171–179 (2016).
2. Glidewell, C., Low, J. N. & Wardell, J. L. Conformational preferences and supramolecular aggregation in 2-nitrophenylthiolates: disulfides and thiosulfonates. *Acta Crystallogr. B* **56,** 893–905 (2000).
3. Belenguer, A. M., Friščić, T., Day, G. M. & Sanders, J. K. M. Solid-state dynamic combinatorial chemistry: reversibility and thermodynamic product selection in covalent mechanosynthesis. *Chem. Sci.* **2,** 696–700 (2011).
4. Perdew, J. P., Burke, K. & Ernzerhof, M. Generalized gradient approximation made simple. *Phys. Rev. Lett.* **77,** 3865–3868 (1996).
5. Blöchl, P. E. Projector augmented-wave method. *Phys. Rev. B* **50,** 17953 (1994).
6. Kresse, G. & Joubert, D. From ultrasoft pseudopotentials to the projector augmented-wave method. *Phys. Rev. B* **59,** 1758 (1999).
7. Grimme, S. Semiempirical GGA-type density functional constructed with a long-range dispersion correction. *J. Comput. Chem.* **27,** 1787–1799 (2006).
8. Kresse, G. & Hafner, J. Ab initio molecular dynamics for liquid metals. *Phys. Rev. B* **47,** 558 (1993).
9. Kresse, G. & Hafner, J. Ab initio molecular-dynamics simulation of the liquid-metal–amorphous-semiconductor transition in germanium. *Phys. Rev. B* **49,** 14251 (1994).
10. Kresse, G. & Furthmüller, J. Efficiency of ab-initio total energy calculations for metals and semiconductors using a plane-wave basis set. *Comput. Mater. Sci.* **6,** 15–50 (1996).
11. Kresse, G. & Furthmüller, J. Efficient iterative schemes for ab initio total-energy calculations using a plane-wave basis set. *Phys. Rev. B* **54,** 11169 (1996).
12. Monkhorst, H. J. & Pack, J. D. Special points for Brillouin-zone integrations. *Phys. Rev. B* **13,** 5188–5192 (1976).
13. Mathew, K., Sundararaman, R., Letchworth-Weaver, K., Arias, T. A. & Hennig, R. G. Implicit solvation model for density-functional study of nanocrystal surfaces and reaction pathways. *J. Chem. Phys.* **140,** 84106 (2014).
14. Mathew, K. & Hennig, R. G. Implicit self-consistent description of electrolyte in plane-wave density-functional theory. *ArXiv160103346 Cond-Mat* (2016).
15. Macrae, C. F. *et al.* Mercury CSD 2.0 – new features for the visualization and investigation of crystal structures. *J. Appl. Crystallogr.* **41,** 466–470 (2008).
16. Hartman, P. & Bennema, P. The attachment energy as a habit controlling factor. *J. Cryst. Growth* **49,** 145–156 (1980).
17. Mayo, S. L., Olafson, B. D. & Goddard, W. A. DREIDING: a generic force field for molecular simulations. *J. Phys. Chem.* **94,** 8897–8909 (1990).
18. Gasteiger, J. & Marsili, M. Iterative partial equalization of orbital electronegativity—a rapid access to atomic charges. *Tetrahedron* **36,** 3219–3228 (1980).
19. *Materials Studio*. (Biovia, 2004).
20. Belenguer, A. M.; Lampronti, G. I.; Wales, D. J.; Sanders, J. K. M. Direct Observation of Intermediates in a Thermodynamically Controlled Solid-State Dynamic Covalent Reaction. *Journal of the American Chemical Society*, **136**, 16156-16166 (2014).
21. Cougnon, F. B. L.; Sanders, J. K. M. *Accounts of Chemical Research*, **45**, 2211 (2011).
22. Black, S. P.; Sanders, J. K. M.; Stefankiewicz, A. R.: Disulfide exchange: exposing supramolecular reactivity through dynamic covalent chemistry. *Chemical Society Reviews*, **43**, 1861-1872 (2014).
23. Herrmann, A.: Dynamic combinatorial/covalent chemistry: a tool to read, generate and modulate the bioactivity of compounds and compound mixtures. *Chemical Society Reviews*, **43**, 1899-1933 (2014).
24. Jin, Y.; Yu, C.; Denman, R. J.; Zhang, W.: Recent advances in dynamic covalent chemistry. *Chemical Society Reviews*, **42**, 6634-6654 (2013).
25. Corbett, P. T.; Leclaire, J.; Vial, L.; West, K. R.; Wietor, J.-L.; Sanders, J. K. M.; Otto, S.: Dynamic Combinatorial Chemistry. *Chemical Reviews*, **106**, 3652-3711 (2006).

26. Fischer, F.; Heidrich, A.; Greiser, S.; Benemann, S.; Rademann, K.; Emmerling, F. Polymorphism of mechanochemically synthesized Cocrystals: a case study. *Crystal Growth & Design*, **16**, 1701-1707 (2016).

27. Cohelo, A. TOPAS-Academic, Coelho Software: Brisbane, Australia, (2007).

28. Cheary, R. W., Coelho, A., Cline, J. P.. Fundamental Parameters Line Profile Fitting in Laboratory Difiractometers. *Journal of Research of the National Institute of Standards and Technology*, **109**(1) (2004)

29. Madsen I.C. & Scarlett N.V.Y., "Quantitative Phase Analysis", in "Powder Diffraction – Theory and Practice", edited by Dinnabier R.E. & Billinge S.J.L., RCS publishing (2008).

30. Payzant, E. Andrew. "Other Topics." in Principles and Applications of Powder Diffraction: 365-380, edited by Clearfield, Abraham, Joseph Henry Reibenspies, and Nattamai Bhuvanesh. John Wiley and Sons, Ltd., (2008).

31. Black, D. R., Windover, D., Henins, A., Filliben, J., & Cline, J. P.. Certification of standard reference material 660B. *Powder Diffraction*, ***26***(02), 155-158 (2011).
32. Cheary, R. W., Coelho, A., Cline, J. P. (2004). Fundamental Parameters Line Profile Fitting in Laboratory Difiractometers. *Journal of Research of the National Institute of Standards and Technology*, ***109***(1).
33. A. M. Belenguer, G. I. Lampronti, D. J. Wales and J. K. M. Sanders, *Journal of the American Chemical Society*, 2014, **136**, 16156-16166. 33.
34. Fischer, M. U. Schmidt, S. Greiser and F. Emmerling, *Acta Crystallographica Section C: Structural Chemistry*, 2016, **72**, 217-224.
35. F. Fischer, A. Heidrich, S. Greiser, S. Benemann, K. Rademann and F. Emmerling, *Crystal Growth & Design*, 2016, **16**, 1701-1707.
